# Design, Synthesis, and *In Vitro* and *In Silico* Approaches of Novel Indanone Derivatives as Multifunctional Anti-Alzheimer Agents

**DOI:** 10.1021/acsomega.2c06906

**Published:** 2022-12-07

**Authors:** Begüm Nurpelin Sağlık, Serkan Levent, Derya Osmaniye, Asaf Evrim Evren, Abdullah Burak Karaduman, Yusuf Özkay, Zafer Asım Kaplancıklı

**Affiliations:** † Department of Pharmaceutical Chemistry, Faculty of Pharmacy, Anadolu University, 26470 Eskişehir, Turkey; ‡ Central Research Laboratory (MERLAB), Faculty of Pharmacy, Anadolu University, 26470 Eskişehir, Turkey; ⊥ Department of Pharmacy Services, Vocational School of Health Services, Bilecik Şeyh Edebali University, 11230 Bilecik, Turkey; ∥ Department of Pharmaceutical Toxicology, Faculty of Pharmacy, Anadolu University, 26470 Eskişehir, Turkey

## Abstract

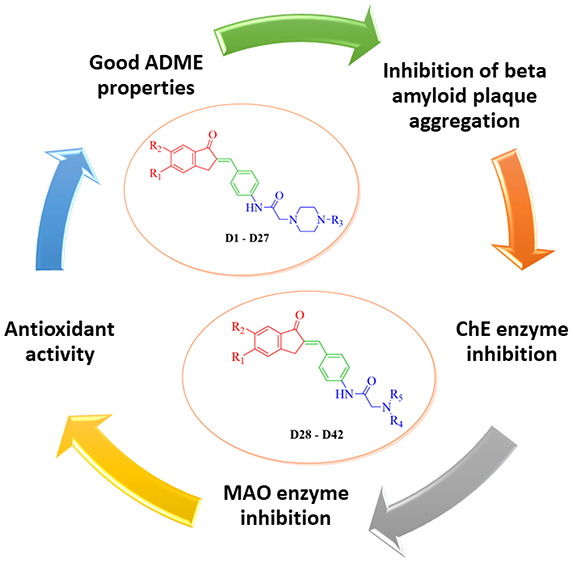

Alzheimer’s disease (AD) is a neurological, progressive illness that typically affects the elderly and is clinically distinguished by memory and cognitive decline. Due to a number of factors, including the absence of a radical treatment, an increase in the patient population over time, the high cost of care and treatment, and a significant decline in patients’ quality of life, the importance of this disease has increased. These factors have all prompted increased interest among researchers in this field. The chemical structure of the donepezil molecule, the most popular and effective treatment response for AD, served as the basis for the design and synthesis of 42 novel indan-1-one derivatives in this study. Using IR, ^1^H, and ^13^C NMR as well as mass spectroscopic techniques, the compounds’ structures were identified. Research on the compounds’ antioxidant activities, cholinesterase (ChE) enzyme inhibition, monoamine oxidase (MAO) A and B inhibitory activities, β-amyloid plaque inhibition, and cytotoxicity impact was carried out. Inhibition of β-amyloid plaque aggregation; effective inhibition of AChE, BChE, and MAO-B enzymes; and significant antioxidant activity were all demonstrated by compounds **D28**–**D30** and **D37**–**D39**. Because of their various actions, it was hypothesized that the related compounds may be useful in treating AD symptoms as well as providing palliative care.

## 1 Introduction

Alzheimer’s disease (AD), the most prevalent form of dementia and a lethal neurodegenerative condition marked by memory and cognitive problems, primarily affects the elderly.^1−3^ It is a disease of the neurological system that results in damage to the brain’s neurons and presents as a progressive loss of cognitive abilities, including speech, judgment, focus, and memory.^4^ There is currently no conclusive effective therapy for the progressive and cognitive dysfunction brought on by aging in AD, since the pathophysiology of these symptoms is still unknown.^5^ According to estimates, there are already 35 million AD sufferers globally. By 2030, that figure is predicted to rise to 65 million, and by 2050 it will reach 115 million.^6,7^ These numbers highlight the importance of developing an effective treatment.

The loss of cholinergic neurons in the basal frontal cortex, intracellular neurofibrillary tangles caused by τ-protein hyperphosphorylation, extracellular β-amyloid (Aβ) plaques, and oxidative stress are the most significant pathogenic characteristics of AD.^8,9^ Insufficient cholinergic transmission, which causes the emergence of cognitive, functional, and behavioral symptoms, is one cause that is given a significant role in AD. As a result, the cholinergic hypothesis postulates that increasing the insufficient level of acetylcholine in the brain will consequently inhibit cholinesterase enzymes.^10−12^ As a consequence, the goal of therapy is often to ameliorate cholinergic system dysfunction using either receptor agonists or cholinesterase (ChE) inhibitors.^13^

Currently, three FDA-approved drugs (donepezil, galantamine, and rivastigmine) that are based on the “one drug–one target” strategy treat symptoms by inhibiting AChE, demonstrating the shortcomings of this approach for the complex nature of AD. On the other hand, the most cutting-edge therapeutic approach now being used is based on the “one drug–multiple targets” strategy, which recommends using drugs with multiple actions at various target sites.^14,15^ A promising approach for treating complicated and multifaceted neurodegenerative illnesses is a multitarget directed ligand strategy that targets monoamine oxidase B (MAO-B).^16^ In AD, this strategy primarily uses dual inhibitors of MAO and acetylchoninesterase (AChE). In addition to their proven neuroprotective or neurorestorative and cognition-improving properties, MAO-B inhibitors have also been suggested to have therapeutic potential in AD. This is because they also have a favorable effect on monoaminergic transmission.^17^

**Figure 1:**
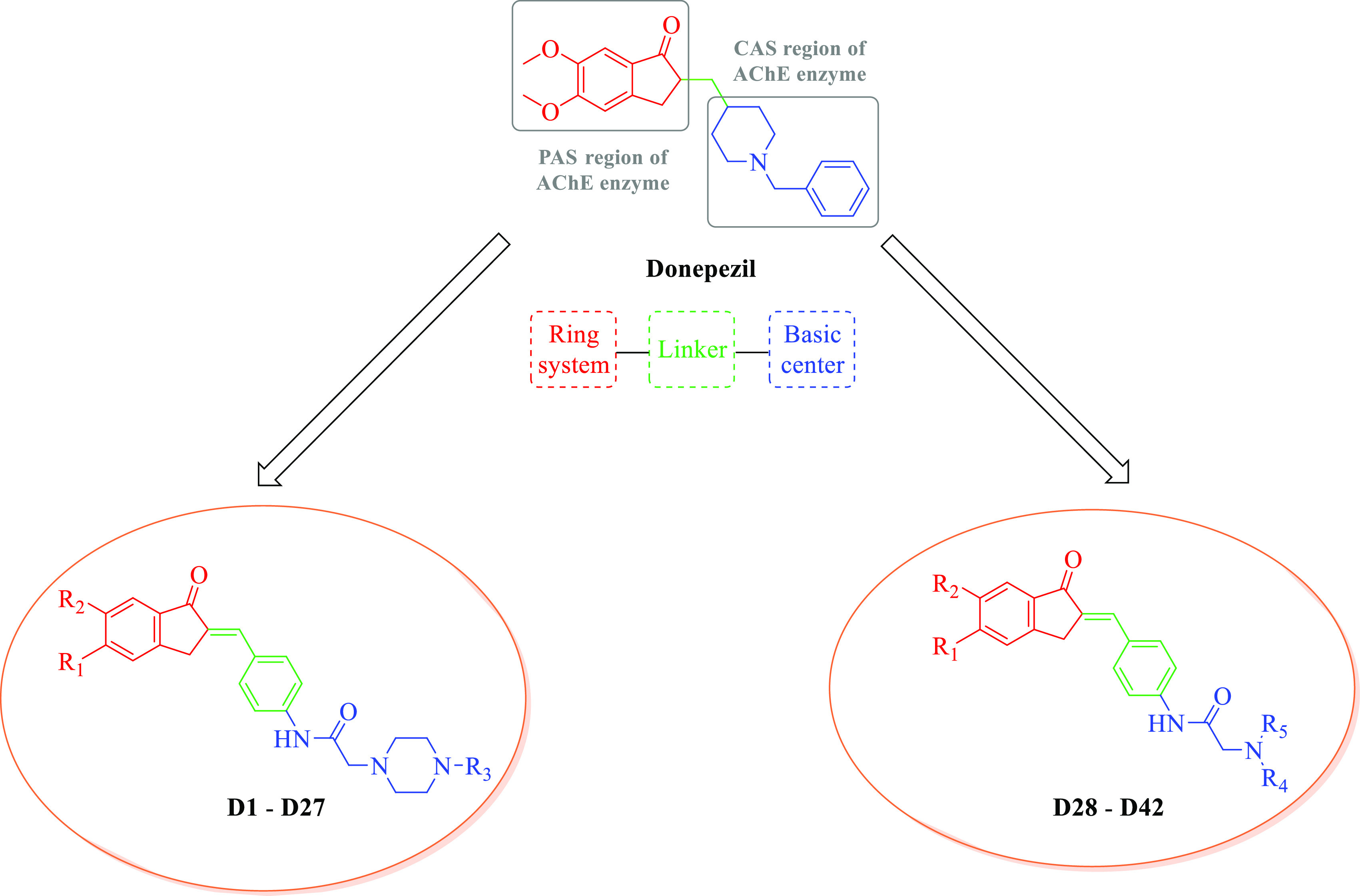
Design of the synthesized compounds (**D1**–**D42**).

Donepezil is the most frequently prescribed drug for treating all stages of AD and has the best treatment response. It produces a strong inhibition of AChE due to binding both the catalytic and peripheral anionic sites. This “double binding” feature should be taken into consideration when designing new drugs and treatment approaches. In this study, while taking into account the chemical structure of the donepezil molecule, we aimed to design (Figure 1), synthesize, and investigate the biological effects of new compounds containing piperazine and various secondary amine derivatives and to review relevant molecular modeling studies. In addition, the current literature indicates that the chalcones are associated with the selective inhibition of MAO-B.^14−16^ Therefore, target compounds were designed to include chalcones and the aim was to create selective activity against MAO-B. Thus, the aim was to create a potent multitarget directed ligand approach for the treatment of AD. It is common knowledge that preventing β-amyloid neurotoxicity during AD pathogenesis is crucial for palliative care. Therefore, in addition to investigating the pharmacokinetic activities of the proposed compounds, β-amyloid inhibitory capabilities were also examined. In order to prevent neurodegeneration and consequent neuronal death, oxidative stress reduction is also a key component of AD treatment.^12,15,16^ Oxidative stress is decreased by antioxidant compounds. Hence, the antioxidative capabilities of the designed compounds were also evaluated.

## 2 Experimental Section

### 2.1 Chemistry

All the substances utilized in the synthesis were sourced from Sigma-Aldrich (St. Louis, MO) or Merck (Darmstadt, Germany). The melting points of the obtained compounds were determined using MP90 computerized melting point equipment (Mettler Toledo, OH) and are displayed uncorrected. A Bruker 300 and 75 MHz digital FT-NMR spectrometer (Bruker Bioscience, Billerica, MA) was used to record the ^1^H NMR and ^13^C NMR spectra, respectively, in DMSO-*d*_6_. Additionally, 2D correlation techniques, HMBC, HSQC, and NOESY were performed with same instruments. The splitting patterns in the NMR spectra were identified and classified as follows: singlet (s), doublet (d), triplet (t), double doublet (dd), and multiplet (m). The coupling constants (*J*) are stated in hertz (Hz). On a Shimadzu LCMS-IT-TOF system (Kyoto, Japan), mass spectra were recorded using the electrospray ionization (ESI) technique. The purity of the molecules was examined using silica gel 60 F_254_ with thin-layer chromatography (Merck KGaA, Darmstadt, Germany). The synthesized compounds and their resulting spectra are shared in the “Analytical results of the compounds” part of the Supporting Information.

#### 2.1.1 General Procedure for the Synthesis of the Compounds

##### 2.1.1.1 Synthesis of 5-Methoxy-, 6-Methoxy-, and 5,6-Dimethoxy-2-(4-acetamidobenzylidene)-2,3-dihydro-1*H*-inden-1-one Derivatives (**A1**–**A3**)

Potassium hydroxide (100 mmol, 5.6 g) was dissolved in methanol. A suitable indan-1-one derivative (100 mmol) and 4-acetamidobenzaldehyde (100 mmol, 16.3 g) were added to the solution, and the mixture was allowed to stir at room temperature. The reaction mixture was stirred for 48 h, and the colored precipitate was filtered off. The crude product was dried and crystallized with ethanol.

##### 2.1.1.2 Synthesis of 5-Methoxy-, 6-Methoxy-, and 5,6-Dimethoxy-2-(4-aminobenzylidene)-2,3-dihydro-1*H*-inden-1-one Derivatives (**B1**–**B3**)

5-Methoxy-, 6-methoxy-, and 5,6-dimethoxy-2-(4-acetamidobenzylidene)-2,3-dihydro-1*H*-inden-1-one derivatives (**A1**–**A3**, respectively) (70 mmol) were dissolved in ethanol (50 mL), and HCl (10 mL) was added to the solution. The reaction mixture was boiled under reflux for 12 h, then subsequently added to ice water and neutralized with NH_3_. The precipitate was removed using a filter, rinsed with water, dried, and crystallized using ethanol.

##### 2.1.1.3 Synthesis of 2-Chloro-*N*-(4-((5-Methoxy-, 6-Methoxy-, and 5,6-Dimethoxy-1-oxo-2,3-dihydro-1*H*-inden-2-ylidene)methyl)phenyl)acetamide Derivatives (**C1**–**C3**)

The 5-methoxy-, 6-methoxy-, or 5,6-dimethoxy-2-(4-aminobenzylidene)-2,3-dihydro-1*H*-inden-1-one derivative (**B1**–**B3**, respectively) (30 mmol) was dissolved in 200 mL of tetrahydrofuran (THF), then the solution was placed in an ice bath on a magnetic heater and stirrer. Triethylamine (TEA) (33 mmol, 4.63 mL) was added to this solution. Chloroacetyl chloride (33 mmol, 2.63 mL) was dissolved in 10 mL of THF and placed into a dropping funnel. The chloroacetyl chloride solution was added dropwise to the 5-methoxy-, 6-methoxy-, or 5,6-dimethoxy-2-(4-aminobenzylidene)-2,3-dihydro-1*H*-inden-1-one derivative (**B1**–**B3**, respectively) solution under constant stirring. After the dripping process was complete, the reaction was continued for one more hour. After THF was removed under reduced pressure, ice water was added to the residue, and the solid product was filtered off. The crude product was washed with plenty of water, dried, and crystallized with ethanol.

##### 2.1.1.4 General Procedure for the Synthesis of Target Compounds (**D1**–**D42**)

The 2-chloro-*N*-(4-((5-methoxy-, 6-methoxy-, or 5,6-dimethoxy-1-oxo-2,3-dihydro-1*H*-inden-2-ylidene)methyl)phenyl)acetamide derivative (**C1**–**C3**, respectively) (1 mmol) and potassium carbonate (1 mmol, 0.138 g) were dissolved in acetone, and the appropriate piperazine or secondary amine derivative (1 mmol) was added to the solution. The reaction mixture was kept under reflux at 40 °C for 12 h. The end of the reaction was determined by TLC, and acetone was evaporated by placing an open container in a fume hood. The resulting residue was washed with water and filtered. The crude product was dried and crystallized with ethanol.

### 2.2 Biological Activity Studies

#### 2.2.1 *In Vitro* ChE Enzyme Inhibition Assay

According to the modified Ellman approach outlined in our team’s earlier work, the AChE and BChE inhibitory activities of the obtained compounds were evaluated.^18−21^ The enzymes utilized in the experiment were human AChE (CAS no. 9000-81-1) and human BChE (CAS no. 9001-08-5).

#### 2.2.2 *In Vitro* MAO Enzyme Inhibition Assay

The fluorometric approach, which was outlined in our team’s previously reported studies, was used to evaluate the synthesized compounds’ inhibitory effects against MAO-A and MAO-B.^22−25^ The enzymes employed in the experiment were recombinant human MAO-A and MAO-B enzymes.

#### 2.2.3 Enzyme Kinetics Studies of ChE and MAO Enzymes

The experimental procedures used in the kinetic investigations were identical to those used in the inhibition experiments. The molecules were utilized at the calculated value of 2 × IC_50_, IC_50_, and IC_50_/2, unlike the inhibition approach. Acetylthiocholine iodide (ATC) and butyrylthiocholine iodide (BTC) solutions were utilized as the substrates for the ChE enzymes, with serial dilutions at six different concentrations (600, 300, 150, 75, 37.5, and 18.75 μM). The tyramine substrate solution (concentrations ranging from 20 to 0.625 μM) was produced for the MAO enzymes at six different concentrations. Separate measurements were conducted both with and without an inhibitor. Using the Microsoft Office Excel 2013 program, the resulting absorbance values were compared to various substrate concentrations, and Lineweaver–Burk plots were created.^19−22^

#### 2.2.4 DPPH Free-Radical Scavenging Activity Assay

This approach is based on evaluating the free-radical scavenging abilities of the stable 1,1-diphenyl-2-picrylhydrazyl (DPPH) radical. To create the DPHH solution, 9.86 mg of DPPH was weighed and diluted to 25 mL with methanol. The test wells were then filled with 100 μL of the DPPH solution and 100 μL of the test solutions. Only 200 μL of methanol was used for the blank reading, while the control reading required the usage of 100 μL each of methanol and the DPPH solution. Following incubation, a spectrophotometric measurement at 517 nm was carried out.^26,27^

#### 2.2.5 Beta Amyloid 1–42 (Aβ42) Inhibitor Screening Assays

The Beta Amyloid 1–42 (Aβ42) Ligand Screening Assay kit (BioVision, Milpitas, CA) technique based on the fluorometric approach served as the basis for the experimental process.^21,28,29^

#### 2.2.6 Cytotoxicity Test

The cytotoxicity tests were performed using the NIH/3T3 mouse embryonic fibroblast cell line (ATCC CRL-1658, London, UK). The NIH/3T3 cells were incubated according to the manufacturer’s recommendations. In 96-well plates, NIH/3T3 cells were seeded at a density of 1 × 10^4^ cells per well. The MTT test was conducted in line with the previously mentioned standards.^19,22,23,28,30^

### 2.3 Prediction of ADME Parameters

Using the QikProp 4.8^31^ software and an *in silico* technique, the physicochemical properties of the produced compounds **D1**–**D42** were computed in order to predict the pharmacokinetic profiles of these compounds.

### 2.4 Molecular Docking Studies

The structure-based *in silico* docking method was applied to determine possible binding and interaction points of the synthesized compounds that were determined to be effective with the active sites of the relevant enzymes (**D19**–**D30** and **D34**–**D39** for AChE; **D34**, **D35**, and **D37**–**D39** for BChE; **D28** and **D29** for MAO-A; and **D28**–**D32** and **D37**–**D41** for MAO-B). For this purpose, protein–ligand interaction analysis was performed using the crystal structures of AChE (PDB code 4EY7),^32^ BChE (PDB code 4BDS),^33^ MAO-A (PDB code 2Z5X),^34^ and MAO-B (PDB code 2V5Z).^35^

The Protein Preparation Wizard procedure in LigPrep 3 was used to initially create the crystal structure for docking investigations.^36^ The OPLS 2005 force field was used to alter the bond lengths, and an automated determination of the potential charges of the atoms on the charged amino acids under the specified ambient conditions was produced. Using the LigPrep 3.8 tool,^37^ the compounds were prepared for molecular docking experiments. Glide 7.1^38^ was used to build the grids, and the single precision (SP) docking version of that module was used to do docking experiments.

### 2.5 Molecular Dynamics Simulations Studies

The molecular dynamics simulations (MDS) were performed using the Maestro Desmond interface program.^39^ All MDS were carried out for 100 ns to analyze the stability of the identified hits from the *in vitro* with docking results. System setup preparation, MDS, and interaction analysis calculations were carried out according to the same procedure from previous studies.^24,40^

## 3 Results and Discussions

### 3.1 Chemistry

**Scheme 1:**
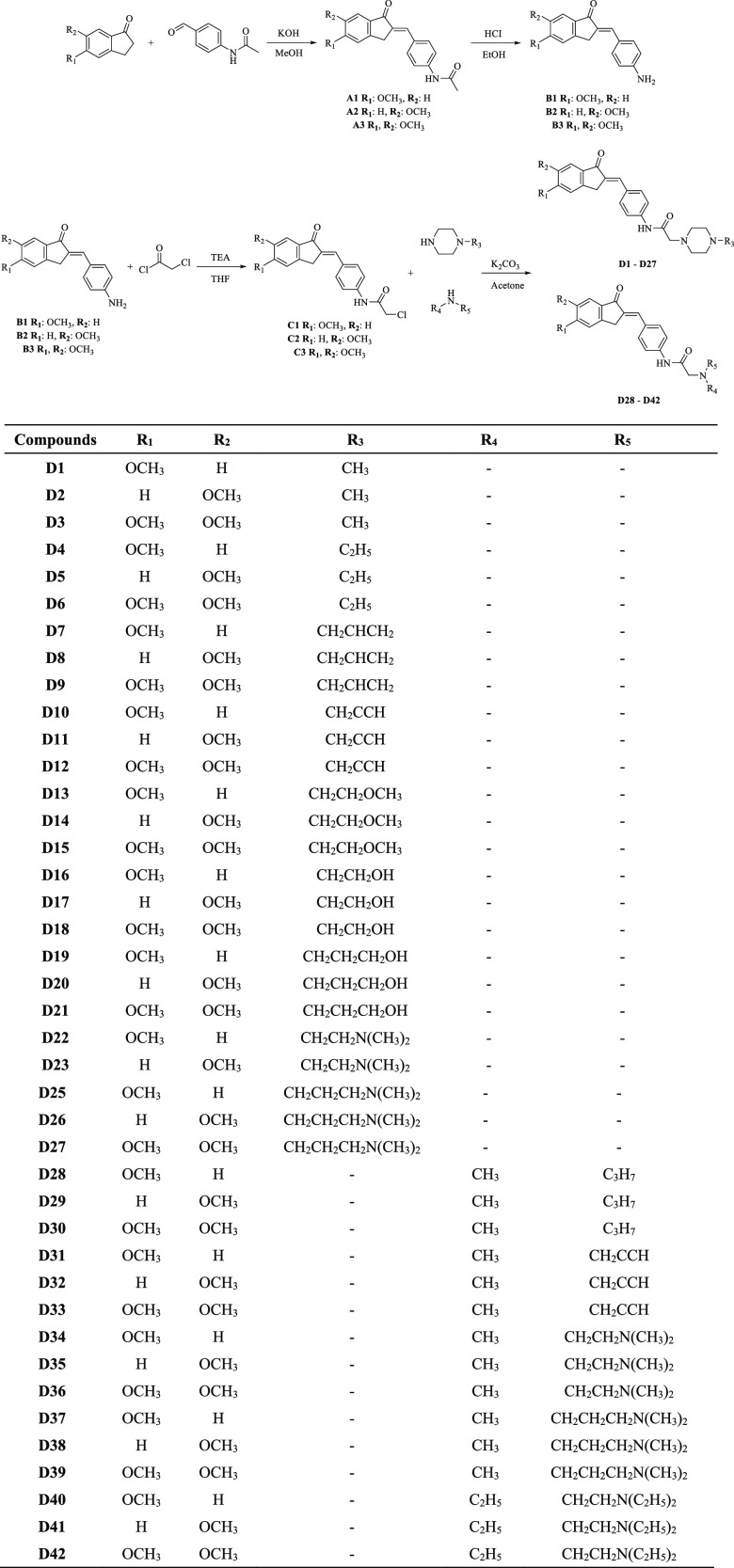
Synthesis Method for Compounds **D1**–**D42**

Syntheses were carried out in four stages (Scheme 1). In the first stage, 5-methoxy-, 6-methoxy-, and 5,6-dimethoxy-indan-1-one derivatives were subjected to potassium hydroxide-catalyzed Claisen Schmidt condensation with 4-acetamido benzaldehyde in methanol, resulting in 5-methoxy-, 6-methoxy-, and 5,6-dimethoxy-2-(4-acetamidobenzylidene)-2,3-dihydro-1*H*-inden-1-one (**A1**–**A3**, respectively) derivatives. Then, these derivatives were solved in ethanol and hydrolyzed with HCl, and 5-methoxy-, 6-methoxy-, and 5,6-dimethoxy-2-(4-aminobenzylidene)-2,3-dihidro-1*H*-inden-1-one (**B1**–**B3**, respectively) derivatives were obtained in the second stage of the reaction. In the third stage of the synthesis, 2-chloro-*N*-(4-((5-methoxy-, 6-methoxy-, and 5,6-dimethoxy-1-oxo-2,3-dihydro-1*H*-inden-2-ylidene)methyl)phenyl)acetamide (**C1**–**C3**) derivatives were obtained through the acetylation of 5-methoxy-, 6-methoxy-, and 5,6-dimethoxy-2-(4-aminobenzylidene)-2,3-dihydro-1*H*-inden-1-one (**B1**–**B3**, respectively) derivatives with chloroacetyl chloride in tetrahydrofuran. In the final stage of the reaction, 4-substituted piperazine-1-yl and substituted secondary amine derivatives were subjected to substitution reaction with **C1**–**C3** in acetone with potassium carbonate, resulting in the synthesis of the target compounds, namely, 2-substituted *N*-(4-((5-methoxy-, 6-methoxy-, and 5,6-dimethoxy-1-oxo-2,3-dihydro-1*H*-inden-2-yliden)methyl)phenyl)acetamides (**D1**–**D42**).

Upon examination of the chemical structures of the synthesized compounds, the presence of a 1-indanone ring was observed as common in all compounds. The specific stretching band of carbonyl (C=O) on this group was observed within the range of 1664–1749 cm^–1^ in IR spectroscopy. In addition to 1-indanone carbonyl, in all compounds, the specific stretching band of carbonyl (C=O) in the acetamide (−NHCOCH_2_−) functional group was found in the range of 1585–1683 cm^–1^. The N–H stretching band on the acetamide group was observed within the 3194–3417 cm^–1^ range in the spectra. The data for these stretching bands observed in the spectra were consistent with the literature data.^41^

The details of the ^1^H NMR results of the derivatives with methoxy groups at the fifth position of the indanone ring were as follows: The methoxy protons observed at 3.90 ppm in the starting substance did not have any significant effect on the electronic periphery due to the distance to vaious substituents and generated a peak at 3.89 or 3.90 ppm. The methylene protons in the indanone ring were also observed between 4.04 and 4.06 ppm. The aromatic protons at the fourth, sixth, and seventh positions of the ring were mostly doublet, doublet of doublet, and doublet, respectively, and sometimes multiplets due to engaging with other protons. The chemical shift values of these were 7.17–7.18, 7.02–7.03, and 7.70–7.73 ppm, respectively. The hydrogens belonging to the disubstituted benzene ring were observed at 7.70 to 7.79 ppm. When the methoxy group shifted from the fifth position to the sixth position, protons were observed between 3.82 and 3.84 ppm. Similarly, the methylene protons in the ring were detected to be between 3.99 and 4.02 ppm without any significant change. The aromatic protons in the indanone ring generated peaks were doublet, doublet of doublet, and doublet, as in the previous derivatives. The chemical shift values of the protons at the fourth, fifth, and seventh positions were, respectively, 7.54–7.58, 7.27–7.29, and 7.22–7.25 ppm. The alkene hydrogen bonded to indanone was observed as a singlet at 7.46–7.48 ppm. The hydrogens of the disubstituted benzene ring were found as two doublets from 7.70 to 7.79 ppm.

The values of methoxy at the fifth and sixth positions of the final main structure were determined with two-dimensional NMR spectroscopy, where the fifth position was in a slightly higher area. The fifth position was found around 3.83 ppm, while the methoxy in the sixth position was observed to be around 3.90 ppm. The aromatic protons in indanone were found as two singlets around 7.20 ppm, while aliphatic methylene protons were observed as singlets at 3.97–3.98 ppm. In addition, amide protons in all of the derivatives were found around 10 ppm. All the other aliphatic and aromatic proton peaks of substituted piperazine and amine derivatives that remained out of the common chemical structure and were present as the variable group were observed within the typical areas, as expected.

The common structural particles in the synthesized compounds generated peaks in the ^13^C NMR spectra, as expected. The total carbon counts were determined by taking those that were identical in terms of electronic periphery, and the number of peaks observed was as expected. Amide and ketone carbonyls (C=O) among the specific functional groups were observed within the ranges of 155.00–171.40 and 191.90–193.60 ppm, respectively, which was consistent with the literature data.^42^ Excluding these generated peaks, the aliphatic carbons were within the range of 11.70–79.90 ppm, while aromatic carbons generated peaks within the range of 104.80–165.30 ppm, which was consistent with the literature data.

Two-dimensional NMR (HSQC and HMBC) was carried out in order to determine and match the areas of all H and C atoms within the chemical structures of the synthesized compounds. For this purpose, **C1**–**C3** compounds that were obtained in the third stage of the synthesis pathway and were present as common in structures of all the test compounds as the target compounds were derived from those analyzed with HSQC and HMBC techniques. Additionally, using the NOESY technique, the isomer type (*E*/*Z*) was determined. It was detected that the synthesized compounds were *E*-isomers because of the observation of the correlation between the protons of 10th and 13rd positions in the structure. If there was a correlation between the protons of 11th and 13rd positions, it could be said that the compounds had *Z*-isomers. However, as can be seen from the relevant spectra, no such correlation was detected. Therefore, it was clearly decided that the synthesized compounds were *E*-isomers. Details of HSQC, HMBC, and NOESY techniques are shared in the Supporting Information. All *in silico* studies applied in this paper were carried out in accordance with this finding (as the *E*-isomer).

The mass spectra of the synthesized compounds were compiled with the positive ionization technique using the electron spray method. It was clearly seen that the molecular weights of the compounds were consistent with the [M + H]^+^ peaks obtained. Additionally, [M + 2H]^2+^ peaks that were observed in compounds **D1**–**D27** due to the nitrogen atoms of the piperazine ring were detected as the half values of the main peak (all the spectra of the synthesized compounds are shared in the Supporting Information).

### 3.2 Evaluation of the Biological Activity Studies

#### 3.2.1 ChE Enzyme Inhibition

It was observed from the AChE enzyme inhibition results that the vast majority of the compounds in the series demonstrated a high rate of activity at a concentration of 10^–3^ M. At this concentration, compounds **D1**–**D4**, **D6**–**D13**, **D15**–**D30**, and **D33**–**D42** had inhibition rates higher than 50%. Among these, compounds **D3**, **D6**, **D12**, **D15**, **D18**–**D30**, **D34**–**D39**, and **D42** demonstrated quite strong AChE enzyme inhibition profiles by generating at least 90% activity. The second step of the enzyme activity test was reserved for compounds **D19**–**D30** and **D34**–**D39**, since they displayed more than 50% inhibition effects at a dosage of 10^–4^ M. At levels of 10^–3^ and 10^–4^, respectively, the reference drug donepezil exhibited inhibitory action at rates of 99.156 ± 1.302% and 97.395 ± 1.255%. For the second step of the AChE enzyme inhibition experiment, further concentrations of the chosen compounds were generated using serial dilution (Supporting Information Table S1).

The results of BChE enzyme activity revealed that all derivatives, excluding compound **D10**, had activities higher than 50% at a concentration of 10^–3^ M. When compared with the results obtained in the AChE enzyme inhibition test at this concentration, almost all the compounds in the series showed more selective inhibition against the BChE enzyme. However, when the results of the activity at a concentration of 10^–4^ M were analyzed, this selectivity was likely to end because only compounds **D34**, **D35**, and **D37**–**D39** showed inhibition higher than 50% at the latter concentration. The reference compound tacrine had inhibitory activity at rates of 99.827 ± 1.378% and 98.651 ± 1.402% at concentrations of 10^–3^ and 10^–4^ M, respectively (Supporting Information Table S1). Tacrine and compounds **D34**, **D35**, and **D37**–**D39** were selected for the second stage of the BChE enzyme inhibition test.

The IC_50_ value of donepezil was 0.0201 ± 0.0001 μM on the AChE enzyme, while the IC_50_ values of compounds **D19**–**D30** and **D34**–**D39** were in the range of 0.0224 ± 0.0008 and 0.2347 ± 0.0113 μM. Among these compounds, **D28**–**D30** were found to have the strongest AChE inhibition with the lowest IC_50_ values. The IC_50_ values of compounds **D28**, **D29**, and **D30** were calculated as 0.0248 ± 0.0010, 0.0224 ± 0.0008, and 0.0257 ± 0.0009 μM, respectively (Table 1). These compounds demonstrated inhibition profiles with IC_50_ values very close to that of donepezil.

**Table 1:** IC_50_ Values of the Selected Compounds, Donepezil, and Tacrine against AChE and BChE

	human AChE inhibition (%)							
compounds	10^–3^ M	10^–4^ M	10^–5^ M	10^–6^ M	10^–7^ M	10^–8^ M	10^–9^ M	IC_50_ (μM)
**D19**	95.234 ± 1.652	92.743 ± 1.253	89.186 ± 1.085	82.147 ± 0.988	70.698 ± 1.075	33.624 ± 0.624	20.593 ± 0.596	0.0324 ± 0.0012
**D20**	98.265 ± 1.112	91.115 ± 1.365	86.270 ± 1.249	77.151 ± 0.975	68.264 ± 0.633	40.196 ± 0.528	21.089 ± 0.429	0.0311 ± 0.0011
**D21**	95.659 ± 1.198	91.743 ± 1.466	87.251 ± 1.298	81.790 ± 1.098	70.626 ± 1.004	38.271 ± 0.678	19.285 ± 0.593	0.0292 ± 0.0008
**D22**	91.897 ± 1.468	86.624 ± 1.567	78.991 ± 1.165	70.412 ± 1.075	63.360 ± 0.988	42.985 ± 0.633	18.756 ± 0.521	0.0527 ± 0.0016
**D23**	92.458 ± 1.568	87.634 ± 1.677	82.446 ± 1.206	70.113 ± 1.063	68.927 ± 0.945	35.468 ± 0.518	20.646 ± 0.418	0.0510 ± 0.0014
**D24**	93.224 ± 1.719	90.486 ± 1.298	82.415 ± 1.102	72.846 ± 1.267	63.963 ± 1.088	41.281 ± 0.623	18.773 ± 0.411	0.0469 ± 0.0019
**D25**	92.555 ± 1.623	88.713 ± 1.185	71.067 ± 1.014	58.928 ± 0.952	43.220 ± 0.548	35.281 ± 0.417	23.416 ± 0.389	0.1826 ± 0.0074
**D26**	93.294 ± 1.498	80.485 ± 1.208	78.128 ± 1.018	60.629 ± 0.974	40.281 ± 0.598	35.642 ± 0.758	24.486 ± 0.622	0.1763 ± 0.0062
**D27**	91.220 ± 1.298	87.293 ± 1.118	82.141 ± 1.026	73.266 ± 0.994	41.223 ± 0.562	31.625 ± 0.389	22.488 ± 0.419	0.1305 ± 0.0042
**D28**	**98.627 ± 1.207**	**95.284 ± 1.311**	**92.778 ± 1.026**	**87.149 ± 1.058**	**72.022 ± 0.958**	**35.961 ± 0.623**	**19.450 ± 0.455**	**0.0248 ± 0.0010**
**D29**	**97.184 ± 1.318**	**94.285 ± 1.458**	**91.758 ± 1.205**	**88.269 ± 1.017**	**75.558 ± 0.974**	**34.289 ± 0.857**	**20.115 ± 0.357**	**0.0224 ± 0.0008**
**D30**	**98.211 ± 1.366**	**96.552 ± 1.285**	**91.783 ± 1.208**	**88.266 ± 1.104**	**74.285 ± 1.052**	**36.588 ± 0.627**	**16.447 ± 0.421**	**0.0257 ± 0.0009**
**D34**	98.265 ± 1.112	91.115 ± 1.365	75.964 ± 1.119	63.446 ± 0.985	41.213 ± 0.528	32.472 ± 0.588	23.471 ± 0.429	0.1911 ± 0.0087
**D35**	95.659 ± 1.198	91.743 ± 1.466	73.197 ± 1.116	61.759 ± 1.085	42.872 ± 0.628	33.640 ± 0.520	21.483 ± 0.448	0.2150 ± 0.0088
**D36**	91.897 ± 1.468	86.624 ± 1.567	78.629 ± 1.028	58.757 ± 0.893	38.195 ± 0.539	33.123 ± 0.491	20.615 ± 0.374	0.2347 ± 0.0113
**D37**	92.458 ± 1.568	87.634 ± 1.677	83.474 ± 1.229	75.115 ± 1.076	61.476 ± 0.965	39.522 ± 0.489	19.740 ± 0.508	0.0482 ± 0.0017
**D38**	93.224 ± 1.719	90.486 ± 1.298	81.649 ± 1.174	73.998 ± 0.956	68.182 ± 0.862	37.216 ± 0.562	19.713 ± 0.711	0.0473 ± 0.0016
**D39**	92.555 ± 1.623	88.713 ± 1.185	81.661 ± 1.156	73.281 ± 0.952	64.264 ± 0.687	40.199 ± 0.528	20.482 ± 0.492	0.0458 ± 0.0009
donepezil	99.156 ± 1.302	97.395 ± 1.255	93.583 ± 1.167	91.277 ± 1.074	76.982 ± 0.951	35.459 ± 0.453	18.410 ± 0.411	0.0201 ± 0.0001

	human BChE inhibition (%)							
compounds	10^–3^M	10^–4^M	10^–5^M	10^–6^M	10^–7^M	10^–8^M	10^–9^M	IC_50_ (μM)
**D34**	91.635 ± 1.028	84.778 ± 1.249	76.288 ± 1.136	71.886 ± 1.007	49.668 ± 0.856	31.472 ± 0.405	20.451 ± 0.399	0.1323 ± 0.0051
**D35**	90.168 ± 1.388	81.242 ± 1.245	74.627 ± 1.128	65.884 ± 1.007	54.926 ± 0.974	33.415 ± 0.814	17.994 ± 0.411	0.1505 ± 0.0048
**D37**	**94.227 ± 1.391**	**89.716 ± 1.247**	**82.699 ± 1.007**	**71.392 ± 1.348**	**59.282 ± 0.754**	**32.478 ± 0.355**	**18.477 ± 0.471**	**0.0839 ± 0.0034**
**D38**	**93.266 ± 1.374**	**90.553 ± 1.299**	**85.415 ± 1.301**	**78.621 ± 1.114**	**58.663 ± 0.952**	**28.779 ± 0.714**	**18.417 ± 0.633**	**0.0782 ± 0.0029**
**D39**	**92.759 ± 1.365**	**87.290 ± 1.247**	**81.445 ± 1.331**	**73.693 ± 1.148**	**55.411 ± 0.678**	**34.666 ± 0.511**	**21.642 ± 0.485**	**0.0750 ± 0.0032**
tacrine	99.827 ± 1.378	98.651 ± 1.402	96.282 ± 1.399	92.487 ± 1.125	82.614 ± 1.058	42.753 ± 0.794	26.536 ± 0.633	0.0064 ± 0.0002

Tacrine acted as a BChE enzyme inhibitor, with an IC_50_ value of 0.0064 ± 0.0002 μM. The IC_50_ values of compounds **D34** and **D35** were 0.1323 ± 0.0051 and 0.1505 ± 0.0048 μM, respectively. These compounds were found to have lower inhibition profiles compared to tacrine. Among the selected compounds, derivatives **D37**–**D39** were the most effective derivatives against the BChE enzyme with low IC_50_ values. The IC_50_ values of these compounds were 0.0839 ± 0.0034, 0.0782 ± 0.0029, and 0.0750 ± 0.0032 μM, respectively (Table 1). These compounds demonstrated lower BChE enzyme activities compared to tacrine despite having similar IC_50_ values.

#### 3.2.2 MAO Enzyme Inhibition

It was understood from the enzyme activity results of the MAO-A enzyme that most of the compounds (**D1**, **D3**–**D8**, **D11**–**D13**, and **D15**–**D42**) had inhibition activities higher than 50% at a concentration of 10^–3^ M. The reference compound moclobemide showed activity at a rate of 94.121 ± 2.760% at the same concentration. The compounds that showed at least 90% inhibition at this concentration, such as moclobemide, were **D6**, **D12**, **D21**, **D26**, **D27**, **D29**, **D31**, **D37**, **D38**, and **D42**. When a concentration of 10^–4^ M was examined in the enzyme activity results, moclobemide showed activity at the rate of 82.143 ± 2.691%, while only compounds **D28** and **D29** displayed inhibition higher than 50% and were selected for the second stage of the enzyme inhibition study on the MAO-A enzyme (Supporting Information Table S2).

The reference drug selegiline had an inhibition rate of 98.589 ± 2.055% at a concentration of 10^–3^ M when the MAO-B enzyme activity findings were analyzed. At this dosage, every synthesized molecule had greater than 50% activity and, when compared to the MAO-A enzyme, exhibited a selective effect on the MAO-B enzyme. Among these compounds, **D6**, **D12**, **D19**, **D21**, **D26**–**D32**, **D35**, and **D37**–**D42** had at least 90% activity at this concentration. When the rates at a concentration of 10^–4^ M were investigated, compounds **D28**–**D32** and **D37**–**D41** exceeded 50% inhibitory activity. On the other hand, selegiline had a 94.850 ± 1.114% inhibition effect at this concentration (Supporting Information Table S2). Selegiline and compounds **D28**–**D32** and **D37**–**D41** were selected for the second stage of the MAO-B enzyme inhibition test.

The IC_50_ value of moclobemide on the MAO-A enzyme was calculated as 6.0613 ± 0.262 μM. The IC_50_ values of the selected compounds, namely, **D28** and **D29**, were, respectively, 0.1108 ± 0.0047 and 0.1116 ± 0.0042 μM. This data showed that compounds **D28** and **D29** were the most effective derivatives against the MAO-A enzyme, and these compounds had 50× stronger inhibition effects when compared to moclobemide (Table 2).

**Table 2:** IC_50_ Values of the Selected Compounds, Moclobemide, and Selegiline against MAO-A and MAO-B

	human MAO-A inhibition (%)							
compounds	10^–3^ M	10^–4^ M	10^–5^ M	10^–6^ M	10^–7^ M	10^–8^ M	10^–9^ M	IC_50_ (μM)
**D28**	**89.622 ± 1.288**	**81.755 ± 1.305**	**72.658 ± 1.114**	**63.449 ± 1.085**	**58.995 ± 0.895**	**39.186 ± 0.714**	**18.744 ± 0.521**	**0.1108 ± 0.0047**
**D29**	**90.224 ± 1.358**	**84.633 ± 1.247**	**77.417 ± 1.126**	**67.298 ± 1.084**	**49.422 ± 0.567**	**33.993 ± 0.422**	**24.647 ± 0.328**	**0.1116 ± 0.0042**
moclobemide	94.121 ± 2.760	82.143 ± 2.691	60.458 ± 2.559	36.151 ± 1.984	22.135 ± 1.337	18.166 ± 0.812	14.128 ± 0.725	6.0613 ± 0.262

	human MAO-B inhibition (%)							
compounds	10^–3^ M	10^–4^ M	10^–5^ M	10^–6^ M	10^–7^ M	10^–8^ M	10^–9^ M	IC_50_ (μM)
**D28**	**92.551 ± 1.334**	**89.261 ± 1.241**	**80.474 ± 1.015**	**75.198 ± 1.078**	**66.984 ± 0.958**	**41.454 ± 0.547**	**18.774 ± 0.421**	**0.0409 ± 0.0019**
**D29**	**91.822 ± 1.368**	**87.593 ± 1.249**	**82.171 ± 1.211**	**74.229 ± 1.302**	**65.991 ± 1.842**	**40.287 ± 0.745**	**20.445 ± 0.628**	**0.0412 ± 0.0018**
**D30**	**97.513 ± 1.388**	**90.225 ± 1.471**	**82.456 ± 1.378**	**74.626 ± 1.288**	**65.979 ± 0.966**	**38.111 ± 0.458**	**19.753 ± 0.632**	**0.0456 ± 0.0017**
**D31**	93.568 ± 1.477	88.717 ± 1.356	81.483 ± 1.244	75.944 ± 1.058	61.928 ± 1.014	35.478 ± 0.485	18.775 ± 0.511	0.0619 ± 0.0028
**D32**	94.777 ± 1.421	89.638 ± 1.266	81.482 ± 1.805	72.663 ± 1.324	59.585 ± 0.977	34.199 ± 0.514	21.476 ± 0.429	0.0665 ± 0.0024
**D37**	**98.398 ± 1.629**	**91.559 ± 1.238**	**85.497 ± 1.118**	**78.261 ± 1.074**	**72.182 ± 1.099**	**40.115 ± 0.561**	**18.247 ± 0.418**	**0.0312 ± 0.0008**
**D38**	**94.796 ± 1.429**	**88.636 ± 1.375**	**84.555 ± 1.582**	**78.197 ± 1.407**	**62.490 ± 0.925**	**42.286 ± 0.623**	**20.744 ± 0.318**	**0.0359 ± 0.0013**
**D39**	**95.294 ± 1.059**	**87.214 ± 1.821**	**84.774 ± 1.637**	**78.638 ± 1.248**	**60.223 ± 0.956**	**36.193 ± 0.488**	**25.623 ± 0.359**	**0.0393 ± 0.0011**
**D40**	92.88/ ± 1.236	88.757 ± 1.301	80.622 ± 1.475	65.215 ± 0.975	43.136 ± 0.662	25.742 ± 0.425	19.881 ± 0.365	0.2030 ± 0.0098
**D41**	90.177 ± 1.365	81.794 ± 1.288	73.287 ± 1.418	65.423 ± 0.975	42.214 ± 0.563	32.664 ± 0.427	21.484 ± 0.385	0.2089 ± 0.0095
selegiline	98.589 ± 2.055	94.850 ± 1.114	87.412 ± 1.028	79.558 ± 1.057	66.248 ± 1.112	43.015 ± 0.958	15.107 ± 0.340	0.0374 ± 0.0016

The IC_50_ value of selegiline was 0.0374 ± 0.0016 μM in the second stage of the MAO-B enzyme activity assay. Compounds **D40** and **D41** had the weakest MAO-B inhibition effects among the selected compounds, with IC_50_ values of 0.2030 ± 0.0098 and 0.2089 ± 0.0095 μM, respectively. These compounds were followed by compounds **D31** and **D32**, with IC_50_ values of 0.0619 ± 0.0028 and 0.0665 ± 0.0024 μM, respectively. Meanwhile, derivatives **D28**, **D29**, and **D30** demonstrated MAO-B inhibition effects very close to that of selegiline, with enzyme activity values of 0.0409 ± 0.0019, 0.0412 ± 0.0018, and 0.0456 ± 0.0017 μM, respectively. These compounds were also the most effective against the AChE enzyme. Therefore, compounds **D28**, **D29**, and **D30** are effective on both the AChE and MAO-B enzymes and are highly important compounds in the treatment of AD. Among the selected compounds, **D37**, **D38**, and **D39** were the most effective on the MAO-B enzyme, with IC_50_ values of 0.0312 ± 0.0008, 0.0359 ± 0.0013, and 0.0393 ± 0.0011 μM, respectively. Compound **D39** had an inhibition effect very similar to that of selegiline. Compounds **D37** and **D38** had higher MAO-B enzyme inhibition effects when compared to selegiline (IC_50_ value of 0.0374 ± 0.0016 μM), with IC_50_ values of 0.0312 ± 0.0008 and 0.0359 ± 0.0013 μM, respectively (Table 2).

#### 3.2.3 Enzyme Kinetics

The absorbance data from the experiments and the substrate concentrations were used to generate Lineweaver–Burk plots. The graphic’s *x*-axis stands for 1/[S] (1/substrate concentration), while the *y*-axis displays values for 1/*V*, which stands for 1/absorbance. Four lines for enzyme kinetic assays at the 2 × IC_50_, IC_50_, IC_50_/2, and control group (i.e., without an inhibitor) doses of the test substances are shown in the graphics. The intersection of these four lines on the diagram was used to define the type of substrate and inhibitor reaction against the enzyme.

Reversible and irreversible inhibition of enzymes are the two categories that inhibition is commonly subdivided into. In irreversible inhibition, the inhibitor either creates a recalcitrant complex structure or establishes a covalent bond with the enzyme. The category of reversible inhibition is further subdivided into mixed, competitive, noncompetitive, and uncompetitive types. In Lineweaver–Burk graphs, the kind of inhibition is classified as uncompetitive if four lines are parallel, competitive if they intersect on the *y*-axis, noncompetitive if they do so on the *x*-axis, and mixed if they cross in any of the graphic’s regions but not on any of its axes.^19−22^

**Figure 2:**
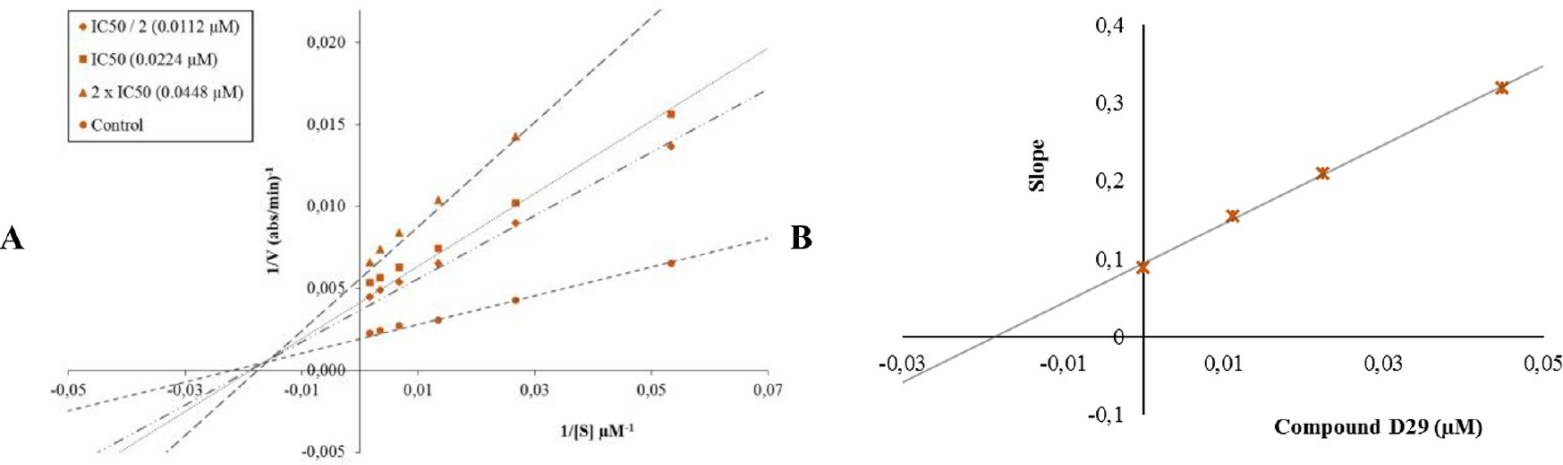
(A) Lineweaver–Burk plots for the inhibition of AChE by compound **D29**. [S], substrate concentration (μM); *V*, reaction velocity (1/*V* (abs/min)^−1^). Inhibitor concentrations are shown at the left. (B) Secondary plot for the calculation of the steady-state inhibition constant (*K*_i_) of compound **D29**. *K*_i_ was calculated as 0.0185 μM.

**Figure 3:**
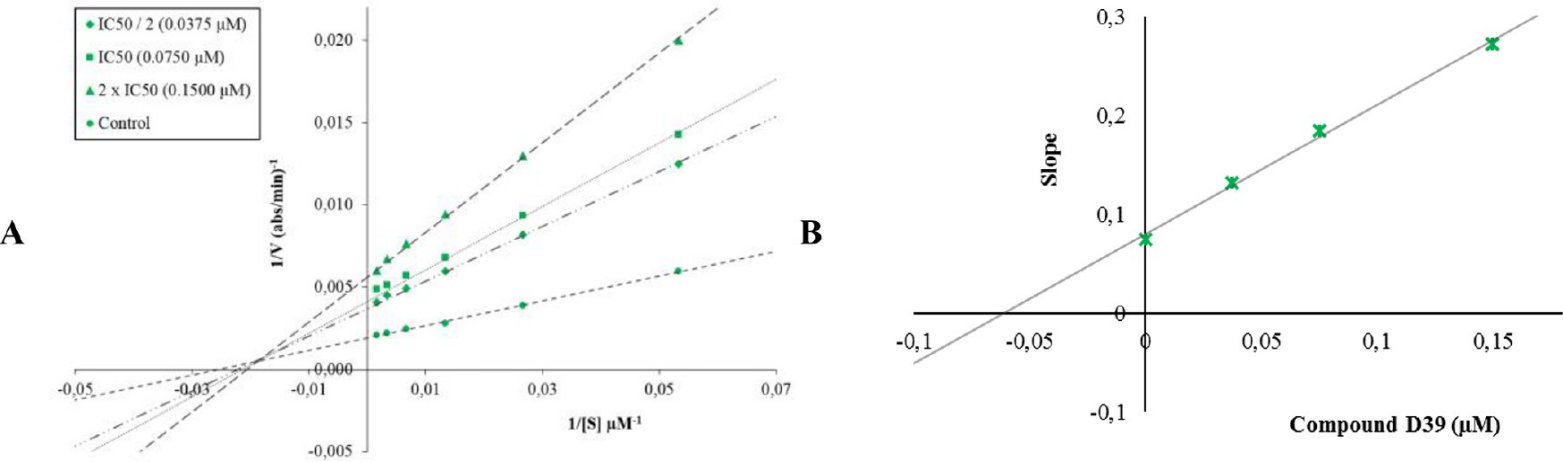
(A) Lineweaver–Burk plots for the inhibition of BChE by compound **D39**. [S], substrate concentration (μM); *V*, reaction velocity (1/*V* (abs/min)^−1^). Inhibitor concentrations are shown at the left. (B) Secondary plot for the calculation of the steady-state inhibition constant (*K*_i_) of compound **D39**. *K*_i_ was calculated as 0.0617 μM.

Compounds **D29**, **D39**, **D28**, and **D37**, which were the most effective derivatives against the AChE, BChE, MAO-A, and MAO-B enzymes, respectively, were selected for enzyme kinetic studies. The Lineweaver–Burk curves obtained as a result of the enzyme kinetic studies of compound **D29** on the AChE enzyme (Figure 2) and those of compound **D39** on the BChE enzyme (Figure 3) showed that the four lines intersected in the area outside the axes. According to the Lineweaver–Burk curves, compound **D29** had mixed inhibition activity on the AChE enzyme and compound **D39** had mixed inhibition activity on the BChE enzyme.

**Figure 4:**
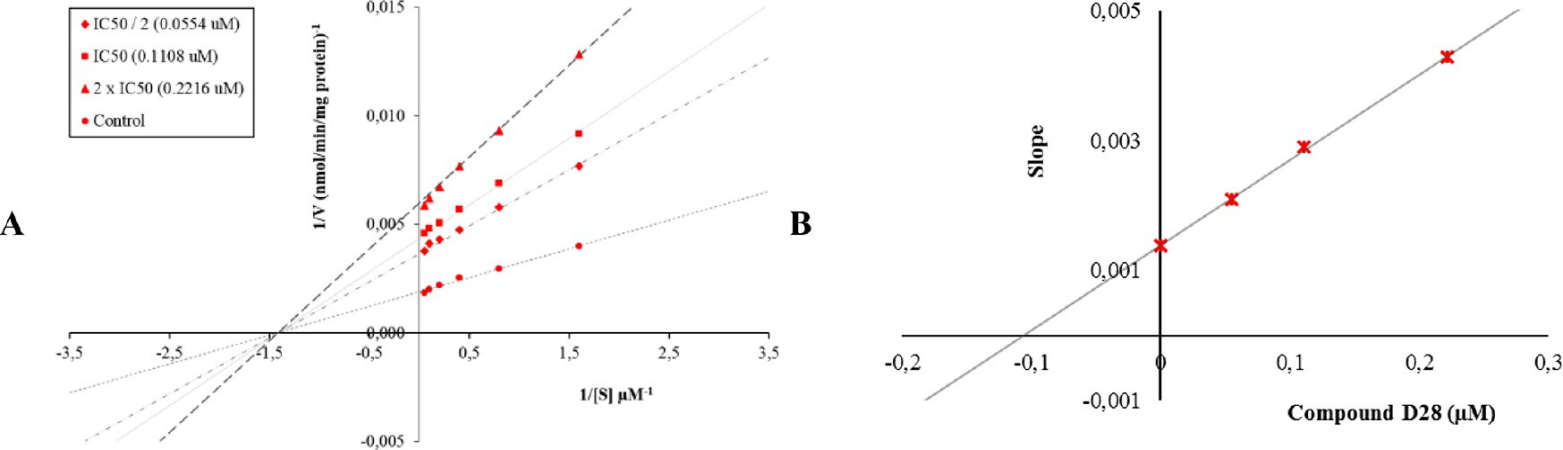
(A) Lineweaver–Burk plots for the inhibition of MAO-A by compound **D28**. [S], substrate concentration (μM); *V*, reaction velocity (1/*V* (abs/min)^−1^). Inhibitor concentrations are shown at the left. (B) Secondary plot for the calculation of the steady-state inhibition constant (*K*_i_) of compound **D28**. *K*_i_ was calculated as 0.1069 μM.

**Figure 5:**
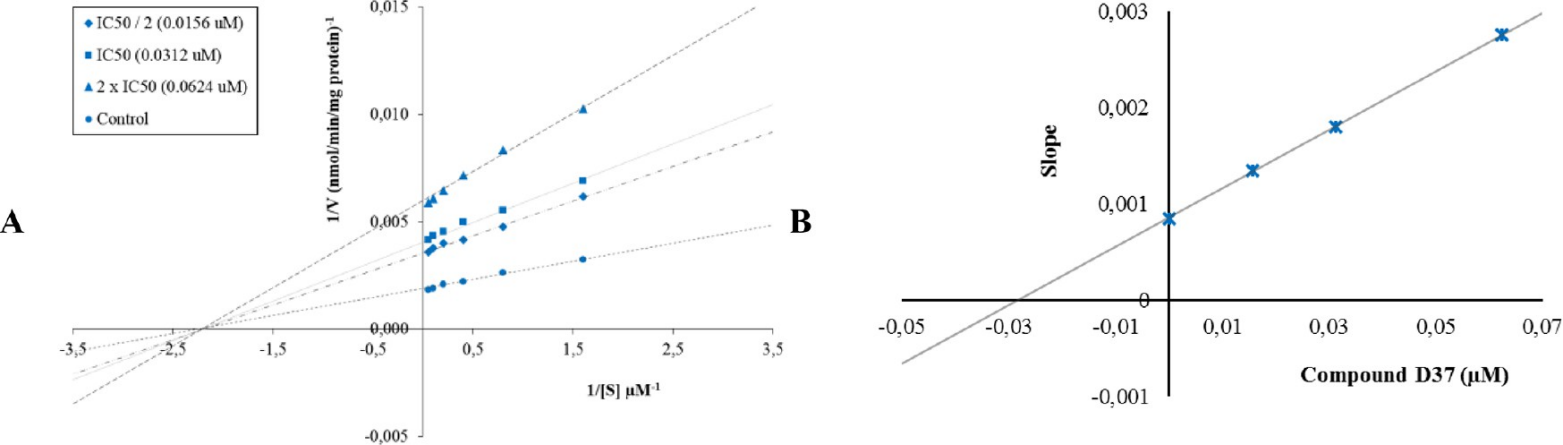
(A) Lineweaver–Burk plots for the inhibition of MAO-B by compound **D37**. [S], substrate concentration (μM); *V*, reaction velocity (1/*V* (abs/min)^−1^). Inhibitor concentrations are shown at the left. (B) Secondary plot for the calculation of the steady-state inhibition constant (*K*_i_) of compound **D37**. *K*_i_ was calculated as 0.0297 μM.

The Lineweaver–Burk curves obtained as a result of the enzyme kinetic studies of compound **D28** on the MAO-A enzyme (Figure 4) and compound **D37** on the MAO-B enzyme (Figure 5) showed that four lines intersected on the *x*-axis. According to the Lineweaver–Burk curves, compound **D28** showed noncompetitive inhibition against the MAO-A enzyme, as did derivative **D37** on the MAO-B enzyme.

#### 3.2.4 DPPH Free-Radical Scavenging Antioxidant Activity

**Figure 6:**
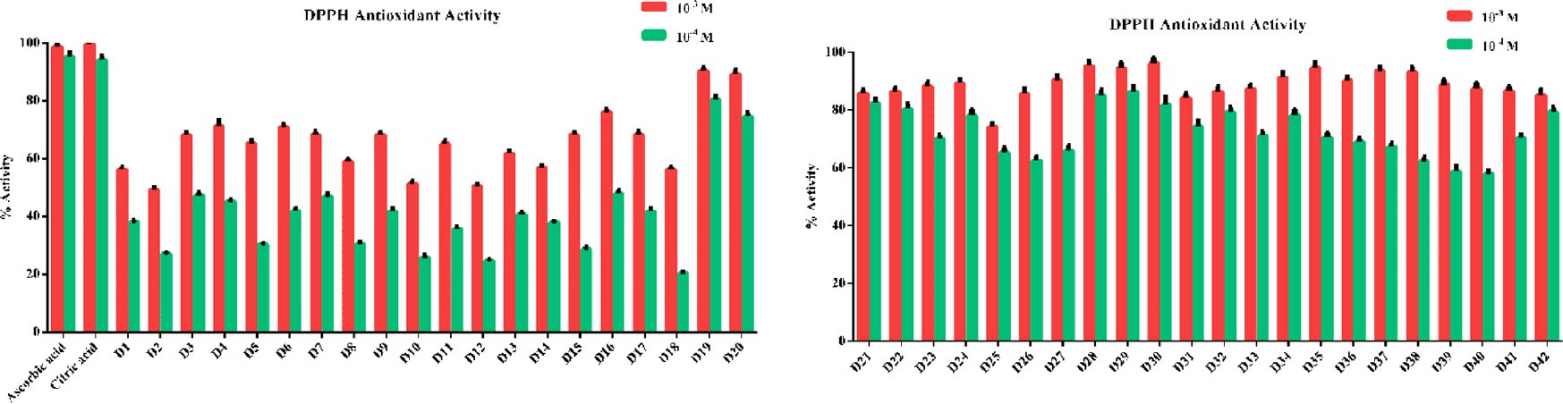
% DPPH antioxidant activity of the synthesized compounds **D1**–**D42** and reference agents.

For the DPPH free-radical scavenging activity test, test compounds were prepared at concentrations of 10^–3^ and 10^–4^ M. Using the absorbance changes determined as a result of the spectroscopic measurement, the % DPPH free-radical scavenging activities of the synthesized compounds and reference materials (ascorbic acid and citric acid) were determined. The % antioxidant activities of all the tested compounds were calculated based on the control. According to the results of the experiment, the reference compounds showed antioxidant activities at ratios from 98.75% ± 1.67 to 95.22% ± 1.47 and from 99.48% ± 1.45 to 94.26% ± 1.37 in ascorbic acid and citric acid at concentrations of 10^–3^ and 10^–4^ M, respectively, proving that the experimental protocol was implemented and worked correctly.^43,44^ Among the test compounds, all the compounds, except compound **D2** at a concentration of 10^–3^ M, showed more than 50% antioxidant activity. Over 50% antioxidant activity was observed in compounds **D19**–**D42** at a concentration of 10^–4^ M. When the results of the DPPH free-radical scavenging activity were evaluated in general, it was determined that the test compounds showed strong antioxidant activity. Among the compounds, **D19**, **D27**–**D30**, and **D34**–**D38** were determined as the compounds with the strongest antioxidant effects, showing 90% or more DPPH free-radical scavenging activity (Figure 6). The compounds **D19**, **D27**–**D30**, and **D34**–**D38** and the standard agents were selected at this step to calculate their IC_50_ values on DPPH antioxidant activity. For this purpose, the related compounds were prepared at their further concentrations by serial dilution (10^–3^–10^–9^ M concentrations). The IC_50_ values of ascorbic acid and citric acid were calculated as 0.165 ± 0.007 μM and 0.194 ± 0.009 μM, respectively. The IC_50_ values of the test derivatives were determined to be 0.362 ± 0.015 μM for **D19**, 0.310 ± 0.013 μM for **D27**, 0.210 ± 0.010 μM for **D28**, 0.188 ± 0.008 μM for **D29**, 0.246 ± 0.011 μM for **D30**, 0.302 ± 0.014 μM for **D34**, 0.238 ± 0.011 μM for **D35**, 0.251 ± 0.010 μM for **D36**, 0.179 ± 0.007 μM for **D37**, and 0.190 ± 0.0078 μM for **D38**.

#### 3.2.5 Beta Amyloid 1–42 (Aβ42) Inhibitor Screening Study

**Figure 7:**
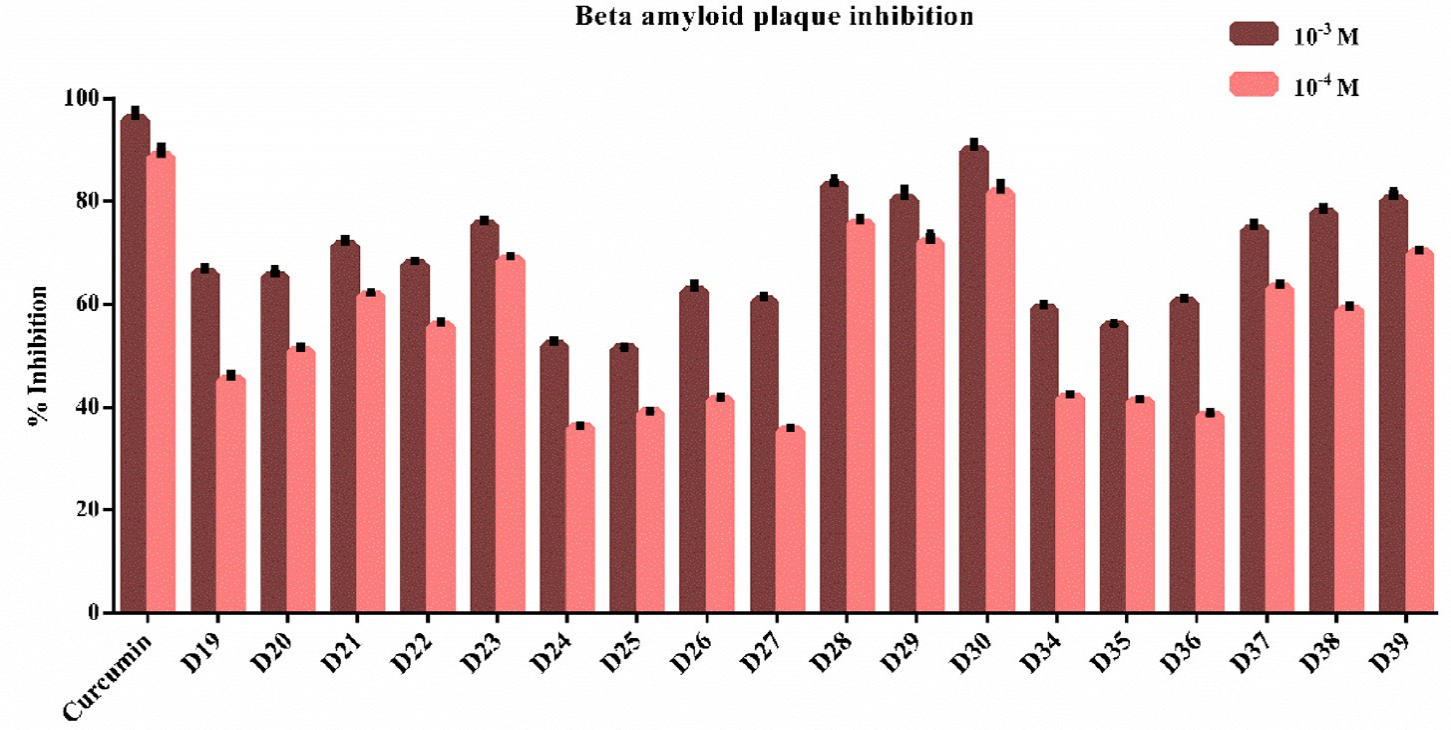
% β-Amyloid plaque inhibition of synthesized compounds **D19**–**D30** and **D34**–**D39** and curcumin.

Using the Beta Amyloid 1–42 (Aβ42) Ligand Screening Assay kit based on the fluorometric approach, the β-amyloid aggregation inhibitory abilities of compounds **D19**–**D30** and **D34**–**D39**, which exhibited strong inhibition effects against AChE and BChE enzymes, were investigated. The test procedure was based on the kit protocol, and curcumin was used as a positive control. β-Amyloid plaque inhibition profiles were examined by preparing compounds **D19**–**D30** and **D34**–**D39** at concentrations of 10^–3^ and 10^–4^ M. When the kit protocol was completed, the % inhibition ratios of curcumin and the test compounds were calculated based on the control group. Curcumin displayed inhibitions of 95.882 ± 1.968% and 88.211 ± 2.167% at concentrations of 10^–3^ and 10^–4^ M, respectively. More than 50% inhibition was seen in all the tested compounds at a concentration of 10^–3^ M. At this concentration, it was determined that compounds **D28**–**D30** and **D39** showed 80% or more inhibition. It was determined that compounds **D20**–**D23**, **D28**–**D30**, and **D37**–**D39** exceeded 50% inhibition at a concentration of 10^–4^ M. Among all of the tested compounds, **D28**–**D30** and **D39** were determined to have the highest inhibition rates. At concentrations of 10^–3^ and 10^–4^ M, compound **D28** showed inhibitions of 82.748 ± 1.682% and 75.627 ± 1.043%, compound **D29** showed inhibitions of 80.265 ± 2.046% and 71.891 ± 1.884%, derivative **D30** showed inhibitions of 89.633 ± 1.856% and 81.484 ± 1.957%, and **D39** showed inhibitions of 80.112 ± 1.879% and 69.760 ± 0.778%, respectively (Figure 7). These compounds (**D28**, **D29**, **D30**, and **D39**) and curcumin were selected at this step to calculate their IC_50_ values on β-amyloid plaque aggregation. For this purpose, the related compounds were prepared at their further concentrations by serial dilution. The IC_50_ value of curcumin was calculated as 0.1055 ± 0.0041 μM; however, those of compounds **D28**, **D29**, **D30**, and **D39** were 0.1467 ± 0.0053, 0.1235 ± 0.0049, 0.1780 ± 0.0074, and 0.1966 ± 0.0079 μM, respectively. These results suggested that, in addition to their capability to inhibit AChE and BChE enzymes, the related compounds had the capacity to prevent β-amyloid plaque aggregation. Enzyme inhibition will increase cholinergic activity and prevent the buildup of β-amyloid plaque, one of the main causes of AD. This may indicate a more effective approach to treating AD. Among the synthesized compounds, **D28**–**D30** and **D39** were found to be effective in the treatment of AD due to their ability to inhibit cholinesterase enzymes and prevent β-amyloid plaque accumulation.

#### 3.2.6 Cytotoxicity Test

The NIH/3T3 mouse fibroblast healthy cell line was used in the cytotoxicity studies of the compounds that were synthesized and found to be effective against the *in vitro* enzyme assays. Nonlinear regression analysis was used to evaluate the test compounds’ IC_50_ values relative to the computed % inhibition values, and the cytotoxic characteristics of associated molecules were examined.

As mentioned above, compounds **D19**–**D30** and **D34**–**D39** were found to be effective on the AChE enzyme, compounds **D34**, **D35**, and **D37**–**D39** were effective on the BChE enzyme, derivatives **D28** and **D29** were effective on the MAO-A enzyme, and compounds **D28**–**D32** and **D37**–**D41** were effective on the MAO-B enzyme. The inhibitory activities of these compounds on the related enzymes (IC_50_ values in the range of 0.0224–0.2347 μM) and the IC_50_ values on the NIH/3T3 fibroblast cell line (in the range of 0.6012–75.4598 μM) are given in Table 3. This finding revealed that the compounds showed inhibition against the AChE, BChE, MAO-A, and MAO-B enzymes at concentrations 25–320× lower than the concentration at which the compounds exerted cytotoxic activity against the NIH/3T3 cells. As a result, it was determined that these compounds are active against the related enzymes and are not toxic at their IC_50_ concentrations.

**Table 3:** IC_50_ Values of the Selected Compounds against the NIH/3T3 Cell Line

	IC_50_ values (μM)				
compounds	AChE enzyme	BChE enzyme	MAO-A enzyme	MAO-B enzyme	NIH/3T3 cell line
**D19**	0.0324 ± 0.0012				10.4692 ± 0.5169
**D20**	0.0311 ± 0.0011				20.0437 ± 1.0018
**D21**	0.0292 ± 0.0008				39.5103 ± 1.8234
**D22**	0.0527 ± 0.0016				32.5436 ± 0.5229
**D23**	0.0510 ± 0.0014				2.1463 ± 0.1270
**D24**	0.0469 ± 0.0019				18.6417 ± 0.1188
**D25**	0.1826 ± 0.0074				13.7831 ± 0.3688
**D26**	0.1763 ± 0.0062				21.7890 ± 0.5558
**D27**	0.1305 ± 0.0042				10.4602 ± 0.4670
**D28**	0.0248 ± 0.0010		0.1108 ± 0.0047	0.0409 ± 0.0019	6.5162 ± 0.1750
**D29**	0.0224 ± 0.0008		0.1116 ± 0.0042	0.0412 ± 0.0018	73.5769 ± 1.7563
**D30**	0.0257 ± 0.0009			0.0456 ± 0.0017	0.6012 ± 0.0145
**D31**				0.0619 ± 0.0028	26.0454 ± 1.2761
**D32**				0.0665 ± 0.0024	281.6974 ± 7.0432
**D34**	0.1911 ± 0.0087	0.1323 ± 0.0051			75.4598 ± 2.1756
**D35**	0.2150 ± 0.0088	0.1505 ± 0.0048			53.9423 ± 1.2166
**D36**	0.2347 ± 0.0113				3.6181 ± 0.1289
**D37**	0.0482 ± 0.0017	0.0839 ± 0.0034		0.0312 ± 0.0008	2.2865 ± 0.1365
**D38**	0.0473 ± 0.0016	0.0782 ± 0.0029		0.0359 ± 0.0013	6.8118 ± 0.1183
**D39**	0.0458 ± 0.0009	0.0750 ± 0.0032		0.0393 ± 0.0011	12.3498 ± 0.4158
**D40**				0.2030 ± 0.0098	6.8741 ± 0.2974
**D41**				0.2089 ± 0.0095	5.8898 ± 0.1187

### 3.3 Prediction of ADME Parameters

Using QikProp 4.8 software, the ADME properties of the prepared compounds **D1**–**D42** were assessed.^31^ Drug-likeness qualities were assessed using QikProp in addition to the ADME features. Lipinski’s rule of five and Jorgensen’s rule of three were used to evaluate the compounds’ drug-likeness.^45−48^ The calculated ADME parameters are presented in Supporting Information Table S3. All the parameters remain within the reference ranges, as can be observed. Because they did not result in more than one violation of the rules of three and five, the derived compounds **D1**–-**D42** were in conformity with the established parameters. The synthesized compounds were shown to exhibit pharmacokinetic characteristics that may be acceptable for clinical usage after taking the findings of the ADME parameter tests into account.

### 3.4 Evaluation of Molecular Docking Studies

#### 3.4.1 Molecular Docking Studies of the AChE Enzyme

Docking experiments were performed on the AChE enzyme’s crystal structure to ascertain how the molecules **D19**–**D30** and **D34**–**D39**, which demonstrated strong AChE enzyme inhibitory effects, would interact with the active site (PDB code 4EY7)^32^ (Supporting Information Figure S1). Presented below are the molecular docking results of compounds **D28**–**D30**, which were the most potent inhibitor agents on AChE.

It was remarkable to see that the catalytic site (CAS) and the peripheral anionic site (PAS) were two distinct binding sites on the AChE enzyme. The CAS area was shown to connect to the amino acids Ser203, Glu334, His447, Trp86, Tyr130, Tyr133, Tyr337, and Phe338, whereas the PAS region was found to bind to the amino acids Tyr72, Asp74, Tyr124, Tyr341, and Trp286.^49−52^ The active compounds settle in the gorge formed by both regions, bind to the enzyme, and exert their effects. Numerous modeling studies have shown that donepezil binds with both AChE enzyme regions and settles extremely effectively in the gorge thanks to its dual binding side or DBS property.^49−52^

When the docking poses of compounds **D19**–**D30** and **D34**–**D39** (Supporting Information Figure S1) were examined, it was observed that they bound to the AChE enzyme in a position similar to that of donepezil. When the chemical structures of the related compounds were examined, it was seen that an aromatic structure formed by indanone and benzene rings and a polar structure formed by piperazine and aliphatic amino groups came together. According to the docking poses of the compounds, it was observed that the aromatic structure was placed in the PAS region of the enzyme active region and the polar structure interacted with the CAS region. The related molecules behaved similarly to donepezil in that they interacted with the DBS and accumulated on the enzyme’s active site.

The molecular modeling studies on donepezil (Supporting Information Figure S1) showed that the π–π interaction between the indanone ring and the indole ring of the Trp286 amino acid in the PAS region of the enzyme active site is important for binding.^53−55^ The fact that this interaction was observed with Trp286 in the experiments with both donepezil and the related compounds during the modeling studies indicated that the chosen method and the path followed were correct. The π–π interaction between the indanone ring and the Trp286 indole was clearly seen in the docking poses of compounds **D28**–**D30**. The carbonyl group in the indanone ring is a very active structure in terms of interaction. When the docking poses were examined, it was seen that a hydrogen bond was formed between the carbonyl oxygen and the amino group of the Phe295 amino acid in the related compounds. The π–π interaction between the benzene ring in the chemical structure and the phenyl ring of the Tyr341 amino acid is an important factor in binding to the enzyme active site. While in compound **D29** this interaction was seen with the phenyl ring of the Phe338 amino acid, for compounds **D28** and **D30** the benzene ring in the structure formed a π–π interaction with both the phenyl rings of the Tyr124 and Phe338 amino acids in the enzyme active site.

The aromatic structure formed by the indanone and benzene rings in the compounds and the polar structure formed by the substituted piperazine and aliphatic secondary amine groups are linked by the amide group. Since the amide structure contains both amino and carbonyl groups, it very conveniently forms hydrogen bonds by acting as a hydrogen acceptor and donor. The hydrogen bond formed with the carbonyl of the amide group was observed between the compounds **D28**–**D30** and the amino group of the Ser203 amino acid. In addition to the mentioned hydrogen bonds, the amide group in compound **D29** formed two more hydrogen bonds with the carbonyl and the amino group in amino acids Gly121 and Gly122. In terms of the design of the compounds, the presence of the amide group in the structure is very important in order for it to resemble the structure of the acetylcholine molecule. In the enzymatic mechanism of acetylcholine, the ester group in the structure undergoes nucleophilic attack by the amino acid Ser203 and is broken down into the choline group and acetic acid.^56,57^ The hydrogen bonds determined by the carbonyl of the amide group in the structures of the related compounds with other amino acids, especially the Ser203 amino acid, were compatible with the acetylcholine mechanism. These findings indicated that the related compounds can bind to the AChE enzyme more strongly and selectively than acetylcholine.

In compounds **D28**–**D30**, the *N*-methyl-*N*-propylamine alkyl group was substituted by the amide moiety. The methyl and propyl chains in the alkyl group both affected the conformation of the related compounds and contributed to the van der Waals interaction with the amino acids in the enzyme active site. The N atom in the alkyl group left and formed a hydrogen bond with the hydroxyl of the amino acid Glu202. In addition, it was seen that the N atom in compounds **D28** and **D29** established a cation−π interaction with the indole ring of the amino acid Trp86.

**Figure 8:**
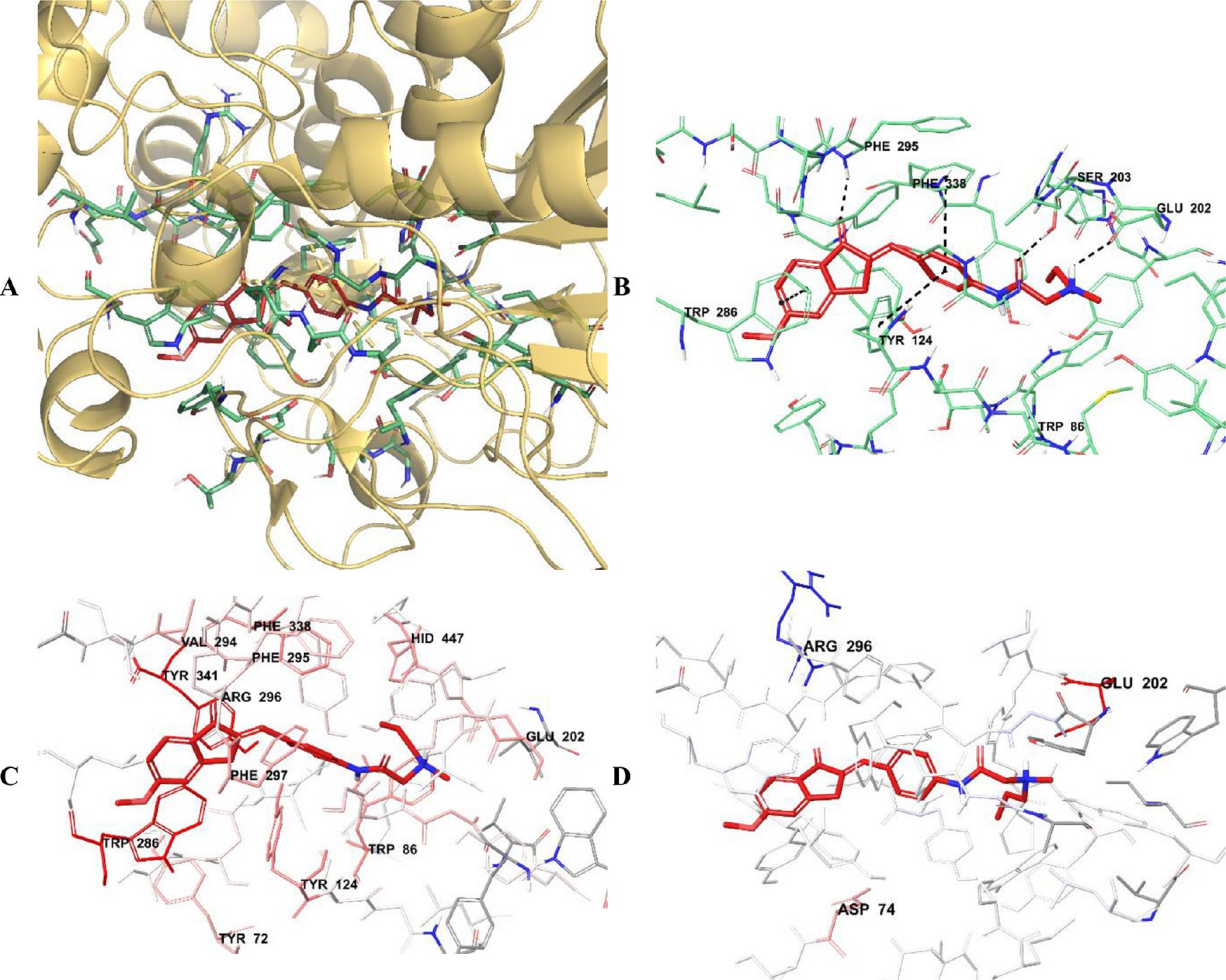
(A) Three-dimensional placement pose and (B) three-dimensional interaction mode of compound **D28** in the active site of AChE. The inhibitor and important residues in the active site of the enzyme are represented by a tube model and colored with red and aquamarine, respectively. (C) Van der Waals and (D) electrostatic interactions of this compound with the active region of AChE. The active ligand has a lot of favorable van der Waals (red and pink) and electrostatic (blue, red, and pink) interactions (AChE PDB code 4EY7).

**Figure 9:**
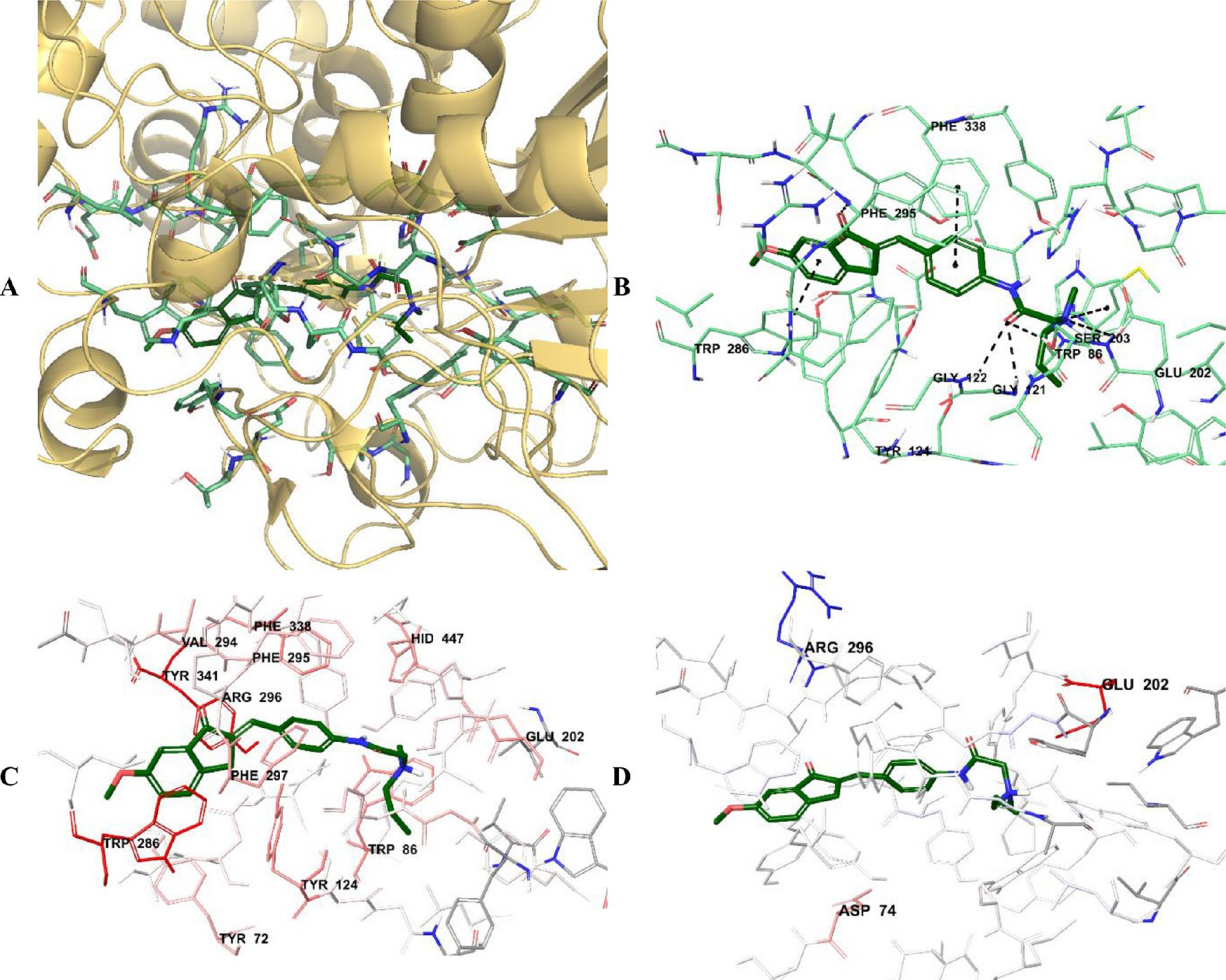
(A) Three-dimensional placement pose and (B) three-dimensional interaction mode of compound **D29** in the active site of AChE. The inhibitor and important residues in the active site of the enzyme are represented by a tube model and colored with dark green and aquamarine, respectively. (C) Van der Waals and (D) electrostatic interactions of this compound with the active region of AChE. The active ligand has a lot of favorable van der Waals (red and pink) and electrostatic (blue, red, and pink) interactions (AChE PDB code 4EY7).

**Figure 10:**
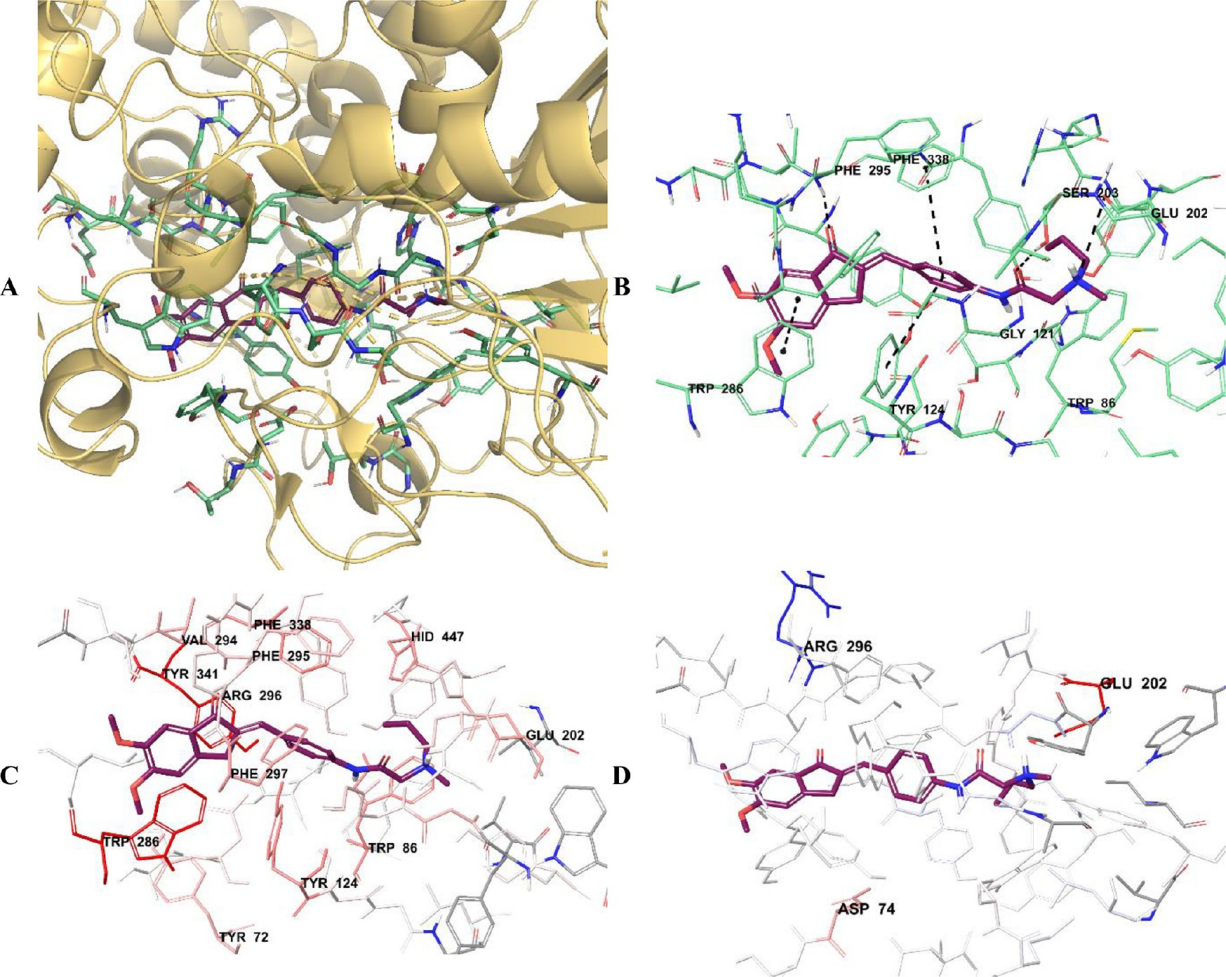
(A) Three-dimensional placement pose and (B) three-dimensional interaction mode of compound **D30** in the active site of AChE. The inhibitor and important residues in the active site of the enzyme are presented by a tube model and colored with maroon and aquamarine, respectively. (C) Van der Waals and (D) electrostatic interactions of this compound with the active region of AChE. The active ligand has a lot of favorable van der Waals (red and pink) and electrostatic (blue, red, and pink) interactions (AChE PDB code 4EY7).

According to the results of the modeling studies, compounds **D28**, **D29**, and **D30** (Figures 8–10) exhibited the most similar interactions with donepezil and stronger binding with both the PAS and CAS regions when compared to the other compounds in the series; additionally, they were the most active compounds in the series. Compounds **D28**, **D29**, and **D30** were determined as the most effective AChE enzyme inhibitor candidates among the synthesized compounds, with IC_50_ values of 0.0248 ± 0.0010, 0.0224 ± 0.0008, and 0.0257 ± 0.0009 μM, respectively.

The van der Waals and electrostatic interactions of compounds **D28**–**D30** with amino acids in the enzyme active site were visualized with the Per-Residue Interaction panel, where red and pink indicate strong van der Waals interactions while blue, red, and pink represent strong electrostatic interactions. When analyzing the van der Waals interactions for compounds **D28**–**D30**, it was seen that there were strong van der Waals interactions with amino acids Tyr72, Trp86, Tyr124, Glu202, Trp286, Val294, Phe295, Arg296, Phe297, Phe338, Tyr341, and His447 at the enzyme active site. When the electrostatic interactions of the same compounds were examined, it was determined that there were strong interactions with amino acids Asp74, Glu202, and Arg296.

#### 3.4.2 Molecular Docking Studies of the BChE Enzyme

**Figure 11:**
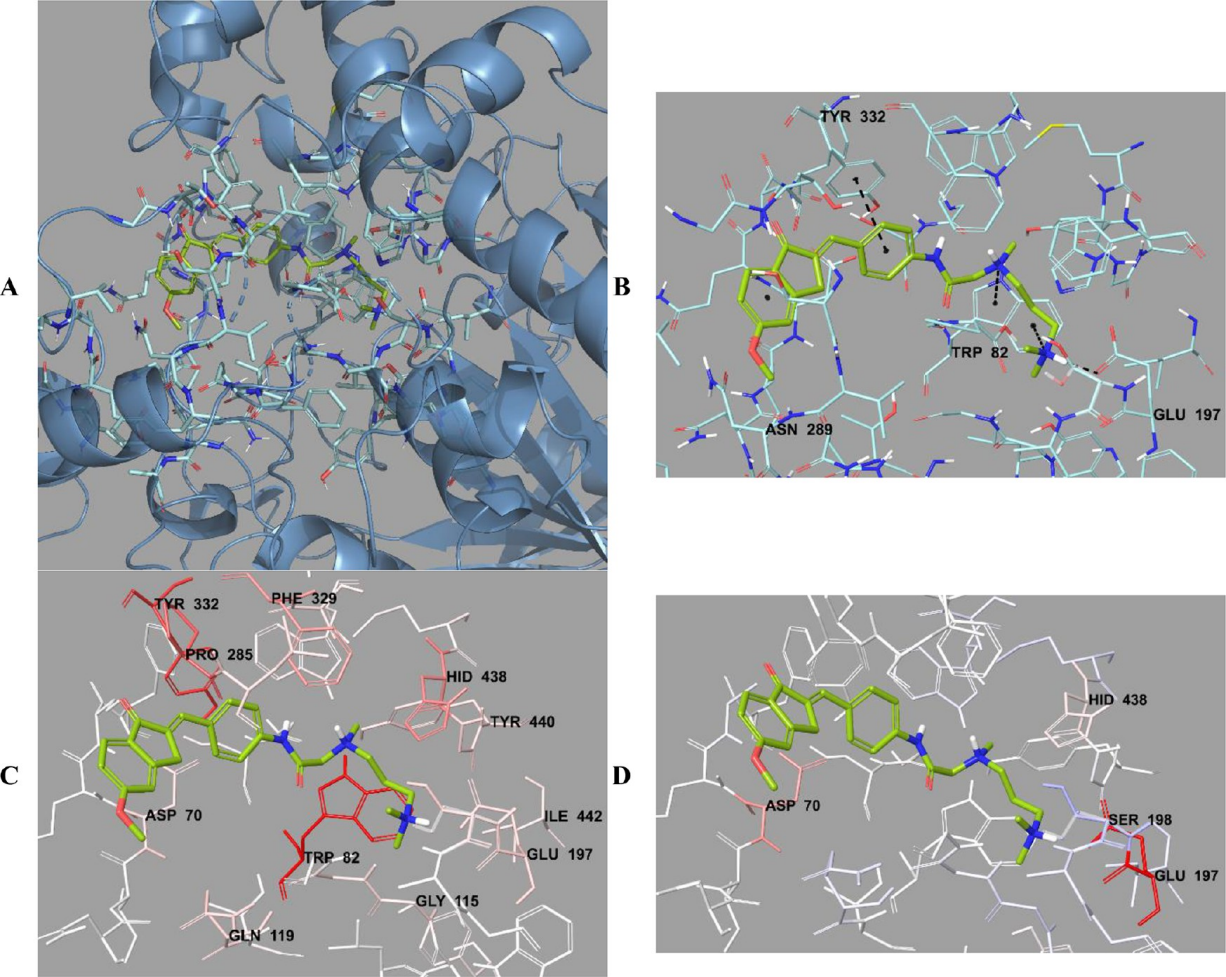
(A) Three-dimensional placement pose and (B) three-dimensional interaction mode of compound **D37** in the active site of BChE. The inhibitor and important residues in the active site of the enzyme are represented by a tube model and colored with lime green and turquoise, respectively. (C) Van der Waals and (D) electrostatic interactions of this compound with the active region of BChE. The active ligand has a lot of favorable van der Waals (red and pink) and electrostatic (blue, red, and pink) interactions (BChE PDB code 4BDS).

**Figure 12:**
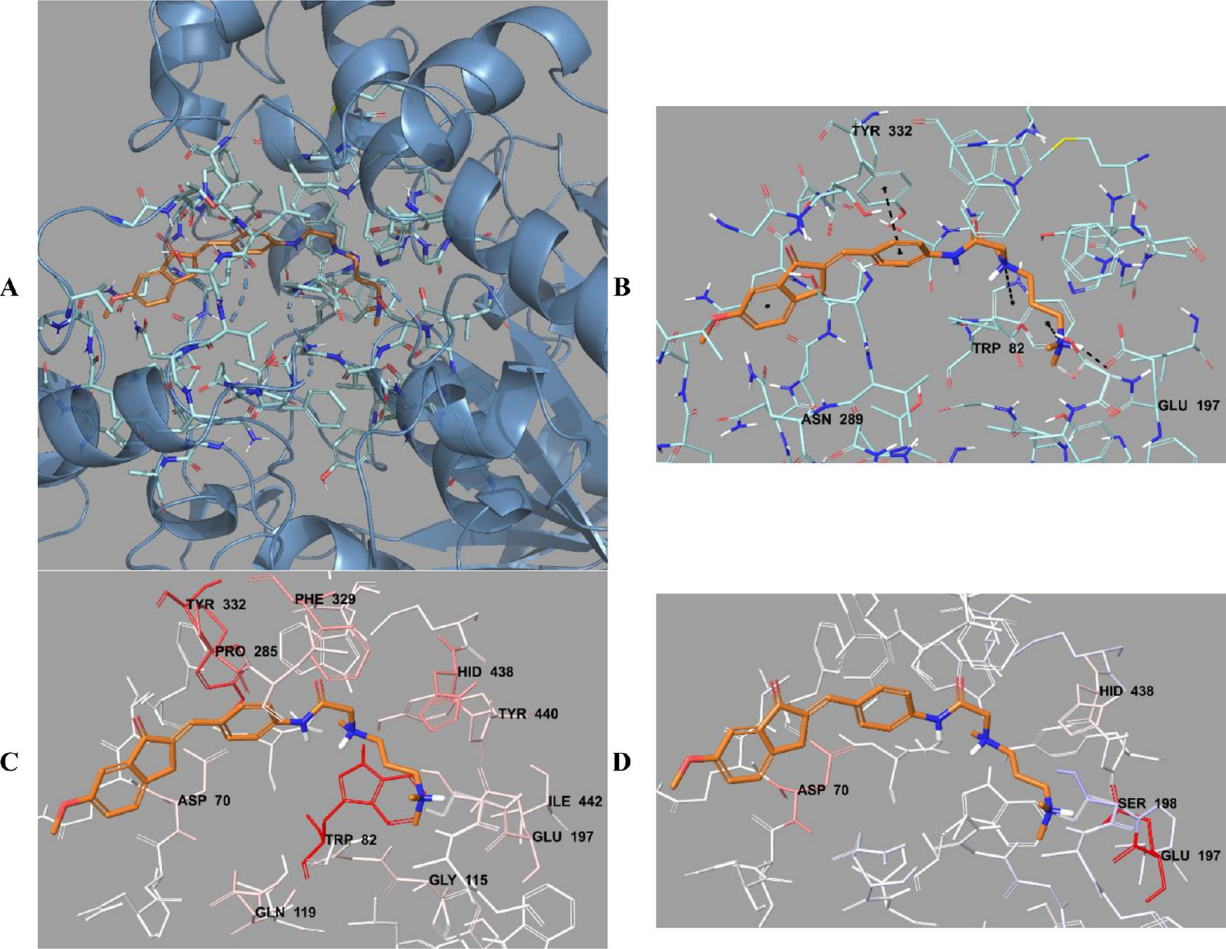
(A) Three-dimensional placement pose and (B) three-dimensional interacting mode of compound **D38** in the active site of BChE. The inhibitor and important residues in the active site of the enzyme are represented by a tube model and colored with orange and turquoise, respectively. (C) Van der Waals and (D) electrostatic interactions of this compound with the active region of BChE. The active ligand has a lot of favorable van der Waals (red and pink) and electrostatic (blue, red, and pink) interactions (BChE PDB code 4BDS).

**Figure 13:**
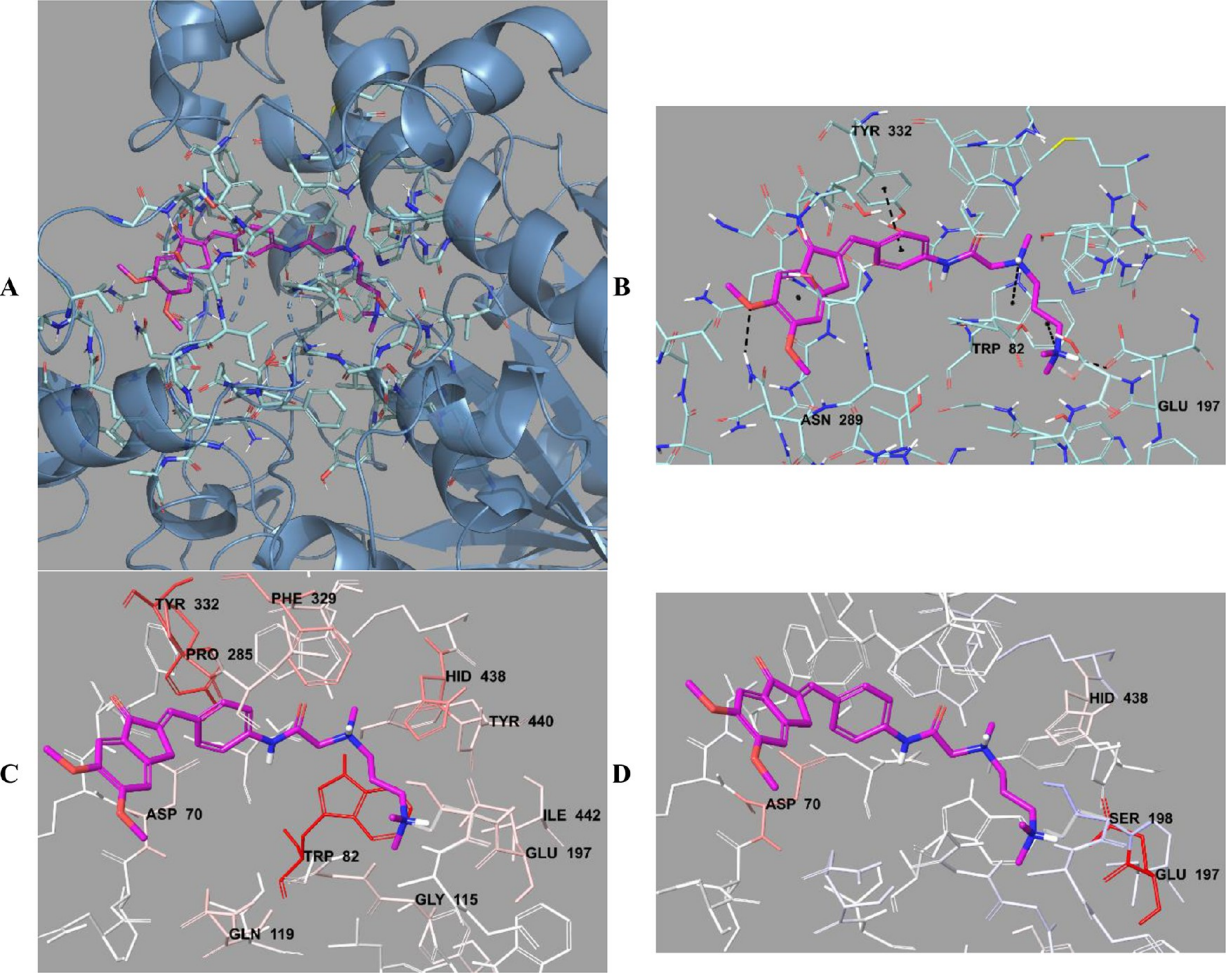
(A) Three-dimensional placement pose and (B) three-dimensional interaction mode of compound **D39** in the active site of BChE. The inhibitor and important residues in the active site of the enzyme are represented by a tube model and colored with pink and turquoise, respectively. (C) Van der Waals and (D) electrostatic interactions of this compound with the active region of BChE. The active ligand has a lot of favorable van der Waals (red and pink) and electrostatic (blue, red, and pink) interactions (BChE PDB code 4BDS).

The possible interactions with the enzyme active sites of compounds **D34**, **D35**, and **D37**–**D39** were also determined and found to be effective on the BChE enzyme evaluated in this study using the crystal structure of the BChE enzyme (PDB code 4BDS)^33^ (Supporting Information Figure S2). As shown below, the molecular docking results of compounds **D37**–**D39**, which were the most potent inhibitor agents on BChE, are presented in Figures 11–13.

When the docking images of compounds **D37**–**D39** were examined, it was determined that three compounds showed four common interactions. The phenyl ring in the middle of the chemical structure created a π–π interaction with the phenyl ring of the amino acid Tyr322. The terminal N atom of the dimethylamino group formed a hydrogen bond with the amino group of the amino acid Glu197. In the structure, two N atoms in the amide-linked *N*-methyl-*N*-(3-(dimethylamino)propyl)amino group were quaternized and formed two cation−π interactions with the Trp82 amino acid. In addition to all of these interactions, compound **D39** showed another interaction through the methoxy group located at the sixth position of the indanone ring. It was observed that hydrogen bonding occurred between the methoxy oxygen and the amino group of the amino acid Asn289. As a result, it was seen that especially the terminal N atoms made strong polar interactions with the active site. This finding can explain why compounds **D37**–**D39** (with IC_50_ values of 0.0839 ± 0.0034, 0.0782 ± 0.0029, and 0.0750 ± 0.0032 μM, respectively) were the most potent BChE enzyme inhibitors in the series.

The van der Waals and electrostatic interactions of compounds **D37**–**D39** with the amino acids in the enzyme active site were visualized with the Per-Residue Interaction panel. Accordingly, strong van der Waals interactions with amino acids Asp70, Trp82, Gly115, Gln119, Glu197, Pro285, Phe329, Tyr332, His438, Tyr440, and Ile442 (in pink and red) appeared for compounds **D37**–**D39**. In terms of the electrostatic interactions, it was determined that these compounds interacted strongly with amino acids Asp70, Glu197, Ser198, and His438 (pink, blue, and red).

#### 3.4.3 Molecular Docking Studies of the MAO-A Enzyme

Docking studies were carried out on the crystal structure of the MAO-A enzyme (PDB code 2Z5X)^34^ in order to determine the possible interactions of compounds **D28** and **D29**, which were effective on the MAO-A enzyme. In the studies, the docking technique performed with Glide 7.1^38^ software was applied, and the grid was formed by centering the N5 atom of the FAD residue, which was located in the enzyme catalytic region.^58−60^

**Figure 14:**
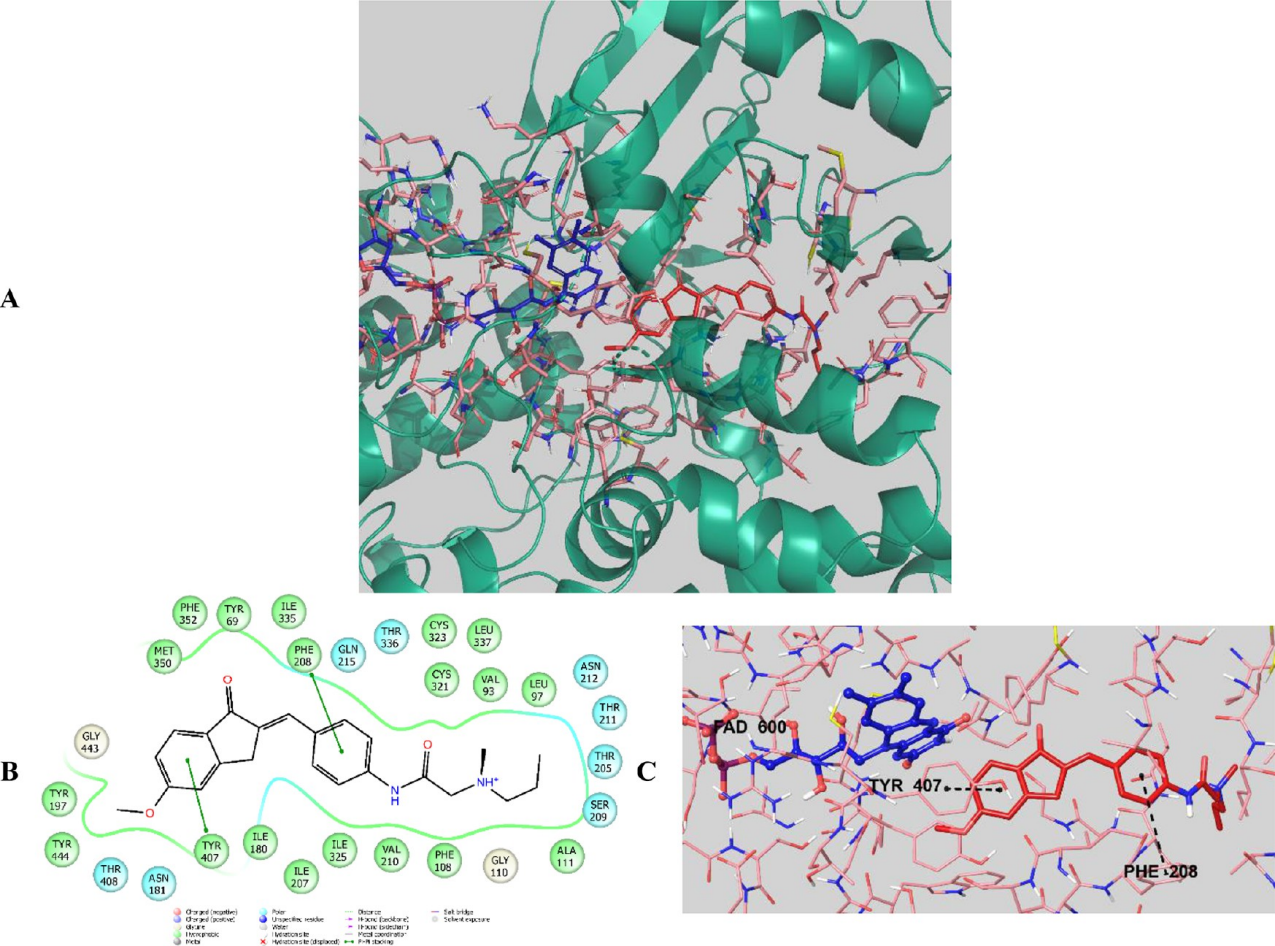
(A) Three-dimensional placement pose and (B) two-dimensional and (C) three-dimensional interaction modes of compound **D28** in the active site of MAO-A. The inhibitor and important residues in the active site of the enzyme are represented by a tube model and colored with red and plum, respectively (MAO-A PDB code 2Z5X).

**Figure 15:**
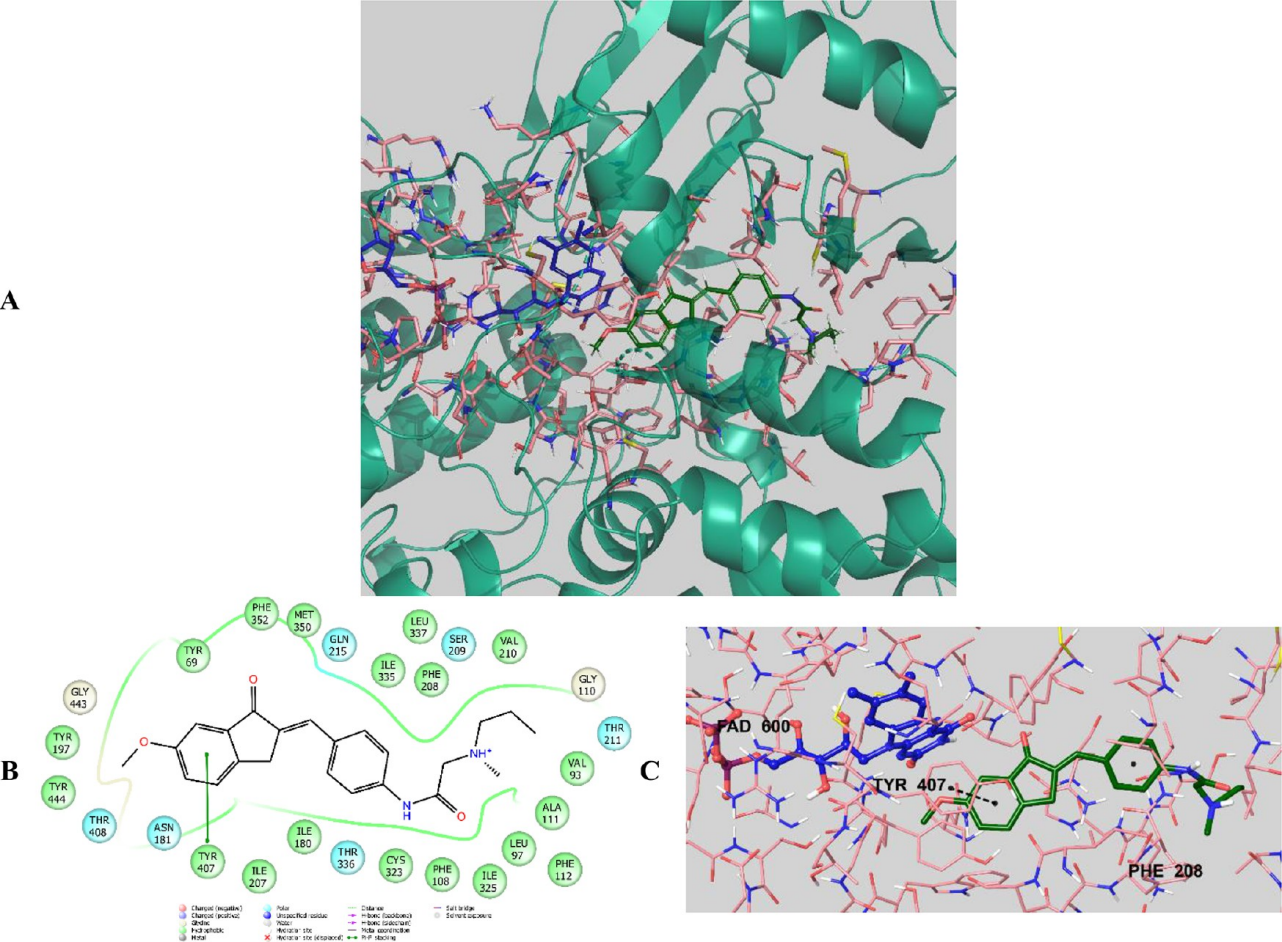
(A) Three-dimensional placement pose and (B) two-dimensional and (C) three-dimensional interaction modes of compound **D29** in the active site of MAO-A. The inhibitor and important residues in the active site of the enzyme are represented by a tube model and colored with dark green and plum, respectively (MAO-A PDB code 2Z5X).

Looking at the docking poses of compounds **D28** and **D29** (Figures 14 and 15), it was seen that there was a π–π interaction between the phenyl of the indanone ring and the phenyl of Tyr407 in both compounds. In compound **D28**, unlike compound **D29**, it was determined that the phenyl ring in the middle of the structure established a π–π interaction with the phenyl of Phe208. These compounds were the most effective derivatives, with IC_50_ values of 0.1108 ± 0.0047 and 0.1116 ± 0.0042 μM, respectively.

#### 3.4.4 Molecular Docking Studies of the MAO-B Enzyme

**Figure 16:**
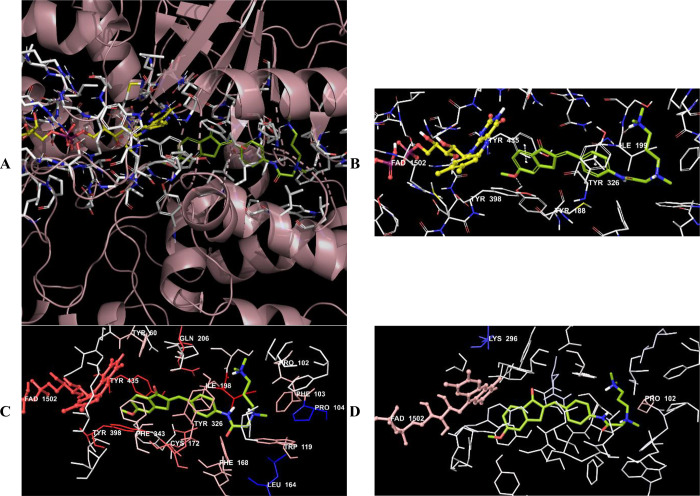
(A) Three-dimensional placement pose and (B) three-dimensional interaction mode of compound **D37** in the active site of MAO-B. The inhibitor and important residues in the active site of the enzyme are represented by a tube model and colored with lime green and white, respectively. (C) Van der Waals and (D) electrostatic interactions of this compound with the active region of MAO-B. The active ligand has a lot of favorable van der Waals (red and pink) and electrostatic (blue, red, and pink) interactions (MAO-B PDB code 2V5Z).

**Figure 17:**
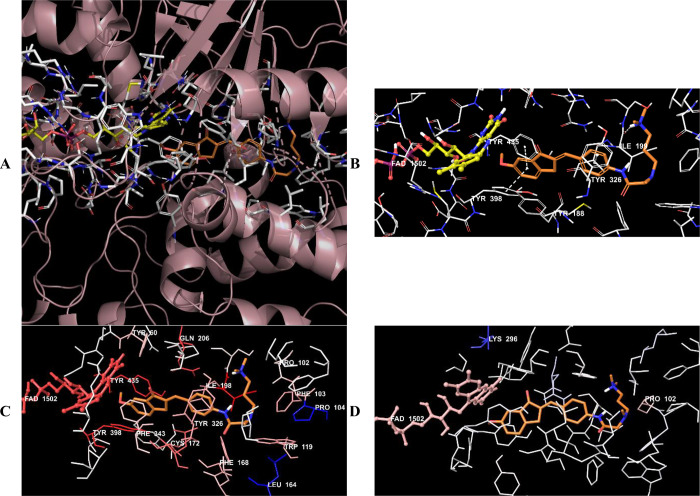
(A) Three-dimensional placement pose and (B) three-dimensional interaction mode of compound **D38** in the active site of MAO-B. The inhibitor and important residues in the active site of the enzyme are represented by a tube model and colored with orange and white, respectively. (C) Van der Waals and (D) electrostatic interactions of this compound with the active region of MAO-B. The active ligand has a lot of favorable van der Waals (red and pink) and electrostatic (blue, red, and pink) interactions (MAO-B PDB code 2V5Z).

**Figure 18:**
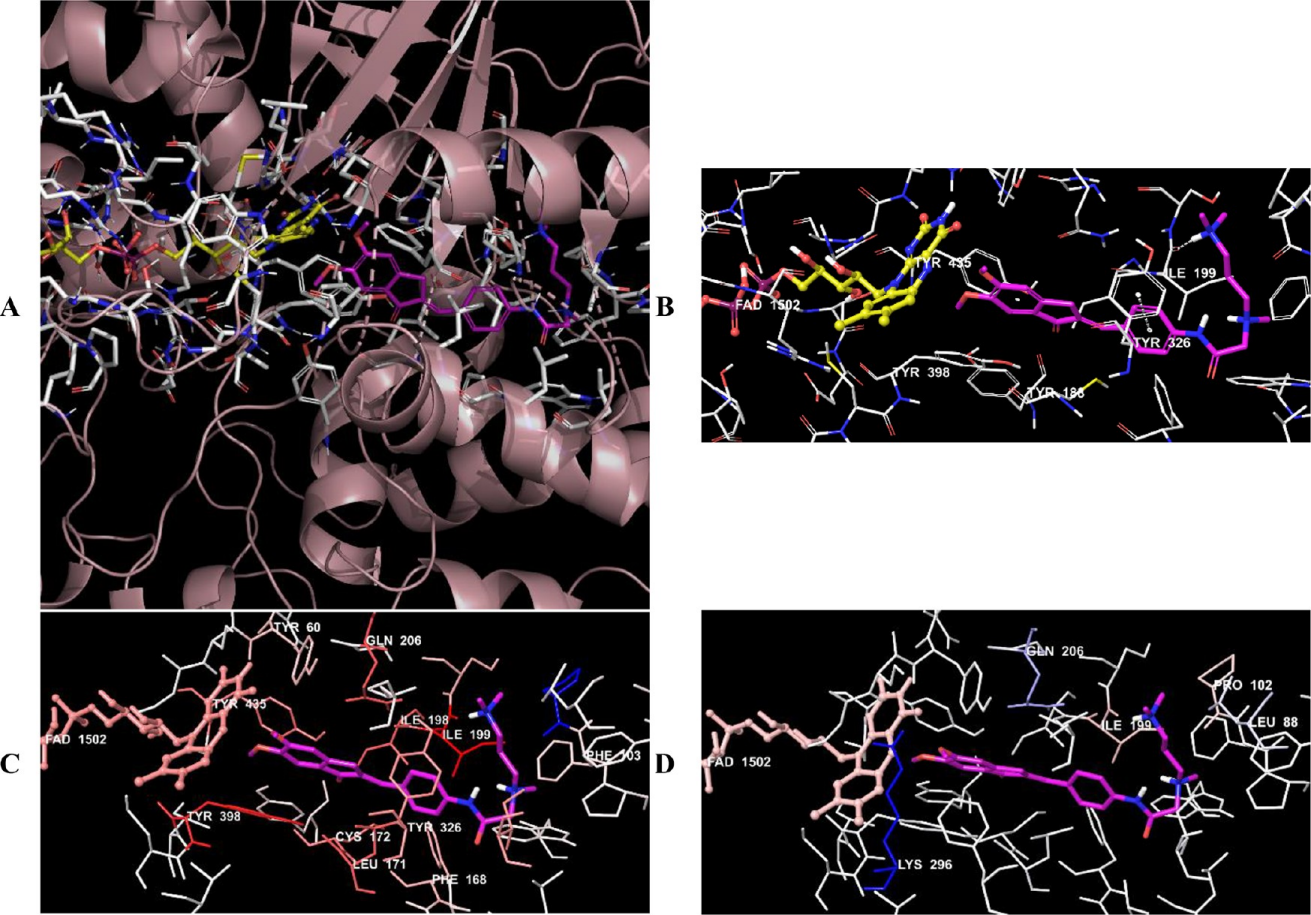
(A) Three-dimensional placement pose and (B) three-dimensional interaction mode of compound **D39** in the active site of MAO-B. The inhibitor and important residues in the active site of the enzyme are presented as a tube model and colored with pink and white, respectively. (C) Van der Waals and (D) electrostatic interactions of this compound with active region of MAO-B. The active ligand has a lot of favorable van der Waals (red and pink) and electrostatic (blue, red, and pink) interactions (MAO-B PDB code 2V5Z).

Docking studies were carried out on the crystal structure of the MAO-B enzyme (PDB code 2V5Z)^35^ in order to determine the possible interactions of compounds **D28**–**D32** and **D37**–**D41**, which were effective on the MAO-B enzyme, with the enzyme active site (Supporting Information Figure S3). In the studies, the docking technique performed with Glide 7.1^38^ software was applied and the grid was formed by centering the N5 atom of the FAD residue, which was located in the enzyme catalytic region.^58−60^ As shown below, the molecular docking results of compounds **D37**–**D39**, which were the most potent inhibitor agents on MAO-B, are presented (Figures 16–18).

Compounds **D37**–**D39** were analyzed according to the results of docking studies on the MAO-B enzyme. Except for derivative **D39**, the phenyl of the indanone ring established a π–π interaction with the amino acids in the enzyme active site, thereby providing an important binding point in the substrate binding site. In compound **D37**, a π–π interaction appeared to occur between the phenyl of the indanone ring and the phenyl of the Tyr435 amino acid. In compound **D38**, this interaction was detected as two π–π interactions with the phenyl rings of amino acids Tyr398 and Tyr435.

The methoxy groups located at the fifth, sixth, and 5,6-positions on the indanone ring in these compounds are very important in terms of polar interactions. The methoxy oxygen shows the ability to form hydrogen bonds with amino acids in the active site. However, when the two-dimensional interaction poses of compounds **D37**–**D39** were examined, it was observed that this could only be achieved with the methoxy group in the fifth position. The oxygen of the 5-methoxy group in the structure of compound **D37** formed a hydrogen bond with the hydroxyl of Tyr198. It can be argued that this interaction was not observed conformationally in compounds containing methoxy groups in the sixth position and the 5,6-dipositions.

The phenyl ring, which was located in the middle of the structures of compounds **D37**–**D39**, strengthened the binding by nonpolar interactions in the substrate binding region. The phenyl ring established a π–π interaction with the phenyl of Tyr326 in the active site of the enzyme. Moreover, amide-substituted secondary amine groups were attached in these compounds. In particular, the N atoms in the structures had the potential to form important interactions with the amino acid Ile199, which was located in the enzyme active site, connected the substrate binding and entrance cavities, and also acted as a gate. The terminal N atoms in the structure formed a hydrogen bond with the carbonyl of Ile199 in the active site, thereby providing the key interaction in the substrate or inhibitor orientation. In addition, the N atoms in question can perform charge transfer interactions with amino acids in the enzyme active site by quaternization. In these compounds, the N atoms at the end of the structure formed a salt bridge with amino acid Glu84.

The van der Waals and electrostatic interactions of compounds **D37**–**D39** with amino acids in the enzyme active site were visualized with the Per-Residue Interaction panel. According to this panel, the van der Waals interactions (red and pink amino acids) were with amino acids Tyr60, Pro102, Phe103, Trp119, Phe168, Leu171, Cys172, Ile198, Ile199, Gln206, Tyr326, Phe343, Tyr398, and Tyr435. Electrostatic interactions were also determined with amino acids Leu88, Pro102, Ile199, Gln206, and Lbs296 (blue, red, and pink amino acids).

It was seen that compounds **D37**–**D39** structurally interacted through indanone and phenyl rings and substituted amine groups, completely binding to the active site. These compounds showed strong binding modes to substrate binding and access cavities by forming key interactions with amino acids important in enzyme active sites and were determined as compounds with effective enzyme inhibition profiles in the series. Compounds **D37**–**D39** had the most effective MAO-B enzyme activities among the series, with IC_50_ values of 0.0312 ± 0.0008, 0.0359 ± 0.0013, and 0.0393 ± 0.0011 μM, respectively.

### 3.5 Evaluation of Molecular Dynamic Simulations Studies

#### 3.5.1 Molecular Investigation of the Binding Modes on the AChE Enzyme

**Figure 19:**
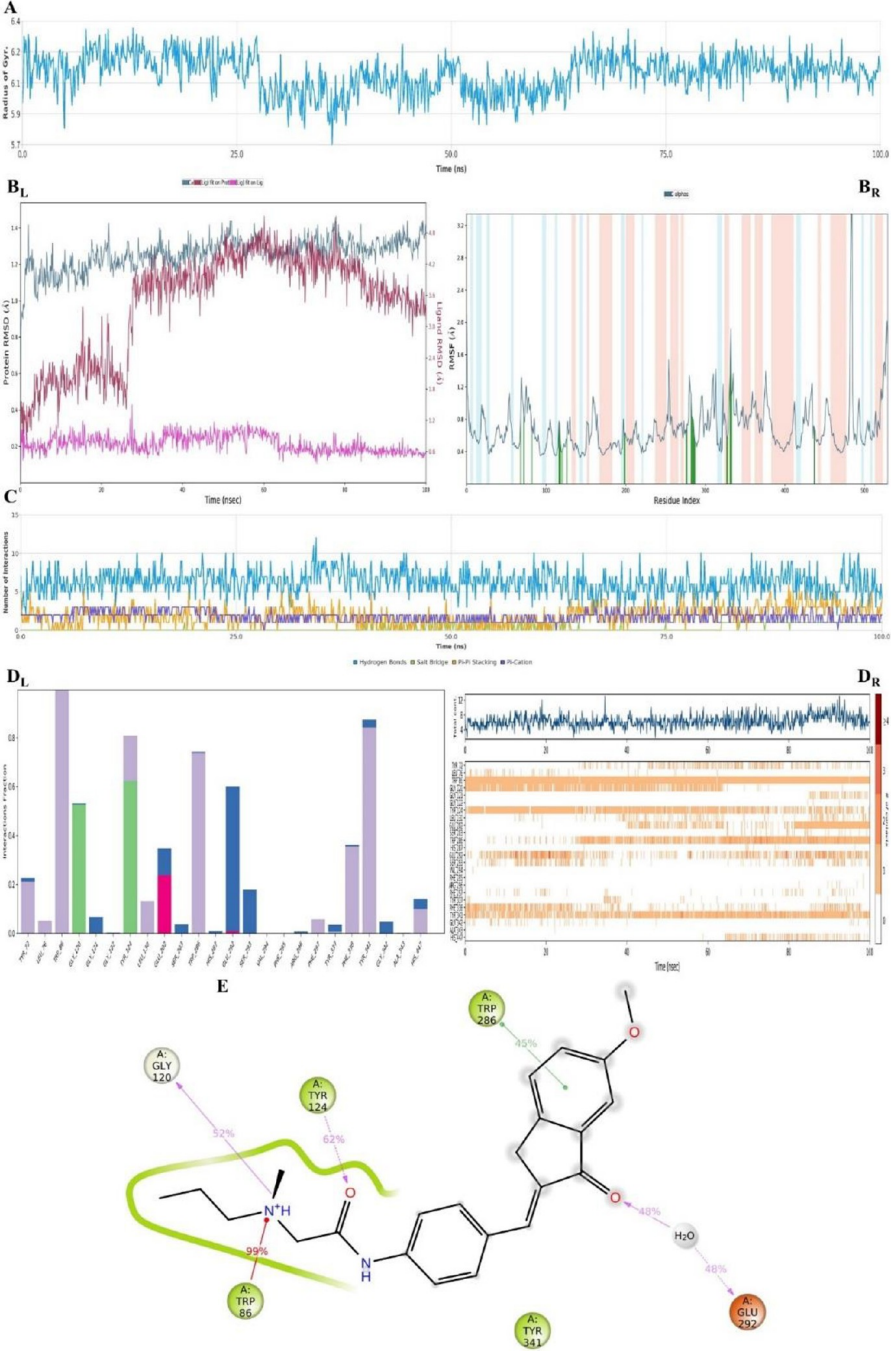
Plots of the MDS results for the compound **D29**–AChE enzyme complex. Stability properties: (A) Rg, (B_L_) RMSD, and (B_R_) RMSF plots. Interaction properties: (C) number of interactions–interaction types–time plot, (D_L_) interaction fraction–residue diagram, (D_R_) total connections–residues–time plot, and (E) 2D interaction pose with the connection strength (cut off of 0.2) at the active region.

**Figure 20:**
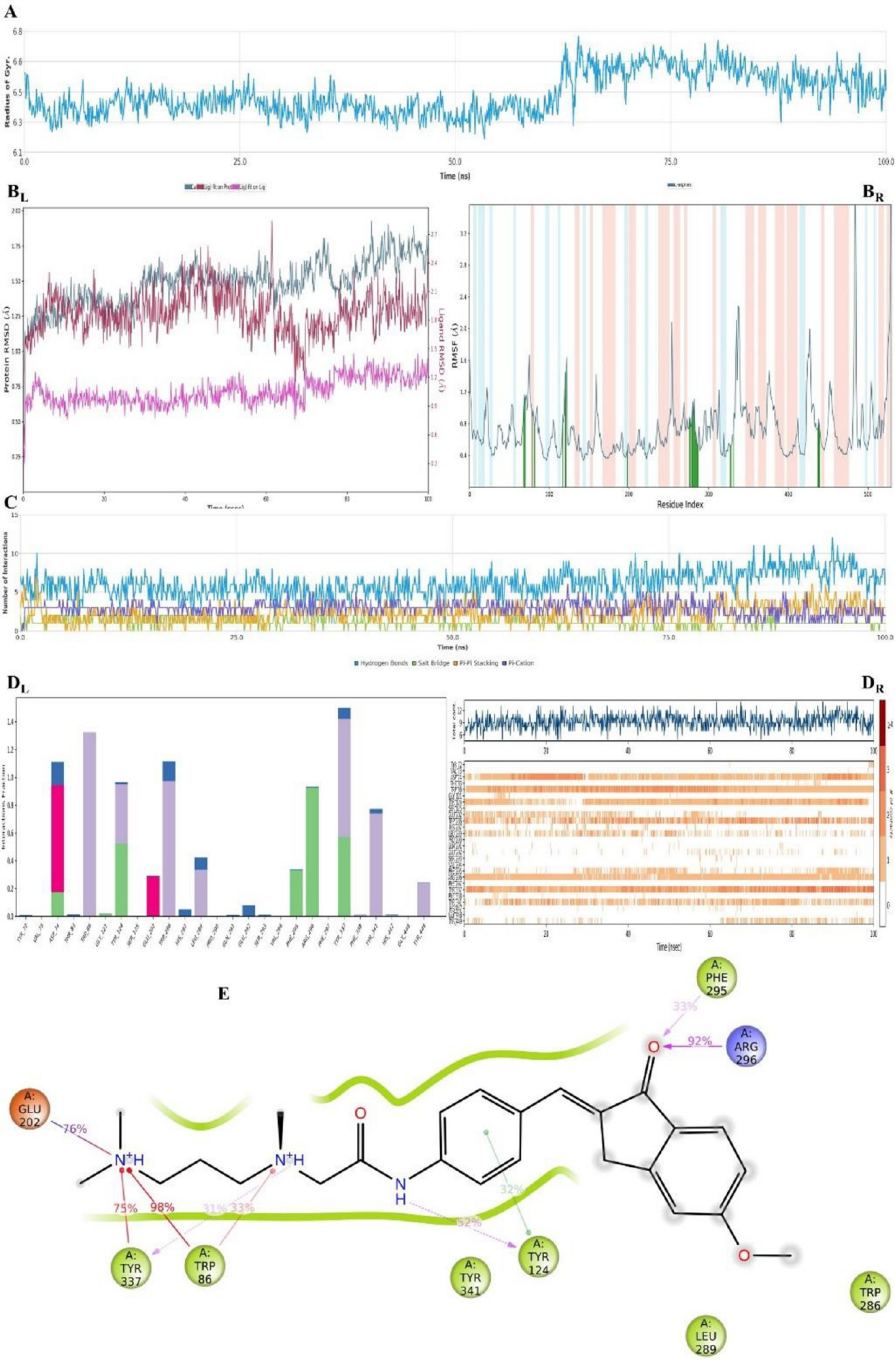
Plots of the MDS results for the compound **D37**–AChE enzyme complex. Stability properties: (A) Rg, (B_L_) RMSD, and (B_R_) RMSF plots. Interaction properties: (C) number of interactions–interaction types–time plot, (D)_L_) interaction fraction–residue diagram, (D_R_) total connections–residues–time plot, and (E) 2D interaction pose with the connection strength (cut off of 0.2) at the active region.

To understand the effects of environmental changes and classify the SAR more specifically, the MDS study was performed for both **D29**–AChE and **D37**–AChE enzyme complexes. The results are shown in Figures 19 and 20, respectively.

For both results, the stability of the systems was preserved (Figures 19A and B and 20A and B). When compound **D29** changed its conformation slightly around 25 ns (Supporting Video S1 and Figure 19), these changes were clearly seen from the RG plot, but they were not drastic changes. One possible explanation for why this happened is that when the interactions between compound **D29** and Phe338 ended, the interactions between compound **D29** and Tyr286 started at the same time. Moreover, similar fluctuations were observed from the Rg plot of the **D37**–AChE complex (see Figure 20 and Supporting Video S2). However, they were not drastic changes either.

According to Figures 19C–E, there were H-bonds, salt bridges, π–π stackings, and π–cation interactions. The interactions with Trp86 (π–cation) and Tyr341 (π–π stackings) were protected during the entire simulation. However, the interaction with Tyr341 was observed under 30% bond strength, so it was thought that it only contributed to the complex stability. The fact that the stability and inhibition activity were mostly related to interactions with Trp86, Tyr124, Gly120, Glu292, and Trp286, which was like donepezil. On the other hand, compound **D29** showed some hydrophobic interactions with Tyr72, Leu76, Leu130, Phe297, Phe338, Tyr341, and His447; some ionic interactions with Glu202 and Glu292; and some water-mediated H-bonds with Tyr72, Gly121, Glu202, Ser203, Glu292, Ser293, Arg296, Tyr337, Gly342, and His447 residues. Additionally, at around 65 ns, the interactions with Gly120 ended, while the interactions with His447 started. The acetylcholine was broken down into acetate and choline, which was catalyzed within the esteratic subsite (Ser203, Glu334, and His447).^61^ Owing to their position, these two created a region that allowed the ligand or substrate to bind to amino acid Ser203, and it is suggested that both amino acids have important roles in the hydrolase function of the AChE inhibition activity. As a result, the main reason for the **D29** inhibition activity, and thus complex stabilization, was mostly related to Trp86 and Tryp286 (CAS and PAS), Gly120, Tyr124, and Glu292. In addition to these H-bonds, there were also some aromatic H-bonds (cyan dashes in video) that formed between the ligand and amino acids Tyr124, Glu292, Phe295, Phe297, Tyr337, Phe338, and Gly342 (Supporting Video S1).

According to Figures 20C–E, there were H-bonds, salt bridges, π–π stackings, and π–cation interactions. The interactions with Asp74, Tyr86, Arg296, and Tyr337 were observed continuously. Moreover, the interactions with Trp86, Tyr124, Glu202, Phe295, Arg296, and Tyr337 were found above 30% bond strength. In addition to this, compound **D37** also interacted with the Trp286, Leu289, and Tyr449 residues via hydrophobic interactions. Moreover, it formed some water-mediated H-bonds with the Tyr72, Thr83, Leu289, Gln291, Glu292, Ser293, Tyr337, and Tyr341 residues. Furthermore, aromatic H-bonds (cyan dashes in the video) were also observed with Trp286, Gln291, Ser293, Tyr337, Phe338, and Tyr341, (Supporting Video S2).

In fact, even though the relation with Tyr286 was seen often, the bond stability was not quite enough because it did not have sufficient bond strength. However, it may have contributed to the inhibition activity by suffusing the pocket entrance, which may have caused the substrate to not reach the active region. In conclusion, the inhibition activity was mostly related to the CAS amino acids, but the stability was also supported by the PAS.

#### 3.5.2 Molecular Investigation of the Binding Modes on the MAO-B Enzyme

**Figure 21:**
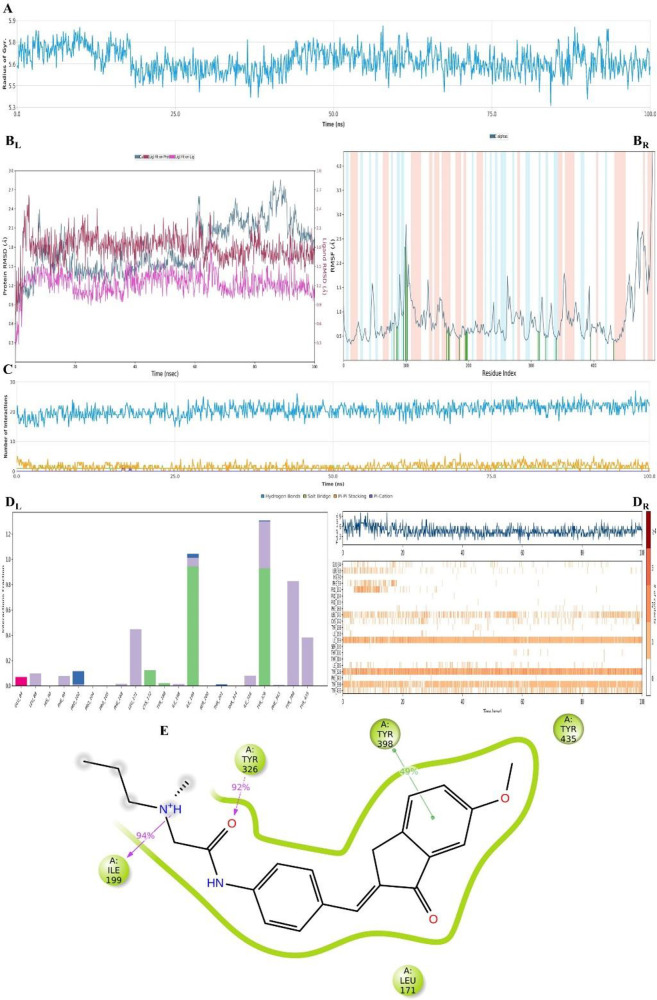
Plots of the MDS results for the compound **D29**–MAO-B enzyme complex. Stability properties: (A) Rg, (B_L_) RMSD, and (B_R_) RMSF plots. Interaction properties: (C) number of interactions–interaction types–time plot, (D)_L_) interaction fraction–residue diagram, (D_R_) total connections–residues–time plot, and (E) 2D interaction pose with the connection strength (cut off of 0.2) at the active region.

**Figure 22:**
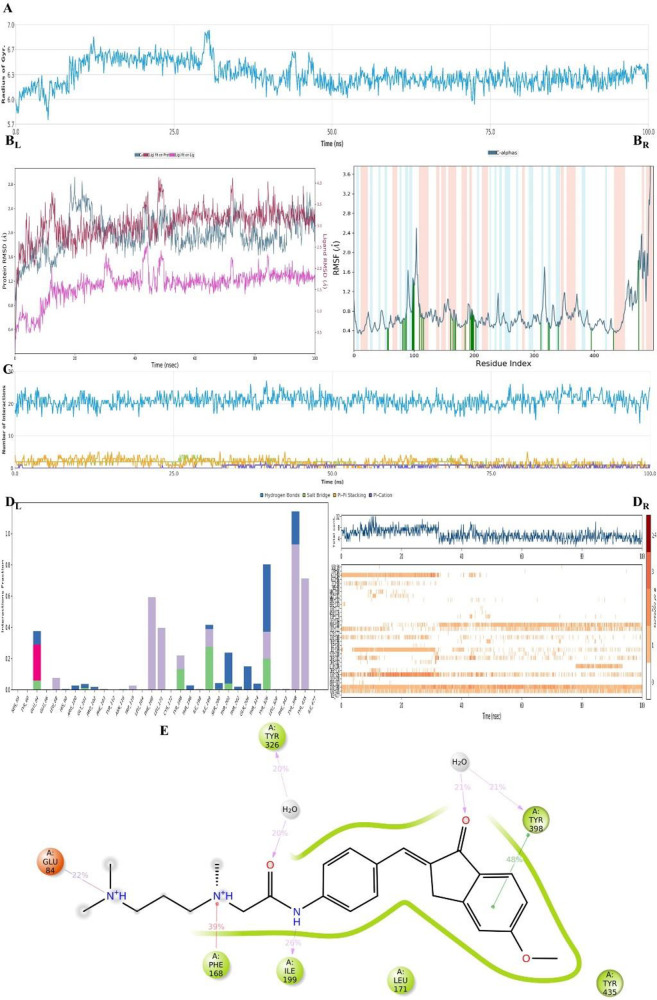
Plots of the MDS results for the compound **D37**–MAO-B enzyme complex. Stability properties: (A) Rg, (B_L_) RMSD, and (B_R_) RMSF plots. Interaction properties: (C) number of interactions–interaction types–time plot, (D)_L_) interaction fraction–residue diagram, (D_R_) total connections–residues–time plot, and (E) 2D interaction pose with the connection strength (cut off of 0.2) at the active region.

The MDS study was performed for MAO-B enzyme complexes of both compounds (**D29** and **D37**). The results are shown in Figures 21 and 22. For both compound–enzyme complexes, the stability was protected during the entire simulation (Figures 21A and B and 22A and B). Therefore, the interactions of the compounds were found to be reliable.

According to Figure 21C and D, there were H-bonds, salt bridges, π–π stackings, and π–cation interactions. Compound **D29** interacted with Ile199 (H-bond strength of 94%), Tyr326 (H-bond strength of 92%), and Tyr398 (π–π stacking strengt of : 49%) on an ongoing basis during the simulation; thus, it was suggested that these amino acids were arbiter residues on the MAO-B enzyme activity. Additionally, some connections were observed, but their interaction fractions were not as high as those of these three amino acids. In fact, only one ionic interaction (with Glu84) was observed between compound **D29** and the MAO-B enzyme. Moreover, the hydrophobic interactions were with amino acids Leu88, Phe99, Pro102, Phe168, Leu171, Ile198, Ile199, Ile316, Tyr326, Tyr398, and Tyr435. When the water-mediated H-bonds were observed with amino acids Pro102, Ile199, and Thr201, the direct H-bonds were seen with amino acids Cys172, Tyr188, Ile199, and Tyr326. Moreover, there were some aromatic H-bonds (cyan dashes in the video) that were formed between the ligand and the Ile198, Ile199, Tyr326, Tyr435, and FAD residues (Supporting Video S3).

According to Figures 22C–E, the interaction types of compound **D37** were the same as those of compound **D29**. However, the amino acids they interacted with were different. Even though compound **D37** connected with Ile199 (H-bond strength of 26%) and Tyr326 (water-mediated H-bond strength of 20%), these amino acids did not highly affect the MAO-B inhibitory activity in the same way as the activity of **D29**. In fact, the inhibitory activity was mostly related to interactions with amino acids Tyr398 and Tyr435. Moreover, at around 30 ns, the interactions with Glu84 ended when the interactions with the Phe168 residue started, but the stability of the complex was not affected as a result. However, the hydrophobic interactions were observed with the Leu88, Trp119, Phe168, Leu171, Tyr188, Ile198, Ile199, Tyr326, Tyr398, and Tyr435 residues. Additionally, when direct H-bonds were formed with amino acids Glu84, Gly101, Tyr188, Ile199, Thr201, and Tyr326, water-mediated H-bonds were observed with the Glu84, His90, Arg100, Gly101, Pro102, Thr196, Ser200, Thr201, Thr202, Gln206, Thr314, Tyr326, and Tyr398 residues. Meanwhile, there was only one ionic interaction, which was with Glu84. Furthermore, the aromatic H-bonds (cyan dashes in the video) were formed between the ligand and the Tyr60, Cys172, Phe168, Ile198, Gln206, Tyr326, Phe343, Tyr398, Tyr435, and FAD residues (Supporting Video S4).

According to the literature, all the mentioned amino acids were described as significant residues, but amino acids Tyr398 and Tyr435 were more attractive than the others because of their role in the enzyme mechanism.^21,22,24^ Although there were a few differences between the interactions of compounds **D29** and **D37**, the experimental studies for both compounds were supported by molecular docking and molecular dynamic simulations and explained how the compounds bonded to or stabilized the enzyme active pocket. Due to their interaction with the FAD protein and closing the cavity of the substrate binding region, it was found that their main action mechanism was mainly related to the above explanation, but their distinct activity was related to which amino acids they bonded to.

## 4 Conclusion

For possible utility in the treatment of AD, 42 novel indanone compounds were developed and synthesized in this research. With the use of IR, ^1^H NMR, ^13^C NMR, and mass spectroscopic techniques, the structures of the derived compounds were verified. In addition to studies on the biological activities of the final compounds in comparison to those of references, molecular modeling studies was carried out.

Accordingly, compounds **D19**–**D30** and **D34**–**D39** were determined to be effective derivatives against AChE. Molecular docking studies showed these molecules to have double binding properties similar to donepezil. Compounds **D28**–**D30** showed stronger interactions when compared to the other compounds and were the most effective derivatives, with low IC_50_ values. The kinetic studies determined that compound **D29** exhibited mixed inhibition of the enzyme. Compounds **D34**, **D35**, and **D37**–**D39** were determined to be effective derivatives against BChE. Among these, compounds **D37**–**D39** were determined to be the most effective derivatives, which was consistent with the molecular modeling studies. The kinetic studies determined that compound **D39** exhibited mixed inhibition of the enzyme. Compounds **D28** and **D29** were determined to be the most effective compounds against MAO-A. The docking studies showed that these compounds can very effectively bind to the active site of the MAO-A enzyme. The kinetic studies determined that compound **D28** exhibited noncompetitive inhibition of the enzyme. The studies conducted revealed that compounds **D28**–**D32** and **D37**–**D41** exhibited the strongest inhibition of MAO-B. Derivatives **D37**–**D39** showed stronger binding and interaction profiles with the enzyme active site when compared to the other compounds. This finding was compatible with the enzyme activity studies. The kinetic studies determined that compound **D39** exhibited noncompetitive inhibition of MAO-B. The activities of the target compounds were investigated using the DPPH antioxidant activity method to determine their oxidative stress profiles in the context of AD. Most of the compounds in the series showed very strong antioxidant activity. The Beta Amyloid 1–42 (Aβ42) Ligand Screening Assay kit was used to investigate the effects of compounds **D19**–**D30** and **D34**–**D39**, which strongly inhibited AChE and BChE on the amyloid plaques. The results suggested that compounds **D28**–**D30** and **D39** strongly inhibited β-amyloid plaque aggregation. The cytotoxicity of synthesized compounds **D19**–**D30** and **D34**–**D41**, which showed biological activity, was tested using the MTT method. The compounds in question did not cause toxicity at the studied concentrations.

AChE and MAO-B inhibitors can individually be effective in the treatment of AD. Substances that can simultaneously inhibit both enzymes (dual inhibition) are prominent and valuable for the treatment of AD. Among the synthesized compounds, **D28**–**D30** showed inhibitory activity against both AChE and MAO-B and thus emerged as compounds that could be effective in the treatment of AD. In addition, compounds **D37**–**D39**, which were active against MAO-B, are promising for the treatment of Parkinson’s disease. Future research should seek to develop, manufacture, and study the activities of new and more powerful compounds with comparable chemical structures in light of the findings of the activity and molecular modeling investigations undertaken here.

## Supporting Information Available

Biological activity evaluation, prediction of ADME parameters, molecular docking studies, and analytical results of the compounds (PDF)
**D29–AChE** (AVI)
**D37–AChE** (AVI)
**D29–MAO**-**B** (AVI)
**D37–MAO**-**B** (AVI)

## Supplementary Material

ao2c06906_si_001.pdf

ao2c06906_si_002.avi

ao2c06906_si_003.avi

ao2c06906_si_004.avi

ao2c06906_si_005.avi
