# Supplementary material for: Design, Synthesis, and In Vitro and In Silico Approaches of Novel Indanone Derivatives as Multifunctional Anti-Alzheimer Agents
Source: ACS Omega. 2022 Dec 7;7(50):47378–404. doi: 10.1021/acsomega.2c06906 (PMC9774391; doi:10.1021/acsomega.2c06906)
Supplement: Supplementary file 1 — ao2c06906_si_001.pdf [file ao2c06906_si_001.pdf]

SUPPORTING INFORMATION

Design, synthesis, in vitro and in silico approaches of novel indanone derivatives as multifunctional anti-Alzheimer agents

Begüm Nurpelin Sağlık[a,b,*], Serkan Levent[a,b], Derya Osmaniye[a,b], Asaf Evrim Evren[a,c], Abdullah Burak Karaduman[d], Yusuf Özkay[a,b], Zafer Asım Kaplancıklı[a]

[a]*Department of Pharmaceutical Chemistry, Faculty of Pharmacy, Anadolu University, Eskişehir, Turkey*

[b]*Central Research Laboratory (MERLAB), Faculty of Pharmacy, Anadolu University, Eskişehir, Turkey*

[c]*Department of Pharmacy Services, Vocational School of Health Services, Bilecik Şeyh Edebali University, Bilecik, Turkey*

[d]*Department of Pharmaceutical Toxicology, Faculty of Pharmacy, Anadolu University, Eskişehir, Turkey*

* Corresponding author

E-mail address: bnsaglik@anadolu.edu.tr (B.N. Sağlık)

Tel: +90-222-3350580/3774, *Fax*: +90-222-3350750

Address: Anadolu University, Faculty of Pharmacy, Department of Pharmaceutical Chemistry, 26470, Eskişehir, Turkey.

1. Biological activity evaluation

Table S1. % Inhibition of the synthesized compounds, donepezil and tacrine against AChE and BChE.

Compounds	Human AChE Inhibition %		Human BChE Inhibition %	
	10^{-3} M	10^{-4}M	10^{-3} M	10^{-4}M
**D1**	**83.204 ± 1.087**	47.270 ± 0.887	**94.585 ± 0.972**	32.756 ± 0.455
**D2**	**50.345 ± 0.731**	42.927 ± 0.601	**61.303 ± 0.180**	23.599 ± 0.226
**D3**	**99.263 ± 2.338**	44.144 ± 0.906	**98.127 ± 1.567**	40.425 ± 0.548
**D4**	**74.466 ± 1.019**	38.187 ± 0.479	**92.856 ± 2.411**	21.206 ± 0.110
**D5**	38.630 ± 0.518	31.715 ± 0.499	**55.589 ± 0549**	26.295 ± 0.211
**D6**	**97.472 ± 2.235**	31.298 ± 0.697	**96.511 ± 0.317**	32.801 ± 0.908
**D7**	**61.340 ± 1.007**	34.801 ± 0.472	**56.779 ± 0.971**	21.198 ± 0.378
**D8**	**51.014 ± 0.995**	41.963 ± 0.661	**51.038 ± 0.667**	28.437 ± 0.409
**D9**	**81.316 ± 1.128**	45.181 ± 0.996	**82.834 ± 1.026**	28.169 ± 0.679
**D10**	**71.341 ± 1.012**	29.397 ± 0.587	41.288 ± 0.755	26.603 ± 1.349
**D11**	**66.572 ± 0.985**	49.906 ± 0.809	**91.661 ±1.205**	33.867 ± 0.569
**D12**	**96.538 ± 2.702**	49.674 ± 0.988	**98.437 ± 1.789**	48.756 ± 0.529
**D13**	**71.220 ± 1.075**	42.997 ± 0.998	**90.132 ± 1.459**	23.872 ± 0.934
**D14**	44.187 ± 0.999	36.281 ± 0.664	**73.658 ± 1.506**	33.015 ± 0.721
**D15**	**91.618 ± 2.563**	43.422 ± 0.877	**94.575 ± 1.603**	43.516 ± 0.966
**D16**	**88.026 ± 1.091**	42.630 ± 1.850	**87.633 ± 0.618**	23.618 ± 0.448
**D17**	**73.714 ± 1.057**	48.056 ± 0.669	**75.532 ± 0.061**	21.065 ± 0.804
**D18**	**94.686 ± 1.973**	45.304 ± 1.007	**99.207 ± 0.887**	41.754 ± 0.766
**D19**	**95.234 ± 1.652**	**92.743 ± 1.253**	**83.484 ± 1.368**	32.541 ± 0.337
**D20**	**98.265 ± 1.112**	**91.115 ± 1.365**	**82.569 ± 1.048**	20.069 ± 0.277
**D21**	**95.659 ± 1.198**	**91.743 ± 1.466**	**99.740 ± 2.822**	40.588 ± 0.978
**D22**	**91.897 ± 1.468**	**86.624 ± 1.567**	**99.494 ± 1.226**	39.459 ± 0.970
**D23**	**92.458 ± 1.568**	**87.634 ± 1.677**	**98.983 ± 1.678**	41.485 ± 0.741
**D24**	**93.224 ± 1.719**	**90.486 ± 1.298**	**97.932 ± 2.016**	49.401 ± 0.559
**D25**	**92.555 ± 1.623**	**88.713 ± 1.185**	**95.732 ± 2.131**	32.005 ± 0.667
**D26**	**93.294 ± 1.498**	**80.485 ± 1.208**	**56.178 ± 0.826**	31.070 ± 0.415
**D27**	**91.220 ± 1.298**	**87.293 ± 1.118**	**94.828 ± 0.731**	35.863 ± 0.444
**D28**	**98.627 ± 1.207**	**95.284 ± 1.311**	**61.288 ± 0.997**	36.636 ± 0.667
**D29**	**97.184 ± 1.318**	**94.285 ± 1.458**	**61.128 ± 0.990**	26.914 ± 0.019
**D30**	**98.211 ± 1.366**	**96.552 ± 1.285**	**99.551 ± 2.015**	46.430 ± 0.408
**D31**	41.288 ± 0.966	27.107 ± 0.803	**98.767 ± 2.689**	41.566 ± 0.988
**D32**	33.750 ± 0.885	26.356 ± 0.565	**90.854 ± 1.288**	24.694 ± 0.455
**D33**	**52.441 ± 1.236**	31.964 ± 0.462	**99.019 ± 1.755**	46.563 ± 0.845
**D34**	**98.265 ± 1.112**	**91.115 ± 1.365**	**91.635 ± 1.028**	**84.778 ± 1.249**
**D35**	**95.659 ± 1.198**	**91.743 ± 1.466**	**90.168 ± 1.388**	**81.242 ± 1.245**
**D36**	**91.897 ± 1.468**	**86.624 ± 1.567**	**99.979 ± 1.249**	41.018 ± 0.608
**D37**	**92.458 ± 1.568**	**87.634 ± 1.677**	**94.227 ± 1.391**	**89.716 ± 1.247**
**D38**	**93.224 ± 1.719**	**90.486 ± 1.298**	**93.266 ± 1.374**	**90.553 ± 1.299**

**D39**	**92.555 ± 1.623**	**88.713 ± 1.185**	**92.759 ± 1.365**	**87.290 ± 1.247**
**D40**	**81.266 ± 1.112**	41.557 ± 0.995	**95.128 ± 1.244**	44.328 ± 0.642
**D41**	**80.969 ± 1.598**	36.590 ± 0.399	**99.149 ± 0.704**	46.331 ± 1.367
**D42**	**99.818 ± 2.008**	44.208 ± 0.710	**99.413 ± 1.293**	45.905 ± 0.206
**Donepezil**	99.156 ± 1.302	97.395 ± 1.255	-	-
**Tacrine**	-	-	99.827 ± 1.378	98.651 ± 1.402

Table S2. % Inhibition of the synthesized compounds, moclobemide and selegiline against MAO-A and MAO-B.

Compounds	Human MAO-A Inhibition %		Human MAO-B Inhibition %	
	10^{-3} M	10^{-4}M	10^{-3} M	10^{-4}M
**D1**	**59.022 ± 0.598**	21.358 ± 0.424	**65.123 ± 0.623**	38.652 ± 0.512
**D2**	46.596 ± 0.853	27.718 ± 0.909	**75.268 ± 0.987**	45.327 ± 0.859
**D3**	**57.517 ± 0.875**	26.480 ± 0.365	**67.248 ± 0.751**	36.244 ± 0.452
**D4**	**67.637 ± 1.058**	20.921 ± 0.455	**77.148 ± 1.059**	36.285 ± 0.496
**D5**	**66.594 ± 1.487**	24.732 ± 0.514	**75.629 ± 1.085**	44.367 ± 0.627
**D6**	**93.265 ± 0.309**	33.855 ± 1.132	**95.456 ± 1.248**	48.623 ± 1.132
**D7**	**61.886 ± 0.618**	41.402 ± 0.808	**66.327 ± 0.557**	47.895 ± 0.952
**D8**	**57.153 ± 1.338**	26.647 ± 0.455	**78.628 ± 1.148**	46.221 ± 0.547
**D9**	32.570 ± 0.389	24.514 ± 0.459	**58.627 ± 0.523**	34.759 ± 0.384
**D10**	48.926 ± 0.966	22.424 ± 0.662	**68.956 ± 0.578**	42.627 ± 0.652
**D11**	**76.981 ± 1.588**	25.497 ± 0.520	**79.624 ± 1.128**	35.627 ± 0.488
**D12**	**95.759 ± 0.490**	45.146 ± 0.466	**96.236 ± 1.338**	48.962 ± 0.623
**D13**	**82.658 ± 1.210**	35.532 ± 0.6232	**84.627 ± 1.114**	44.622 ± 0.557
**D14**	48.492 ± 0.749	27.945 ± 0.243	**68.975 ± 0.847**	39.337 ± 0.652
**D15**	**70.522 ± 0.536**	20.255 ± 0.412	**80.221 ± 1.456**	40.118 ± 0.508
**D16**	**65.764 ± 0.779**	22.015 ± 0.233	**75.628 ± 1.102**	42.875 ± 0.629
**D17**	**82.451 ± 0.529**	34.394 ± 0.508	**84.627 ± 1.045**	45.378 ± 0.669
**D18**	**63.834 ± 0.683**	28.393 ± 0.097	**75.627 ± 1.204**	47.336 ± 0.457
**D19**	**60.937 ± 1.865**	25.327 ± 0.632	**90.237 ± 1.455**	43.175 ± 0.877
**D20**	**81.865 ± 0.243**	43.291 ± 0.429	**83.624 ± 1.045**	41.627 ± 0.508
**D21**	**91.205 ± 1.208**	26.946 ± 0.541	**93.517 ± 1.375**	46.208 ± 0.587
**D22**	**76.898 ± 0.232**	37.460 ± 0.411	**85.345 ± 0.957**	48.627 ± 0.605
**D23**	**78.171 ± 0.969**	22.933 ± 0.497	**81.324 ± 1.046**	43.248 ± 0.884
**D24**	**64.076 ± 0.323**	25.257 ± 0.621	**75.628 ± 1.426**	46.324 ± 0.521
**D25**	**80.381 ± 0.646**	43.105 ± 0.646	**78.956 ± 1.308**	40.112 ± 0.630
**D26**	**95.124 ± 0.828**	44.349 ± 0.914	**94.268 ± 0.997**	41.345 ± 0.874
**D27**	**95.085 ± 0.687**	44.381 ± 2.292	**94.628 ± 1.025**	43.126 ± 0.452
**D28**	**89.622 ± 1.288**	**81.755 ± 1.305**	**92.551 ± 1.334**	**89.261 ± 1.241**
**D29**	**90.224 ± 1.358**	**84.633 ± 1.247**	**91.822 ± 1.368**	**87.593 ± 1.249**
**D30**	**74.057 ± 2.094**	28.267 ± 1.077	**97.513 ± 1.388**	**90.225 ± 1.471**
**D31**	**92.739 ± 1.309**	23.855 ± 0.572	**93.568 ± 1.477**	**88.717 ± 1.356**
**D32**	**76.742 ± 1.666**	29.662 ± 0.752	**94.777 ± 1.421**	**89.638 ± 1.266**
**D33**	**84.292 ± 1.288**	29.910 ± 0.552	**82.128 ± 1.185**	39.264 ± 0.665
**D34**	**82.123 ± 1.246**	38.288 ± 0.458	**78.456 ± 1.308**	47.265 ± 0.676
**D35**	**88.458 ± 0.758**	21.678 ± 0.623	**90.652 ± 1.245**	42.526 ± 0.547
**D36**	**82.441 ± 0.873**	42.985 ± 0.771	**84.627 ± 1.014**	45.238 ± 0.584
**D37**	**96.511 ± 0.736**	37.778 ± 0.960	**98.398 ± 1.629**	**91.559 ± 1.238**
**D38**	**94.976 ± 2.393**	24.067 ± 0.853	**94.796 ± 1.429**	**88.636 ± 1.375**
**D39**	**89.769 ± 1.437**	36.216 ± 0.756	**95.294 ± 1.059**	**87.214 ± 1.821**
**D40**	**84.265 ± 1.267**	41.275 ± 0.577	**92.88/ ± 1.236**	**88.757 ± 1.301**

**D41**	**82.827 ± 2.121**	22.505 ± 0.622	**90.177 ± 1.365**	**81.794 ± 1.288**
**D42**	**97.714 ± 0.411**	40.176 ± 0.736	**95.214 ± 1.624**	40.177 ± 0.824
**Moclobemide**	94.121 ± 2.760	82.143 ± 2.691	-	-
**Selegiline**	-	-	98.589 ± 2.055	94.850 ± 1.114

2. Prediction of ADME parameters

Table S3. Calculated ADME parameters of compounds **D1-D42**.

Comp.	MW	RB	DM	MV	DHB	AHB	PSA	logP	logS	PCaco	logBB	PMDCK	CNS	PM	%HOA	VRF	VRT
**D1**	405.496	6	6.661	1335.401	1	9.25	84.767	2.287	-3.147	67.065	-0.133	32.636	1	5	73.028	0	0
**D2**	405.496	6	5.480	1335.408	1	9.25	84.772	2.287	-3.147	67.065	-0.133	32.636	1	5	73.028	0	0
**D3**	435.522	7	7.386	1391.745	1	10	90.141	2.247	-3.053	67.980	-0.186	33.117	1	6	72.898	0	0
**D4**	419.522	7	7.771	1383.038	1	9.25	81.156	2.611	-3.345	74.348	-0.149	36.483	1	5	75.724	0	0
**D5**	419.522	7	9.774	1383.001	1	9.25	81.156	2.610	-3.343	74.348	-0.149	36.483	1	5	75.722	0	0
**D6**	449.549	8	6.401	1444.592	1	10	88.554	2.588	-3.378	71.730	-0.243	35.096	1	6	75.312	0	0
**D7**	431.533	8	6.970	1428.903	1	9.25	84.404	2.947	-3.621	74.858	-0.228	36.753	1	6	77.749	0	0
**D8**	431.533	8	5.881	1428.909	1	9.25	84.409	2.948	-3.620	74.858	-0.227	36.753	1	6	77.749	0	0
**D9**	461.560	9	6.790	1489.734	1	10	89.601	2.923	-3.688	73.057	-0.321	35.798	1	7	77.415	0	1
**D10**	429.518	8	8.218	1410.024	1.5	9.25	84.494	2.783	-3.622	69.091	-0.281	33.702	1	6	76.164	0	0
**D11**	429.518	8	6.488	1410.031	1.5	9.25	84.499	2.783	-3.621	69.091	-0.280	33.702	1	6	76.164	0	0
**D12**	459.544	9	6.139	1476.960	1.5	10	92.622	2.811	-3.741	68.977	-0.363	33.643	1	7	76.313	0	1
**D13**	449.549	9	6.749	1479.169	1	10.95	90.715	2.480	-3.314	76.691	-0.308	37.727	1	6	75.200	0	0
**D14**	449.549	9	8.767	1479.133	1	10.95	90.714	2.480	-3.312	76.691	-0.307	37.727	1	6	75.198	0	0
**D15**	479.575	10	6.847	1544.724	1	11.70	94.259	2.497	-3.324	77.879	-0.367	38.359	1	7	75.416	0	1
**D16**	435.522	9	6.830	1401.934	2	10.95	101.693	1.645	-2.684	28.298	-0.776	12.842	0	6	62.563	0	0
**D17**	435.522	9	5.901	1401.940	2	10.95	101.698	1.645	-2.683	28.298	-0.776	12.842	0	6	62.563	0	0
**D18**	465.548	10	9.129	1477.098	2	11.70	112.653	1.726	-2.920	28.595	-0.878	12.988	0	7	63.113	0	1
**D19**	449.549	10	6.765	1445.434	2	10.95	98.863	1.971	-2.751	34.200	-0.730	15.760	0	6	65.942	0	0
**D20**	449.549	10	5.324	1445.441	2	10.95	98.868	1.971	-2.751	34.200	-0.730	15.760	0	6	65.942	0	0
**D21**	479.575	11	5.985	1530.494	2	11.70	112.066	1.961	-3.241	22.244	-1.111	9.900	-2	7	62.537	0	1

**D22**	462.591	9	4.557	1533.843	1	11.25	85.838	2.144	-2.339	18.103	-0.339	8.767	1	6	62.009	0	1
**D23**	462.591	9	7.333	1533.925	1	11.25	85.416	2.144	-2.330	18.094	-0.338	8.762	1	6	62.005	0	1
**D24**	492.617	10	8.673	1601.074	1	12	96.162	2.164	-2.392	18.256	-0.408	8.847	1	7	62.194	0	1
**D25**	476.617	10	9.610	1585.725	1	11.25	85.227	2.467	-2.642	18.205	-0.416	8.820	1	6	63.944	0	1
**D26**	476.617	10	7.648	1585.732	1	11.25	85.232	2.467	-2.641	18.205	-0.416	8.820	1	6	63.944	0	1
**D27**	506.644	11	7.440	1629.931	1	12	90.634	2.379	-2.434	19.749	-0.428	9.631	1	7	51.105	1	1
**D28**	378.470	8	8.995	1279.532	1	7.25	75.650	3.378	-4.318	324.931	-0.535	162.380	1	4	91.680	0	0
**D29**	378.470	8	7.372	1279.539	1	7.25	75.655	3.378	-4.317	324.931	-0.535	162.380	1	4	91.681	0	0
**D30**	408.496	9	7.518	1345.665	1	8	83.817	3.402	-4.430	324.934	-0.615	162.382	1	5	91.819	0	0
**D31**	374.438	8	4.446	1237.956	1	7.25	76.075	3.102	-4.035	294.071	-0.580	145.777	1	5	89.290	0	0
**D32**	374.438	8	6.262	1237.920	1.5	7.25	76.075	3.102	-4.033	294.071	-0.579	145.777	1	5	89.288	0	0
**D33**	404.465	9	7.125	1304.044	1.5	8	84.237	3.144	-4.135	306.478	-0.635	152.436	1	6	89.858	0	0
**D34**	407.511	9	7.959	1349.338	1	9.25	77.484	2.470	-2.426	92.136	-0.105	46.002	1	5	76.567	0	0
**D35**	407.511	9	6.883	1349.344	1	9.25	77.489	2.470	-2.426	92.136	-0.105	46.002	1	5	76.568	0	0
**D36**	437.538	10	8.566	1426.134	1	10	88.608	2.500	-2.834	76.882	-0.312	37.828	1	6	75.336	0	0
**D37**	421.538	10	8.825	1416.826	1	9.25	79.090	2.809	-3.119	72.440	-0.350	35.472	1	5	76.685	0	0
**D38**	421.538	10	7.408	1416.833	1	9.25	79.095	2.809	-3.119	72.440	-0.350	35.472	1	5	76.685	0	0
**D39**	451.564	11	7.648	1472.576	1	10	85.606	2.795	-2.963	79.680	-0.353	39.319	1	6	77.342	0	0
**D40**	449.592	12	7.142	1497.058	1	9.25	78.087	3.476	-3.125	115.575	-0.206	58.773	1	5	84.218	0	0
**D41**	449.592	12	5.915	1497.065	1	9.25	78.092	3.476	-3.125	115.575	-0.206	58.773	1	5	84.218	0	0
**D42**	479.618	13	5.977	1581.021	1	10	84.801	3.535	-3.535	96.266	-0.403	48.235	1	6	83.146	0	0

MW: Molecular weight; **RB:** Number of rotatable bonds (recommended value: 0-15); **DM:** Computed dipole moment of the molecule (recommended value: 1-12.5); **MV:** Total solvent-accessible volume in cubic angstroms using a probe with a 1.4 Å radius (recommended value: 500-2000); **DHB:** Estimated number of hydrogen bonds that would be donated by the solute to water molecules in an aqueous solution (recommended value: 0-6); **AHB:** Estimated number of hydrogen bonds that would be accepted by the solute from water molecules in an aqueous solution (recommended value: 2-20); **PSA:** Van der Waals surface area of polar nitrogen and oxygen atoms and carbonyl carbon atoms (recommended value: 7-200); **logP:** Predicted octanol/water partition coefficient (recommended value: -2-6.5); **logS:** Predicted aqueous solubility, log S. S in mol dm^{-3} is the concentration of the solute in a saturated solution that is in equilibrium with the crystalline solid (recommended value: -6.5-0.5); **PCaco:** Predicted apparent Caco-2 cell permeability in nm/sec. Caco-2 cells are a model for the gut-blood barrier.

QikProp predictions are for non-active transport (recommended value: <25 poor, >500 great); **logBB:** Predicted brain/blood partition coefficient (recommended value: -3-1.2); **PMDCK:** Predicted apparent MDCK cell permeability in nm/sec. MDCK cells are considered to be a good mimic for the bloodbrain barrier. QikProp predictions are for non-active transport (recommended value: <25 poor, >500 great); **PM:** Number of likely metabolic reactions (recommended value: 1-8); **CNS:** Predicted central nervous system activity on a –2 (inactive) to +2 (active) scale (recommended value: –2 (inactive), +2 (active)); **%HOA:** Predicted human oral absorption on 0 to 100% scale. The prediction is based on a quantitative multiple linear regression model. This property usually correlates well with Human Oral-Absorption, as both measure the same property (recommended value: >80% is high, <25% is poor); **VRF:** Number of violations of Lipinski's rule of five. The rules are: mol_MW < 500, QPlogPo/w < 5, donorHB ≤ 5, accptHB≤ 10. Compounds that satisfy these rules are considered druglike. (The "five" refers to the limits, which are multiples of 5.) (maximum is 4); **VRT:** Number of violations of Jorgensen's rule of three. The three rules are: QPlogS > -5.7, QP PCaco > 22 nm/s, # Primary Metabolites < 7. Compounds with fewer (and preferably no) violations of these rules are more likely to be orally available (maximum is 3).

3. Molecular docking studies

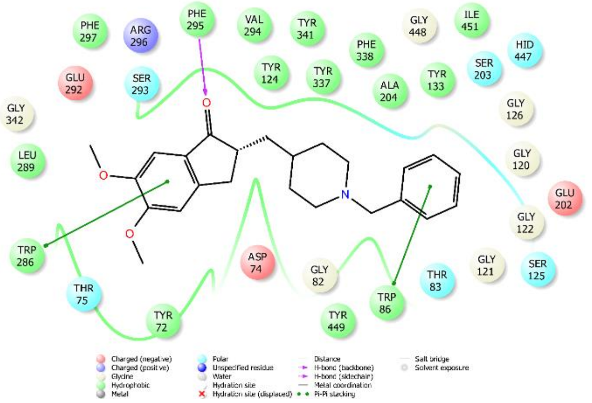

Donepezil

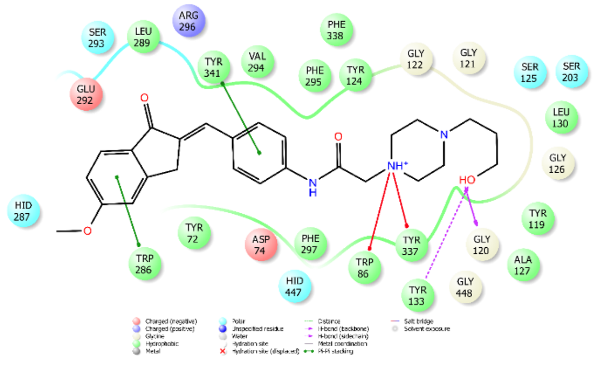

D19

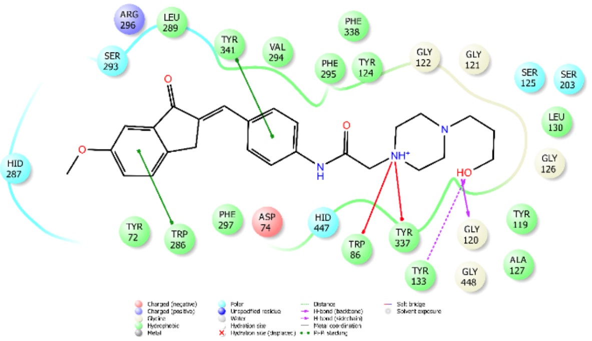

D20

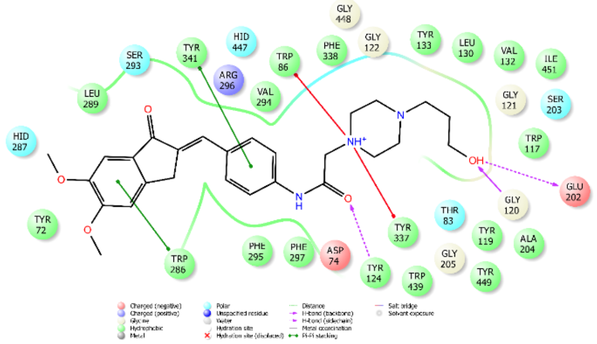

D21

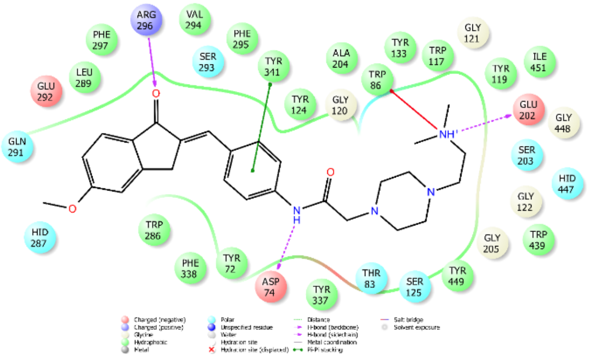

D22

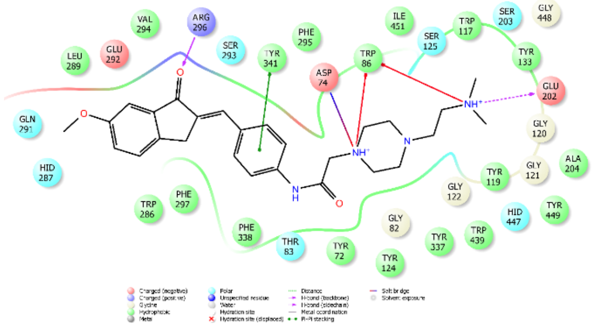

D23

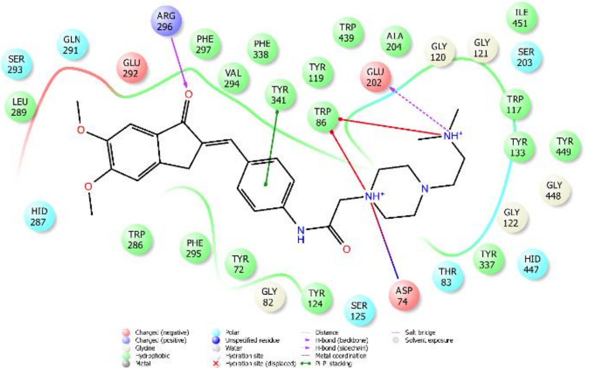

D24

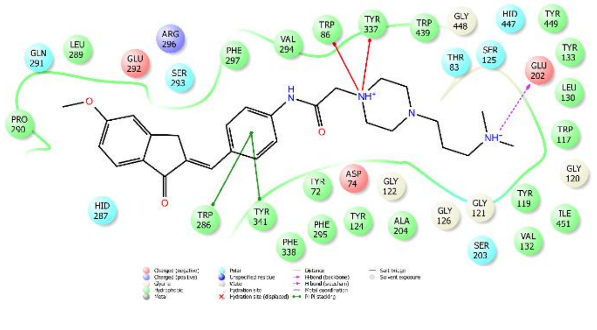

D25

Figure S1. The two-dimensional interacting mode of compounds **D19-D30**, **D34-D39** and donepezil in the active region of AChE.

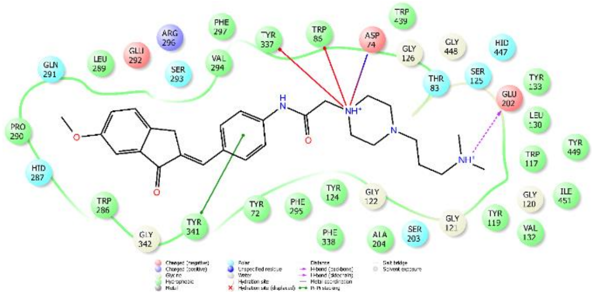

D26

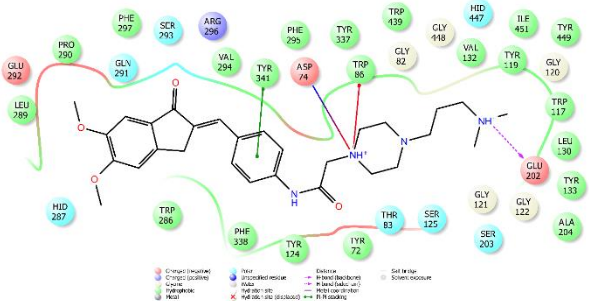

D27

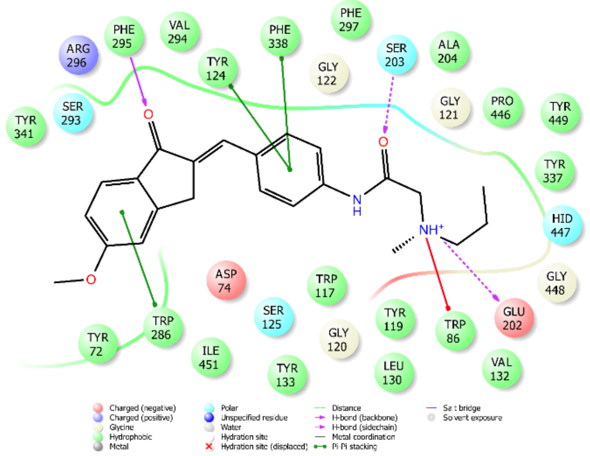

D28

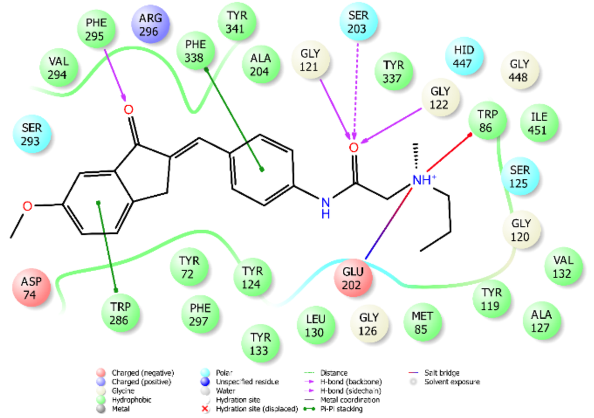

D29

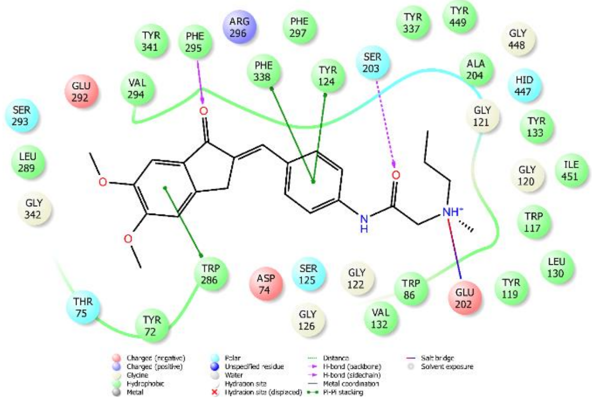

D30

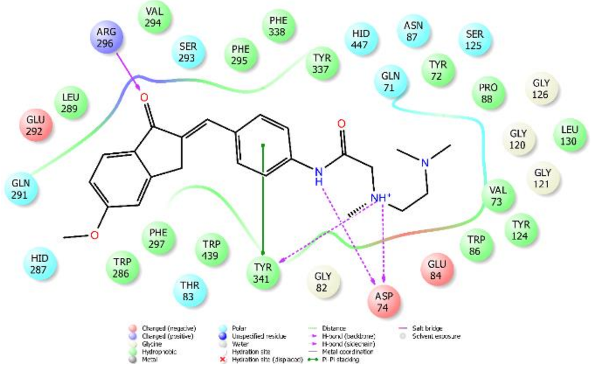

D34

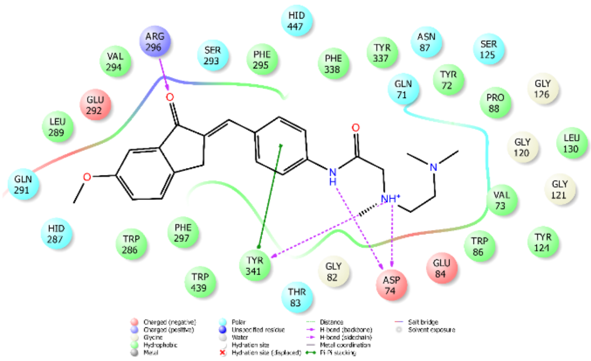

D35

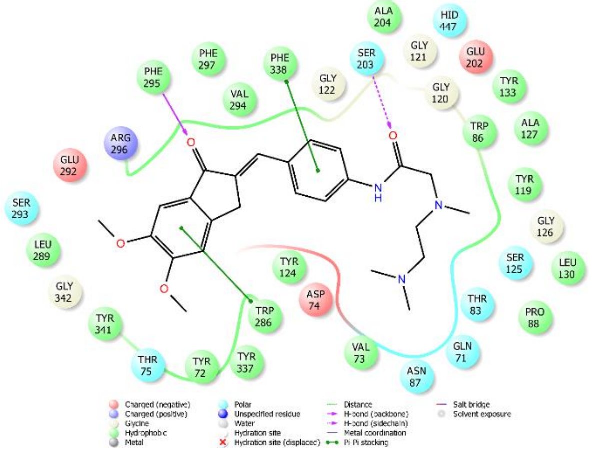

D36

Figure S1 (continued)**:** The two-dimensional interacting mode of compounds **D19-D30**, **D34-D39** and donepezil in the active region of AChE.

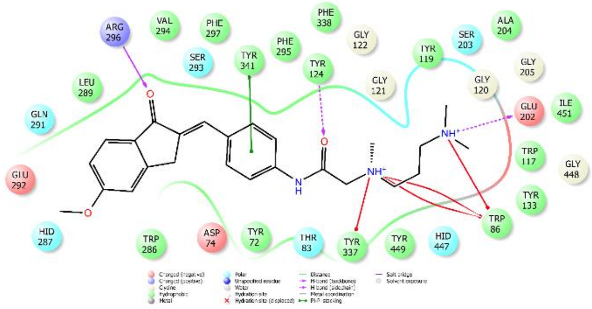

D37

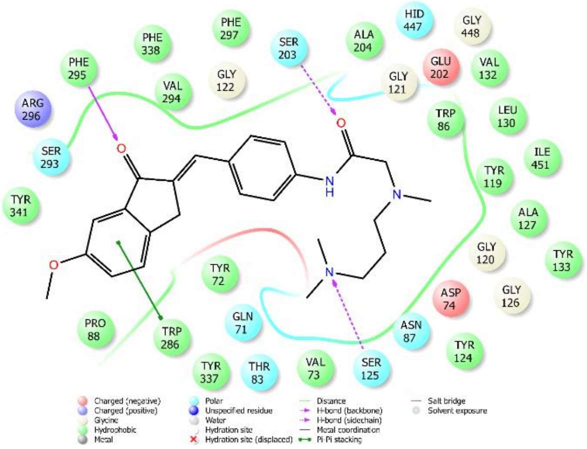

D38

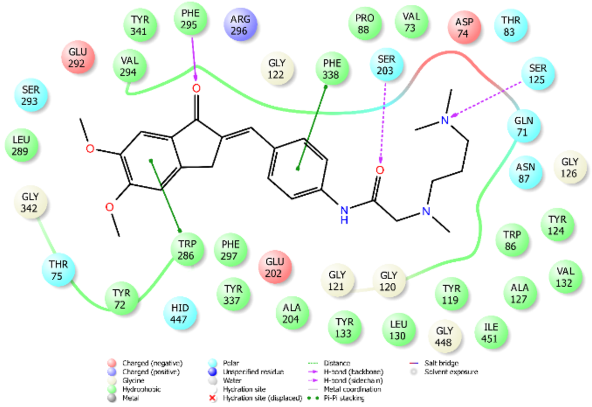

D39

Figure S1 (continued)**:** The two-dimensional interacting mode of compounds **D19**-**D30**, **D34**-**D39** and donepezil in the active region of AChE.

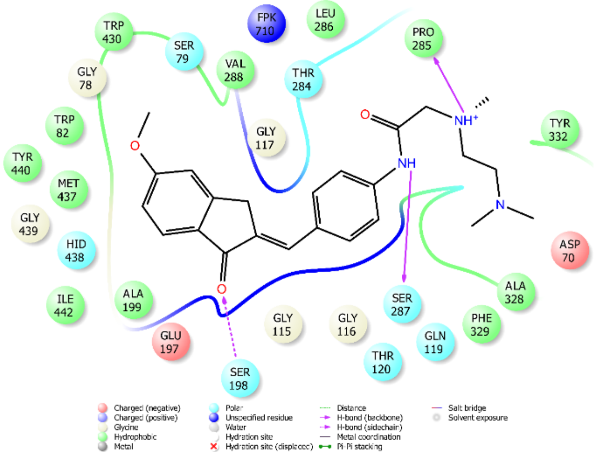

D34

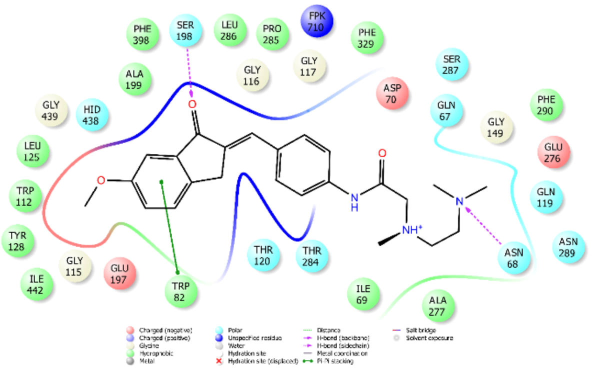

D35

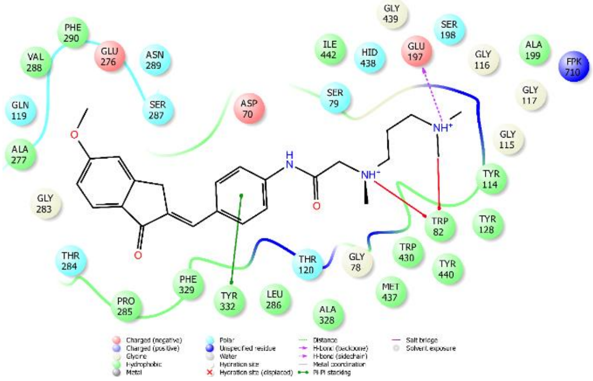

D37

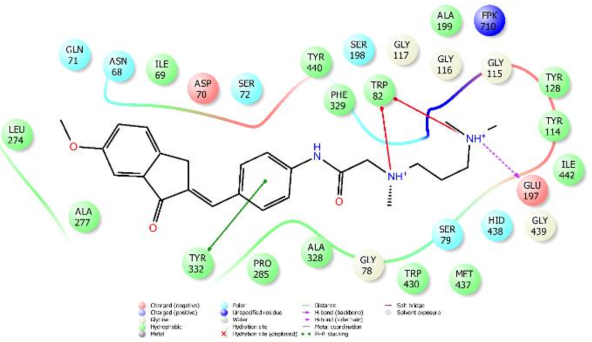

D38

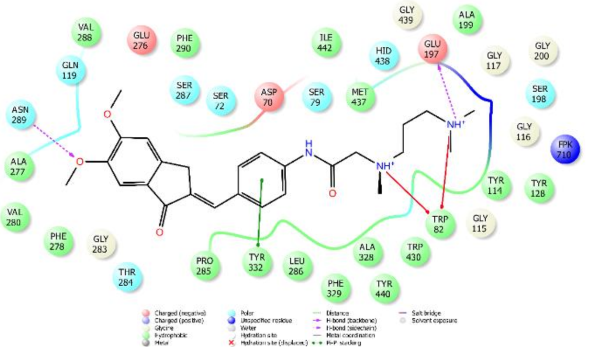

D39

Figure S2. The two-dimensional interacting mode of compounds **D34**, **D35**, **D37-D39** in the active region of BChE.

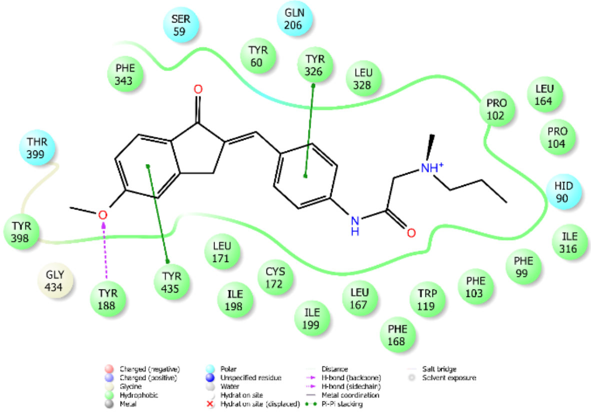

D28

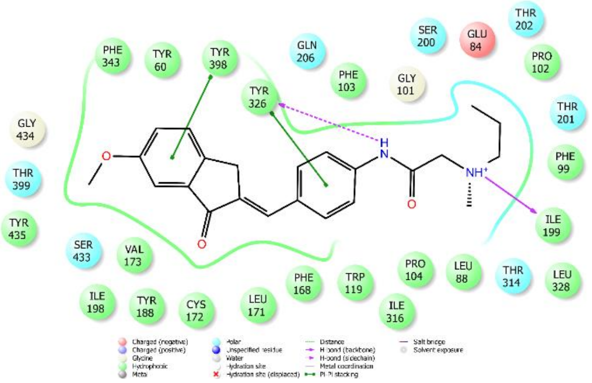

D29

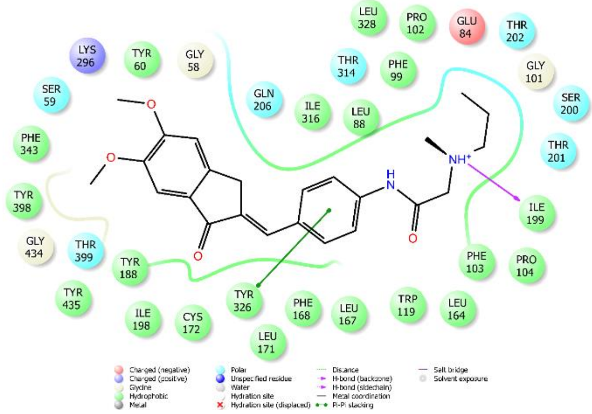

D30

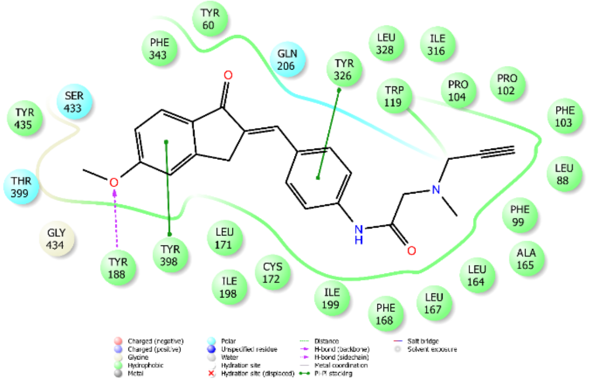

D31

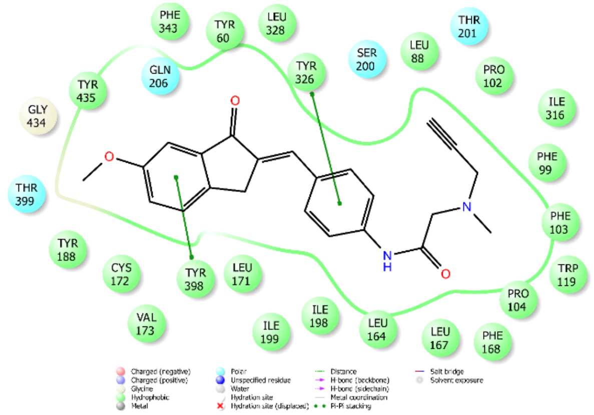

D32

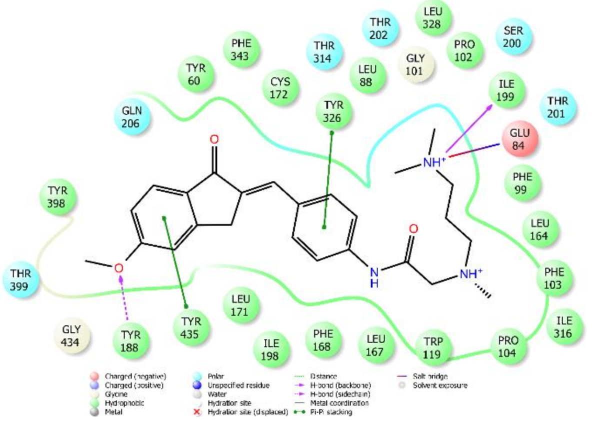

D37

Figure S3. The two-dimensional interacting mode of compounds **D28-D32**, **D37-D41** in the active region of MAO-B.

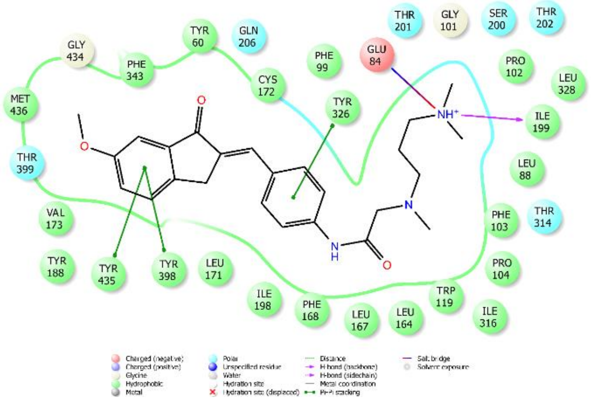

D38

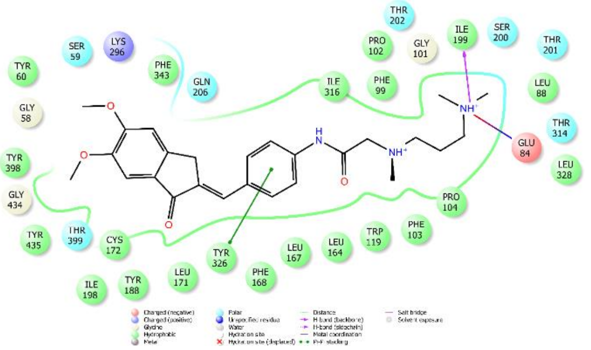

D39

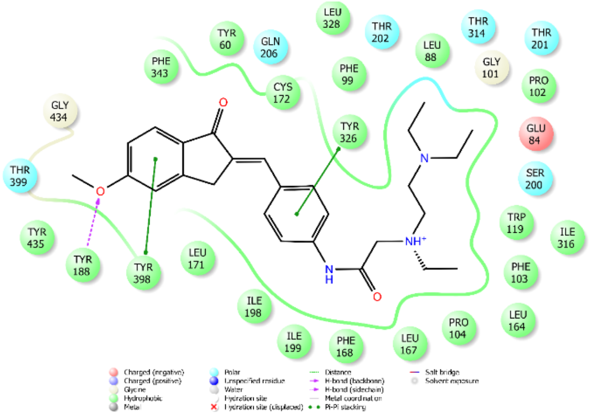

D40

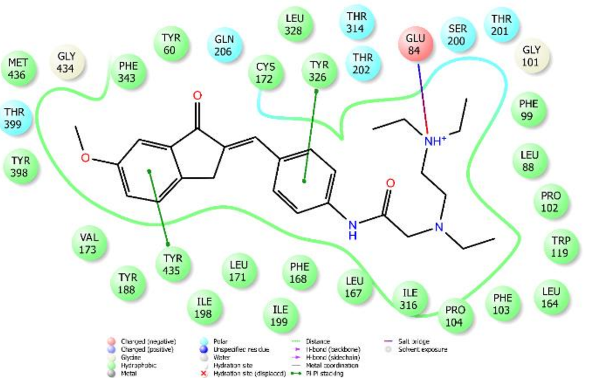

D41

Figure S3 (continued)**:** The two-dimensional interacting mode of compounds **D28-D32**, **D37-D41** in the active region of MAO-B.

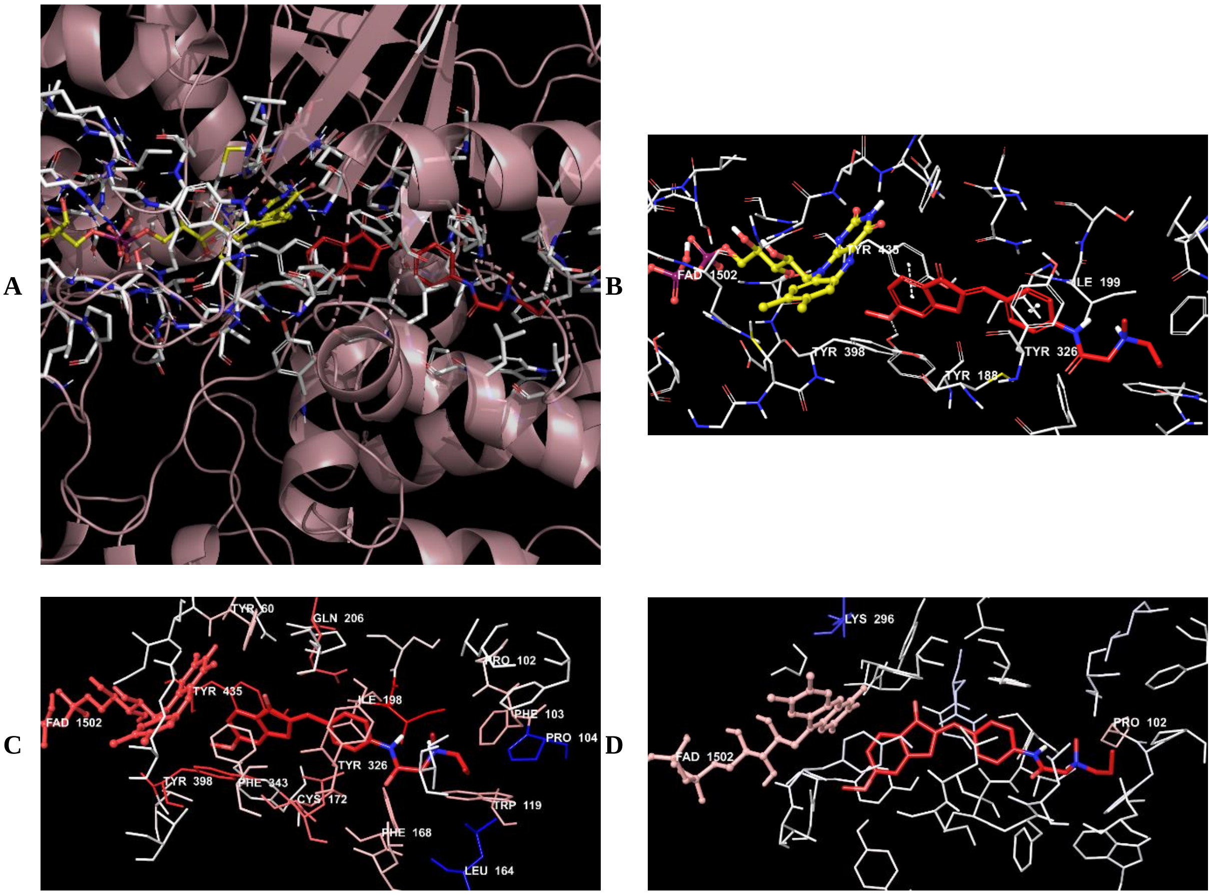

Figure S4. The three-dimensional placement pose (**A**) and three-dimensional interacting mode (**B**) of compound **D28** in the active site of MAO-B. The inhibitor and important residues in the active site of enzyme are presented by tube model and colored with red and white, respectively. The van der Waals (**C**) and electrostatic (**D**) interactions of this compound with active region of MAO-B. The active ligand has a lot of favorable van der Waals (red and pink) and electrostatic (blue, red, and pink) interactions (MAO-B PDB Code: 2V5Z).

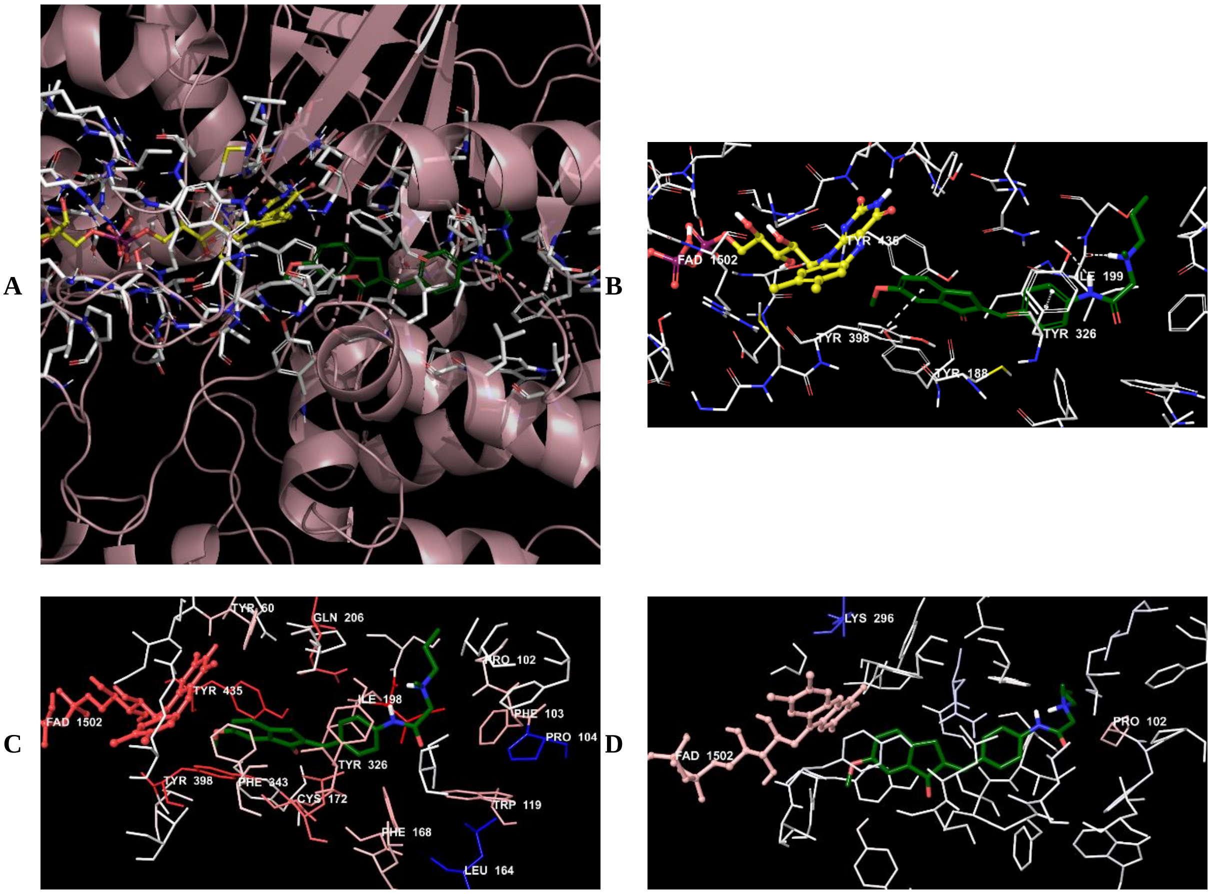

Figure S5. The three-dimensional placement pose (**A**) and three-dimensional interacting mode (**B**) of compound **D29** in the active site of MAO-B. The inhibitor and important residues in the active site of enzyme are presented by tube model and colored with dark green and white, respectively. The van der Waals (**C**) and electrostatic (**D**) interactions of this compound with active region of MAO-B. The active ligand has a lot of favorable van der Waals (red and pink) and electrostatic (blue, red, and pink) interactions (MAO-B PDB Code: 2V5Z).

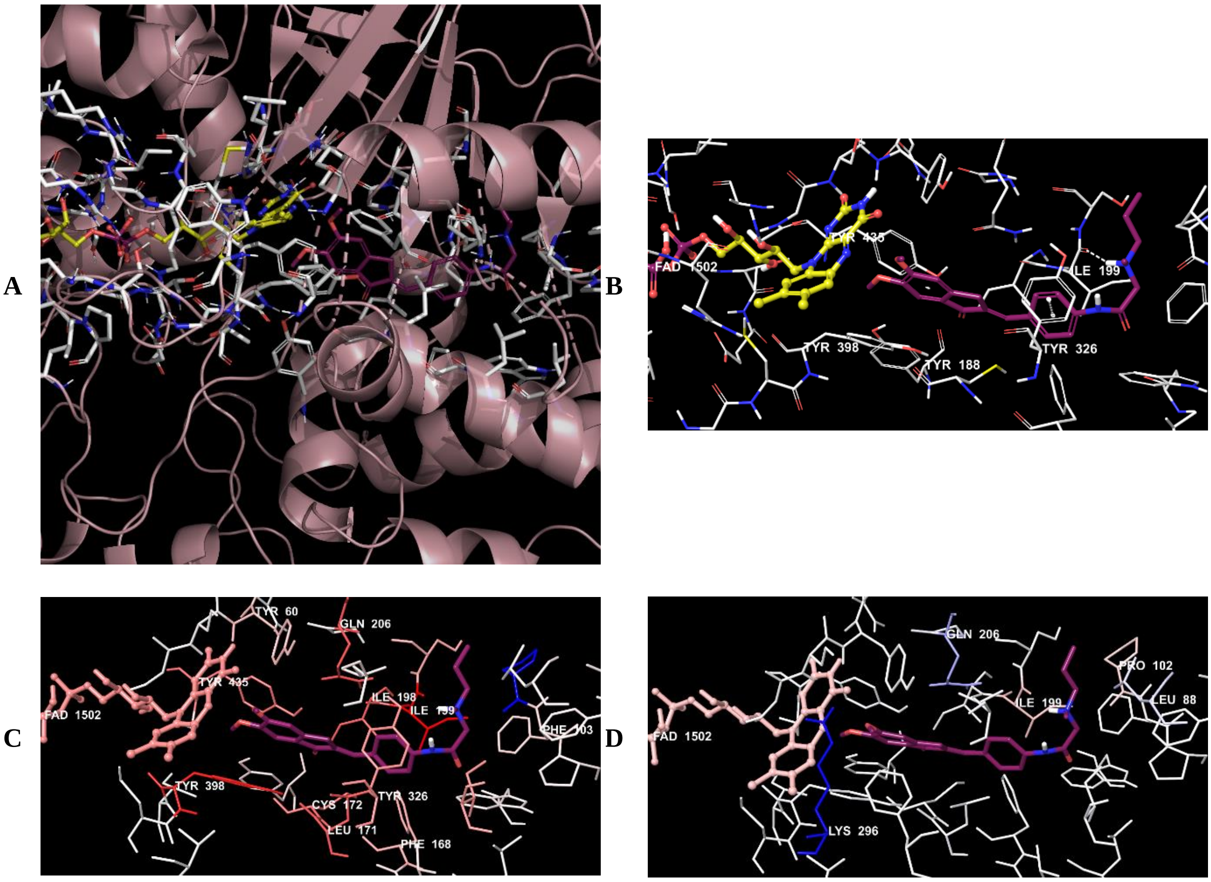

Figure S6. The three-dimensional placement pose (**A**) and three-dimensional interacting mode (**B**) of compound **D30** in the active site of MAO-B. The inhibitor and important residues in the active site of enzyme are presented by tube model and colored with maroon and white, respectively. The van der Waals (**C**) and electrostatic (**D**) interactions of this compound with active region of MAO-B. The active ligand has a lot of favorable van der Waals (red and pink) and electrostatic (blue, red, and pink) interactions (MAO-B PDB Code: 2V5Z).

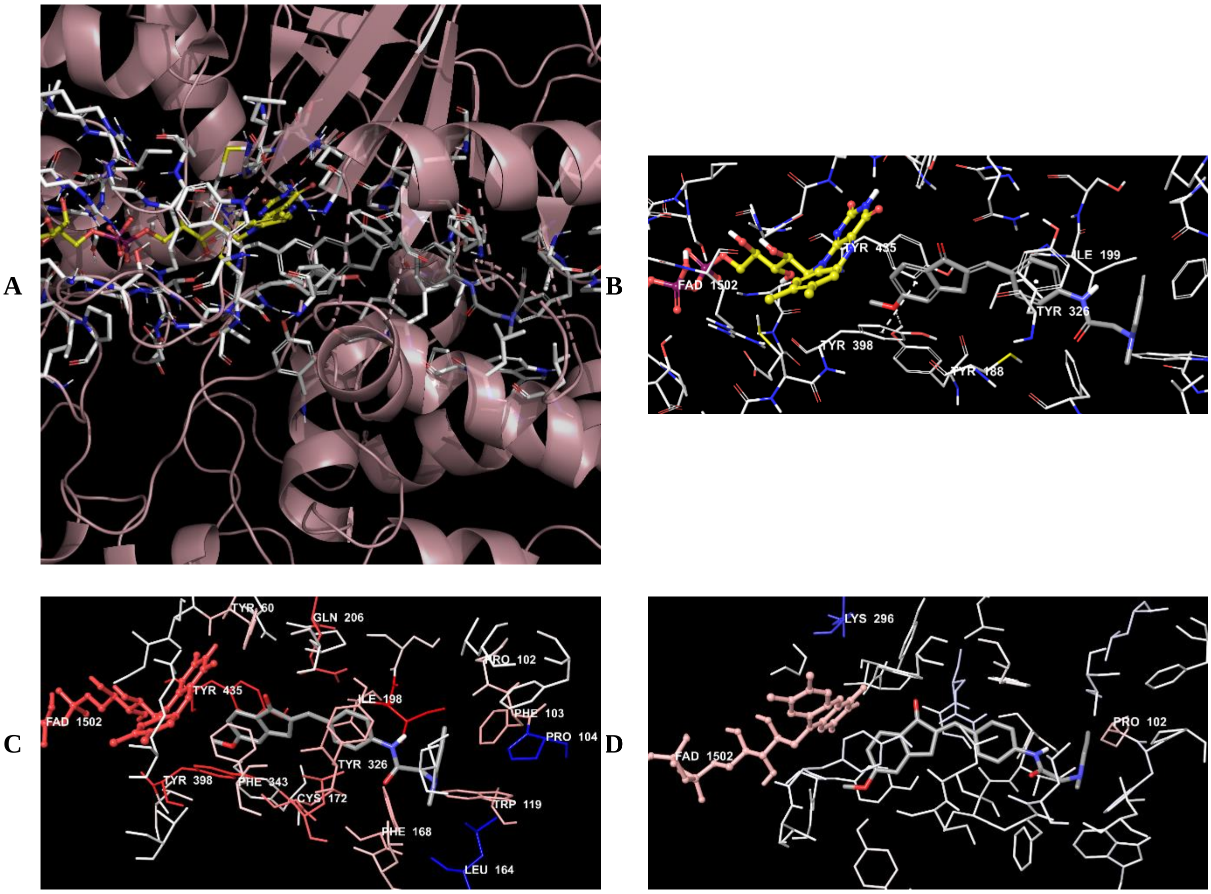

Figure S7. The three-dimensional placement pose (**A**) and three-dimensional interacting mode (**B**) of compound **D31** in the active site of MAO-B. The inhibitor and important residues in the active site of enzyme are presented by tube model and colored with grey and white, respectively. The van der Waals (**C**) and electrostatic (**D**) interactions of this compound with active region of MAO-B. The active ligand has a lot of favorable van der Waals (red and pink) and electrostatic (blue, red, and pink) interactions (MAO-B PDB Code: 2V5Z).

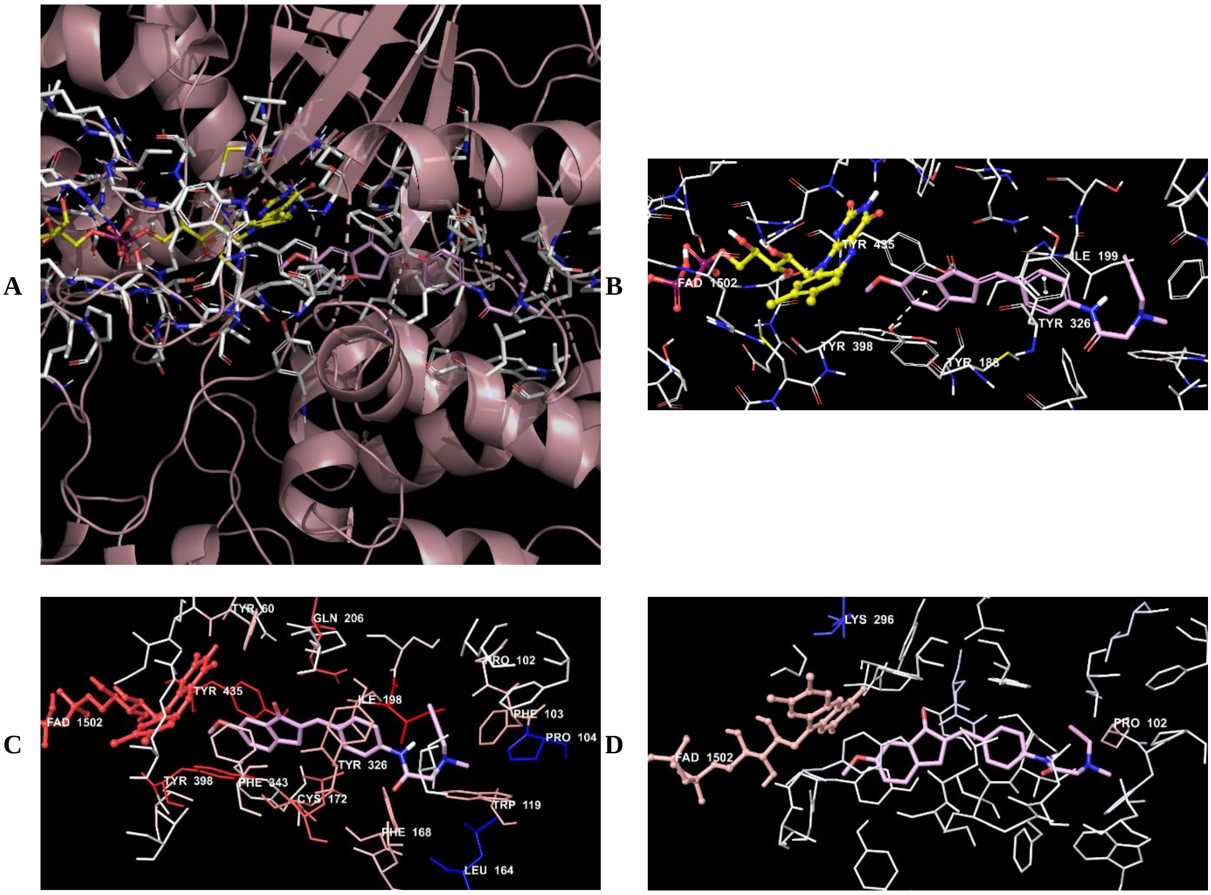

Figure S8. The three-dimensional placement pose (**A**) and three-dimensional interacting mode (**B**) of compound **D32** in the active site of MAO-B. The inhibitor and important residues in the active site of enzyme are presented by tube model and colored with purple and white, respectively. The van der Waals (**C**) and electrostatic (**D**) interactions of this compound with active region of MAO-B. The active ligand has a lot of favorable van der Waals (red and pink) and electrostatic (blue, red, and pink) interactions (MAO-B PDB Code: 2V5Z).

4. Analytical results of the compounds

*5-Methoxy-2-(4-acetamidobenzylidene)-2,3-dihydro-1H-inden-1-one (**A1**)*

Yellow powder. M.P.: 171.8 °C. Yield: 75%.

IR (ATR) ν_{max} (cm^{-1}): 3334 (N-H), 1674 (indanone C=O), 1595 (amide C=O), 1585-1527 (C=C), 1247 (C-N), 1089 (C-O), 823 (1,4-disubstituted benzene).

^{1}H-NMR (300 MHz, DMSO-d_6) δ (ppm): 2.08 (3H, s, CH_3), 3.89 (3H, s, OCH_3), 4.05 (2H, s, CH_2), 7.03 (1H, dd, J_1=8.49 Hz, J_2=2.25 Hz, methoxy-1-oxo-indenylidene CH), 7.18 (1H, d, *J*=2.07 Hz, methoxy-1-oxo-indenylidene CH), 7.39 (1H, s, C=CH), 7.71 (5H, s, disubstituted benzene CH, methoxy-1-oxo-indenylidene CH), 10.23 (1H, s, NH).

^{13}C-NMR (75 MHz, DMSO-d_6) δ (ppm): 24.6, 32.4, 56.2, 110.6, 115.7, 119.4, 125.8, 130.0, 131.1, 131.8, 131.9, 134.3, 141.0, 153.2, 165.2, 169.1, 192.0.

HRMS (ESI) (m/z) $[M+H]^+$: $C_{19}H_{17}NO_3$ calculated: 308.1281, found: 308.1275.

DOPNALAB

	Item	Value
	Acquired Date&Time	22.08.2019 10:37:21
	Acquired by	System Administrator
	Filename	C:\Users\dopnalab\Desktop\NURPELİN\DOKTORA TEZ\A11.ispd
	Spectrum name	A11
	Sample name	A1
	Sample ID	
	Option	
	Comment	
	No. of Scans	50
	Resolution	4 [cm-1]
	Apodization	Happ-Genzel

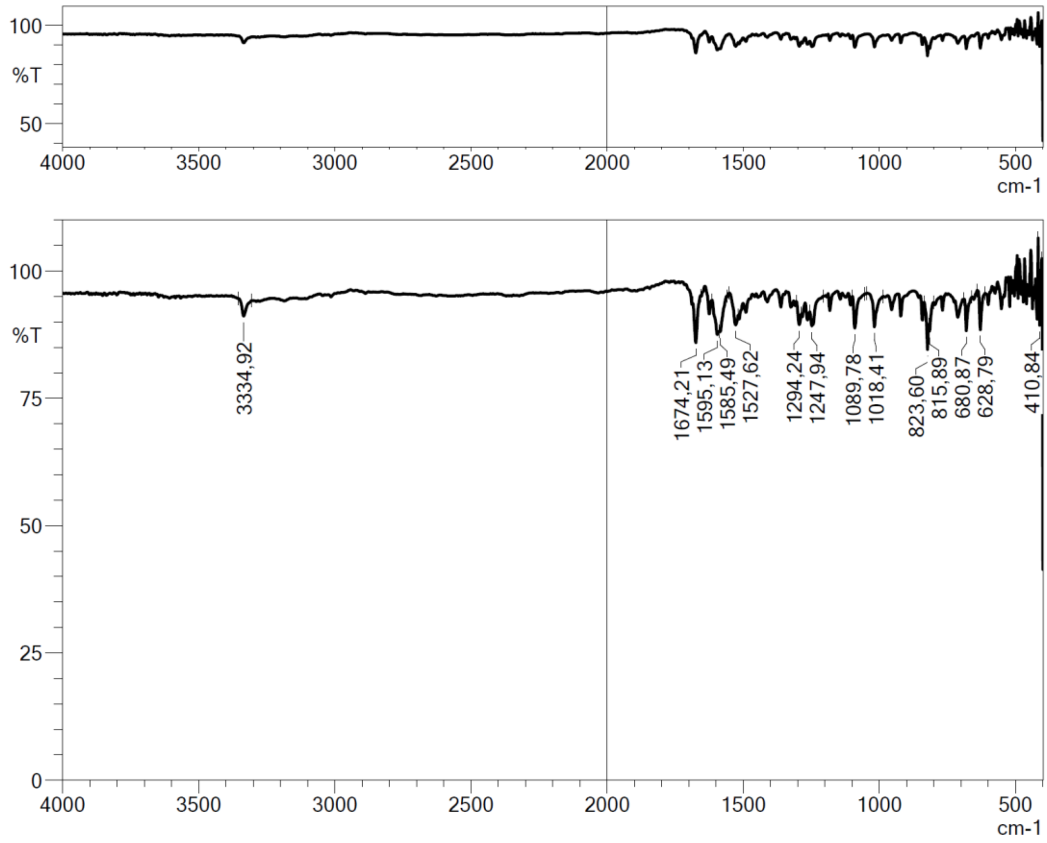

Figure S9. Compound **A1** IR report.

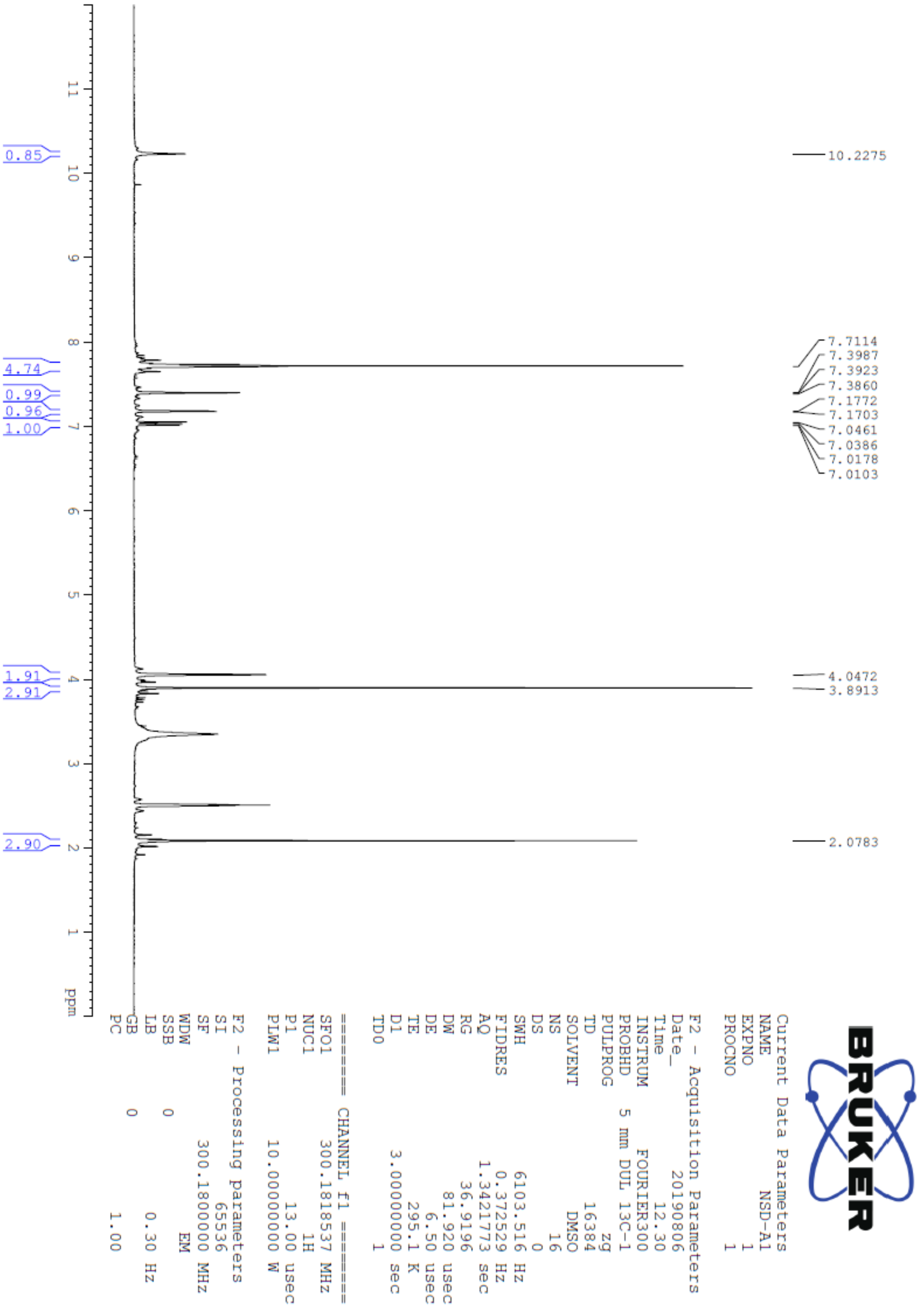

Figure S10. Compound **A1** ^{1}H-NMR spectrum.

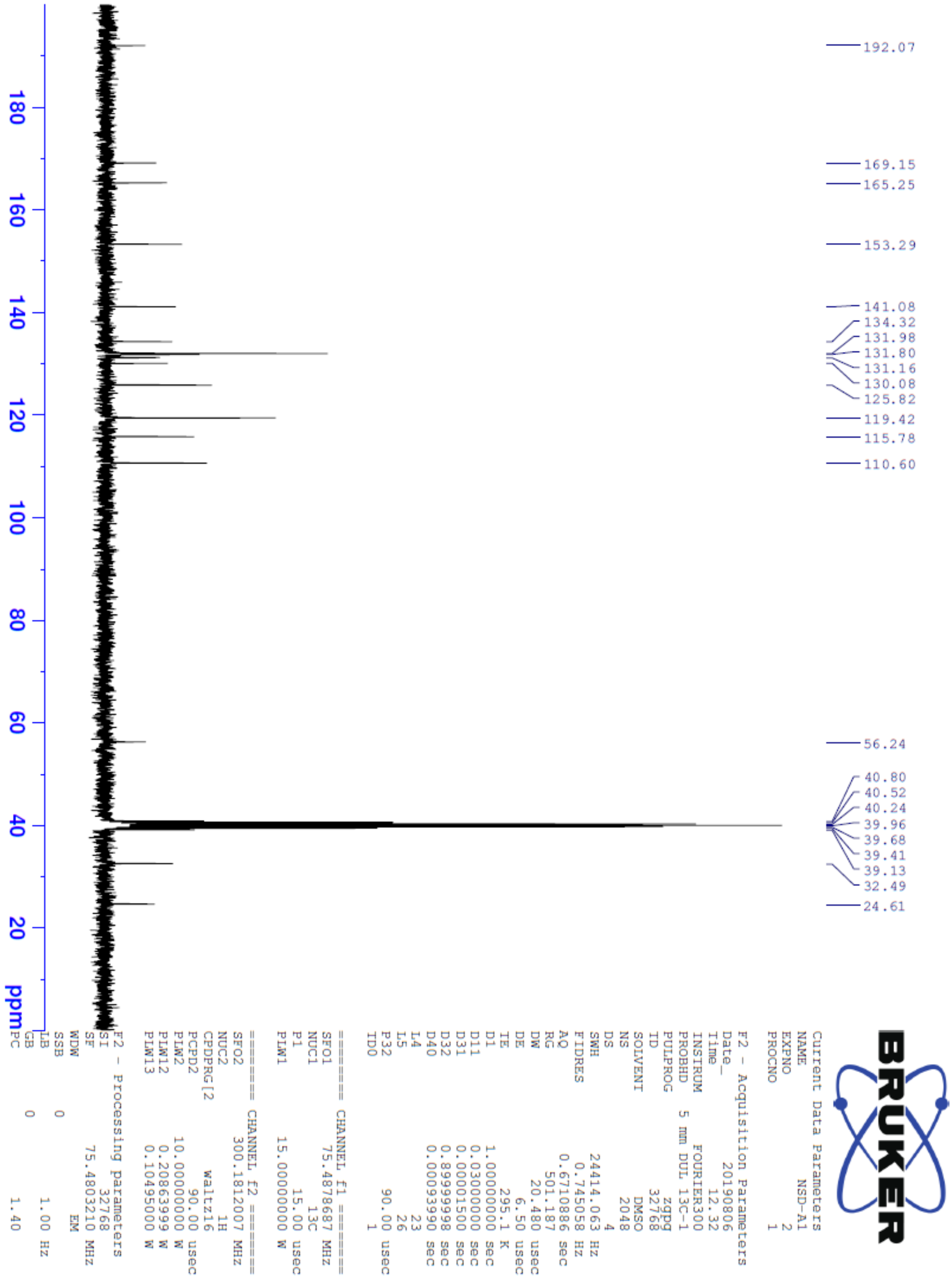

Figure S11. Compound **A1** ^{13}C-NMR spectrum.

Data File: C:\LabSolutions\Data\Analiz\bns\A-1_2.lcd

Elmt	Val.	Min	Max	Elmt	Val.	Min	Max	Elmt	Val.	Min	Max	Elmt	Val.	Min	Max	Use Adduct
H	1	0	50	O	2	3	8	S	2	0	1	Ru	2	0	0	H
C	4	0	50	F	1	0	0	Cl	1	0	1	Pd	2	0	0	
N	3	0	4	P	3	0	0	Br	1	0	0	I	3	0	0	

Error Margin (ppm):	10	DBE Range:	10.0 - 30.0	Electron Ions:	both
HC Ratio:	unlimited	Apply N Rule:	yes	Use MSn Info:	yes
Max Isotopes:	3	Isotope RI (%):	1.00	Isotope Res:	9000
MSn Iso RI (%):	10.00	MSn Logic Mode:	AND	Max Results:	500

Event#: 1 MS(E+) Ret. Time : 2.573 Scan# : 387

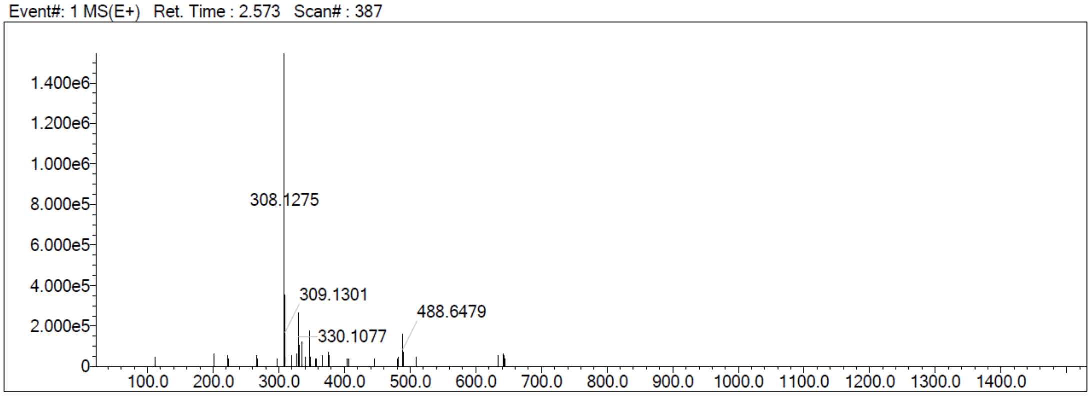

Measured region for 308.1275 m/z

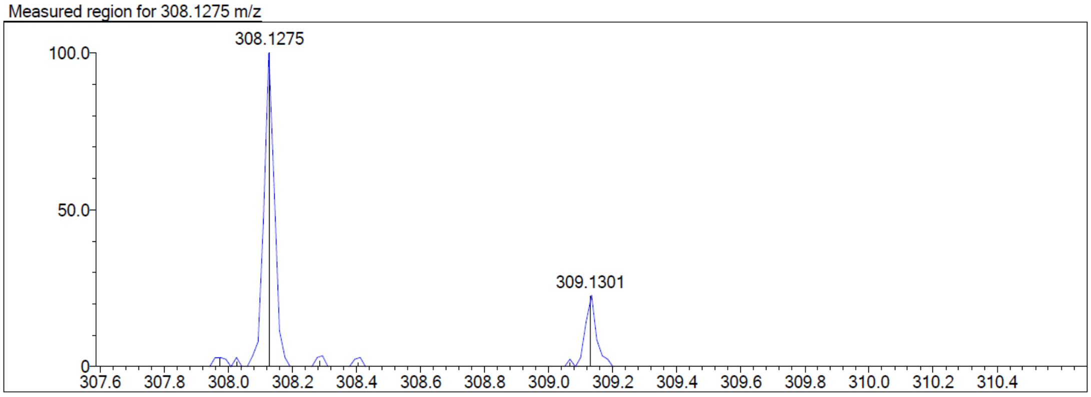

C19 H17 N O3 [M+H]+ : Predicted region for 308.1281 m/z

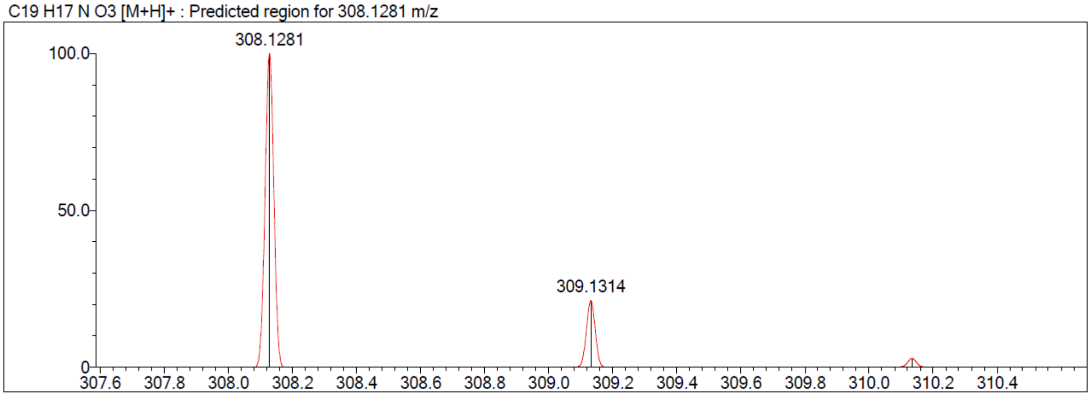

Rank	Score	Formula (M)	Ion	Meas. m/z	Pred. m/z	Df. (mDa)	Df. (ppm)	Iso	DBE
1	71.73	C19 H17 N O3	[M+H]+	308.1275	308.1281	-0.6	-1.95	73.47	12.0

Figure S12. Compound **A1** HRMS report.

*6-Methoxy-2-(4-acetamidobenzylidene)-2,3-dihydro-1H-inden-1-one (**A2**)*

Yellow powder. M.P.: 185.9 °C. Yield: 79%.

IR (ATR) ν_{max} (cm^{-1}): 3325 (N-H), 1695 (indanone C=O), 1678 (amide C=O), 1587-1512 (C=C), 1168 (C-N), 1103 (C-O), 827 (1,4-disubstituted benzene).

^{1}H-NMR (300 MHz, DMSO-*d_6*) δ (ppm): 2.08 (3H, s, CH_3), 3.83 (3H, s, OCH_3), 3.99 (2H, s, CH_2), 7.23 (1H, d, *J*=2.46 Hz, methoxy-1-oxo-indenylidene CH), 7.27 (1H, dd, J_1=8.28 Hz, J_2=2.55 Hz, methoxy-1-oxo-indenylidene CH), 7.48 (1H, s, C=CH), 7.56 (1H, d, *J*=8.34 Hz, methoxy-1-oxo-indenylidene CH), 7.72 (4H, s, disubstituted benzene CH), 10.21 (1H, s, NH).

^{13}C-NMR (75 MHz, DMSO-*d_6*) δ (ppm): 24.5, 31.7, 55.9, 55.9, 105.9, 119.4, 123.6, 127.9, 129.9, 132.2, 133.0, 134.4, 139.1, 141.3, 142.9, 159.5, 169.1, 193.5.

HRMS (ESI) (m/z) $[M+H]^+$: $C_{19}H_{17}NO_3$ calculated: 308.1281, found: 308.1269.

DOPNALAB

	Item	Value
	Acquired Date&Time	22.08.2019 10:43:15
	Acquired by	System Administrator
	Filename	C:\Users\dopnalab\Desktop\NURPELİN\DOKTORA TEZ\A21.ispd
	Spectrum name	A21
	Sample name	A2
	Sample ID	
	Option	
	Comment	
	No. of Scans	50
	Resolution	4 [cm-1]
	Apodization	Happ-Genzel

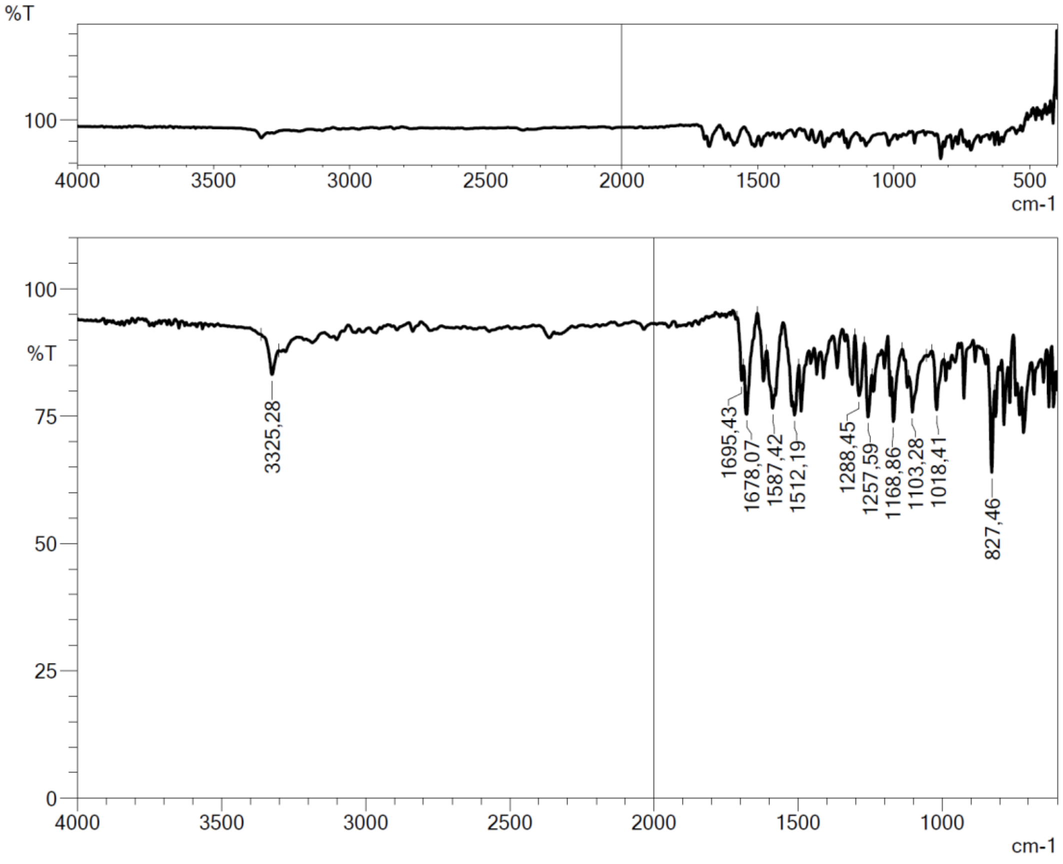

Figure S13. Compound **A2** IR report.

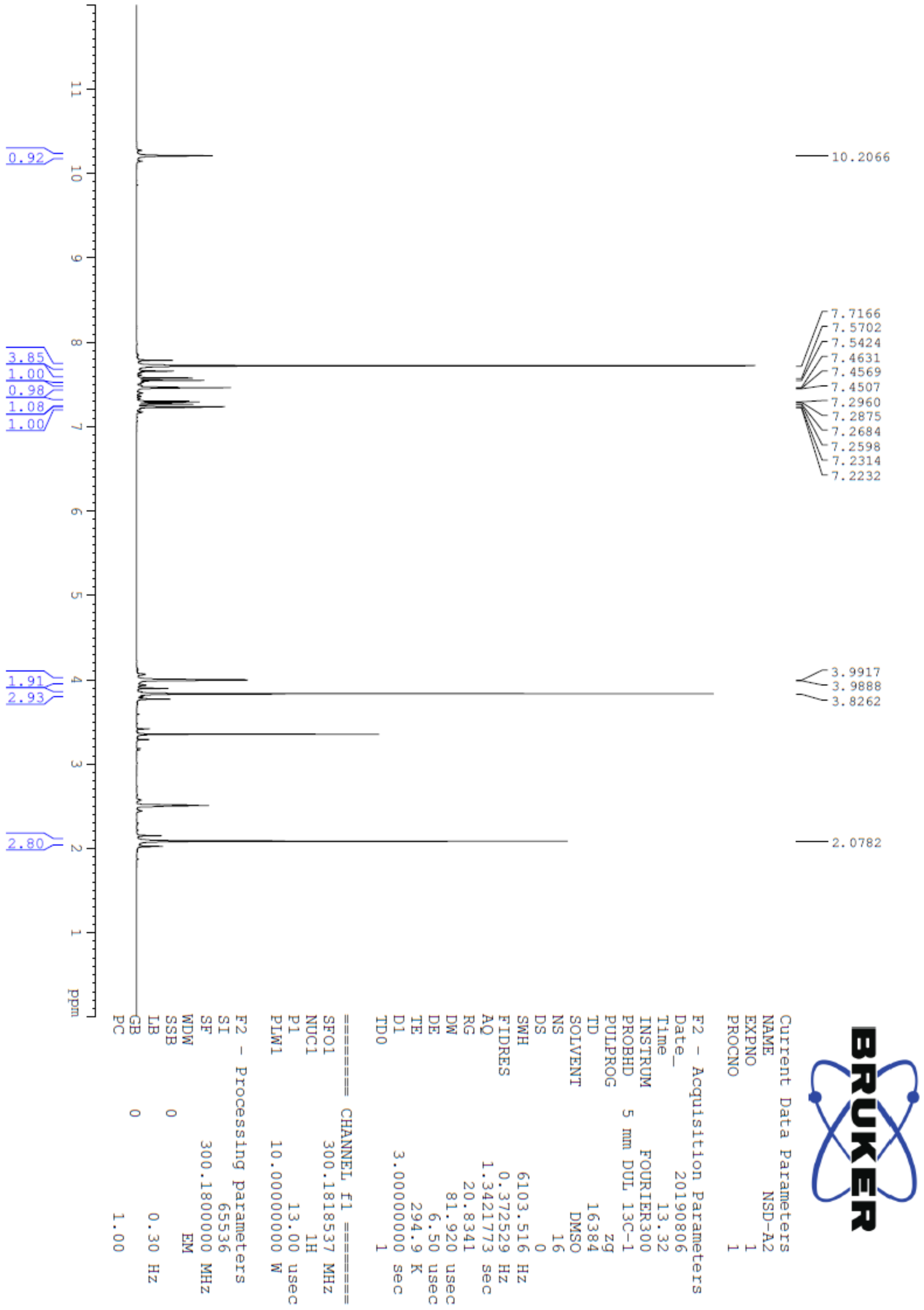

Figure S14. Compound **A2** ^{1}H-NMR spectrum.

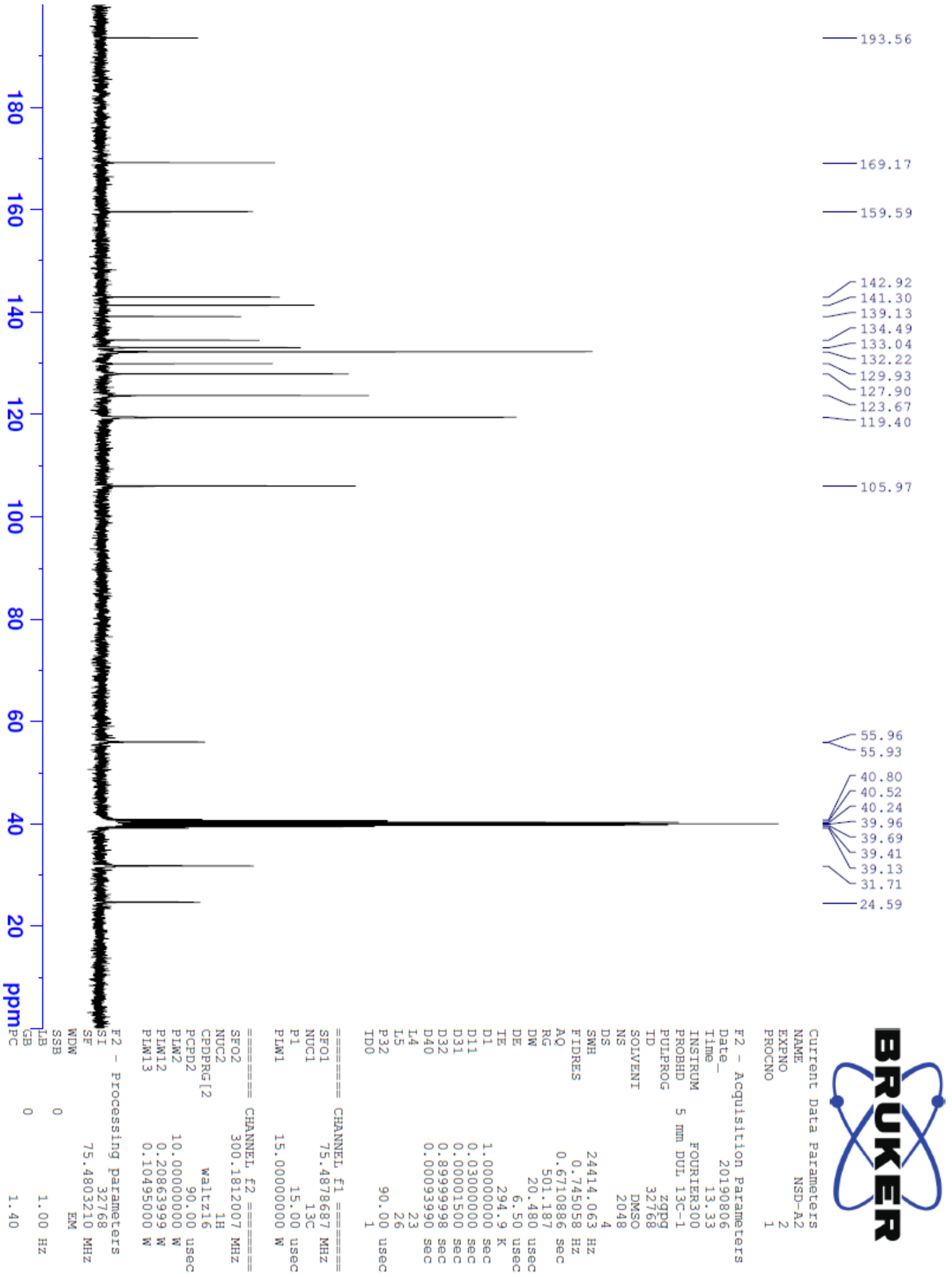

Figure S15. Compound **A2** ^{13}C-NMR spectrum.

Data File: C:\LabSolutions\Data\Analiz\bns\A-2_3.lcd

Elmt	Val.	Min	Max	Elmt	Val.	Min	Max	Elmt	Val.	Min	Max	Elmt	Val.	Min	Max	Use Adduct
H	1	0	50	O	2	2	8	S	2	0	1	Ru	2	0	0	H
C	4	0	50	F	1	0	0	Cl	1	0	1	Pd	2	0	0	
N	3	0	4	P	3	0	0	Br	1	0	0	I	3	0	0	

Error Margin (ppm): 10
HC Ratio: unlimited
Max Isotopes: 3
MSn Iso RI (%): 10.00

DBE Range: 10.0 - 30.0
Apply N Rule: yes
Isotope RI (%): 1.00
MSn Logic Mode: AND

Electron Ions: both
Use MSn Info: yes
Isotope Res: 9000
Max Results: 500

Event#: 1 MS(E+) Ret. Time : 7.173 Scan# : 1077

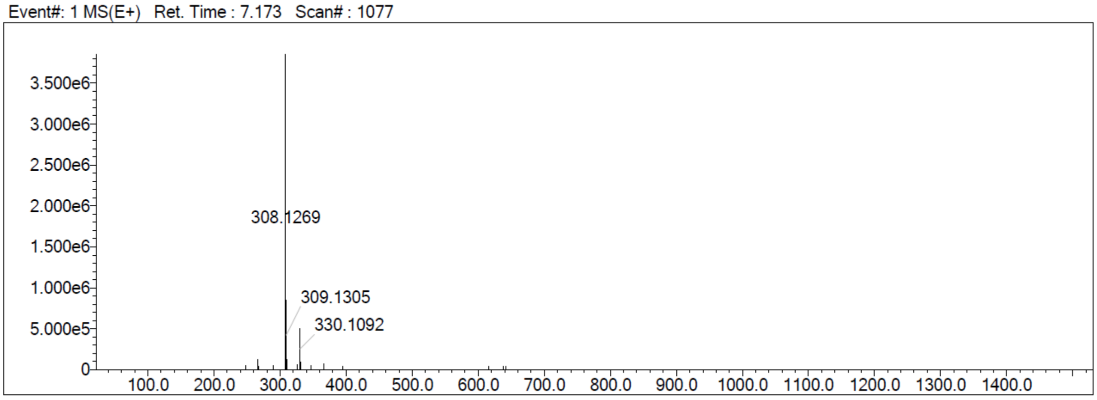

Measured region for 308.1269 m/z

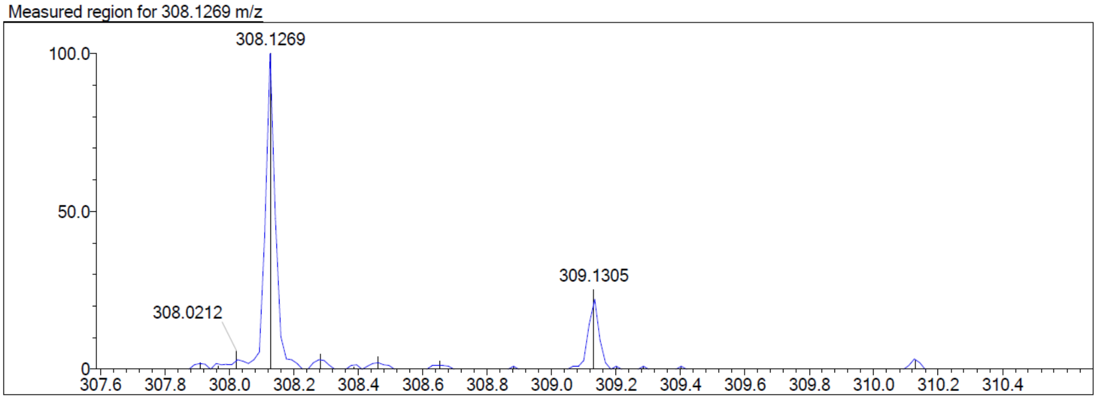

C19 H17 N O3 [M+H]+ : Predicted region for 308.1281 m/z

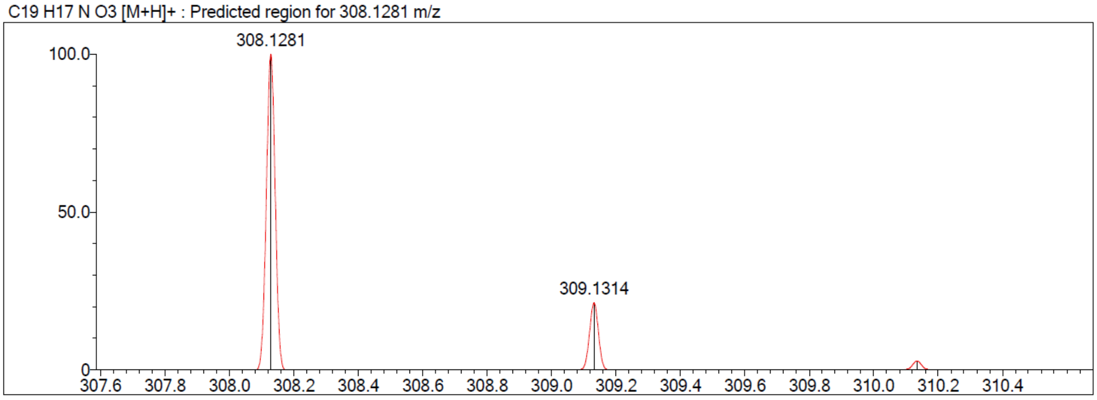

Rank	Score	Formula (M)	Ion	Meas. m/z	Pred. m/z	Df. (mDa)	Df. (ppm)	Iso	DBE
1	63.07	C19 H17 N O3	[M+H]+	308.1269	308.1281	-1.2	-3.89	67.98	12.0

Figure S16. Compound **A2** HRMS report.

*5,6-Dimethoxy-2-(4-acetamidobenzylidene)-2,3-dihydro-1H-inden-1-one (**A3**)*

Yellow powder. M.P.: 120.4 °C. Yield: 72%.

IR (ATR) ν_{max} (cm^{-1}): 3300 (N-H), 1689 (indanone C=O), 1672 (amide C=O), 1589-1523 (C=C), 1132 (C-N), 1095 (C-O), 819 (1,4-disubstituted benzene).

^{1}H-NMR (300 MHz, DMSO-*d_6*) δ (ppm): 2.08 (3H, s, CH_3), 3.83 (3H, s, OCH_3), 3.90 (3H, s, OCH_3), 3.97 (2H, s, CH_2), 7.18 (1H, s, methoxy-1-oxo-indenylidene CH), 7.21 (1H, s, methoxy-1-oxo-indenylidene CH), 7.36 (1H, s, C=CH), 7.70 (4H, s, disubstituted benzene CH), 10.19 (1H, s, NH).

^{13}C-NMR (75 MHz, DMSO-*d_6*) δ (ppm): 24.5, 32.0, 56.0, 56.1, 56.4, 56.4, 104.9, 108.4, 119.4, 130.1, 130.5, 131.3, 131.8, 134.5, 140.9, 145.3, 149.7, 155.5, 169.1, 192.3.

HRMS (ESI) (m/z) $[M+H]^+$: $C_{20}H_{19}NO_4$ calculated: 338.1387, found: 338.1385.

DOPNALAB

	Item	Value
	Acquired Date&Time	22.08.2019 10:47:34
	Acquired by	System Administrator
	Filename	C:\Users\dopnalab\Desktop\NURPELİN\DOKTORA TEZ\A31.ispd
	Spectrum name	A31
	Sample name	A3
	Sample ID	
	Option	
	Comment	
	No. of Scans	50
	Resolution	4 [cm-1]
	Apodization	Happ-Genzel

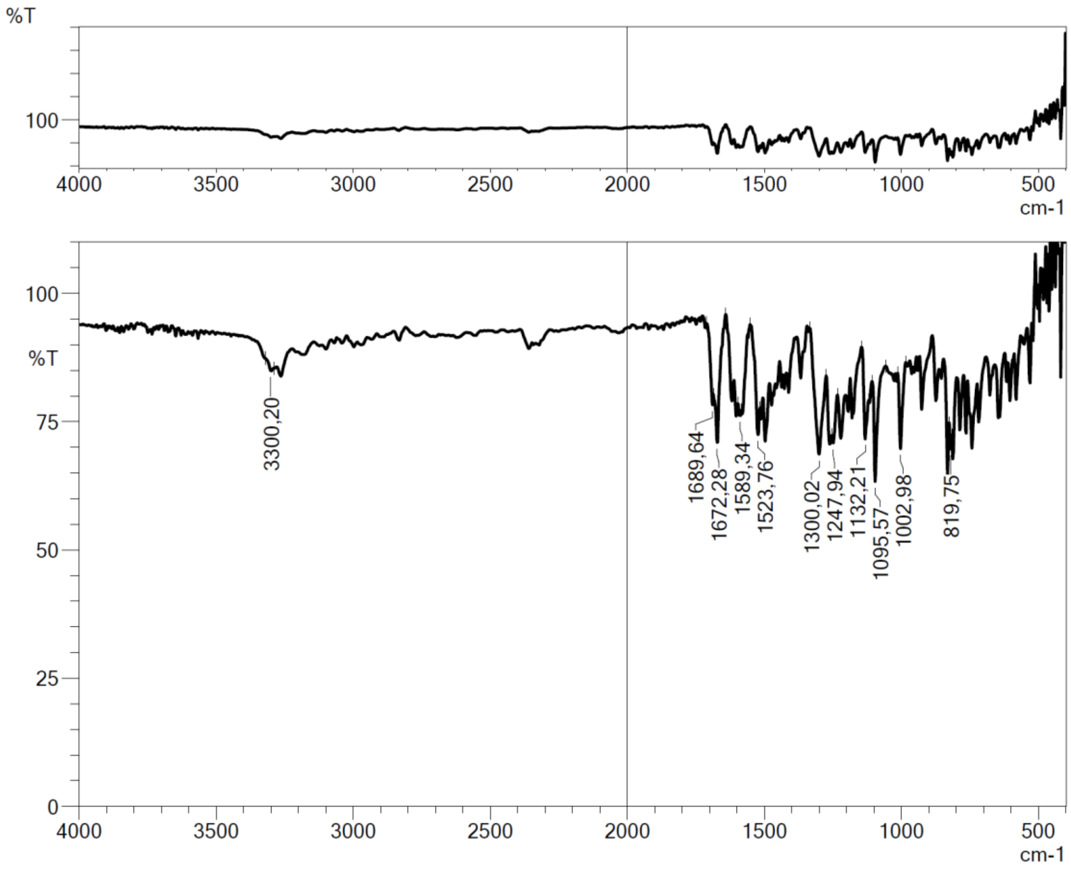

Figure S17. Compound **A3** IR report.

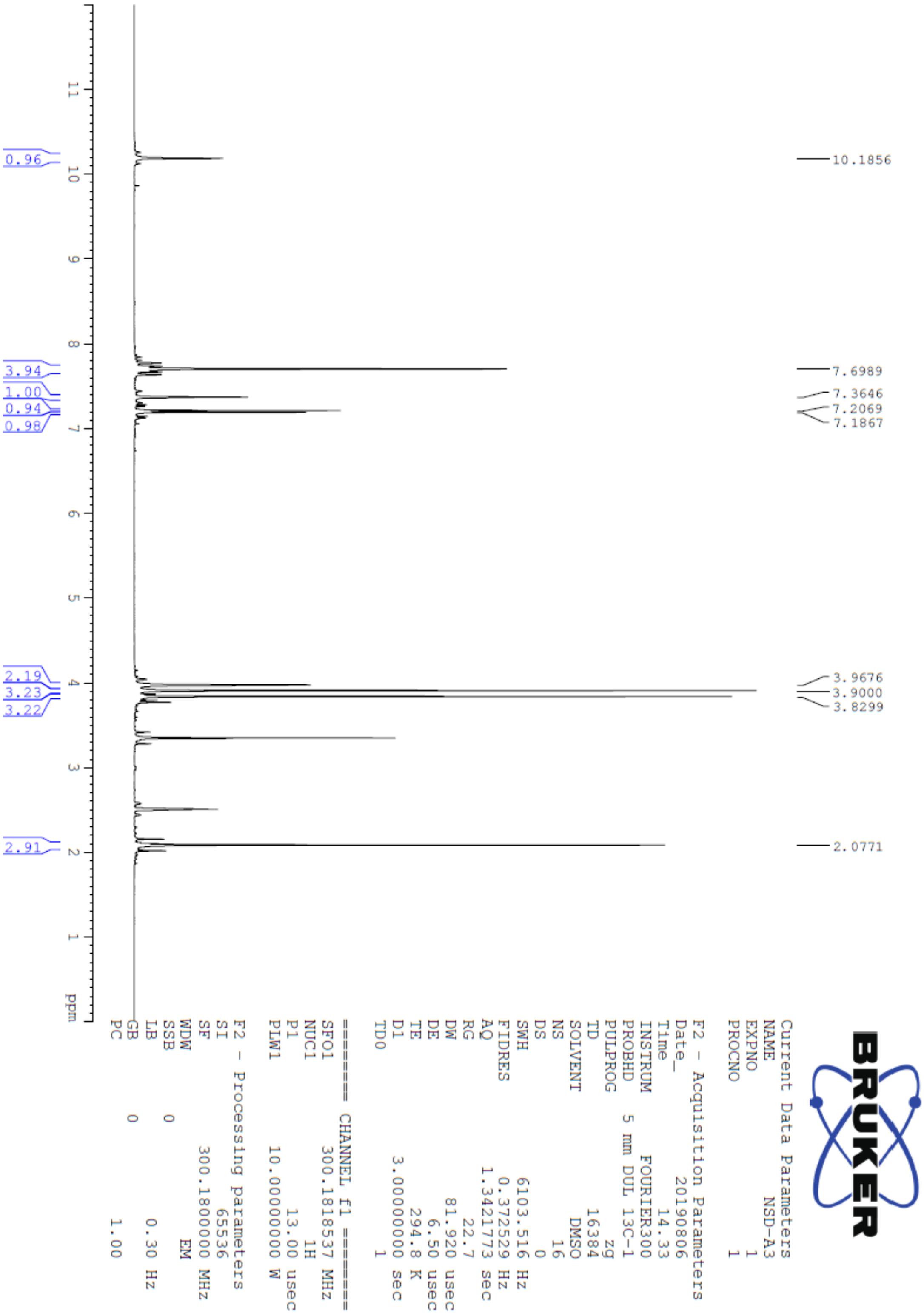

Figure S18. Compound **A3** ^{1}H-NMR spectrum.

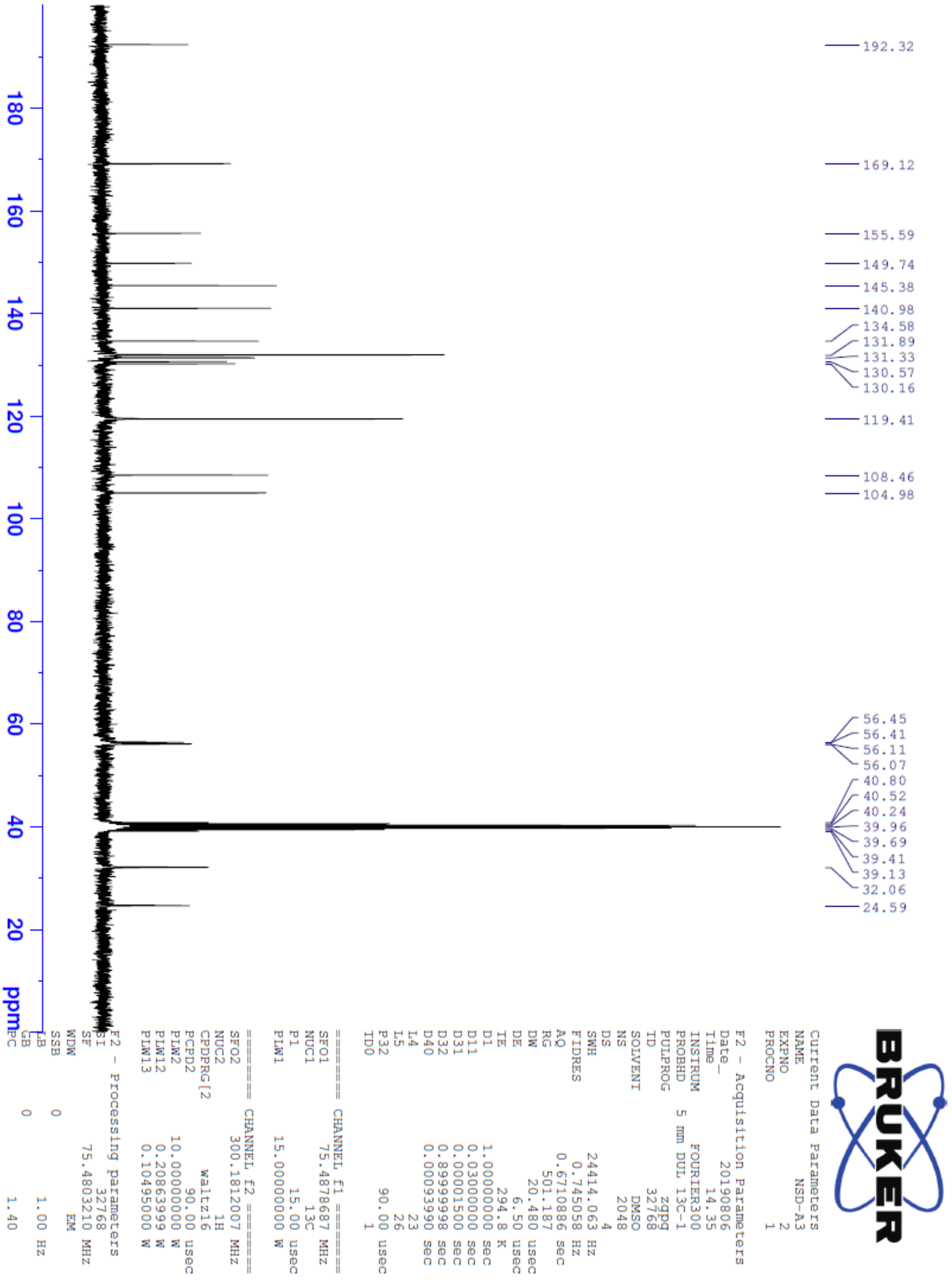

Figure S19. Compound **A3** ^{13}C-NMR spectrum.

Data File: C:\LabSolutions\Data\Analiz\bns\A-3_4.lcd

Elmt	Val.	Min	Max	Elmt	Val.	Min	Max	Elmt	Val.	Min	Max	Elmt	Val.	Min	Max	Use Adduct
H	1	0	50	O	2	2	8	S	2	0	1	Ru	2	0	0	H
C	4	0	50	F	1	0	0	Cl	1	0	1	Pd	2	0	0	
N	3	0	4	P	3	0	0	Br	1	0	0	I	3	0	0	

Error Margin (ppm):	10	DBE Range:	10.0 - 30.0	Electron Ions:	both
HC Ratio:	unlimited	Apply N Rule:	yes	Use MSn Info:	yes
Max Isotopes:	3	Isotope RI (%):	1.00	Isotope Res:	9000
MSn Iso RI (%):	10.00	MSn Logic Mode:	AND	Max Results:	500

Event#: 1 MS(E+) Ret. Time : 6.373 -> 6.600 Scan# : 957 -> 991

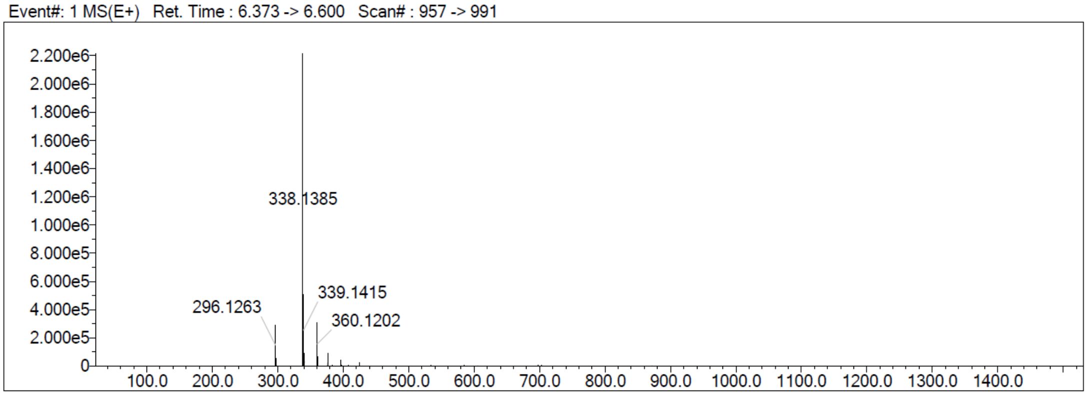

Measured region for 338.1385 m/z

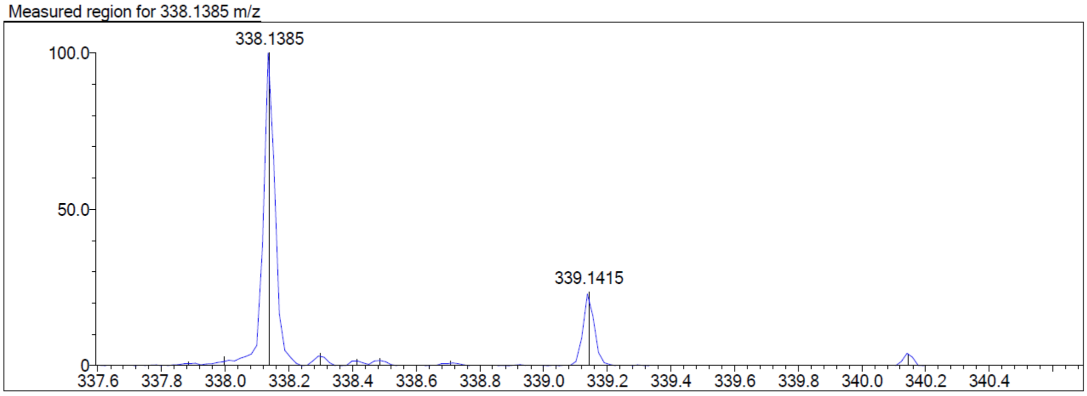

C20 H19 N O4 [M+H]+ : Predicted region for 338.1387 m/z

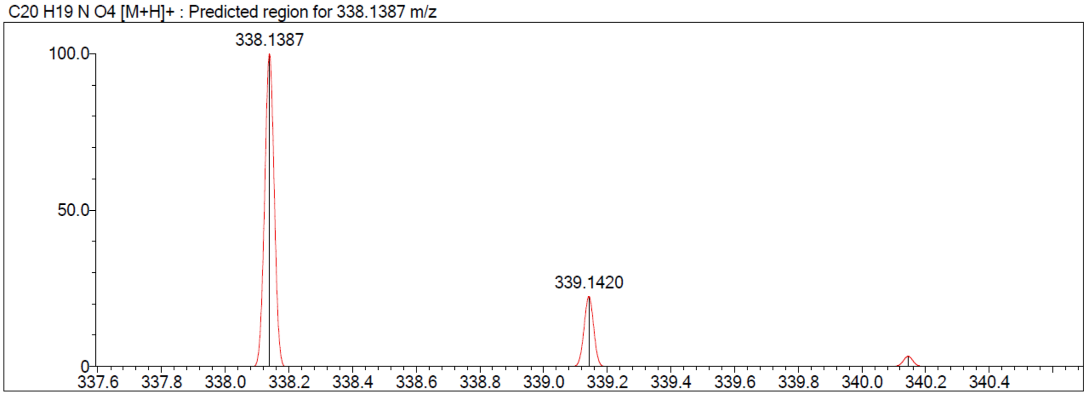

Rank	Score	Formula (M)	Ion	Meas. m/z	Pred. m/z	Df. (mDa)	Df. (ppm)	Iso	DBE
1	82.64	C20 H19 N O4	[M+H]+	338.1385	338.1387	-0.2	-0.59	82.64	12.0

Figure S20. Compound **A3** HRMS report.

*5-Methoxy-2-(4-aminobenzylidene)-2,3-dihydro-1H-inden-1-one (**B1**)*

Yellow powder. M.P.: 188.9 °C. Yield: 80%.

IR (ATR) ν_{max} (cm^{-1}): 3423 and 3321 (NH_2), 1664 (indanone C=O), 1571-1512 (C=C), 1180 (C-N), 1091 (C-O), 829 (1,4-disubstituted benzene).

^{1}H-NMR (300 MHz, DMSO-d_6) δ (ppm): 3.88 (3H, s, OCH_3), 3.95 (2H, s, CH_2), 5.87 (2H, s, NH_2), 6.64 (2H, d, J=8.55 Hz, disubstituted benzene CH), 7.00 (1H, dd, J_1=8.46 Hz, J_2=2.28 Hz, methoxy-1-oxo-indenylidene CH), 7.16 (1H, d, J=2.01 Hz, methoxy-1-oxo-indenylidene CH), 7.31 (1H, s, C=CH), 7.46 (2H, d, J=8.61 Hz, disubstituted benzene CH), 7.67 (1H, d, J=8.46 Hz, methoxy-1-oxo-indenylidene CH).

^{13}C-NMR (75 MHz, DMSO-d_6) δ (ppm): 32.7, 56.1, 56.1, 110.6, 114.2, 115.4, 122.7, 125.3, 129.7, 131.7, 133.2, 133.5, 151.4, 152.7, 164.7, 191.9.

HRMS (ESI) (m/z) $[M+H]^+$: $C_{17}H_{15}NO_2$ calculated: 266.1176, found: 266.1166.

DOPNALAB

	Item	Value
	Acquired Date&Time	22.08.2019 10:52:37
	Acquired by	System Administrator
	Filename	C:\Users\dopnalab\Desktop\NURPELİN\DOKTORA TEZ\B11.ispd
	Spectrum name	B11
	Sample name	B1
	Sample ID	
	Option	
	Comment	
	No. of Scans	50
	Resolution	4 [cm-1]
	Apodization	Happ-Genzel

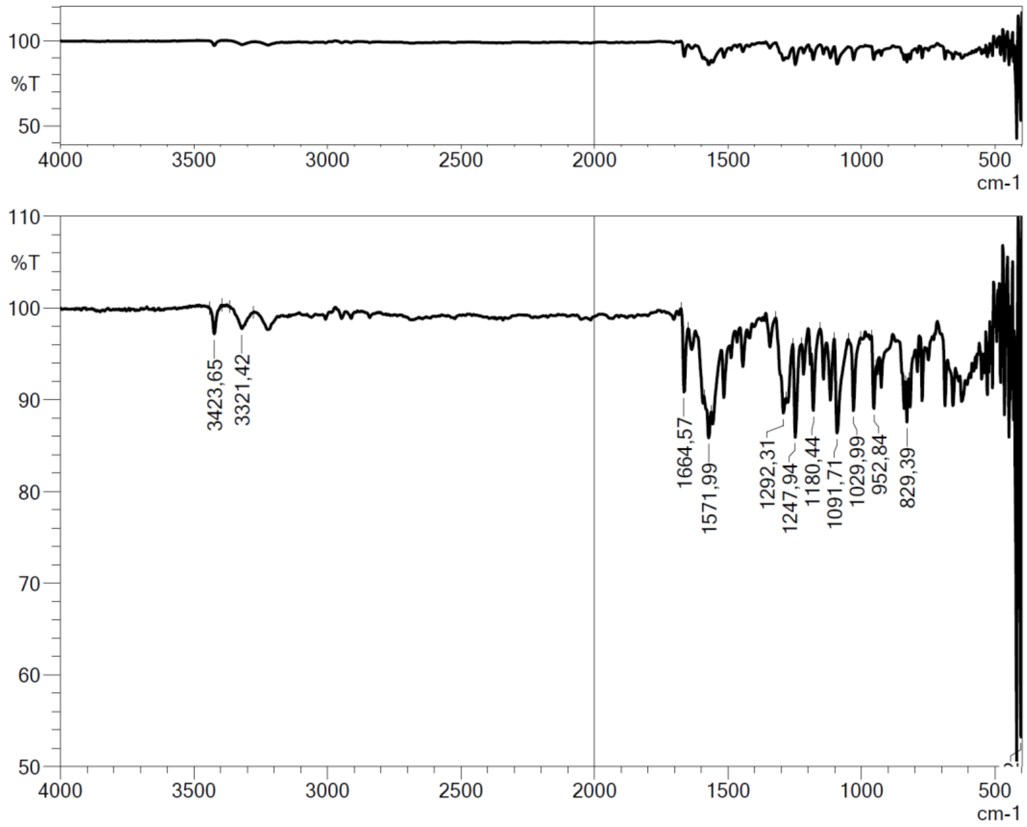

Figure S21. Compound **B1** IR report.

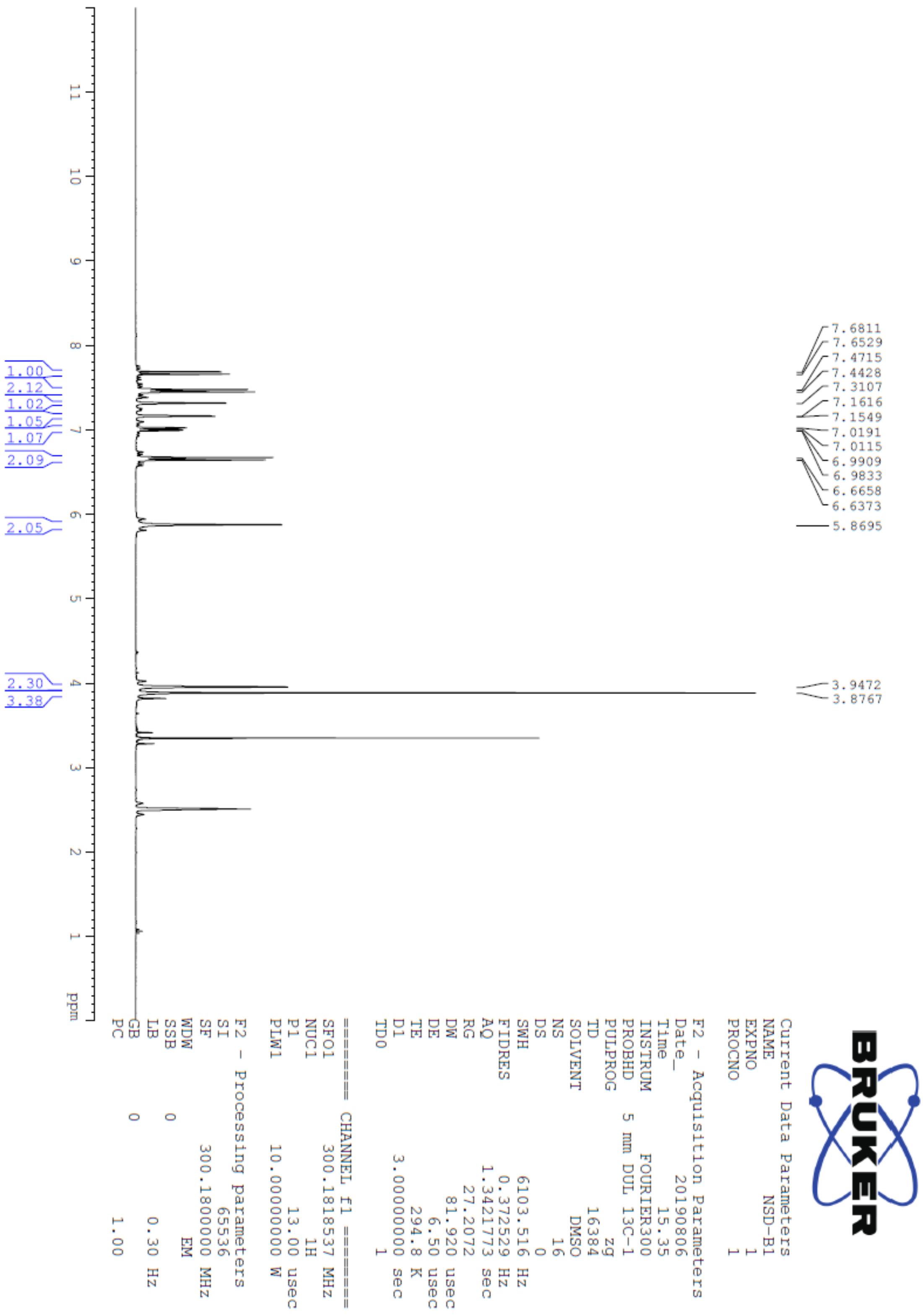

Figure S22. Compound **B1** ^{1}H-NMR spectrum.

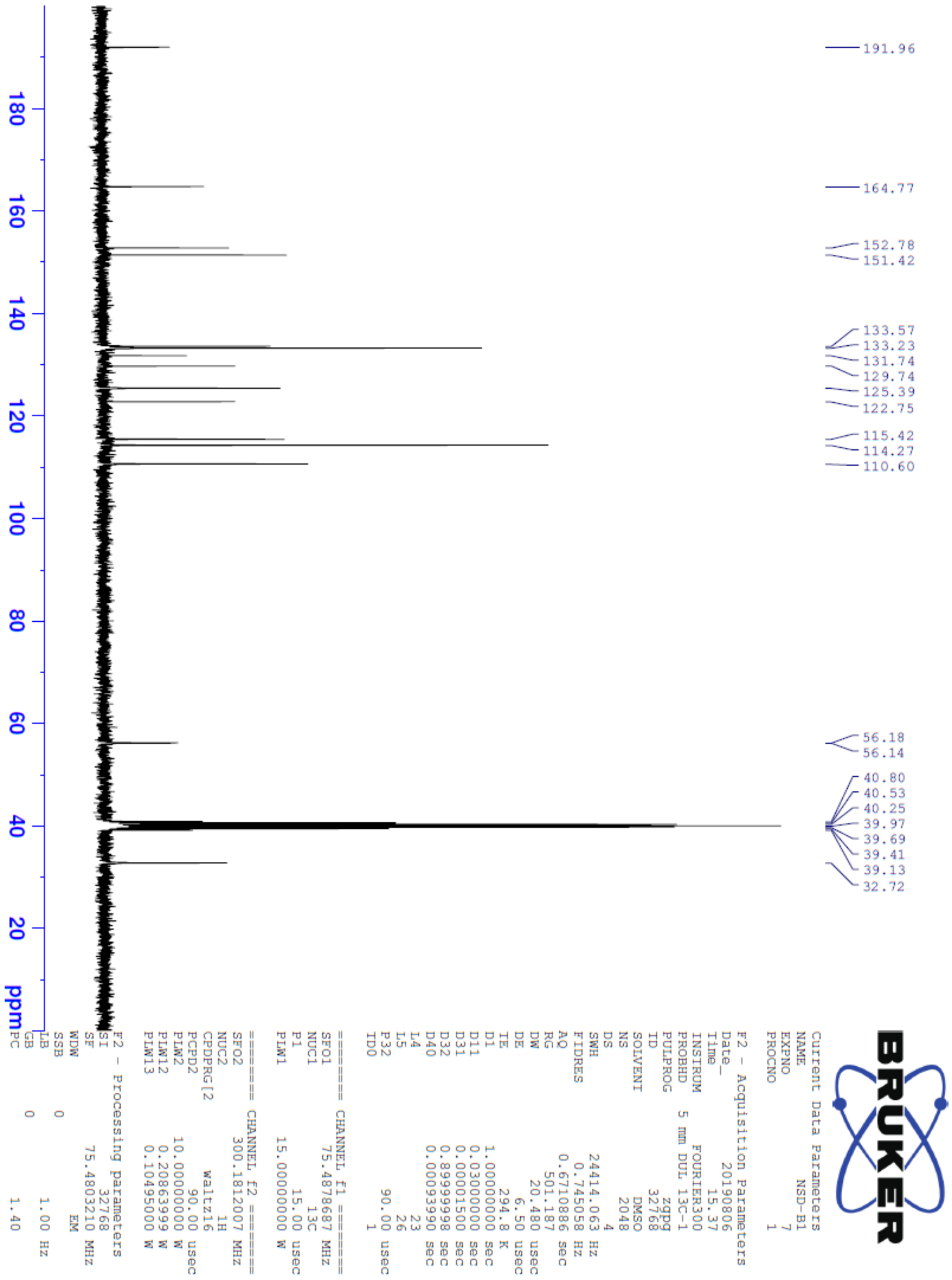

Figure S23. Compound **B1** ^{13}C-NMR spectrum.

Data File: C:\LabSolutions\Data\Analiz\bns\A-1_H_2.lcd

Elmt	Val.	Min	Max	Elmt	Val.	Min	Max	Elmt	Val.	Min	Max	Elmt	Val.	Min	Max	Use Adduct
H	1	0	50	O	2	2	8	S	2	0	1	Ru	2	0	0	H
C	4	0	50	F	1	0	0	Cl	1	0	1	Pd	2	0	0	
N	3	0	4	P	3	0	0	Br	1	0	0	I	3	0	0	

Error Margin (ppm):	10	DBE Range:	10.0 - 30.0	Electron Ions:	both
HC Ratio:	unlimited	Apply N Rule:	yes	Use MSn Info:	yes
Max Isotopes:	3	Isotope RI (%):	1.00	Isotope Res:	9000
MSn Iso RI (%):	10.00	MSn Logic Mode:	AND	Max Results:	500

Event#: 1 MS(E+) Ret. Time : 6.827 -> 7.040 Scan# : 1025 -> 1057

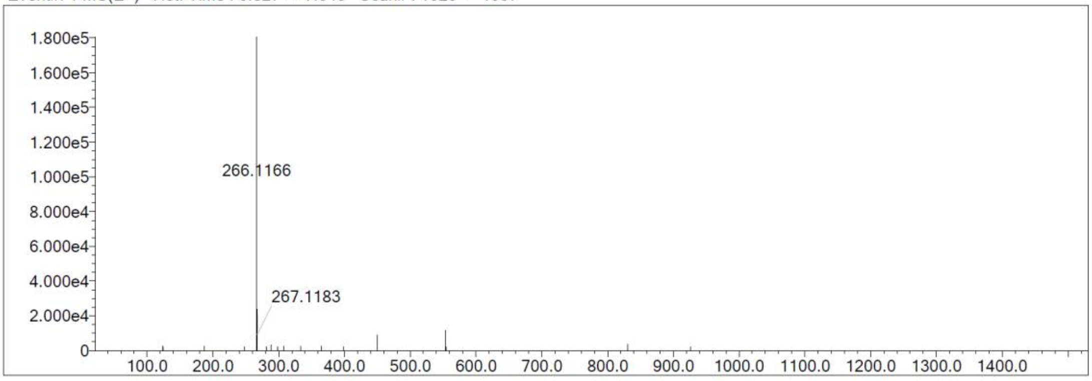

Measured region for 266.1166 m/z

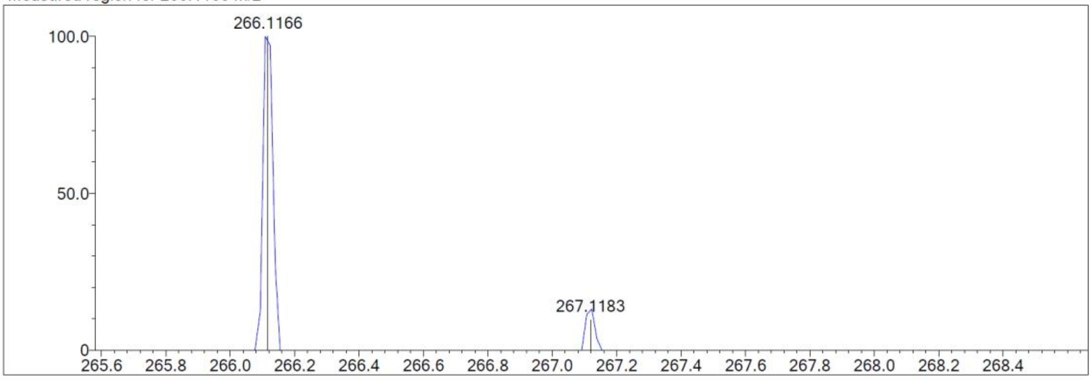

C17 H15 N O2 [M+H]+ : Predicted region for 266.1176 m/z

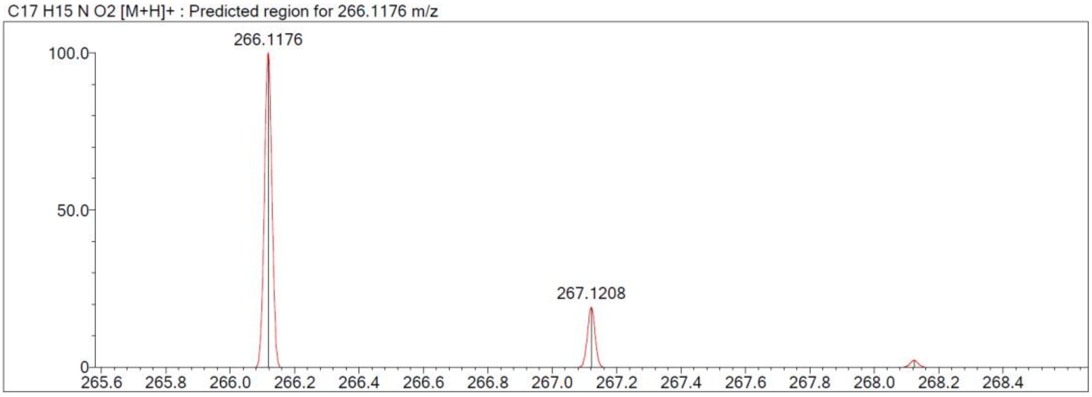

Rank	Score	Formula (M)	Ion	Meas. m/z	Pred. m/z	Df. (mDa)	Df. (ppm)	Iso	DBE
1	64.94	C17 H15 N O2	[M+H]+	266.1166	266.1176	-1.0	-3.76	69.76	11.0

Figure S24. Compound **B1** HRMS report.

*6-Methoxy-2-(4-aminobenzylidene)-2,3-dihydro-1H-inden-1-one (**B2**)*

Yellow powder. M.P.: 205.7 °C. Yield: 75%.

IR (ATR) ν_{max} (cm^{-1}): 3425 and 3344 (NH_2), 1664 (indanone C=O), 1560-1433 (C=C), 1182 (C-N), 1116 (C-O), 817 (1,4-disubstituted benzene).

^{1}H-NMR (300 MHz, DMSO-*d_6*) δ (ppm): 3.82 (3H, s, OCH_3), 3.90 (2H, s, CH_2), 5.94 (2H, s, NH_2), 6.66 (2H, d, *J*=8.58 Hz, disubstituted benzene CH), 7.21 (1H, dd, J_1=7.50 Hz, J_2=2.50 Hz, methoxy-1-oxo-indenylidene CH), 7.25 (1H, d, *J*=2.58 Hz, methoxy-1-oxo-indenylidene CH), 7.39 (1H, s, C=CH), 7.48 (2H, d, *J*=8.67 Hz, disubstituted benzene CH), 7.54 (1H, d, *J*=8.28 Hz, methoxy-1-oxo-indenylidene CH).

^{13}C-NMR (75 MHz, DMSO-*d_6*) δ (ppm): 31.9, 55.8, 55.9, 105.8, 114.2, 122.6, 122.8, 127.7, 129.9, 133.5, 134.8, 139.8, 142.4, 151.7, 159.5, 193.2.

HRMS (ESI) (m/z) $[M+H]^+$: $C_{17}H_{15}NO_2$ calculated: 266.1176, found: 266.1178.

DOPNALAB

	Item	Value
	Acquired Date&Time	22.08.2019 10:56:41
	Acquired by	System Administrator
	Filename	C:\Users\dopnalab\Desktop\NURPELİN\DOKTORA TEZ\B21.ispd
	Spectrum name	B21
	Sample name	B2
	Sample ID	
	Option	
	Comment	
	No. of Scans	50
	Resolution	4 [cm-1]
	Apodization	Happ-Genzel

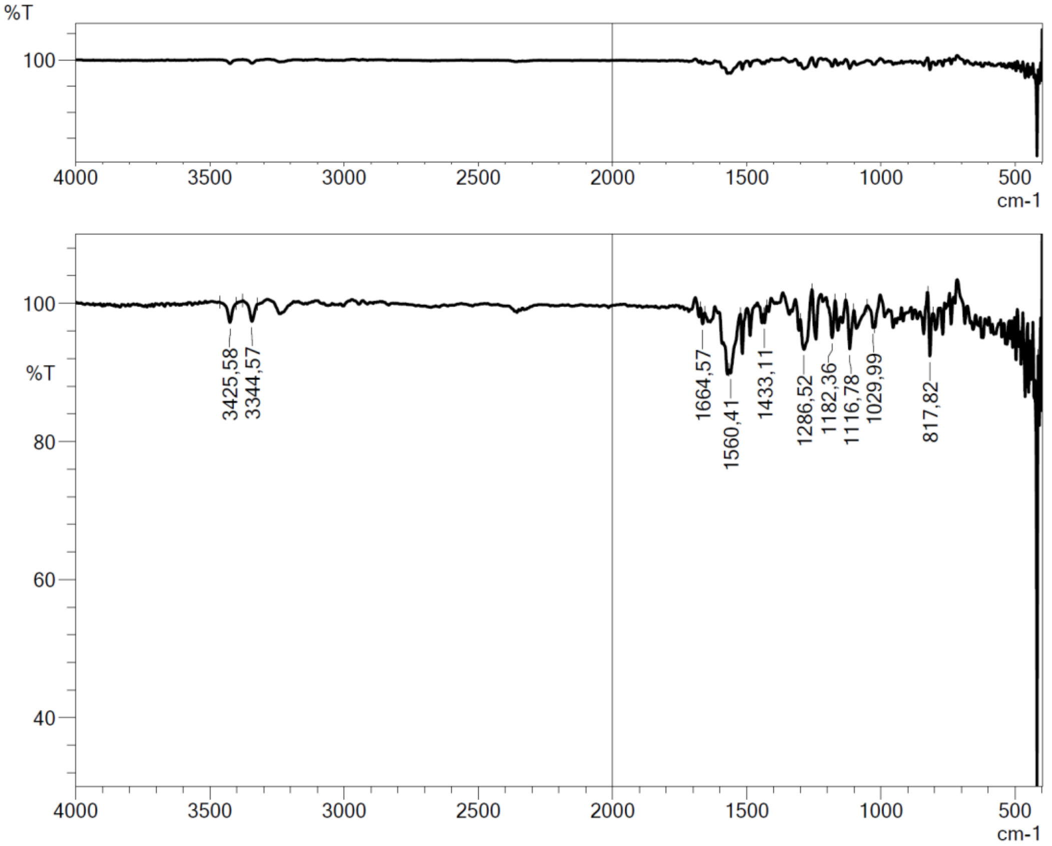

Figure S25. Compound **B2** IR report.

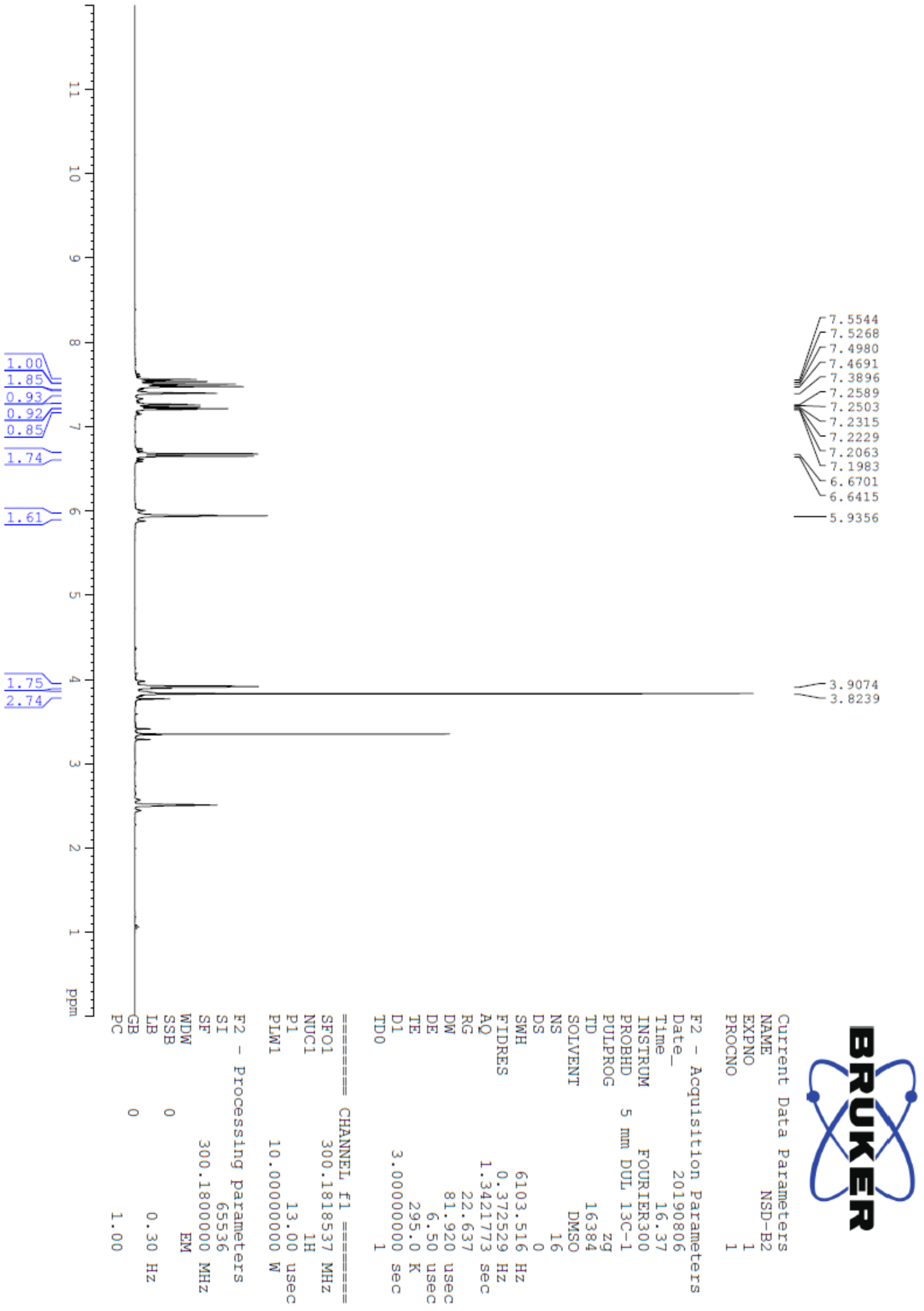

Figure S26. Compound **B2** ^{1}H-NMR spectrum.

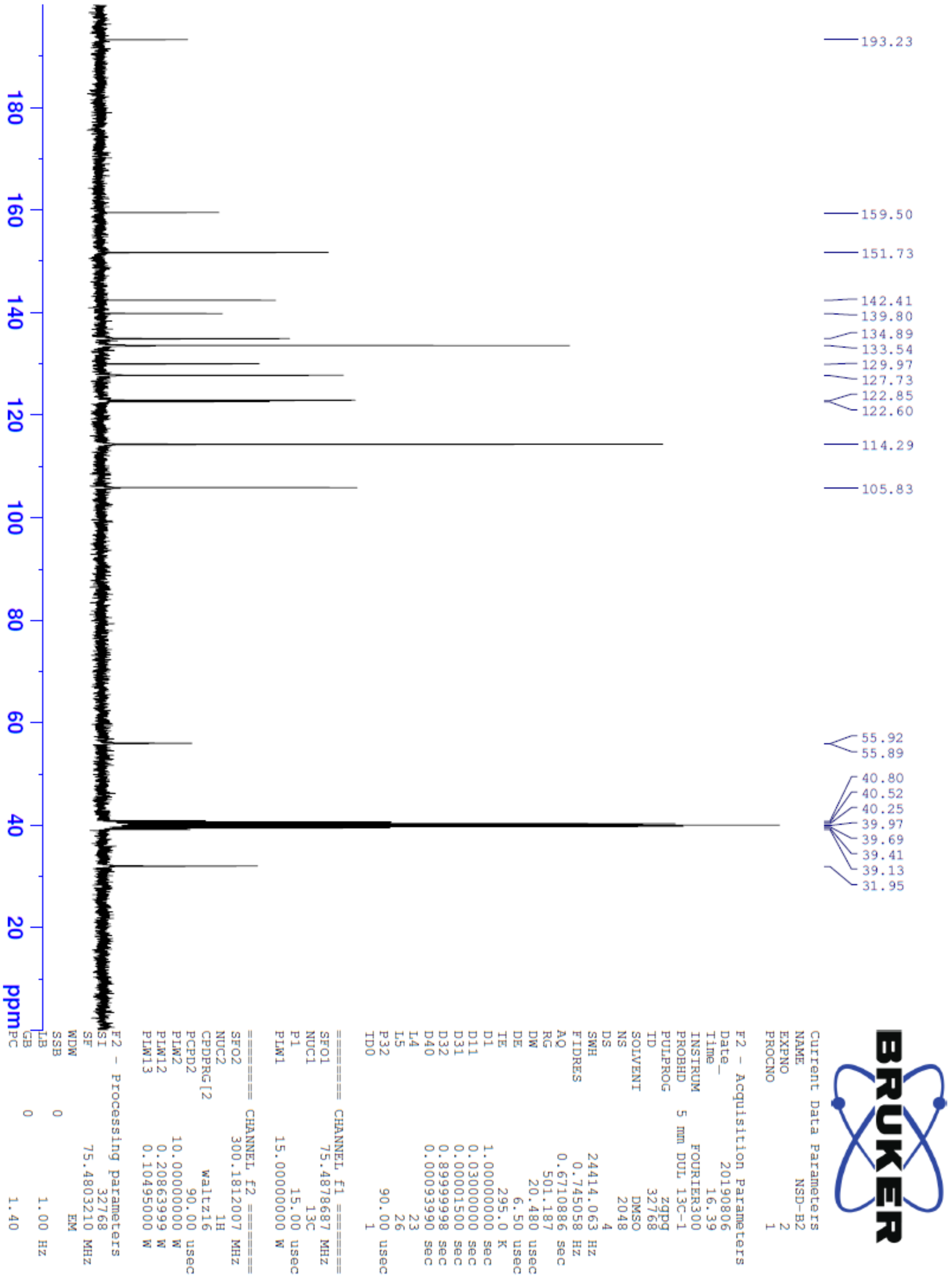

Figure S27. Compound **B2** ^{13}C-NMR spectrum.

Formula Predictor Report - A-2_H_2.lcd Page 1 of 1

Data File: C:\LabSolutions\Data\Analiz\bns\A-2_H_2.lcd

Elmt	Val.	Min	Max	Elmt	Val.	Min	Max	Elmt	Val.	Min	Max	Elmt	Val.	Min	Max	Use Adduct
H	1	0	50	O	2	2	8	S	2	0	1	Ru	2	0	0	H
C	4	0	50	F	1	0	0	Cl	1	0	1	Pd	2	0	0	
N	3	0	4	P	3	0	0	Br	1	0	0	I	3	0	0	

Error Margin (ppm):	10	DBE Range:	10.0 - 30.0	Electron Ions:	both
HC Ratio:	unlimited	Apply N Rule:	yes	Use MSn Info:	yes
Max Isotopes:	3	Isotope RI (%):	1.00	Isotope Res:	9000
MSn Iso RI (%):	10.00	MSn Logic Mode:	AND	Max Results:	500

Event#: 1 MS(E+) Ret. Time : 7.107 -> 7.267 Scan# : 1067 -> 1091

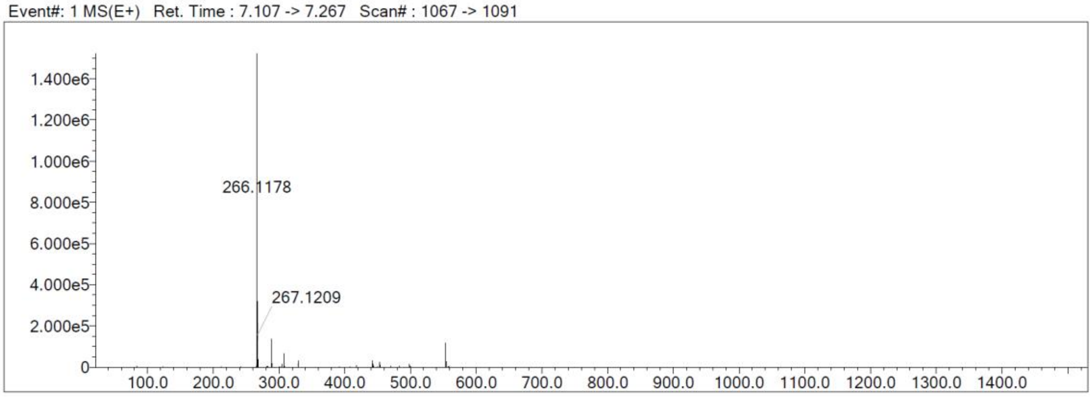

Measured region for 266.1178 m/z

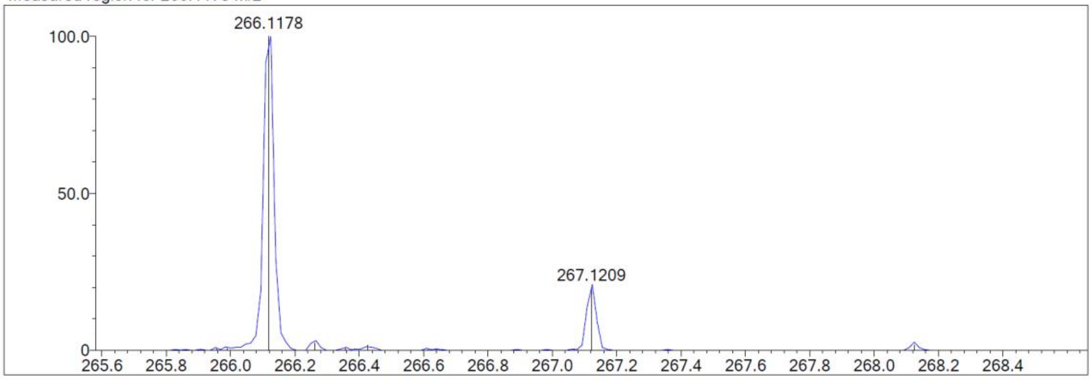

C17 H15 N O2 [M+H]+ : Predicted region for 266.1176 m/z

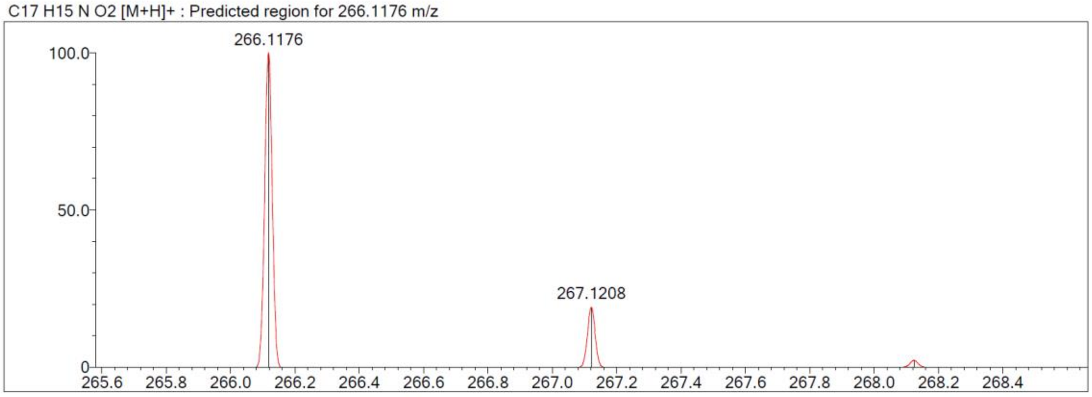

Rank	Score	Formula (M)	Ion	Meas. m/z	Pred. m/z	Df. (mDa)	Df. (ppm)	Iso	DBE
1	99.63	C17 H15 N O2	[M+H]+	266.1178	266.1176	0.2	0.75	99.63	11.0

Figure S28. Compound **B2** HRMS report.

*2-Chloro-N-(4-((5-methoxy-1-oxo-2,3-dihydro-1H-inden-2-ylidene)methyl)phenyl) acetamide **(C1)***

Yellow powder. M.P.: 175.4 °C. Yield: 82%.

IR (ATR) ν$_{max}$ (cm^{-1}): 3390 (N-H), 3188-3115 (aromatic C-H), 1712 (indanone C=O), 1672 (amide C=O), 1595-1533 (C=C), 1247 (C-N), 1091 (C-O), 823 (1,4-disubstituted benzene).

^{1}H-NMR (300 MHz, DMSO-*d$_6$*) δ (ppm): 3.90 (3H, s, OCH_3), 4.06 (2H, s, CH_2), 4.30 (2H, s, CH_2), 7.03 (1H, dd, J_1=8.49 Hz, J_2=2.25 Hz, methoxy-1-oxo-indenylidene CH), 7.17 (1H, d, J=2.01 Hz, methoxy-1-oxo-indenylidene CH), 7.41 (1H, s, C=CH), 7.71 (1H, d, J=8.45 Hz, methoxy-1-oxo-indenylidene CH), 7.74 (4H, s, disubstituted benzene CH), 10.54 (1H, s, NH).

^{13}C-NMR (75 MHz, DMSO-*d$_6$*) δ (ppm): 32.4, 56.2, 56.2, 110.6, 115.8, 119.8, 125.8, 130.9, 131.1, 131.5, 132.0, 134.8, 140.1, 153.3, 165.3, 165.4, 192.0.

HRMS (ESI) (m/z) [M+H]$^+$: $C_{19}H_{16}ClNO_3$ calculated: 342.0891, found: 342.0891.

Table S4. Hydrogen and carbon values (ppm) of compound **C1** determined by two-dimensional NMR.

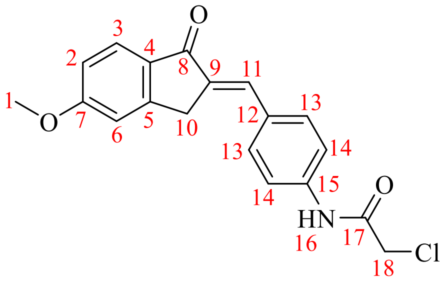

C1

Position	¹H	¹³C
**1**	3,90	56,3
**2**	7,03	115,8
**3**	7,71	125,9
**4**	-	153,3
**5**	-	131,1
**6**	7,17	110,6
**7**	-	165,4
**8**	-	192,0
**9**	-	134,9
**10**	4,06	32,5
**11**	7,41	131,5
**12**	-	131,0
**13**	7,74	132,0
**14**	7,74	119,9
**15**	-	140,1
**16**	10,54	-
**17**	-	165,3
**18**	4,30	44,1

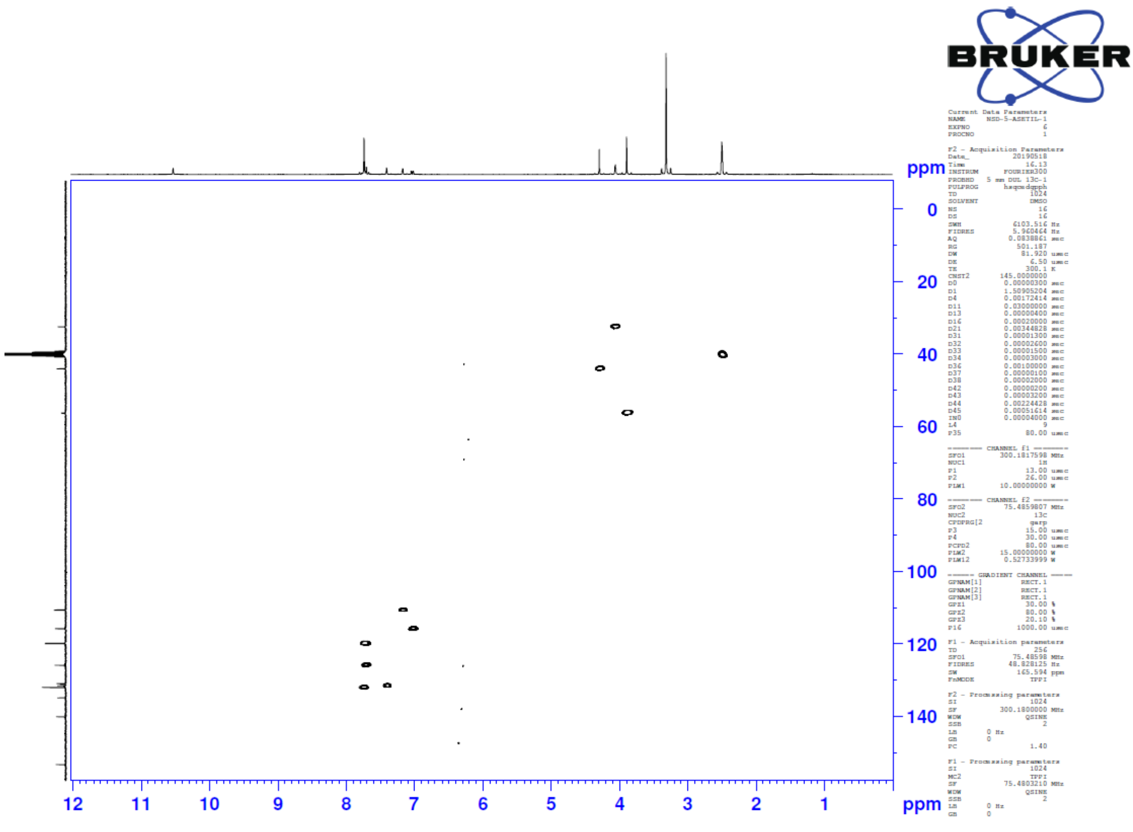

Figure S29. Compound **C1** HSQC report.

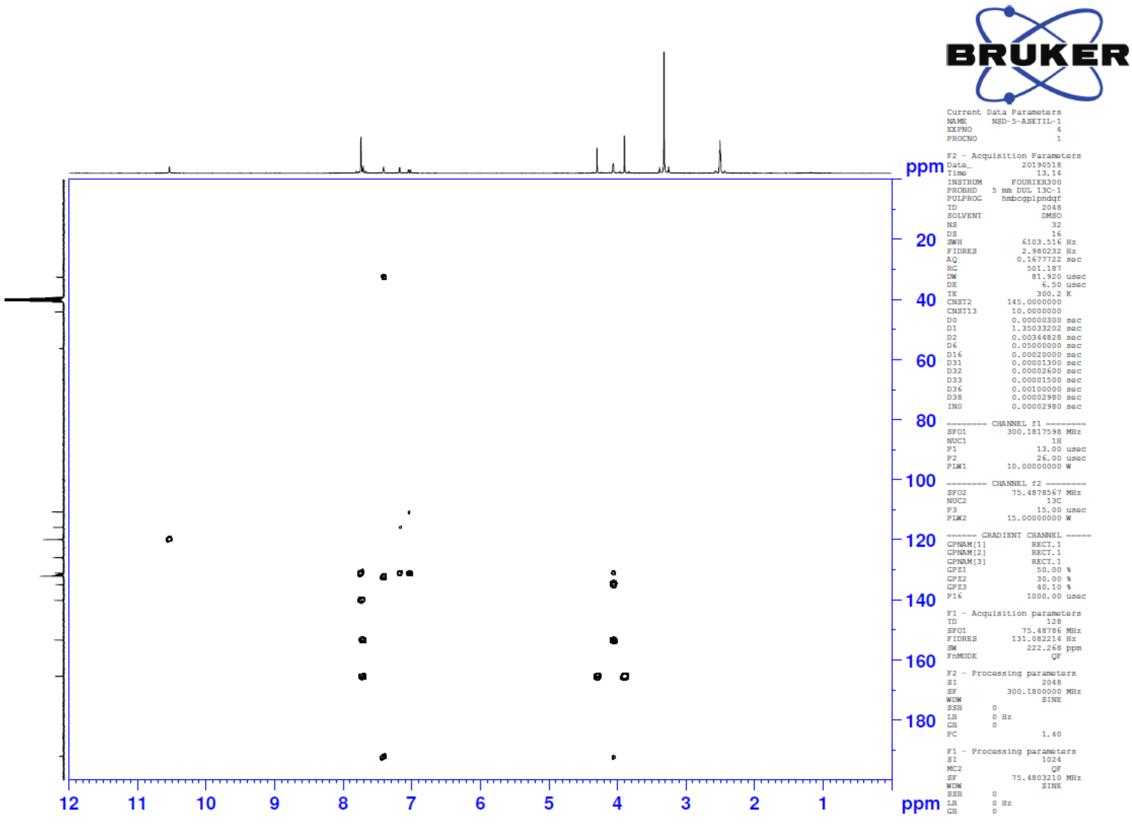

Figure S30. Compound **C1** HMBC report.

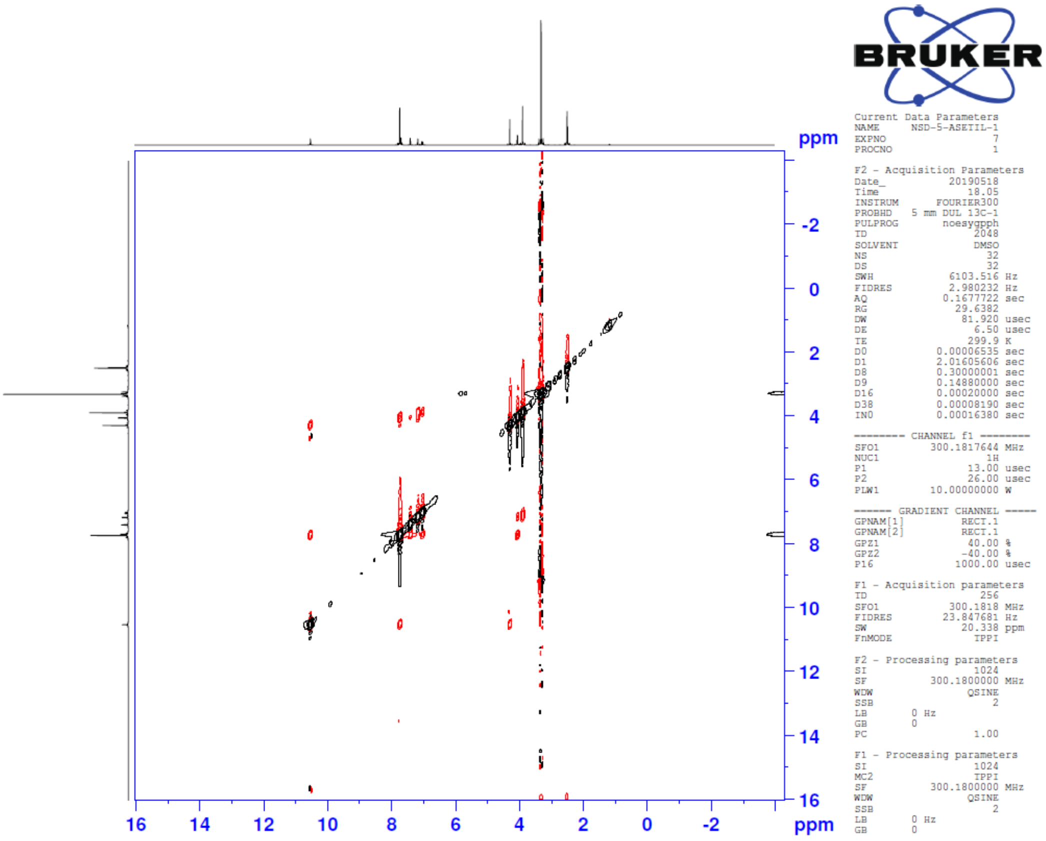

Figure S31. Compound **C1** NOESY report.

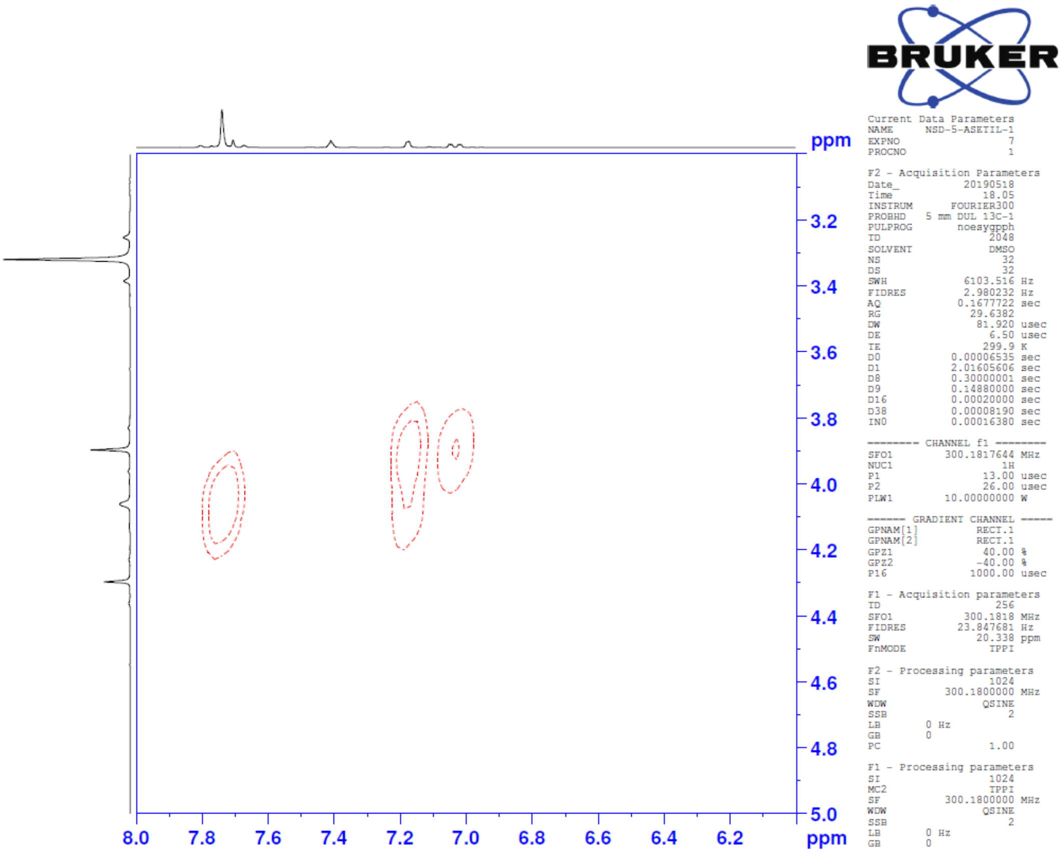

Figure S32. Compound **C1** NOESY report (zoom version).

DOPNALAB

	Item	Value
	Acquired Date&Time	22.08.2019 13:48:19
	Acquired by	System Administrator
	Filename	C:\Users\dopnalab\Desktop\NURPELİN\DOKTORA TEZ\C11.ispd
	Spectrum name	C11
	Sample name	C1
	Sample ID	
	Option	
	Comment	
	No. of Scans	50
	Resolution	4 [cm-1]
	Apodization	Happ-Genzel

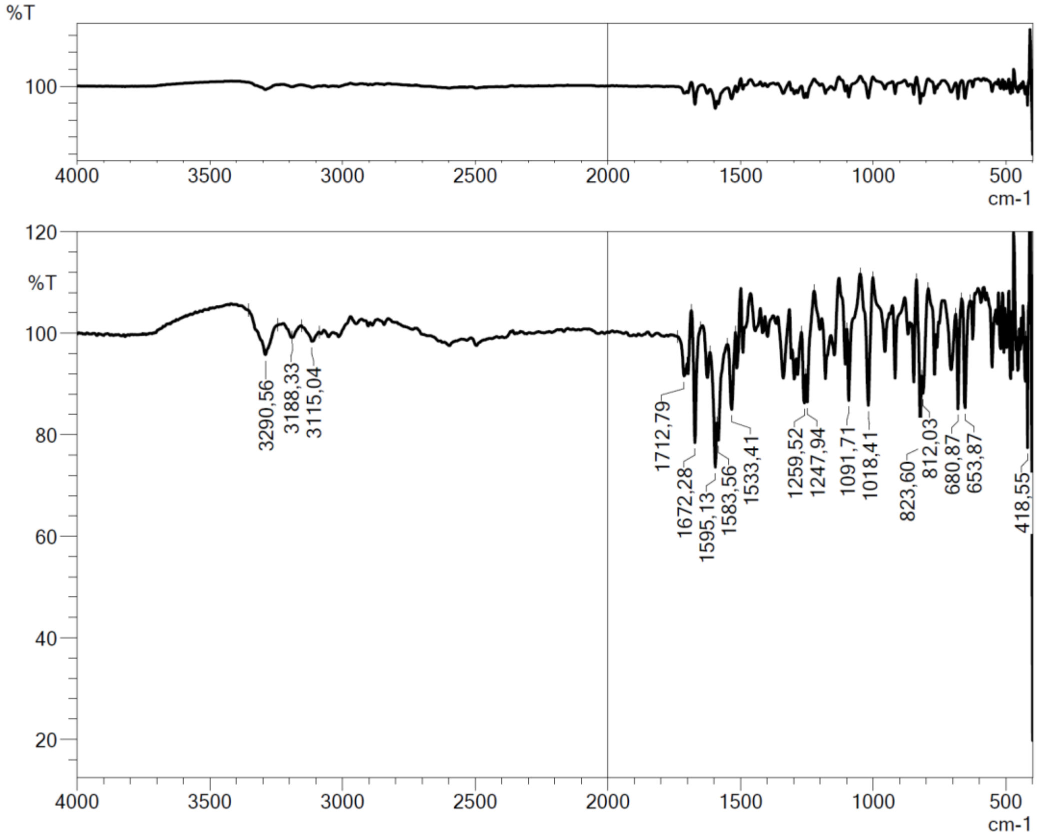

Figure S33. Compound **C1** IR report.

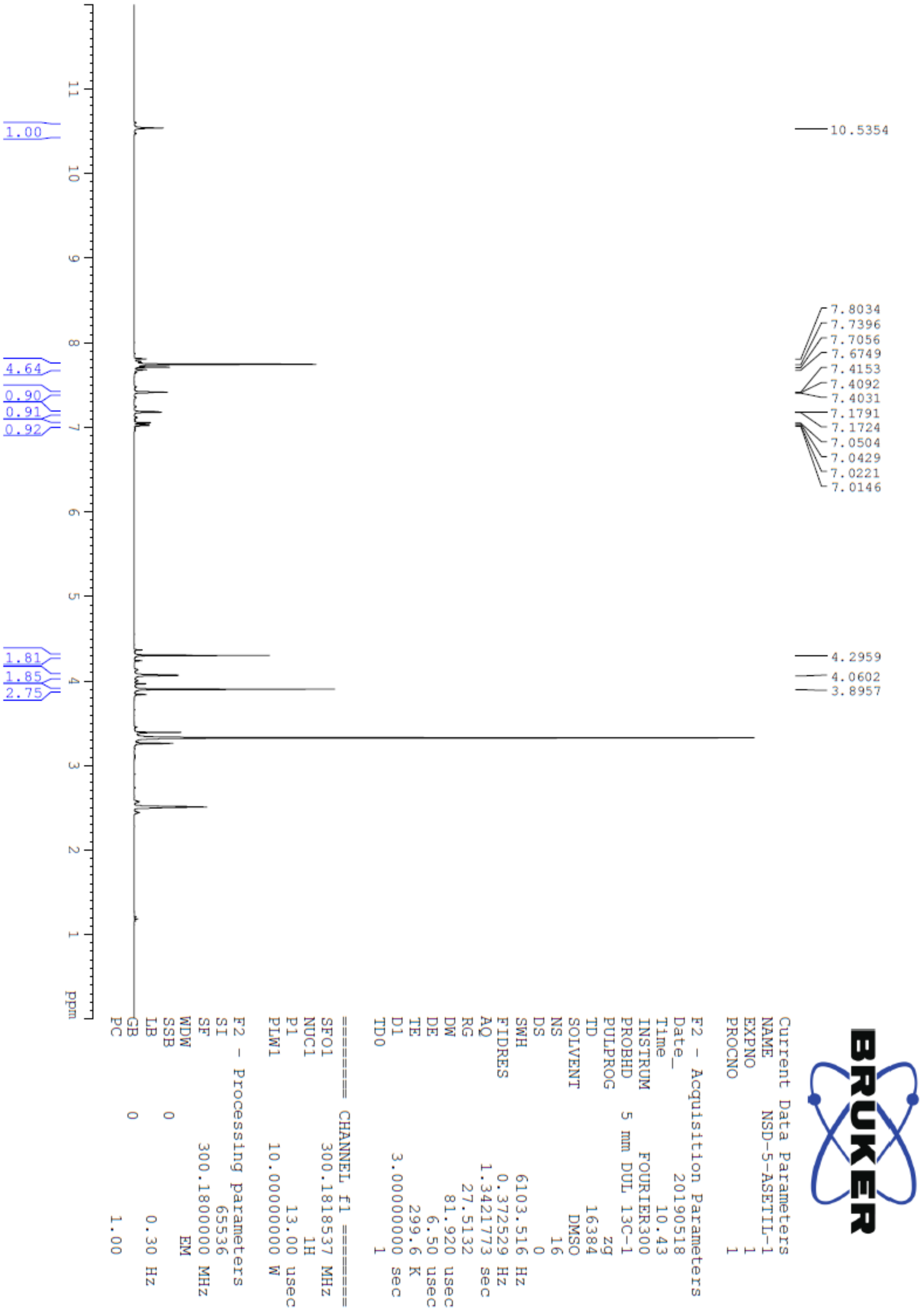

Figure S34. Compound **C1** ^{1}H-NMR spectrum.

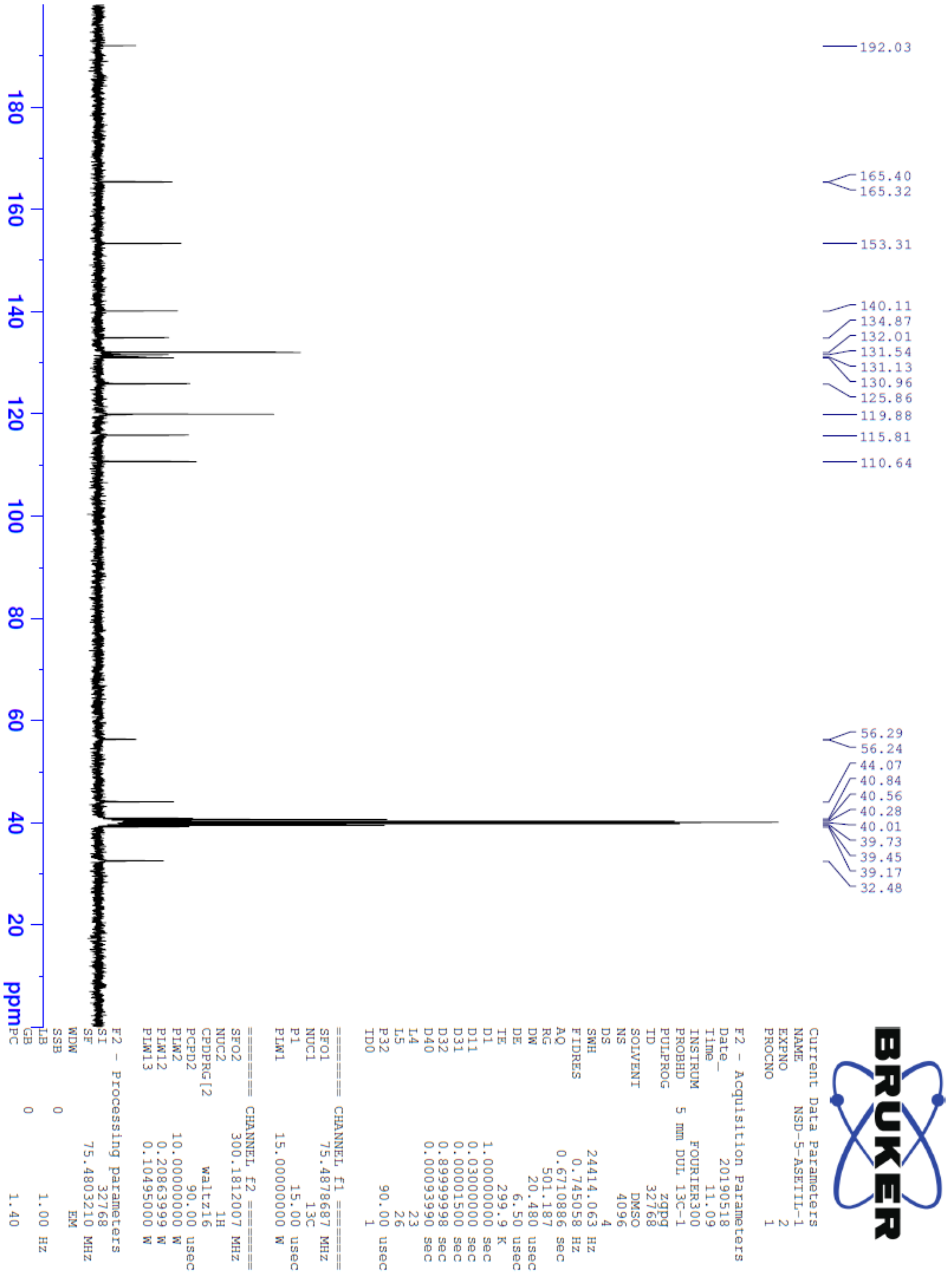

Figure S35. Compound **C1** ^{13}C-NMR spectrum.

Formula Predictor Report - C1.lcd

Page 1 of 1

Data File: C:\LabSolutions\Data\Analiz\bns\NSD-28_7.lcd

Elmt	Val.	Min	Max	Elmt	Val.	Min	Max	Elmt	Val.	Min	Max	Elmt	Val.	Min	Max	Use Adduct
H	1	0	50	O	2	2	8	S	2	0	1	Ru	2	0	0	H
C	4	0	50	F	1	0	0	Cl	1	1	1	Pd	2	0	0	
N	3	0	4	P	3	0	0	Br	1	0	0	I	3	0	0	

Error Margin (ppm):	5	DBE Range:	10.0 - 30.0	Electron Ions:	both
HC Ratio:	unlimited	Apply N Rule:	yes	Use MSn Info:	yes
Max Isotopes:	3	Isotope RI (%):	1.00	Isotope Res:	9000
MSn Iso RI (%):	10.00	MSn Logic Mode:	AND	Max Results:	500

Event#: 1 MS(E+) Ret. Time : 7.240 -> 7.373 Scan# : 1087 -> 1107

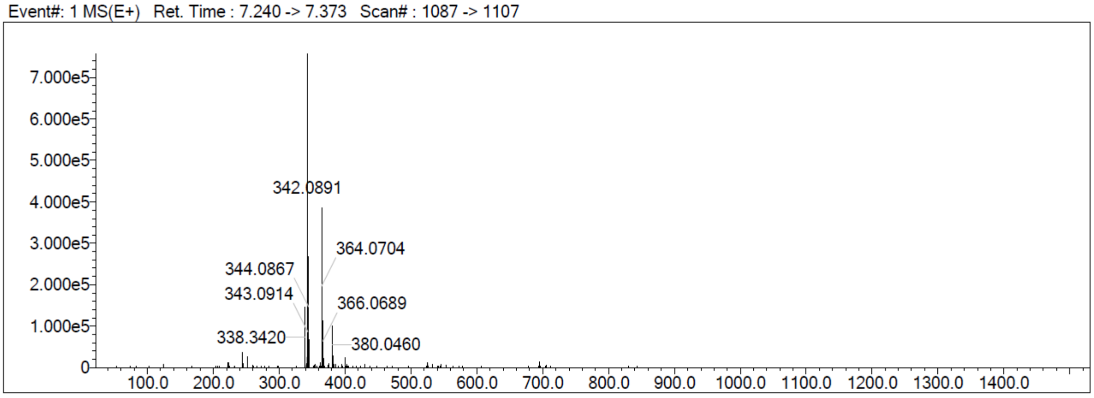

Measured region for 342.0891 m/z

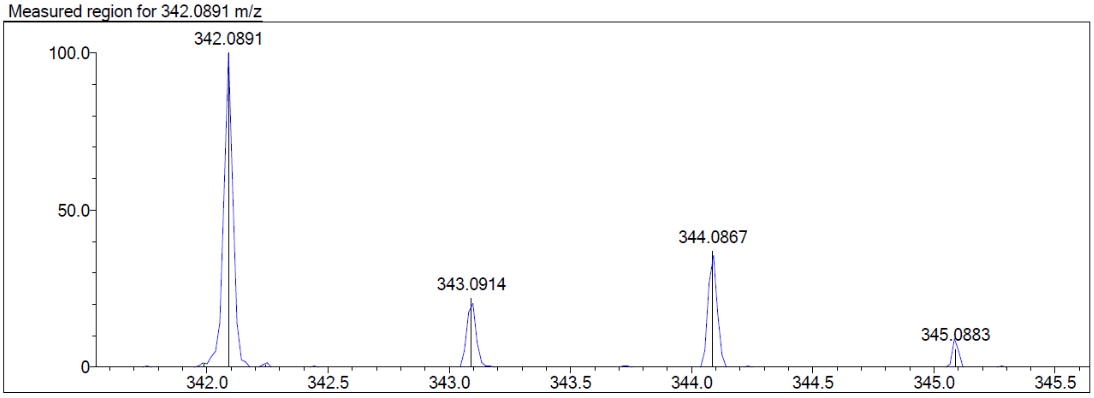

C19 H16 N O3 Cl [M+H]+ : Predicted region for 342.0891 m/z

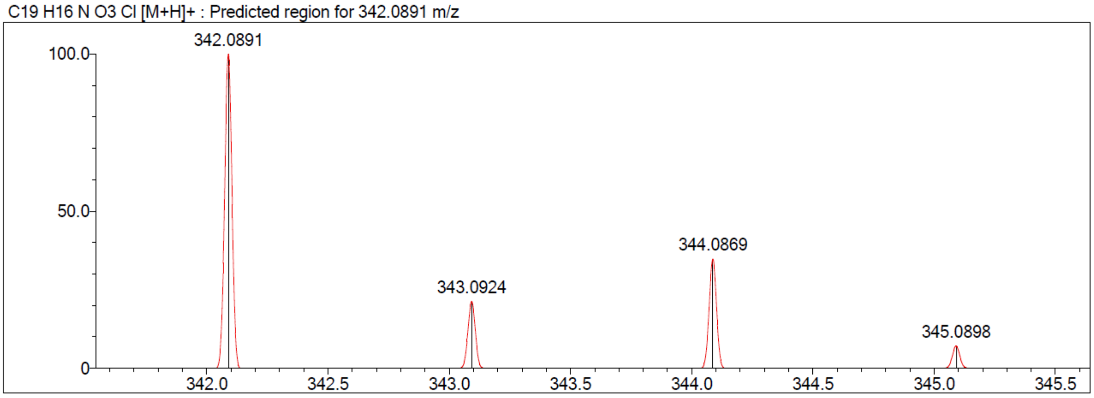

Rank	Score	Formula (M)	Ion	Meas. m/z	Pred. m/z	Df. (mDa)	Df. (ppm)	Iso	DBE
1	100.00	C19 H16 N O3 Cl	[M+H]+	342.0891	342.0891	-0.0	0.00	100.00	12.0

Figure S36. Compound **C1** HRMS report.

*2-Chloro-N-(4-((6-methoxy-1-oxo-2,3-dihydro-1H-inden-2-ylidene)methyl)phenyl) acetamide (**C2**)*

Yellow powder. M.P.: 169.2 °C. Yield: 80%.

IR (ATR) ν$_{max}$ (cm^{-1}): 3300 (N-H), 3109-2995 (aromatic C-H), 1707 (indanone C=O), 1680 (amide C=O), 1589-1487 (C=C), 1168 (C-N), 1097 (C-O), 823 (1,4-disubstituted benzene).

^{1}H-NMR (300 MHz, DMSO-*d$_6$*) δ (ppm): 3.81 (3H, s, OCH_3), 3.99 (2H, s, CH_2), 4.29 (2H, s, CH_2), 7.22 (1H, d, *J*=2.46 Hz, methoxy-1-oxo-indenylidene CH), 7.27 (1H, dd, J_1=8.28 Hz, J_2=2.58 Hz, methoxy-1-oxo-indenylidene CH), 7.46 (1H, s, C=CH), 7.54 (1H, d, *J*=8.34 Hz, methoxy-1-oxo-indenylidene CH), 7.74 (4H, s, disubstituted benzene CH), 10.54 (1H, s, NH).

^{13}C-NMR (75 MHz, DMSO-*d$_6$*) δ (ppm): 31.6, 44.0, 55.9, 55.9, 106.0, 119.8, 123.7, 127.8, 130.7, 132.2, 132.7, 134.9, 139.0, 140.3, 142.9, 159.6, 165.4, 193.5.

HRMS (ESI) (m/z) [M+H]$^+$: $C_{19}H_{16}ClNO_3$ calculated: 342.0891, found: 342.0897.

Table S5. Hydrogen and carbon values (ppm) of compound **C2** determined by two-dimensional NMR.

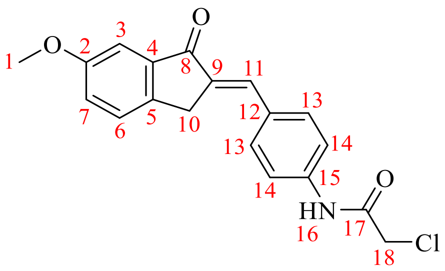

C2

Position	^{1}H	^{13}C
**1**	3,81	56,0
**2**	-	159,6
**3**	7,22	106,0
**4**	-	142,9
**5**	-	139,1
**6**	7,54	127,9
**7**	7,27	123,7
**8**	-	193,5
**9**	-	135,0
**10**	3,99	31,7
**11**	7,46	132,8
**12**	-	130,8
**13**	7,74	132,2
**14**	7,74	119,8
**15**	-	140,3
**16**	10,54	-
**17**	-	165,4
**18**	4,29	44,1

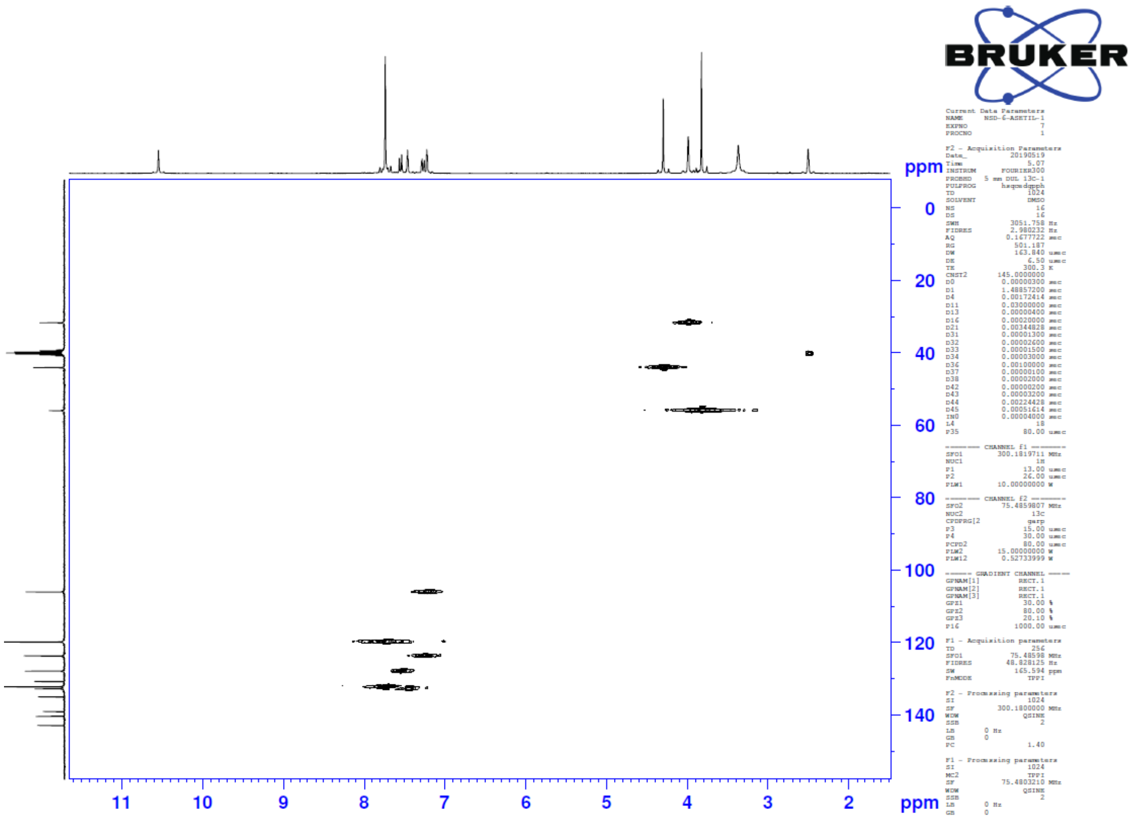

Figure S37. Compound **C2** HSQC report.

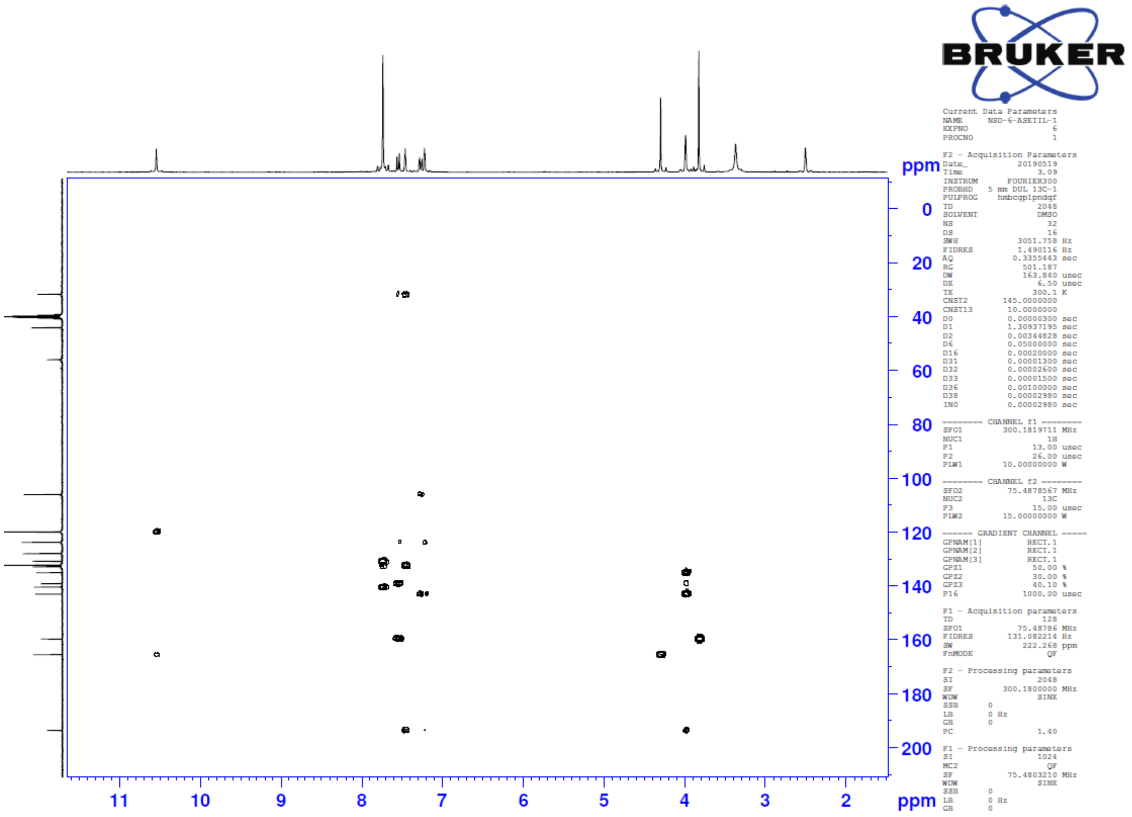

Figure S38. Compound **C2** HMBC report.

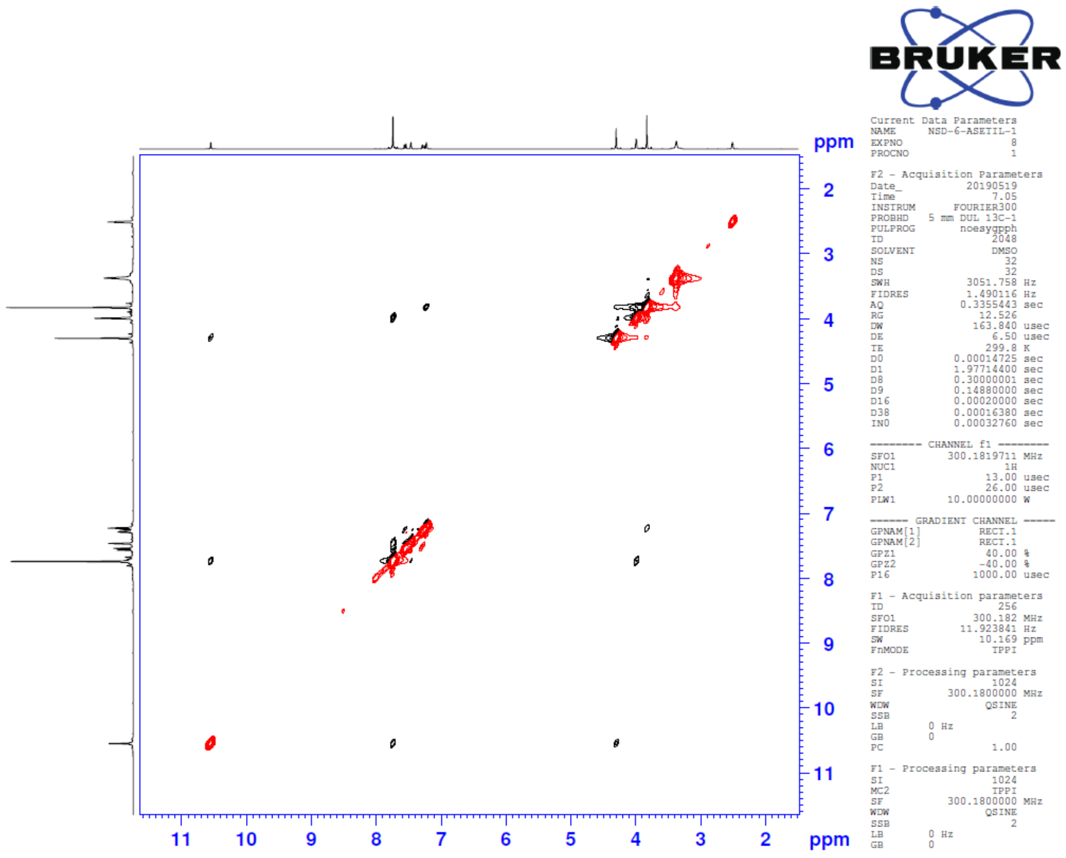

Figure S39. Compound **C2** NOESY report.

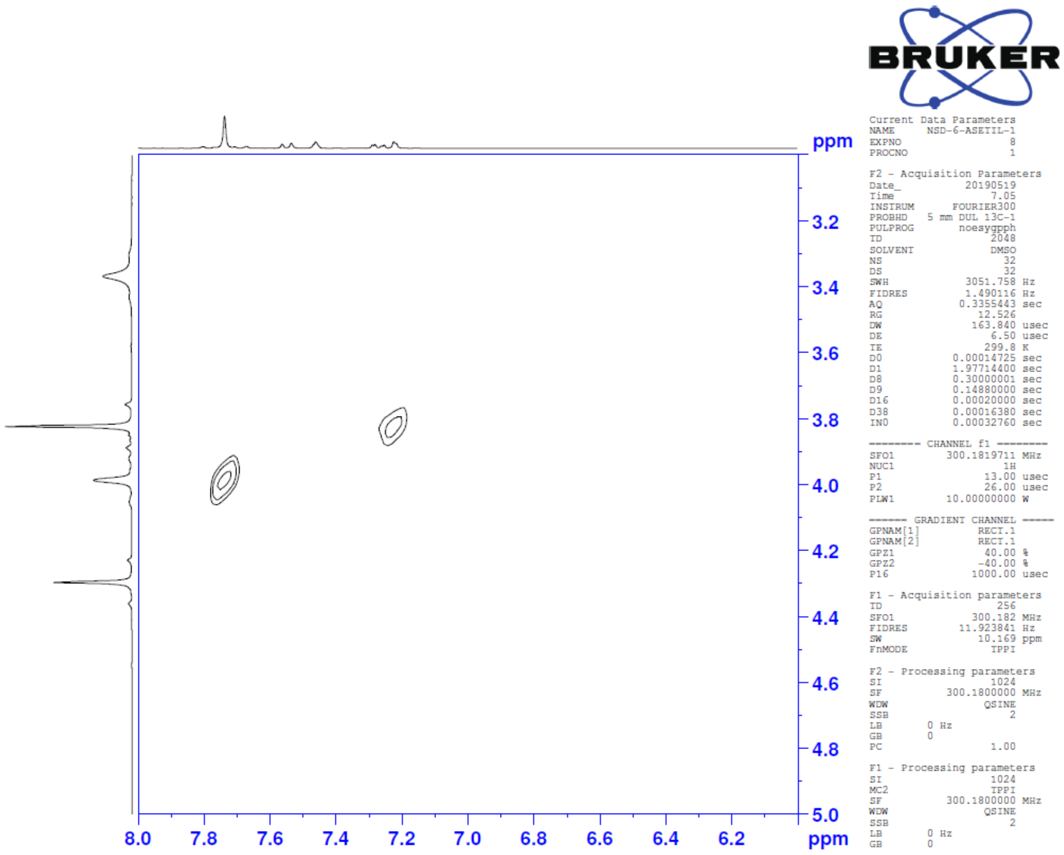

Figure S40. Compound **C2** NOESY report (zoom version).

DOPNALAB

	Item	Value
	Acquired Date&Time	22.08.2019 13:51:07
	Acquired by	System Administrator
	Filename	C:\Users\dopnalab\Desktop\NURPELİN\DOKTORA TEZ\C21.ispd
	Spectrum name	C21
	Sample name	C2
	Sample ID	
	Option	
	Comment	
	No. of Scans	50
	Resolution	4 [cm-1]
	Apodization	Happ-Genzel

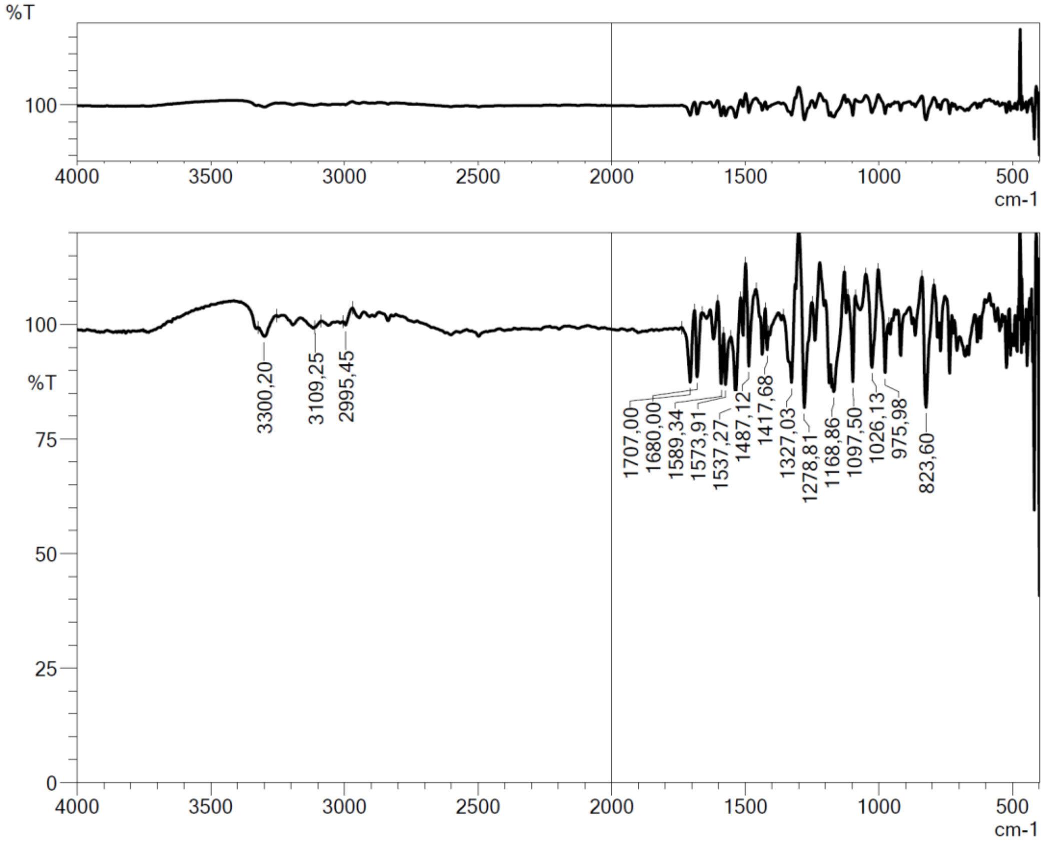

Figure S41. Compound **C2** IR report.

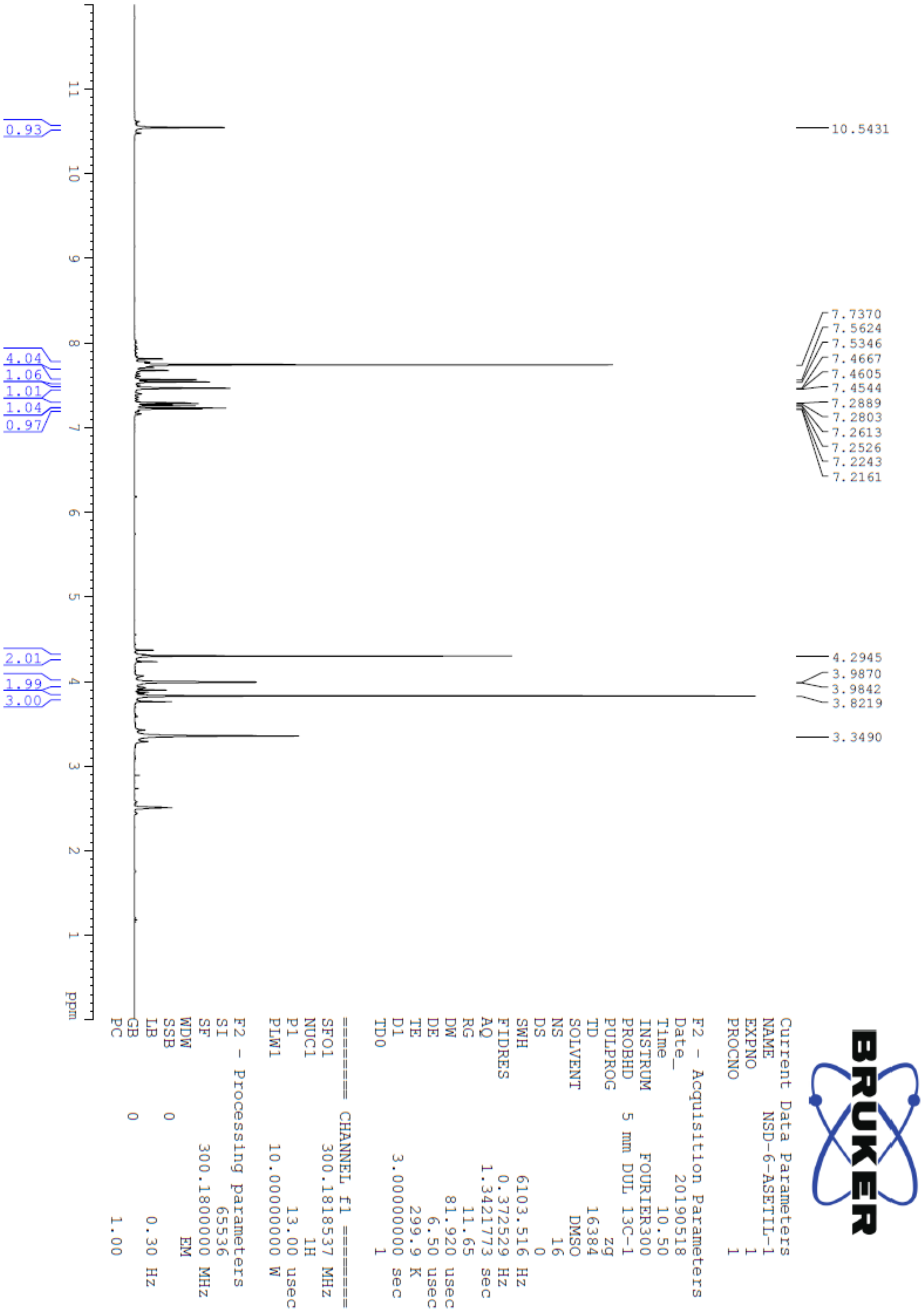

Figure S42. Compound **C2** ^{1}H-NMR spectrum.

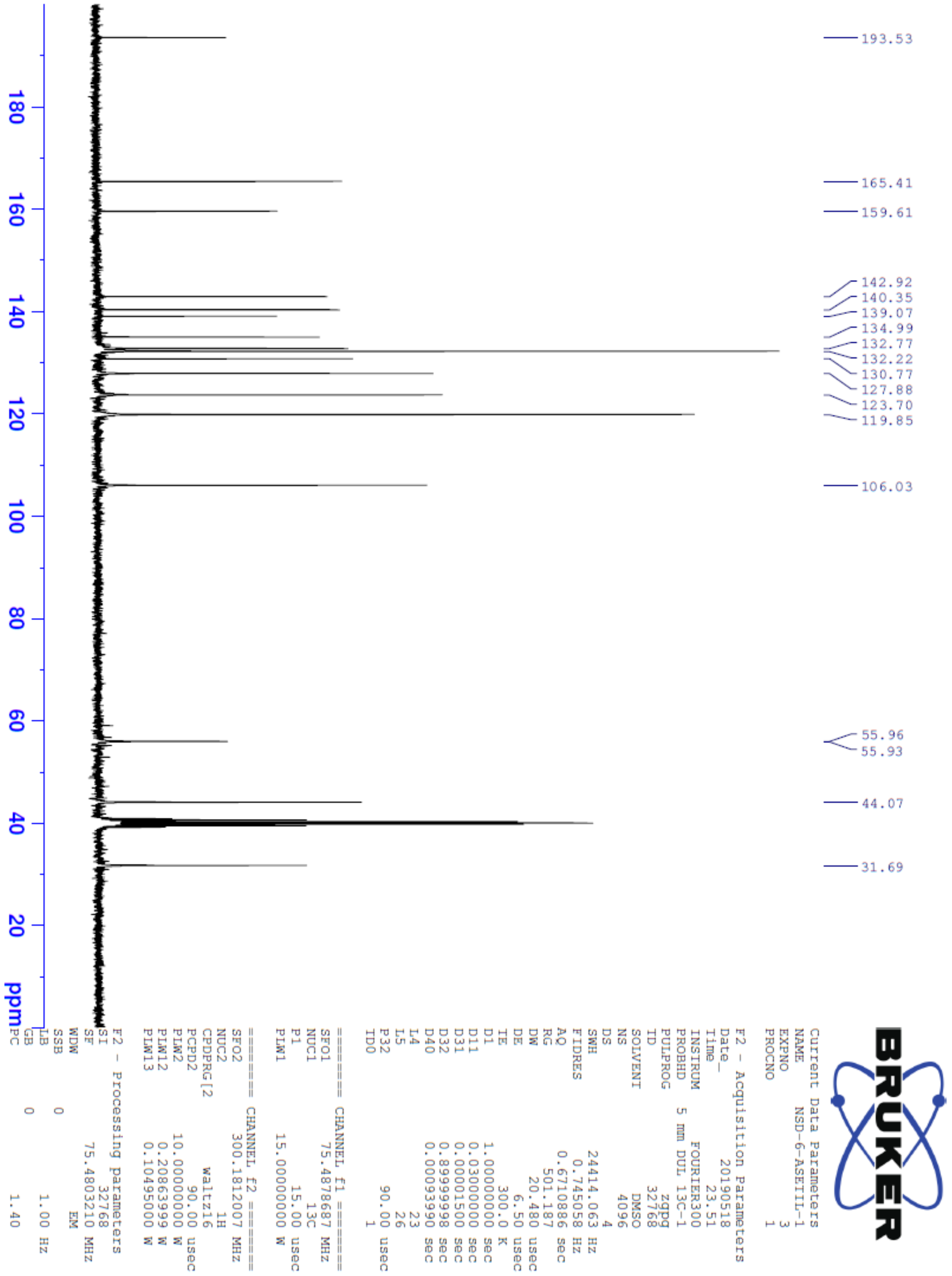

Figure S43. Compound **C2** ^{13}C-NMR spectrum.

Data File: C:\LabSolutions\Data\Analiz\Serkan\NSD-5_23.lcd

Elmt	Val.	Min	Max	Elmt	Val.	Min	Max	Elmt	Val.	Min	Max	Elmt	Val.	Min	Max	Use Adduct
H	1	0	50	O	2	2	8	S	2	0	1	Ru	2	0	0	H
C	4	0	50	F	1	0	0	Cl	1	1	1	Pd	2	0	0	
N	3	0	4	P	3	0	0	Br	1	0	0	I	3	0	0	

Error Margin (ppm):	5	DBE Range:	10.0 - 30.0	Electron Ions:	both
HC Ratio:	unlimited	Apply N Rule:	yes	Use MSn Info:	yes
Max Isotopes:	3	Isotope RI (%):	1.00	Isotope Res:	9000
MSn Iso RI (%):	10.00	MSn Logic Mode:	AND	Max Results:	500

Event#: 1 MS(E+) Ret. Time : 3.853 -> 4.040 Scan# : 579 -> 607

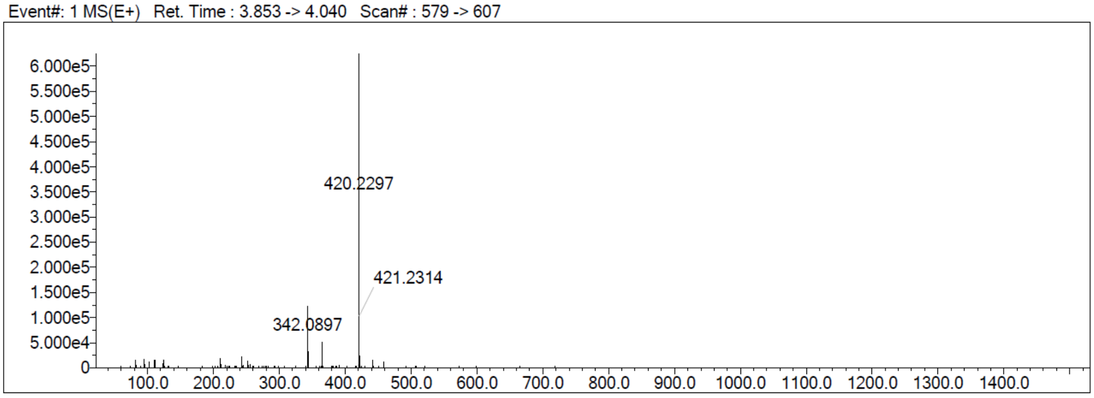

Measured region for 342.0897 m/z

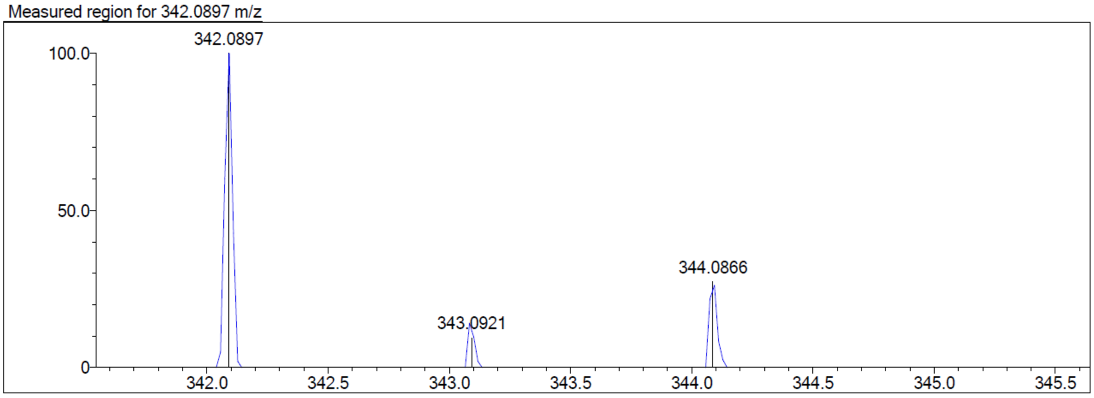

C19 H16 N O3 Cl [M+H]+ : Predicted region for 342.0891 m/z

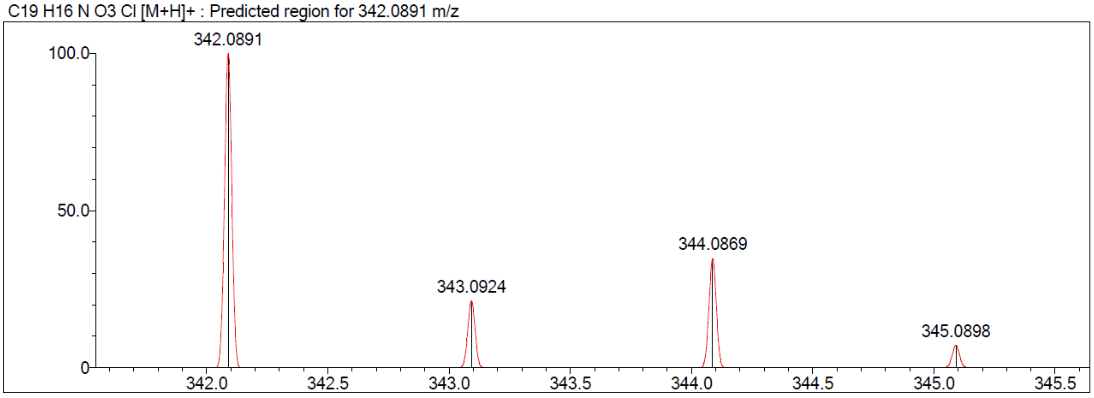

Rank	Score	Formula (M)	Ion	Meas. m/z	Pred. m/z	Df. (mDa)	Df. (ppm)	Iso	DBE
1	47.18	C19 H16 N O3 Cl	[M+H]+	342.0897	342.0891	0.6	1.75	48.08	12.0

Figure S44. Compound **C2** HRMS report.

*2-Chloro-N-(4-((5,6-dimethoxy-1-oxo-2,3-dihydro-1H-inden-2-ylidene)methyl)phenyl) acetamide (**C3**)*

Yellow powder. M.P.: 117.2 °C. Yield: 83%.

IR (ATR) ν_{max} (cm^{-1}): 3288 (N-H), 3188-3111 (aromatic C-H), 1707 (indanone C=O), 1670 (amide C=O), 1600-1506 (C=C), 1230 (C-N), 1097 (C-O), 825 (1,4-disubstituted benzene).

^{1}H-NMR (300 MHz, DMSO-d_6) δ (ppm): 3.83 (3H, s, OCH3), 3.90 (3H, s, OCH3), 3.97 (2H, s, CH2), 4.30 (2H, s, CH2), 7.18 (1H, d, J=7.14 Hz, methoxy-1-oxo-indenylidene CH), 7.21 (1H, d, J=2.45 Hz, methoxy-1-oxo-indenylidene CH), 7.37 (1H, s, C=CH), 7.72 (4H, s, disubstituted benzene CH), 10.56 (1H, s, NH).

^{13}C-NMR (75 MHz, DMSO-d_6) δ (ppm): 32.0, 44.0, 56.1, 56.1, 56.4, 56.4, 105.0, 108.4, 119.8, 130.5, 131.0, 131.0, 131.9, 135.0, 140.0, 145.4, 149.7, 155.6, 165.3, 192.2.

HRMS (ESI) (m/z) $[M+H]^+$: $C_{20}H_{18}ClNO_4$ calculated: 372.0997, found: 372.0994.

Table S6. Hydrogen and carbon values (ppm) of compound **C3** determined by two-dimensional NMR.

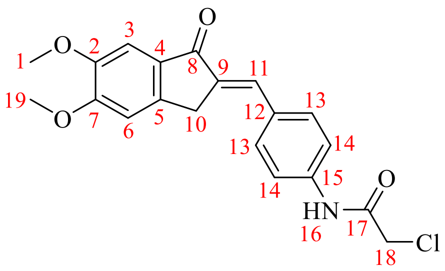

C3

**Position**	**^{1}H**	**^{13}C**
**1**	3,90	56,4
**2**	-	155,7
**3**	7,21	105,0
**4**	-	145,4
**5**	-	130,5
**6**	7,18	108,5
**7**	-	149,8
**8**	-	192,3
**9**	-	135,1
**10**	3,97	32,1
**11**	7,37	131,1
**12**	-	131,0
**13**	7,72	131,9
**14**	7,72	119,8
**15**	-	140,1
**16**	10,56	-
**17**	-	165,4
**18**	4,30	44,1
**19**	3,83	56,1

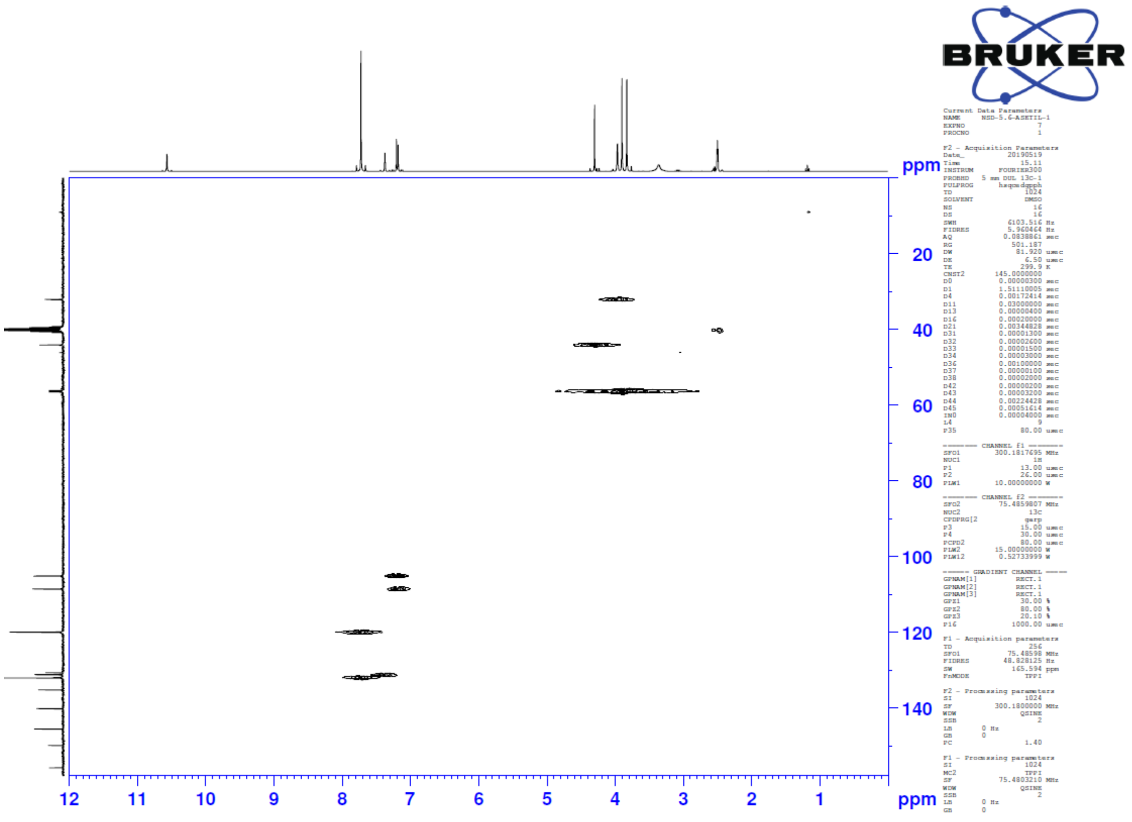

Figure S45. Compound **C3** HSQC report.

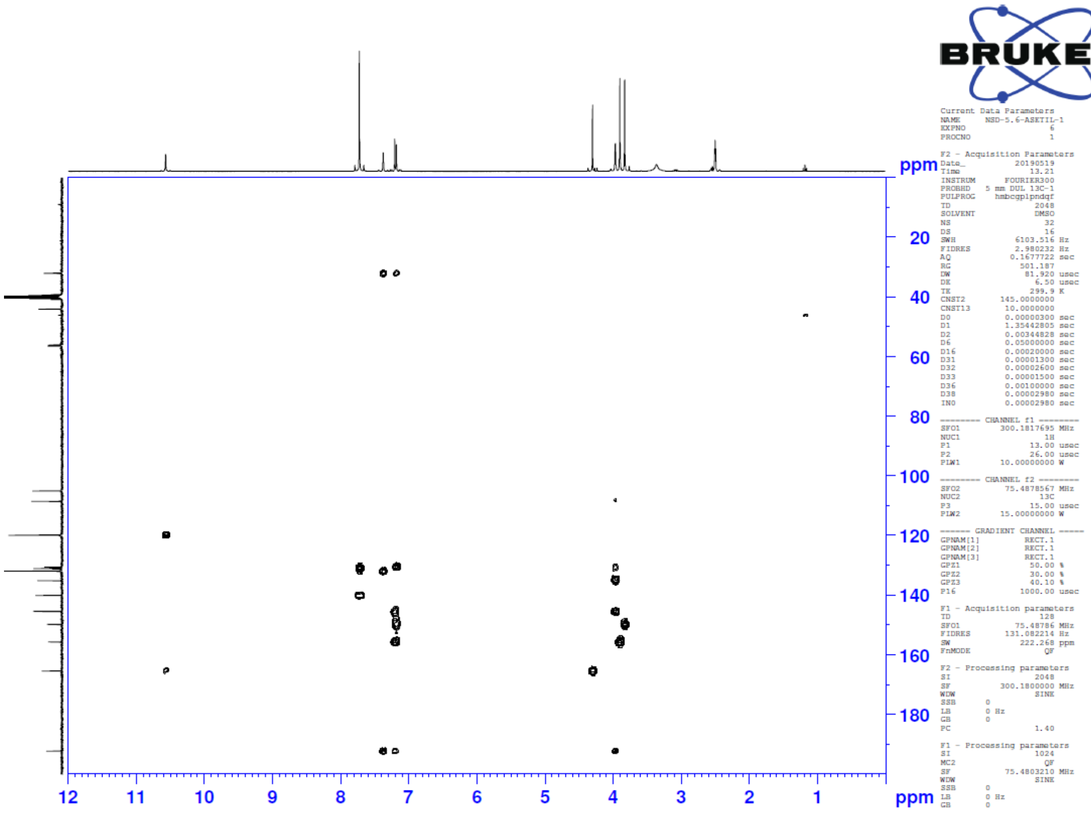

Figure S46. Compound **C3** HMBC report.

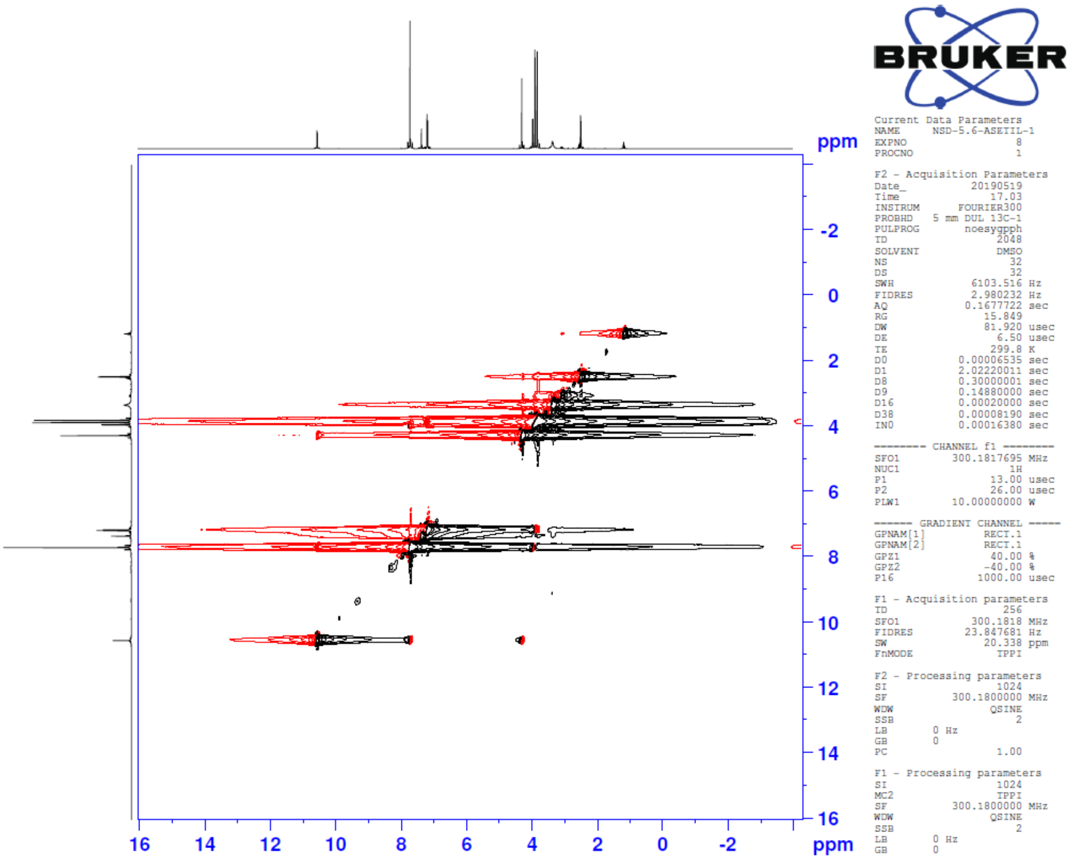

Figure S47. Compound **C3** NOESY report.

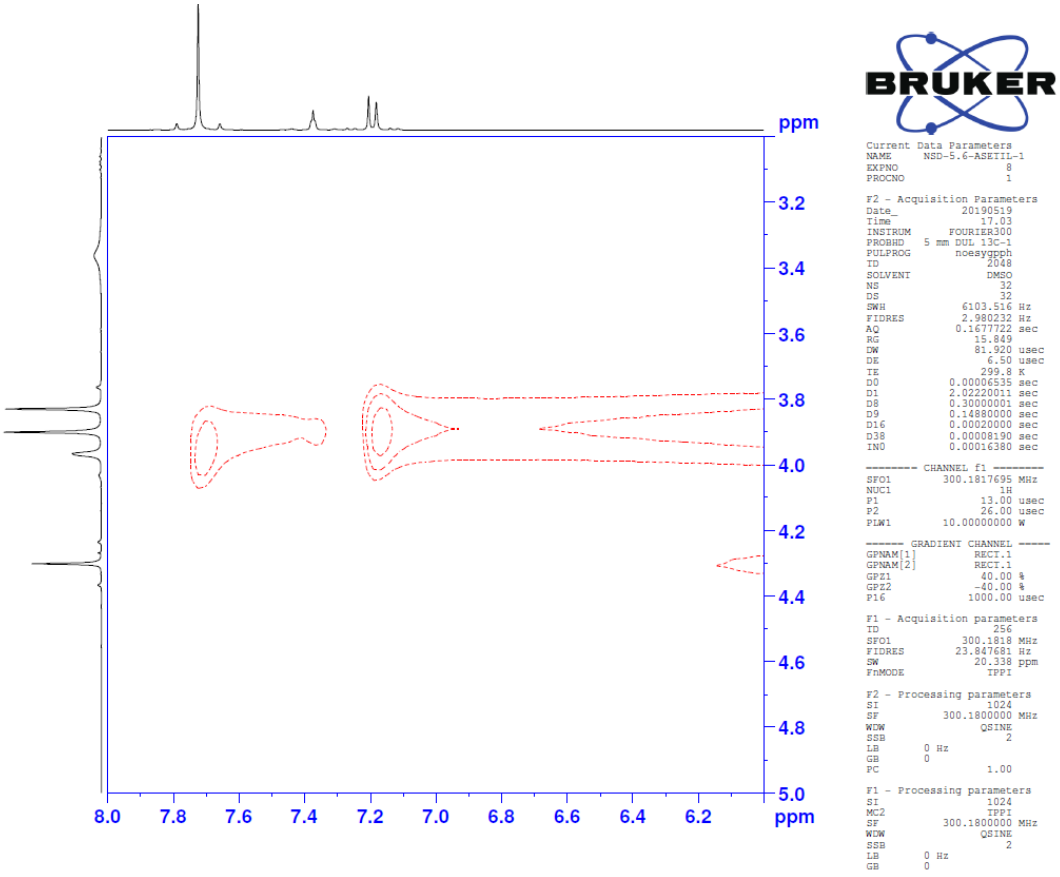

Figure S48. Compound **C3** NOESY report (zoom version).

DOPNALAB

	Item	Value
	Acquired Date&Time	22.08.2019 13:53:53
	Acquired by	System Administrator
	Filename	C:\Users\dopnalab\Desktop\NURPELİN\DOKTORA TEZ\C31.ispd
	Spectrum name	C31
	Sample name	C3
	Sample ID	
	Option	
	Comment	
	No. of Scans	50
	Resolution	4 [cm-1]
	Apodization	Happ-Genzel

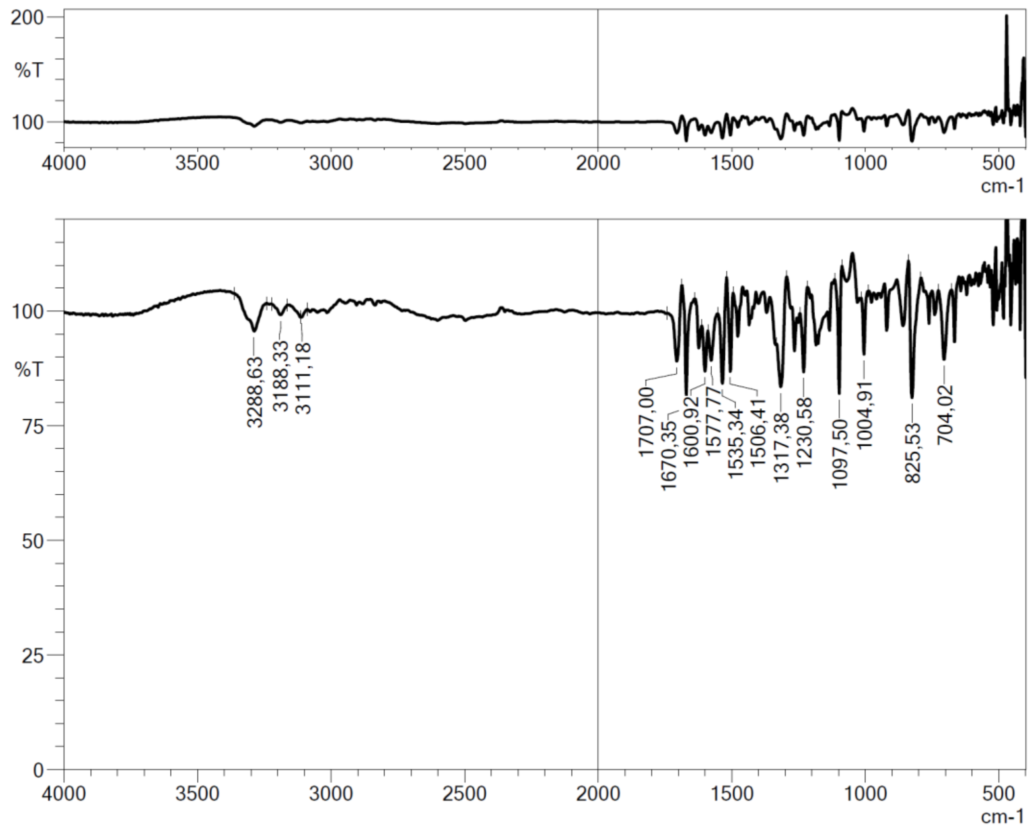

Figure S49. Compound **C3** IR report.

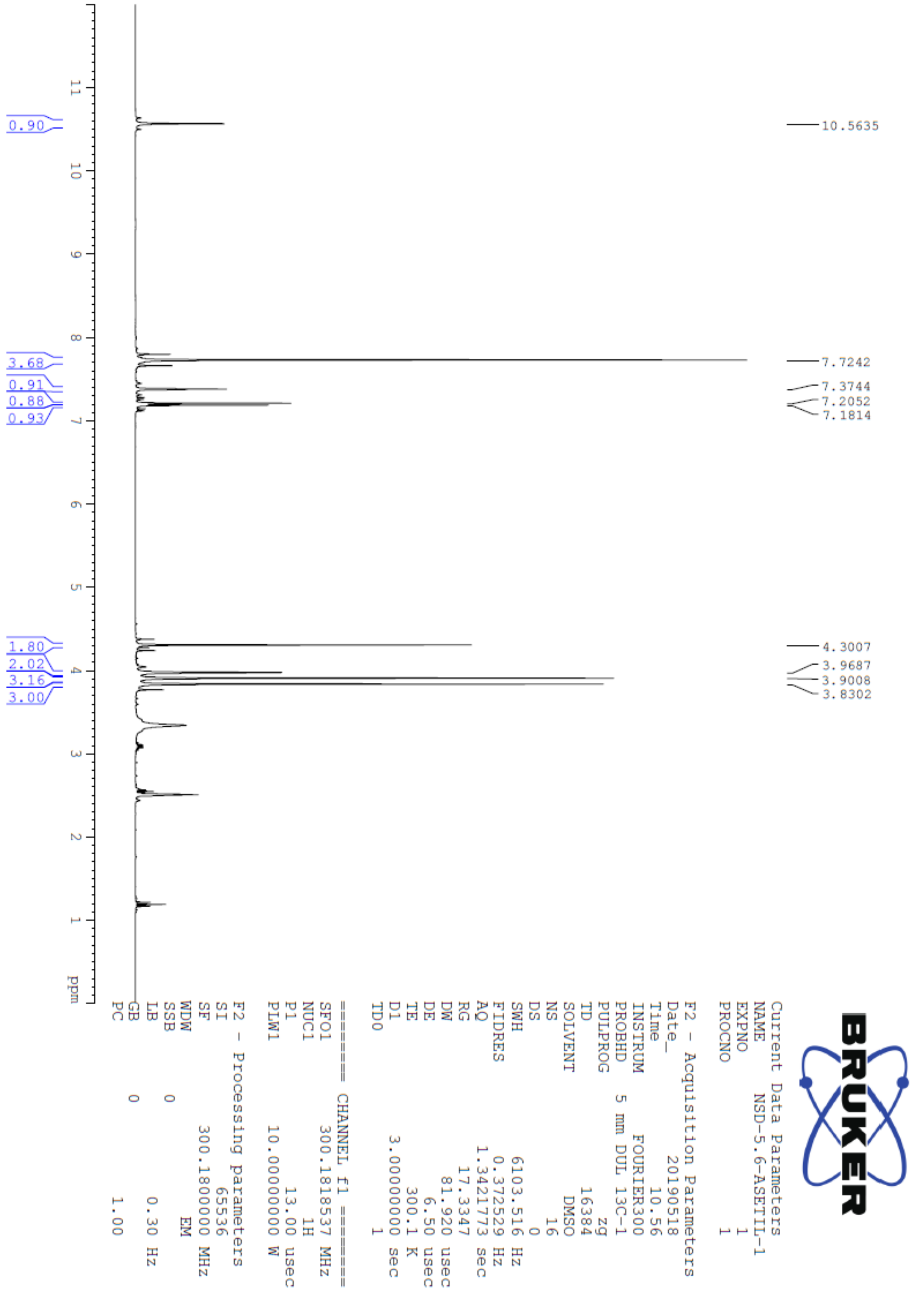

Figure S50. Compound **C3** ^{1}H-NMR spectrum.

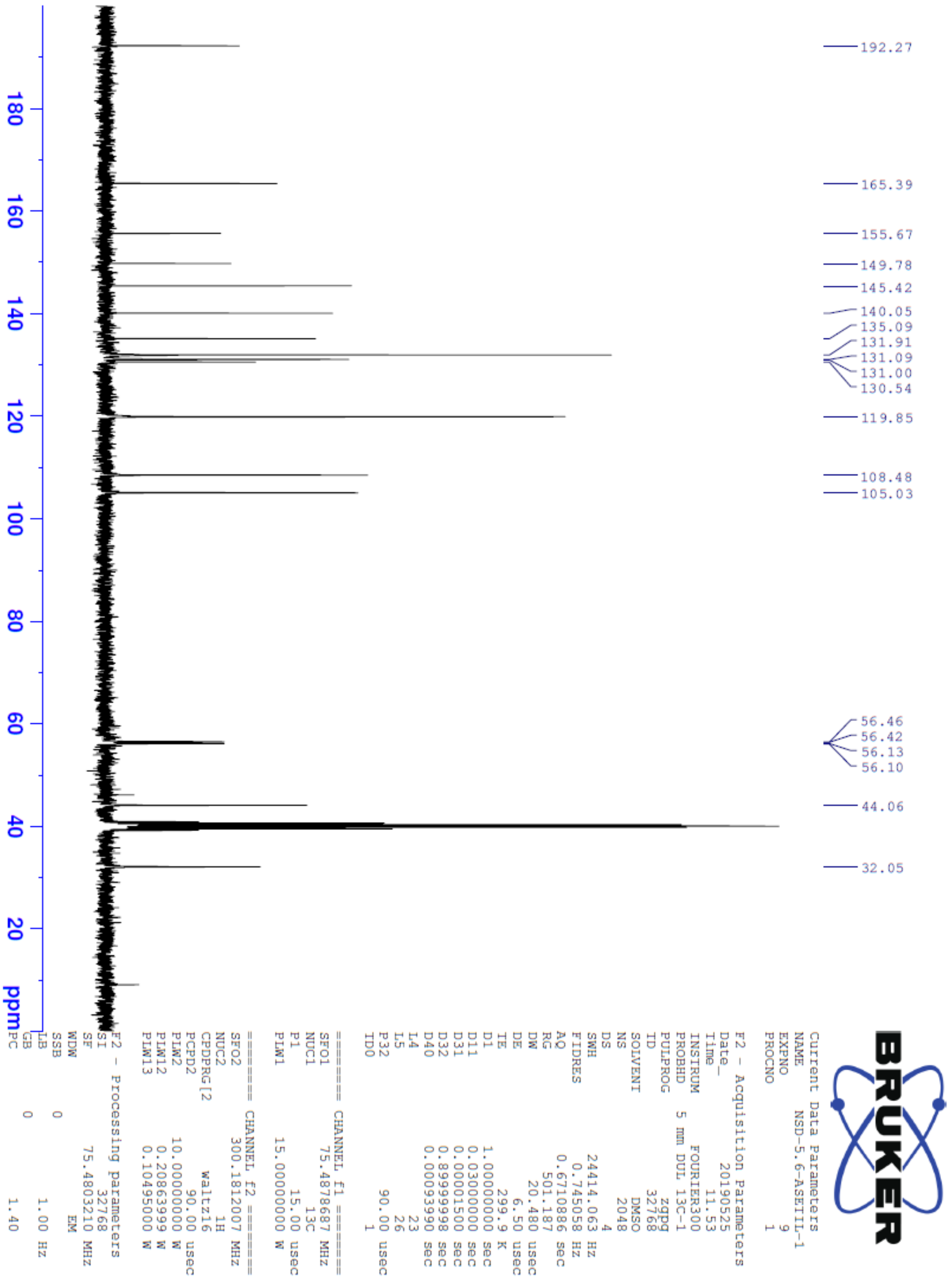

Figure S51. Compound **C3** ^{13}C-NMR spectrum.

Data File: C:\LabSolutions\Data\Analiz\bns\NSD-33_9.lcd

Elmt	Val.	Min	Max	Elmt	Val.	Min	Max	Elmt	Val.	Min	Max	Elmt	Val.	Min	Max	Use Adduct
H	1	0	50	O	2	2	8	S	2	0	1	Ru	2	0	0	H
C	4	0	50	F	1	0	0	Cl	1	1	1	Pd	2	0	0	
N	3	0	4	P	3	0	0	Br	1	0	0	I	3	0	0	

Error Margin (ppm):	5	DBE Range:	10.0 - 30.0	Electron Ions:	both
HC Ratio:	unlimited	Apply N Rule:	yes	Use MSn Info:	yes
Max Isotopes:	3	Isotope RI (%):	1.00	Isotope Res:	9000
MSn Iso RI (%):	10.00	MSn Logic Mode:	AND	Max Results:	500

Event#: 1 MS(E+) Ret. Time : 6.827 -> 6.960 Scan# : 1025 -> 1045

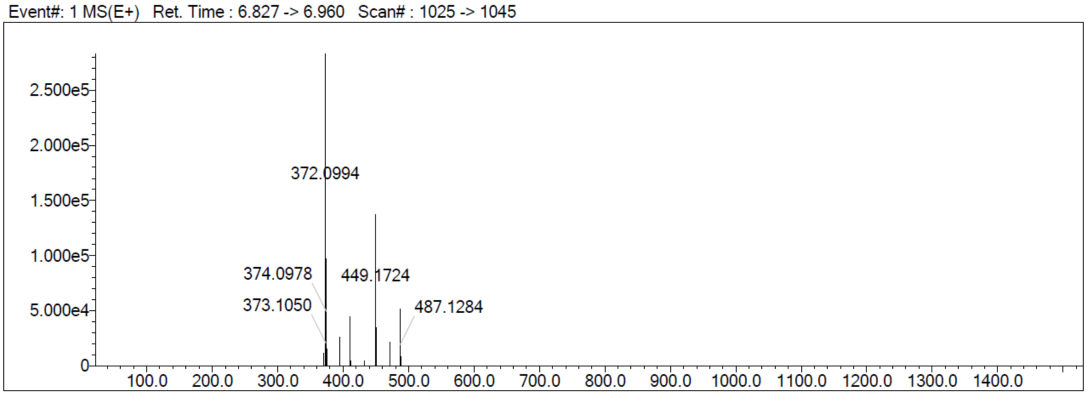

Measured region for 372.0994 m/z

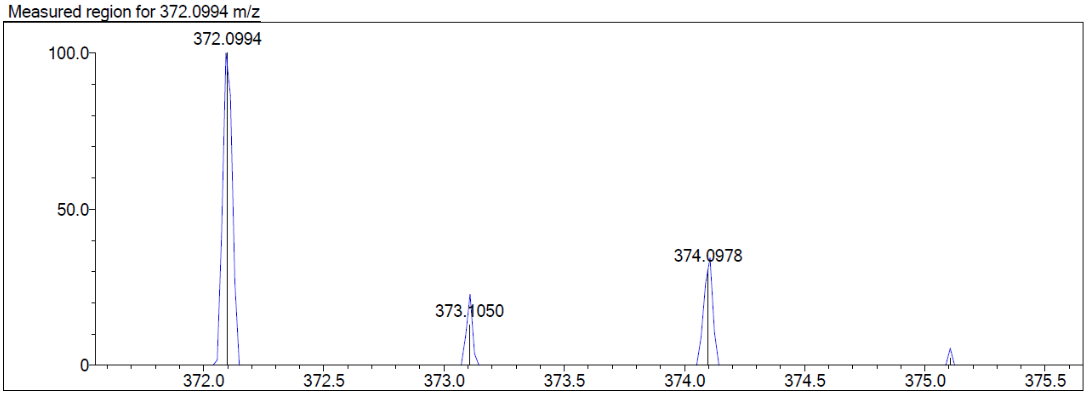

C20 H18 N O4 Cl [M+H]+ : Predicted region for 372.0997 m/z

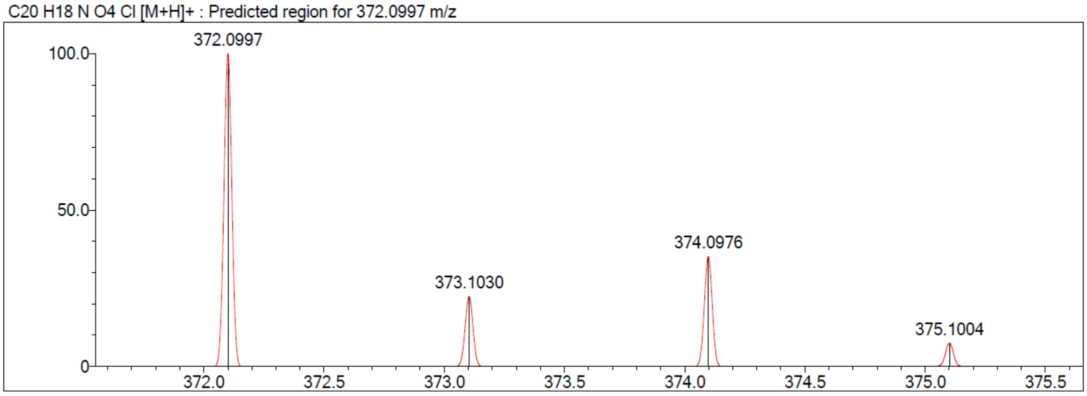

Rank	Score	Formula (M)	Ion	Meas. m/z	Pred. m/z	Df. (mDa)	Df. (ppm)	Iso	DBE
1	72.91	C20 H18 N O4 Cl	[M+H]+	372.0994	372.0997	-0.3	-0.81	72.91	12.0

Figure S52. Compound **C3** HRMS report.

*2-(4-Methylpiperazine-1-yl)-N-(4-((5-methoxy-1-oxo-2,3-dihydro-1H-inden-2-ylidene)methyl) phenyl)acetamide (**D1**)*

Light brown powder. M.P.: 145.7 °C. Yield: 89%.

IR (ATR) ν_{max} (cm^{-1}): 3346 (N-H), 1685 (indanone C=O), 1631 (amide C=O), 1558-1517 (C=C), 1251 (C-N), 1103 (C-O), 842 (1,4-disubstituted benzene).

^{1}H-NMR (300 MHz, DMSO-d_6) δ (ppm): 2.17 (3H, s, CH_3), 2.38 (4H, bs, piperazine CH_2), 2.52 (4H, bs, piperazine CH_2), 3.15 (2H, s, CH_2), 3.90 (3H, s, OCH_3), 4.05 (2H, s, CH_2), 7.03 (1H, dd, J_1=8.49 Hz, J_2=2.25 Hz, methoxy-1-oxo-indenylidene CH), 7.18 (1H, d, J=2.01 Hz, methoxy-1-oxo-indenylidene CH), 7.40 (1H, s, C=CH), 7.70-7.73 (3H, m, disubstituted benzene CH, methoxy-1-oxo-indenylidene CH), 7.79 (2H, d, J=8.76 Hz, disubstituted benzene CH), 9.97 (1H, s, NH).

^{13}C-NMR (75 MHz, DMSO-d_6) δ (ppm): 32.5, 46.2, 53.2, 55.0, 56.3, 62.3, 110.6, 115.8, 119.9, 125.8, 130.5, 131.1, 131.7, 131.9, 134.5, 140.4, 153.3, 165.3, 169.1, 192.1.

HRMS (ESI) (m/z) $[M+H]^+$: $C_{24}H_{27}N_3O_3$ calculated: 406.2125, found: 406.2137.

DOPNALAB

Item	Value
Acquired Date&Time	22.08.2019 11:06:24
Acquired by	System Administrator
Filename	C:\Users\dopnalab\Desktop\NURPELİN\DOKTORA TEZ\D13.ispd
Spectrum name	D13
Sample name	D1
Sample ID	
Option	
Comment	
No. of Scans	50
Resolution	4 [cm-1]
Apodization	Happ-Genzel

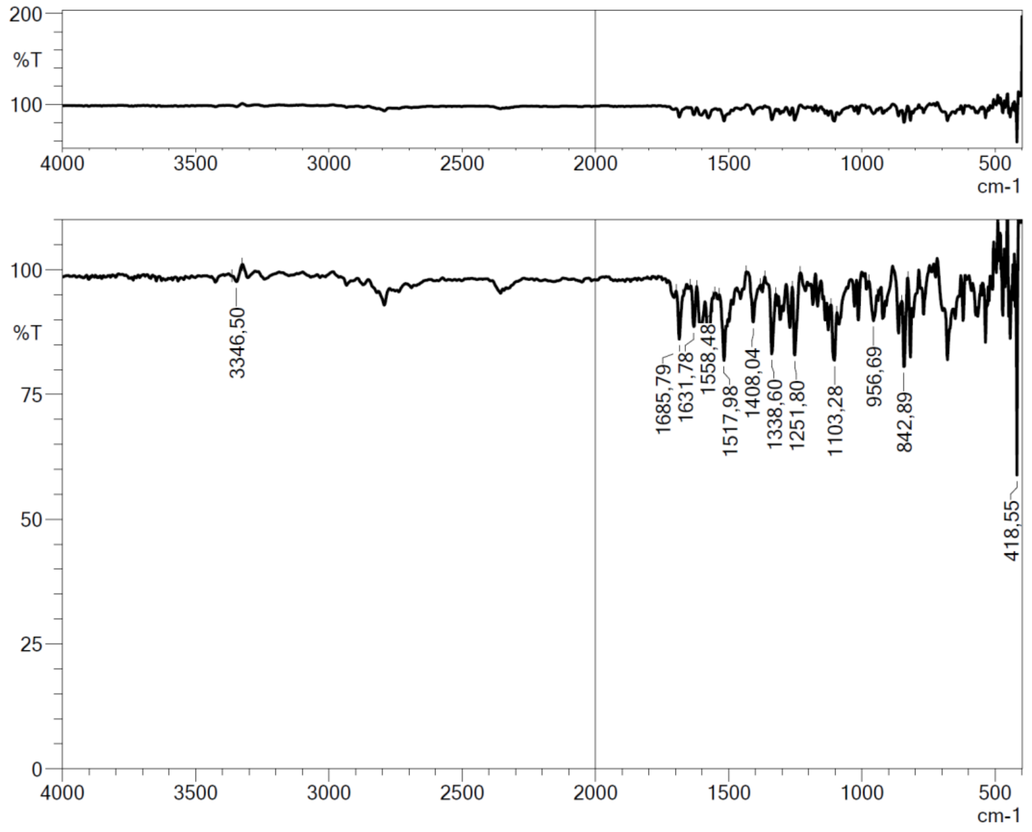

Figure S53. Compound **D1** IR report.

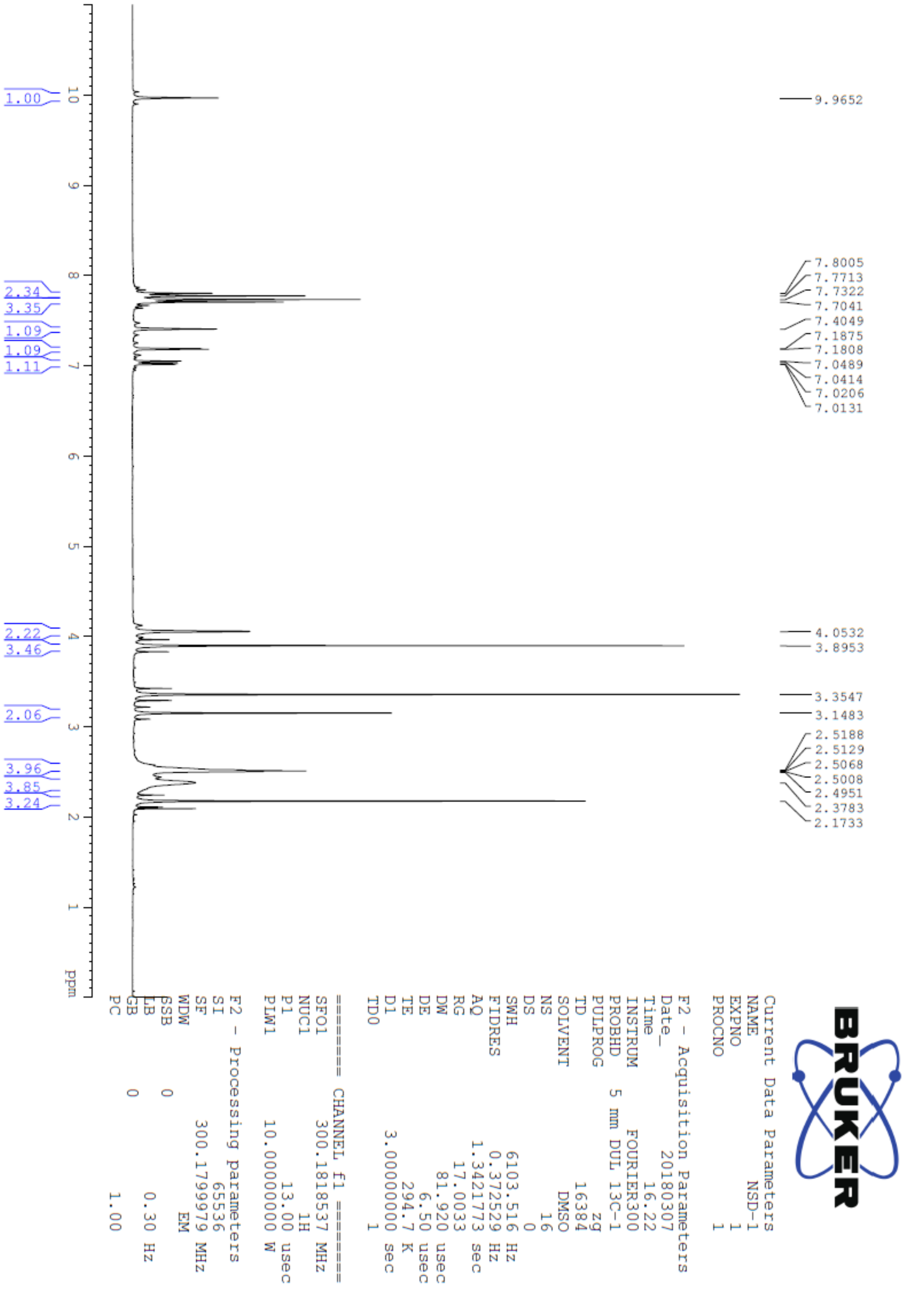

Figure S54. Compound **D1** ^{1}H-NMR spectrum.

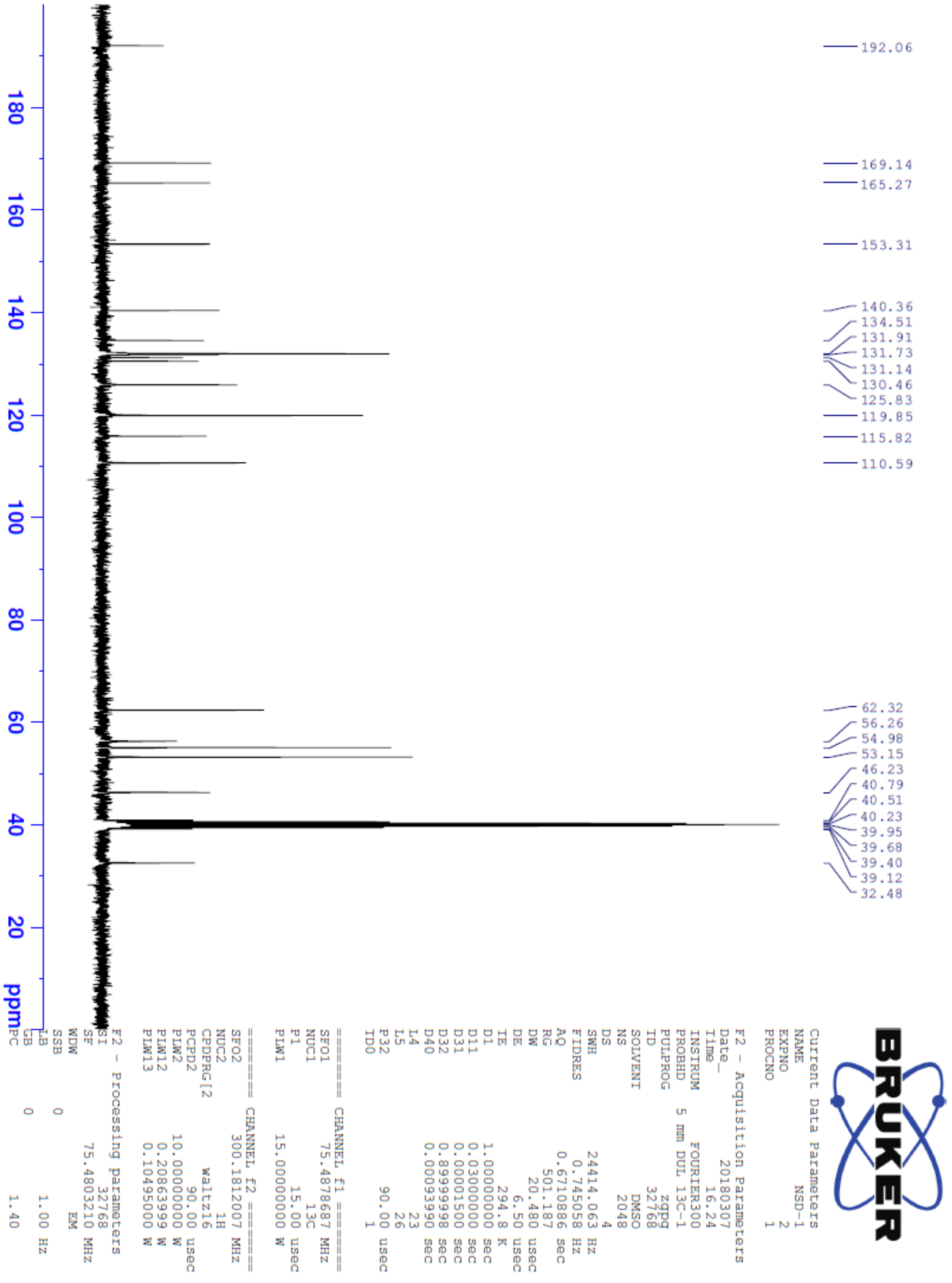

Figure S55. Compound **D1** ^{13}C-NMR spectrum.

Data File: C:\LabSolutions\Data\Analiz\Serkan\NSD-1_19.lcd

Elmt	Val.	Min	Max	Elmt	Val.	Min	Max	Elmt	Val.	Min	Max	Elmt	Val.	Min	Max	Use Adduct
H	1	5	40	O	2	0	5	S	2	0	3	Ru	2	0	0	H
C	4	0	35	F	1	0	0	Cl	1	0	2	I	3	0	0	
N	3	3	6	P	3	0	0	Br	1	0	0					

Error Margin (ppm):	5	DBE Range:	9.0 - 17.0	Electron Ions:	both
HC Ratio:	unlimited	Apply N Rule:	yes	Use MSn Info:	yes
Max Isotopes:	3	Isotope RI (%):	1.00	Isotope Res:	9000
MSn Iso RI (%):	10.00	MSn Logic Mode:	AND	Max Results:	500

Event#: 1 MS(E+) Ret. Time : 2.147 -> 2.360 Scan# : 323 -> 355

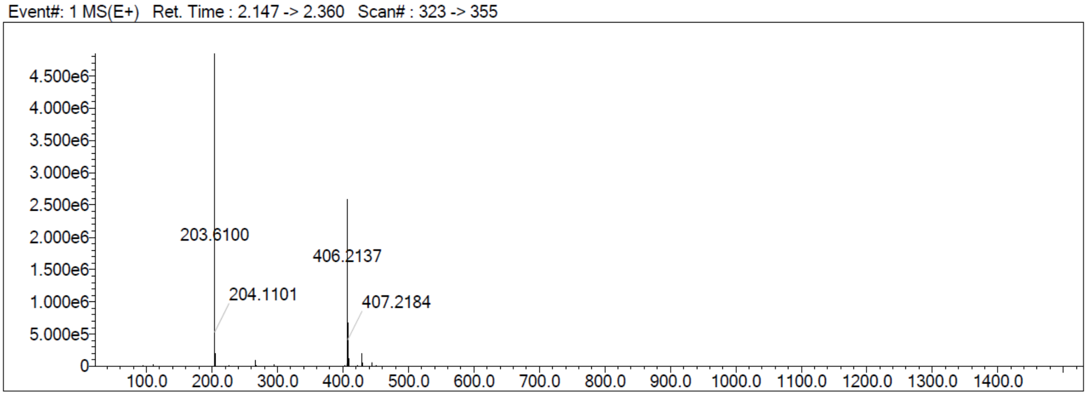

Measured region for 406.2137 m/z

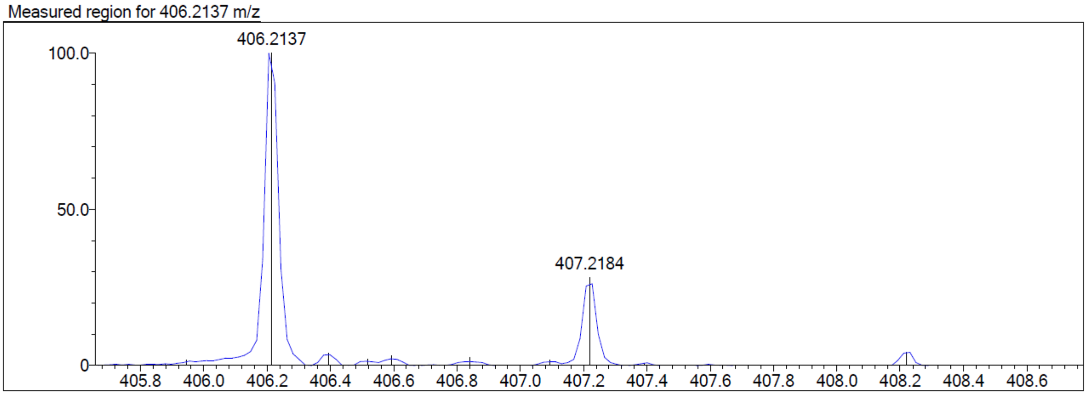

C24 H27 N3 O3 [M+H]+ : Predicted region for 406.2125 m/z

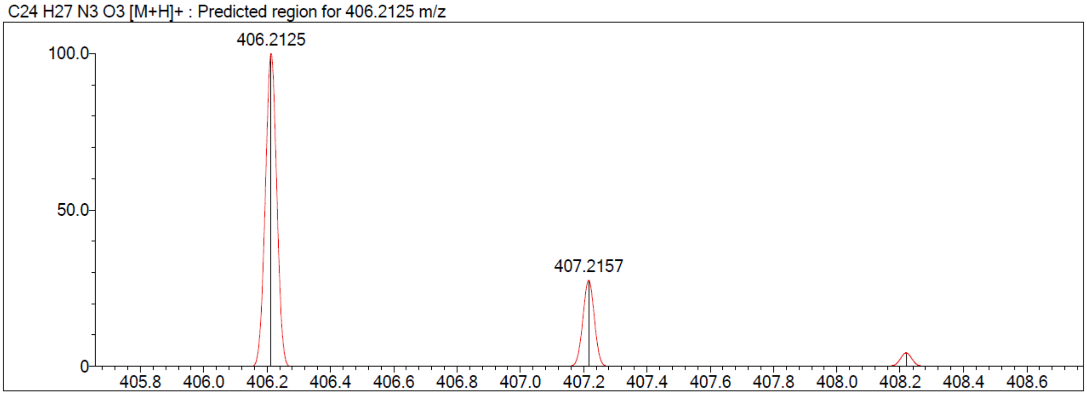

Rank	Score	Formula (M)	Ion	Meas. m/z	Pred. m/z	Df. (mDa)	Df. (ppm)	Iso	DBE
1	94.10	C24 H27 N3 O3	[M+H]+	406.2137	406.2125	1.2	2.95	98.92	13.0

Figure S56. Compound **D1** HRMS report.

*2-(4-Methylpiperazine-1-yl)-N-(4-((6-methoxy-1-oxo-2,3-dihydro-1H-inden-2-ylidene)methyl) phenyl)acetamide (**D2**)*

Brown powder. M.P.: 116.2 °C. Yield: 83%.

IR (ATR) ν_{max} (cm^{-1}): 3361 (N-H), 1683 (indanone C=O), 1662 (amide C=O), 1593-1527 (C=C), 1282 (C-N), 1188 (C-O), 825 (1,4-disubstituted benzene).

^{1}H-NMR (300 MHz, DMSO-d_6) δ (ppm): 2.17 (3H, s, CH_3), 2.38 (4H, bs, piperazine CH_2), 2.56 (4H, bs, piperazine CH_2), 3.15 (2H, s, CH_2), 3.84 (3H, s, OCH_3), 4.02 (2H, s, CH_2), 7.24 (1H, d, *J*=2.46 Hz, methoxy-1-oxo-indenylidene CH), 7.29 (1H, dd, J_1=8.31 Hz, J_2=2.55 Hz, methoxy-1-oxo-indenylidene CH), 7.48 (1H, s, C=CH), 7.58 (1H, d, *J*=8.37 Hz, methoxy-1-oxo-indenylidene CH), 7.74 (2H, d, *J*=9.06 Hz, disubstituted benzene CH), 7.79 (2H, d, *J*=8.97 Hz, disubstituted benzene CH), 9.98 (1H, s, NH).

^{13}C-NMR (75 MHz, DMSO-d_6) δ (ppm): 31.7, 46.2, 53.1, 55.0, 56.0, 62.3, 106.0, 119.9, 123.7, 127.9, 130.3, 132.2, 133.0, 134.7, 139.1, 140.6, 143.0, 159.6, 169.2, 193.6.

HRMS (ESI) (m/z) $[M+H]^+$: $C_{24}H_{27}N_3O_3$ calculated: 406.2125, found: 406.2136.

DOPNALAB

	Item	Value
	Acquired Date&Time	22.08.2019 11:08:48
	Acquired by	System Administrator
	Filename	C:\Users\dopnalab\Desktop\NURPELİN\DOKTORA TEZ\D21.ispd
	Spectrum name	D21
	Sample name	D2
	Sample ID	
	Option	
	Comment	
	No. of Scans	50
	Resolution	4 [cm-1]
	Apodization	Happ-Genzel

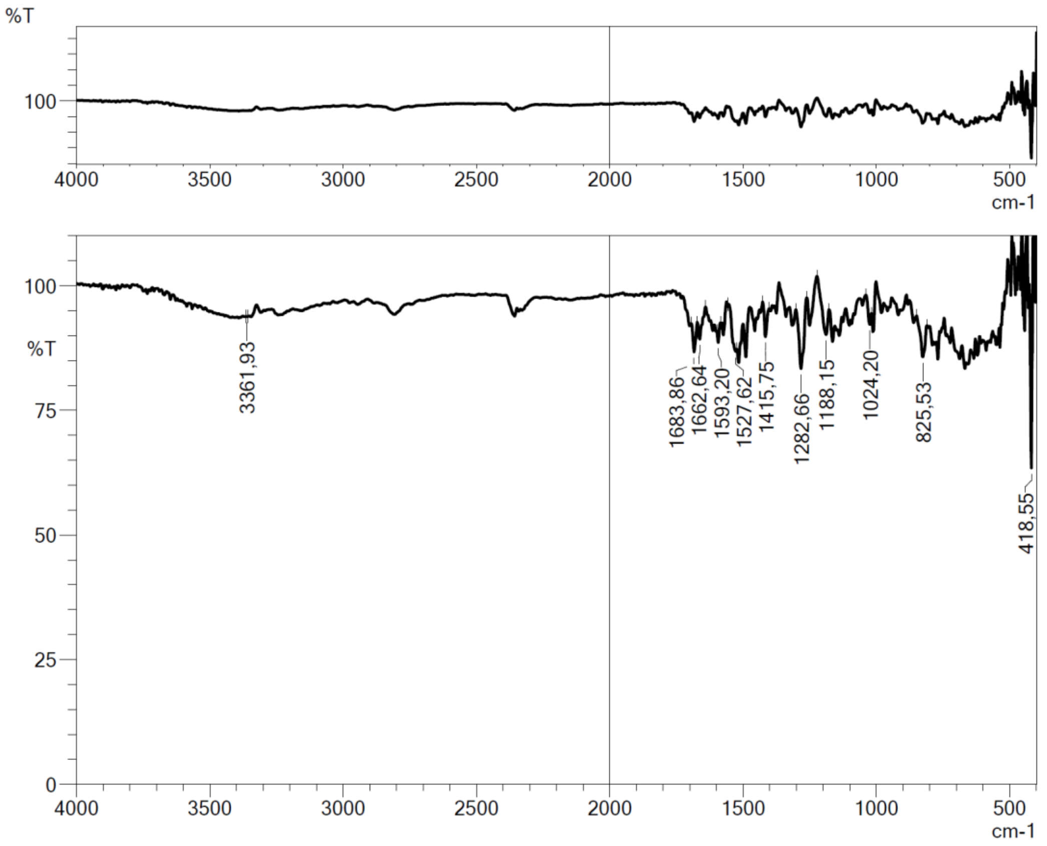

Figure S57. Compound **D2** IR report.

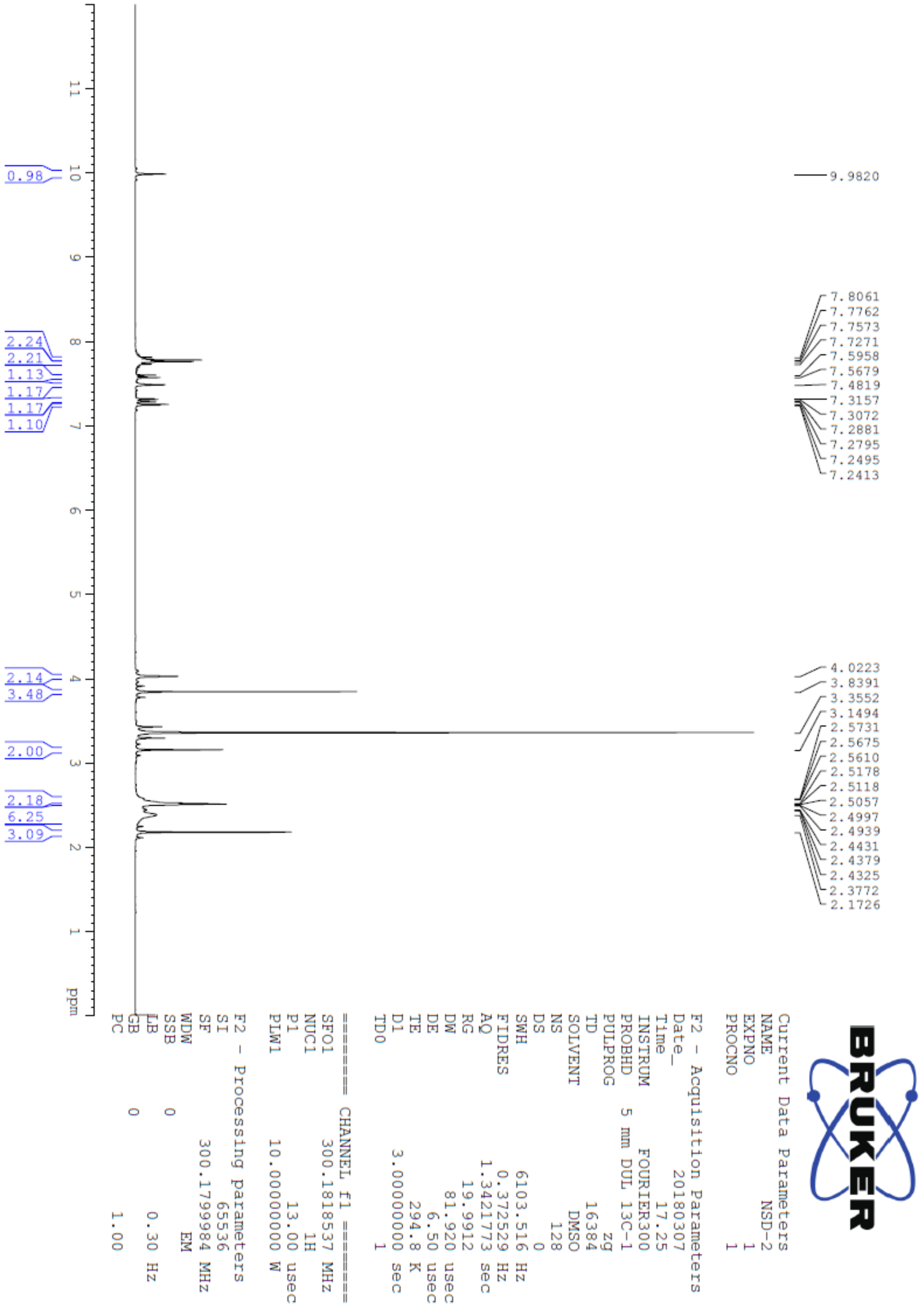

Figure S58. Compound **D2** ^{1}H-NMR spectrum.

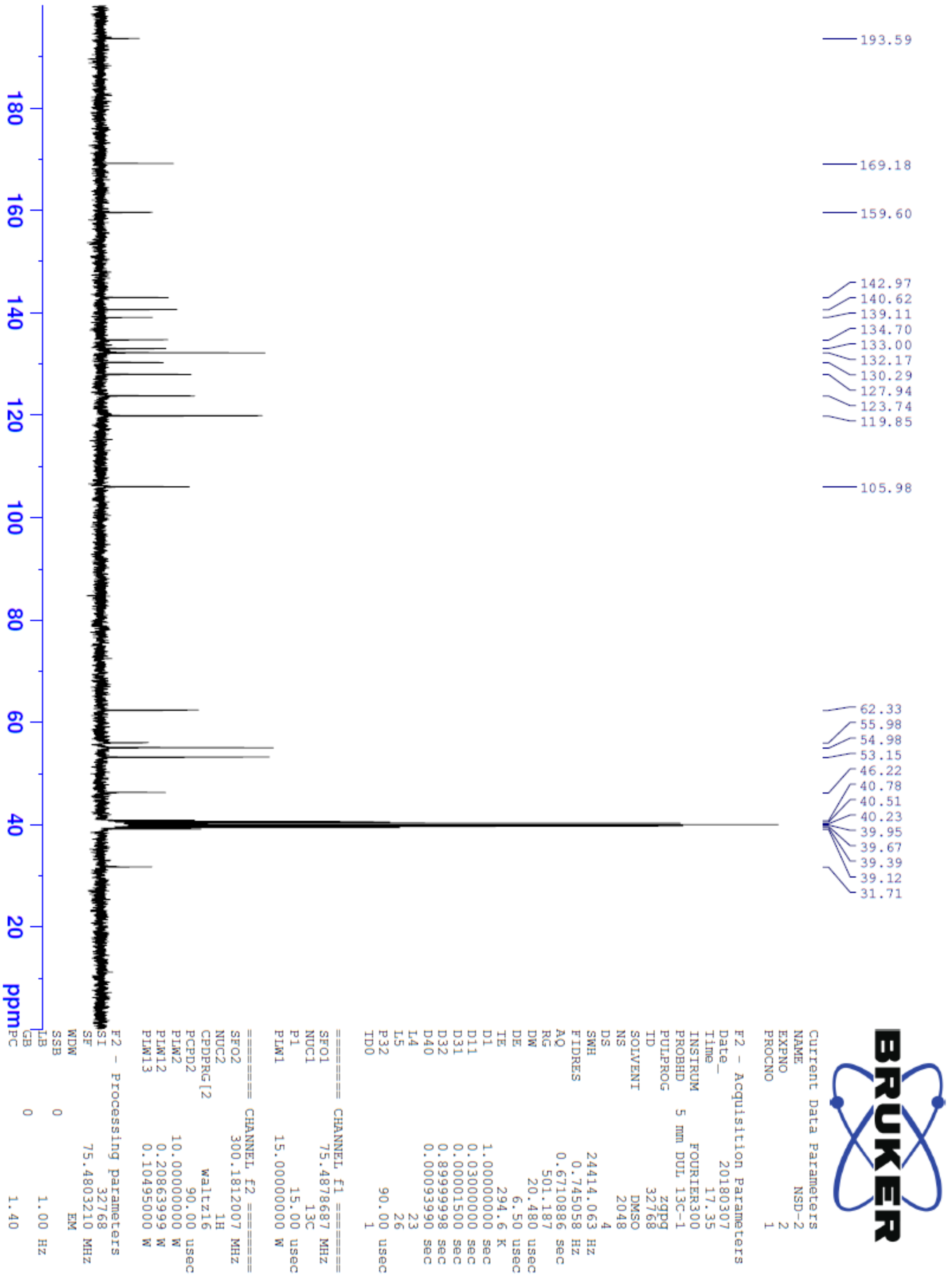

Figure S59. Compound **D2** ^{13}C-NMR spectrum.

Data File: C:\LabSolutions\Data\Analiz\Serkan\NSD-2_20.lcd

Elmt	Val.	Min	Max	Elmt	Val.	Min	Max	Elmt	Val.	Min	Max	Elmt	Val.	Min	Max	Use Adduct
H	1	5	40	O	2	0	5	S	2	0	3	Ru	2	0	0	H
C	4	0	35	F	1	0	0	Cl	1	0	2	I	3	0	0	
N	3	3	6	P	3	0	0	Br	1	0	0					

Error Margin (ppm):	5	DBE Range:	9.0 - 17.0	Electron Ions:	both
HC Ratio:	unlimited	Apply N Rule:	yes	Use MSn Info:	yes
Max Isotopes:	3	Isotope RI (%):	1.00	Isotope Res:	9000
MSn Iso RI (%):	10.00	MSn Logic Mode:	AND	Max Results:	500

Event#: 1 MS(E+) Ret. Time : 2.213 -> 2.387 Scan# : 333 -> 359

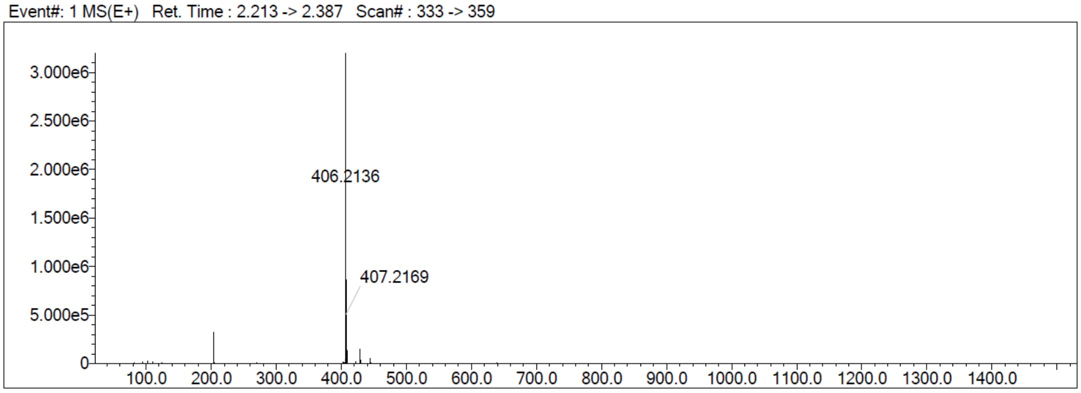

Measured region for 406.2136 m/z

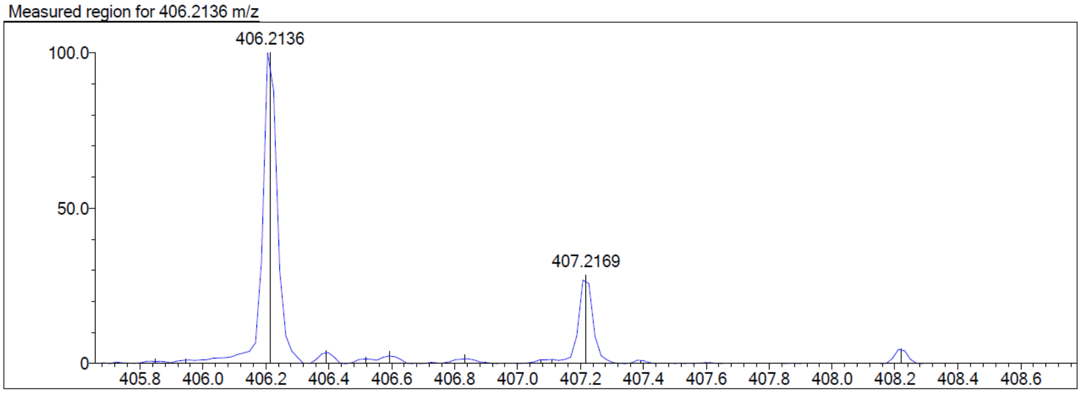

C24 H27 N3 O3 [M+H]+ : Predicted region for 406.2125 m/z

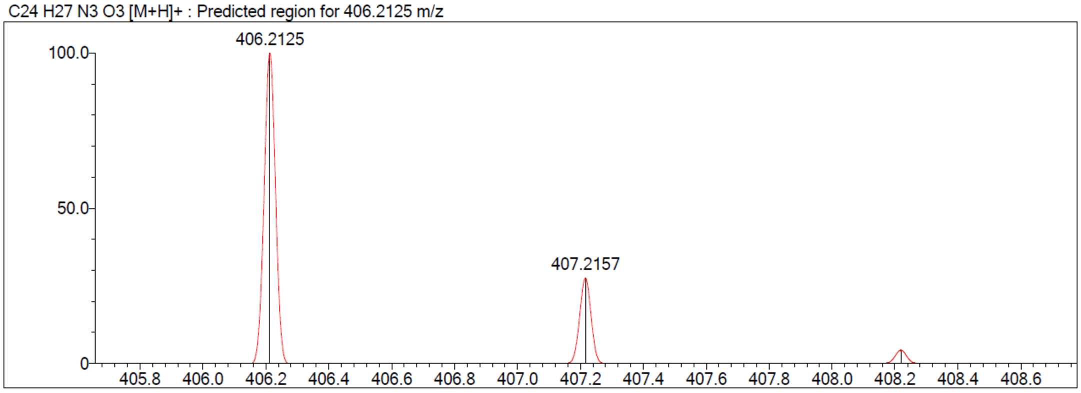

Rank	Score	Formula (M)	Ion	Meas. m/z	Pred. m/z	Df. (mDa)	Df. (ppm)	Iso	DBE
1	91.82	C24 H27 N3 O3	[M+H]+	406.2136	406.2125	1.1	2.71	95.92	13.0

Figure S60. Compound **D2** HRMS report.

*2-(4-Methylpiperazine-1-yl)-N-(4-((5,6-dimethoxy-1-oxo-2,3-dihydro-1H-inden-2-ylidene) methyl)phenyl)acetamide (**D3**)*

Dark yellow powder. M.P.: 127.6 °C. Yield: 78%.

IR (ATR) ν_{max} (cm^{-1}): 3365 (N-H), 1687 (indanone C=O), 1674 (amide C=O), 1577-1498 (C=C), 1220 (C-N), 1089 (C-O), 837 (1,4-disubstituted benzene).

^{1}H-NMR (300 MHz, DMSO-d_6) δ (ppm): 2.17 (3H, s, CH_3), 2.38 (4H, bs, piperazine CH_2), 2.52 (4H, bs, piperazine CH_2), 3.15 (2H, s, CH_2), 3.84 (3H, s, OCH_3), 3.91 (3H, s, OCH_3), 3.98 (2H, s, CH_2), 7.21 (1H, s, methoxy-1-oxo-indenylidene CH), 7.22 (1H, s, methoxy-1-oxo-indenylidene CH), 7.38 (1H, s, C=CH), 7.71 (2H, d, J=8.83 Hz, disubstituted benzene CH, methoxy-1-oxo-indenylidene CH), 7.78 (2H, d, J=8.80 Hz, disubstituted benzene CH), 9.95 (1H, s, NH).

^{13}C-NMR (75 MHz, DMSO-d_6) δ (ppm): 32.1, 46.2, 53.2, 55.0, 56.1, 56.5, 62.3, 105.0, 108.5, 119.9, 130.5, 130.6, 131.3, 131.8, 134.8, 134.8, 140.3, 145.4, 149.7, 155.6, 169.1, 192.3.

HRMS (ESI) (m/z) $[M+H]^+$: $C_{25}H_{29}N_3O_4$ calculated: 436.2231, found: 436.2251.

DOPNALAB

	Item	Value
	Acquired Date&Time	22.08.2019 11:13:25
	Acquired by	System Administrator
	Filename	C:\Users\dopnalab\Desktop\NURPELİN\DOKTORA TEZ\D31.ispd
	Spectrum name	D31
	Sample name	D3
	Sample ID	
	Option	
	Comment	
	No. of Scans	50
	Resolution	4 [cm-1]
	Apodization	Happ-Genzel

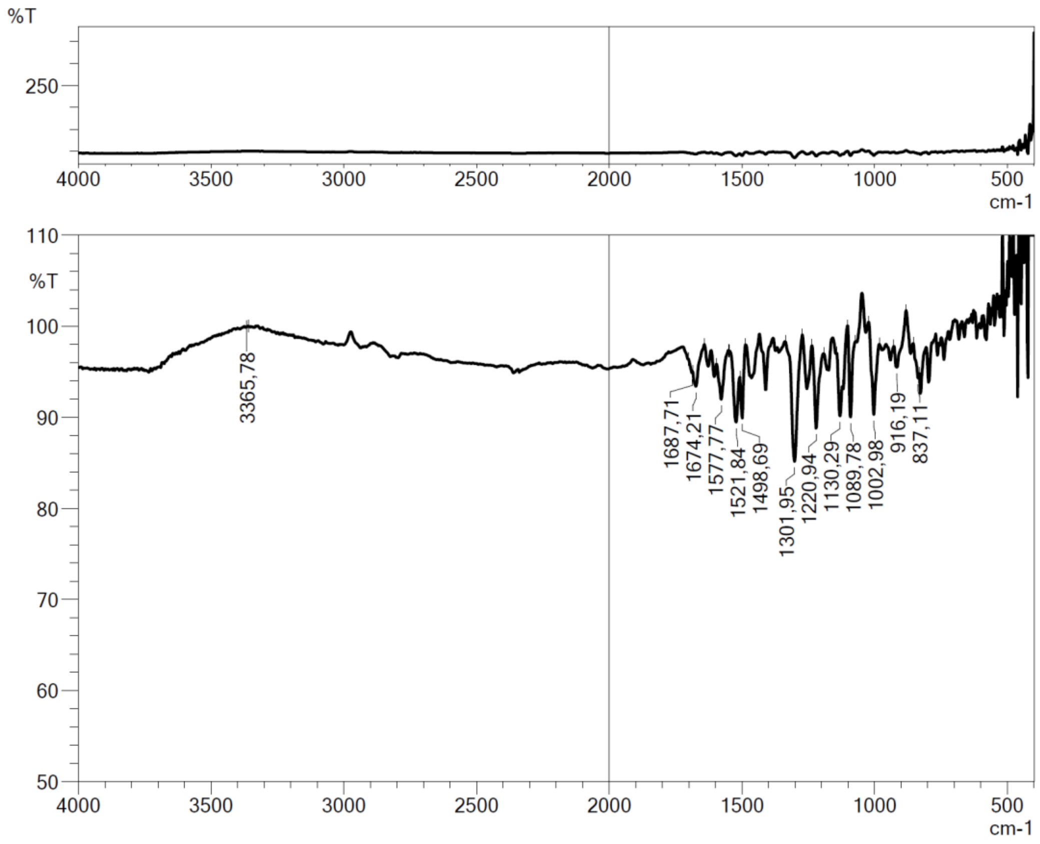

Figure S61. Compound **D3** IR report.

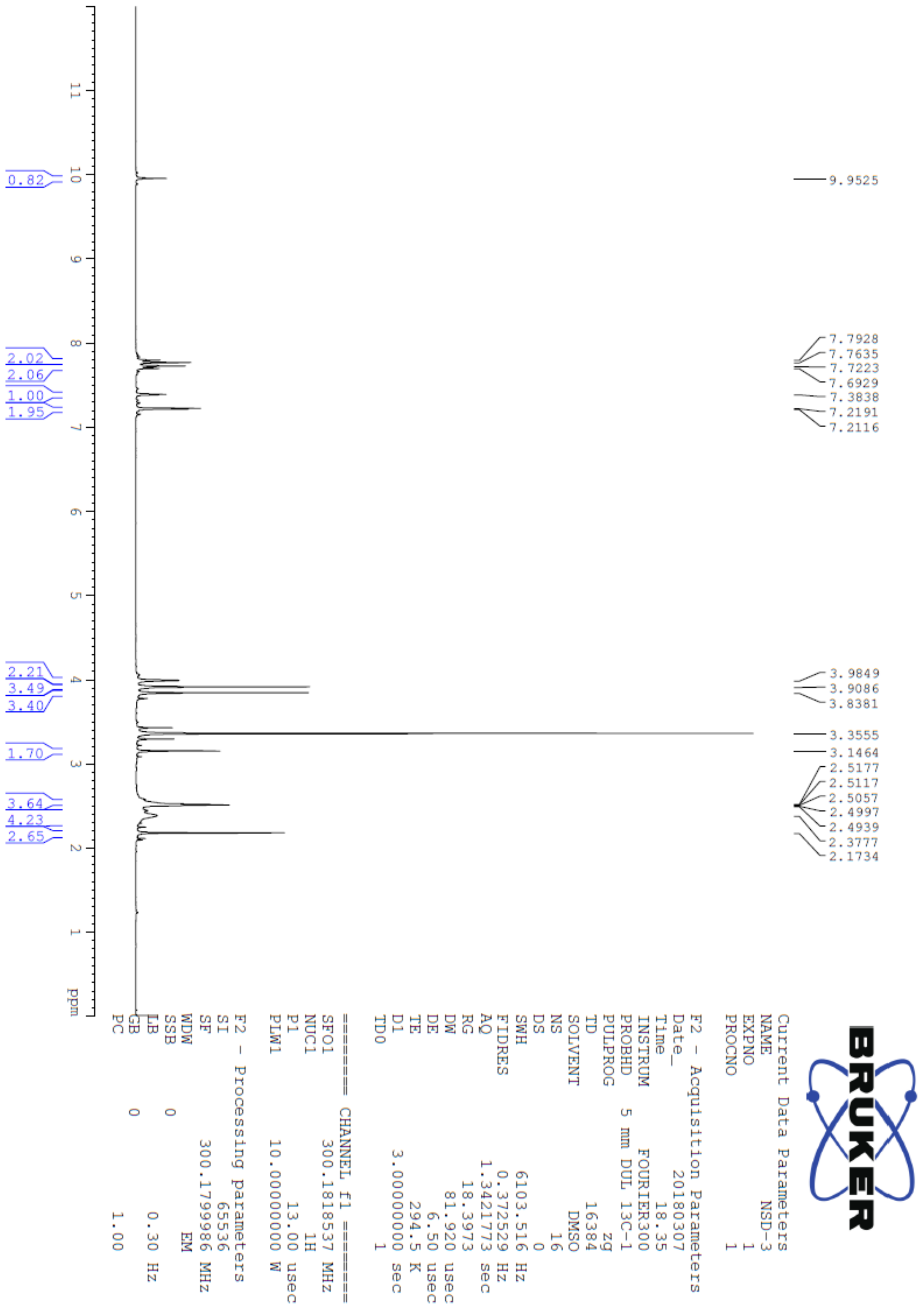

Figure S62. Compound **D3** ^{1}H-NMR spectrum.

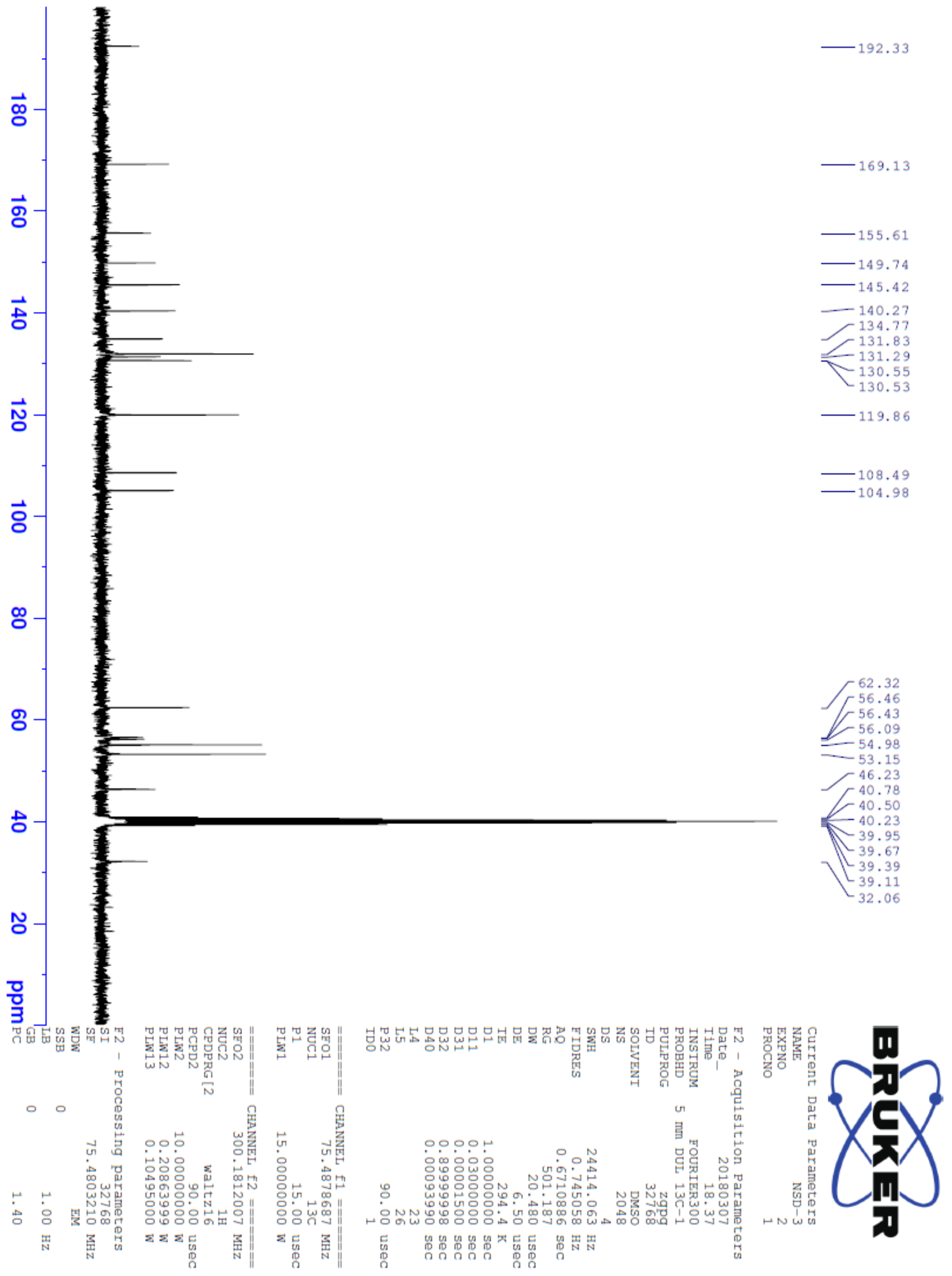

Figure S63. Compound **D3** ^{13}C-NMR spectrum.

Data File: C:\LabSolutions\Data\Analiz\Serkan\NSD-3_21.lcd

Elmt	Val.	Min	Max	Elmt	Val.	Min	Max	Elmt	Val.	Min	Max	Elmt	Val.	Min	Max	Use Adduct
H	1	5	40	O	2	3	5	S	2	0	3	Ru	2	0	0	H
C	4	0	35	F	1	0	0	Cl	1	0	2	I	3	0	0	
N	3	3	6	P	3	0	0	Br	1	0	0					

Error Margin (ppm):	5	DBE Range:	9.0 - 17.0	Electron Ions:	both
HC Ratio:	unlimited	Apply N Rule:	yes	Use MSn Info:	yes
Max Isotopes:	3	Isotope RI (%):	1.00	Isotope Res:	9000
MSn Iso RI (%):	10.00	MSn Logic Mode:	AND	Max Results:	500

Event#: 1 MS(E+) Ret. Time : 2.093 -> 2.227 Scan# : 315 -> 335

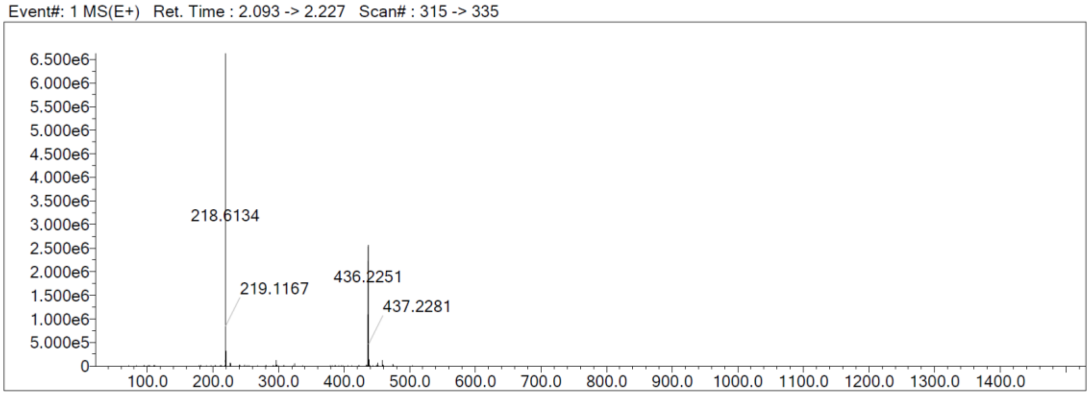

Measured region for 436.2251 m/z

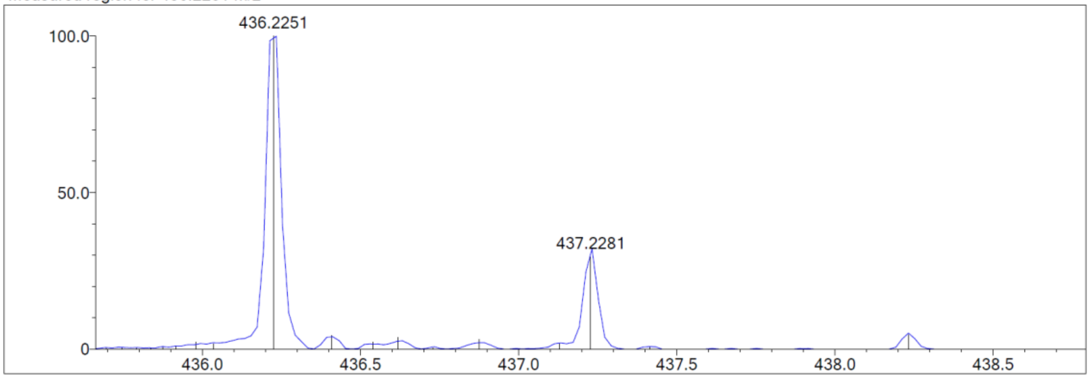

C25 H29 N3 O4 [M+H]+ : Predicted region for 436.2231 m/z

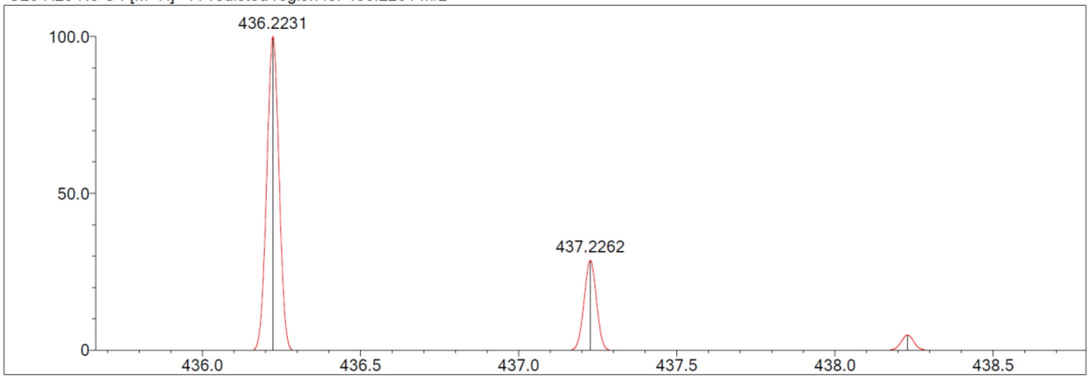

Rank	Score	Formula (M)	Ion	Meas. m/z	Pred. m/z	Df. (mDa)	Df. (ppm)	Iso	DBE
1	91.05	C25 H29 N3 O4	[M+H]+	436.2251	436.2231	2.0	4.58	100.00	13.0

Figure S64. Compound **D3** HRMS report.

*2-(4-Ethylpiperazine-1-yl)-N-(4-((5-methoxy-1-oxo-2,3-dihydro-1H-inden-2-ylidene)methyl) phenyl)acetamide (**D4**)*

Brown powder. M.P.: 205.6 °C. Yield: 79%.

IR (ATR) ν_{max} (cm^{-1}): 3253 (N-H), 1685 (indanone C=O), 1604 (amide C=O), 1570-1500 (C=C), 1172 (C-N), 1087 (C-O), 864 (1,4-disubstituted benzene).

^{1}H-NMR (300 MHz, DMSO-d_6) δ (ppm): 0.98 (3H, t, *J*=7.14 Hz, CH_3), 2.32 (3H, q, *J*=7.18 Hz, CH_2), 2.43 (4H, bs, piperazine CH_2), 2.52 (4H, bs, piperazine CH_2), 3.14 (2H, s, CH_2), 3.89 (3H, s, OCH_3), 4.04 (2H, s, CH_2), 7.02 (1H, dd, J_1=8.49 Hz, J_2=2.25 Hz, methoxy-1-oxo-indenylidene CH), 7.17 (1H, d, *J*=2.01 Hz, methoxy-1-oxo-indenylidene CH), 7.40 (1H, s, C=CH), 7.70-7.72 (3H, m, disubstituted benzene CH, methoxy-1-oxo-indenylidene CH), 7.78 (2H, d, *J*=8.76 Hz, disubstituted benzene CH), 9.95 (1H, s, NH).

^{13}C-NMR (75 MHz, DMSO-d_6) δ (ppm): 12.5, 32.5, 52.1, 52.7, 53.3, 56.3, 62.4, 110.6, 115.8, 119.9, 125.8, 130.5, 131.1, 131.7, 131.9, 134.5, 140.4, 153.3, 165.3, 169.1, 192.0.

HRMS (ESI) (m/z) [M+H]$^+$: $C_{25}H_{29}N_3O_3$ calculated: 420.2282, found: 420.2298.

DOPNALAB

	Item	Value
	Acquired Date&Time	22.08.2019 11:16:27
	Acquired by	System Administrator
	Filename	C:\Users\dopnalab\Desktop\NURPELİN\DOKTORA TEZ\D41.ispd
	Spectrum name	D41
	Sample name	D4
	Sample ID	
	Option	
	Comment	
	No. of Scans	50
	Resolution	4 [cm-1]
	Apodization	Happ-Genzel

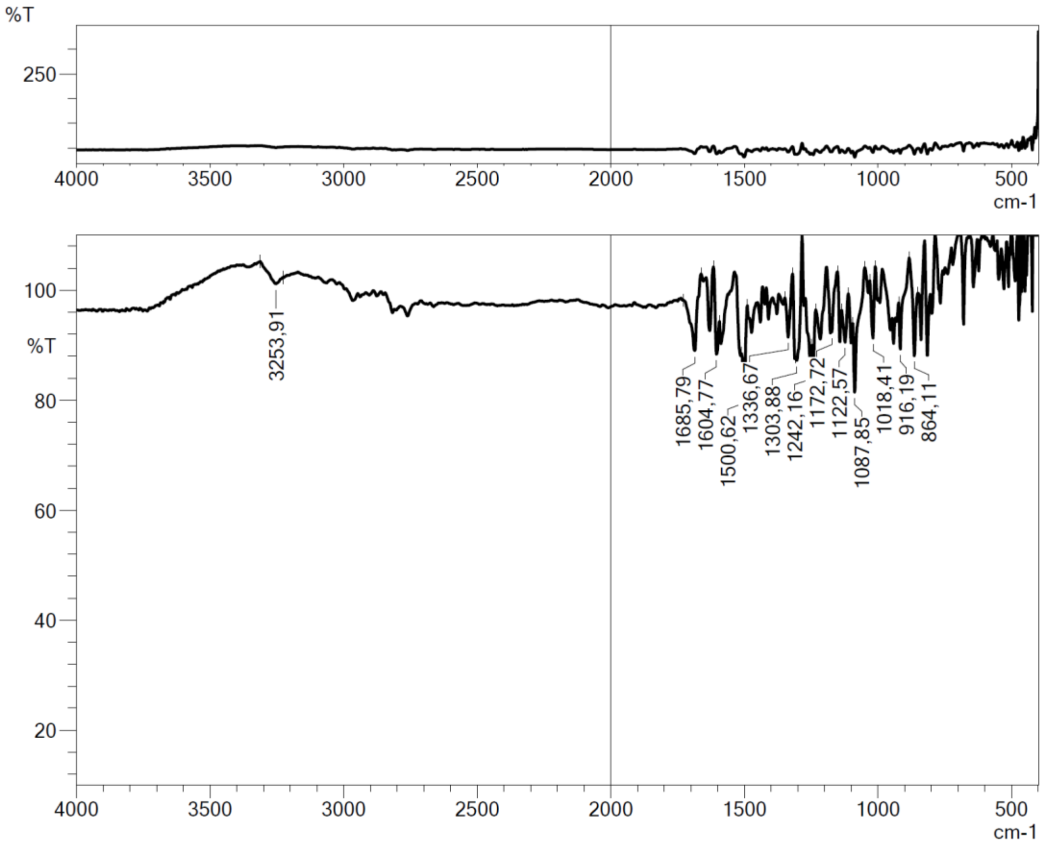

Figure S65. Compound **D4** IR report.

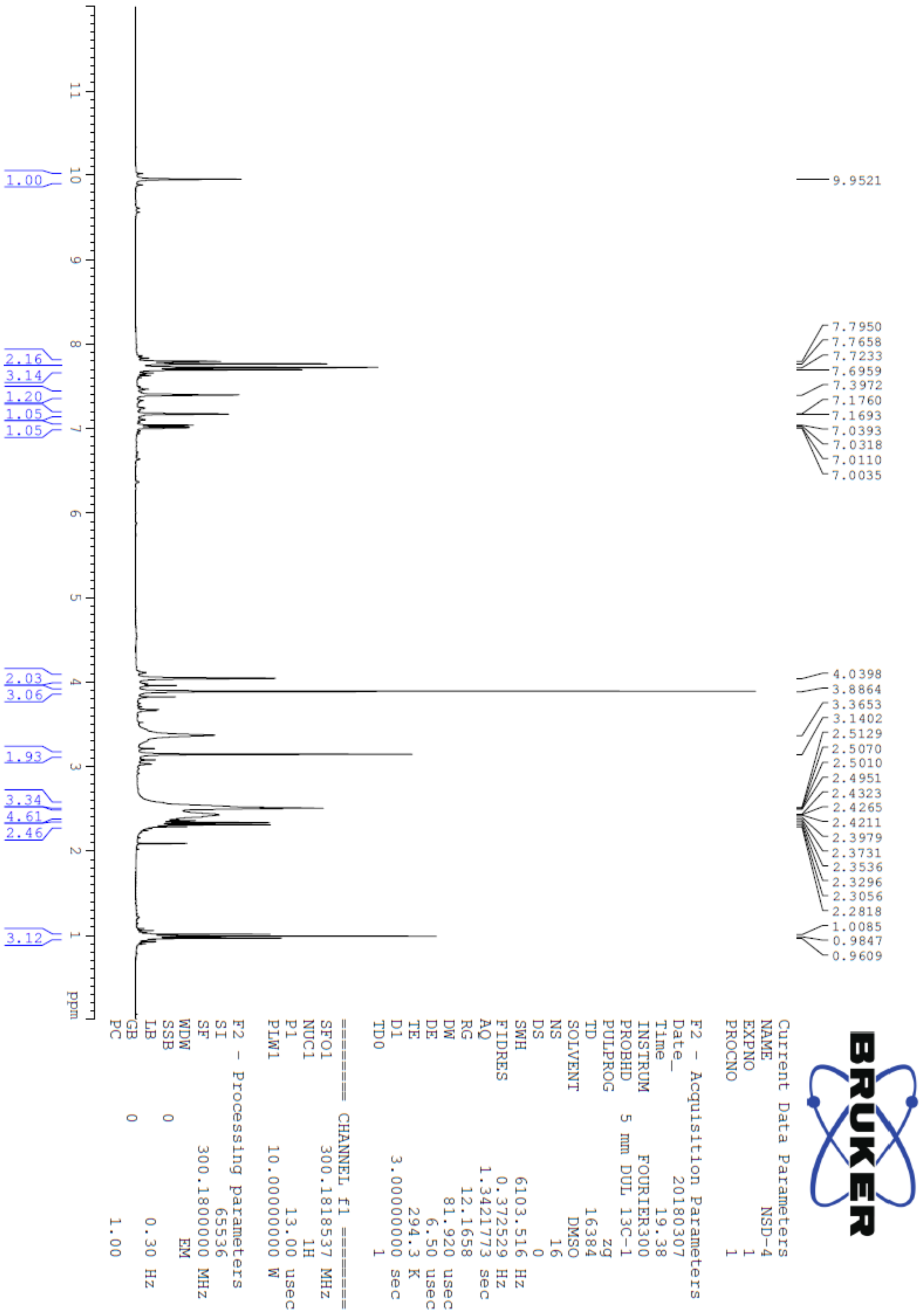

Figure S66. Compound **D4** ^{1}H-NMR spectrum.

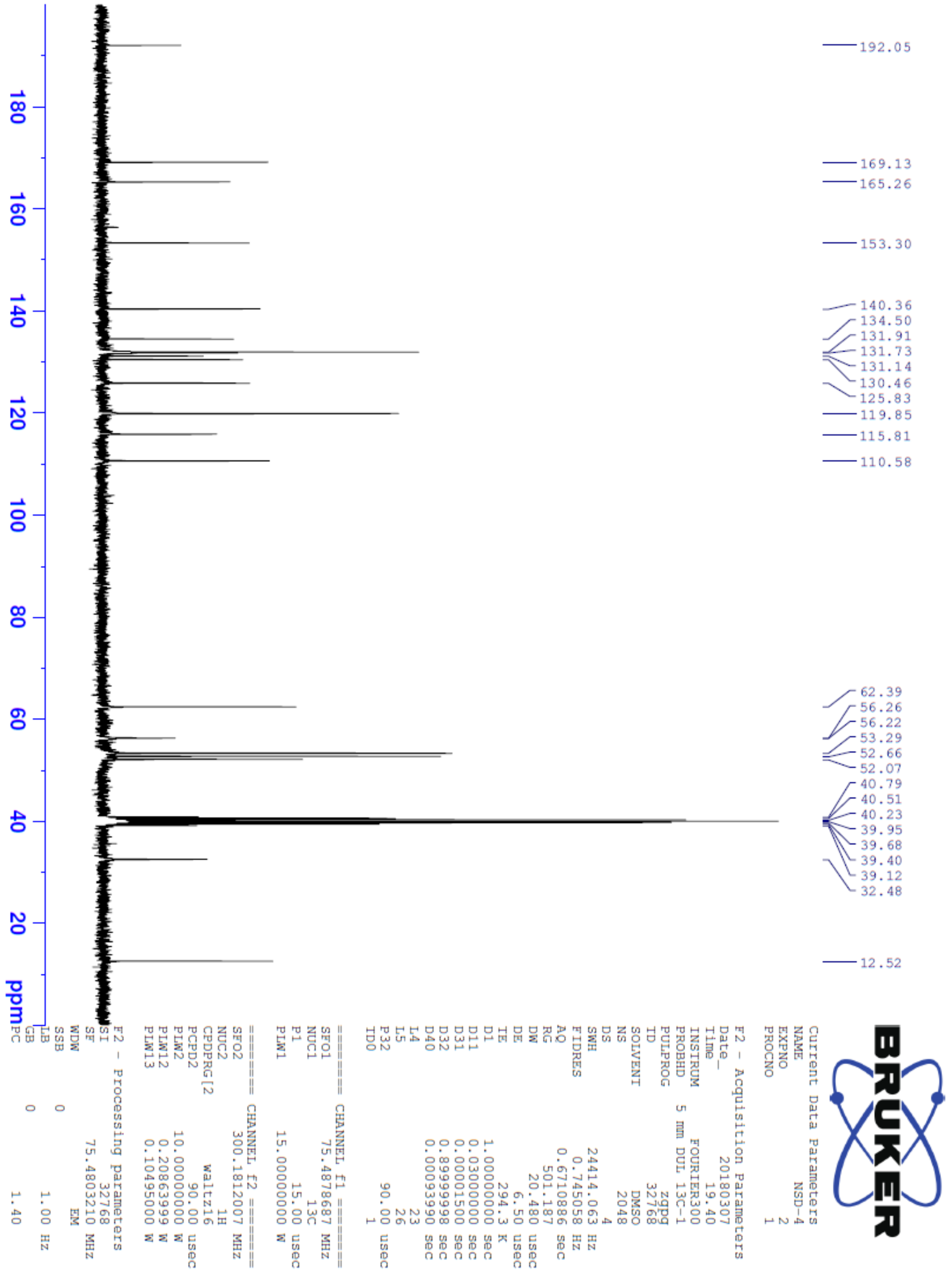

Figure S67. Compound **D4** ^{13}C-NMR spectrum.

Data File: C:\LabSolutions\Data\Analiz\Serkan\NSD-4_22.lcd

Elmt	Val.	Min	Max	Elmt	Val.	Min	Max	Elmt	Val.	Min	Max	Elmt	Val.	Min	Max	Use Adduct
H	1	5	40	O	2	3	5	S	2	0	3	Ru	2	0	0	H
C	4	0	35	F	1	0	0	Cl	1	0	2	I	3	0	0	
N	3	3	6	P	3	0	0	Br	1	0	0					

Error Margin (ppm): 5
HC Ratio: unlimited
Max Isotopes: 3
MSn Iso RI (%): 10.00

DBE Range: 9.0 - 17.0
Apply N Rule: yes
Isotope RI (%): 1.00
MSn Logic Mode: AND

Electron Ions: both
Use MSn Info: yes
Isotope Res: 9000
Max Results: 500

Event#: 1 MS(E+) Ret. Time : 2.173 Scan# : 327

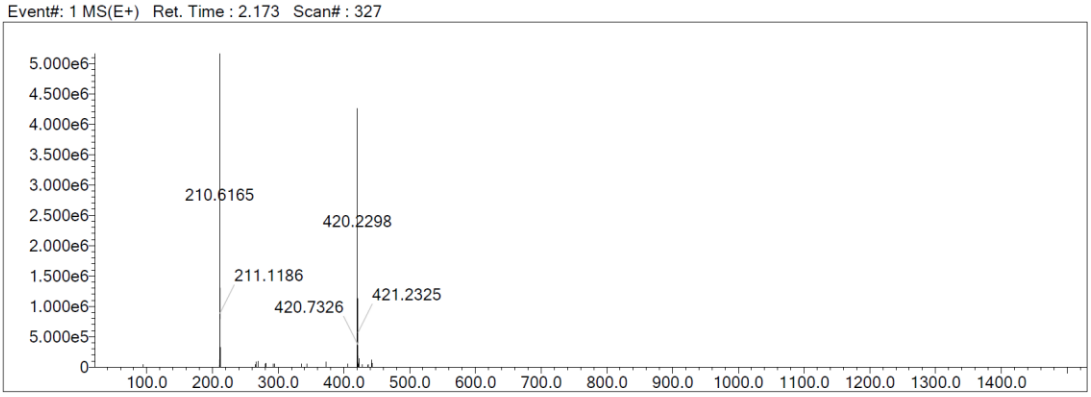

Measured region for 420.2298 m/z

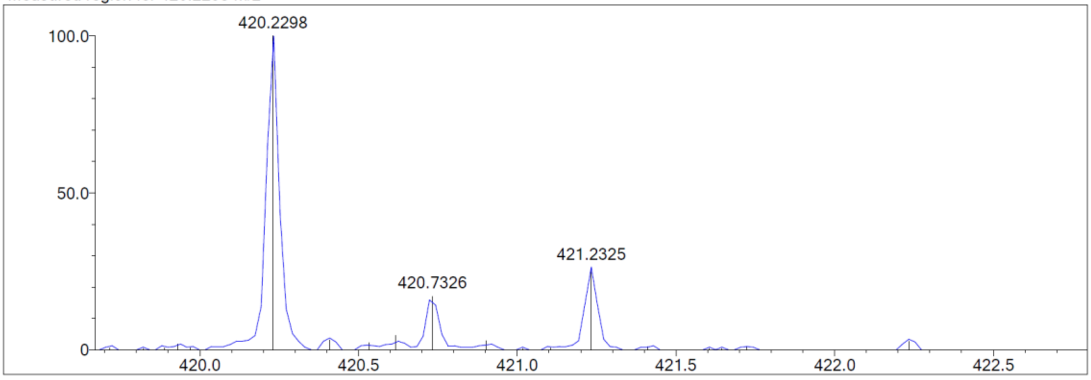

C25 H29 N3 O3 [M+H]+ : Predicted region for 420.2282 m/z

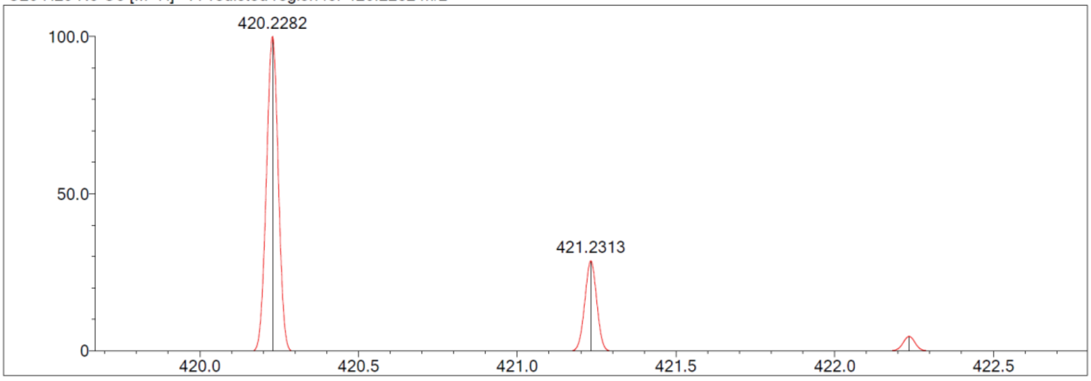

Rank	Score	Formula (M)	Ion	Meas. m/z	Pred. m/z	Df. (mDa)	Df. (ppm)	Iso	DBE
1	75.97	C25 H29 N3 O3	[M+H]+	420.2298	420.2282	1.6	3.81	81.71	13.0

Figure S68. Compound **D4** HRMS report.

*2-(4-Ethylpiperazine-1-yl)-N-(4-((6-methoxy-1-oxo-2,3-dihydro-1H-inden-2-ylidene)methyl) phenyl)acetamide (**D5**)*

Dark beige powder. M.P.: 191.2 °C. Yield: 77%.

IR (ATR) ν_{max} (cm^{-1}): 3329 (N-H), 1699 (indanone C=O), 1672 (amide C=O), 1579-1539 (C=C), 1222 (C-N), 1020 (C-O), 839 (1,4-disubstituted benzene).

^{1}H-NMR (300 MHz, DMSO-*d*$_6$) δ (ppm): 0.99 (3H, t, *J*=7.20 Hz, CH_3), 2.31 (3H, q, *J*=7.20 Hz, CH_2), 2.42 (4H, bs, piperazine CH_2), 2.52 (4H, bs, piperazine CH_2), 3.14 (2H, s, CH_2), 3.83 (3H, s, OCH_3), 4.01 (2H, s, CH_2), 7.24 (1H, d, *J*=2.46 Hz, methoxy-1-oxo-indenylidene CH), 7.29 (1H, dd, J_1=8.31 Hz, J_2=2.55 Hz, methoxy-1-oxo-indenylidene CH), 7.47 (1H, s, C=CH), 7.57 (1H, d, *J*=2.37 Hz, methoxy-1-oxo-indenylidene CH), 7.73 (2H, d, *J*=9.03 Hz, disubstituted benzene CH), 7.78 (2H, d, *J*=8.94 Hz, disubstituted benzene CH), 9.97 (1H, s, NH).

^{13}C-NMR (75 MHz, DMSO-*d*$_6$) δ (ppm): 12.5, 31.7, 52.1, 52.7, 53.3, 55.9, 62.4, 106.0, 119.8, 123.7, 127.9, 130.3, 132.2, 133.0, 134.7, 139.1, 140.6, 143.0, 160.0, 169.2, 193.6.

HRMS (ESI) (m/z) [M+H]$^+$: $C_{25}H_{29}N_3O_3$ calculated: 420.2282, found: 420.2299.

DOPNALAB

	Item	Value
	Acquired Date&Time	22.08.2019 11:19:11
	Acquired by	System Administrator
	Filename	C:\Users\dopnalab\Desktop\NURPELİN\DOKTORA TEZ\D51.ispd
	Spectrum name	D51
	Sample name	D5
	Sample ID	
	Option	
	Comment	
	No. of Scans	50
	Resolution	4 [cm-1]
	Apodization	Happ-Genzel

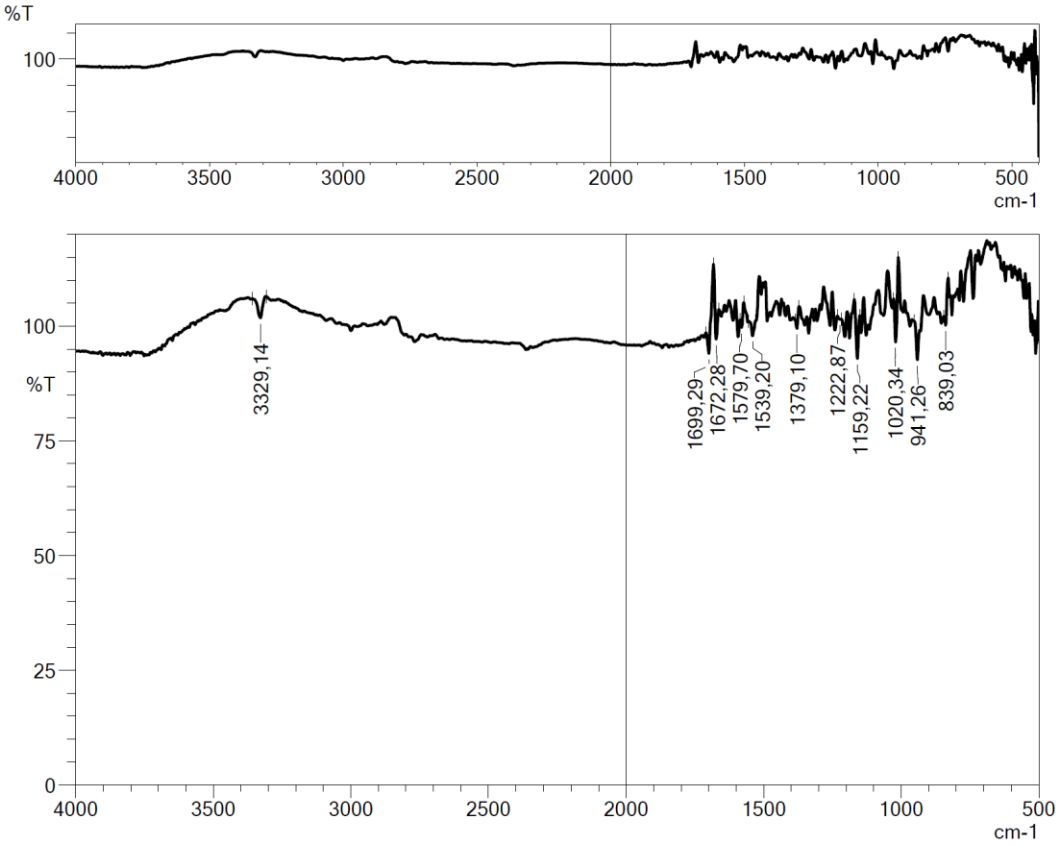

Figure S69. Compound **D5** IR report.

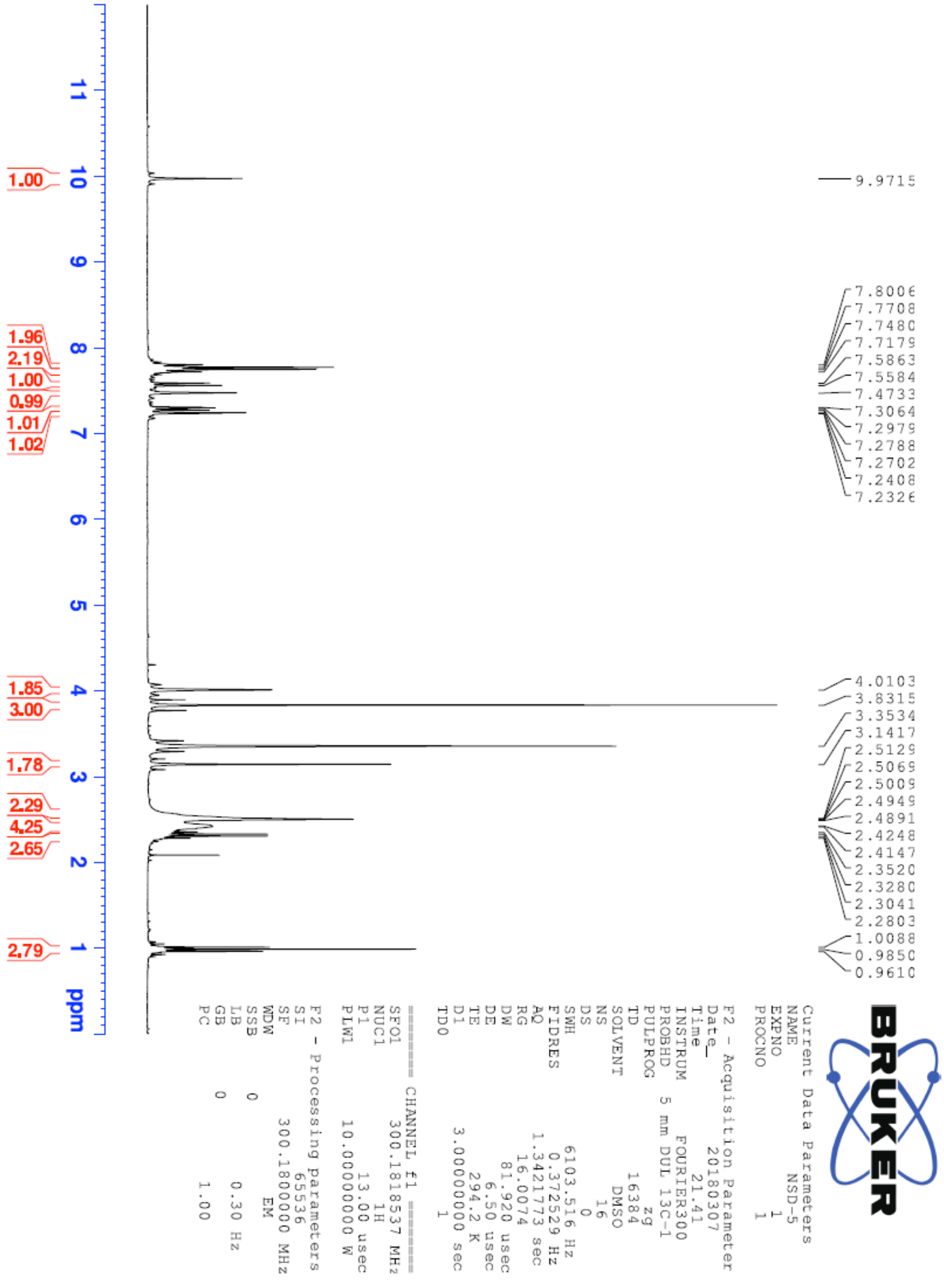

Figure S70. Compound **D5** ^{1}H-NMR spectrum.

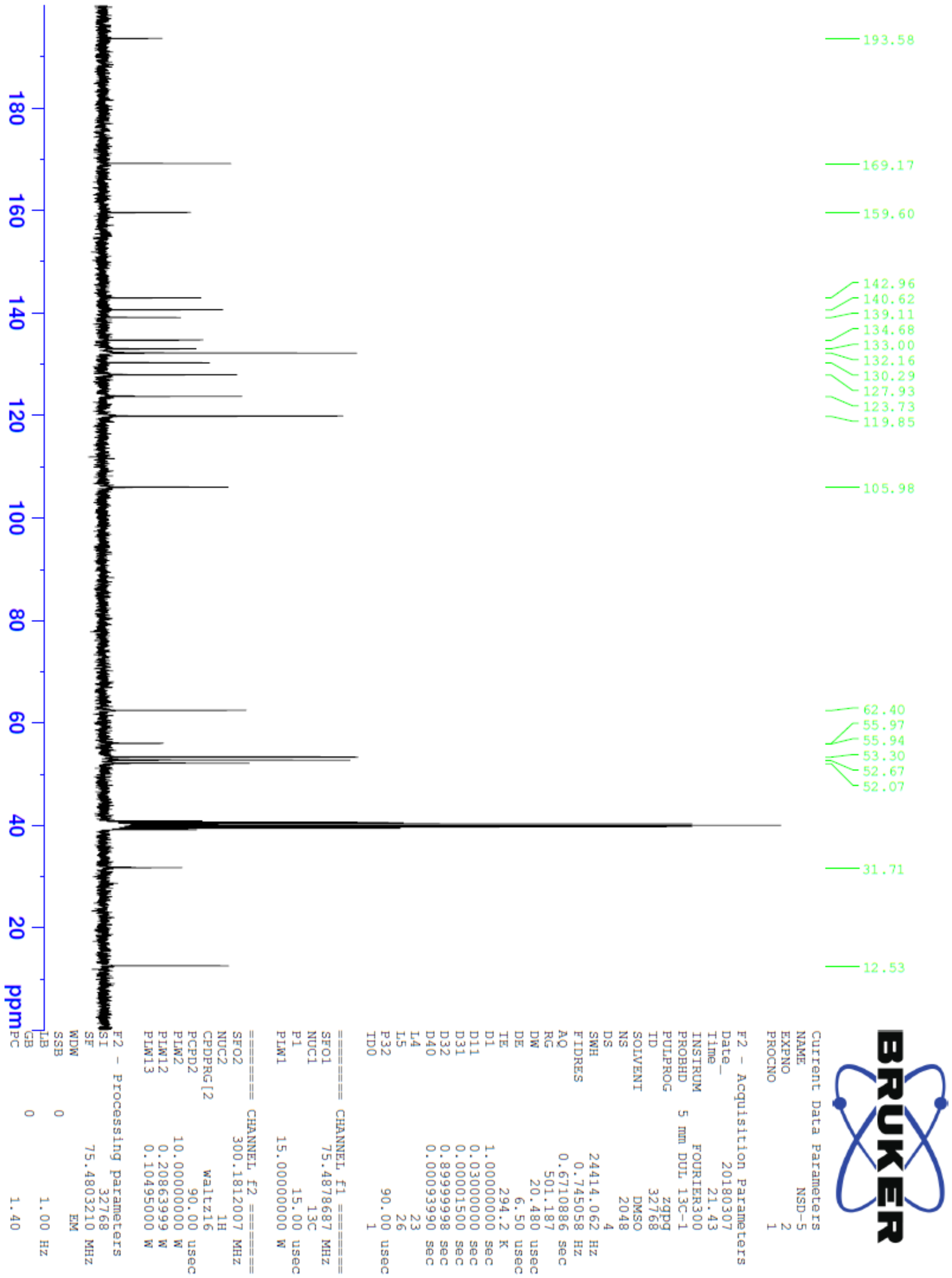

Figure S71. Compound **D5** ^{13}C-NMR spectrum.

Formula Predictor Report - NSD-5_23.lcd　　Page 1 of 1

Data File: C:\LabSolutions\Data\Analiz\Serkan\NSD-5_23.lcd

Elmt	Val.	Min	Max	Elmt	Val.	Min	Max	Elmt	Val.	Min	Max	Elmt	Val.	Min	Max	Use Adduct
H	1	5	40	O	2	3	5	S	2	0	3	Ru	2	0	0	H
C	4	0	35	F	1	0	0	Cl	1	0	2	I	3	0	0	
N	3	3	6	P	3	0	0	Br	1	0	0					

Error Margin (ppm):	5	DBE Range:	9.0 - 17.0	Electron Ions:	both
HC Ratio:	unlimited	Apply N Rule:	yes	Use MSn Info:	yes
Max Isotopes:	3	Isotope RI (%):	1.00	Isotope Res:	9000
MSn Iso RI (%):	10.00	MSn Logic Mode:	AND	Max Results:	500

Event#: 1 MS(E+)　Ret. Time : 2.200 -> 2.387　Scan# : 331 -> 359

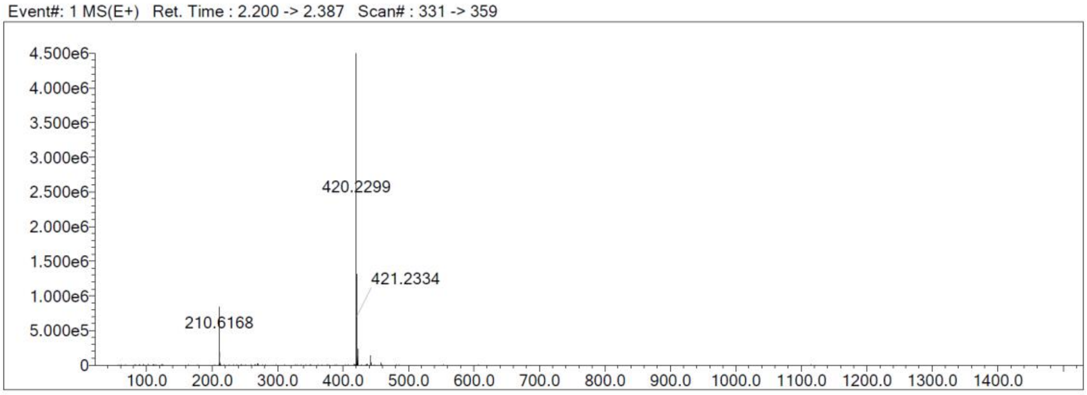

Measured region for 420.2299 m/z

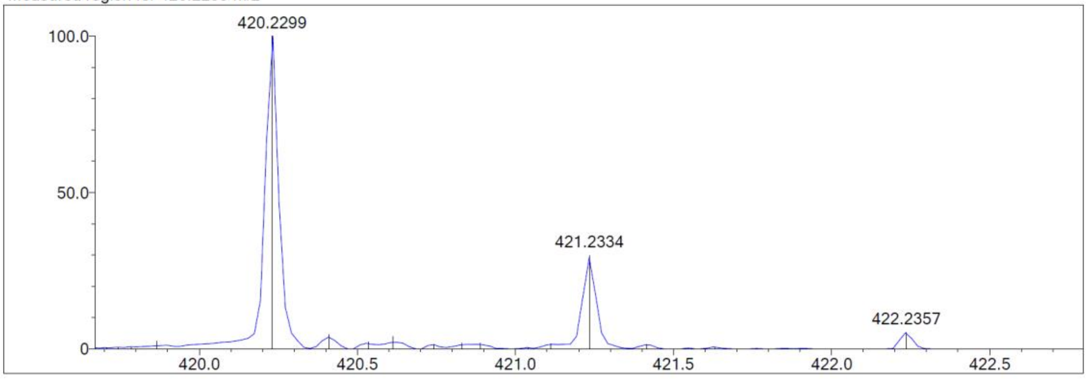

C25 H29 N3 O3 [M+H]+ : Predicted region for 420.2282 m/z

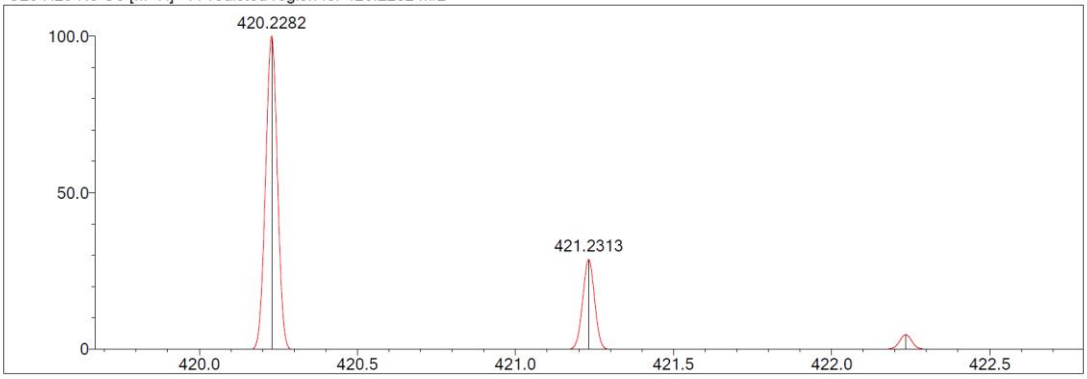

Rank	Score	Formula (M)	Ion	Meas. m/z	Pred. m/z	Df. (mDa)	Df. (ppm)	Iso	DBE
1	78.75	C25 H29 N3 O3	[M+H]+	420.2299	420.2282	1.7	4.05	85.25	13.0

Figure S72. Compound **D5** HRMS report.

*2-(4-Ethylpiperazine-1-yl)-N-(4-((5,6-dimethoxy-1-oxo-2,3-dihydro-1H-inden-2-ylidene) methyl)phenyl)acetamide (**D6**)*

Dark brown powder. M.P.: 133.1 °C. Yield: 76%.

IR (ATR) ν_{max} (cm^{-1}): 3365 (N-H), 1701 (indanone C=O), 1670 (amide C=O), 1635-1471 (C=C), 1220 (C-N), 1128 (C-O), 848 (1,4-disubstituted benzene).

^{1}H-NMR (300 MHz, DMSO-d_6) δ (ppm): 0.98 (3H, t, J=7.19 Hz, CH_3), 2.31 (3H, q, J=7.15 Hz, CH_2), 2.42 (4H, bs, piperazine CH_2), 2.52 (4H, bs, piperazine CH_2), 3.14 (2H, s, CH_2), 3.83 (3H, s, OCH_3), 3.90 (3H, s, OCH_3), 3.97 (2H, s, CH_2), 7.20 (1H, s, methoxy-1-oxo-indenylidene CH), 7.21 (1H, s, methoxy-1-oxo-indenylidene CH), 7.37 (1H, s, C=CH), 7.70 (2H, d, J=8.88 Hz, disubstituted benzene CH), 7.77 (2H, d, J=8.78 Hz, disubstituted benzene CH), 9.94 (1H, s, NH).

^{13}C-NMR (75 MHz, DMSO-d_6) δ (ppm): 12.5, 32.1, 52.1, 52.7, 53.3, 56.1, 56.4, 62.4, 105.0, 108.5, 119.9, 130.5, 130.6, 131.3, 131.8, 134.8, 145.7, 149.7, 155.6, 169.1, 192.3.

HRMS (ESI) (m/z) $[M+H]^+$: $C_{26}H_{31}N_3O_4$ calculated: 450.2387, found: 450.2409.

DOPNALAB

	Item	Value
	Acquired Date&Time	22.08.2019 11:21:48
	Acquired by	System Administrator
	Filename	C:\Users\dopnalab\Desktop\NURPELİN\DOKTORA TEZ\D61.ispd
	Spectrum name	D61
	Sample name	D6
	Sample ID	
	Option	
	Comment	
	No. of Scans	50
	Resolution	4 [cm-1]
	Apodization	Happ-Genzel

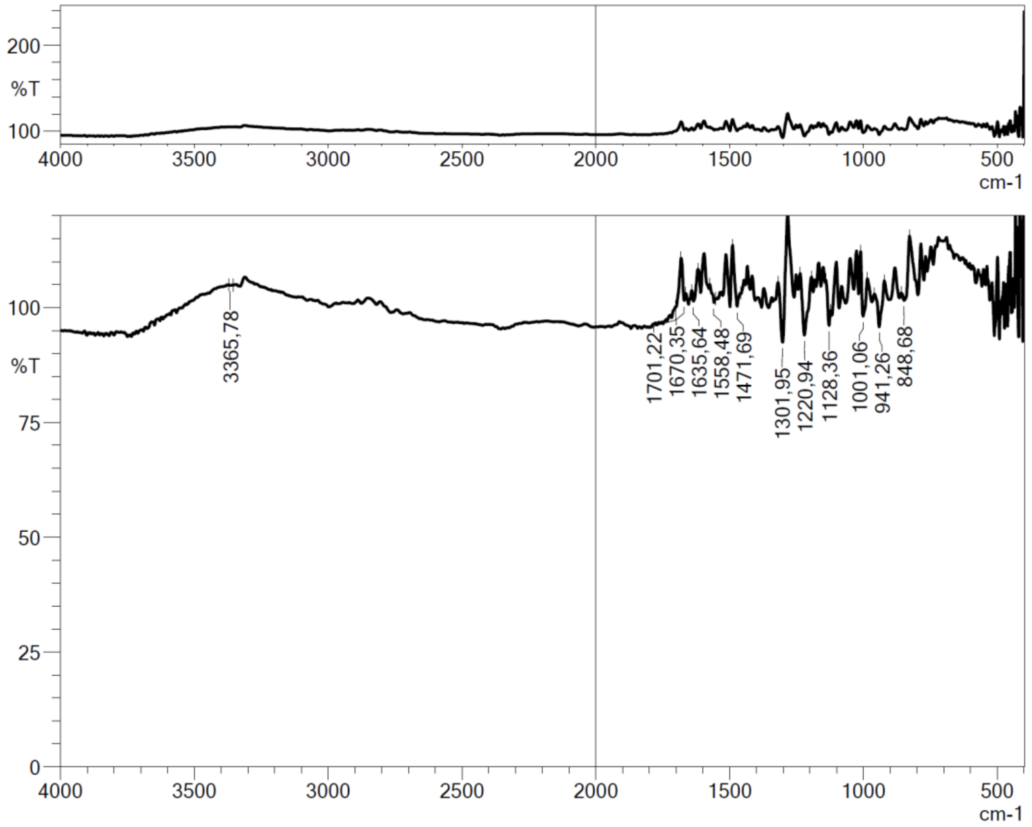

Figure S73. Compound **D6** IR report.

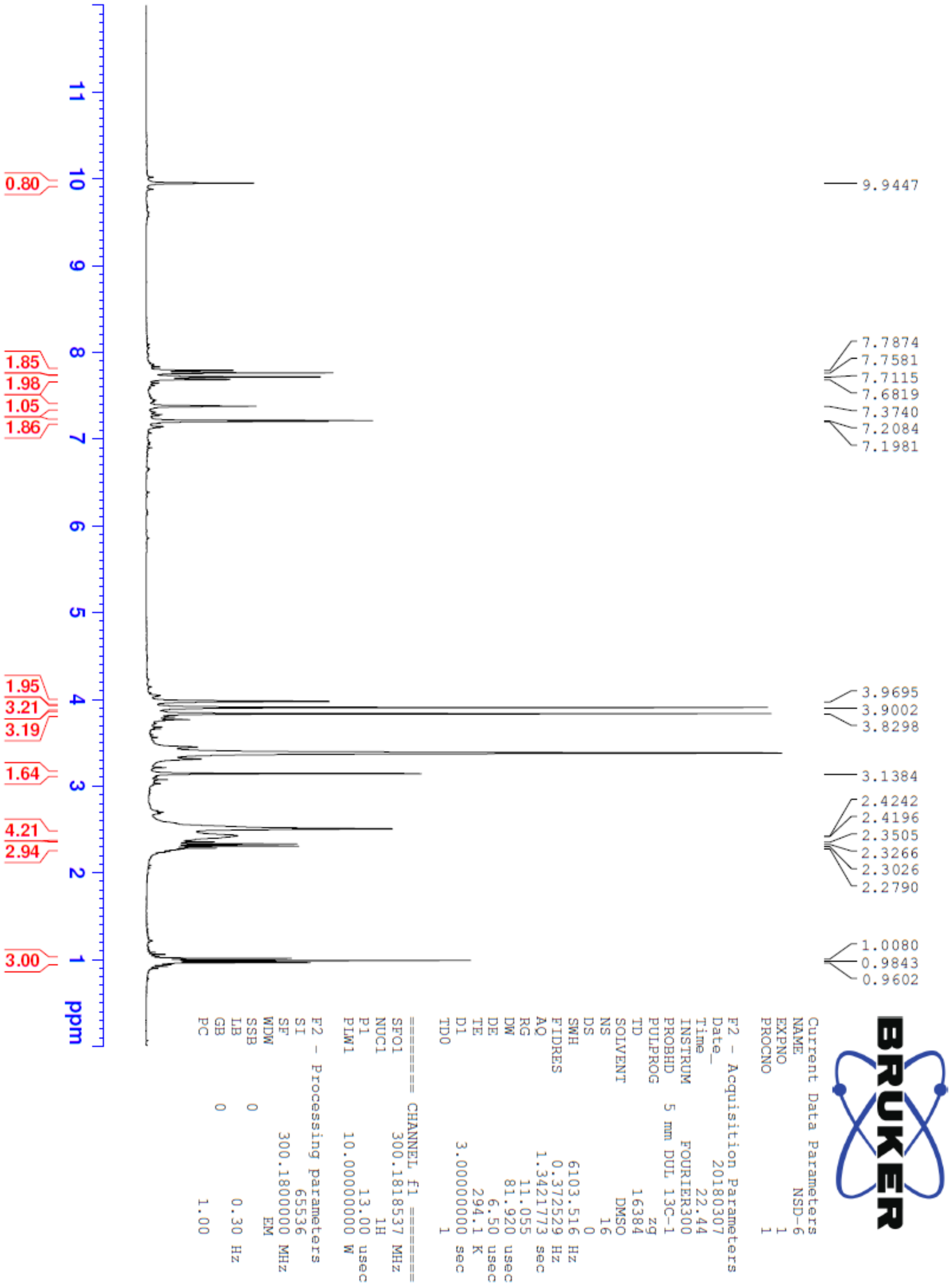

Figure S74. Compound **D6** ^{1}H-NMR spectrum.

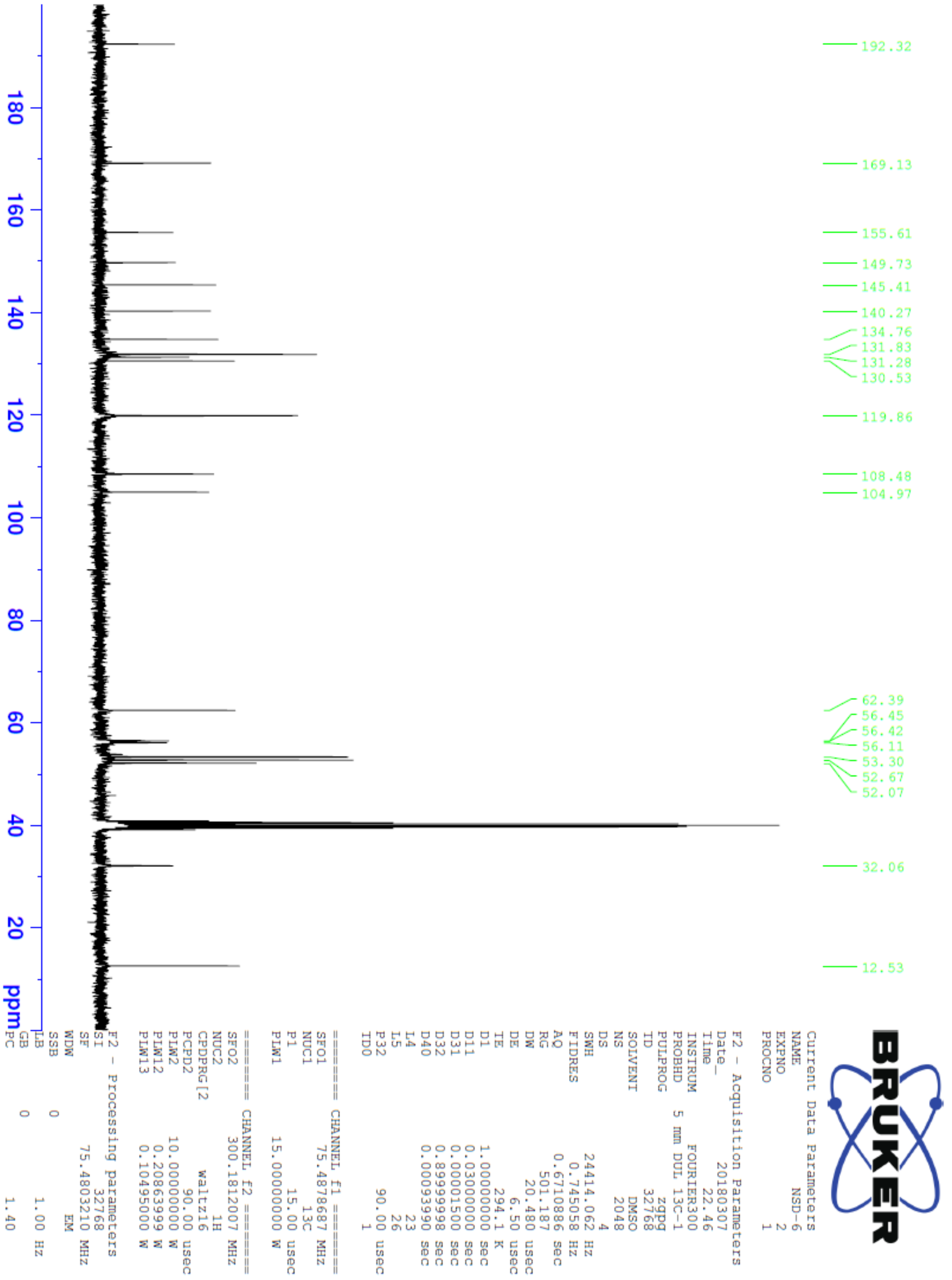

Figure S75. Compound **D6** ^{13}C-NMR spectrum.

Data File: C:\LabSolutions\Data\Analiz\Serkan\NSD-6_24.lcd

Elmt	Val.	Min	Max	Elmt	Val.	Min	Max	Elmt	Val.	Min	Max	Elmt	Val.	Min	Max	Use Adduct
H	1	5	40	O	2	3	5	S	2	0	3	Ru	2	0	0	H
C	4	0	35	F	1	0	0	Cl	1	0	2	I	3	0	0	
N	3	3	6	P	3	0	0	Br	1	0	0					

Error Margin (ppm): 5
HC Ratio: unlimited
Max Isotopes: 3
MSn Iso RI (%): 10.00

DBE Range: 9.0 - 17.0
Apply N Rule: yes
Isotope RI (%): 1.00
MSn Logic Mode: AND

Electron Ions: both
Use MSn Info: yes
Isotope Res: 9000
Max Results: 500

Event#: 1 MS(E+) Ret. Time : 2.107 -> 2.240 Scan# : 317 -> 337

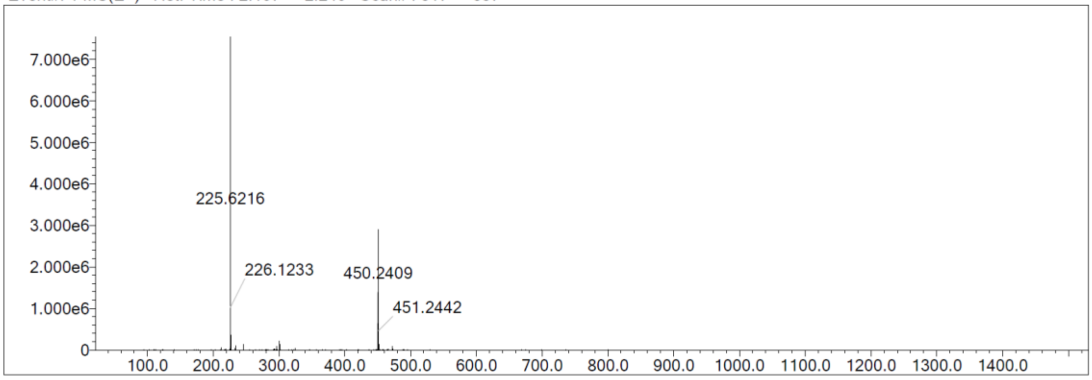

Measured region for 450.2409 m/z

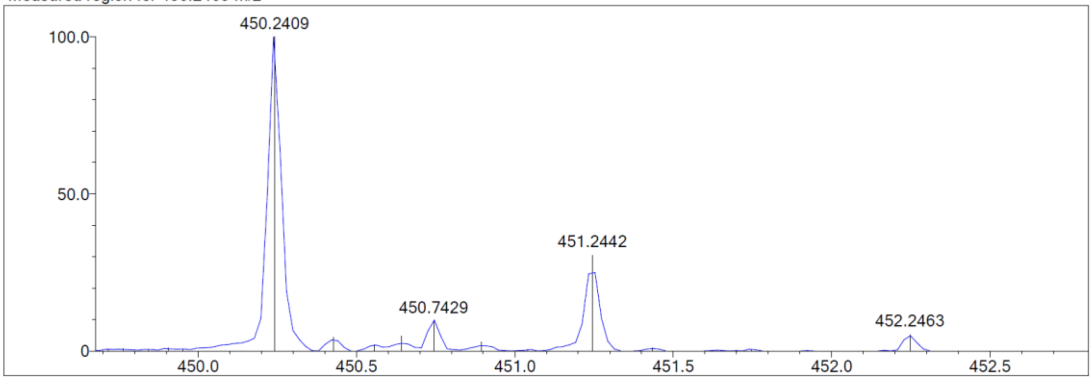

C26 H31 N3 O4 [M+H]+ : Predicted region for 450.2387 m/z

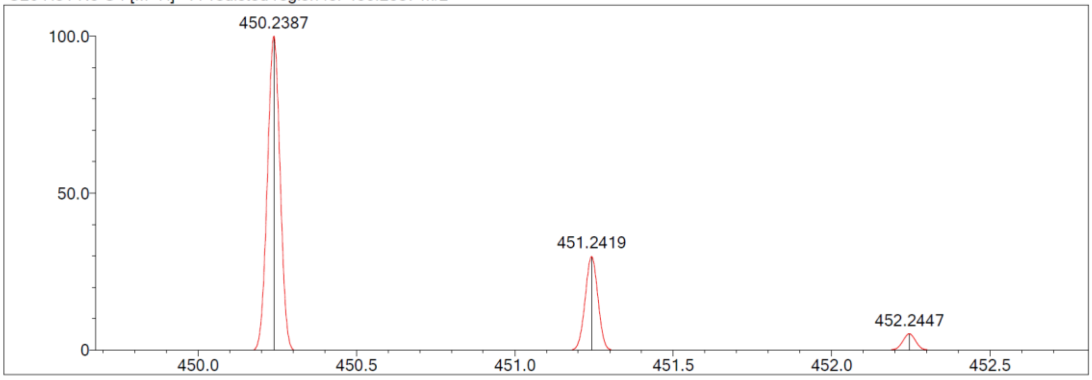

Rank	Score	Formula (M)	Ion	Meas. m/z	Pred. m/z	Df. (mDa)	Df. (ppm)	Iso	DBE
1	84.33	C26 H31 N3 O4	[M+H]+	450.2409	450.2387	2.2	4.89	93.42	13.0

Figure S76. Compound **D6** HRMS report.

*2-(4-Allylpiperazine-1-yl)-N-(4-((5-methoxy-1-oxo-2,3-dihydro-1H-inden-2-ylidene)methyl) phenyl)acetamide (**D7**)*

Dark brown powder. M.P.: 104.6 °C. Yield: 75%.

IR (ATR) ν_{max} (cm^{-1}): 3417 (N-H), 1695 (indanone C=O), 1631 (amide C=O), 1604-1496 (C=C), 1251 (C-N), 1085 (C-O), 806 (1,4-disubstituted benzene).

^{1}H-NMR (300 MHz, DMSO-*d*$_6$) δ (ppm): 2.43 (4H, bs, piperazine CH_2), 2.52 (4H, bs, piperazine CH_2), 2.94 (2H, d, *J*=6.39 Hz, =CH-C$\underline{H_2}$), 3.14 (2H, s, CH_2), 3.89 (3H, s, OCH_3), 4.05 (2H, s, CH_2), 5.10-5.21 (2H, m, HC=$\underline{CH_2}$), 5.74-5.90 (1H, m, $\underline{HC}$=CH_2), 7.02 (1H, dd, J_1=8.49 Hz, J_2=2.22 Hz, methoxy-1-oxo-indenylidene CH), 7.18 (1H, d, *J*=1.98 Hz, methoxy-1-oxo-indenylidene CH), 7.40 (1H, s, C=CH), 7.70-7.73 (3H, m, disubstituted benzene CH, methoxy-1-oxo-indenylidene CH), 7.78 (2H, d, *J*=8.76 Hz, disubstituted benzene CH), 9.96 (1H, s, NH).

^{13}C-NMR (75 MHz, DMSO-*d*$_6$) δ (ppm): 32.5, 52.9, 53.2, 56.3, 61.3, 62.3, 110.6, 115.8, 117.9, 119.9, 125.8, 130.5, 131.1, 131.7, 131.9, 134.5, 140.4, 153.3, 165.3, 169.1, 192.1.

HRMS (ESI) (m/z) $[M+H]^+$: $C_{26}H_{29}N_3O_3$ calculated: 432.2282, found: 432.2300.

DOPNALAB

	Item	Value
	Acquired Date&Time	22.08.2019 11:25:40
	Acquired by	System Administrator
	Filename	C:\Users\dopnalab\Desktop\NURPELİN\DOKTORA TEZ\D71.ispd
	Spectrum name	D71
	Sample name	D7
	Sample ID	
	Option	
	Comment	
	No. of Scans	50
	Resolution	4 [cm-1]
	Apodization	Happ-Genzel

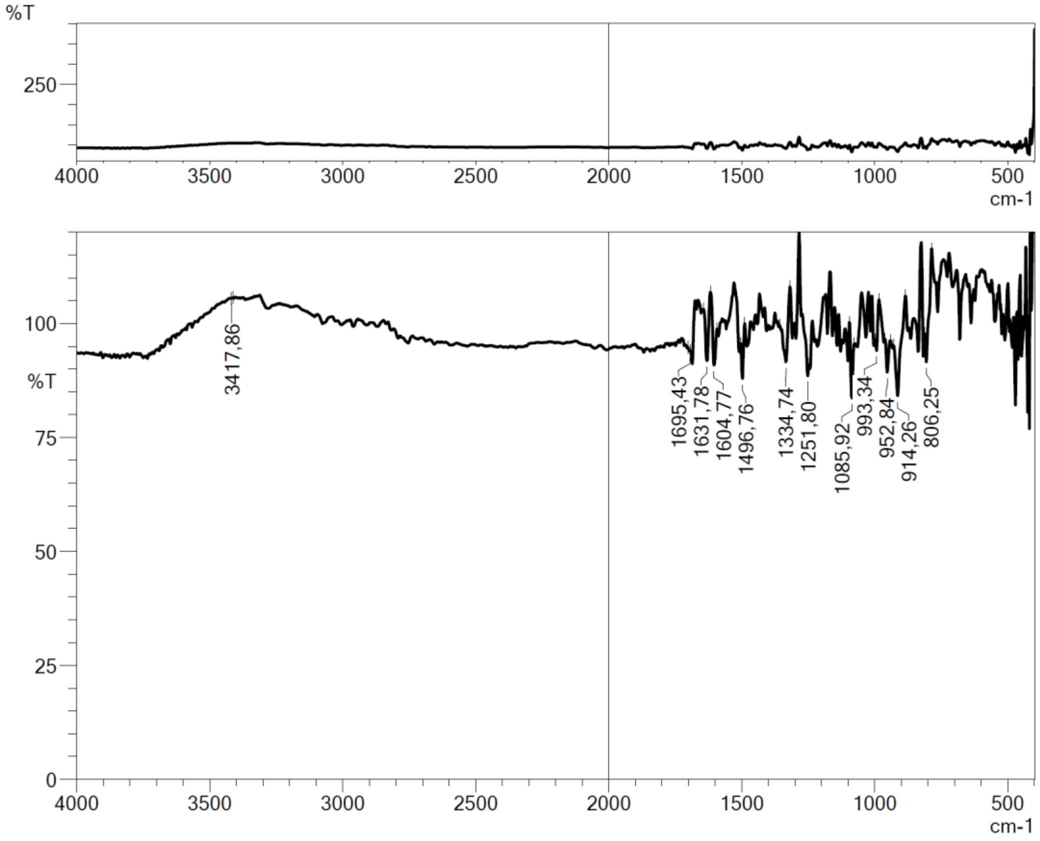

Figure S77. Compound **D7** IR report.

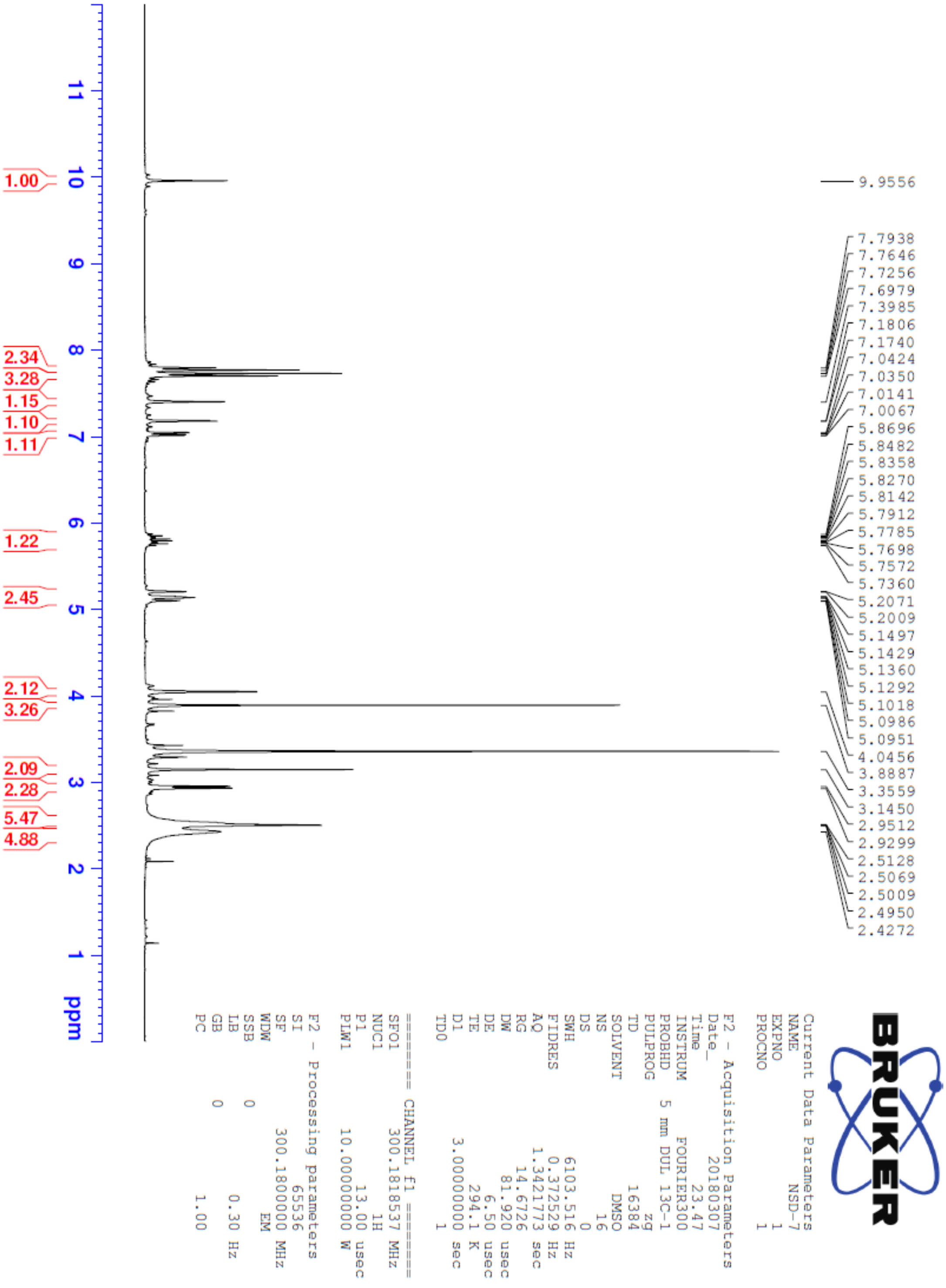

Figure S78. Compound **D7** ^{1}H-NMR spectrum.

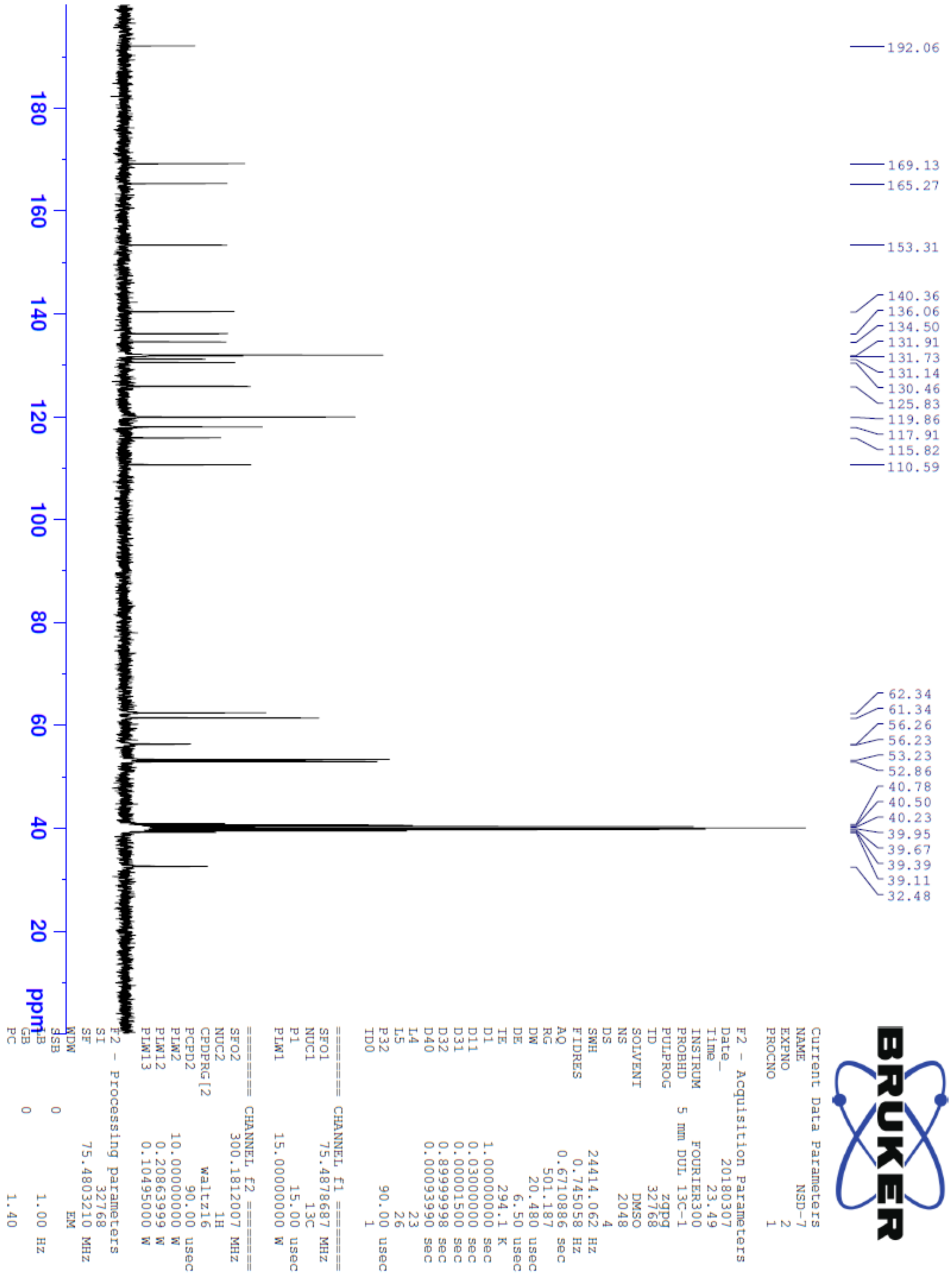

Figure S79. Compound **D7** ^{13}C-NMR spectrum.

Data File: C:\LabSolutions\Data\Analiz\Serkan\NSD-7_25.lcd

Elmt	Val.	Min	Max	Elmt	Val.	Min	Max	Elmt	Val.	Min	Max	Elmt	Val.	Min	Max	Use Adduct
H	1	5	40	O	2	3	5	S	2	0	3	Ru	2	0	0	H
C	4	0	35	F	1	0	0	Cl	1	0	2	I	3	0	0	
N	3	3	6	P	3	0	0	Br	1	0	0					

Error Margin (ppm):	5	DBE Range:	10.0 - 17.0	Electron Ions:	both
HC Ratio:	unlimited	Apply N Rule:	yes	Use MSn Info:	yes
Max Isotopes:	3	Isotope RI (%):	1.00	Isotope Res:	9000
MSn Iso RI (%):	10.00	MSn Logic Mode:	AND	Max Results:	500

Event#: 1 MS(E+) Ret. Time : 2.173 -> 2.400 Scan# : 327 -> 361

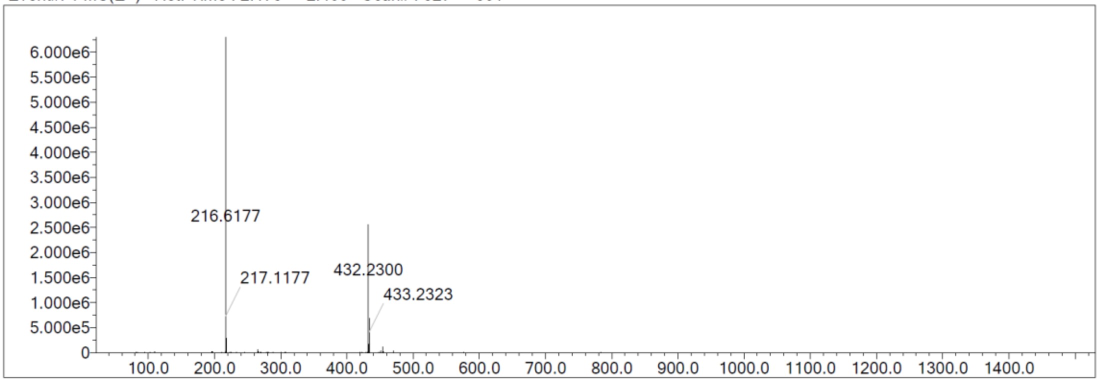

Measured region for 432.2300 m/z

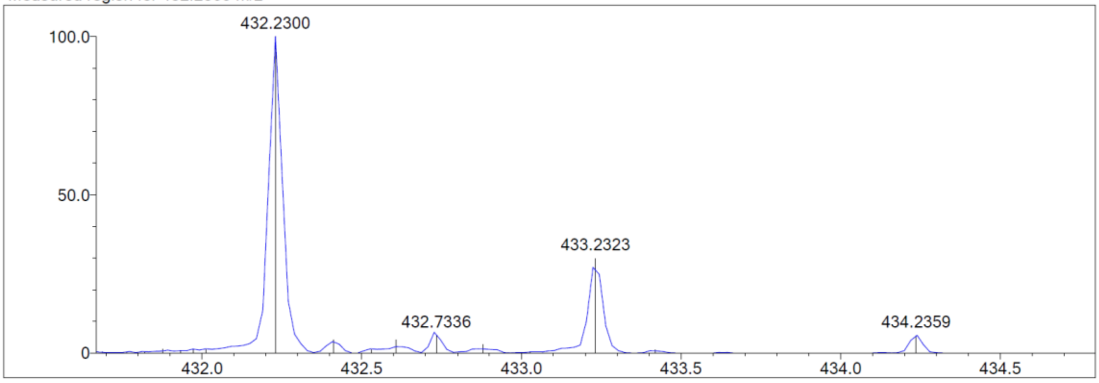

C26 H29 N3 O3 [M+H]+ : Predicted region for 432.2282 m/z

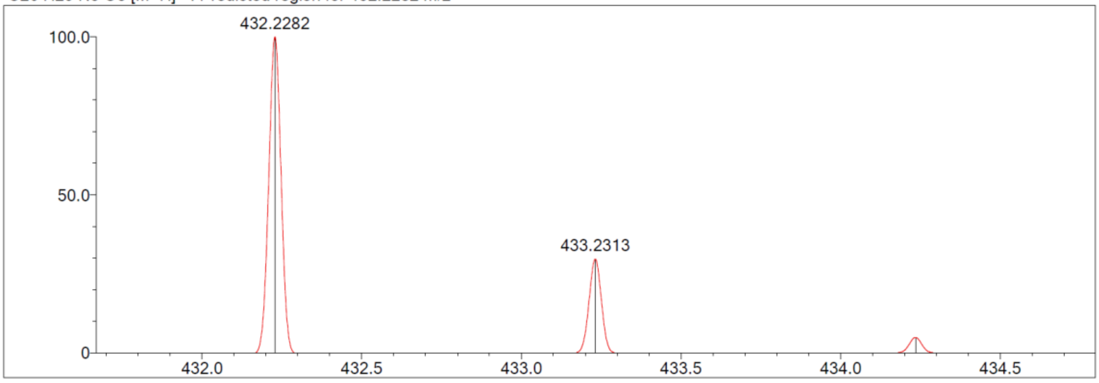

Rank	Score	Formula (M)	Ion	Meas. m/z	Pred. m/z	Df. (mDa)	Df. (ppm)	Iso	DBE
1	86.24	C26 H29 N3 O3	[M+H]+	432.2300	432.2282	1.8	4.16	93.64	14.0

Figure S80. Compound **D7** HRMS report.

*2-(4-Allylpiperazine-1-yl)-N-(4-((6-methoxy-1-oxo-2,3-dihydro-1H-inden-2-ylidene)methyl) phenyl)acetamide (**D8**)*

Yellow powder. M.P.: 180.3 °C. Yield: 86%.

IR (ATR) ν_{max} (cm^{-1}): 3329 (N-H), 1705 (indanone C=O), 1653 (amide C=O), 1593-1525 (C=C), 1199 (C-N), 1157 (C-O), 840 (1,4-disubstituted benzene).

^{1}H-NMR (300 MHz, DMSO-*d_6*) δ (ppm): 2.43 (4H, bs, piperazine CH_2), 2.52 (4H, bs, piperazine CH_2), 2.94 (2H, d, J=6.36 Hz, =CH-C$\underline{H_2}$), 3.15 (2H, s, CH_2), 3.83 (3H, s, OCH_3), 4.01 (2H, s, CH_2), 5.10-5.21 (2H, m, HC=$\underline{CH_2}$), 5.74-5.87 (1H, m, $\underline{HC}$=CH_2), 7.24 (1H, d, J=2.46 Hz, methoxy-1-oxo-indenylidene CH), 7.29 (1H, dd, J_1=8.34 Hz, J_2=2.55 Hz, methoxy-1-oxo-indenylidene CH), 7.48 (1H, s, C=CH), 7.59 (1H, d, J=8.37 Hz, methoxy-1-oxo-indenylidene CH), 7.74 (2H, d, d, J=9.03 Hz, disubstituted benzene CH), 7.79 (2H, d, J=8.97 Hz, disubstituted benzene CH), 9.97 (1H, s, NH).

^{13}C-NMR (75 MHz, DMSO-*d_6*) δ (ppm): 31.7, 52.9, 53.2, 56.0, 61.3, 62.3, 106.0, 117.9, 119.9, 123.7, 130.3, 132.2, 133.0, 134.7, 136.1, 139.1, 140.6, 143.0, 159.6, 169.2, 193.6.

HRMS (ESI) (m/z) $[M+H]^+$: $C_{26}H_{29}N_3O_3$ calculated: 432.2282, found: 432.2297.

DOPNALAB

	Item	Value
	Acquired Date&Time	22.08.2019 11:29:45
	Acquired by	System Administrator
	Filename	C:\Users\dopnalab\Desktop\NURPELİN\DOKTORA TEZ\D81.ispd
	Spectrum name	D81
	Sample name	D8
	Sample ID	
	Option	
	Comment	
	No. of Scans	50
	Resolution	4 [cm-1]
	Apodization	Happ-Genzel

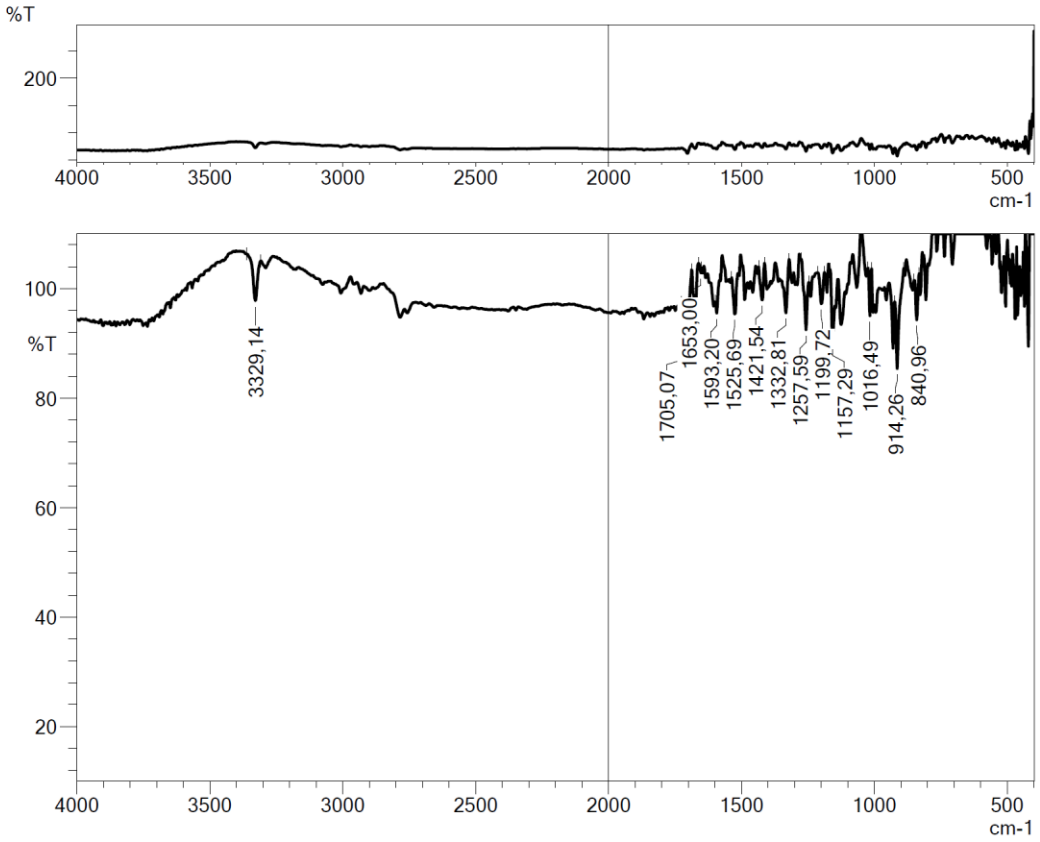

Figure S81. Compound **D8** IR report.

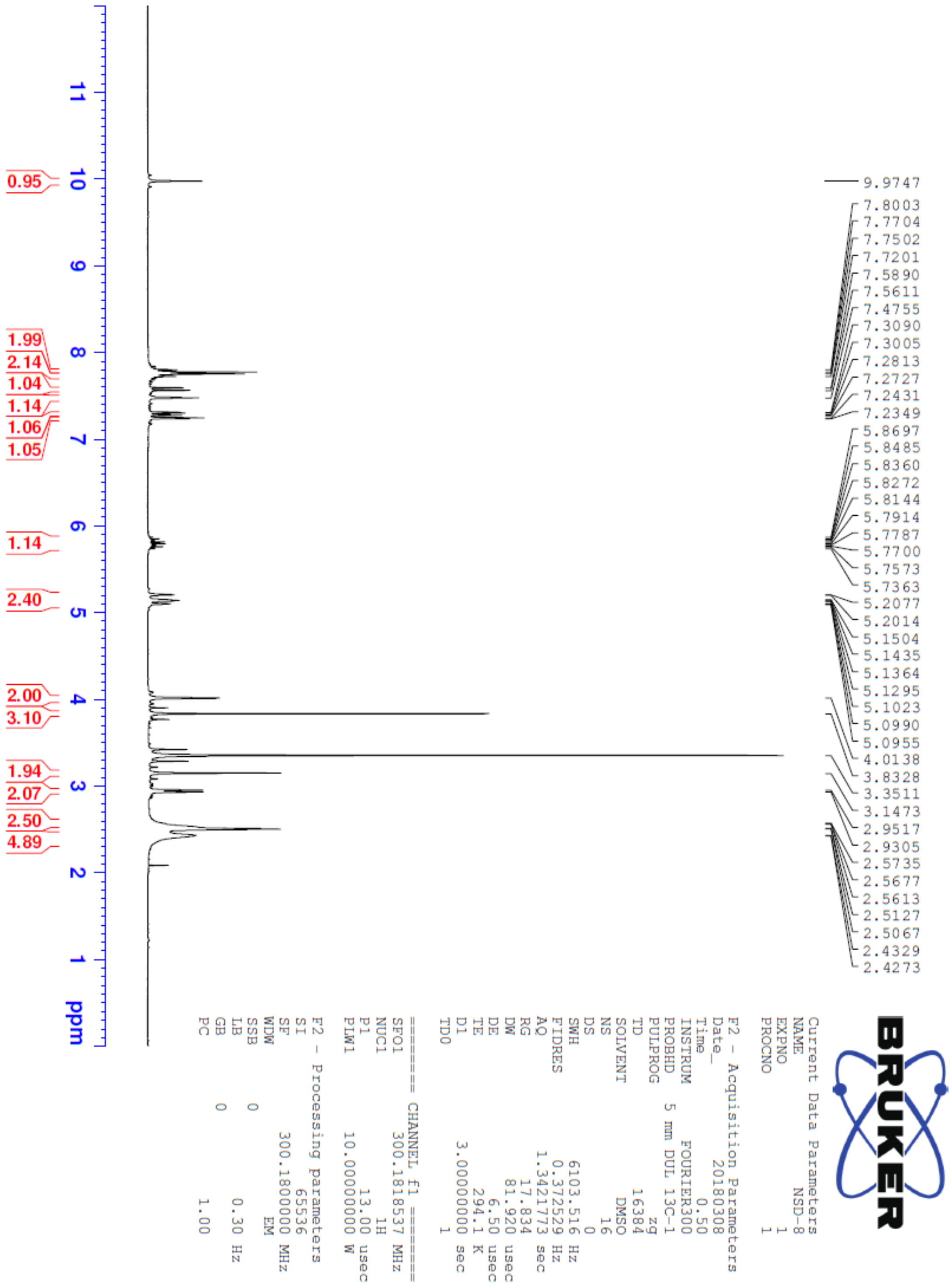

Figure S82. Compound **D8** ^{1}H-NMR spectrum.

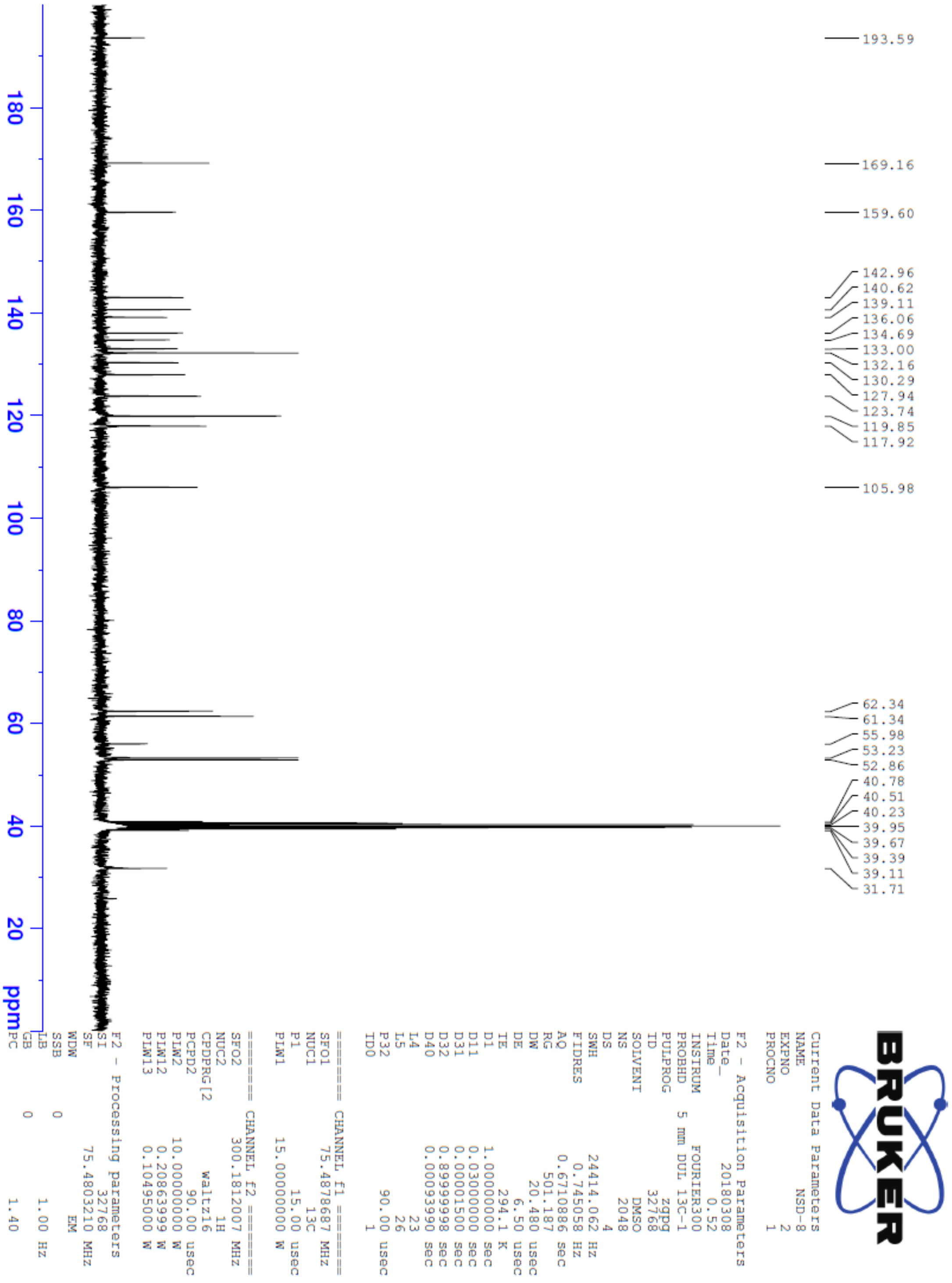

Figure S83. Compound **D8** ^{13}C-NMR spectrum.

Formula Predictor Report - NSD-8_26.lcd

Data File: C:\LabSolutions\Data\Analiz\Serkan\NSD-8_26.lcd

Elmt	Val.	Min	Max	Elmt	Val.	Min	Max	Elmt	Val.	Min	Max	Elmt	Val.	Min	Max	Use Adduct
H	1	5	40	O	2	3	5	S	2	0	3	Ru	2	0	0	H
C	4	0	35	F	1	0	0	Cl	1	0	2	I	3	0	0	
N	3	3	6	P	3	0	0	Br	1	0	0					

Error Margin (ppm): 5
HC Ratio: unlimited
Max Isotopes: 3
MSn Iso RI (%): 10.00

DBE Range: 10.0 - 17.0
Apply N Rule: yes
Isotope RI (%): 1.00
MSn Logic Mode: AND

Electron Ions: both
Use MSn Info: yes
Isotope Res: 9000
Max Results: 500

Event#: 1 MS(E+) Ret. Time : 2.227 -> 2.373 Scan# : 335 -> 357

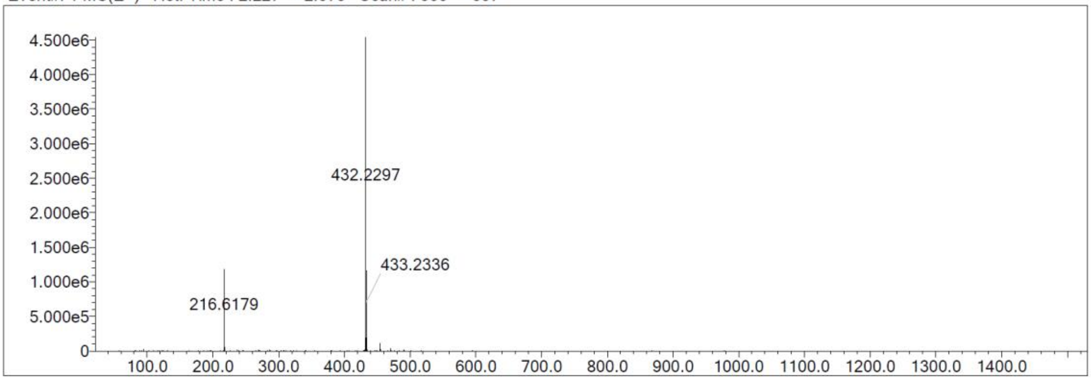

Measured region for 432.2297 m/z

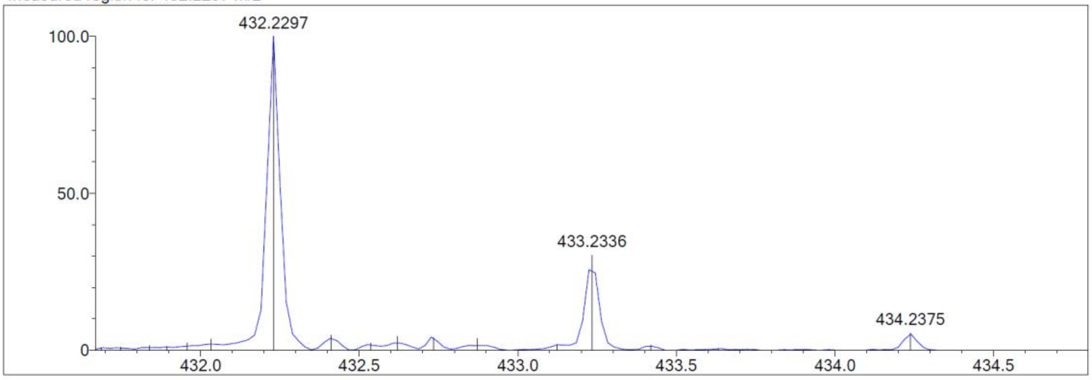

C26 H29 N3 O3 [M+H]+ : Predicted region for 432.2282 m/z

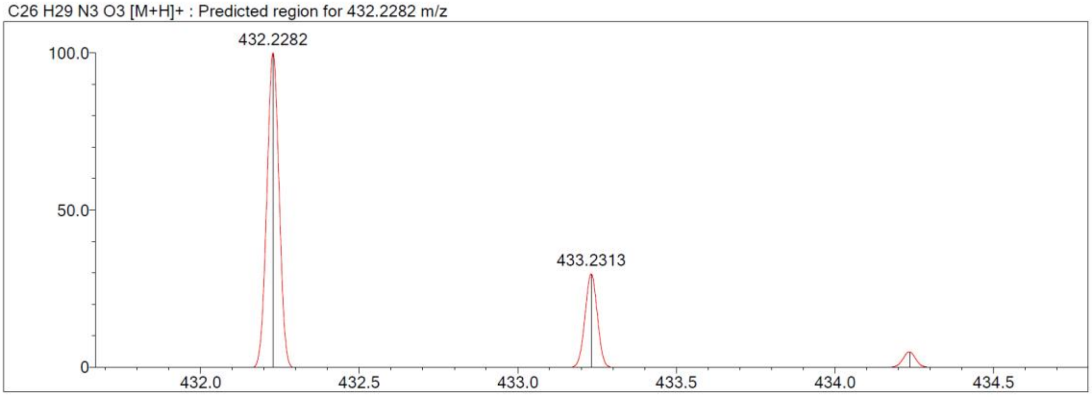

Rank	Score	Formula (M)	Ion	Meas. m/z	Pred. m/z	Df. (mDa)	Df. (ppm)	Iso	DBE
1	84.80	C26 H29 N3 O3	[M+H]+	432.2297	432.2282	1.5	3.47	90.39	14.0

Figure S84. Compound **D8** HRMS report.

*2-(4-Allylpiperazine-1-yl)-N-(4-((5,6-dimethoxy-1-oxo-2,3-dihydro-1H-inden-2-ylidene) methyl)phenyl)acetamide (**D9**)*

Yellow powder. M.P.: 138.5 °C. Yield: 83%.

IR (ATR) ν$_{max}$ (cm^{-1}): 3360 (N-H), 1670 (indanone C=O), 1635 (amide C=O), 1525-1498 (C=C), 1220 (C-N), 1091 (C-O), 840 (1,4-disubstituted benzene).

^{1}H-NMR (300 MHz, DMSO-*d$_6$*) δ (ppm): 2.43 (4H, bs, piperazine CH_2), 2.52 (4H, bs, piperazine CH_2), 2.94 (2H, d, *J*=6.38 Hz, =CH-C$\underline{H_2}$), 3.14 (2H, s, CH_2), 3.83 (3H, s, OCH_3), 3.90 (3H, s, OCH_3), 3.97 (2H, s, CH_2), 5.10-5.21 (2H, m, HC=$\underline{CH_2}$), 5.74-5.87 (1H, m, $\underline{HC}$=CH_2), 7.20 (1H, s, methoxy-1-oxo-indenylidene CH), 7.21 (1H, s, methoxy-1-oxo-indenylidene CH), 7.38 (1H, s, C=CH), 7.70 (2H, d, *J*=8.88 Hz, disubstituted benzene CH), 7.77 (2H, d, *J*=8.81 Hz, disubstituted benzene CH), 9.95 (1H, s, NH).

^{13}C-NMR (75 MHz, DMSO-*d$_6$*) δ (ppm): 32.1, 52.9, 53.2, 56.1, 56.4, 61.3, 62.3, 105.0, 108.5, 117.9, 119.9, 130.5, 131.3, 131.8, 134.8, 136.1, 140.3, 145.4, 149.7, 155.6, 169.1, 192.3.

HRMS (ESI) (m/z) [M+H]$^+$: $C_{27}H_{31}N_3O_4$ calculated: 462.2387, found: 462.2409.

DOPNALAB

	Item	Value
	Acquired Date&Time	22.08.2019 11:32:22
	Acquired by	System Administrator
	Filename	C:\Users\dopnalab\Desktop\NURPELİN\DOKTORA TEZ\D91.ispd
	Spectrum name	D91
	Sample name	D9
	Sample ID	
	Option	
	Comment	
	No. of Scans	50
	Resolution	4 [cm-1]
	Apodization	Happ-Genzel

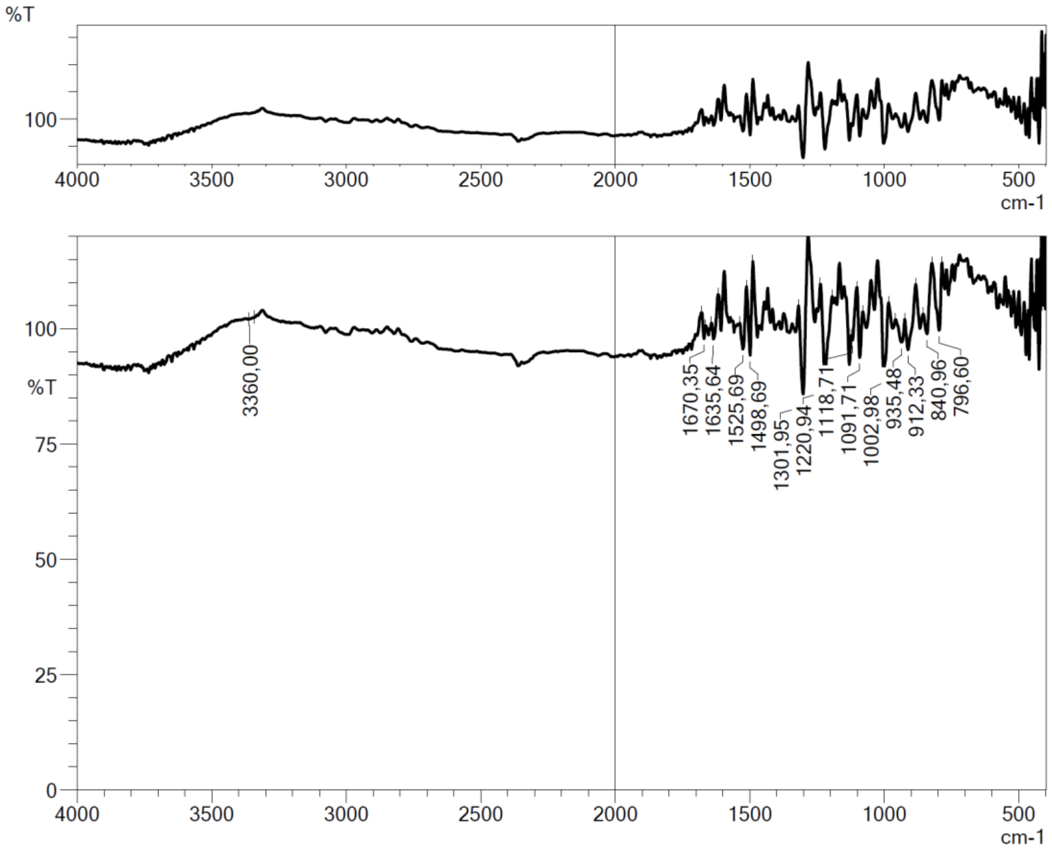

Figure S85. Compound **D9** IR report.

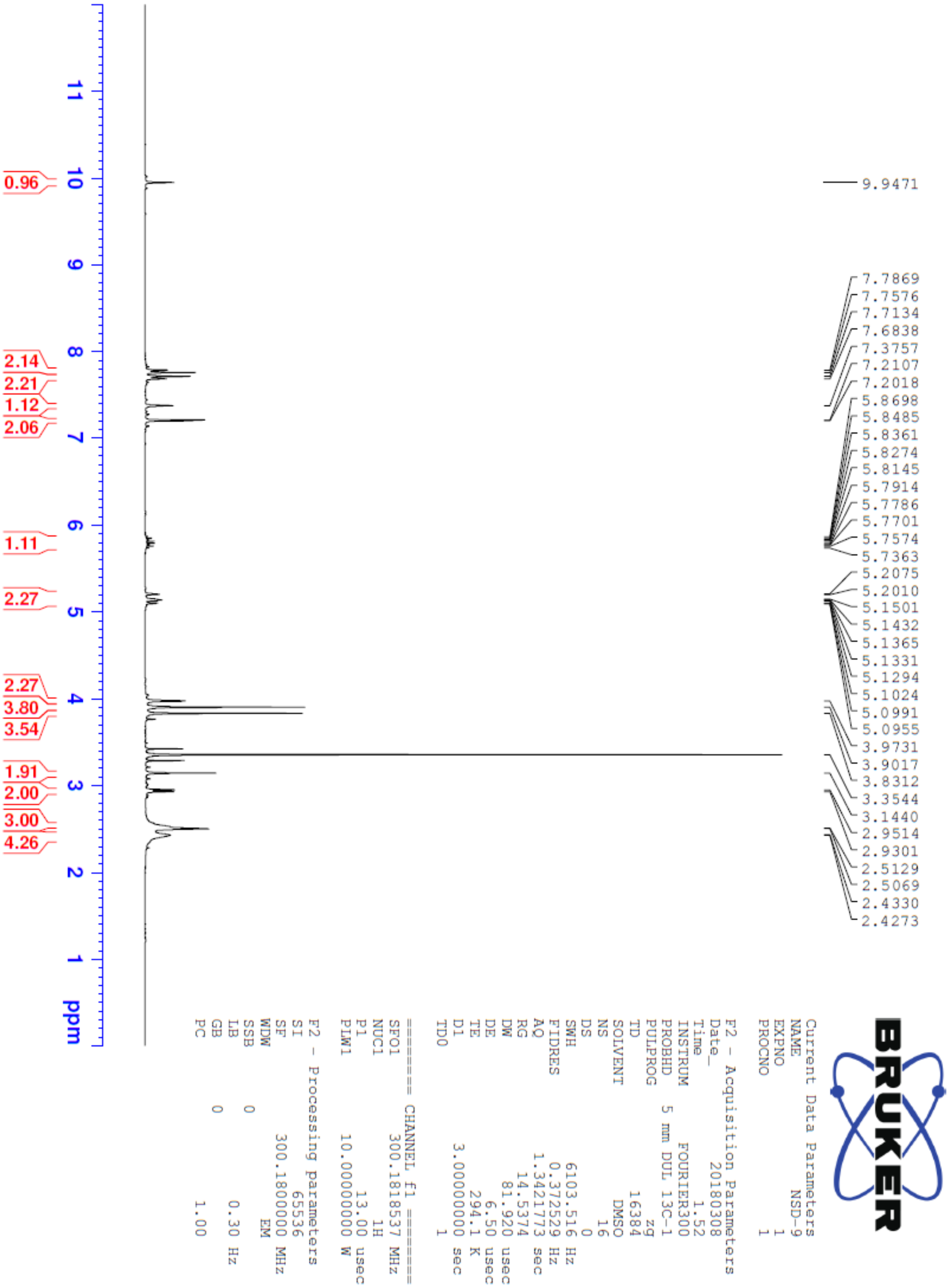

Figure S86. Compound **D9** ¹H-NMR spectrum.

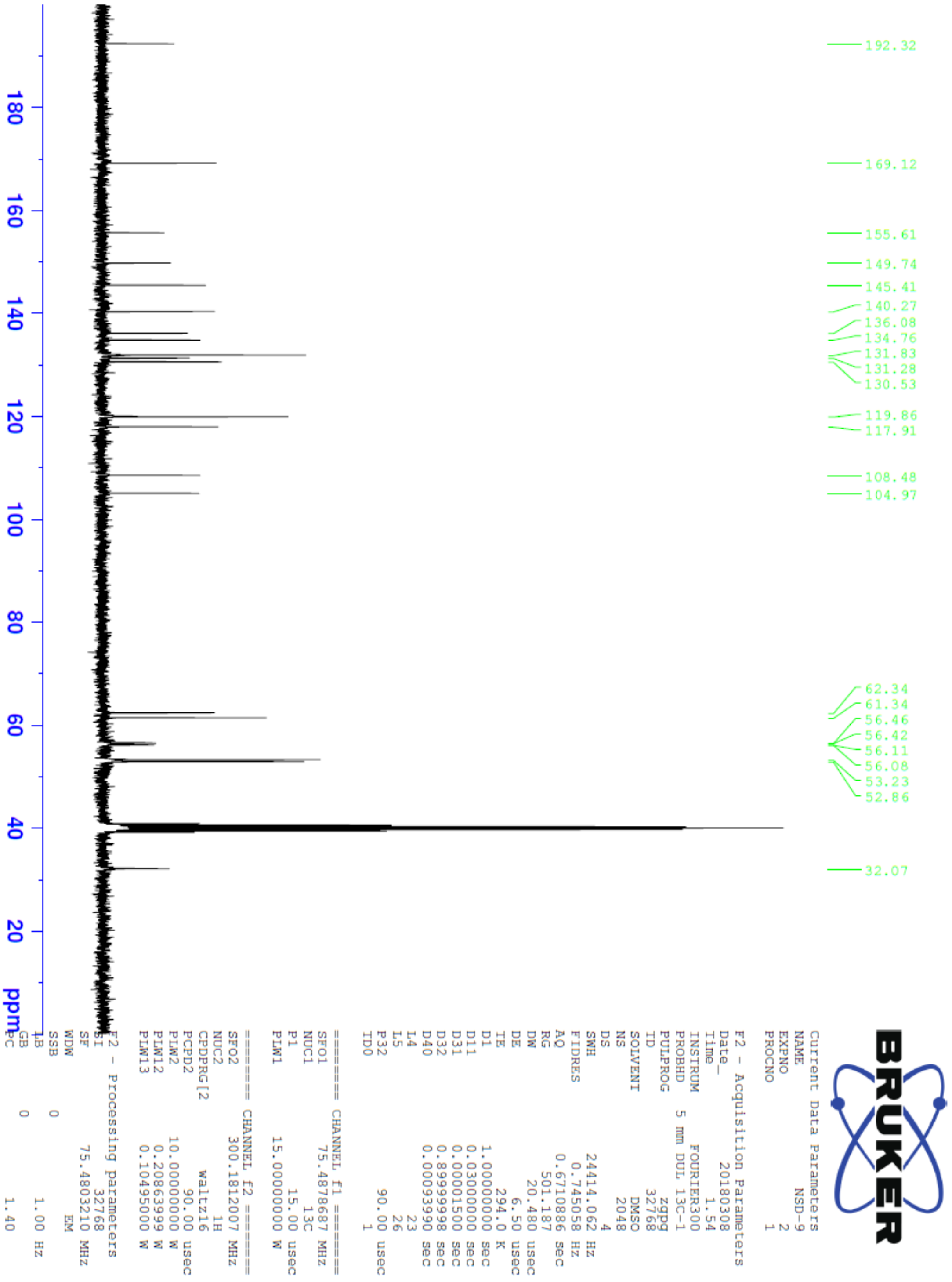

Figure S87. Compound **D9** ^{13}C-NMR spectrum.

Data File: C:\LabSolutions\Data\Analiz\Serkan\NSD-9_49.lcd

Elmt	Val.	Min	Max	Elmt	Val.	Min	Max	Elmt	Val.	Min	Max	Elmt	Val.	Min	Max	Use Adduct
H	1	5	40	O	2	3	5	S	2	0	3	Ru	2	0	0	H
C	4	0	35	F	1	0	0	Cl	1	0	2	I	3	0	0	
N	3	3	6	P	3	0	0	Br	1	0	0					

Error Margin (ppm):	5	DBE Range:	10.0 - 17.0	Electron Ions:	both
HC Ratio:	unlimited	Apply N Rule:	yes	Use MSn Info:	yes
Max Isotopes:	3	Isotope RI (%):	1.00	Isotope Res:	9000
MSn Iso RI (%):	10.00	MSn Logic Mode:	AND	Max Results:	500

Event#: 1 MS(E+) Ret. Time : 2.080 -> 2.293 Scan# : 313 -> 345

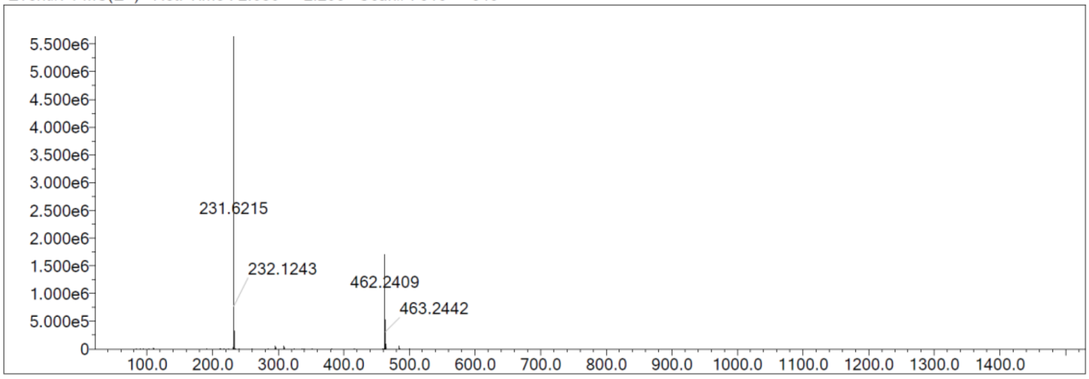

Measured region for 462.2409 m/z

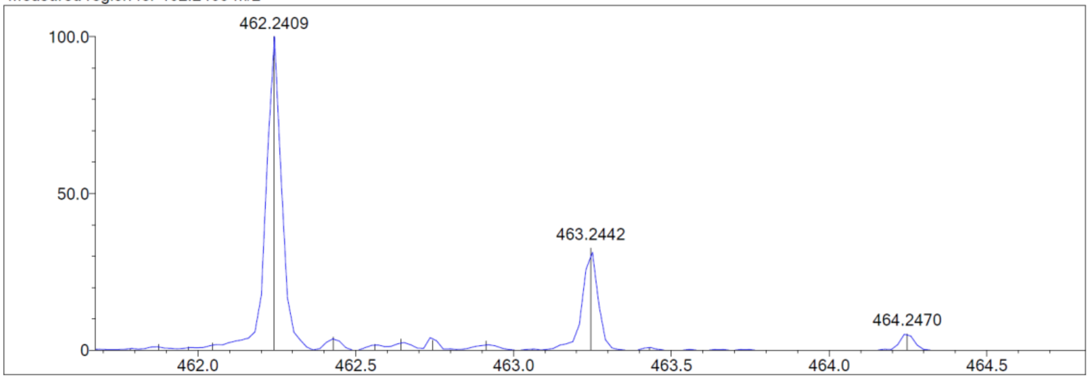

C27 H31 N3 O4 [M+H]+ : Predicted region for 462.2387 m/z

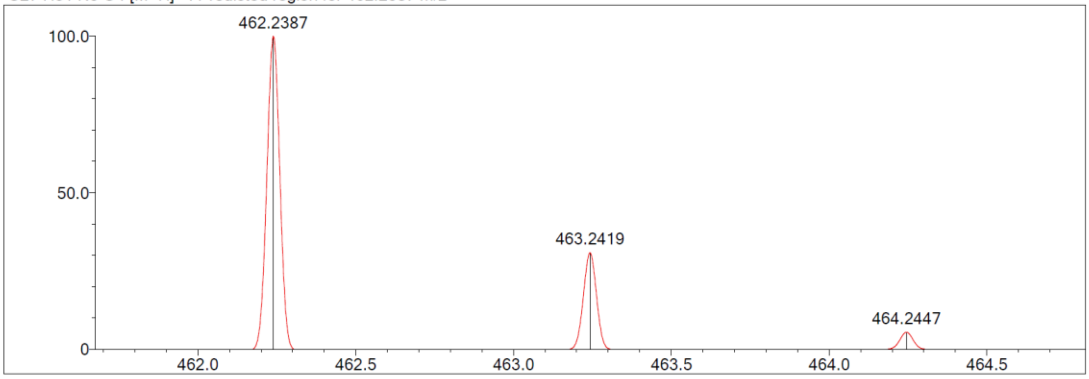

Rank	Score	Formula (M)	Ion	Meas. m/z	Pred. m/z	Df. (mDa)	Df. (ppm)	Iso	DBE
1	82.04	C27 H31 N3 O4	[M+H]+	462.2409	462.2387	2.2	4.76	90.55	14.0

Figure S88. Compound **D9** HRMS report.

*2-(4-Propargylpiperazine-1-yl)-N-(4-((5-methoxy-1-oxo-2,3-dihydro-1H-inden-2-ylidene) methyl)phenyl)acetamide (**D10**)*

Brown powder. M.P.: 141.5 °C. Yield: 80%.

IR (ATR) ν_{max} (cm^{-1}): 3290 (N-H), 1670 (indanone C=O), 1629 (amide C=O), 1585-1498 (C=C), 1255 (C-N), 1087 (C-O), 864 (1,4-disubstituted benzene).

^{1}H-NMR (300 MHz, DMSO-*d_6*) δ (ppm): 2.54 (8H, bs, piperazine CH_2), 3.16-3.17 (3H, m, CH, CH_2), 3.89 (3H, s, OCH_3), 4.05 (2H, s, CH_2), 7.03 (1H, dd, J_1=8.49 Hz, J_2=2.22 Hz, methoxy-1-oxo-indenylidene CH), 7.18 (1H, d, J=1.98 Hz, methoxy-1-oxo-indenylidene CH), 7.40 (1H, s, C=CH), 7.70-7.73 (3H, m, disubstituted benzene CH, methoxy-1-oxo-indenylidene CH), 7.78 (2H, d, J=8.73 Hz, disubstituted benzene CH), 9.98 (1H, s, NH).

^{13}C-NMR (75 MHz, DMSO-*d_6*) δ (ppm): 32.5, 46.5, 51.5, 53.1, 56.3, 62.3, 76.2, 79.9, 110.6, 115.8, 119.9, 125.8, 130.5, 131.1, 131.7, 131.9, 134.5, 140.4, 153.3, 165.3, 169.1, 192.0.

HRMS (ESI) (m/z) $[M+H]^+$: $C_{26}H_{27}N_3O_3$ calculated: 430.2125, found: 430.2136.

DOPNALAB

	Item	Value
	Acquired Date&Time	22.08.2019 11:35:16
	Acquired by	System Administrator
	Filename	C:\Users\dopnalab\Desktop\NURPELİN\DOKTORA TEZ\D101.ispd
	Spectrum name	D101
	Sample name	D10
	Sample ID	
	Option	
	Comment	
	No. of Scans	50
	Resolution	4 [cm-1]
	Apodization	Happ-Genzel

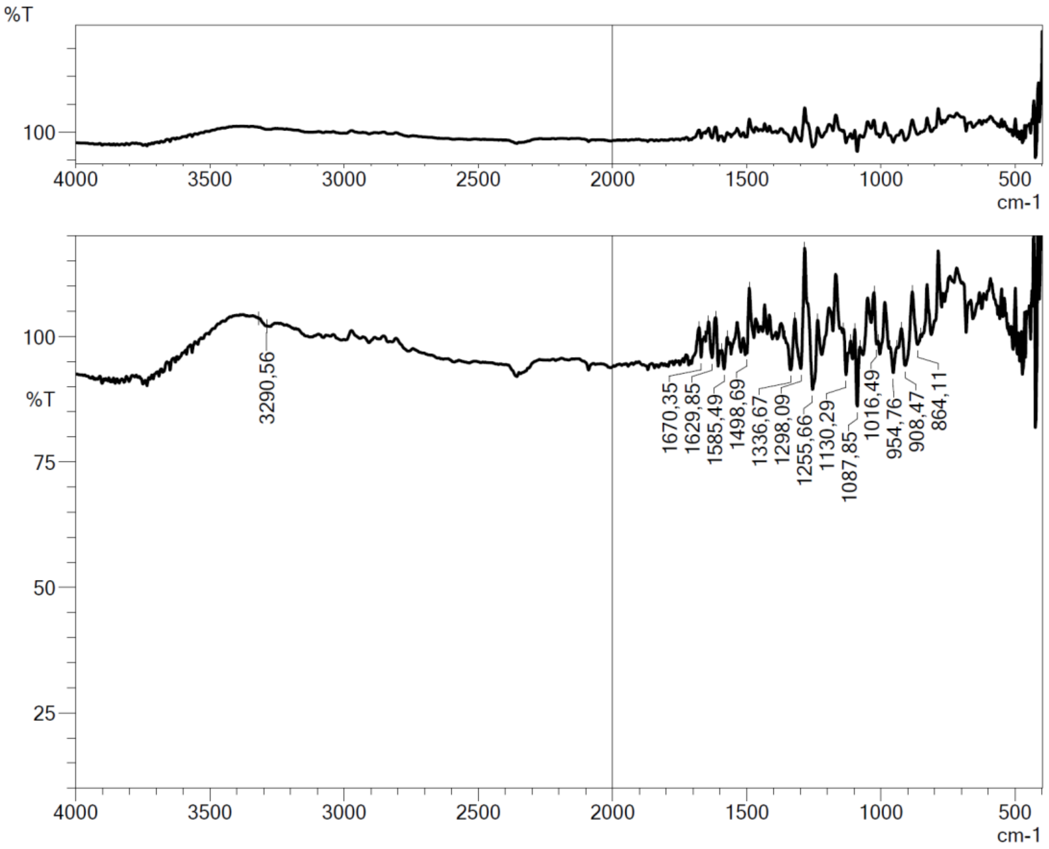

Figure S89. Compound **D10** IR report.

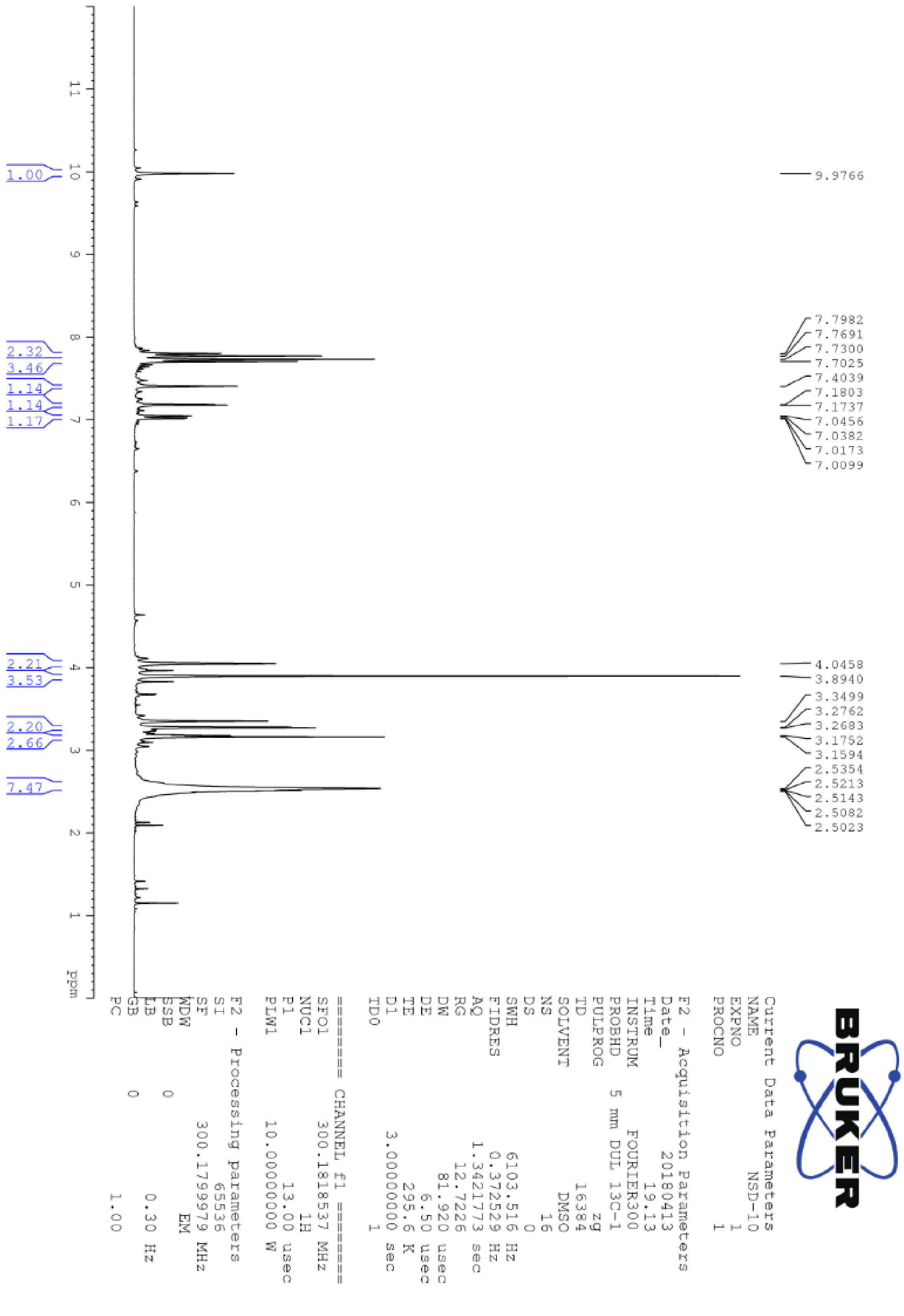

Figure S90. Compound **D10** ^{1}H-NMR spectrum.

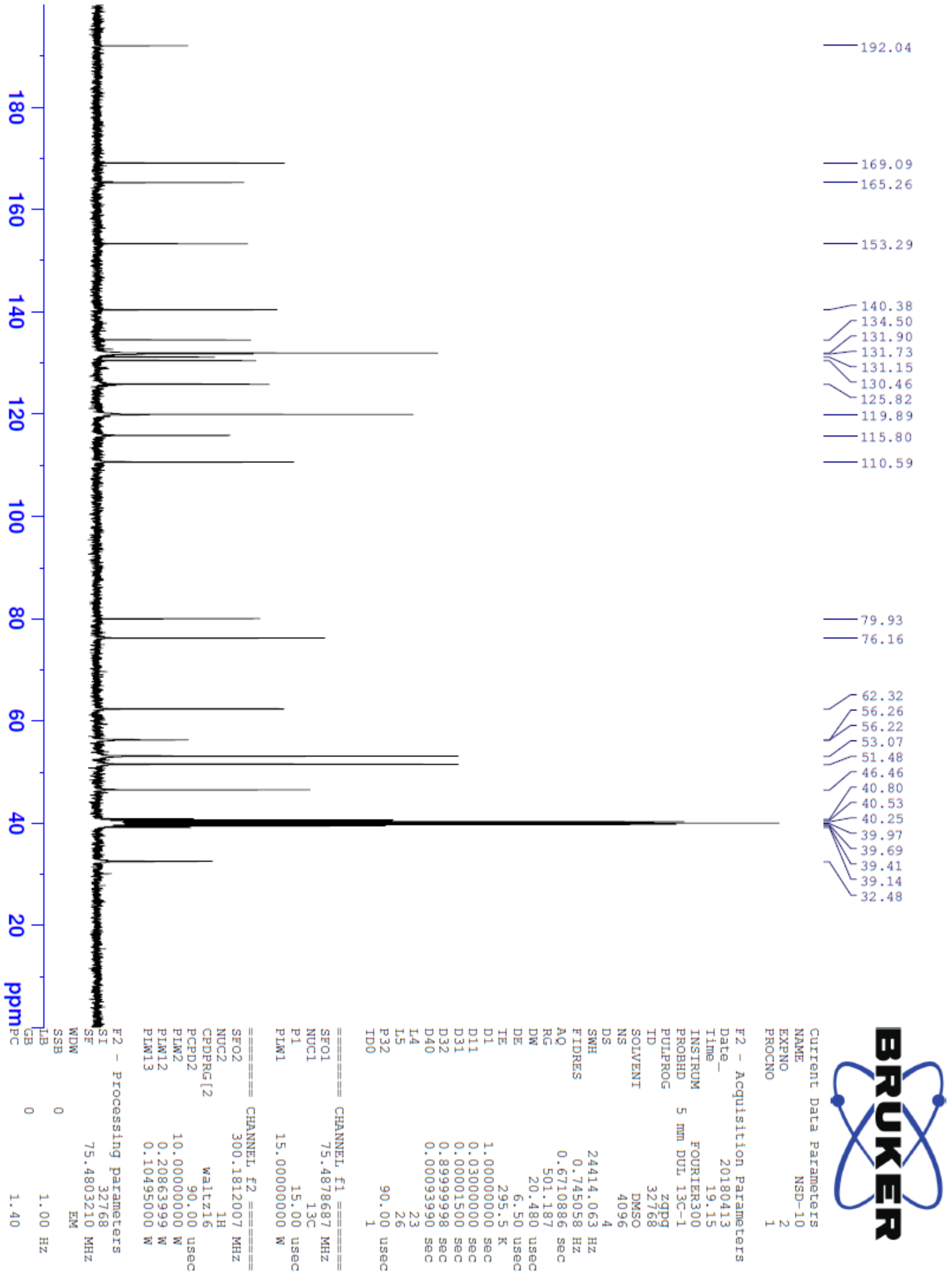

Figure S91. Compound **D10** ^{13}C-NMR spectrum.

Formula Predictor Report - NSD-10_50.lcd   Page 1 of 1

Data File: C:\LabSolutions\Data\Analiz\Serkan\NSD-10_50.lcd

Elmt	Val.	Min	Max	Elmt	Val.	Min	Max	Elmt	Val.	Min	Max	Elmt	Val.	Min	Max	Use Adduct
H	1	5	40	O	2	3	5	S	2	0	3	Ru	2	0	0	H
C	4	0	35	F	1	0	0	Cl	1	0	2	I	3	0	0	
N	3	3	6	P	3	0	0	Br	1	0	0					

Error Margin (ppm): 5
HC Ratio: unlimited
Max Isotopes: 3
MSn Iso RI (%): 10.00

DBE Range: 10.0 - 17.0
Apply N Rule: yes
Isotope RI (%): 1.00
MSn Logic Mode: AND

Electron Ions: both
Use MSn Info: yes
Isotope Res: 9000
Max Results: 500

Event#: 1 MS(E+)   Ret. Time : 2.267 -> 2.373   Scan# : 341 -> 357

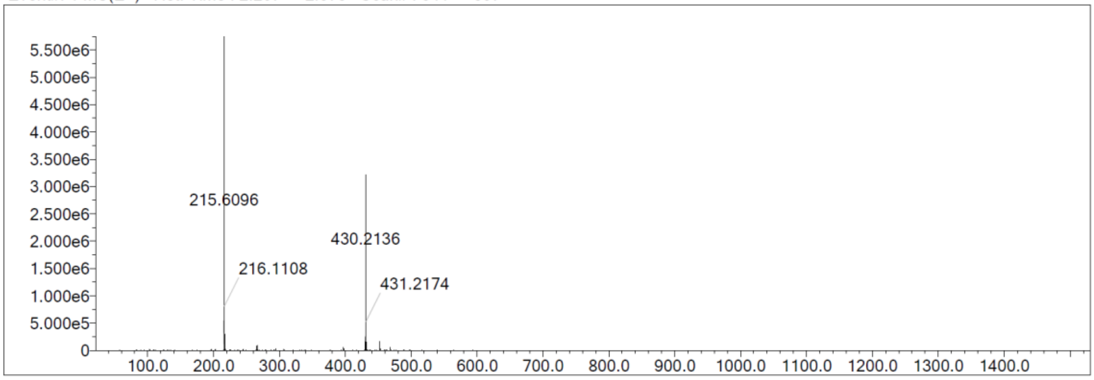

Measured region for 430.2136 m/z

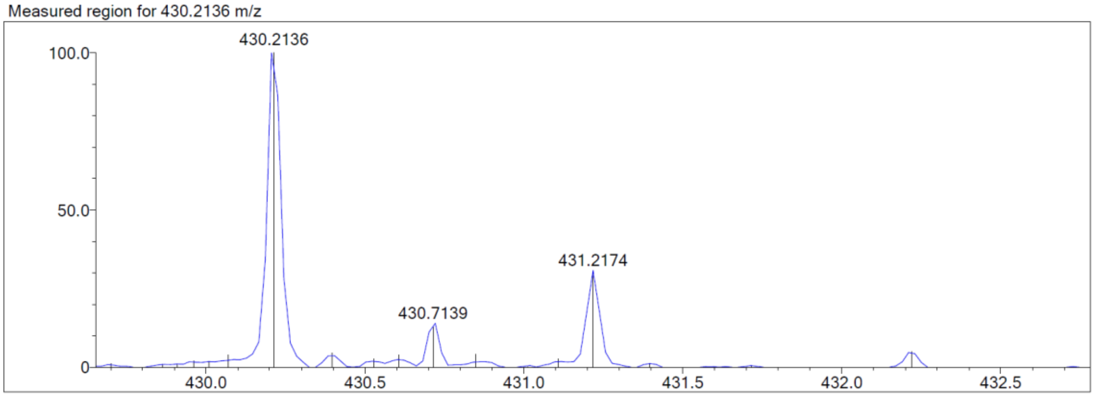

C26 H27 N3 O3 [M+H]+ : Predicted region for 430.2125 m/z

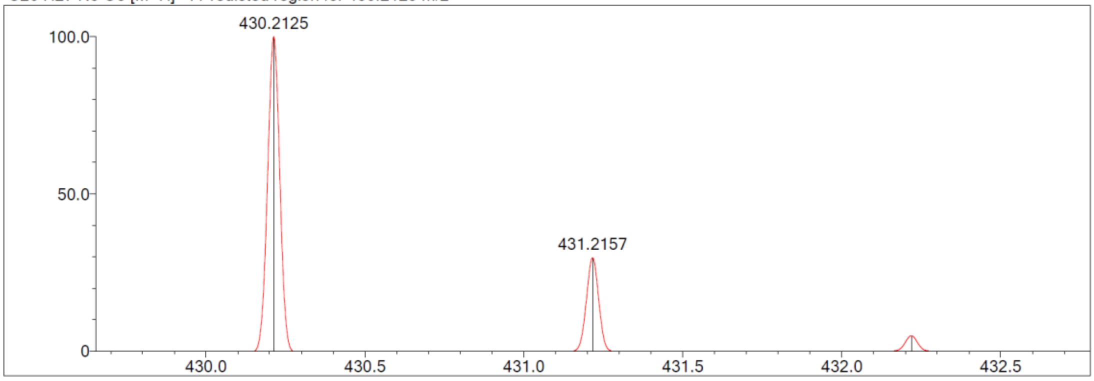

Rank	Score	Formula (M)	Ion	Meas. m/z	Pred. m/z	Df. (mDa)	Df. (ppm)	Iso	DBE
1	91.58	C26 H27 N3 O3	[M+H]+	430.2136	430.2125	1.1	2.56	95.30	15.0

Figure S92. Compound **D10** HRMS report.

*2-(4-Propargylpiperazine-1-yl)-N-(4-((6-methoxy-1-oxo-2,3-dihydro-1H-inden-2-ylidene) methyl)phenyl)acetamide (**D11**)*

Light brown powder. M.P.: 139.7 °C. Yield: 86%.

IR (ATR) ν_{max} (cm^{-1}): 3319 (N-H), 1689 (indanone C=O), 1625 (amide C=O), 1597-1516 (C=C), 1253 (C-N), 1124 (C-O), 817 (1,4-disubstituted benzene).

^{1}H-NMR (300 MHz, DMSO-d_6) δ (ppm): 2.53 (8H, bs, piperazine CH_2), 3.16-3.27 (3H, m, CH, CH_2), 3.83 (3H, s, OCH_3), 4.02 (2H, s, CH_2), 7.24 (1H, d, J=2.43 Hz, methoxy-1-oxo-indenylidene CH), 7.03 (1H, dd, J_1=8.28 Hz, J_2=2.55 Hz, methoxy-1-oxo-indenylidene CH), 7.48 (1H, s, C=CH), 7.58 (1H, d, J=8.34 Hz, methoxy-1-oxo-indenylidene CH), 7.74 (2H, d, J=9.09 Hz, disubstituted benzene CH), 7.79 (2H, d, J=9.03 Hz, disubstituted benzene CH), 10.00 (1H, s, NH).

^{13}C-NMR (75 MHz, DMSO-d_6) δ (ppm): 31.7, 46.5, 51.5, 53.1, 56.0, 62.3, 76.2, 79.9, 106.0, 119.9, 123.7, 127.9, 130.3, 132.2, 133.0, 134.7, 139.1, 140.7, 143.0, 159.6, 169.1, 193.6.

HRMS (ESI) (m/z) $[M+H]^+$: $C_{26}H_{27}N_3O_3$ calculated: 430.2125, found: 430.2130.

DOPNALAB

	Item	Value
	Acquired Date&Time	22.08.2019 11:39:45
	Acquired by	System Administrator
	Filename	C:\Users\dopnalab\Desktop\NURPELİN\DOKTORA TEZ\D111.ispd
	Spectrum name	D111
	Sample name	D11
	Sample ID	
	Option	
	Comment	
	No. of Scans	50
	Resolution	4 [cm-1]
	Apodization	Happ-Genzel

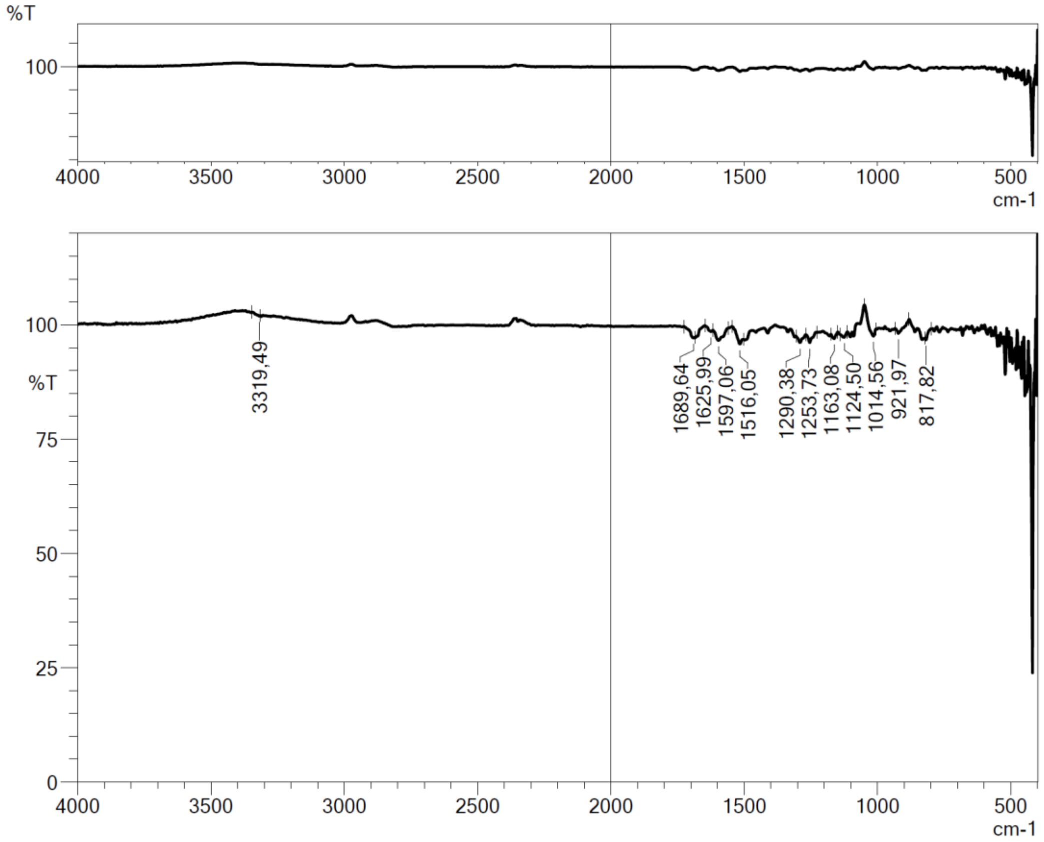

Figure S93. Compound **D11** IR report.

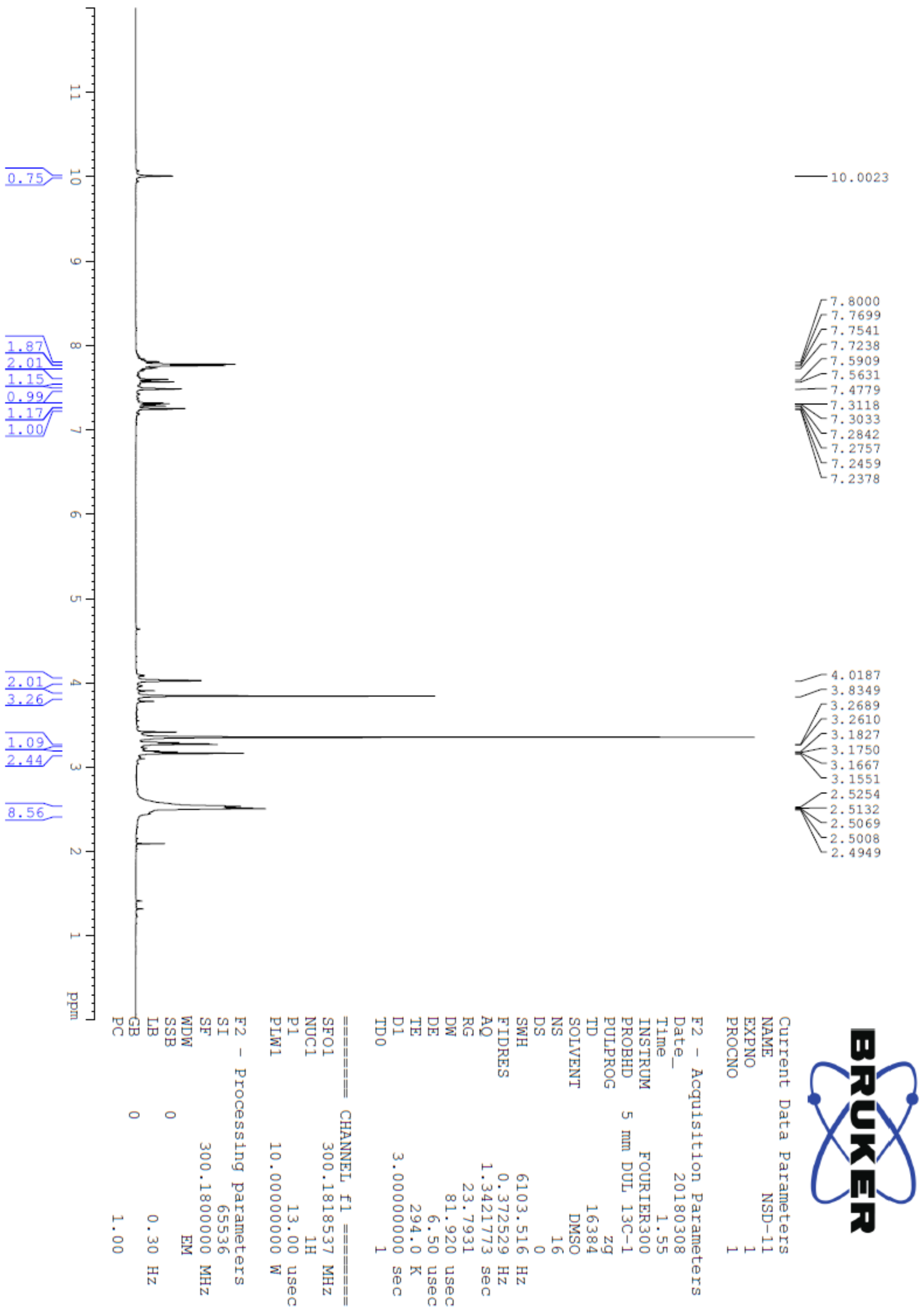

Figure S94. Compound **D11** ^{1}H-NMR spectrum.

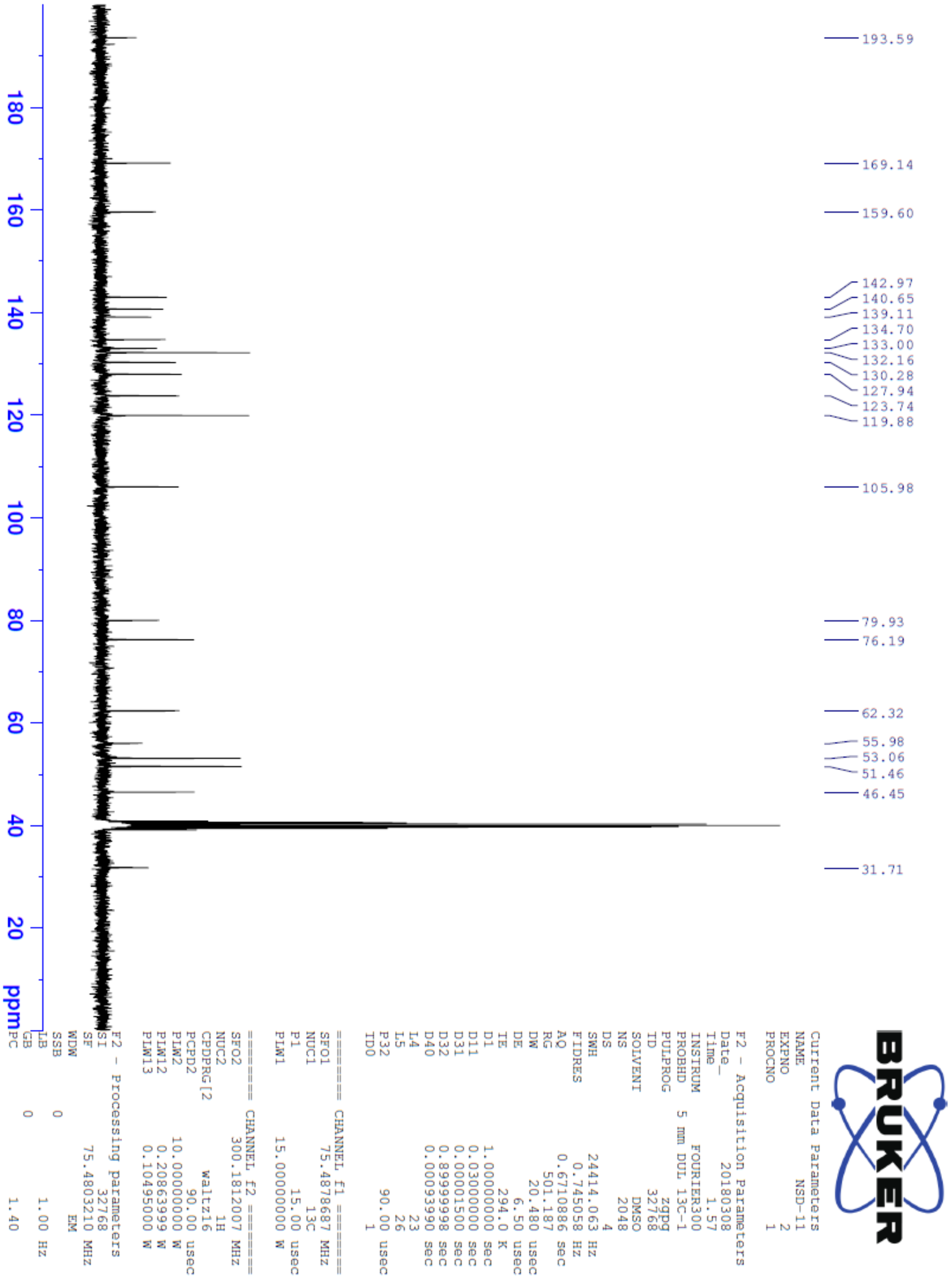

Figure S95. Compound **D11** ^{13}C-NMR spectrum.

Formula Predictor Report - NSD-11_51.lcd Page 1 of 1

Data File: C:\LabSolutions\Data\Analiz\Serkan\NSD-11_51.lcd

Elmt	Val.	Min	Max	Elmt	Val.	Min	Max	Elmt	Val.	Min	Max	Elmt	Val.	Min	Max	Use Adduct
H	1	5	40	O	2	3	5	S	2	0	3	Ru	2	0	0	H
C	4	0	35	F	1	0	0	Cl	1	0	2	I	3	0	0	
N	3	3	6	P	3	0	0	Br	1	0	0					

Error Margin (ppm): 5
HC Ratio: unlimited
Max Isotopes: 3
MSn Iso RI (%): 10.00

DBE Range: 10.0 - 17.0
Apply N Rule: yes
Isotope RI (%): 1.00
MSn Logic Mode: AND

Electron Ions: both
Use MSn Info: yes
Isotope Res: 9000
Max Results: 500

Event#: 1 MS(E+) Ret. Time : 2.120 -> 2.200 Scan# : 319 -> 331

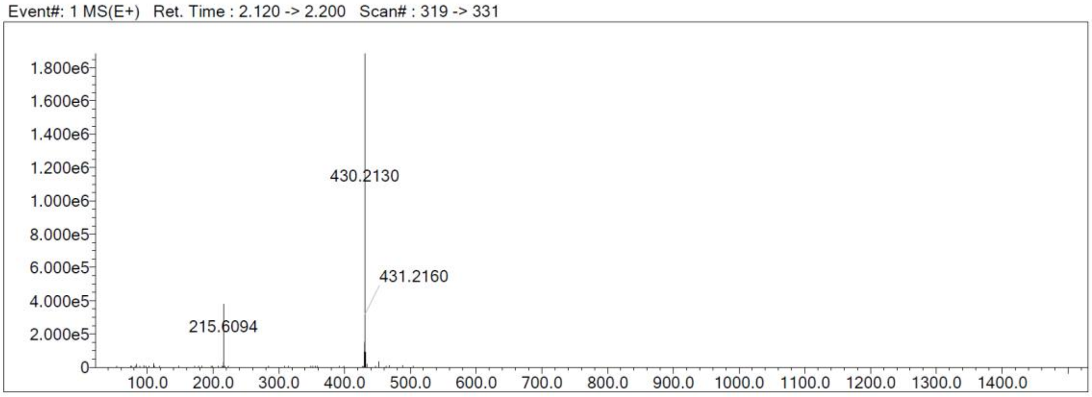

Measured region for 430.2130 m/z

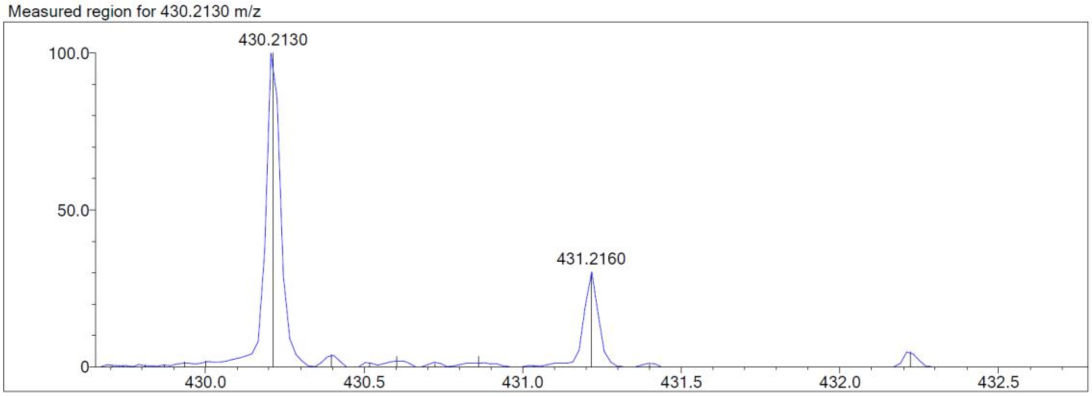

C26 H27 N3 O3 [M+H]+ : Predicted region for 430.2125 m/z

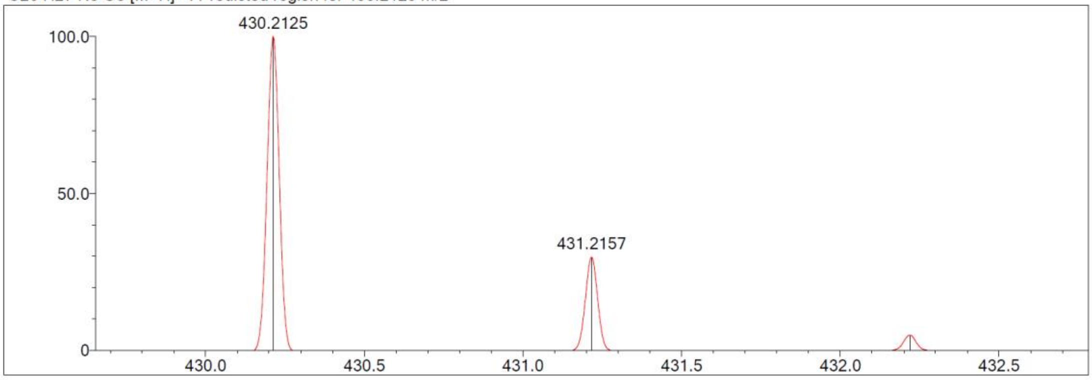

Rank	Score	Formula (M)	Ion	Meas. m/z	Pred. m/z	Df. (mDa)	Df. (ppm)	Iso	DBE
1	93.50	C26 H27 N3 O3	[M+H]+	430.2130	430.2125	0.5	1.16	93.88	15.0

Figure S96. Compound **D11** HRMS report.

*2-(4-Propargylpiperazine-1-yl)-N-(4-((5,6-dimethoxy-1-oxo-2,3-dihydro-1H-inden-2-ylidene) methyl)phenyl)acetamide (**D12**)*

Yellow powder. M.P.: 105.4 °C. Yield: 87%.

IR (ATR) ν_{max} (cm^{-1}): 3332 (N-H), 1695 (indanone C=O), 1625 (amide C=O), 1519-1498 (C=C), 1220 (C-N), 1118 (C-O), 831 (1,4-disubstituted benzene).

^{1}H-NMR (300 MHz, DMSO-*d_6*) δ (ppm): 2.54 (8H, bs, piperazine CH_2), 3.16-3.17 (3H, m, CH, CH_2), 3.27 (1H, d, *J*=2.43 Hz, CH_2) 3.84 (3H, s, OCH_3), 3.91 (3H, s, OCH_3), 3.98 (2H, s, CH_2), 7.21 (1H, s, methoxy-1-oxo-indenylidene CH), 7.22 (1H, s, methoxy-1-oxo-indenylidene CH), 7.38 (1H, s, C=CH), 7.71 (2H, d, *J*=8.80 Hz, disubstituted benzene CH), 7.78 (2H, d, *J*=8.80 Hz, disubstituted benzene CH), 9.97 (1H, s, NH).

^{13}C-NMR (75 MHz, DMSO-*d_6*) δ (ppm): 32.1, 46.5, 51.5, 53.1, 56.1, 56.5, 62.3, 76.2, 79.9, 105.0, 108.5, 119.9, 130.5, 130.6, 131.3, 131.8, 134.8, 140.3, 145.4, 149.7, 155.6, 169.1, 192.3.

HRMS (ESI) (m/z) $[M+H]^+$: $C_{27}H_{29}N_3O_4$ calculated: 460.2231, found: 460.2249.

DOPNALAB

	Item	Value
	Acquired Date&Time	22.08.2019 11:42:24
	Acquired by	System Administrator
	Filename	C:\Users\dopnalab\Desktop\NURPELİN\DOKTORA TEZ\D121.ispd
	Spectrum name	D121
	Sample name	D12
	Sample ID	
	Option	
	Comment	
	No. of Scans	50
	Resolution	4 [cm-1]
	Apodization	Happ-Genzel

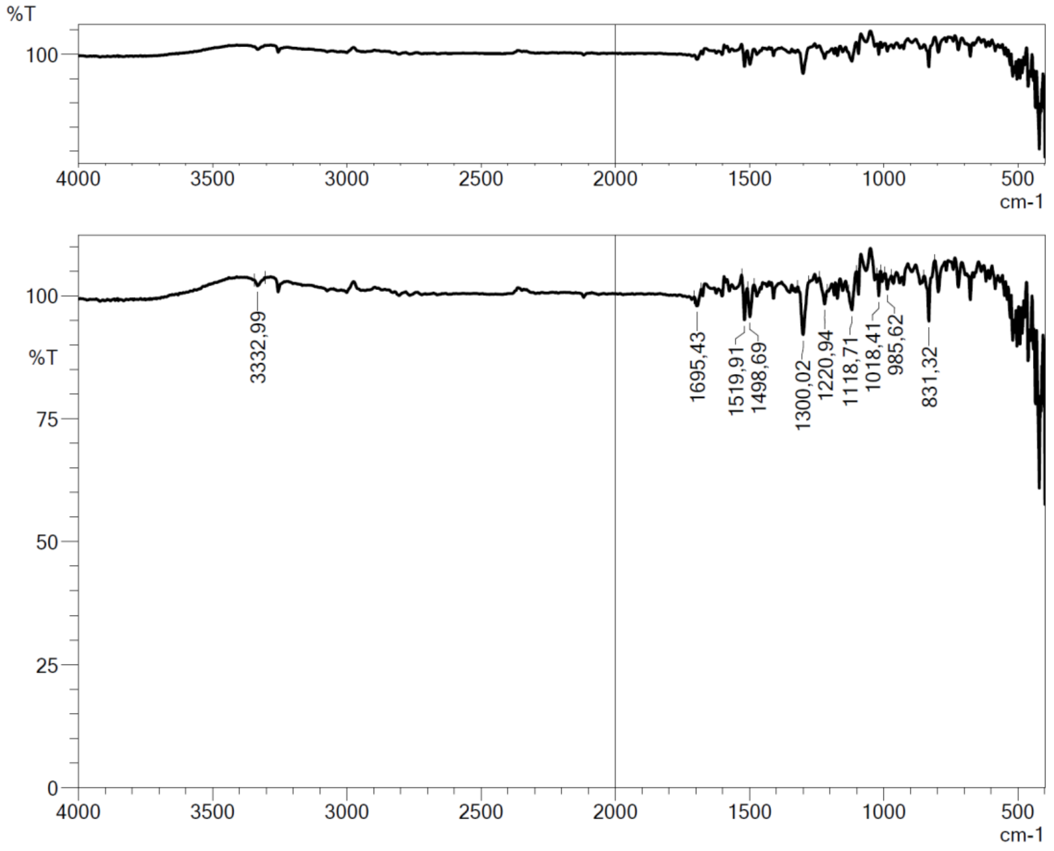

Figure S97. Compound **D12** IR report.

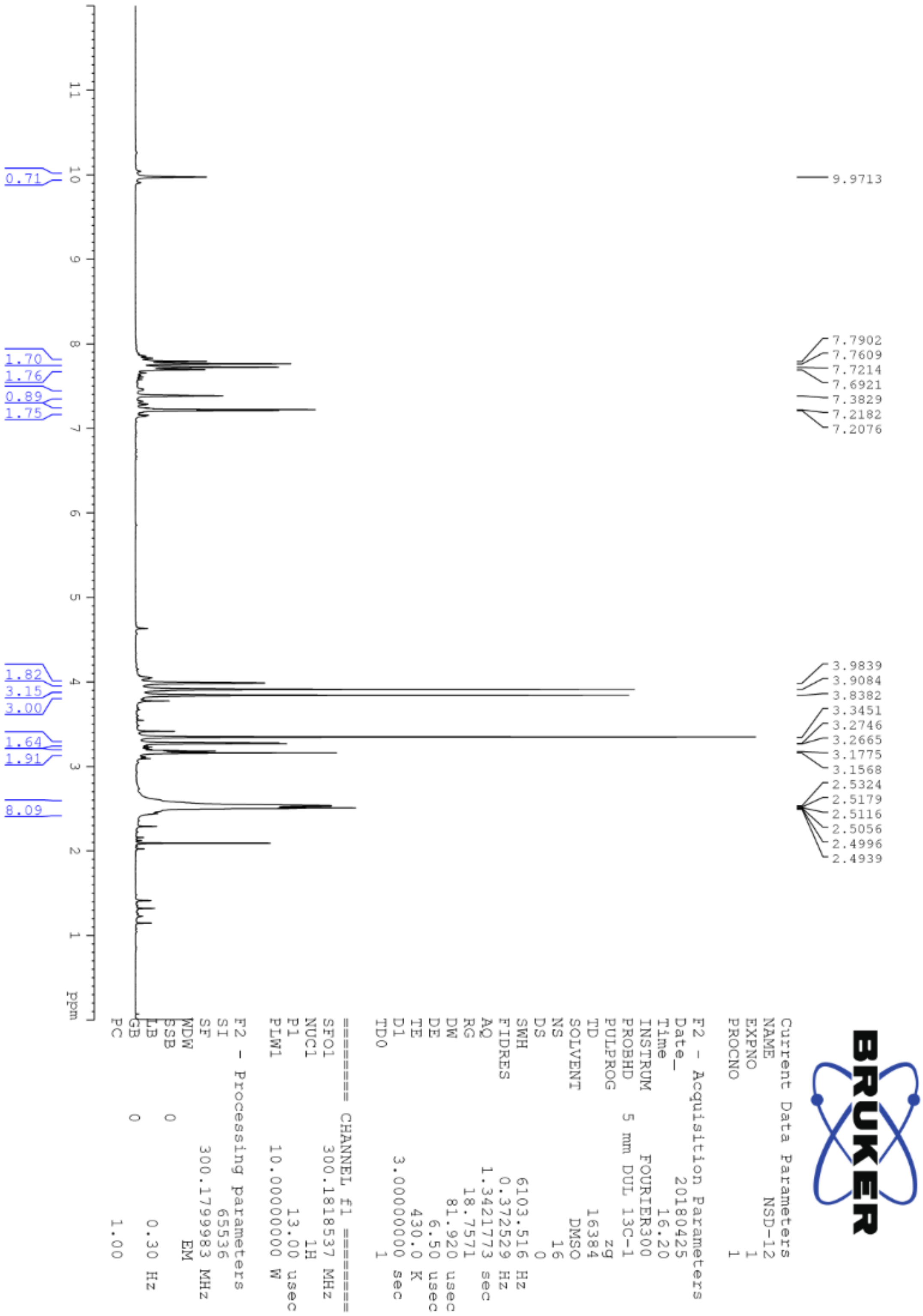

Figure S98. Compound **D12** ^{1}H-NMR spectrum.

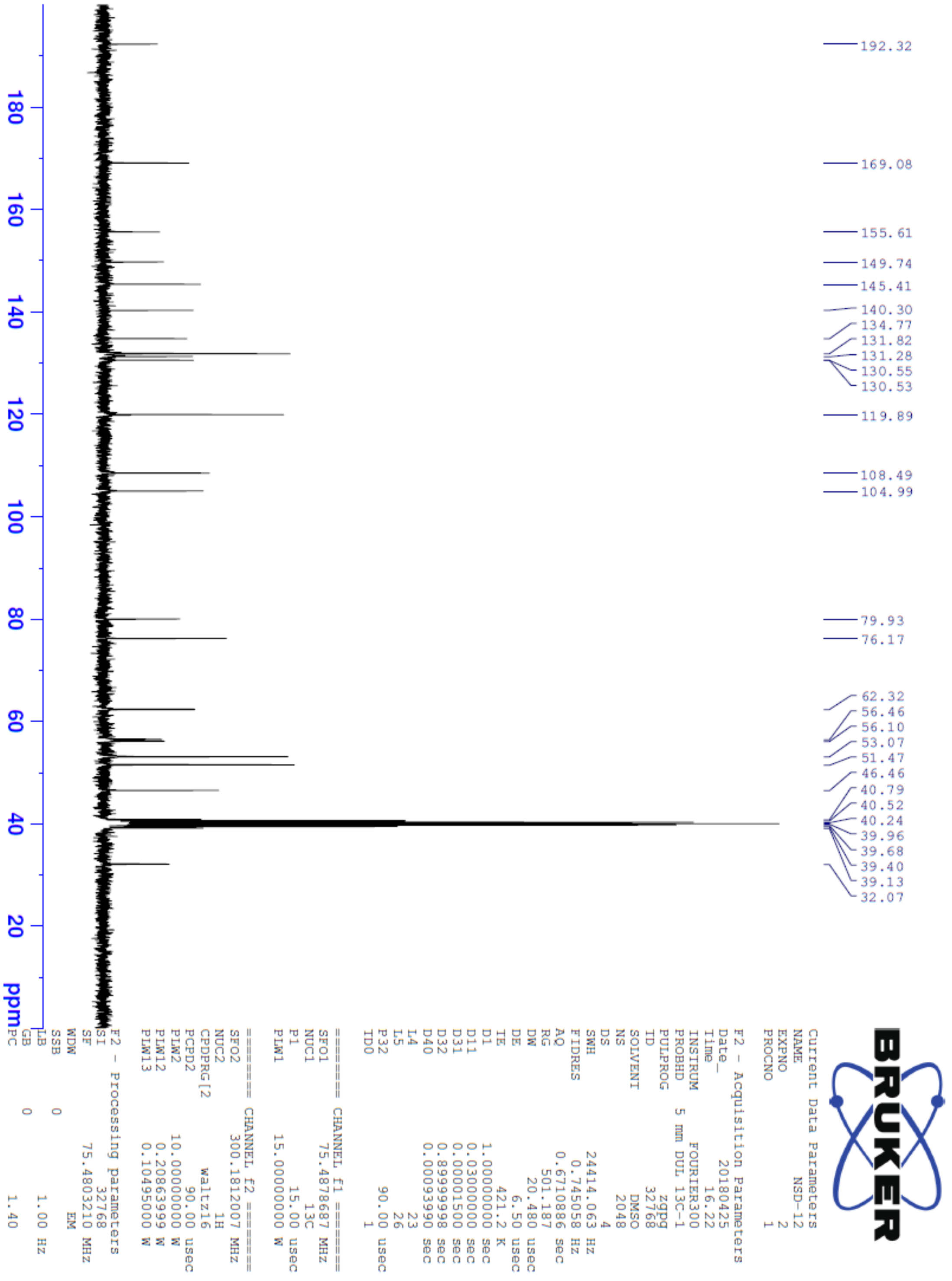

Figure S99. Compound **D12** ^{13}C-NMR spectrum.

Data File: C:\LabSolutions\Data\Analiz\Serkan\NSD-12_52.lcd

Elmt	Val.	Min	Max	Elmt	Val.	Min	Max	Elmt	Val.	Min	Max	Elmt	Val.	Min	Max	Use Adduct
H	1	5	40	O	2	3	5	S	2	0	0	Ru	2	0	0	H
C	4	0	35	F	1	0	0	Cl	1	0	0	I	3	0	0	
N	3	3	6	P	3	0	0	Br	1	0	0					

Error Margin (ppm): 5
HC Ratio: unlimited
Max Isotopes: 3
MSn Iso RI (%): 10.00

DBE Range: 10.0 - 17.0
Apply N Rule: yes
Isotope RI (%): 1.00
MSn Logic Mode: AND

Electron Ions: both
Use MSn Info: yes
Isotope Res: 9000
Max Results: 500

Event#: 1 MS(E+) Ret. Time : 2.200 -> 2.333 Scan# : 331 -> 351

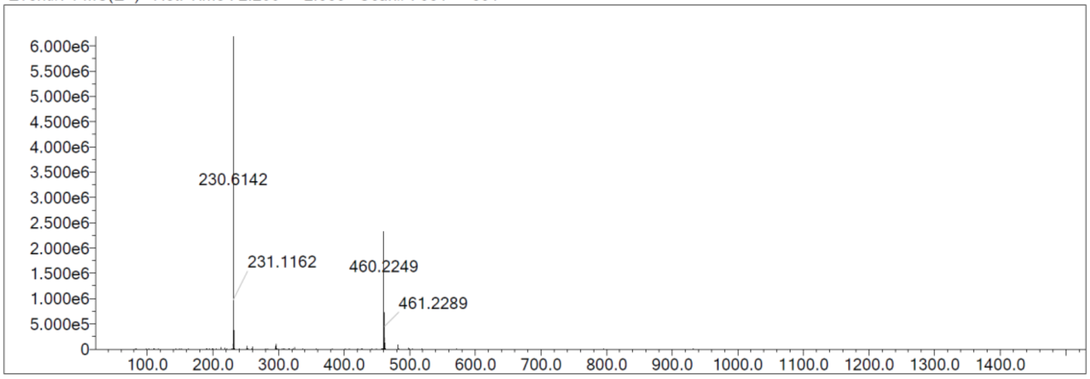

Measured region for 460.2249 m/z

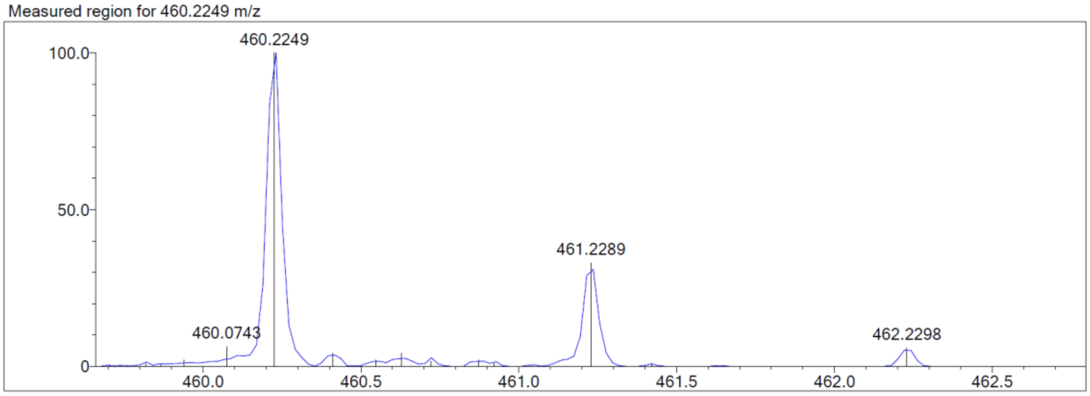

C27 H29 N3 O4 [M+H]+ : Predicted region for 460.2231 m/z

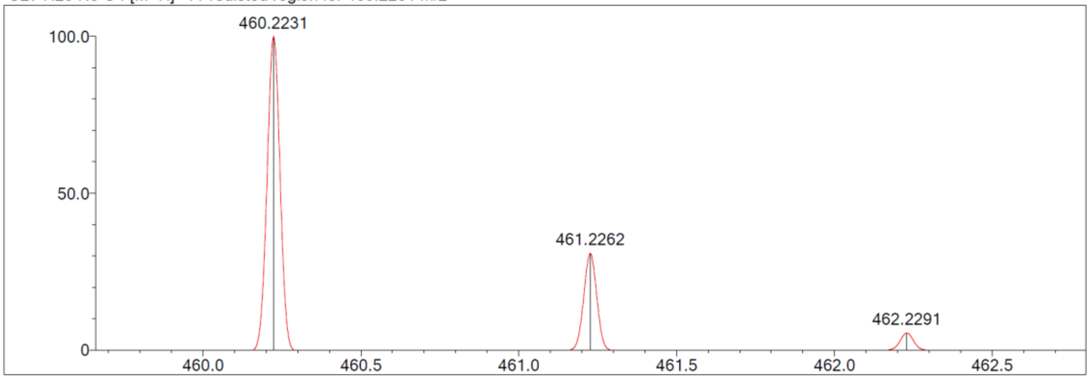

Rank	Score	Formula (M)	Ion	Meas. m/z	Pred. m/z	Df. (mDa)	Df. (ppm)	Iso	DBE
1	84.00	C27 H29 N3 O4	[M+H]+	460.2249	460.2231	1.8	3.91	90.60	15.0

Figure S100. Compound **D12** HRMS report.

*2-(4-(2-Methoxyethyl)piperazine-1-yl)-N-(4-((5-methoxy-1-oxo-2,3-dihydro-1H-inden-2-ylidene)methyl)phenyl) acetamide (**D13**)*

Dark brown powder. M.P.: 198.5 °C. Yield: 85%.

IR (ATR) ν_{max} (cm^{-1}): 3350 (N-H), 1693 (indanone C=O), 1633 (amide C=O), 1539-1498 (C=C), 1269 (C-N), 1103 (C-O), 840 (1,4-disubstituted benzene).

^{1}H-NMR (300 MHz, DMSO-d_6) δ (ppm): 2.54 (10H, bs, CH_2, piperazine CH_2), 3.14 (2H, m, CH_2), 3.42 (2H, t, J=5.82 Hz, CH_2), 3.89 (3H, s, OCH_3), 4.05 (2H, s, CH_2), 7.02 (1H, dd, J_1=8.46 Hz, J_2=1.71 Hz, methoxy-1-oxo-indenylidene CH), 7.17 (1H, s, methoxy-1-oxo-indenylidene CH), 7.40 (1H, s, C=CH), 7.70-7.73 (3H, m, disubstituted benzene CH, methoxy-1-oxo-indenylidene CH), 7.79 (2H, d, J=8.58 Hz, disubstituted benzene CH), 9.95 (1H, s, NH).

^{13}C-NMR (75 MHz, DMSO-d_6) δ (ppm): 32.5, 53.3, 53.4, 56.3, 57.5, 58.5, 62.4, 70.4, 110.6, 115.8, 119.9, 125.8, 130.5, 131.1, 131.7, 131.9, 134.5, 140.3, 153.3, 165.3, 169.1, 192.0.

HRMS (ESI) (m/z) [M+H]$^+$: $C_{26}H_{31}N_3O_4$ calculated: 450.2387, found: 450.2396.

DOPNALAB

	Item	Value
	Acquired Date&Time	22.08.2019 11:45:16
	Acquired by	System Administrator
	Filename	C:\Users\dopnalab\Desktop\NURPELİN\DOKTORA TEZ\D131.ispd
	Spectrum name	D131
	Sample name	D13
	Sample ID	
	Option	
	Comment	
	No. of Scans	50
	Resolution	4 [cm-1]
	Apodization	Happ-Genzel

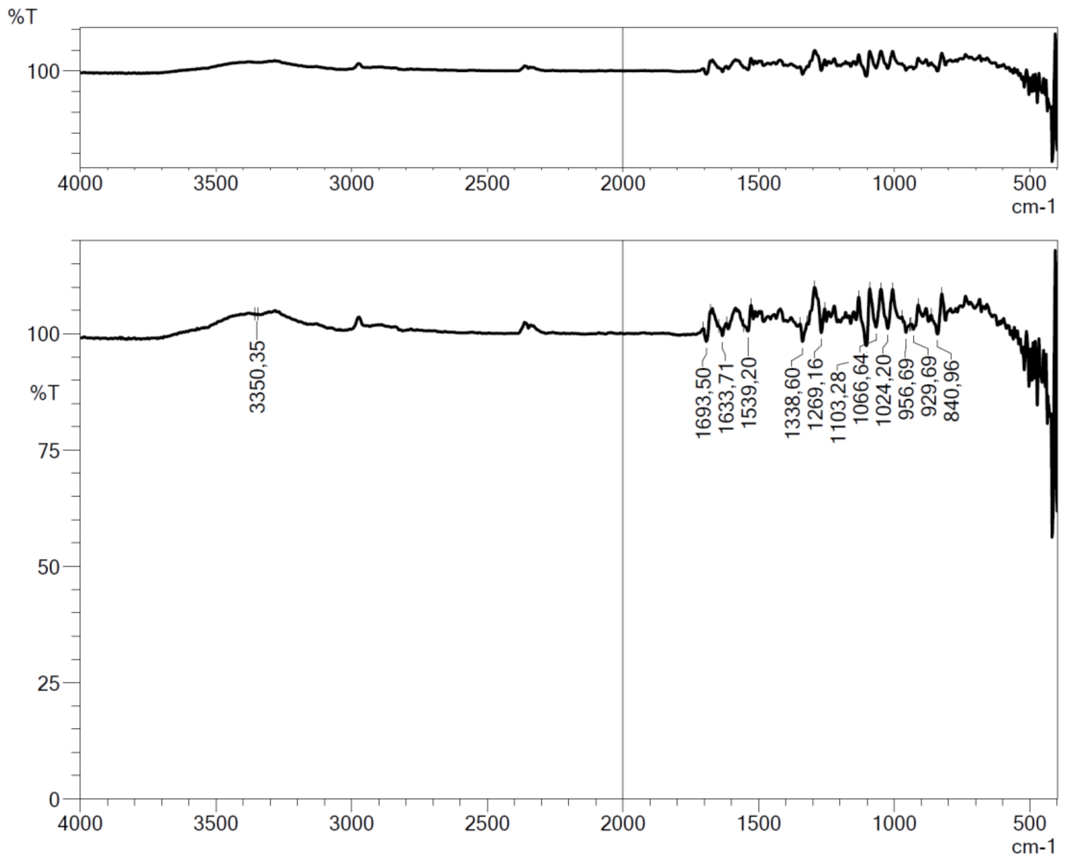

Figure S101. Compound **D13** IR report.

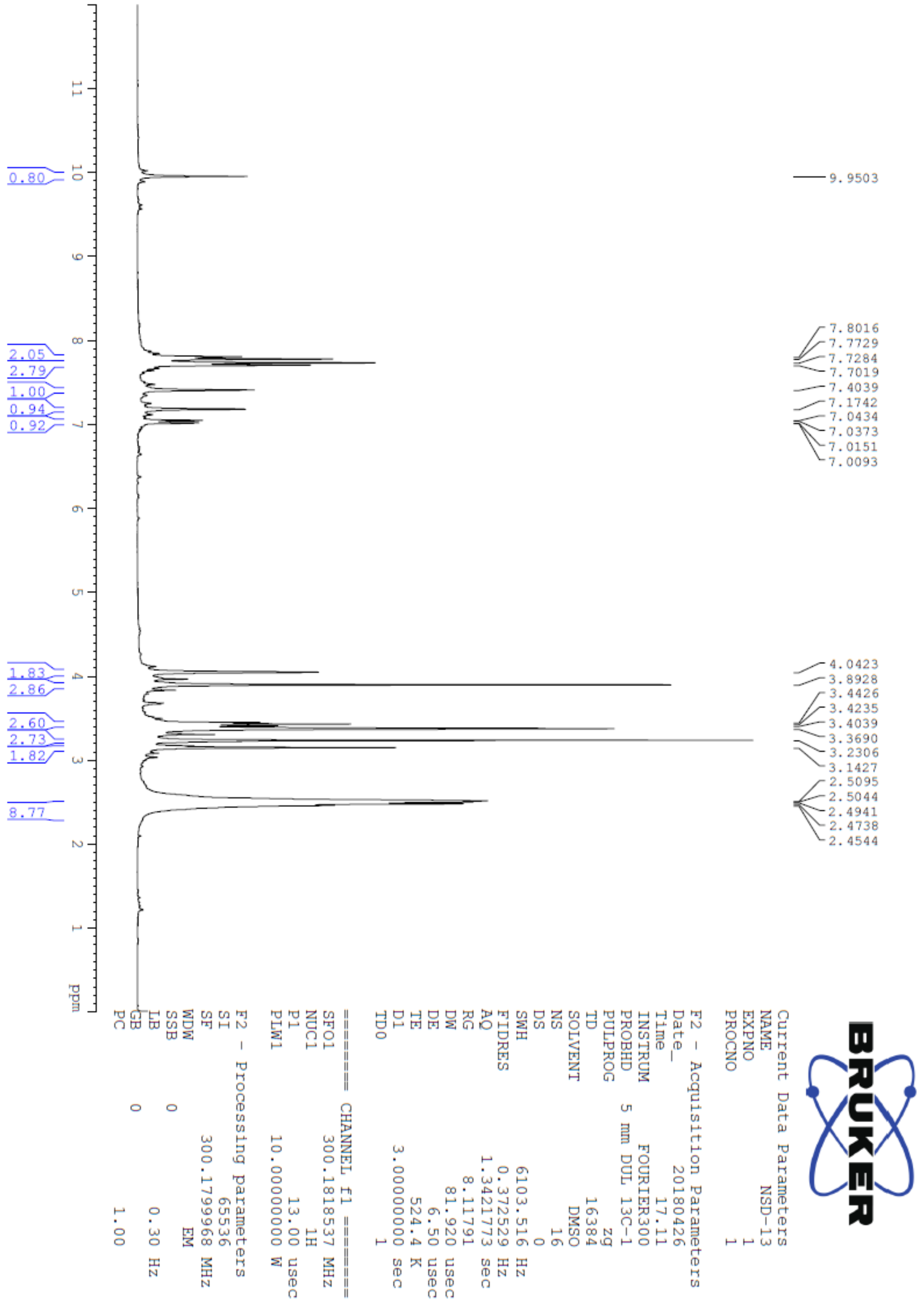

Figure S102. Compound **D13** ^{1}H-NMR spectrum.

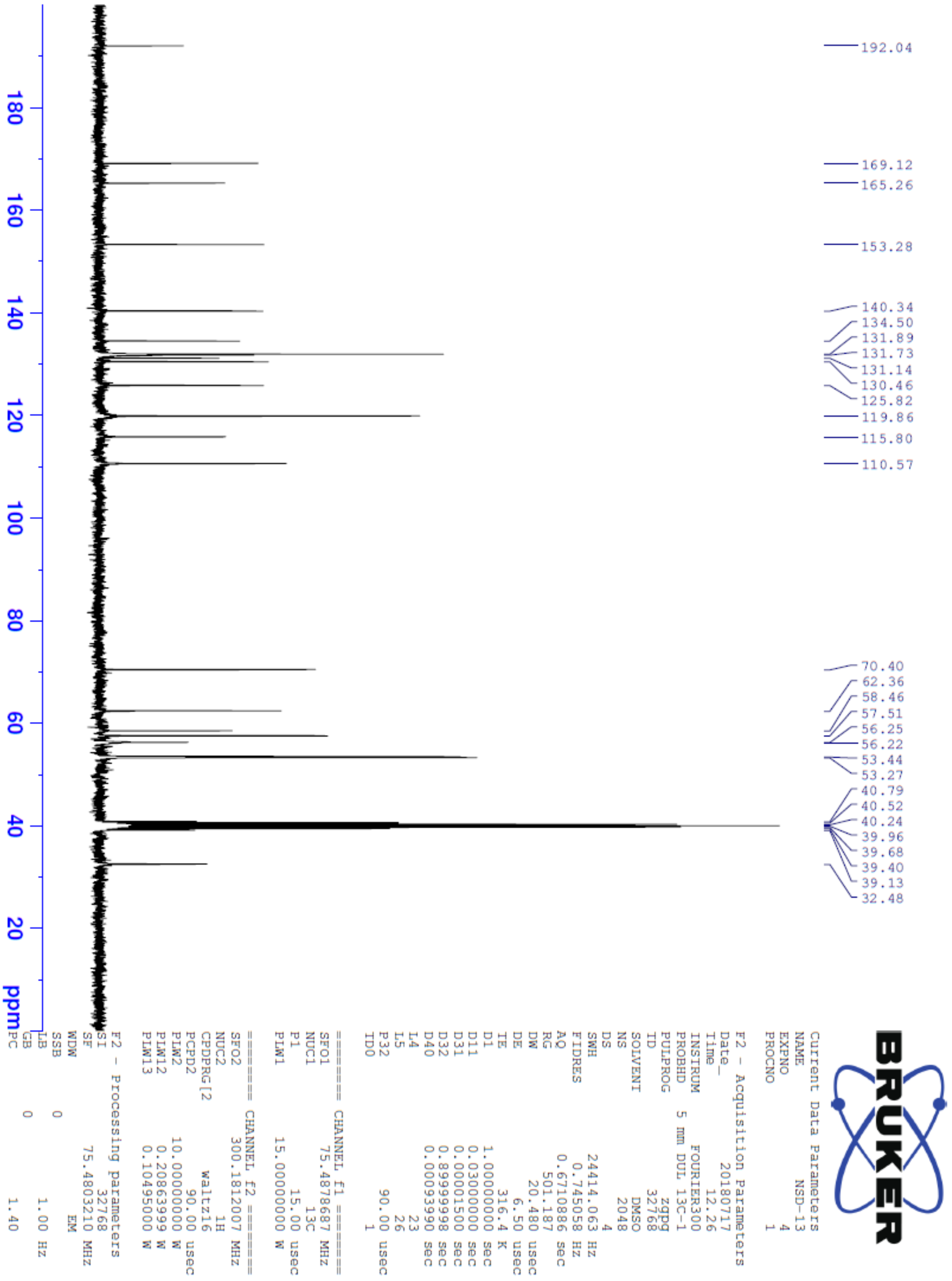

Figure S103. Compound **D13** ^{13}C-NMR spectrum.

Formula Predictor Report - NSD-13_5.lcd Page 1 of 1

Data File: C:\LabSolutions\Data\Analiz\bns\NSD-13_5.lcd

Elmt	Val.	Min	Max	Elmt	Val.	Min	Max	Elmt	Val.	Min	Max	Elmt	Val.	Min	Max	Use Adduct
H	1	0	50	O	2	3	8	S	2	0	1	Ru	2	0	0	H
C	4	0	50	F	1	0	0	Cl	1	0	1	Pd	2	0	0	
N	3	0	4	P	3	0	0	Br	1	0	0	I	3	0	0	

Error Margin (ppm): 5
HC Ratio: unlimited
Max Isotopes: 3
MSn Iso RI (%): 10.00

DBE Range: 10.0 - 30.0
Apply N Rule: yes
Isotope RI (%): 1.00
MSn Logic Mode: AND

Electron Ions: both
Use MSn Info: yes
Isotope Res: 9000
Max Results: 500

Event#: 1 MS(E+) Ret. Time : 6.053 -> 6.827 Scan# : 909 -> 1025

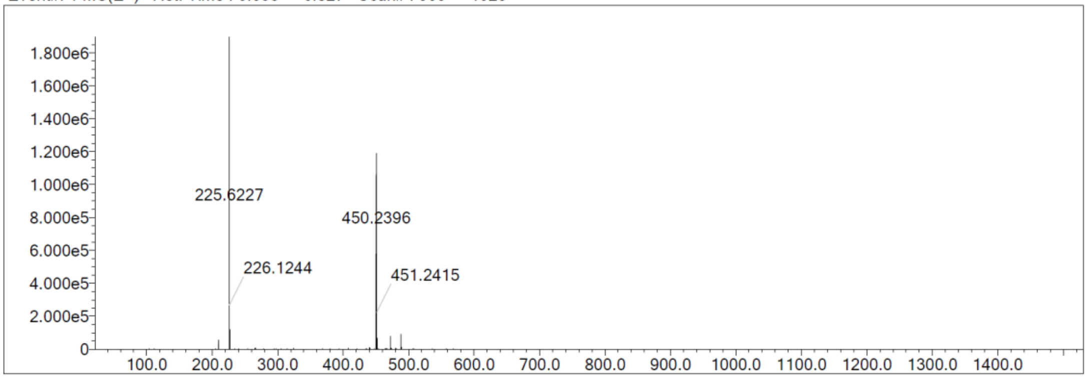

Measured region for 450.2396 m/z

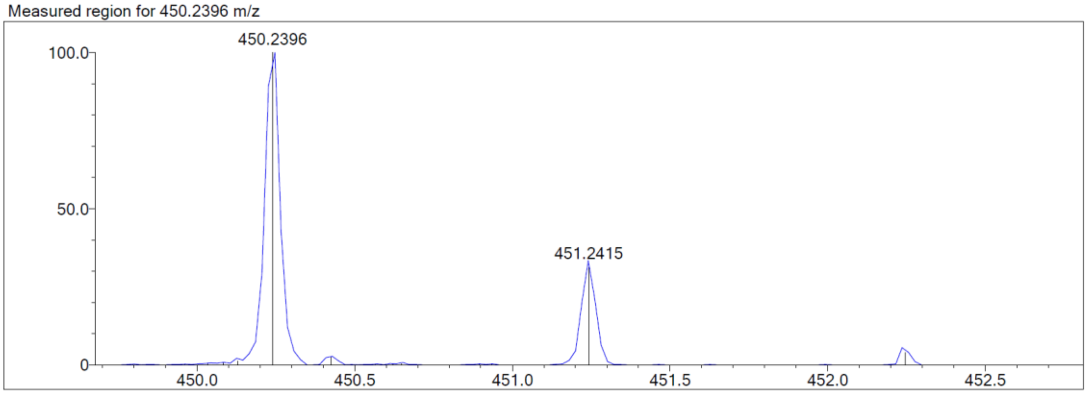

C26 H31 N3 O4 [M+H]+ : Predicted region for 450.2387 m/z

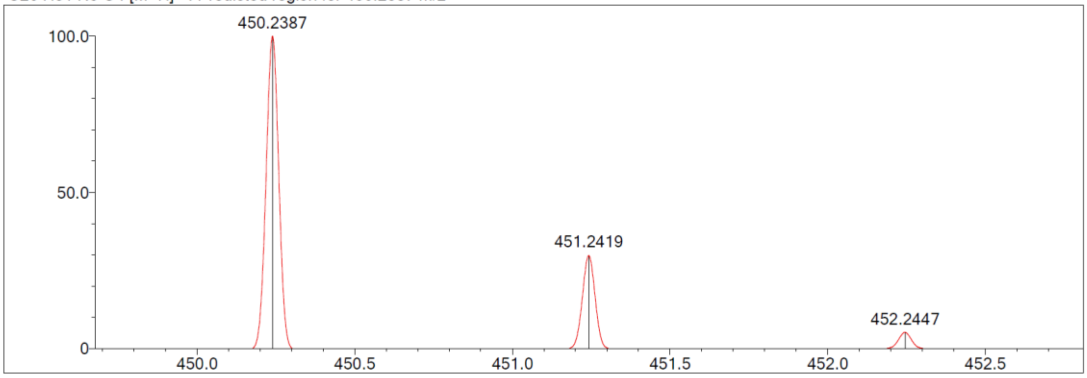

Rank	Score	Formula (M)	Ion	Meas. m/z	Pred. m/z	Df. (mDa)	Df. (ppm)	Iso	DBE
1	89.32	C26 H31 N3 O4	[M+H]+	450.2396	450.2387	0.9	2.00	91.61	13.0

Figure S104. Compound **D13** HRMS report.

*2-(4-(2-Methoxyethyl)piperazine-1-yl)-N-(4-((6-methoxy-1-oxo-2,3-dihydro-1H-inden-2-ylidene)methyl)phenyl) acetamide (**D14**)*

Light brown powder. M.P.: 119.7 °C. Yield: 86%.

IR (ATR) ν_{max} (cm^{-1}): 3327 (N-H), 1697 (indanone C=O), 1614 (amide C=O), 1487-1411 (C=C), 1195 (C-N), 1109 (C-O), 842 (1,4-disubstituted benzene).

^{1}H-NMR (300 MHz, DMSO-d_6) δ (ppm): 2.54 (10H, bs, CH_2, piperazine CH_2), 3.14 (2H, s, CH_2), 3.22 (2H, s, OCH_3), 3.42 (2H, t, J=5.88 Hz, CH_2), 3.83 (3H, s, OCH_3), 4.01 (2H, s, CH_2), 7.24 (1H, d, J=2.49 Hz, methoxy-1-oxo-indenylidene CH), 7.29 (1H, dd, J_1=8.31 Hz, J_2=2.58 Hz, methoxy-1-oxo-indenylidene CH), 7.48 (1H, s, C=CH), 7.57 (1H, d, J=8.37 Hz, methoxy-1-oxo-indenylidene CH), 7.74 (2H, d, J=8.58 Hz, disubstituted benzene CH), 7.79 (2H, d, J=8.97 Hz, disubstituted benzene CH), 9.97 (1H, s, NH).

^{13}C-NMR (75 MHz, DMSO-d_6) δ (ppm): 31.7, 53.3, 53.4, 56.0, 57.5, 58.5, 62.4, 70.4, 106.0, 119.9, 123.7, 127.9, 130.3, 132.2, 133.0, 134.7, 139.1, 140.6, 143.0, 159.6, 169.2, 193.6.

HRMS (ESI) (m/z) $[M+H]^+$: $C_{26}H_{31}N_3O_4$ calculated: 450.2387, found: 450.2396.

DOPNALAB

	Item	Value
	Acquired Date&Time	22.08.2019 11:47:53
	Acquired by	System Administrator
	Filename	C:\Users\dopnalab\Desktop\NURPELİN\DOKTORA TEZ\D141.ispd
	Spectrum name	D141
	Sample name	D14
	Sample ID	
	Option	
	Comment	
	No. of Scans	50
	Resolution	4 [cm-1]
	Apodization	Happ-Genzel

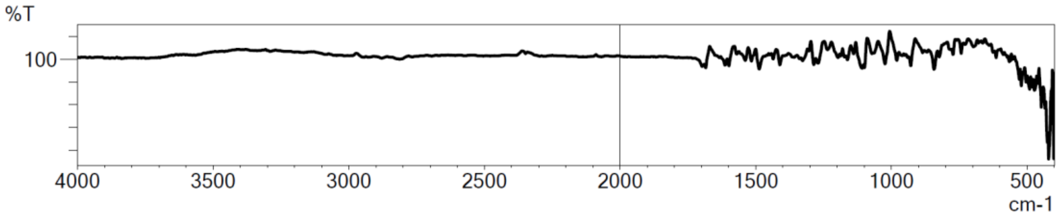

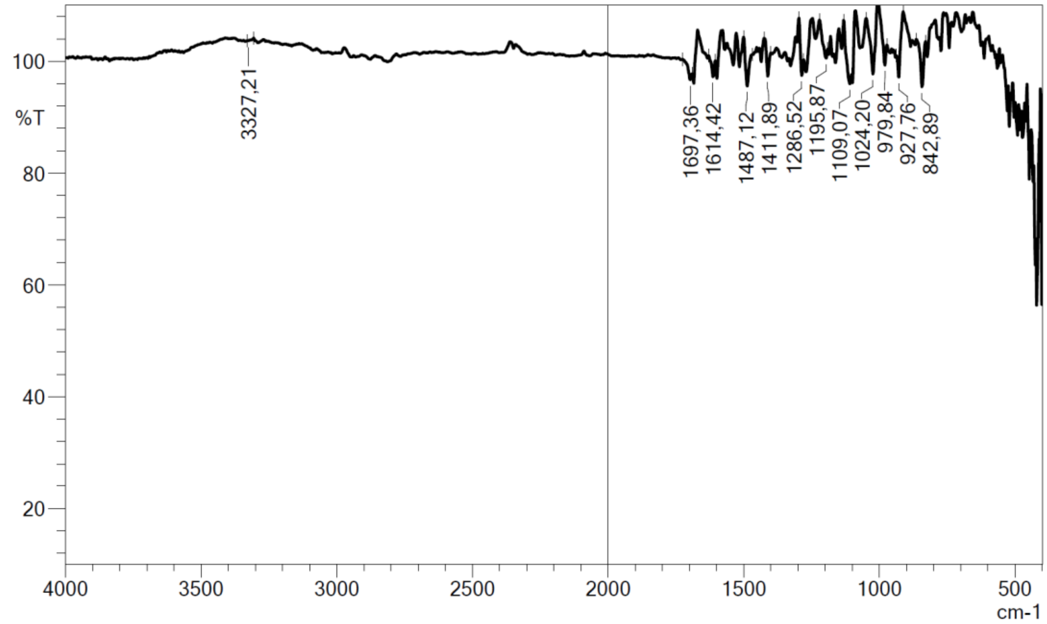

Figure S105. Compound **D14** IR report.

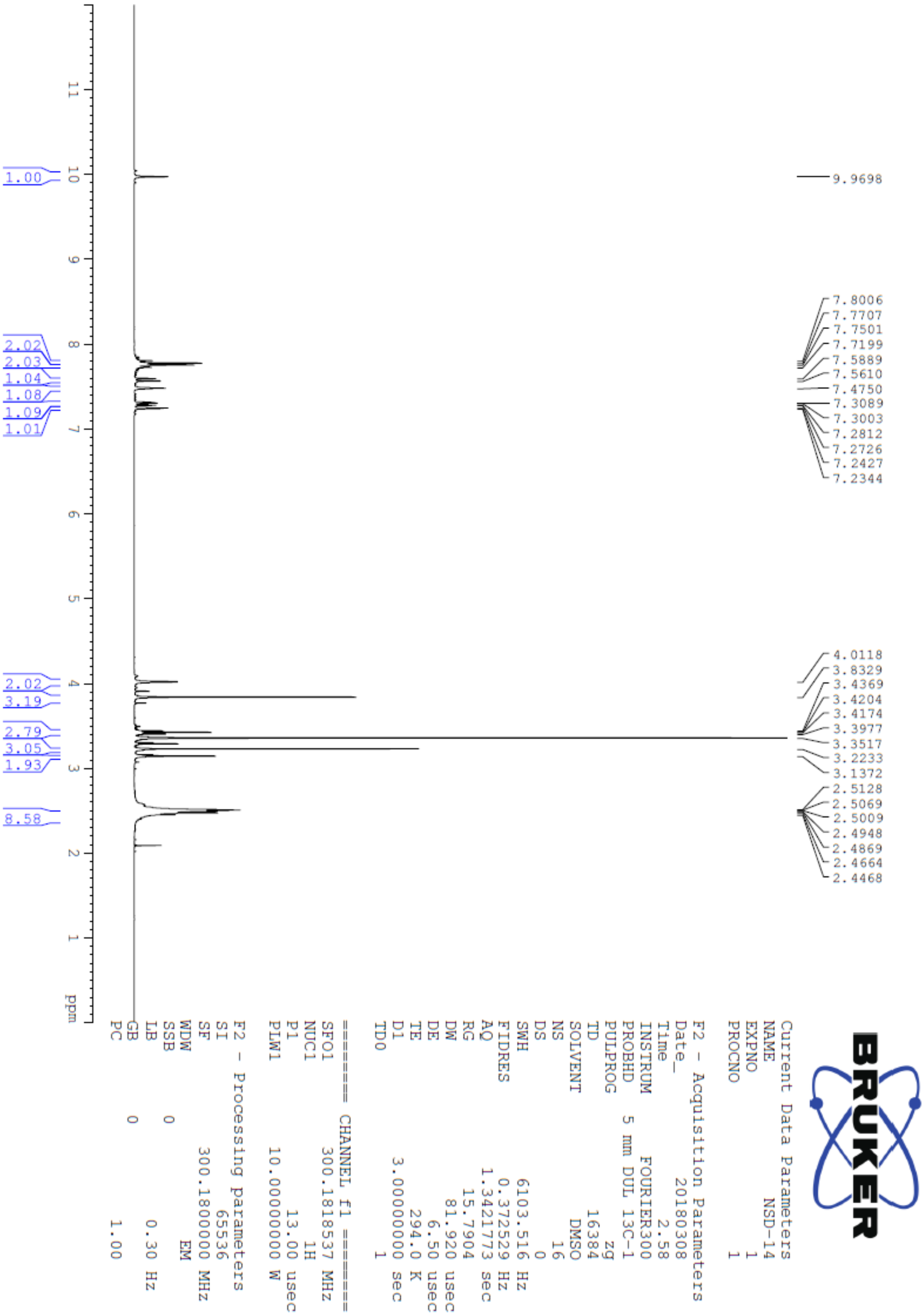

Figure S106. Compound **D14** ^{1}H-NMR spectrum.

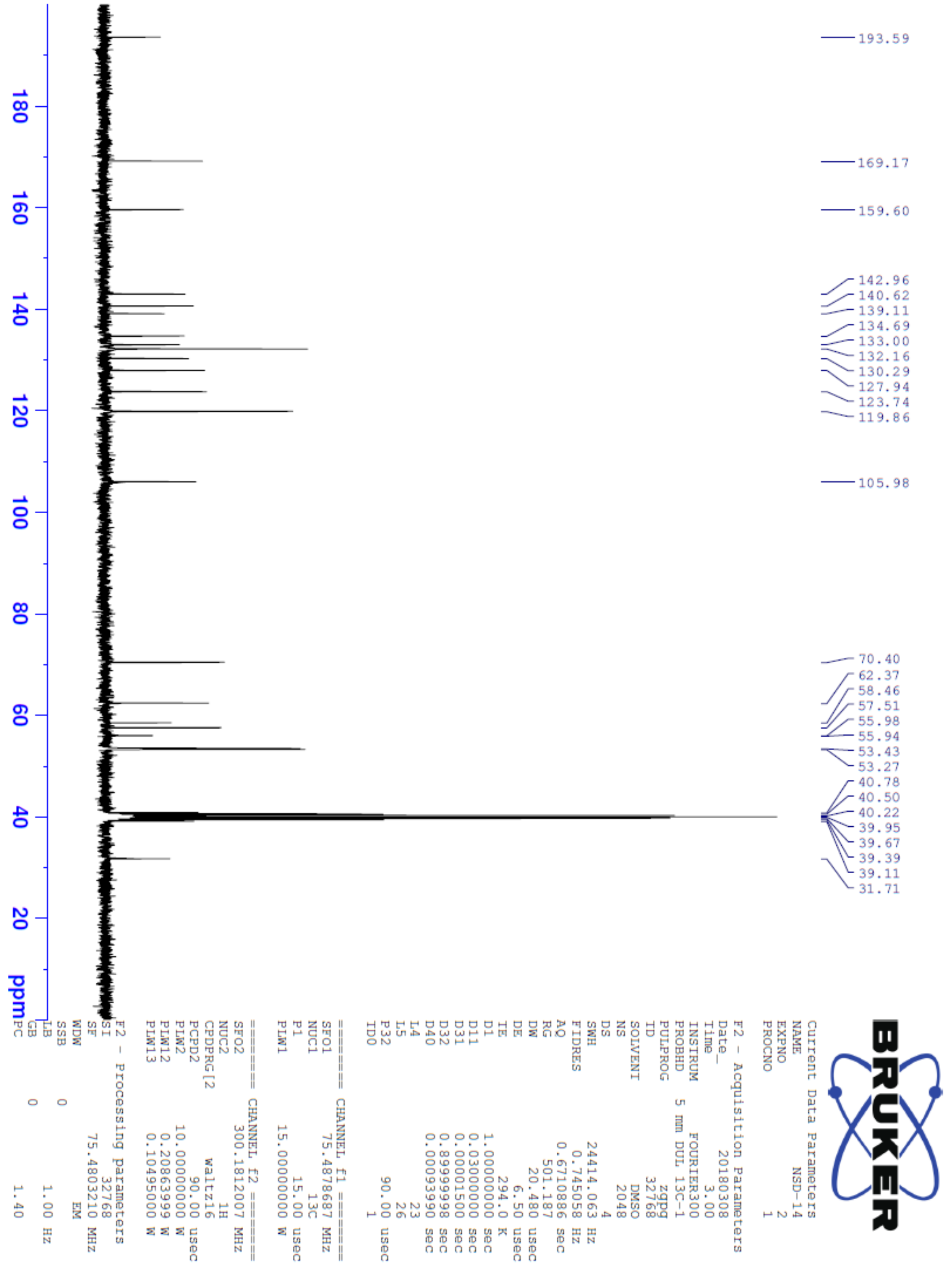

Figure S107. Compound **D14** ^{13}C-NMR spectrum.

Data File: C:\LabSolutions\Data\Analiz\Serkan\NSD-14_53.lcd

Elmt	Val.	Min	Max	Elmt	Val.	Min	Max	Elmt	Val.	Min	Max	Elmt	Val.	Min	Max	Use Adduct
H	1	5	40	O	2	3	5	S	2	0	0	Ru	2	0	0	H
C	4	0	35	F	1	0	0	Cl	1	0	0	I	3	0	0	
N	3	3	6	P	3	0	0	Br	1	0	0					

Error Margin (ppm):	5	DBE Range:	10.0 - 17.0	Electron Ions:	both
HC Ratio:	unlimited	Apply N Rule:	yes	Use MSn Info:	yes
Max Isotopes:	3	Isotope RI (%):	1.00	Isotope Res:	9000
MSn Iso RI (%):	10.00	MSn Logic Mode:	AND	Max Results:	500

Event#: 1 MS(E+) Ret. Time : 2.213 -> 2.467 Scan# : 333 -> 371

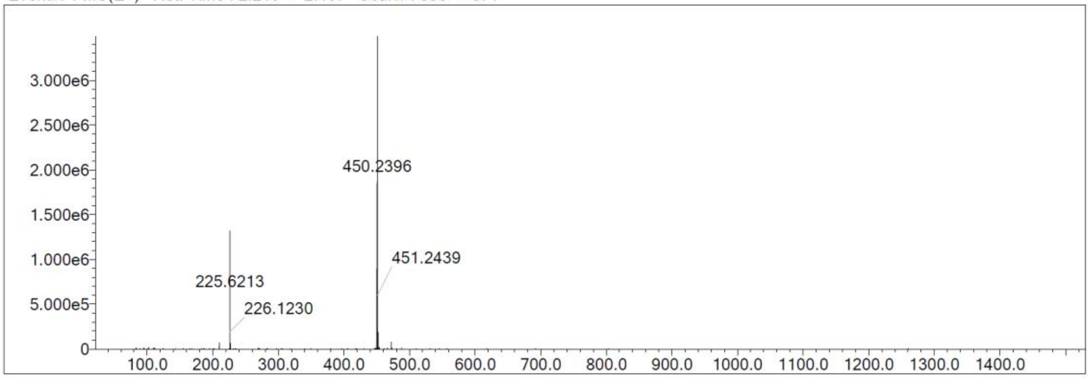

Measured region for 450.2396 m/z

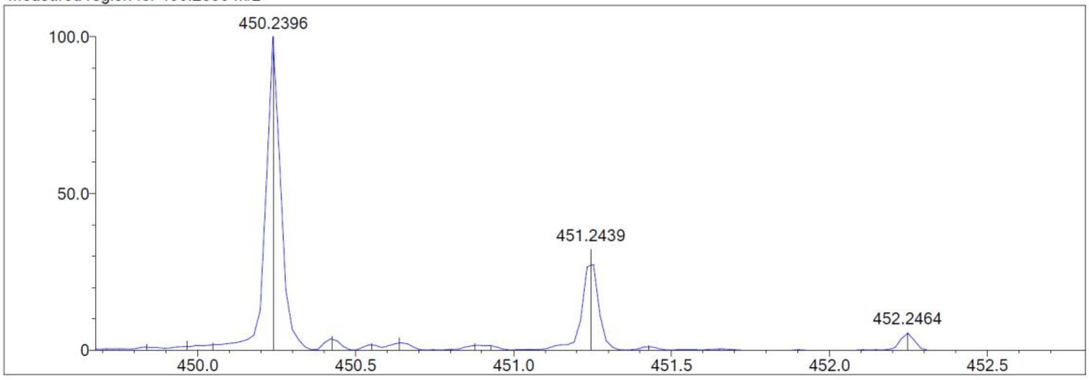

C26 H31 N3 O4 [M+H]+ : Predicted region for 450.2387 m/z

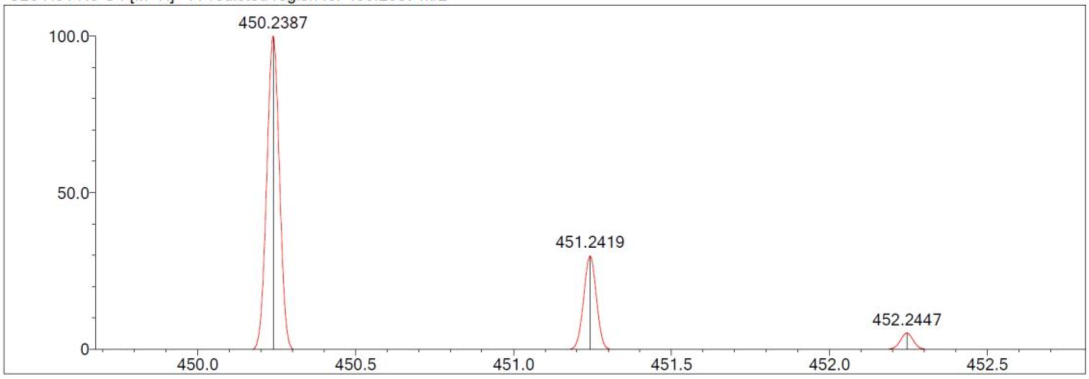

Rank	Score	Formula (M)	Ion	Meas. m/z	Pred. m/z	Df. (mDa)	Df. (ppm)	Iso	DBE
1	97.50	C26 H31 N3 O4	[M+H]+	450.2396	450.2387	0.9	2.00	100.00	13.0

Figure S108. Compound **D14** HRMS report.

*2-(4-(2-Methoxyethyl)piperazine-1-yl)-N-(4-((5,6-dimethoxy-1-oxo-2,3-dihydro-1H-inden-2-ylidene)methyl) phenyl)acetamide (**D15**)*

Yellow powder. M.P.: 124.7 °C. Yield: 78%.

IR (ATR) ν_{max} (cm^{-1}): 3342 (N-H), 1734 (indanone C=O), 1647 (amide C=O), 1533-1471 (C=C), 1250 (C-N), 1114 (C-O), 844 (1,4-disubstituted benzene).

^{1}H-NMR (300 MHz, DMSO-d_6) δ (ppm): 2.54 (10H, bs, CH_2, piperazine CH_2), 3.14 (2H, s, CH_2), 3.23 (3H, s, OCH_3), 3.42 (2H, t, *J*=5.84 Hz, CH_2), 3.84 (3H, s, OCH_3), 3.91 (3H, s, OCH_3), 3.98 (2H, s, CH_2), 7.20 (1H, s, methoxy-1-oxo-indenylidene CH), 7.21 (1H, s, methoxy-1-oxo-indenylidene CH), 7.38 (1H, s, C=CH), 7.70 (2H, d, *J*=8.56 Hz, disubstituted benzene CH), 7.78 (2H, d, *J*=8.80 Hz, disubstituted benzene CH), 9.94 (1H, s, NH).

^{13}C-NMR (75 MHz, DMSO-d_6) δ (ppm): 32.1, 53.3, 53.4, 56.0, 56.4, 57.5, 58.5, 62.4, 70.4, 105.0, 108.5, 119.9, 130.5, 130.6, 131.3, 131.8, 134.8, 140.3, 145.4, 149.7, 155.6, 169.1, 192.3.

HRMS (ESI) (m/z) $[M+H]^+$: $C_{27}H_{33}N_3O_5$ calculated: 480.2493, found: 480.2505.

DOPNALAB

	Item	Value
	Acquired Date&Time	22.08.2019 11:50:30
	Acquired by	System Administrator
	Filename	C:\Users\dopnalab\Desktop\NURPELİN\DOKTORA TEZ\D151.ispd
	Spectrum name	D151
	Sample name	D15
	Sample ID	
	Option	
	Comment	
	No. of Scans	50
	Resolution	4 [cm-1]
	Apodization	Happ-Genzel

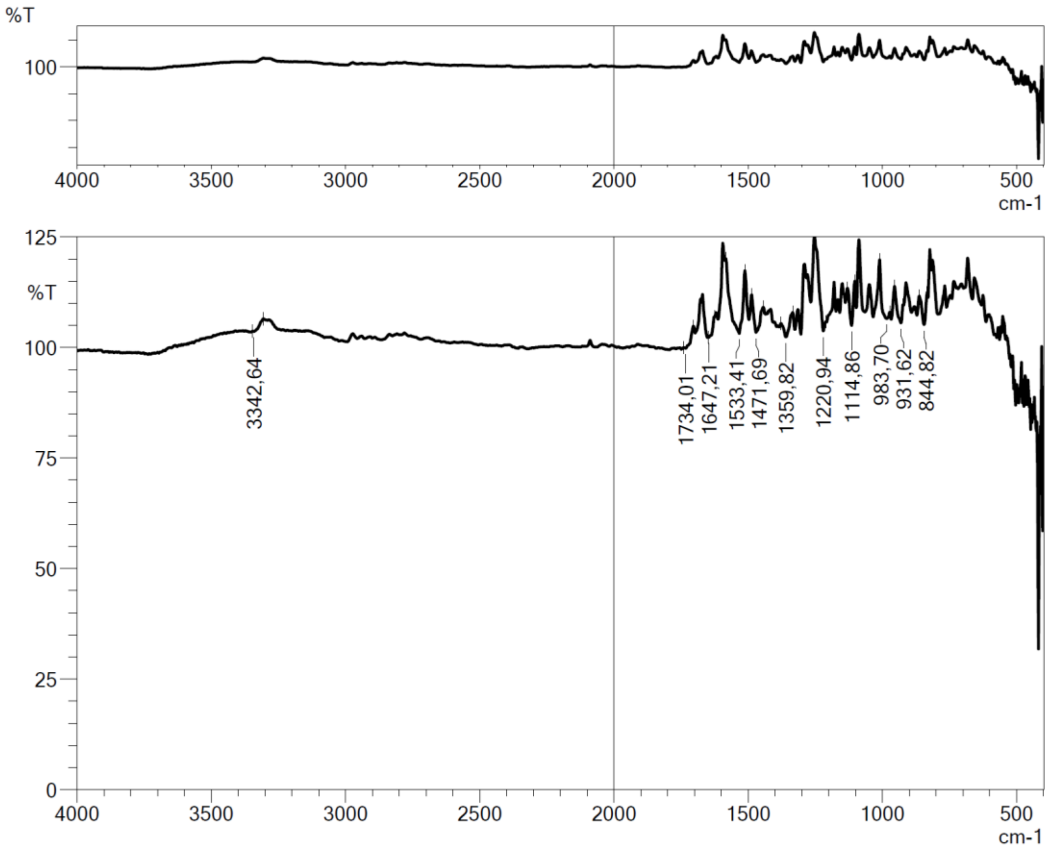

Figure S109. Compound **D15** IR report.

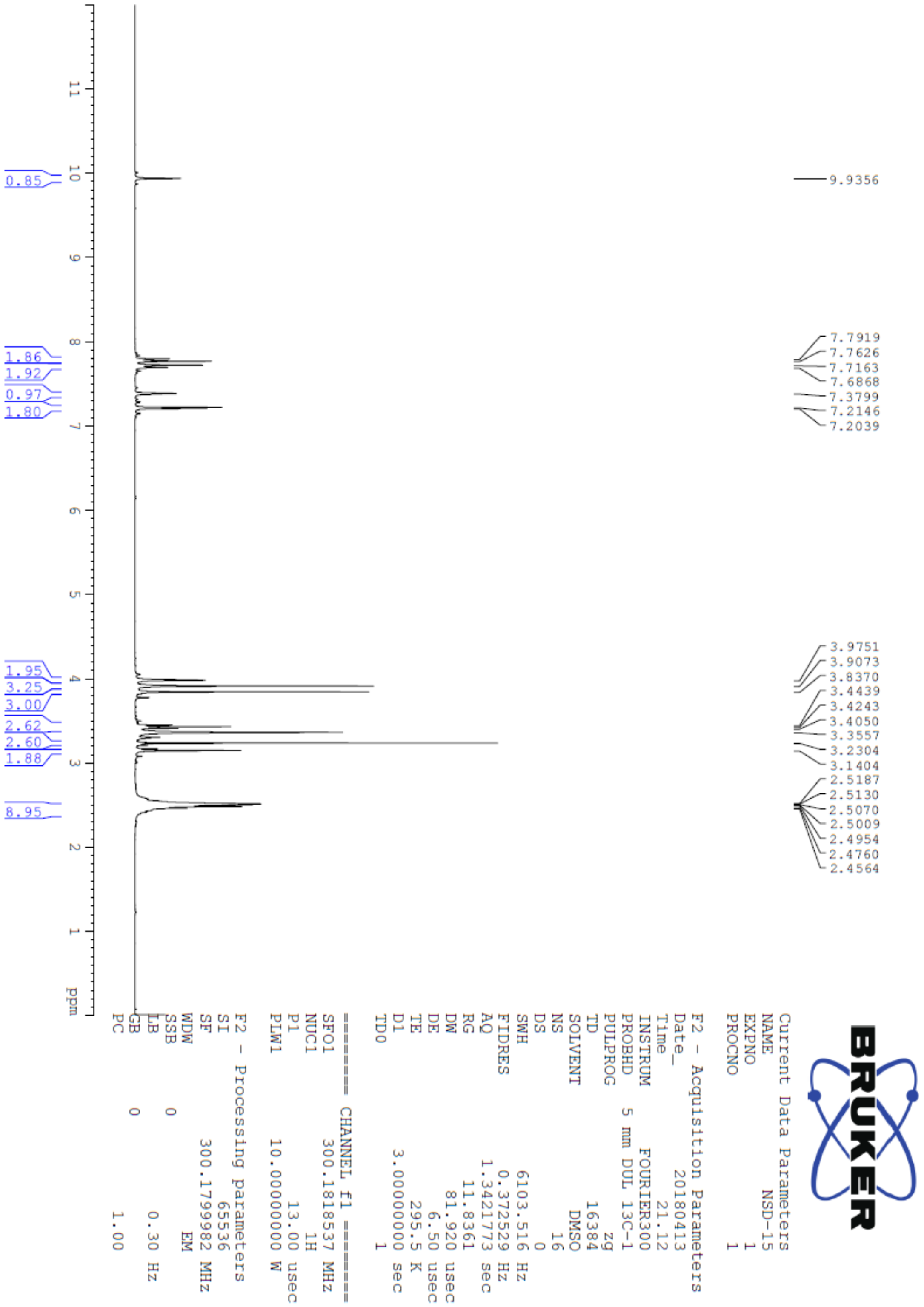

Figure S110. Compound **D15** ^{1}H-NMR spectrum.

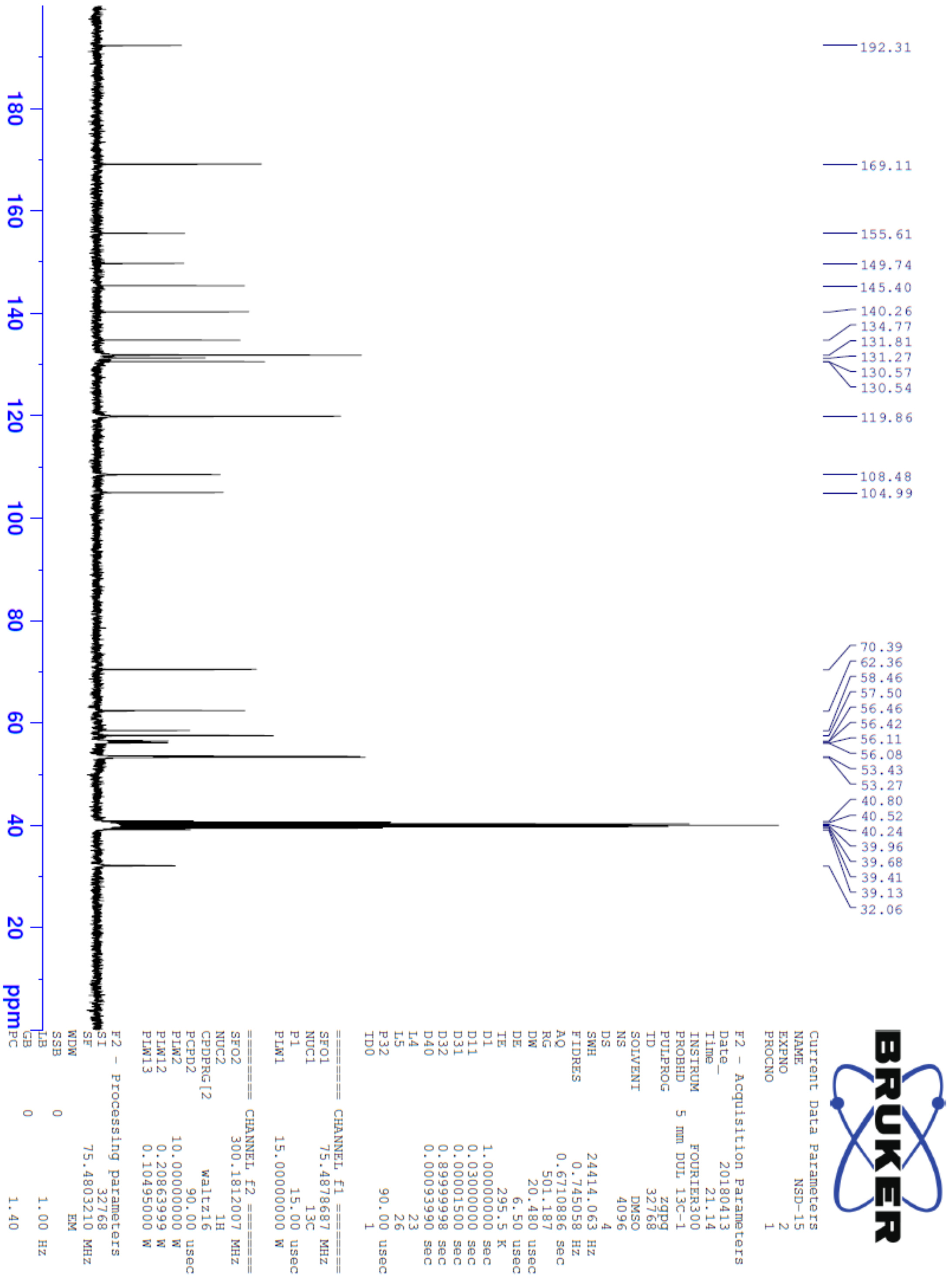

Figure S111. Compound **D15** ^{13}C-NMR spectrum.

Data File: C:\LabSolutions\Data\Analiz\Serkan\NSD-15_54.lcd

Elmt	Val.	Min	Max	Elmt	Val.	Min	Max	Elmt	Val.	Min	Max	Elmt	Val.	Min	Max	Use Adduct
H	1	5	40	O	2	3	5	S	2	0	0	Ru	2	0	0	H
C	4	0	35	F	1	0	0	Cl	1	0	0	I	3	0	0	
N	3	3	6	P	3	0	0	Br	1	0	0					

Error Margin (ppm):	5	DBE Range:	10.0 - 17.0	Electron Ions:	both
HC Ratio:	unlimited	Apply N Rule:	yes	Use MSn Info:	yes
Max Isotopes:	3	Isotope RI (%):	1.00	Isotope Res:	9000
MSn Iso RI (%):	10.00	MSn Logic Mode:	AND	Max Results:	500

Event#: 1 MS(E+) Ret. Time : 2.213 -> 2.480 Scan# : 333 -> 373

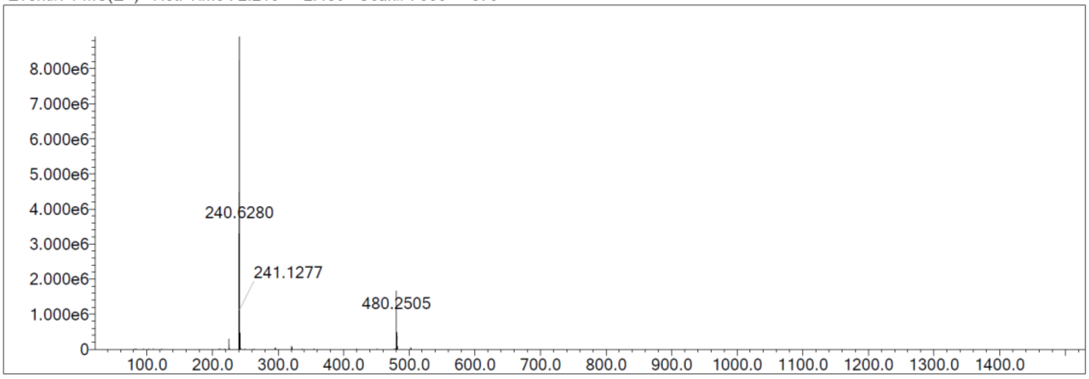

Measured region for 480.2505 m/z

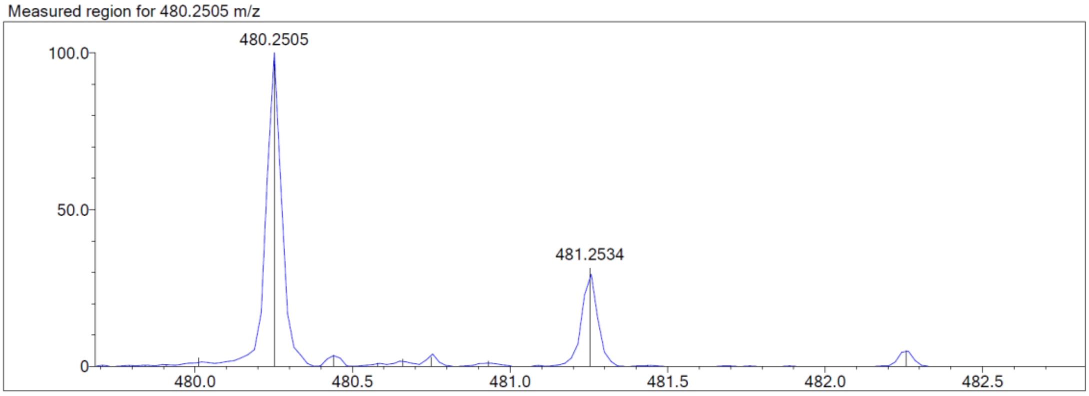

C27 H33 N3 O5 [M+H]+ : Predicted region for 480.2493 m/z

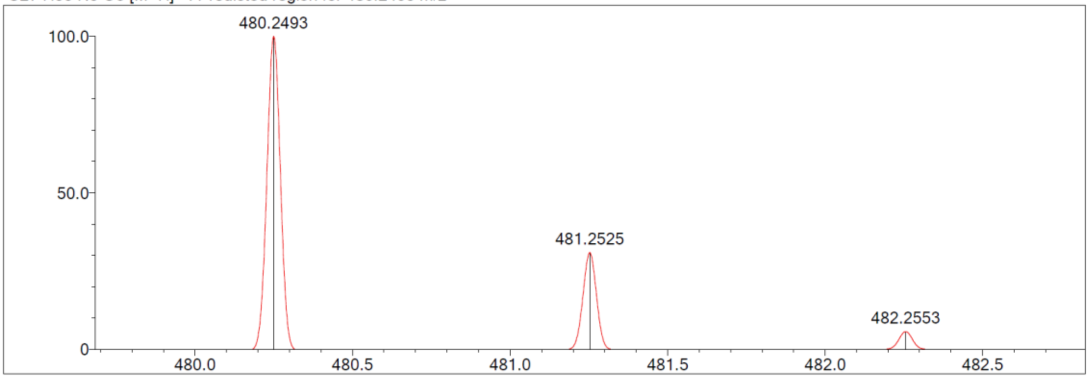

Rank	Score	Formula (M)	Ion	Meas. m/z	Pred. m/z	Df. (mDa)	Df. (ppm)	Iso	DBE
1	91.82	C27 H33 N3 O5	[M+H]+	480.2505	480.2493	1.2	2.50	95.39	13.0

Figure S112. Compound **D15** HRMS report.

*2-(4-(2-Hydroxyethyl)piperazine-1-yl)-N-(4-((5-methoxy-1-oxo-2,3-dihydro-1H-inden-2-ylidene)methyl)phenyl) acetamide (**D16**)*

Light brown powder. M.P.: 138.6 °C. Yield: 82%.

IR (ATR) ν_{max} (cm^{-1}): 3340 (N-H), 1693 (indanone C=O), 1635 (amide C=O), 1541-1498 (C=C), 1211 (C-N), 1064 (C-O), 842 (1,4-disubstituted benzene).

^{1}H-NMR (300 MHz, DMSO-*d$_6$*) δ (ppm): 2.40 (2H, t, *J*=6.33 Hz, CH_2), 2.52 (8H, bs, piperazine CH_2), 3.14 (2H, s, CH_2), 3.42 (2H, t, *J*=5.82 Hz, CH_2), 3.50 (2H, s, CH_2), 3.89 (3H, s, OCH_3), 4.05 (2H, s, CH_2), 4.39 (1H, s, OH), 7.03 (1H, dd, J_1=8.49 Hz, J_2=2.25 Hz, methoxy-1-oxo-indenylidene CH), 7.18 (1H, d, *J*=2.04 Hz, methoxy-1-oxo-indenylidene CH), 7.40 (1H, s, C=CH), 7.70-7.73 (3H, m, disubstituted benzene CH, methoxy-1-oxo-indenylidene CH), 7.79 (2H, d, *J*=8.76 Hz, disubstituted benzene CH), 9.95 (1H, s, NH).

^{13}C-NMR (75 MHz, DMSO-*d$_6$*) δ (ppm): 32.5, 53.3, 53.5, 56.3, 59.0, 60.7, 62.4, 110.6, 115.8, 119.9, 125.8, 130.5, 131.1, 131.7, 131.9, 134.5, 140.4, 153.3, 165.3, 169.1, 192.0.

HRMS (ESI) (m/z) [M+H]$^+$: $C_{25}H_{29}N_3O_4$ calculated: 436.2231, found: 436.2245.

DOPNALAB

	Item	Value
	Acquired Date&Time	22.08.2019 11:54:41
	Acquired by	System Administrator
	Filename	C:\Users\dopnalab\Desktop\NURPELİN\DOKTORA TEZ\D161.ispd
	Spectrum name	D161
	Sample name	D16
	Sample ID	
	Option	
	Comment	
	No. of Scans	50
	Resolution	4 [cm-1]
	Apodization	Happ-Genzel

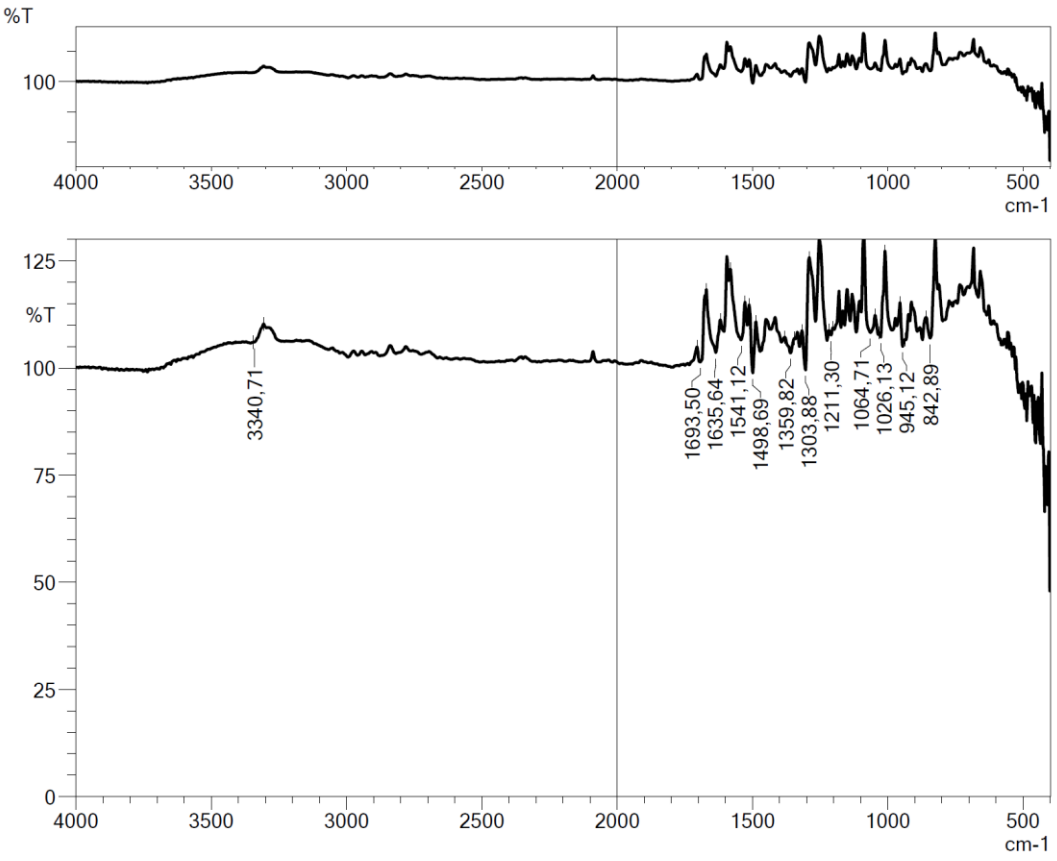

Figure S113. Compound **D16** IR report.

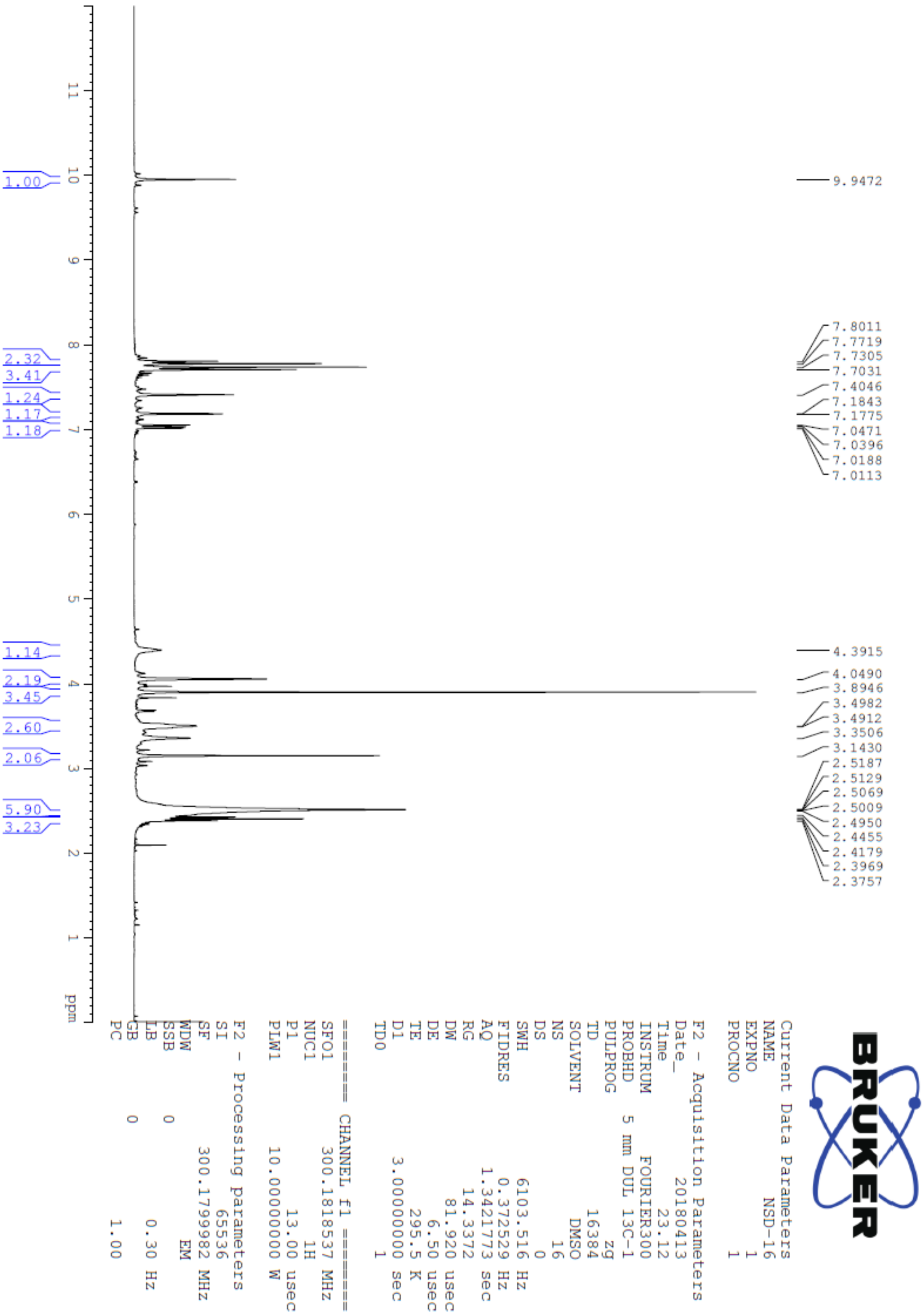

Figure S114. Compound **D16** ^{1}H-NMR spectrum.

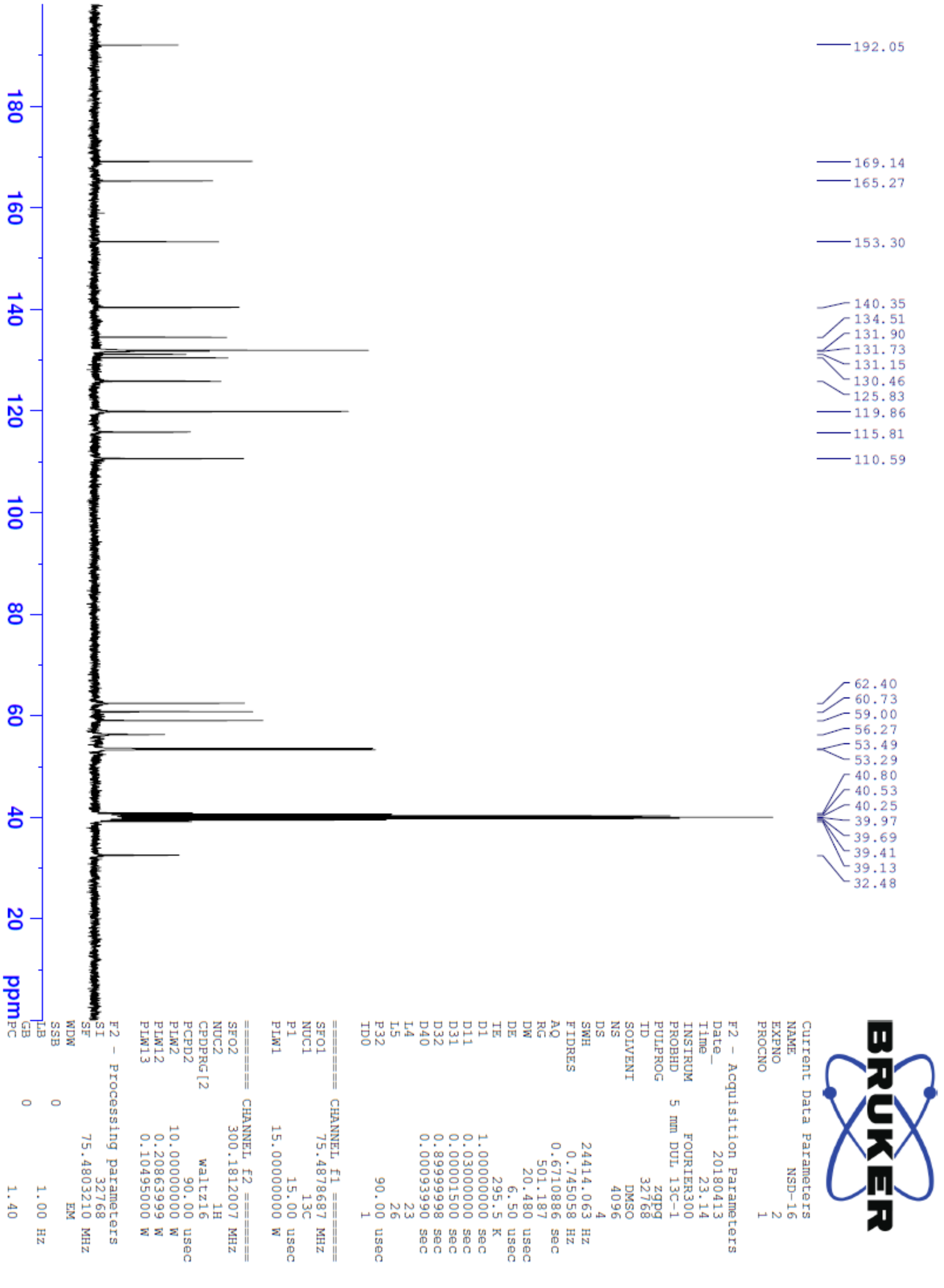

Figure S115. Compound **D16** ^{13}C-NMR spectrum.

Formula Predictor Report - NSD-16_55.lcd Page 1 of 1

Data File: C:\LabSolutions\Data\Analiz\Serkan\NSD-16_55.lcd

Elmt	Val.	Min	Max	Elmt	Val.	Min	Max	Elmt	Val.	Min	Max	Elmt	Val.	Min	Max	Use Adduct
H	1	5	40	O	2	3	5	S	2	0	0	Ru	2	0	0	H
C	4	0	35	F	1	0	0	Cl	1	0	0	I	3	0	0	
N	3	3	6	P	3	0	0	Br	1	0	0					

Error Margin (ppm):	5	DBE Range:	10.0 - 17.0	Electron Ions:	both
HC Ratio:	unlimited	Apply N Rule:	yes	Use MSn Info:	yes
Max Isotopes:	3	Isotope RI (%):	1.00	Isotope Res:	9000
MSn Iso RI (%):	10.00	MSn Logic Mode:	AND	Max Results:	500

Event#: 1 MS(E+) Ret. Time : 2.200 -> 2.387 Scan# : 331 -> 359

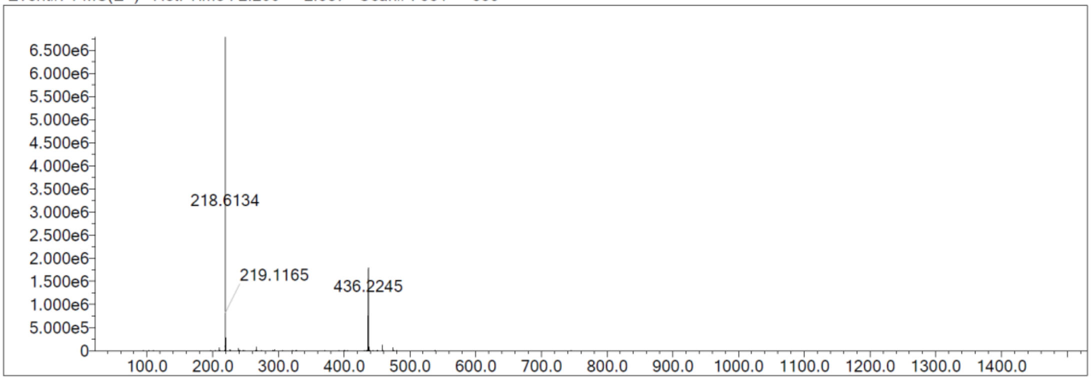

Measured region for 436.2245 m/z

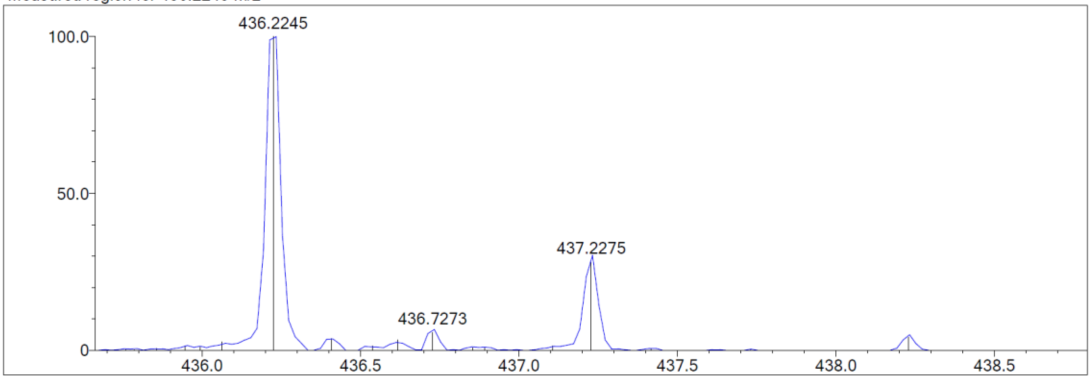

C25 H29 N3 O4 [M+H]+ : Predicted region for 436.2231 m/z

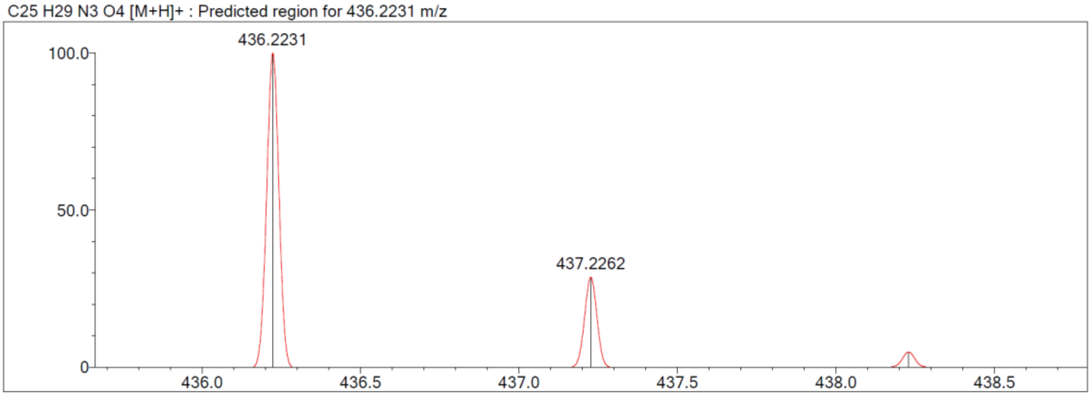

Rank	Score	Formula (M)	Ion	Meas. m/z	Pred. m/z	Df. (mDa)	Df. (ppm)	Iso	DBE
1	94.47	C25 H29 N3 O4	[M+H]+	436.2245	436.2231	1.4	3.21	100.00	13.0

Figure S116. Compound **D16** HRMS report.

*2-(4-(2-Hydroxyethyl)piperazine-1-yl)-N-(4-((6-methoxy-1-oxo-2,3-dihydro-1H-inden-2-ylidene)methyl)phenyl) acetamide (**D17**)*

Light brown powder. M.P.: 152.9 °C. Yield: 89%.

IR (ATR) ν_{max} (cm^{-1}): 3354 (N-H), 1685 (indanone C=O), 1637 (amide C=O), 1548-1490 (C=C), 1209 (C-N), 1058 (C-O), 831 (1,4-disubstituted benzene).

^{1}H-NMR (300 MHz, DMSO-*d_6*) δ (ppm): 2.39 (2H, t, *J*=6.30 Hz, CH_2), 2.52 (8H, bs, piperazine CH_2), 3.14 (2H, s, CH_2), 3.49 (2H, t, *J*=6.20 Hz, CH_2), 3.82 (3H, s, OCH_3), 3.99 (2H, s, CH_2), 4.39 (1H, s, OH), 7.22 (1H, d, *J*=2.34 Hz, methoxy-1-oxo-indenylidene CH), 7.27 (1H, dd, J_1=8.31 Hz, J_2=2.43 Hz, methoxy-1-oxo-indenylidene CH), 7.46 (1H, s, C=CH), 7.56 (1H, d, *J*=8.37 Hz, methoxy-1-oxo-indenylidene CH), 7.72 (2H, d, *J*=8.92 Hz, disubstituted benzene CH), 7.78 (2H, d, *J*=8.83 Hz, disubstituted benzene CH), 9.95 (1H, s, NH).

^{13}C-NMR (75 MHz, DMSO-*d_6*) δ (ppm): 31.7, 53.3, 53.5, 56.0, 59.0, 60.7, 62.4, 106.0, 119.9, 123.7, 127.9, 130.3, 132.1, 133.0, 134.7, 139.1, 140.6, 142.9, 159.6, 169.2, 193.6.

HRMS (ESI) (m/z) $[M+H]^+$: $C_{25}H_{29}N_3O_4$ calculated: 436.2231, found: 436.2251.

DOPNALAB

	Item	Value
	Acquired Date&Time	22.08.2019 11:57:22
	Acquired by	System Administrator
	Filename	C:\Users\dopnalab\Desktop\NURPELİN\DOKTORA TEZ\D171.ispd
	Spectrum name	D171
	Sample name	D17
	Sample ID	
	Option	
	Comment	
	No. of Scans	50
	Resolution	4 [cm-1]
	Apodization	Happ-Genzel

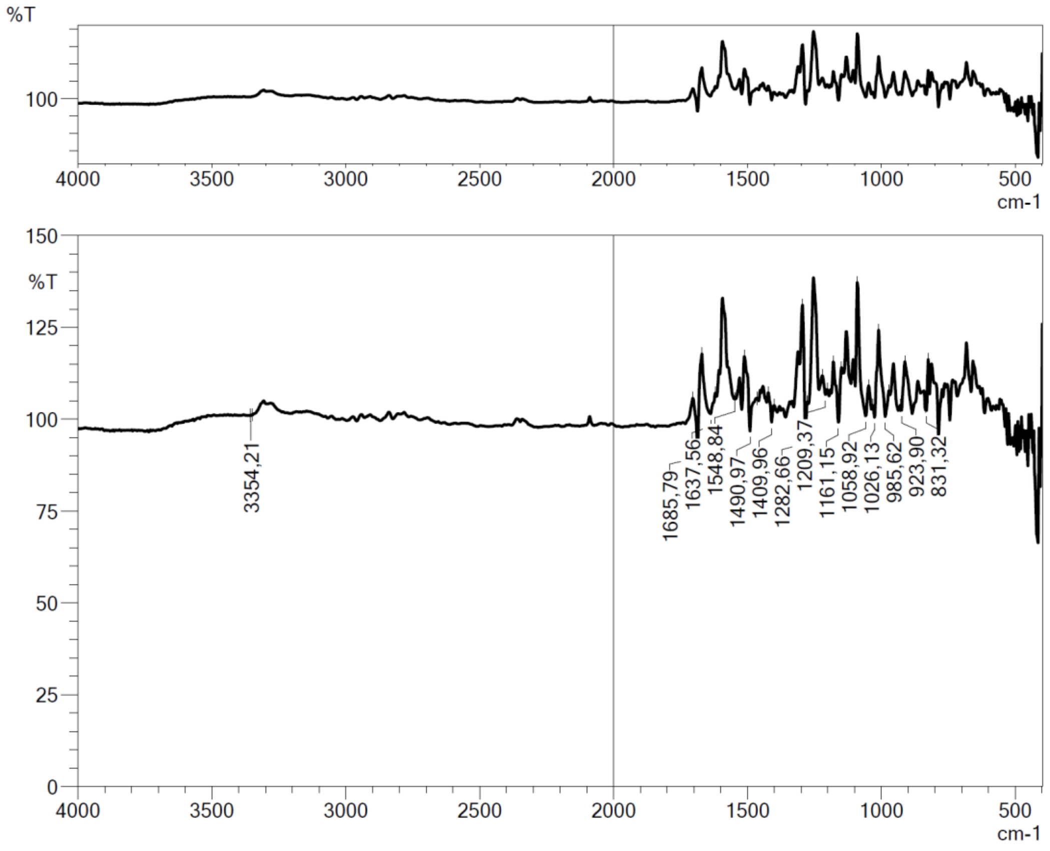

Figure S117. Compound **D17** IR report.

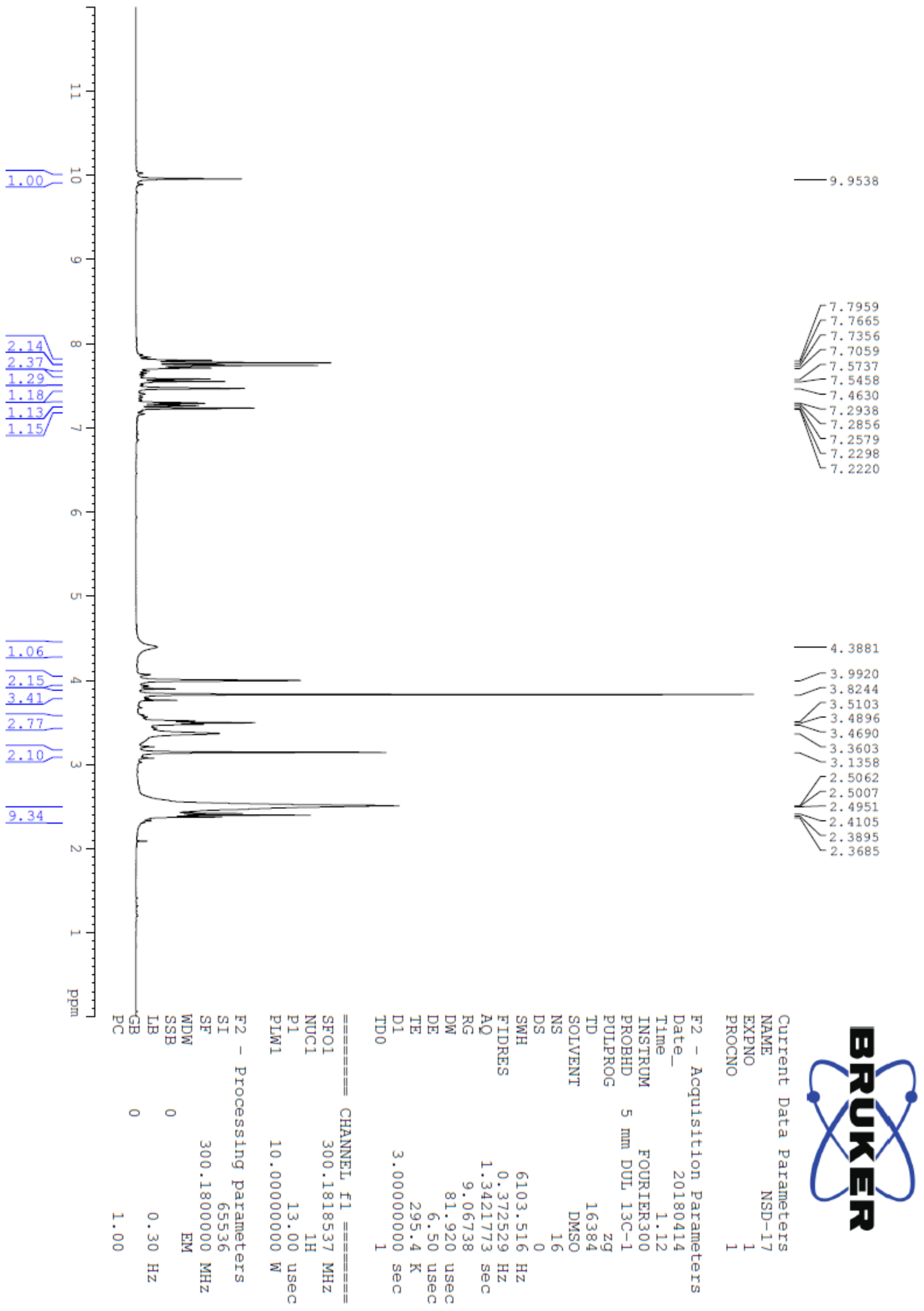

Figure S118. Compound **D17** ^{1}H-NMR spectrum.

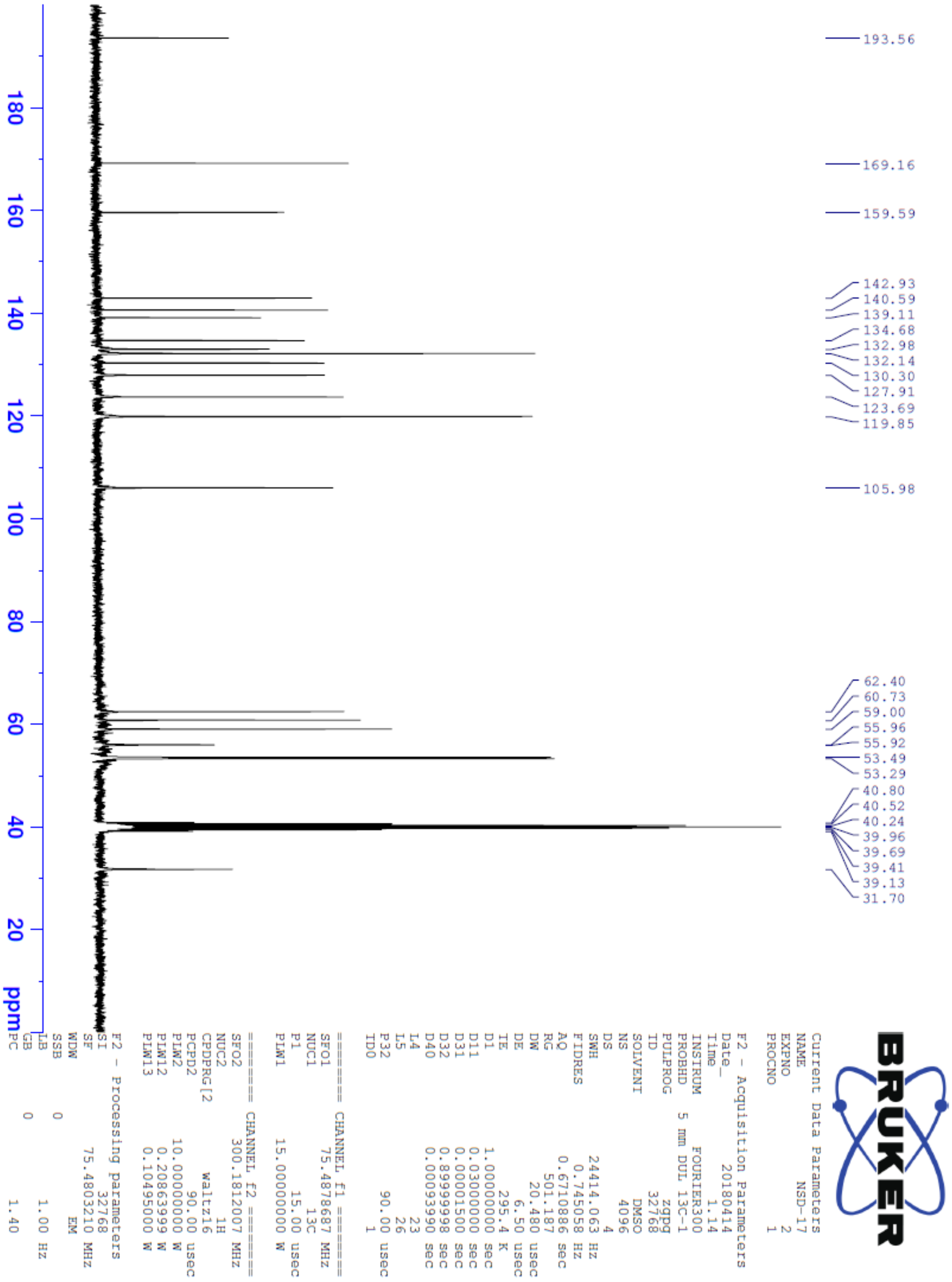

Figure S119. Compound **D17** ^{13}C-NMR spectrum.

Data File: C:\LabSolutions\Data\Analiz\Serkan\NSD-17_56.lcd

Elmt	Val.	Min	Max	Elmt	Val.	Min	Max	Elmt	Val.	Min	Max	Elmt	Val.	Min	Max	Use Adduct
H	1	5	40	O	2	3	5	S	2	0	0	Ru	2	0	0	H
C	4	0	35	F	1	0	0	Cl	1	0	0	I	3	0	0	
N	3	3	6	P	3	0	0	Br	1	0	0					

Error Margin (ppm):	5	DBE Range:	10.0 - 17.0	Electron Ions:	both
HC Ratio:	unlimited	Apply N Rule:	yes	Use MSn Info:	yes
Max Isotopes:	3	Isotope RI (%):	1.00	Isotope Res:	9000
MSn Iso RI (%):	10.00	MSn Logic Mode:	AND	Max Results:	500

Event#: 1 MS(E+) Ret. Time : 2.200 -> 2.427 Scan# : 331 -> 365

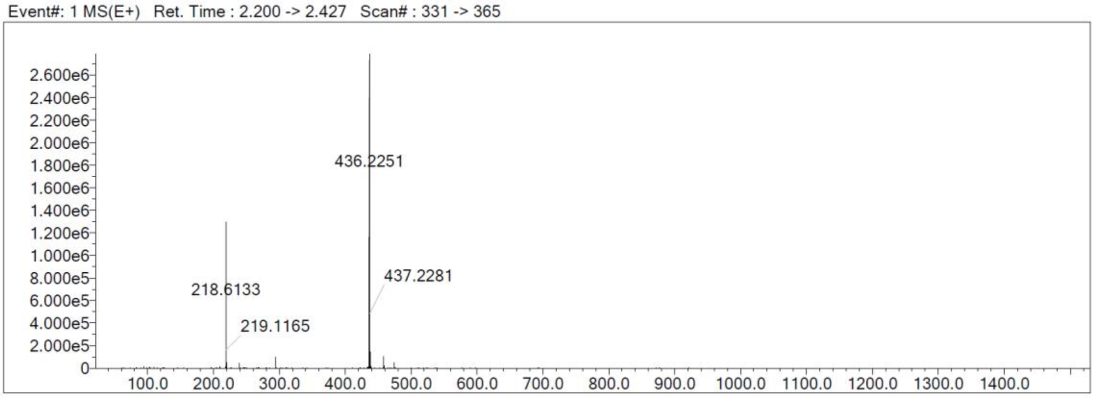

Measured region for 436.2251 m/z

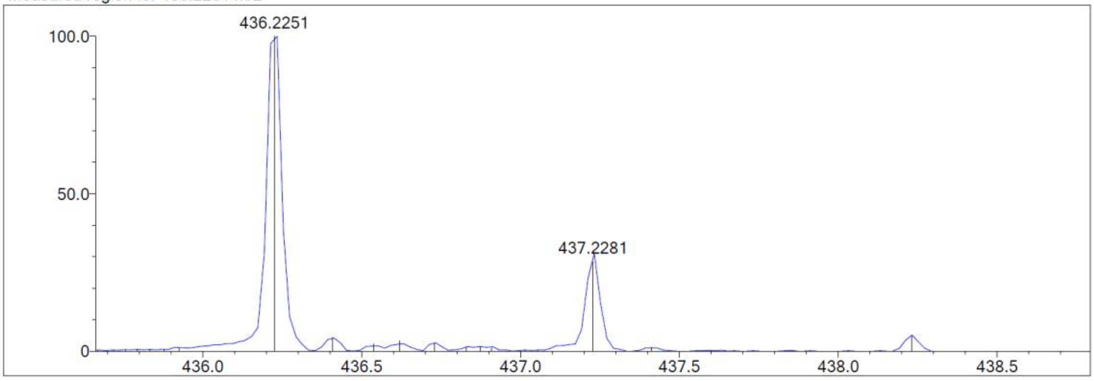

C25 H29 N3 O4 [M+H]+ : Predicted region for 436.2231 m/z

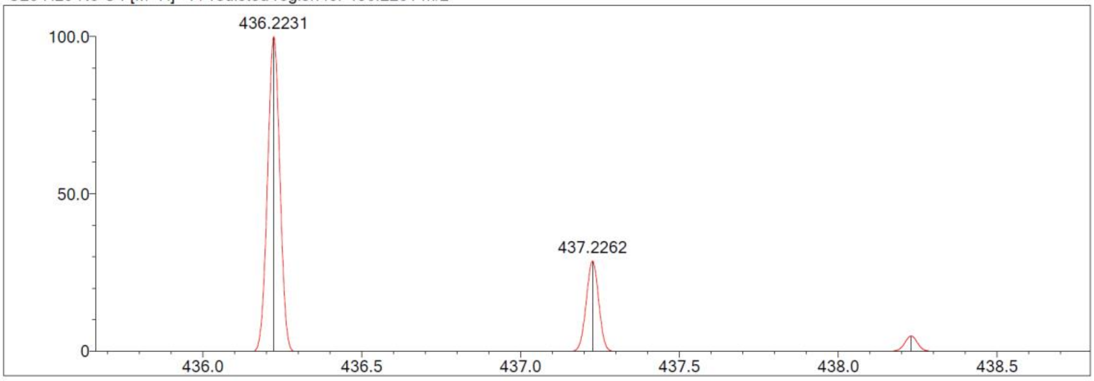

Rank	Score	Formula (M)	Ion	Meas. m/z	Pred. m/z	Df. (mDa)	Df. (ppm)	Iso	DBE
1	91.05	C25 H29 N3 O4	[M+H]+	436.2251	436.2231	2.0	4.58	100.00	13.0

Figure S120. Compound **D17** HRMS report.

*2-(4-(2-Hydroxyethyl)piperazine-1-yl)-N-(4-((5,6-dimethoxy-1-oxo-2,3-dihydro-1H-inden-2-ylidene)methyl) phenyl)acetamide (**D18**)*

Brown powder. M.P.: 165.7 °C. Yield: 79%.

IR (ATR) ν_{max} (cm^{-1}): 3350 (N-H), 1734 (indanone C=O), 1647 (amide C=O), 1533-1471 (C=C), 1219 (C-N), 1058 (C-O), 844 (1,4-disubstituted benzene).

^{1}H-NMR (300 MHz, DMSO-d_6) δ (ppm): 2.40 (2H, t, *J*=6.32 Hz, CH_2), 2.52 (8H, bs, piperazine CH_2), 3.14 (2H, s, CH_2), 3.49 (2H, bs, CH_2), 3.84 (3H, s, OCH_3), 3.91 (3H, s, OCH_3), 3.97 (2H, s, CH_2), 4.40 (1H, s, OH), 7.20 (1H, s, methoxy-1-oxo-indenylidene CH), 7.21 (1H, s, methoxy-1-oxo-indenylidene CH), 7.38 (1H, s, C=CH), 7.70 (2H, d, *J*=8.86 Hz, disubstituted benzene CH), 7.78 (2H, d, *J*=8.74 Hz, disubstituted benzene CH), 9.94 (1H, s, NH).

^{13}C-NMR (75 MHz, DMSO-d_6) δ (ppm): 32.1, 53.3, 53.5, 56.0, 56.4, 59.0, 60.7, 62.4, 105.0, 119.9, 130.5, 130.6, 131.3, 131.8, 134.8, 140.3, 145.4, 149.7, 155.6, 169.1, 192.3.

HRMS (ESI) (m/z) $[M+H]^+$: $C_{26}H_{31}N_3O_5$ calculated: 466.2336, found: 466.2334.

DOPNALAB

	Item	Value
	Acquired Date&Time	22.08.2019 12:00:31
	Acquired by	System Administrator
	Filename	C:\Users\dopnalab\Desktop\NURPELİN\DOKTORA TEZ\D181.ispd
	Spectrum name	D181
	Sample name	D18
	Sample ID	
	Option	
	Comment	
	No. of Scans	50
	Resolution	4 [cm-1]
	Apodization	Happ-Genzel

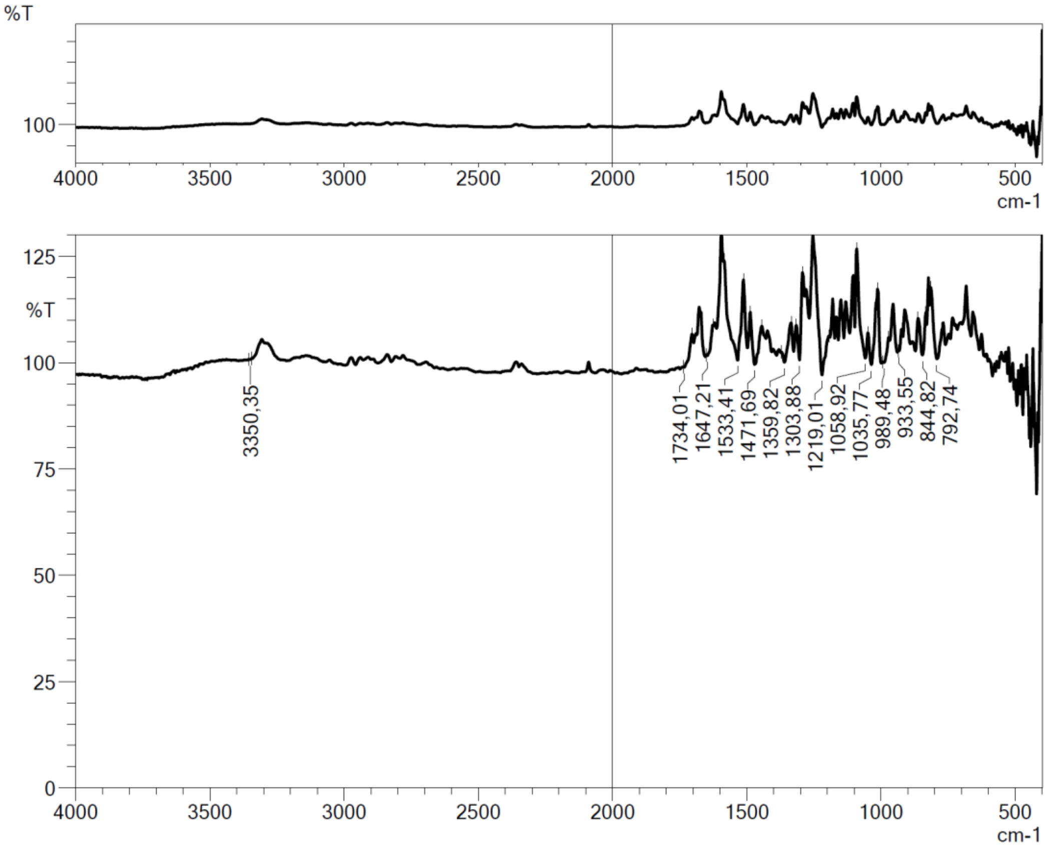

Figure S121. Compound **D18** IR report.

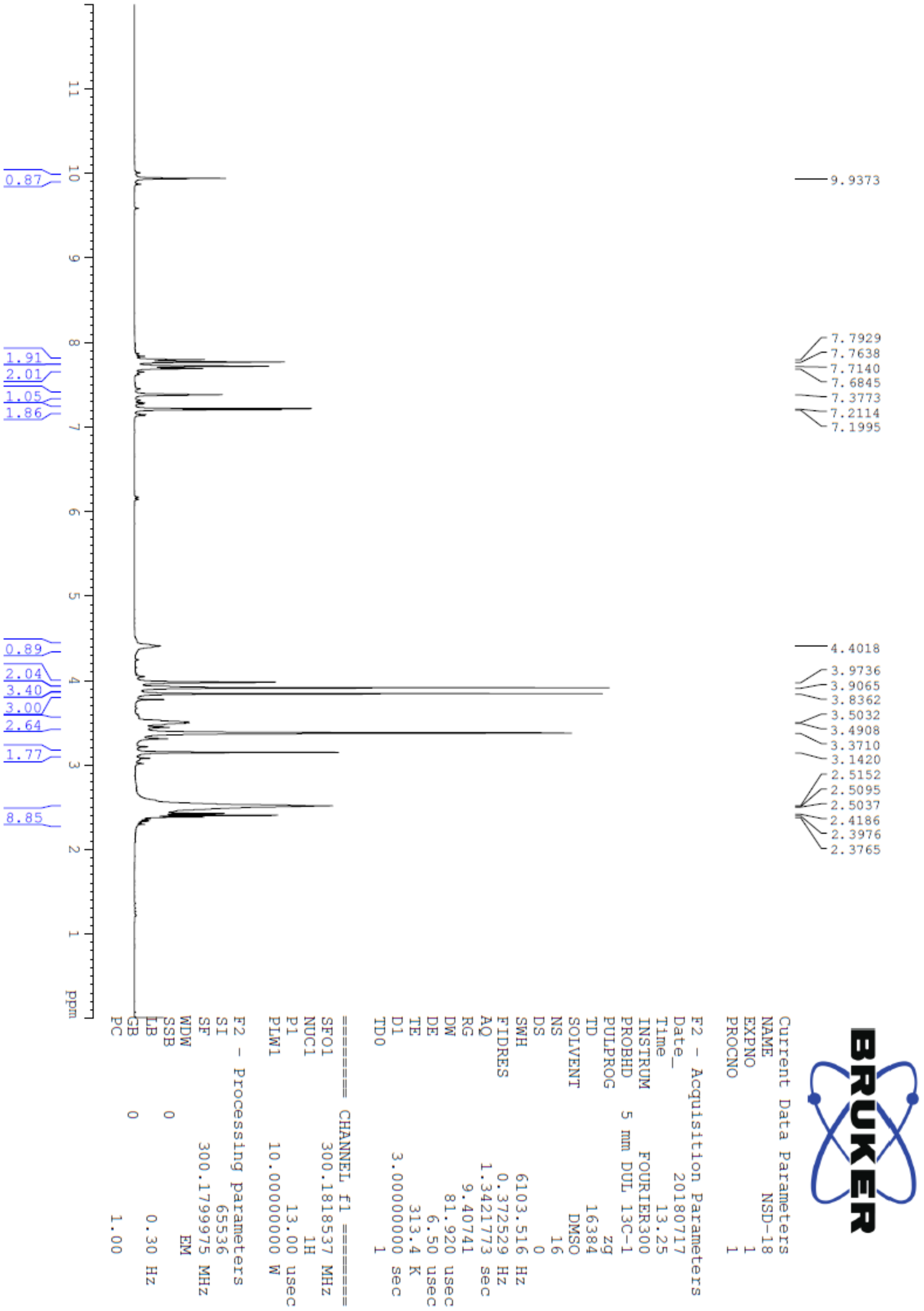

Figure S122. Compound **D18** ^{1}H-NMR spectrum.

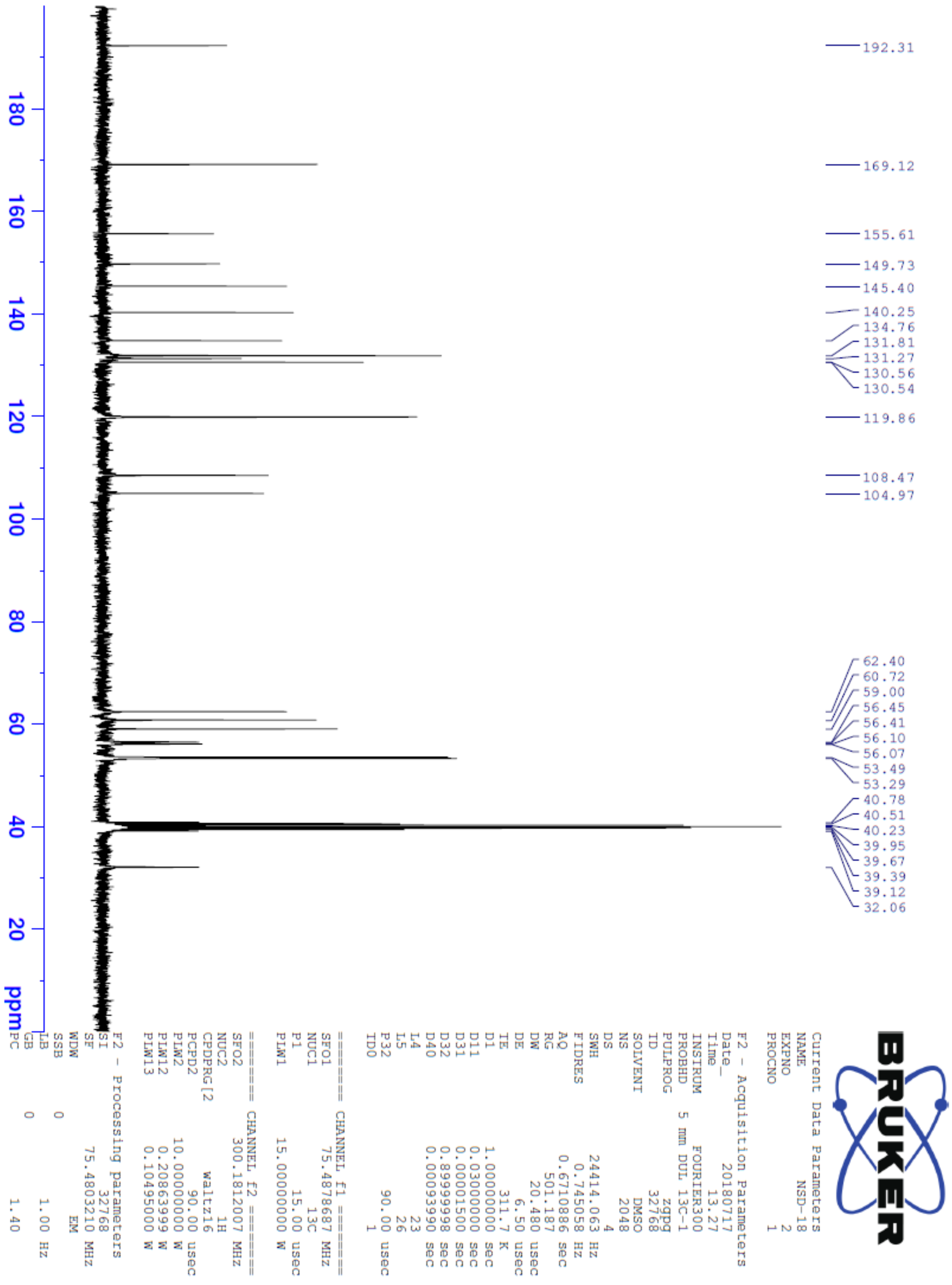

Figure S123. Compound **D18** ^{13}C-NMR spectrum.

Data File: C:\LabSolutions\Data\Analiz\bns\NSD-18_3.lcd

Elmt	Val.	Min	Max	Elmt	Val.	Min	Max	Elmt	Val.	Min	Max	Elmt	Val.	Min	Max	Use Adduct
H	1	5	40	O	2	3	5	S	2	0	0	Ru	2	0	0	H
C	4	0	35	F	1	0	0	Cl	1	0	0	I	3	0	0	
N	3	3	6	P	3	0	0	Br	1	0	0					

Error Margin (ppm):	5	DBE Range:	10.0 - 17.0	Electron Ions:	both
HC Ratio:	unlimited	Apply N Rule:	yes	Use MSn Info:	yes
Max Isotopes:	3	Isotope RI (%):	1.00	Isotope Res:	9000
MSn Iso RI (%):	10.00	MSn Logic Mode:	AND	Max Results:	500

Event#: 1 MS(E+) Ret. Time : 4.920 -> 5.027 Scan# : 739 -> 755

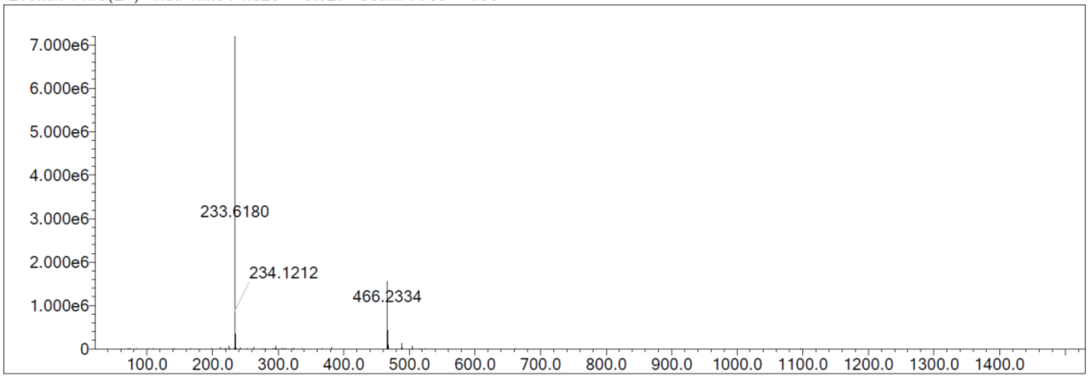

Measured region for 466.2334 m/z

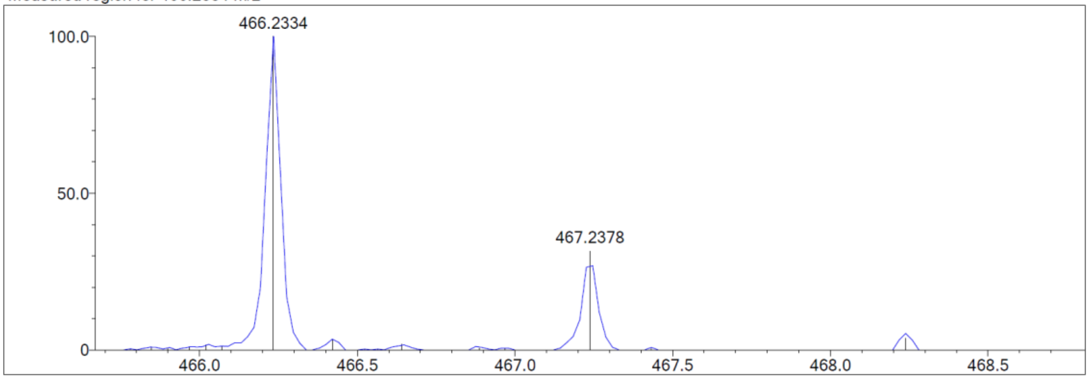

C26 H31 N3 O5 [M+H]+ : Predicted region for 466.2336 m/z

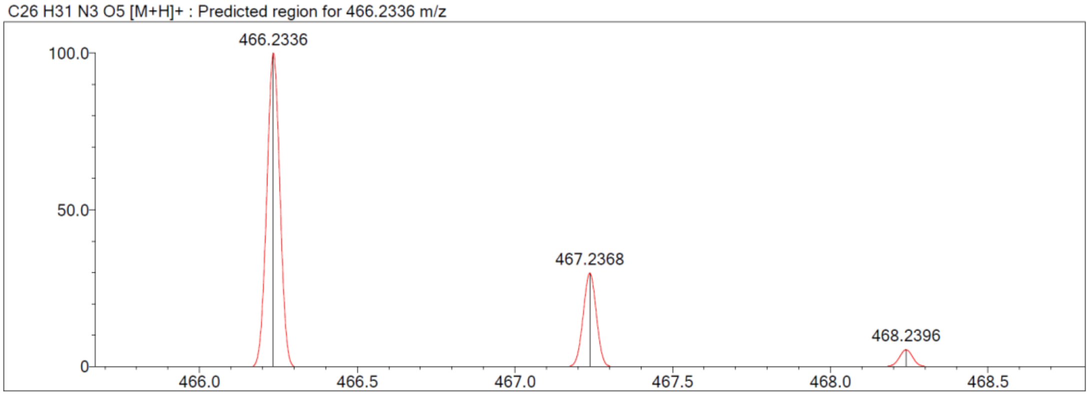

Rank	Score	Formula (M)	Ion	Meas. m/z	Pred. m/z	Df. (mDa)	Df. (ppm)	Iso	DBE
1	100.00	C26 H31 N3 O5	[M+H]+	466.2334	466.2336	-0.2	-0.43	100.00	13.0

Figure S124. Compound **D18** HRMS report.

*2-(4-(3-Hydroxypropyl)piperazine-1-yl)-N-(4-((5-methoxy-1-oxo-2,3-dihydro-1H-inden-2-ylidene)methyl)phenyl) acetamide (**D19**)*

Brown powder. M.P.: 175.2 °C. Yield: 84%.

IR (ATR) ν_{max} (cm^{-1}): 3350 (N-H), 1685 (indanone C=O), 1633 (amide C=O), 1543-1483 (C=C), 1211 (C-N), 1068 (C-O), 837 (1,4-disubstituted benzene).

^{1}H-NMR (300 MHz, DMSO-d_6) δ (ppm): 1.56 (2H, p, *J*=6.71 Hz, CH_2), 2.34 (2H, t, *J*=7.19 Hz, CH_2), 2.54 (8H, bs, CH_2, piperazine CH_2), 3.14 (2H, m, CH, CH_2), 3.43 (2H, t, *J*=6.29 Hz, CH_2), 3.89 (3H, s, OCH_3), 4.05 (2H, s, CH_2), 4.47 (1H, s, OH), 7.02 (1H, dd, J_1=8.49 Hz, J_2=2.19 Hz, methoxy-1-oxo-indenylidene CH), 7.18 (1H, *J*=1.95 Hz, methoxy-1-oxo-indenylidene CH), 7.40 (1H, s, C=CH), 7.70-7.73 (3H, m, disubstituted benzene CH, methoxy-1-oxo-indenylidene CH), 7.79 (2H, d, *J*=8.70 Hz, disubstituted benzene CH), 9.95 (1H, s, NH).

^{13}C-NMR (75 MHz, DMSO-d_6) δ (ppm): 30.1, 32.5, 53.2, 53.3, 55.6, 56.3, 59.9, 62.4, 110.6, 115.8, 119.9, 125.8, 130.5, 131.1, 131.7, 131.9, 134.5, 140.4, 153.3, 165.3, 169.1, 192.0.

HRMS (ESI) (m/z) $[M+H]^+$: $C_{26}H_{31}N_3O_4$ calculated: 450.2387, found: 450.2399.

DOPNALAB

	Item	Value
	Acquired Date&Time	22.08.2019 12:03:15
	Acquired by	System Administrator
	Filename	C:\Users\dopnalab\Desktop\NURPELİN\DOKTORA TEZ\D191.ispd
	Spectrum name	D191
	Sample name	D19
	Sample ID	
	Option	
	Comment	
	No. of Scans	50
	Resolution	4 [cm-1]
	Apodization	Happ-Genzel

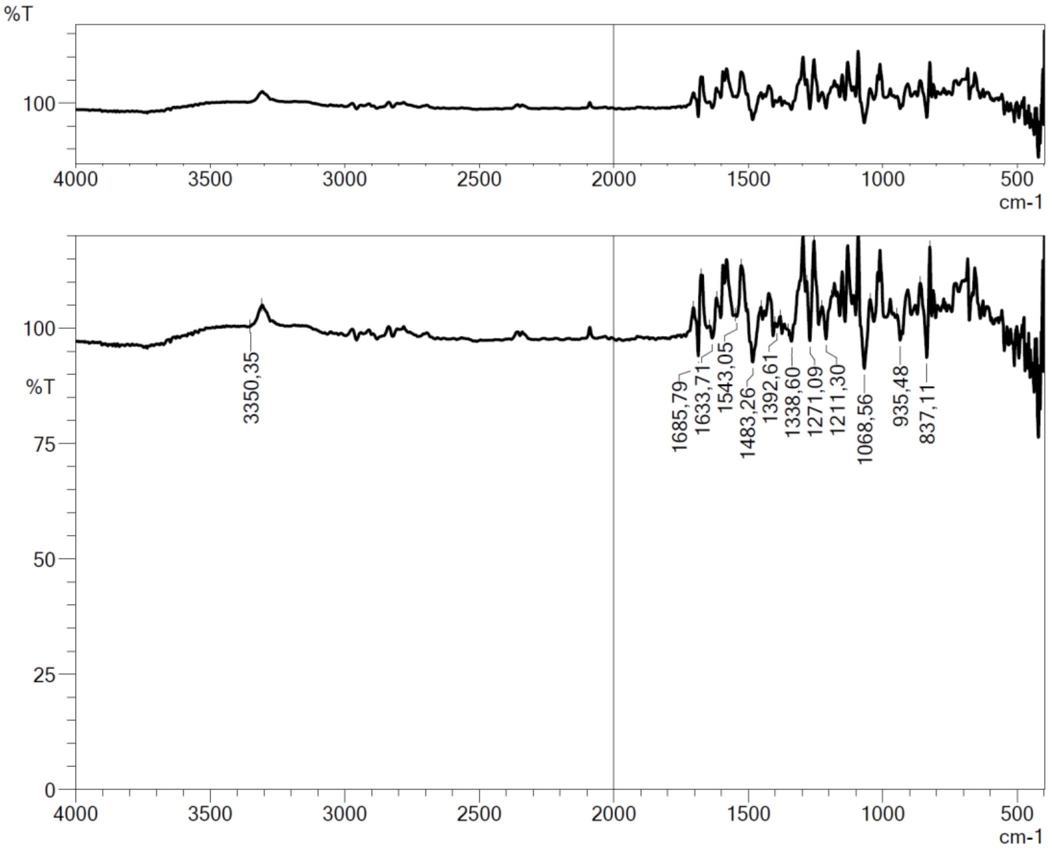

Figure S125. Compound **D19** IR report.

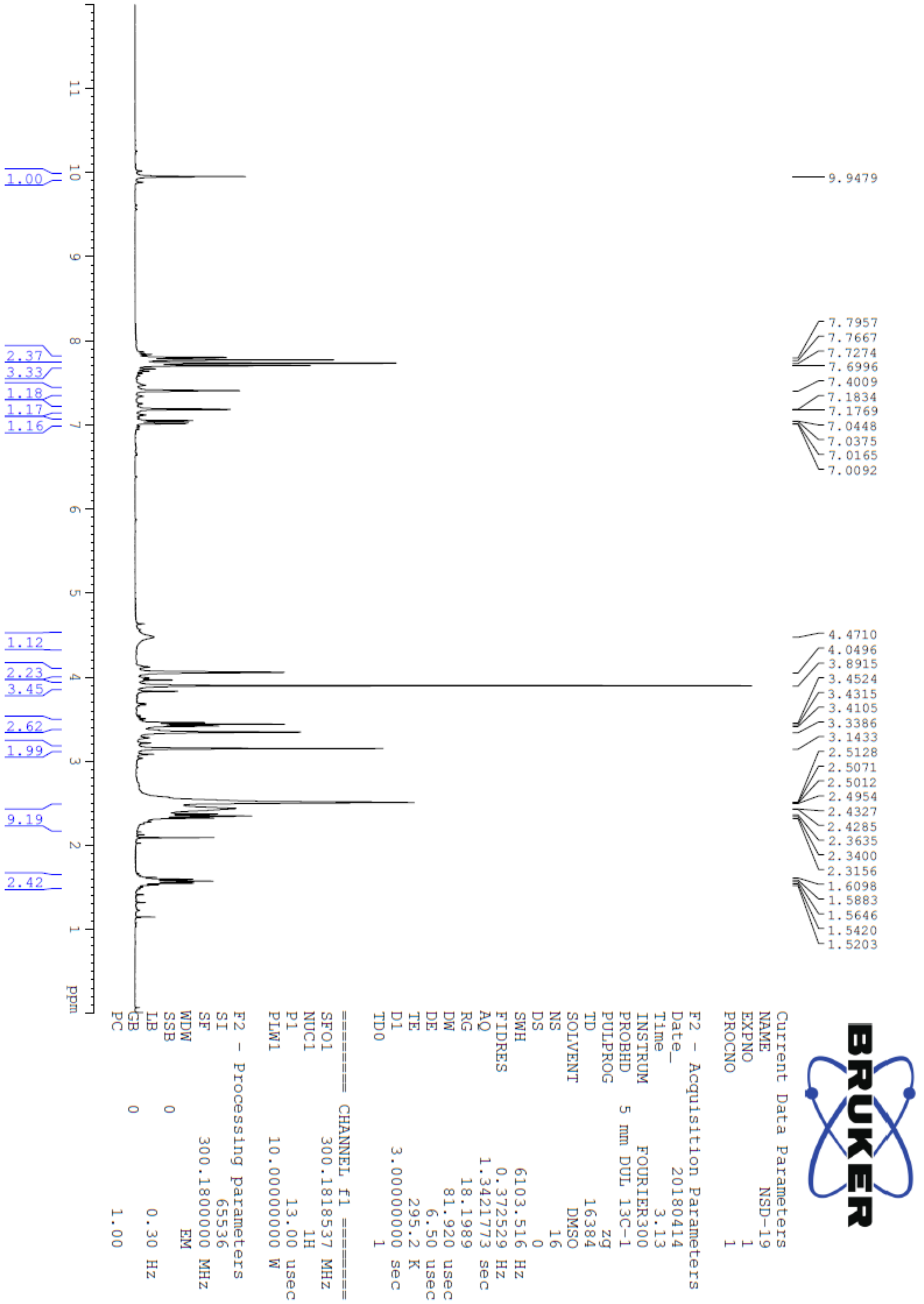

Figure S126. Compound **D19** ^{1}H-NMR spectrum.

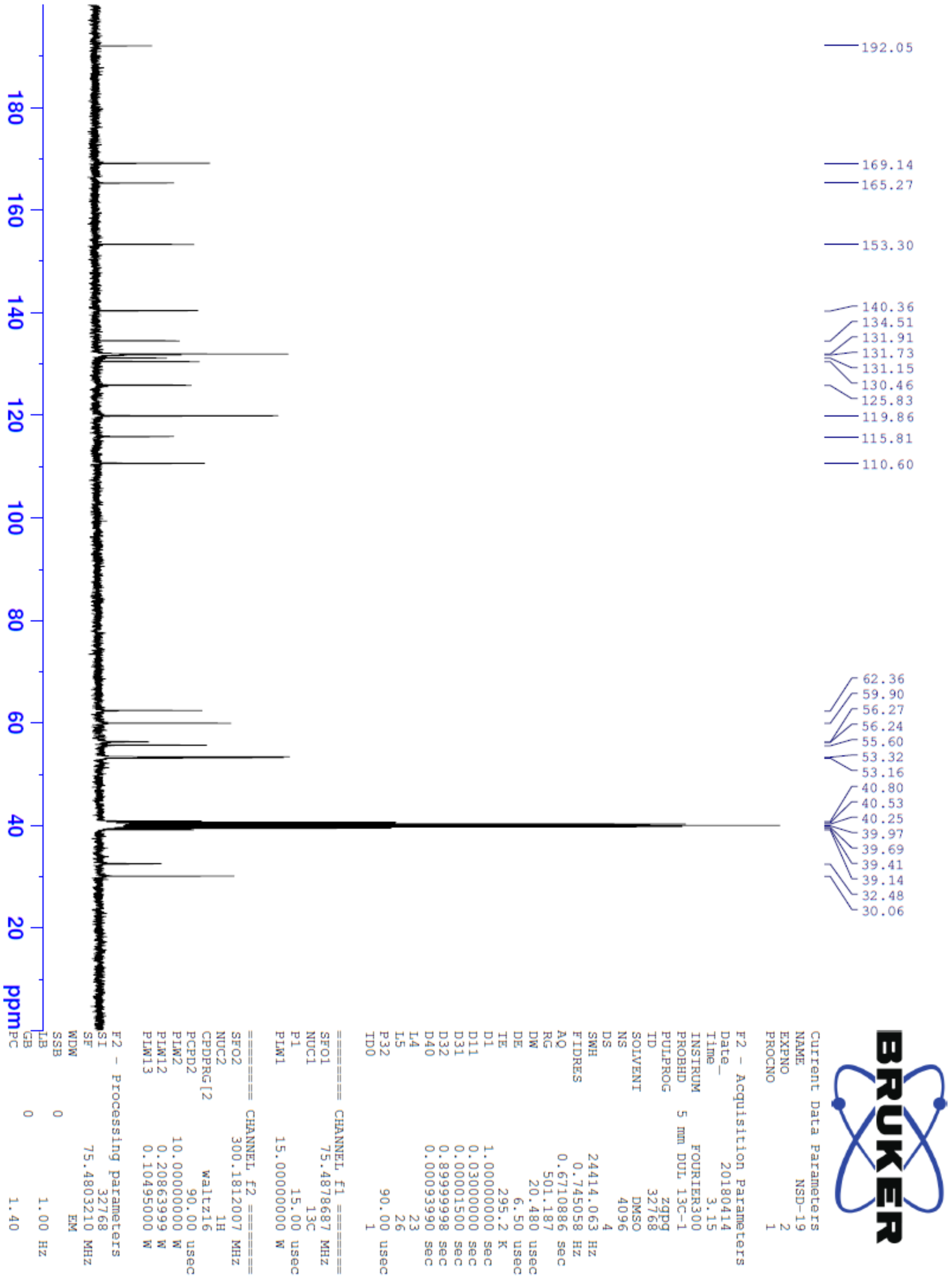

Figure S127. Compound **D19** ^{13}C-NMR spectrum.

Data File: C:\LabSolutions\Data\Analiz\Serkan\NSD-19_57.lcd

Elmt	Val.	Min	Max	Elmt	Val.	Min	Max	Elmt	Val.	Min	Max	Elmt	Val.	Min	Max	Use Adduct
H	1	5	40	O	2	3	5	S	2	0	0	Ru	2	0	0	H
C	4	0	35	F	1	0	0	Cl	1	0	0	I	3	0	0	
N	3	3	6	P	3	0	0	Br	1	0	0					

Error Margin (ppm): 5
HC Ratio: unlimited
Max Isotopes: 3
MSn Iso RI (%): 10.00

DBE Range: 10.0 - 17.0
Apply N Rule: yes
Isotope RI (%): 1.00
MSn Logic Mode: AND

Electron Ions: both
Use MSn Info: yes
Isotope Res: 9000
Max Results: 500

Event#: 1 MS(E+) Ret. Time : 2.200 -> 2.427 Scan# : 331 -> 365

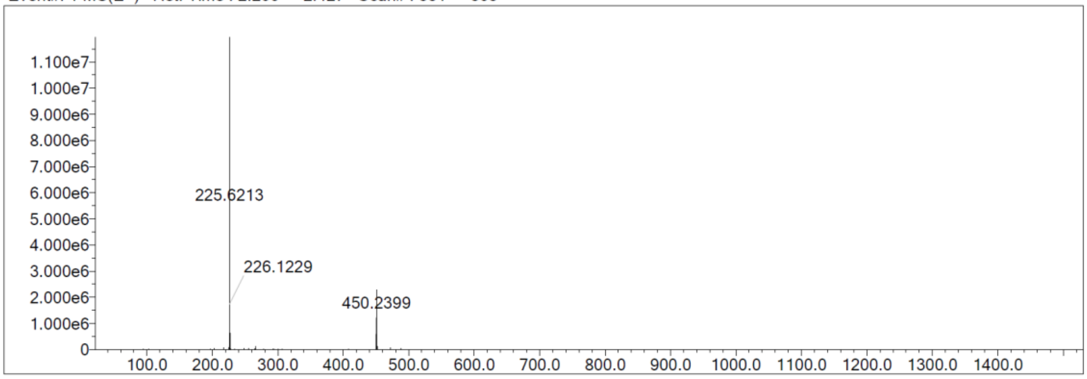

Measured region for 450.2399 m/z

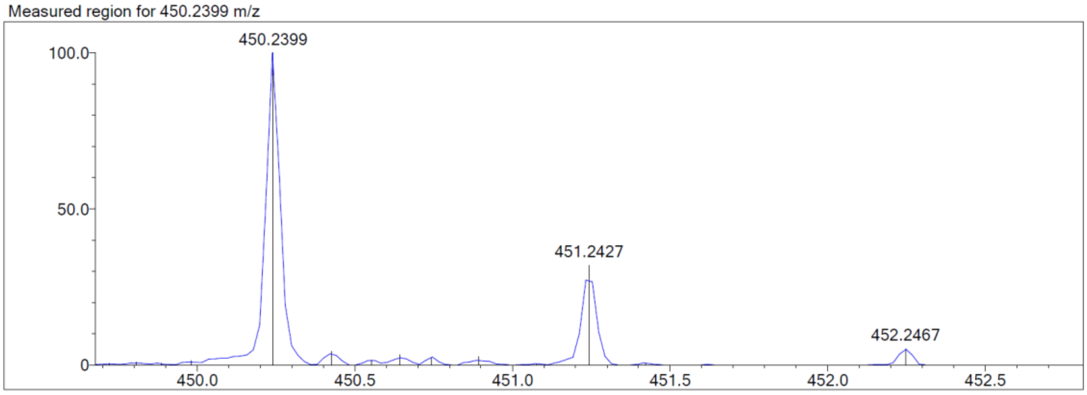

C26 H31 N3 O4 [M+H]+ : Predicted region for 450.2387 m/z

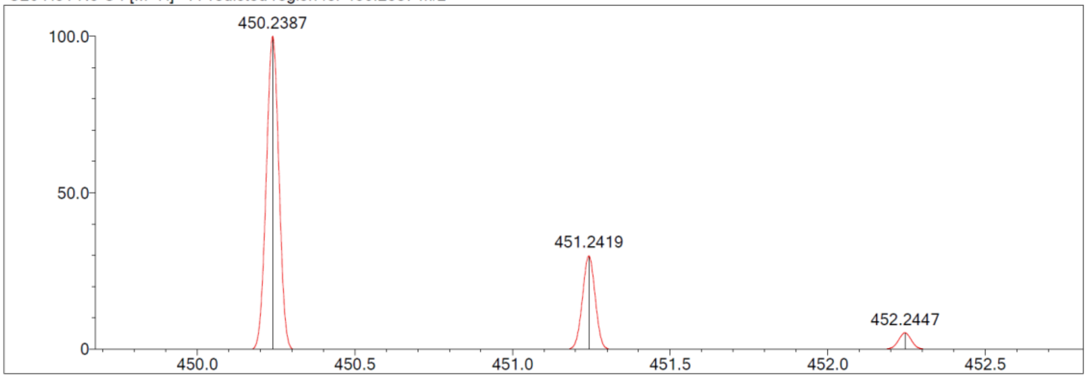

Rank	Score	Formula (M)	Ion	Meas. m/z	Pred. m/z	Df. (mDa)	Df. (ppm)	Iso	DBE
1	95.83	C26 H31 N3 O4	[M+H]+	450.2399	450.2387	1.2	2.67	100.00	13.0

Figure S128. Compound **D19** HRMS report.

*2-(4-(3-Hydroxypropyl)piperazine-1-yl)-N-(4-((6-methoxy-1-oxo-2,3-dihydro-1H-inden-2-ylidene)methyl)phenyl) acetamide (**D20**)*

Yellow powder. M.P.: 181.6 °C. Yield: 79%.

IR (ATR) ν_{max} (cm^{-1}): 3356 (N-H), 1703 (indanone C=O), 1683 (amide C=O), 1616-1436 (C=C), 1190 (C-N), 1095 (C-O), 835 (1,4-disubstituted benzene).

^{1}H-NMR (300 MHz, DMSO-*d_6*) δ (ppm): 1.56 (2H, p, *J*=6.72 Hz, CH_2), 2.34 (2H, t, *J*=7.20 Hz, CH_2), 2.43 (8H, bs, CH_2, piperazine CH_2), 3.15 (2H, s, CH_2), 3.43 (2H, t, *J*=6.29 Hz, CH_2), 3.83 (3H, s, OCH_3), 4.01 (2H, s, CH_2), 4.49 (1H, s, OH), 7.24 (1H, *J*=2.46 Hz, methoxy-1-oxo-indenylidene CH), 7.29 (1H, dd, J_1=8.31 Hz, J_2=2.55 Hz, methoxy-1-oxo-indenylidene CH), 7.48 (1H, s, C=CH), 7.57 (1H, d, *J*=8.35 Hz, methoxy-1-oxo-indenylidene CH), 7.73 (2H, d, *J*=9.00 Hz, disubstituted benzene CH), 7.79 (2H, d, *J*=8.95 Hz, disubstituted benzene CH), 9.97 (1H, s, NH).

^{13}C-NMR (75 MHz, DMSO-*d_6*) δ (ppm): 30.0, 31.7, 53.2, 53.3, 55.6, 56.0, 59.9, 62.4, 106.0, 119.9, 123.7, 127.9, 130.3, 132.2, 133.0, 134.7, 139.1, 140.6, 143.0, 159.6, 169.2, 193.6.

HRMS (ESI) (m/z) $[M+H]^+$: $C_{26}H_{31}N_3O_4$ calculated: 450.2387, found: 450.2395.

DOPNALAB

	Item	Value
	Acquired Date&Time	22.08.2019 12:06:20
	Acquired by	System Administrator
	Filename	C:\Users\dopnalab\Desktop\NURPELİN\DOKTORA TEZ\D201.ispd
	Spectrum name	D201
	Sample name	D20
	Sample ID	
	Option	
	Comment	
	No. of Scans	50
	Resolution	4 [cm-1]
	Apodization	Happ-Genzel

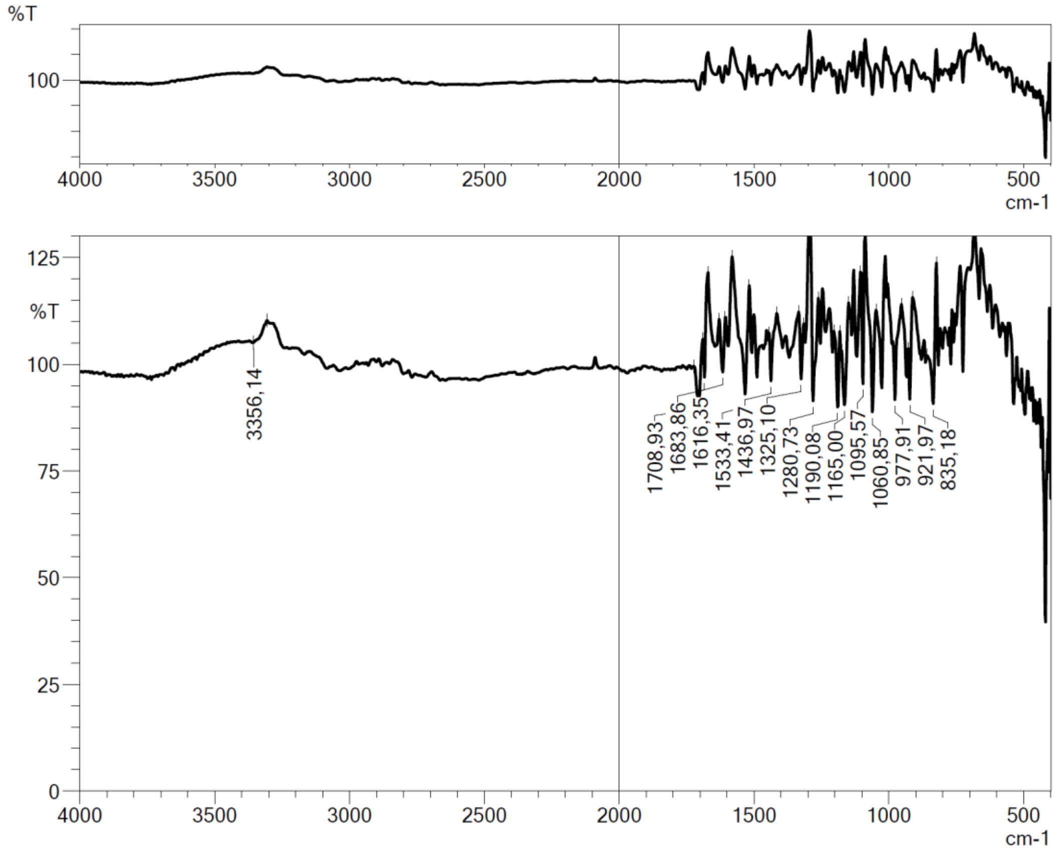

Figure S129. Compound **D20** IR report.

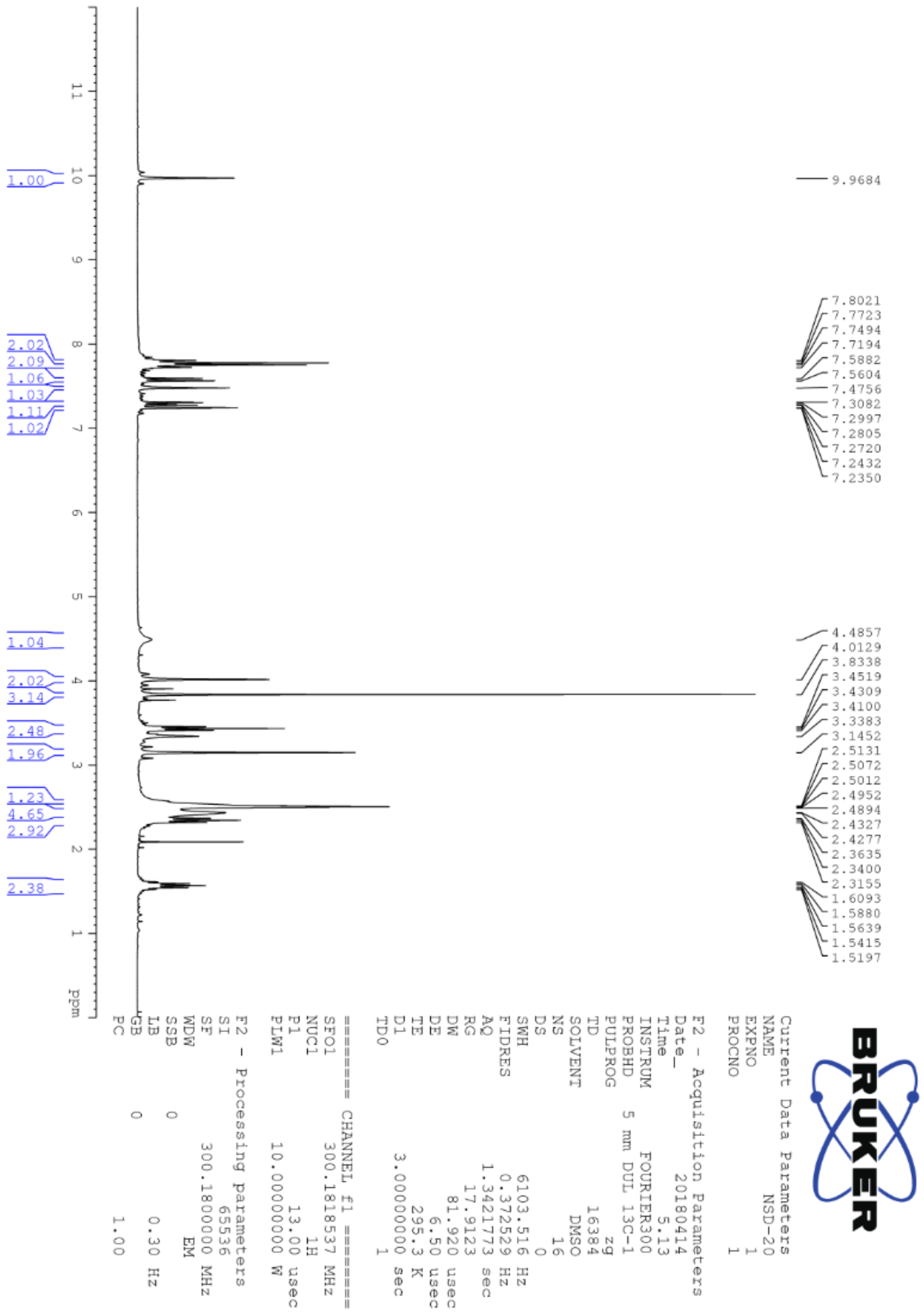

Figure S130. Compound **D20** ^{1}H-NMR spectrum.

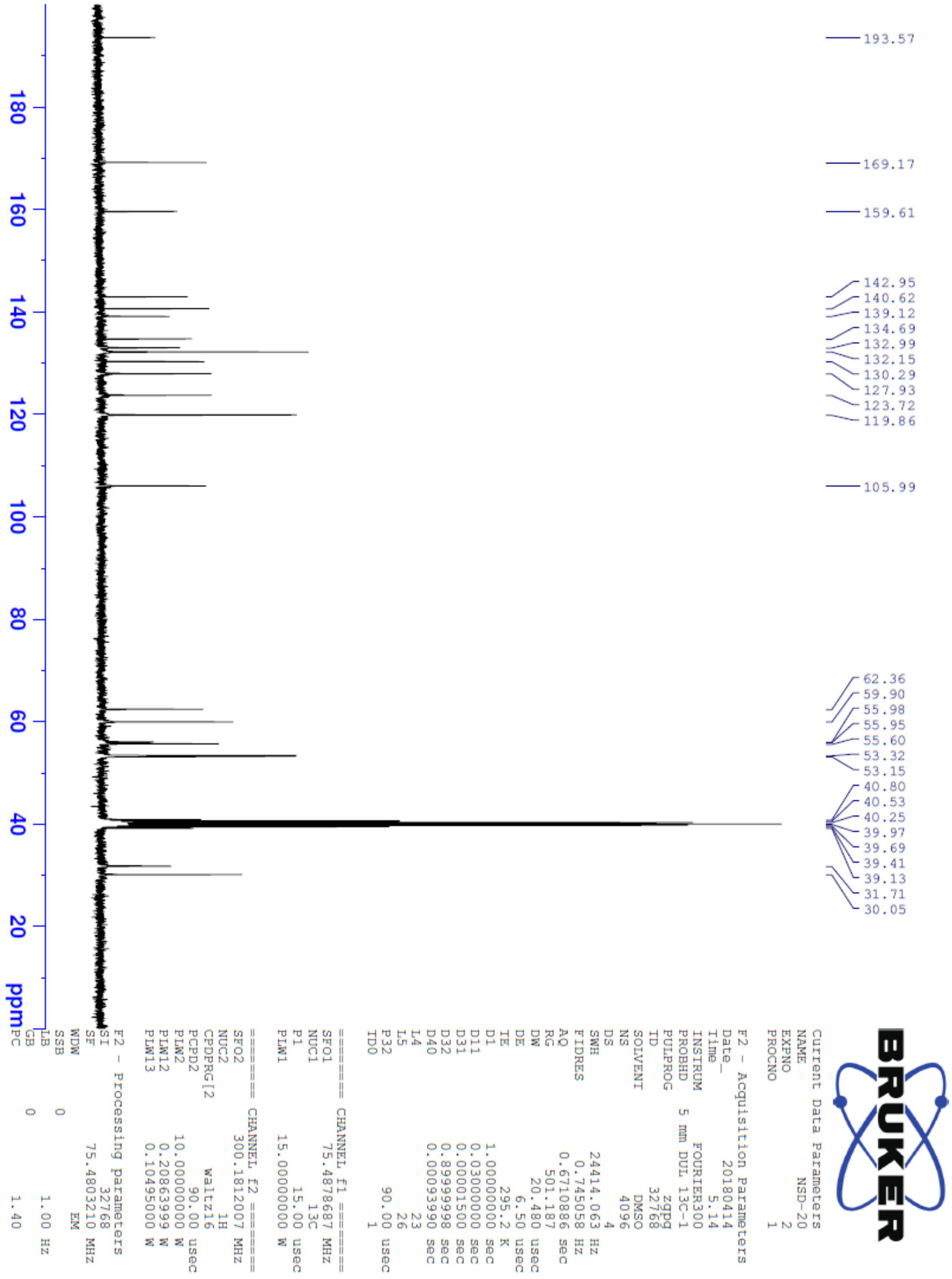

Figure S131. Compound **D20** ^{13}C-NMR spectrum.

Data File: C:\LabSolutions\Data\Analiz\Serkan\NSD-20_58.lcd

Elmt	Val.	Min	Max	Elmt	Val.	Min	Max	Elmt	Val.	Min	Max	Elmt	Val.	Min	Max	Use Adduct
H	1	5	40	O	2	3	5	S	2	0	0	Ru	2	0	0	H
C	4	0	35	F	1	0	0	Cl	1	0	0	I	3	0	0	
N	3	3	6	P	3	0	0	Br	1	0	0					

Error Margin (ppm):	5	DBE Range:	10.0 - 17.0	Electron Ions:	both
HC Ratio:	unlimited	Apply N Rule:	yes	Use MSn Info:	yes
Max Isotopes:	3	Isotope RI (%):	1.00	Isotope Res:	9000
MSn Iso RI (%):	10.00	MSn Logic Mode:	AND	Max Results:	500

Event#: 1 MS(E+) Ret. Time : 2.147 -> 2.320 Scan# : 323 -> 349

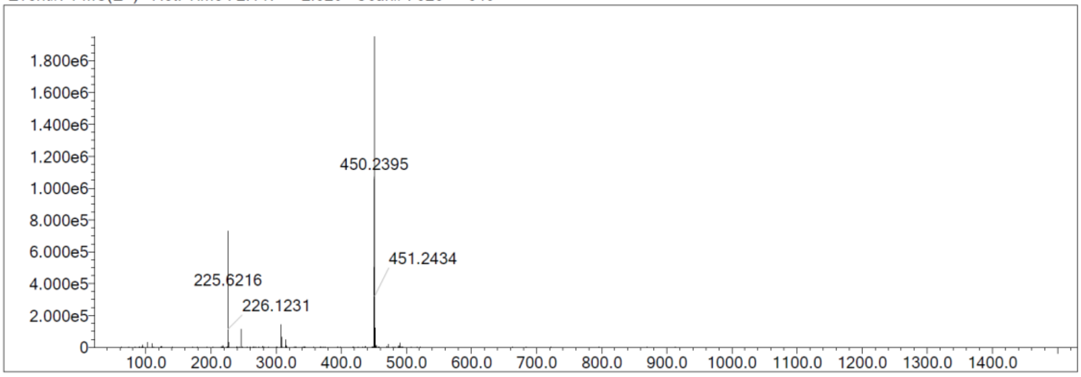

Measured region for 450.2395 m/z

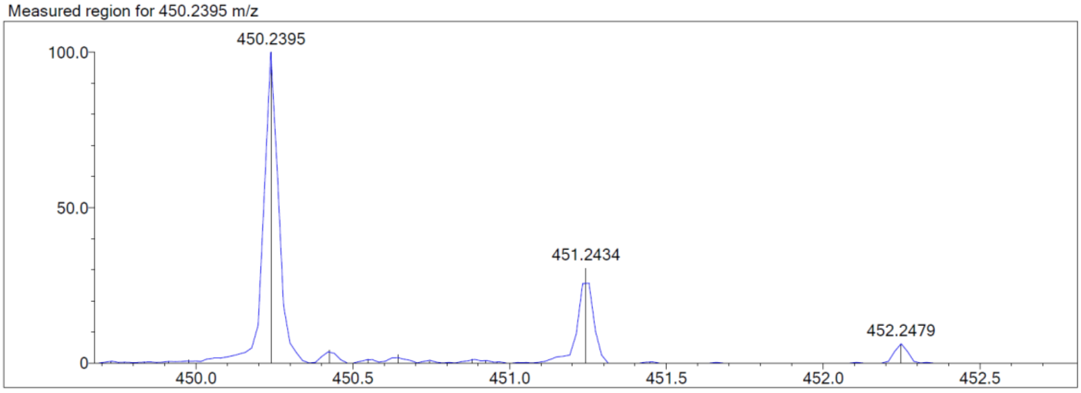

C26 H31 N3 O4 [M+H]+ : Predicted region for 450.2387 m/z

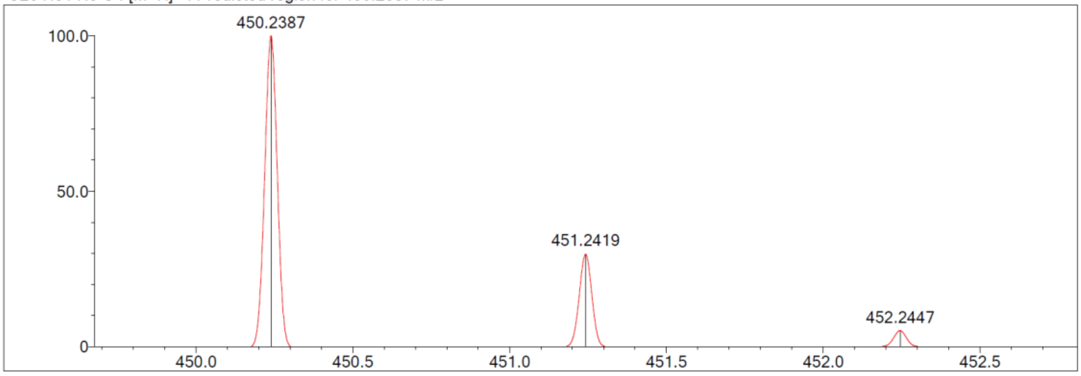

Rank	Score	Formula (M)	Ion	Meas. m/z	Pred. m/z	Df. (mDa)	Df. (ppm)	Iso	DBE
1	94.61	C26 H31 N3 O4	[M+H]+	450.2395	450.2387	0.8	1.78	96.49	13.0

Figure S132. Compound **D20** HRMS report.

*2-(4-(3-Hydroxypropyl)piperazine-1-yl)-N-(4-((5,6-dimethoxy-1-oxo-2,3-dihydro-1H-inden-2-ylidene)methyl) phenyl)acetamide (**D21**)*

Yellow powder. M.P.: 165.9 °C. Yield: 87%.

IR (ATR) ν_{max} (cm^{-1}): 3361 (N-H), 1749 (indanone C=O), 1653 (amide C=O), 1533-1469 (C=C), 1220 (C-N), 1069 (C-O), 848 (1,4-disubstituted benzene).

^{1}H-NMR (300 MHz, DMSO-*d$_6$*) δ (ppm): 1.57 (2H, p, *J*=6.62 Hz, CH_2), 2.34 (2H, t, *J*=7.19 Hz, CH_2), 2.43 (8H, bs, CH_2, piperazine CH_2), 3.14 (2H, s, CH_2), 3.43 (2H, t, *J*=6.29 Hz, CH_2), 3.83 (3H, s, OCH_3), 3.90 (3H, s, OCH_3), 3.98 (2H, s, CH_2), 4.44 (1H, s, OH), 7.20 (1H, s, methoxy-1-oxo-indenylidene CH), 7.21 (1H, s, methoxy-1-oxo-indenylidene CH), 7.38 (1H, s, C=CH), 7.70 (2H, d, *J*=8.86 Hz, disubstituted benzene CH), 7.77 (2H, d, *J*=8.80 Hz, disubstituted benzene CH), 9.91 (1H, s, NH).

^{13}C-NMR (75 MHz, DMSO-*d$_6$*) δ (ppm): 30.0, 32.1, 53.2, 53.3, 55.6, 56.1, 56.5, 59.9, 62.3, 105.1, 108.5, 119.9, 130.5, 130.6, 131.3, 131.8, 134.8, 140.3, 145.4, 149.8, 155.7, 169.1, 192.3.

HRMS (ESI) (m/z) [M+H]$^+$: $C_{27}H_{33}N_3O_5$ calculated: 480.2493, found: 480.2507.

DOPNALAB

	Item	Value
	Acquired Date&Time	22.08.2019 12:09:04
	Acquired by	System Administrator
	Filename	C:\Users\dopnalab\Desktop\NURPELİN\DOKTORA TEZ\D211.ispd
	Spectrum name	D211
	Sample name	D21
	Sample ID	
	Option	
	Comment	
	No. of Scans	50
	Resolution	4 [cm-1]
	Apodization	Happ-Genzel

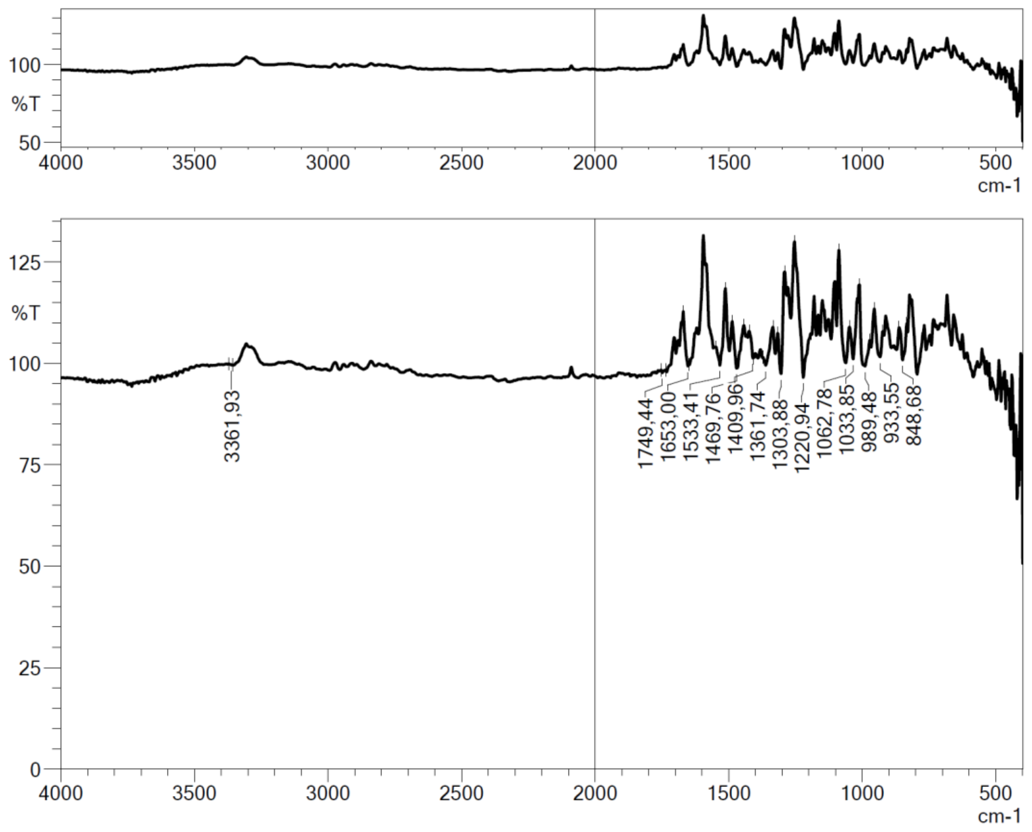

Figure S133. Compound **D21** IR report.

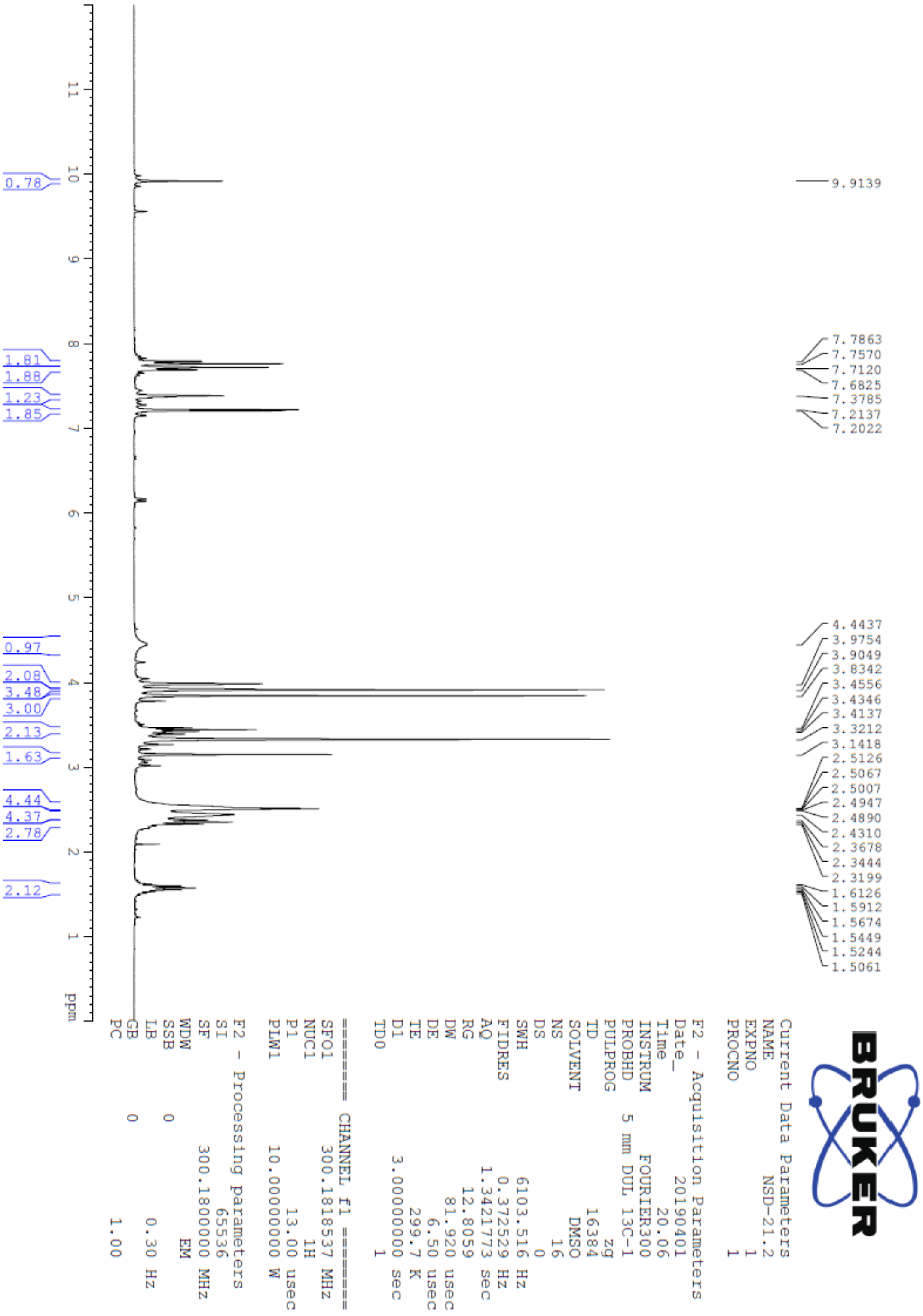

Figure S134. Compound **D21** ^{1}H-NMR spectrum.

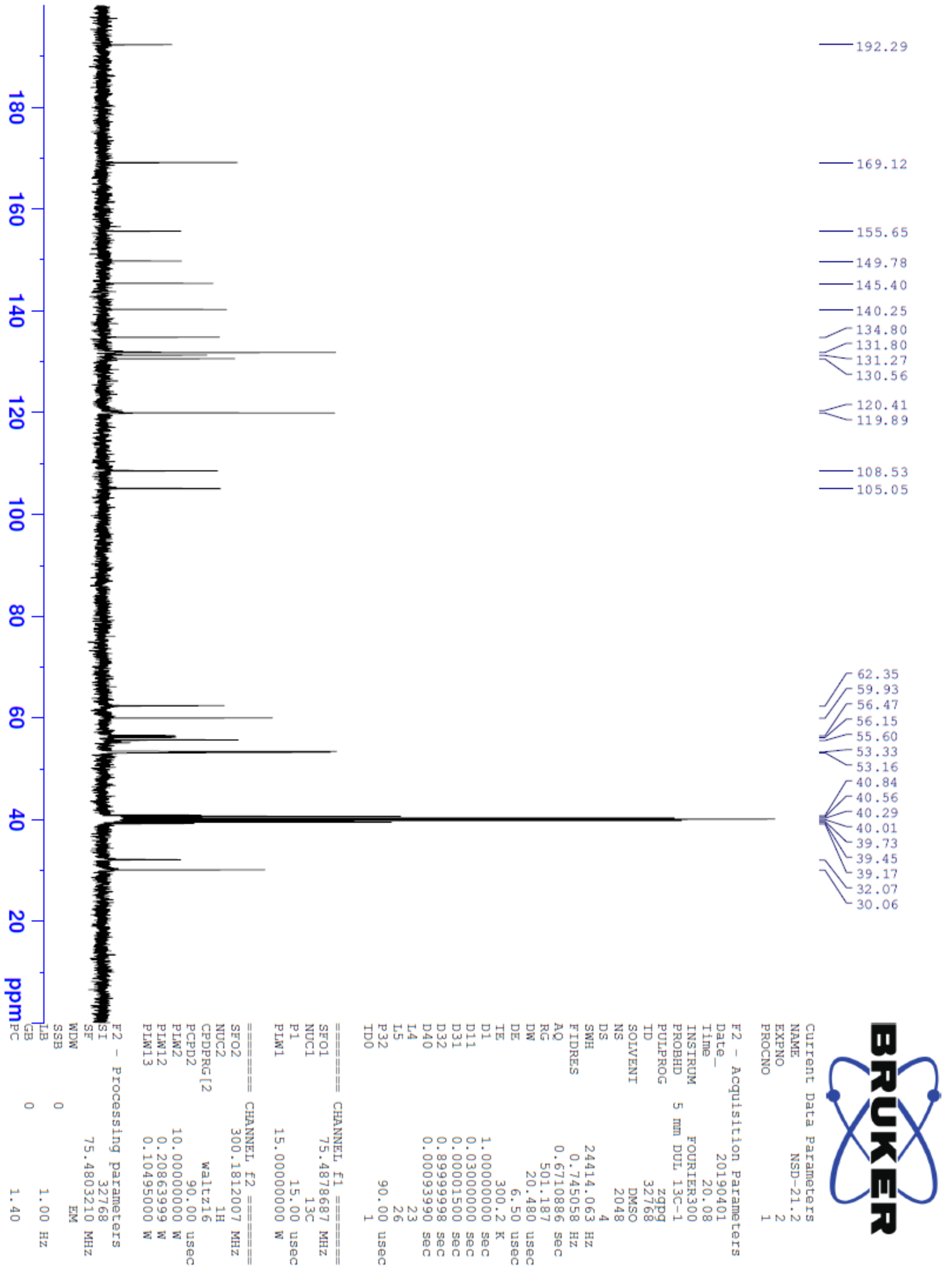

Figure S135. Compound **D21** ^{13}C-NMR spectrum.

Data File: C:\LabSolutions\Data\Analiz\Serkan\NSD-21_59.lcd

Elmt	Val.	Min	Max	Elmt	Val.	Min	Max	Elmt	Val.	Min	Max	Elmt	Val.	Min	Max	Use Adduct
H	1	5	40	O	2	3	5	S	2	0	0	Ru	2	0	0	H
C	4	0	35	F	1	0	0	Cl	1	0	0	I	3	0	0	
N	3	3	6	P	3	0	0	Br	1	0	0					

Error Margin (ppm): 5
HC Ratio: unlimited
Max Isotopes: 3
MSn Iso RI (%): 10.00

DBE Range: 10.0 - 17.0
Apply N Rule: yes
Isotope RI (%): 1.00
MSn Logic Mode: AND

Electron Ions: both
Use MSn Info: yes
Isotope Res: 9000
Max Results: 500

Event#: 1 MS(E+) Ret. Time : 2.107 -> 2.307 Scan# : 317 -> 347

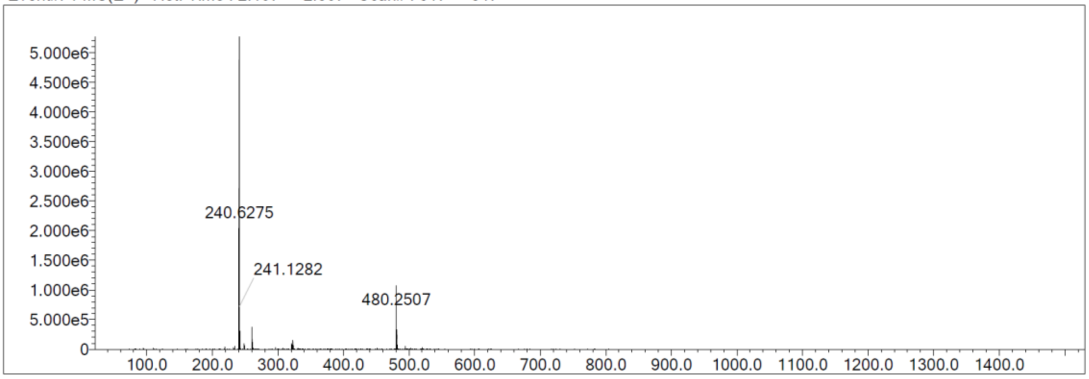

Measured region for 480.2507 m/z

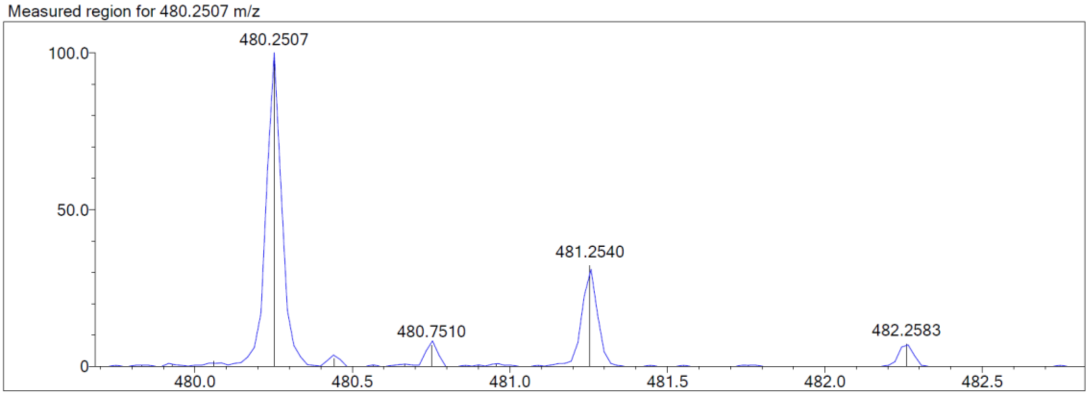

C27 H33 N3 O5 [M+H]+ : Predicted region for 480.2493 m/z

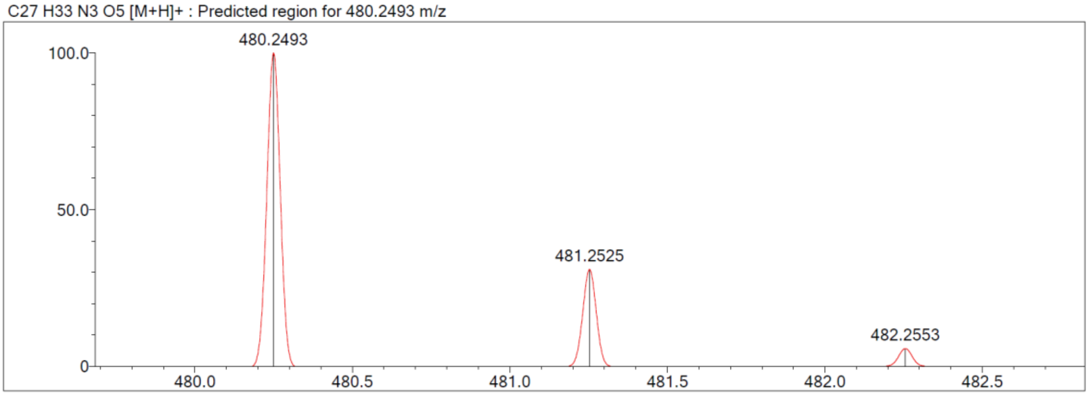

Rank	Score	Formula (M)	Ion	Meas. m/z	Pred. m/z	Df. (mDa)	Df. (ppm)	Iso	DBE
1	94.20	C27 H33 N3 O5	[M+H]+	480.2507	480.2493	1.4	2.92	98.95	13.0

Figure S136. Compound **D21** HRMS report.

*2-(4-(2-(Dimethylamino)ethyl)piperazine-1-yl)-N-(4-((5-methoxy-1-oxo-2,3-dihydro-1H-inden-2-ylidene)methyl)phenyl)acetamide (**D22**)*

Dark orange powder. M.P.: 206.3 °C. Yield: 77%.

IR (ATR) ν_{max} (cm^{-1}): 3340 (N-H), 1697 (indanone C=O), 1645 (amide C=O), 1541-1489 (C=C), 1159 (C-N), 1026 (C-O), 840 (1,4-disubstituted benzene).

^{1}H-NMR (300 MHz, DMSO-d_6) δ (ppm): 2.13 (6H, s, CH_3), 2.32-2.40 (4H, m, CH_2), 2.59 (8H, bs, CH_2, piperazine CH_2), 3.14 (2H, m, CH_2), 3.90 (3H, s, OCH_3), 4.06 (2H, s, CH_2), 7.03 (1H, dd, J_1=8.49 Hz, J_2=2.19 Hz, methoxy-1-oxo-indenylidene CH), 7.18 (1H, J=1.89 Hz, methoxy-1-oxo-indenylidene CH), 7.40 (1H, s, C=CH), 7.70-7.73 (3H, m, disubstituted benzene CH, methoxy-1-oxo-indenylidene CH), 7.79 (2H, d, J=8.82 Hz, disubstituted benzene CH), 9.93 (1H, s, NH).

^{13}C-NMR (75 MHz, DMSO-d_6) δ (ppm): 32.5, 46.0, 53.4, 56.3, 56.4, 57.1, 62.4, 110.6, 115.8, 119.9, 125.8, 130.5, 131.2, 131.7, 131.9, 134.5, 140.4, 153.3, 165.3, 169.1, 192.0.

HRMS (ESI) (m/z) $[M+H]^+$: $C_{27}H_{34}N_4O_3$ calculated: 463.2704, found: 463.2687.

DOPNALAB

	Item	Value
	Acquired Date&Time	22.08.2019 12:11:59
	Acquired by	System Administrator
	Filename	C:\Users\dopnalab\Desktop\NURPELİN\DOKTORA TEZ\D221.ispd
	Spectrum name	D221
	Sample name	D22
	Sample ID	
	Option	
	Comment	
	No. of Scans	50
	Resolution	4 [cm-1]
	Apodization	Happ-Genzel

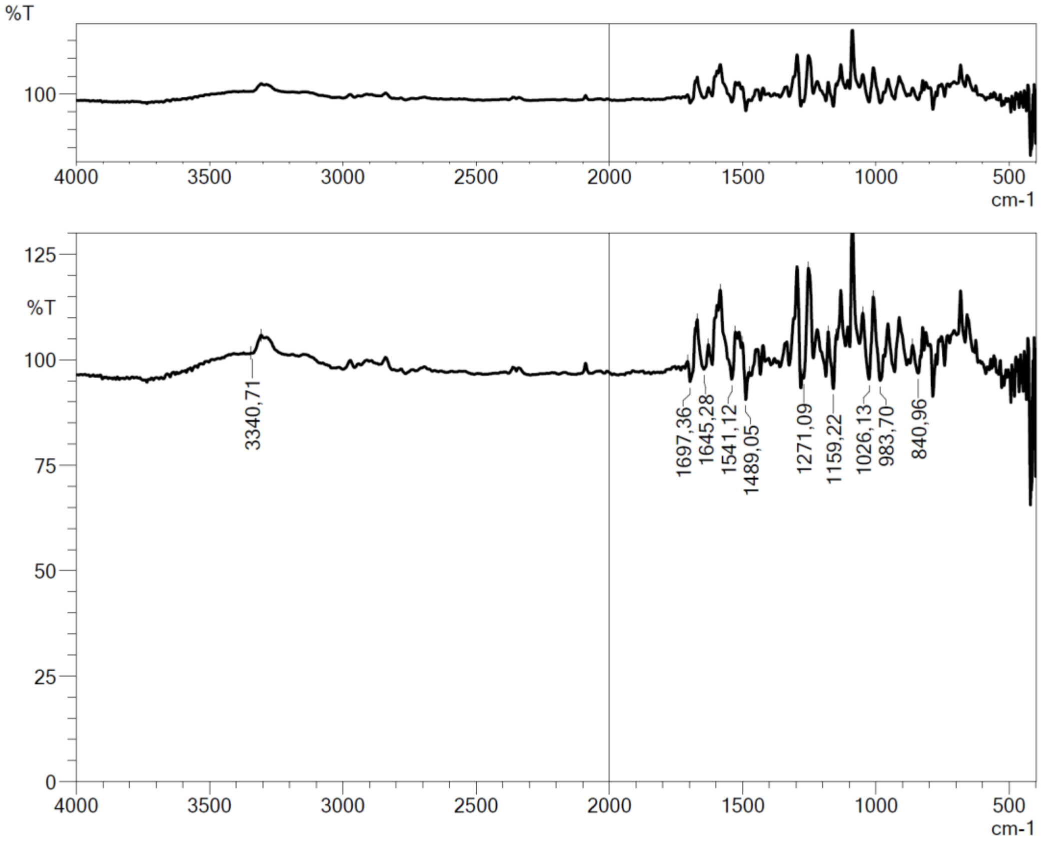

Figure S137. Compound **D22** IR report.

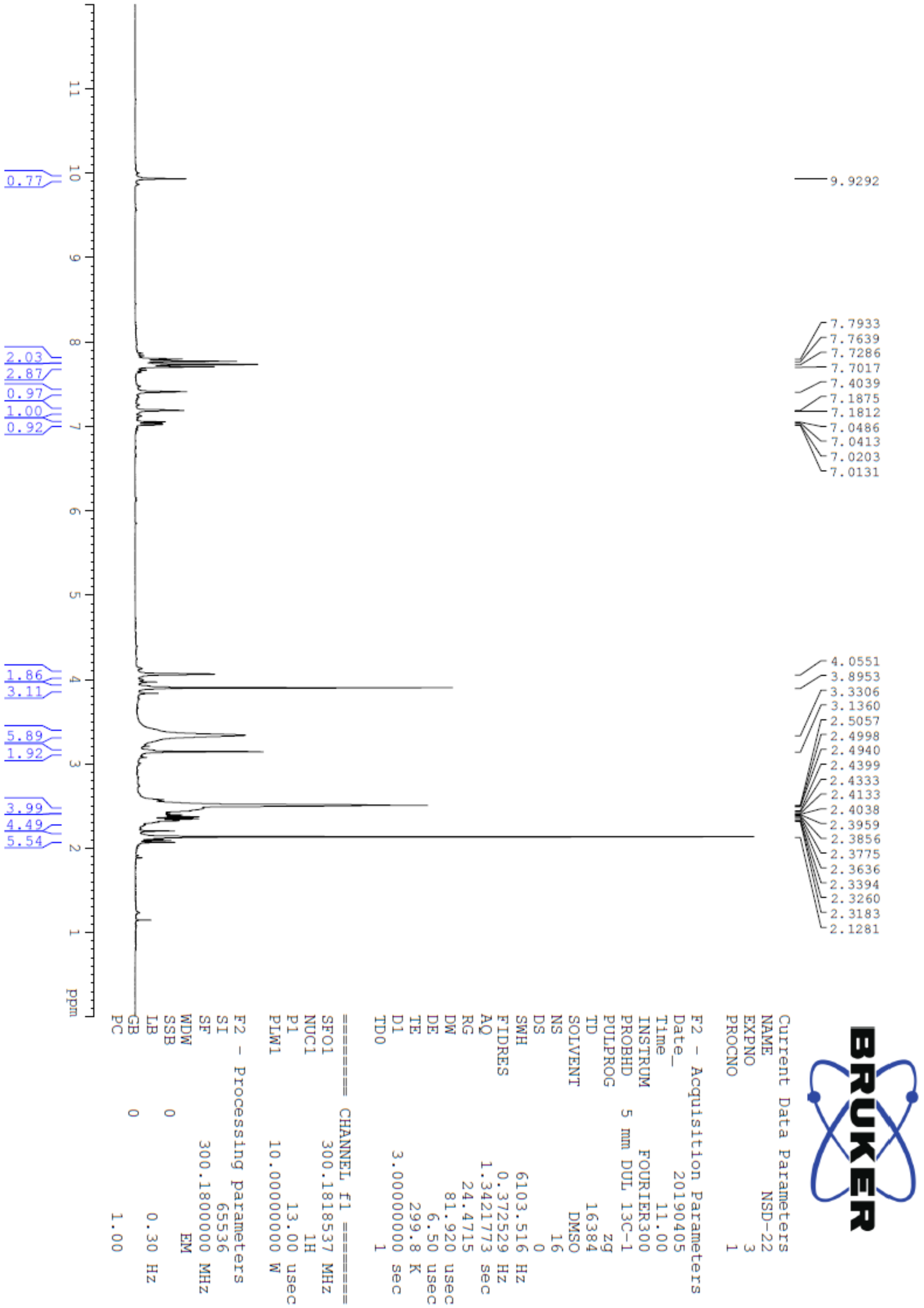

Figure S138. Compound **D22** ^{1}H-NMR spectrum.

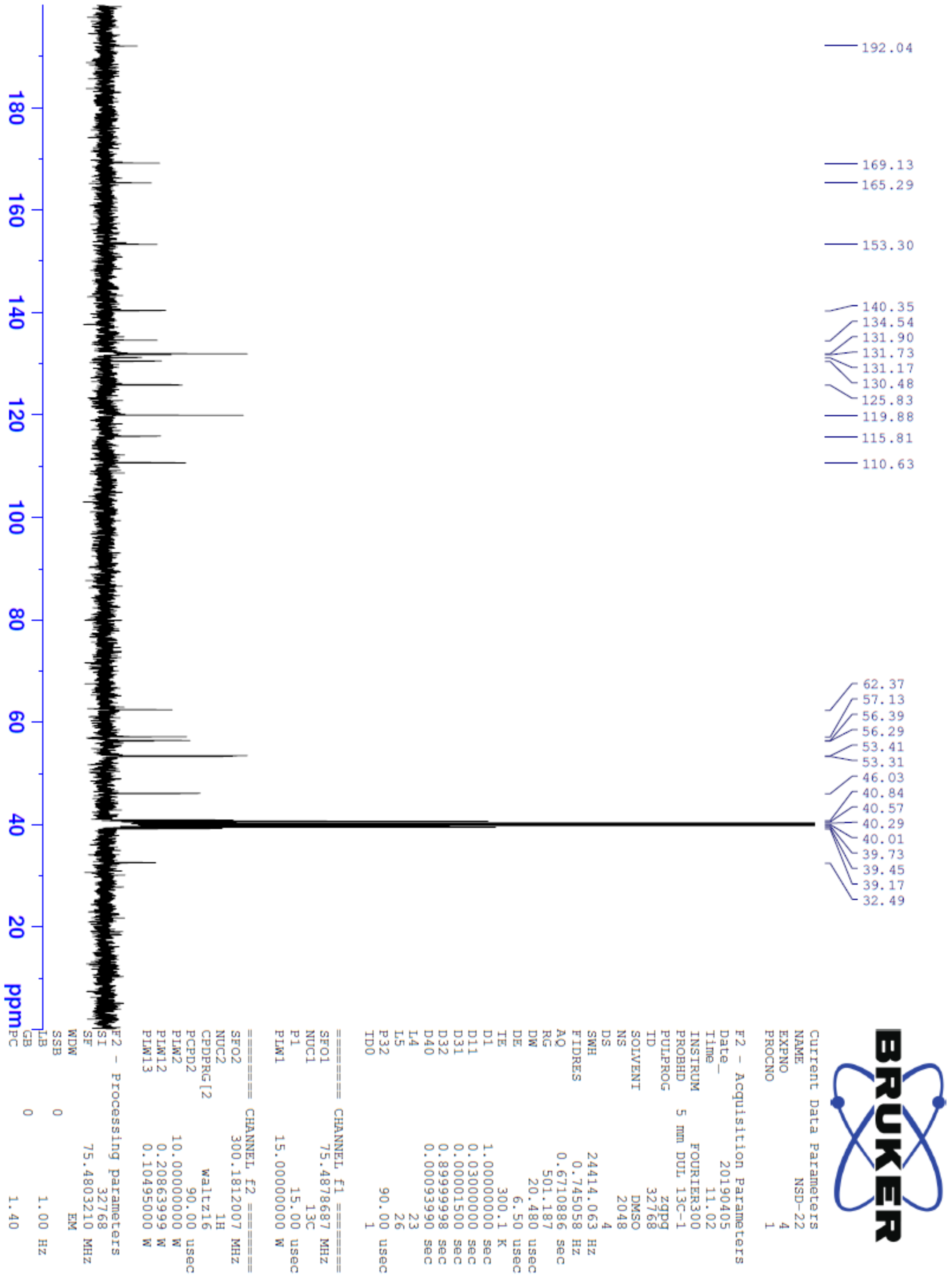

Figure S139. Compound **D22** ^{13}C-NMR spectrum.

Data File: C:\LabSolutions\Data\Analiz\bns\NSD-22_4.lcd

Elmt	Val.	Min	Max	Elmt	Val.	Min	Max	Elmt	Val.	Min	Max	Elmt	Val.	Min	Max	Use Adduct
H	1	5	40	O	2	3	5	S	2	0	0	Ru	2	0	0	H
C	4	0	35	F	1	0	0	Cl	1	0	0	I	3	0	0	
N	3	3	6	P	3	0	0	Br	1	0	0					

Error Margin (ppm): 5
HC Ratio: unlimited
Max Isotopes: 3
MSn Iso RI (%): 10.00

DBE Range: 10.0 - 17.0
Apply N Rule: yes
Isotope RI (%): 1.00
MSn Logic Mode: AND

Electron Ions: both
Use MSn Info: yes
Isotope Res: 9000
Max Results: 500

Event#: 1 MS(E+) Ret. Time : 4.600 -> 4.600 Scan# : 691 -> 691

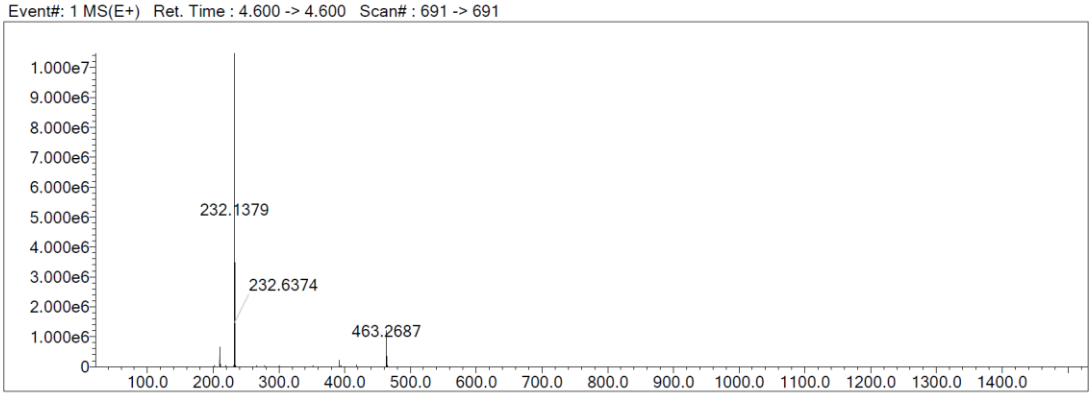

Measured region for 463.2687 m/z

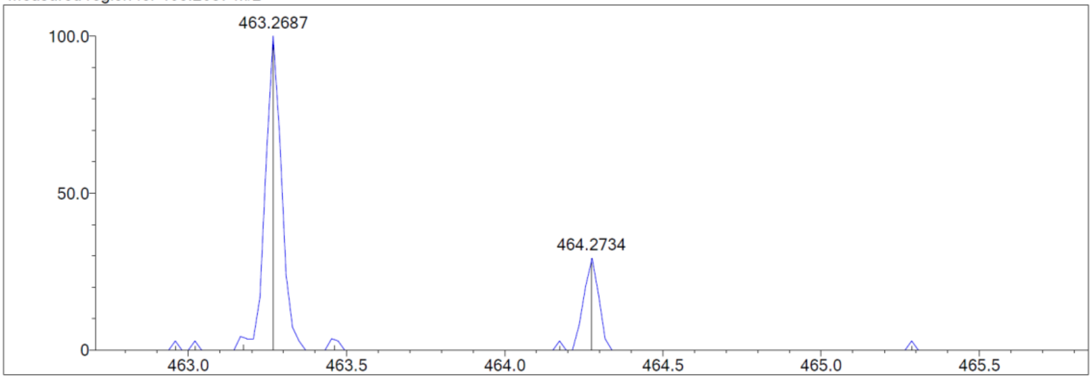

C27 H34 N4 O3 [M+H]+ : Predicted region for 463.2704 m/z

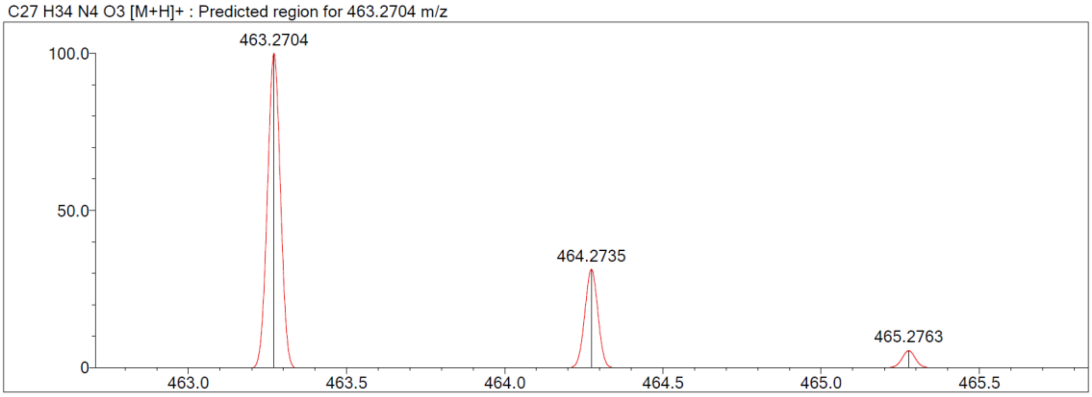

Rank	Score	Formula (M)	Ion	Meas. m/z	Pred. m/z	Df. (mDa)	Df. (ppm)	Iso	DBE
1	58.97	C27 H34 N4 O3	[M+H]+	463.2687	463.2704	-1.7	-3.67	63.19	13.0

Figure S140. Compound **D22** HRMS report.

*2-(4-(2-(Dimethylamino)ethyl)piperazine-1-yl)-N-(4-((6-methoxy-1-oxo-2,3-dihydro-1H-inden-2-ylidene)methyl)phenyl)acetamide (**D23**)*

Brown powder. M.P.: 107.4 °C. Yield: 81%.

IR (ATR) ν_{max} (cm^{-1}): 3340 (N-H), 1697 (indanone C=O), 1647 (amide C=O), 1541-1489 (C=C), 1188 (C-N), 1026 (C-O), 840 (1,4-disubstituted benzene).

^{1}H-NMR (300 MHz, DMSO-d_6) δ (ppm): 2.12 (6H, s, CH_3), 2.31-2.38 (4H, m, CH_2), 2.51 (8H, bs, CH_2, piperazine CH_2), 3.14 (2H, m, CH_2), 3.83 (3H, s, OCH_3), 3.99 (2H, s, CH_2), 7.23 (1H, *J*=2.46 Hz, methoxy-1-oxo-indenylidene CH), 7.28 (1H, dd, J_1=8.28 Hz, J_2=2.55 Hz, methoxy-1-oxo-indenylidene CH), 7.47 (1H, s, C=CH), 7.47 (1H, *J*=8.35 Hz, methoxy-1-oxo-indenylidene CH), 7.72 (2H, d, *J*=9.00 Hz, disubstituted benzene CH), 7.78 (2H, d, *J*=8.86 Hz, disubstituted benzene CH), 9.97 (1H, s, NH).

^{13}C-NMR (75 MHz, DMSO-d_6) δ (ppm): 31.7, 46.0, 53.3, 53.4, 56.0, 56.4, 57.1, 62.4, 106.0, 119.9, 123.7, 127.9, 130.3, 132.1, 133.0, 134.7, 139.1, 140.6, 142.9, 159.6, 169.2, 193.5.

HRMS (ESI) (m/z) $[M+H]^+$: $C_{27}H_{34}N_4O_3$ calculated: 463.2704, found: 463.2714.

DOPNALAB

	Item	Value
	Acquired Date&Time	22.08.2019 12:14:40
	Acquired by	System Administrator
	Filename	C:\Users\dopnalab\Desktop\NURPELİN\DOKTORA TEZ\D231.ispd
	Spectrum name	D231
	Sample name	D23
	Sample ID	
	Option	
	Comment	
	No. of Scans	50
	Resolution	4 [cm-1]
	Apodization	Happ-Genzel

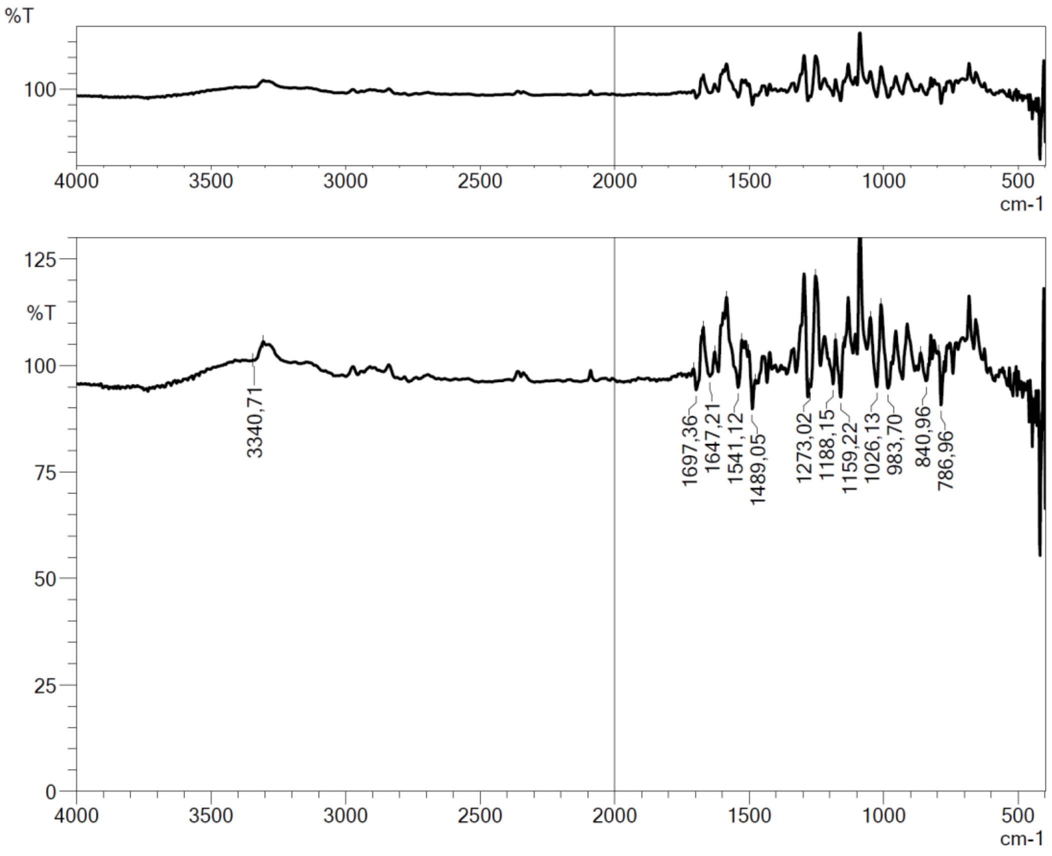

Figure S141. Compound **D23** IR report.

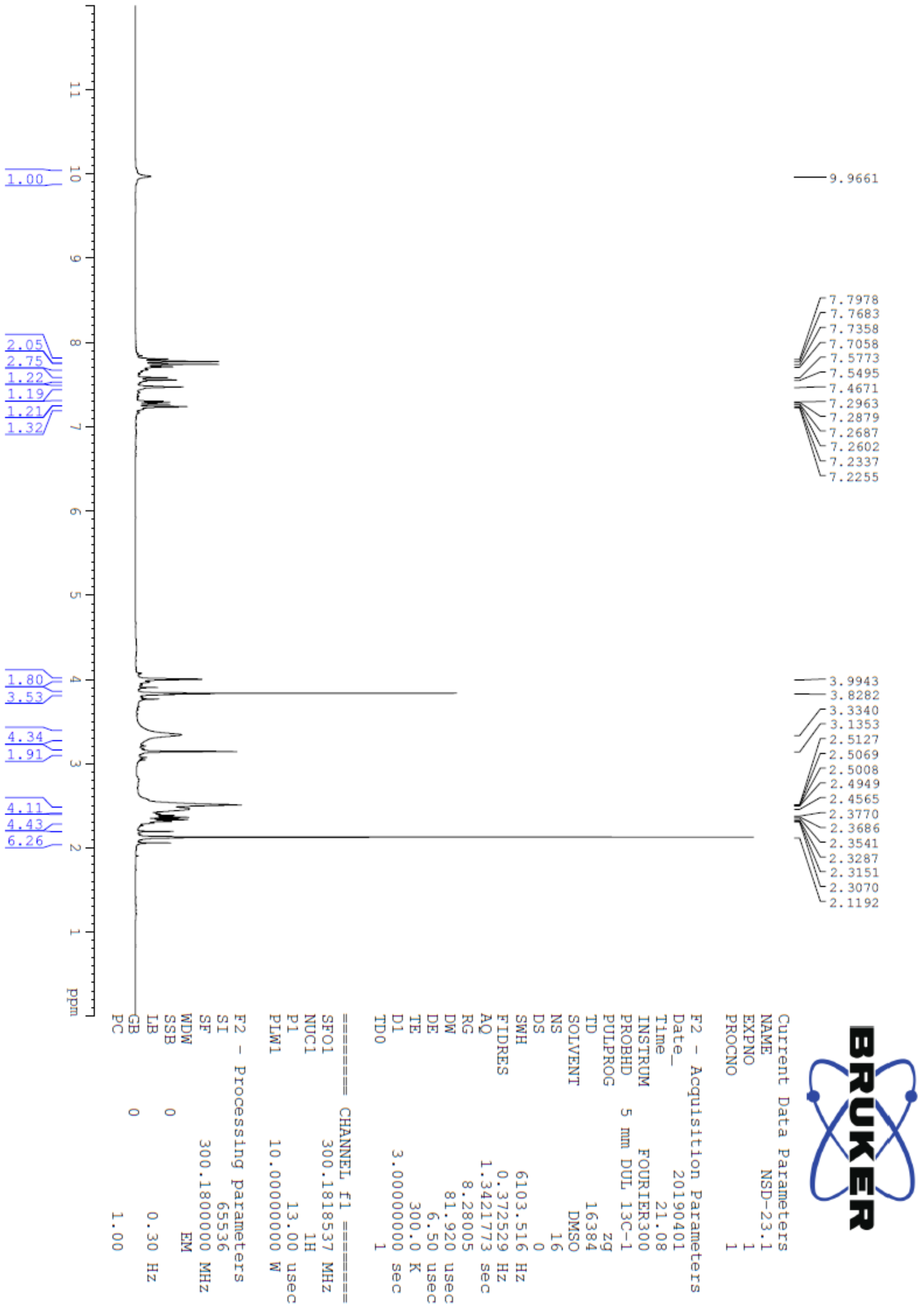

Figure S142. Compound **D23** ^{1}H-NMR spectrum.

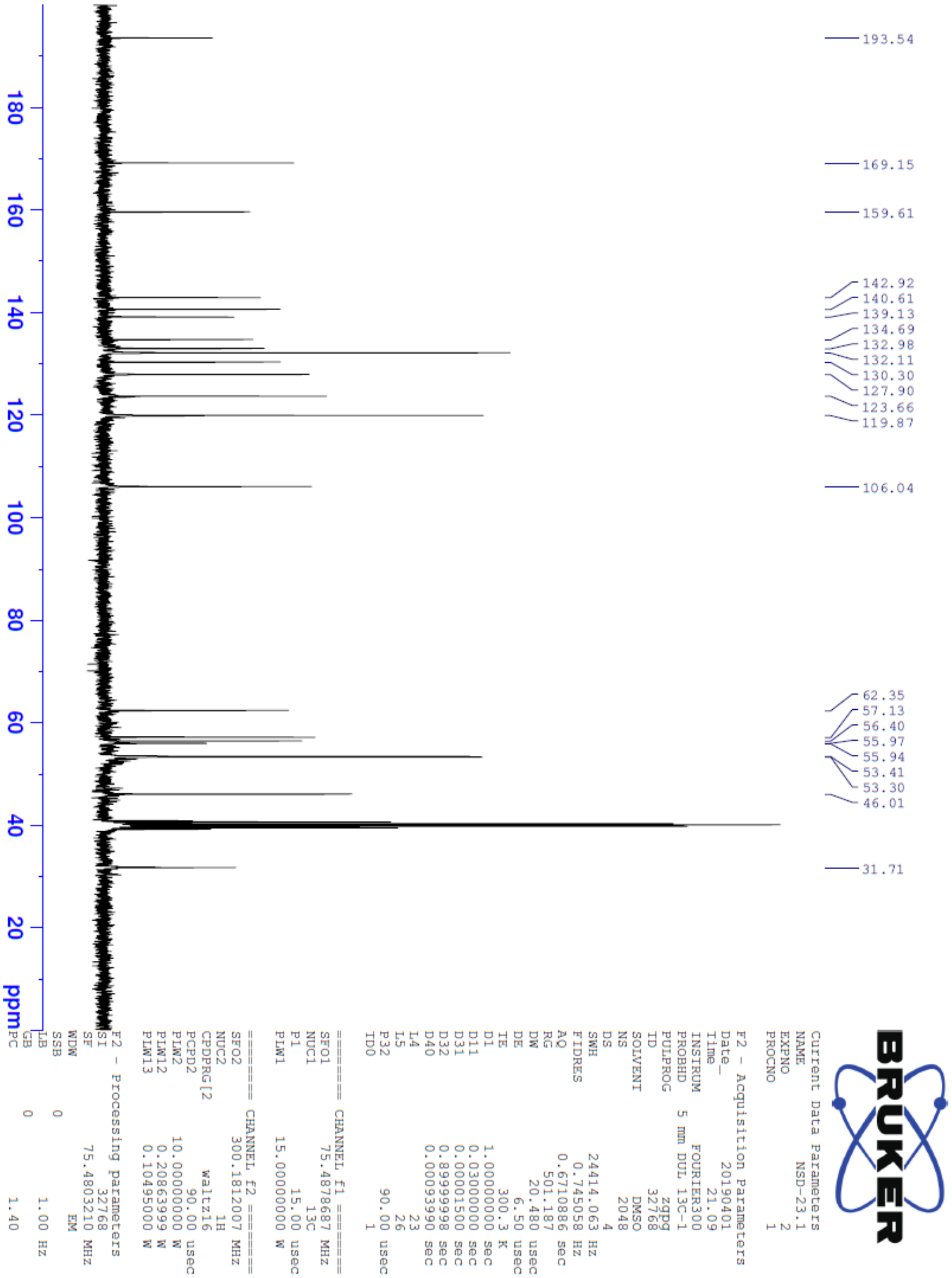

Figure S143. Compound **D23** ^{13}C-NMR spectrum.

Data File: C:\LabSolutions\Data\Analiz\Serkan\NSD-23_63.lcd

Elmt	Val.	Min	Max	Elmt	Val.	Min	Max	Elmt	Val.	Min	Max	Elmt	Val.	Min	Max	Use Adduct
H	1	5	40	O	2	3	5	S	2	0	0	Ru	2	0	0	H
C	4	0	35	F	1	0	0	Cl	1	0	0	I	3	0	0	
N	3	3	6	P	3	0	0	Br	1	0	0					

Error Margin (ppm): 5
HC Ratio: unlimited
Max Isotopes: 3
MSn Iso RI (%): 10.00

DBE Range: 10.0 - 17.0
Apply N Rule: yes
Isotope RI (%): 1.00
MSn Logic Mode: AND

Electron Ions: both
Use MSn Info: yes
Isotope Res: 9000
Max Results: 500

Event#: 1 MS(E+) Ret. Time : 1.493 -> 1.733 Scan# : 225 -> 261

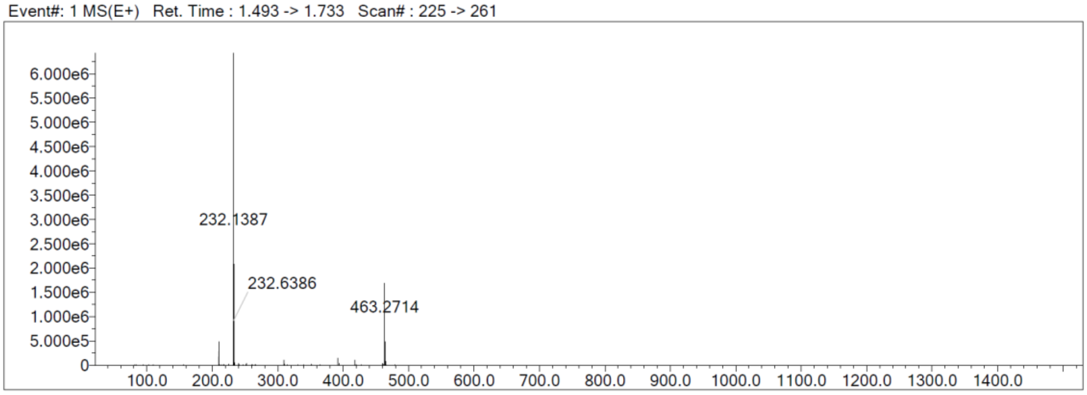

Measured region for 463.2714 m/z

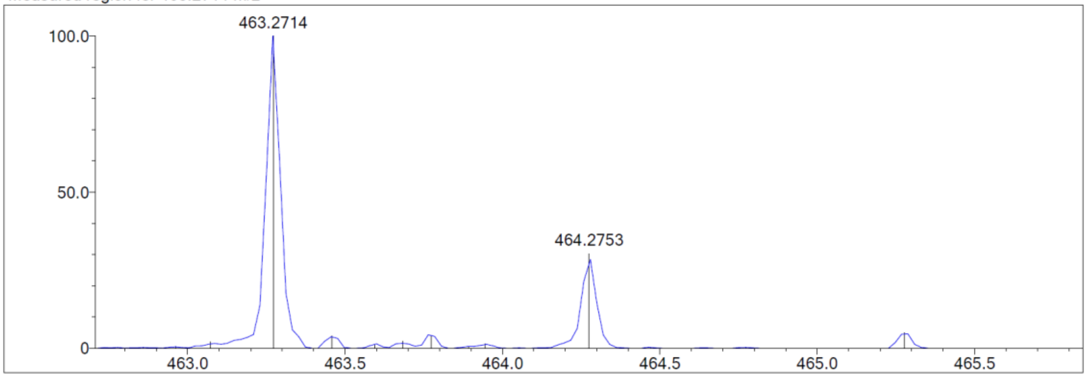

C27 H34 N4 O3 [M+H]+ : Predicted region for 463.2704 m/z

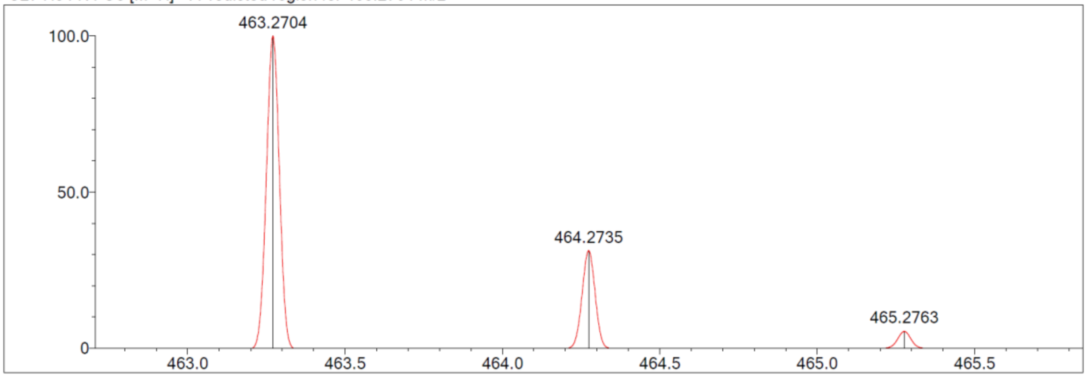

Rank	Score	Formula (M)	Ion	Meas. m/z	Pred. m/z	Df. (mDa)	Df. (ppm)	Iso	DBE
1	92.31	C27 H34 N4 O3	[M+H]+	463.2714	463.2704	1.0	2.16	95.06	13.0

Figure S144. Compound **D23** HRMS report.

*2-(4-(2-(Dimethylamino)ethyl)piperazine-1-yl)-N-(4-((5,6-dimethoxy-1-oxo-2,3-dihydro-1H-inden-2-ylidene))methyl)phenyl)acetamide (**D24**)*

Brown powder. M.P.: 98.2 °C. Yield: 88%.

IR (ATR) ν$_{max}$ (cm^{-1}): 3334 (N-H), 1697 (indanone C=O), 1637 (amide C=O), 1541-1458 (C=C), 1219 (C-N), 1062 (C-O), 842 (1,4-disubstituted benzene).

^{1}H-NMR (300 MHz, DMSO-*d$_6$*) δ (ppm): 2.12 (6H, s, CH_3), 2.31-2.38 (4H, m, CH_2), 2.51 (8H, bs, CH_2, piperazine CH_2), 3.14 (2H, m, CH_2), 3.83 (3H, s, OCH_3), 3.90 (3H, s, OCH_3), 3.96 (2H, s, CH_2), 7.19 (1H, s, methoxy-1-oxo-indenylidene CH), 7.20 (1H, s, methoxy-1-oxo-indenylidene CH), 7.37 (1H, s, C=CH), 7.69 (2H, d, *J*=8.85 Hz, disubstituted benzene CH), 7.77 (2H, d, *J*=8.74 Hz, disubstituted benzene CH), 9.94 (1H, s, NH).

^{13}C-NMR (75 MHz, DMSO-*d$_6$*) δ (ppm): 32.1, 46.0, 53.3, 53.4, 56.1, 56.4, 56.5, 57.1, 62.4, 105.0, 108.5, 119.9, 123.7, 127.9, 130.5, 130.6, 131.3, 131.8, 134.7, 140.3, 145.4, 149.7, 155.6, 169.1, 192.3.

HRMS (ESI) (m/z) [M+H]$^+$: $C_{28}H_{36}N_4O_4$ calculated: 493.2809, found: 493.2824.

DOPNALAB

	Item	Value
	Acquired Date&Time	22.08.2019 12:17:33
	Acquired by	System Administrator
	Filename	C:\Users\dopnalab\Desktop\NURPELİN\DOKTORA TEZ\D241.ispd
	Spectrum name	D241
	Sample name	D24
	Sample ID	
	Option	
	Comment	
	No. of Scans	50
	Resolution	4 [cm-1]
	Apodization	Happ-Genzel

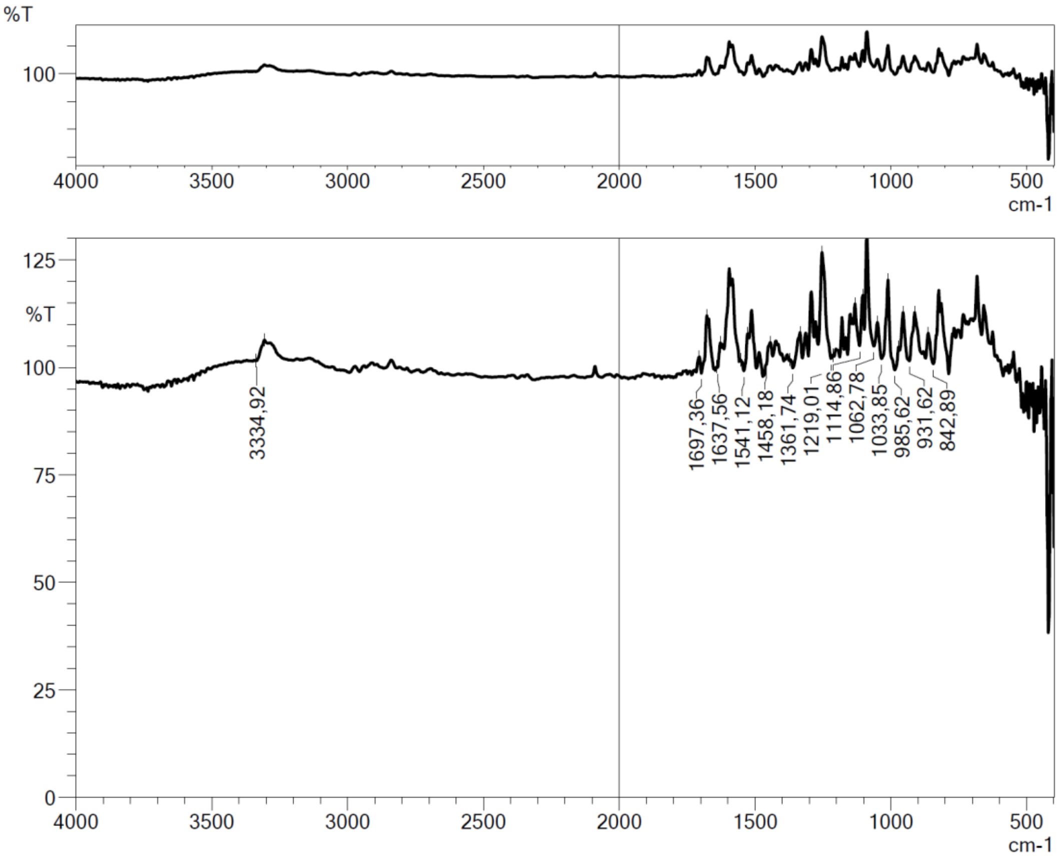

Figure S145. Compound **D24** IR report.

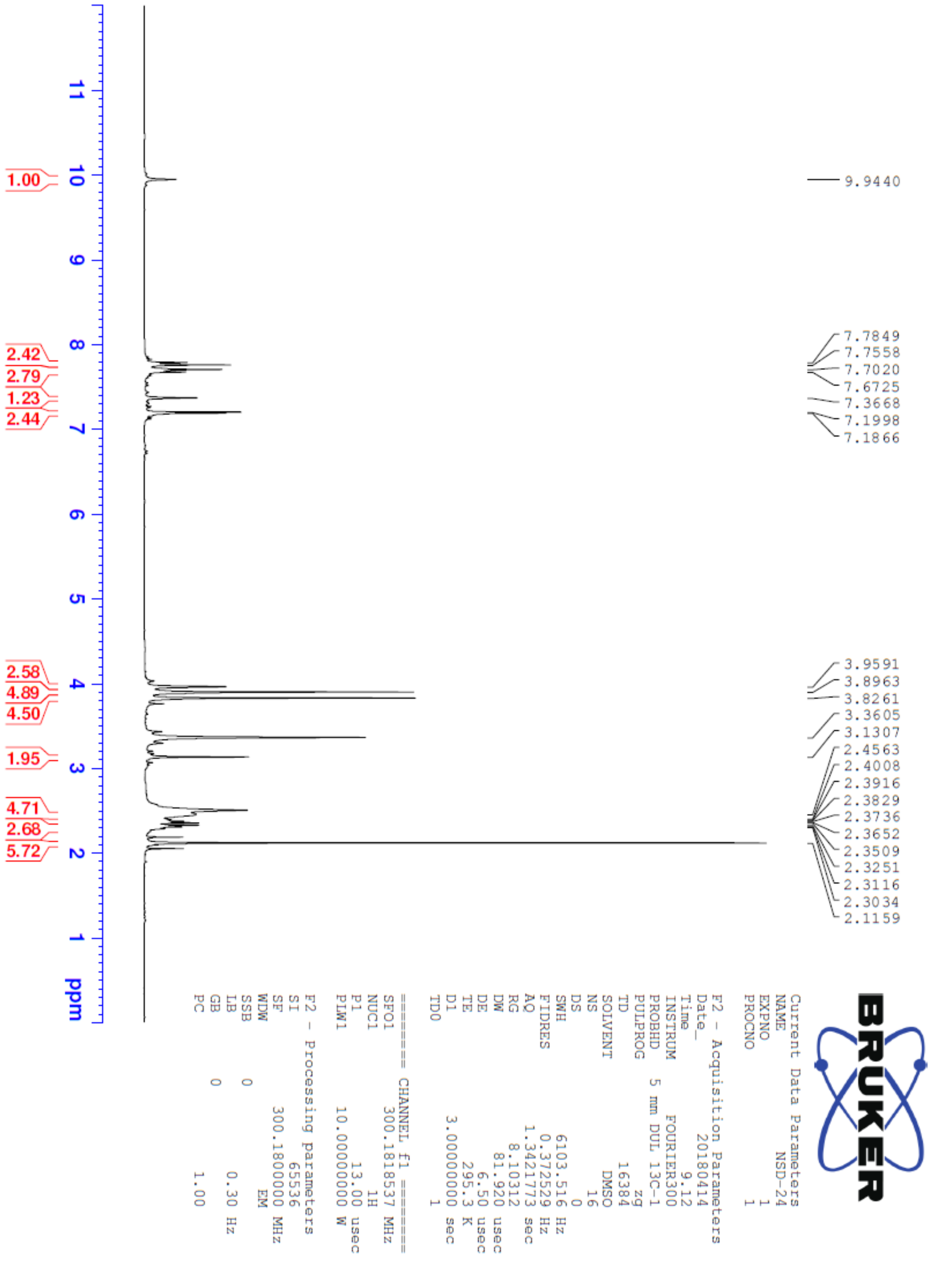

Figure S146. Compound **D24** ^{1}H-NMR spectrum.

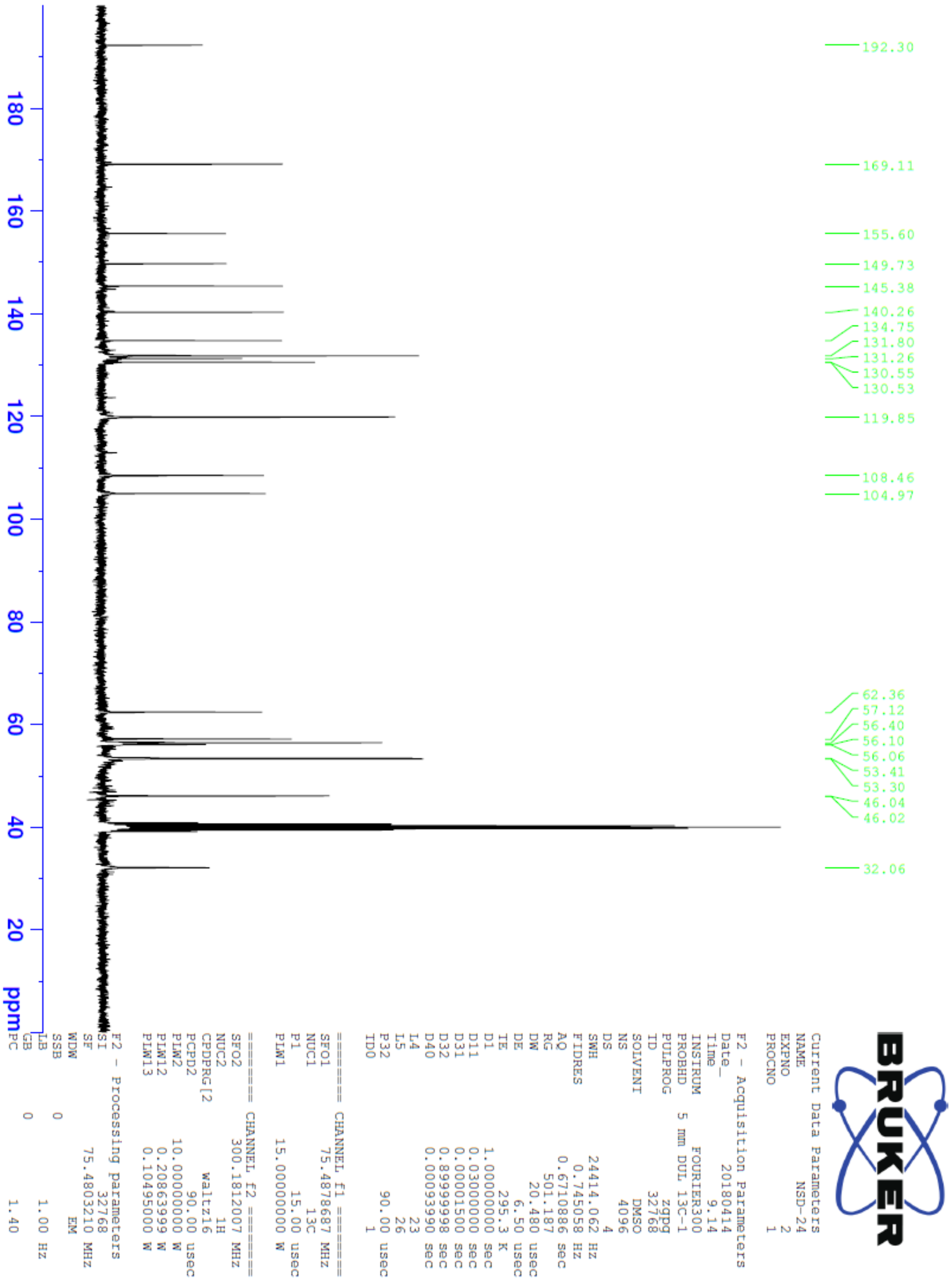

Figure S147. Compound **D24** ^{13}C-NMR spectrum.

Data File: C:\LabSolutions\Data\Analiz\Serkan\NSD-24_64.lcd

Elmt	Val.	Min	Max	Elmt	Val.	Min	Max	Elmt	Val.	Min	Max	Elmt	Val.	Min	Max	Use Adduct
H	1	5	40	O	2	3	5	S	2	0	0	Ru	2	0	0	H
C	4	0	35	F	1	0	0	Cl	1	0	0	I	3	0	0	
N	3	3	6	P	3	0	0	Br	1	0	0					

Error Margin (ppm):	5	DBE Range:	10.0 - 17.0	Electron Ions:	both
HC Ratio:	unlimited	Apply N Rule:	yes	Use MSn Info:	yes
Max Isotopes:	3	Isotope RI (%):	1.00	Isotope Res:	9000
MSn Iso RI (%):	10.00	MSn Logic Mode:	AND	Max Results:	500

Event#: 1 MS(E+) Ret. Time : 1.413 -> 1.747 Scan# : 213 -> 263

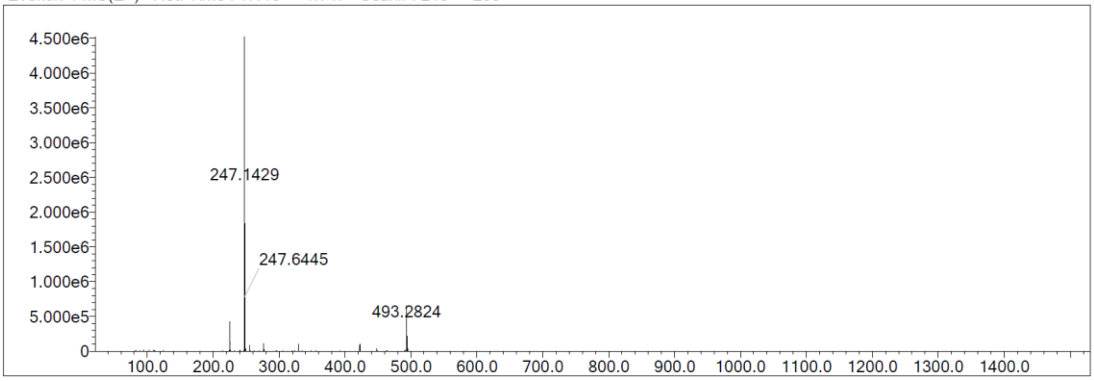

Measured region for 493.2824 m/z

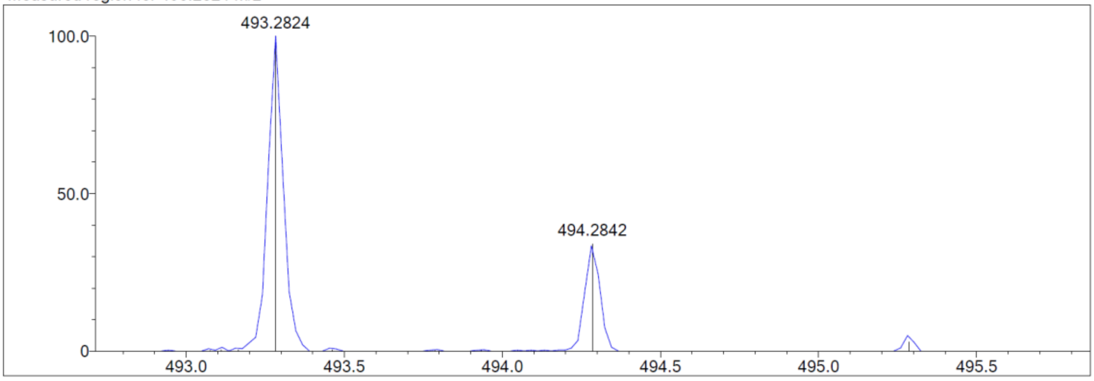

C28 H36 N4 O4 [M+H]+ : Predicted region for 493.2809 m/z

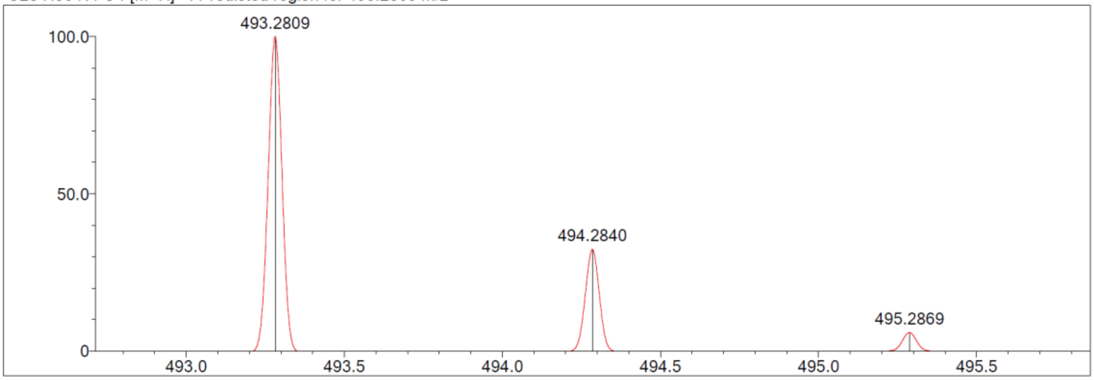

Rank	Score	Formula (M)	Ion	Meas. m/z	Pred. m/z	Df. (mDa)	Df. (ppm)	Iso	DBE
1	90.94	C28 H36 N4 O4	[M+H]+	493.2824	493.2809	1.5	3.04	95.82	13.0

Figure S148. Compound **D24** HRMS report.

*2-(4-(3-(Dimethylamino)propyl)piperazine-1-yl)-N-(4-((5-methoxy-1-oxo-2,3-dihydro-1H-inden-2-ylidene)methyl)phenyl)acetamide (**D25**)*

Dark brown powder. M.P.: 129.3 °C. Yield: 79%.

IR (ATR) ν_{max} (cm^{-1}): 3381 (N-H), 1681 (indanone C=O), 1600 (amide C=O), 1519-1458 (C=C), 1163 (C-N), 1087 (C-O), 827 (1,4-disubstituted benzene).

^{1}H-NMR (300 MHz, DMSO-d_6) δ (ppm): 1.52 (2H, p, *J*=7.15 Hz, CH_2), 2.09 (6H, s, CH_3), 2.18 (2H, t, *J*=7.14 Hz, CH_2), 2.27 (2H, t, *J*=7.43 Hz, CH_2), 2.42 (8H, bs, CH_2, piperazine CH_2), 3.14 (2H, m, CH_2), 3.89 (3H, s, OCH_3), 4.04 (2H, s, CH_2), 7.02 (1H, dd, J_1=8.46 Hz, J_2=2.19 Hz, methoxy-1-oxo-indenylidene CH), 7.17 (1H, *J*=1.80 Hz, methoxy-1-oxo-indenylidene CH), 7.40 (1H, s, C=CH), 7.70-7.72 (3H, m, disubstituted benzene CH, methoxy-1-oxo-indenylidene CH), 7.79 (2H, d, *J*=8.70 Hz, disubstituted benzene CH), 9.97 (1H, s, NH).

^{13}C-NMR (75 MHz, DMSO-d_6) δ (ppm): 25.0, 32.5, 45.7, 53.1, 53.3, 56.2, 56.5, 57.8, 62.4, 110.6, 115.8, 119.9, 125.8, 130.5, 131.2, 131.7, 131.9, 134.5, 140.4, 153.3, 165.3, 169.1, 192.0.

HRMS (ESI) (m/z) $[M+H]^+$: $C_{28}H_{36}N_4O_3$ calculated: 477.2860, found: 477.2855.

DOPNALAB

	Item	Value
	Acquired Date&Time	22.08.2019 12:54:39
	Acquired by	System Administrator
	Filename	C:\Users\dopnalab\Desktop\NURPELİN\DOKTORA TEZ\D251.ispd
	Spectrum name	D251
	Sample name	D25
	Sample ID	
	Option	
	Comment	
	No. of Scans	50
	Resolution	4 [cm-1]
	Apodization	Happ-Genzel

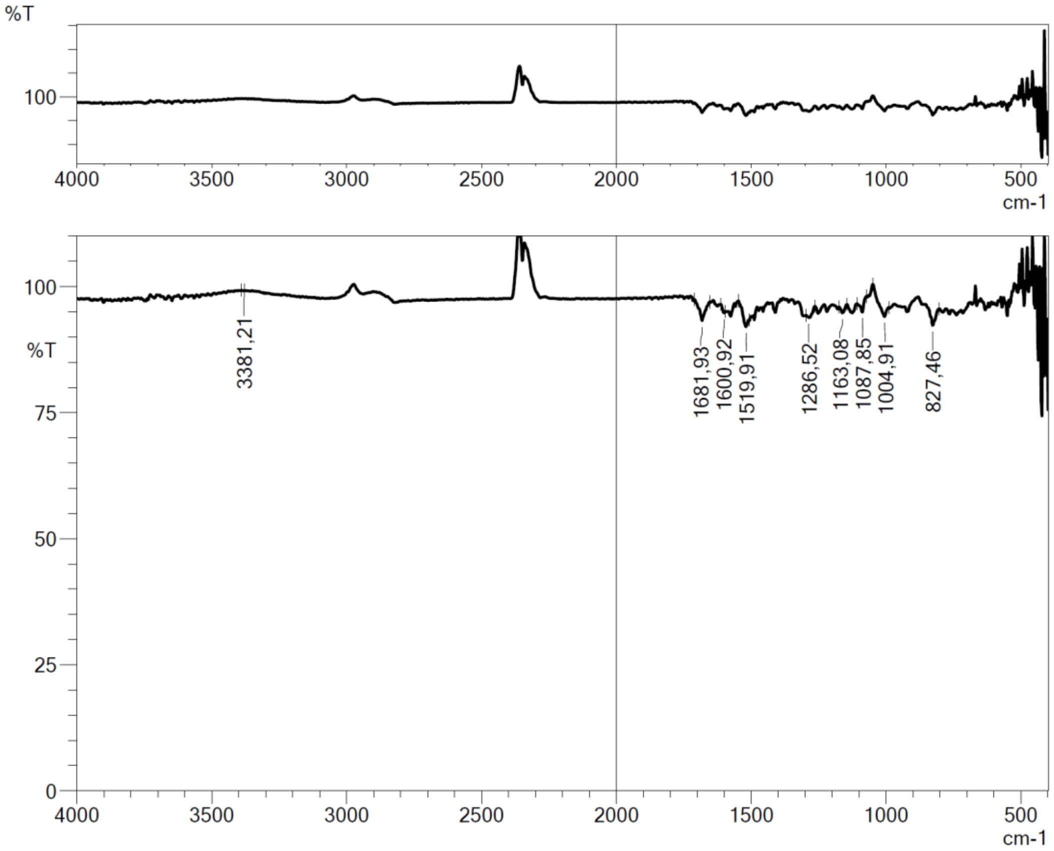

Figure S149. Compound **D25** IR report.

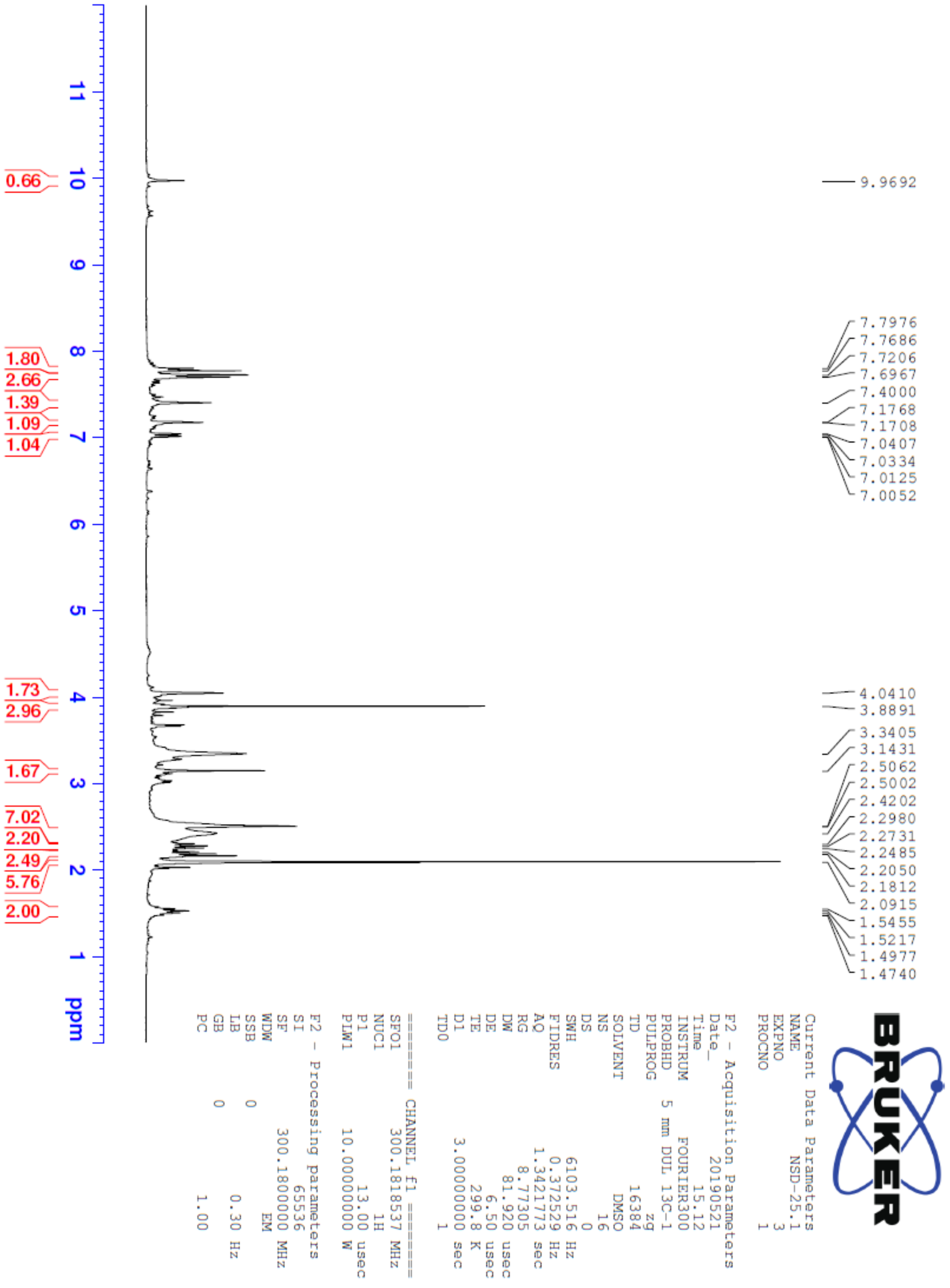

Figure S150. Compound **D25** ^{1}H-NMR spectrum.

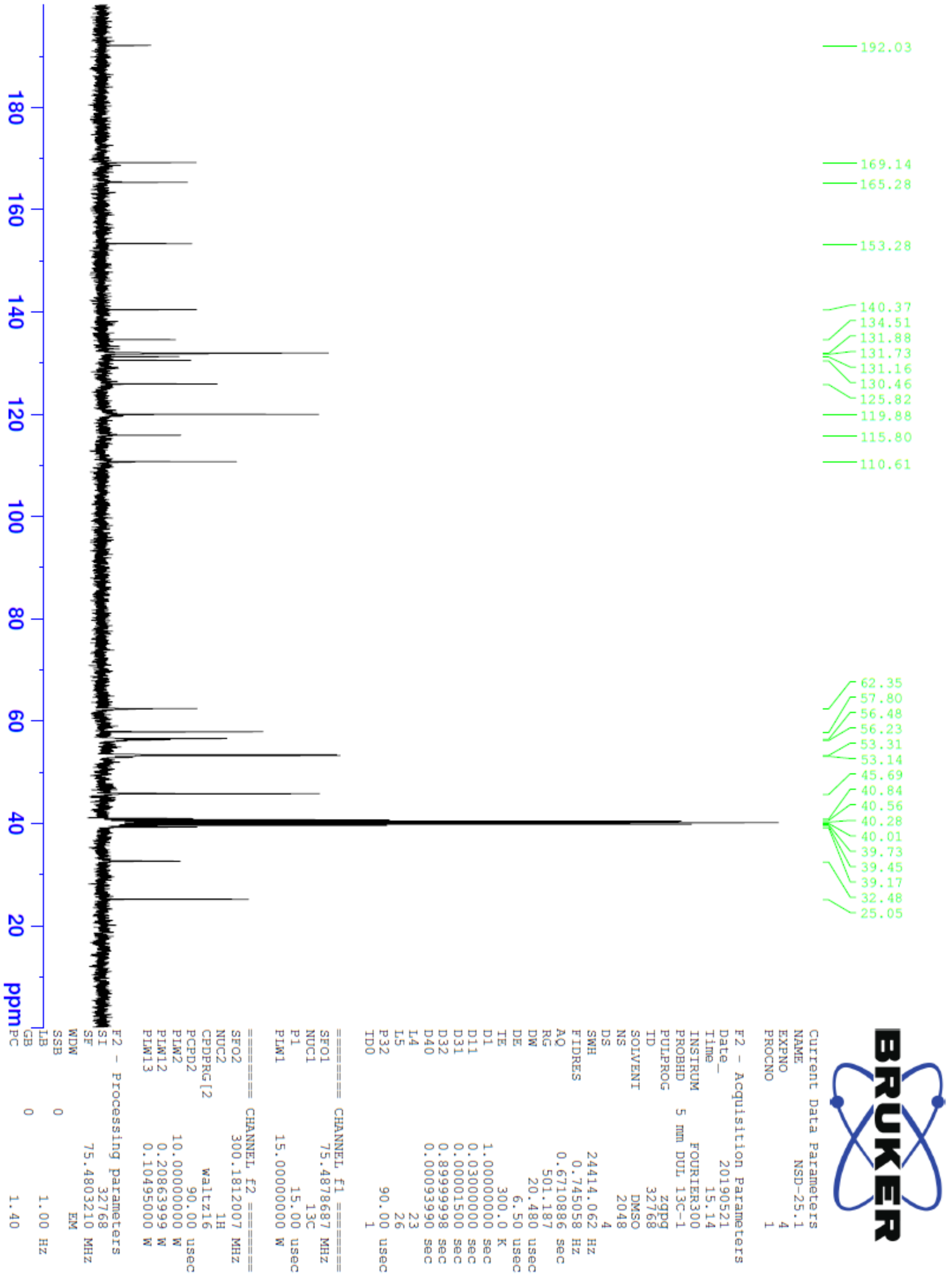

Figure S151. Compound **D25** ^{13}C-NMR spectrum.

Formula Predictor Report - NSD-25_5.lcd   Page 1 of 1

Data File: C:\LabSolutions\Data\Analiz\bns\NSD-25_5.lcd

Elmt	Val.	Min	Max	Elmt	Val.	Min	Max	Elmt	Val.	Min	Max	Elmt	Val.	Min	Max	Use Adduct
H	1	5	40	O	2	3	5	S	2	0	0	Ru	2	0	0	H
C	4	0	35	F	1	0	0	Cl	1	0	0	I	3	0	0	
N	3	3	6	P	3	0	0	Br	1	0	0					

Error Margin (ppm): 5
HC Ratio: unlimited
Max Isotopes: 3
MSn Iso RI (%): 10.00

DBE Range: 10.0 - 17.0
Apply N Rule: yes
Isotope RI (%): 1.00
MSn Logic Mode: AND

Electron Ions: both
Use MSn Info: yes
Isotope Res: 9000
Max Results: 500

Event#: 1 MS(E+) Ret. Time : 4.587 -> 4.707 Scan# : 689 -> 707

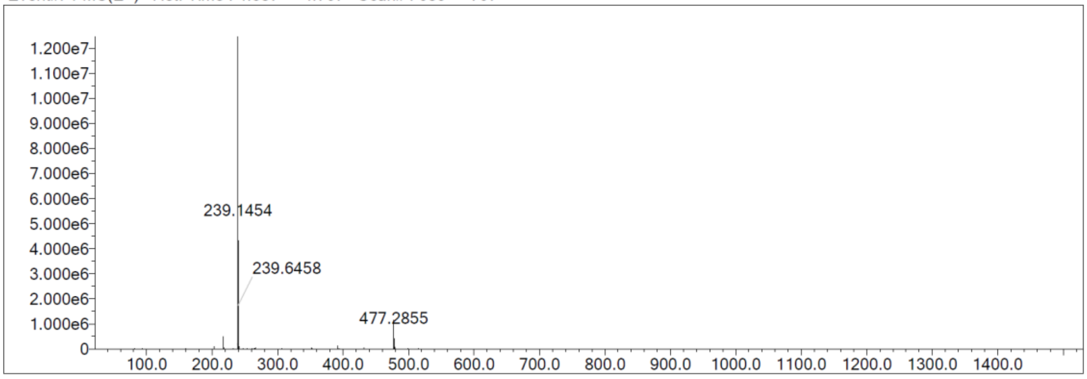

Measured region for 477.2855 m/z

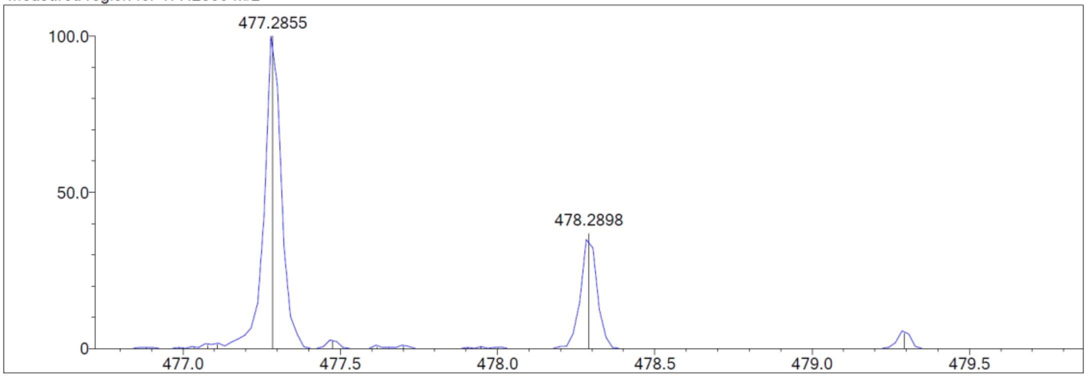

C28 H36 N4 O3 [M+H]+ : Predicted region for 477.2860 m/z

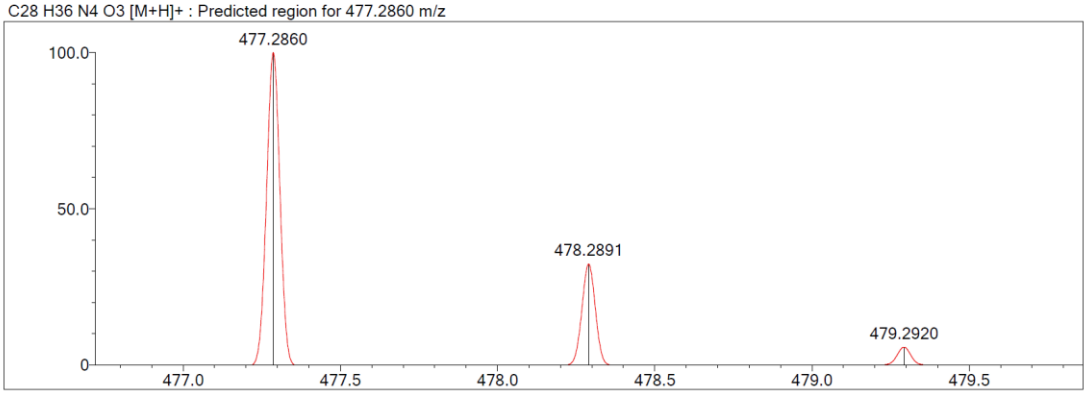

Rank	Score	Formula (M)	Ion	Meas. m/z	Pred. m/z	Df. (mDa)	Df. (ppm)	Iso	DBE
1	91.05	C28 H36 N4 O3	[M+H]+	477.2855	477.2860	-0.5	-1.05	91.16	13.0

Figure S152. Compound **D25** HRMS report.

*2-(4-(3-(Dimethylamino)propyl)piperazine-1-yl)-N-(4-((6-methoxy-1-oxo-2,3-dihydro-1H-inden-2-ylidene)methyl)phenyl)acetamide (**D26**)*

Dark brown powder. M.P.: 118.7 °C. Yield: 75%.

IR (ATR) ν_{max} (cm^{-1}): 3331 (N-H), 1674 (indanone C=O), 1593 (amide C=O), 1517-1489 (C=C), 1161 (C-N), 1020 (C-O), 827 (1,4-disubstituted benzene).

^{1}H-NMR (300 MHz, DMSO-d_6) δ (ppm): 1.52 (2H, p, *J*=7.26 Hz, CH_2), 2.09 (6H, s, CH_3), 2.18 (2H, t, *J*=6.84 Hz, CH_2), 2.27 (2H, t, *J*=7.40 Hz, CH_2), 2.41 (8H, bs, piperazine CH_2), 3.14 (2H, m, CH_2), 3.83 (3H, s, OCH_3), 4.01 (2H, s, CH_2), 7.24 (1H, d, *J*=2.43 Hz, methoxy-1-oxo-indenylidene CH), 7.28 (1H, dd, J_1=8.37 Hz, J_2=2.43 Hz, methoxy-1-oxo-indenylidene CH), 7.48 (1H, s, C=CH), 7.57 (1H, *J*=8.38 Hz, methoxy-1-oxo-indenylidene CH), 7.73 (2H, d, *J*=9.00 Hz, disubstituted benzene CH), 7.79 (2H, d, *J*=8.89 Hz, disubstituted benzene CH), 9.99 (1H, s, NH).

^{13}C-NMR (75 MHz, DMSO-d_6) δ (ppm): 25.0, 31.7, 45.7, 53.1, 53.3, 56.0, 56.5, 57.8, 62.4, 106.0, 119.9, 123.7, 127.9, 130.3, 132.1, 133.0, 134.7, 139.1, 140.6, 142.9, 159.6, 169.2, 193.6.

HRMS (ESI) (m/z) $[M+H]^+$: $C_{28}H_{36}N_4O_3$ calculated: 477.2860, found: 477.2865.

DOPNALAB

	Item	Value
	Acquired Date&Time	22.08.2019 12:59:11
	Acquired by	System Administrator
	Filename	C:\Users\dopnalab\Desktop\NURPELİN\DOKTORA TEZ\D261.ispd
	Spectrum name	D261
	Sample name	D26
	Sample ID	
	Option	
	Comment	
	No. of Scans	50
	Resolution	4 [cm-1]
	Apodization	Happ-Genzel

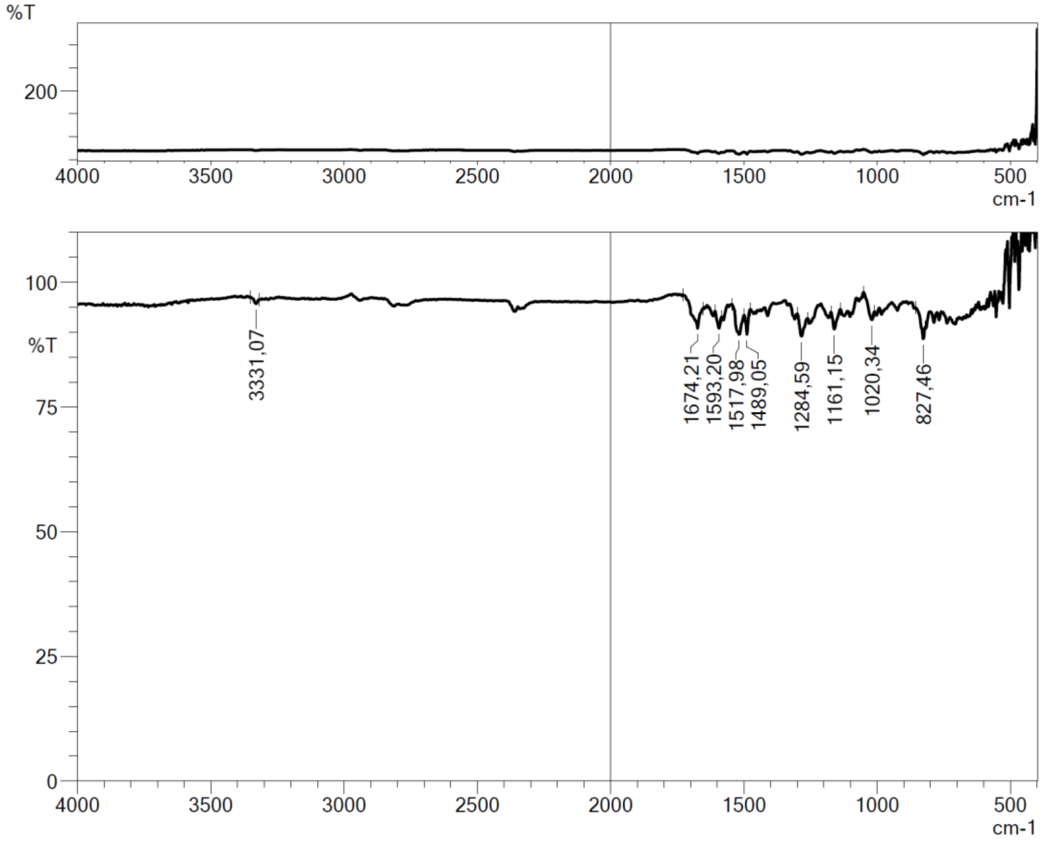

Figure S153. Compound **D26** IR report.

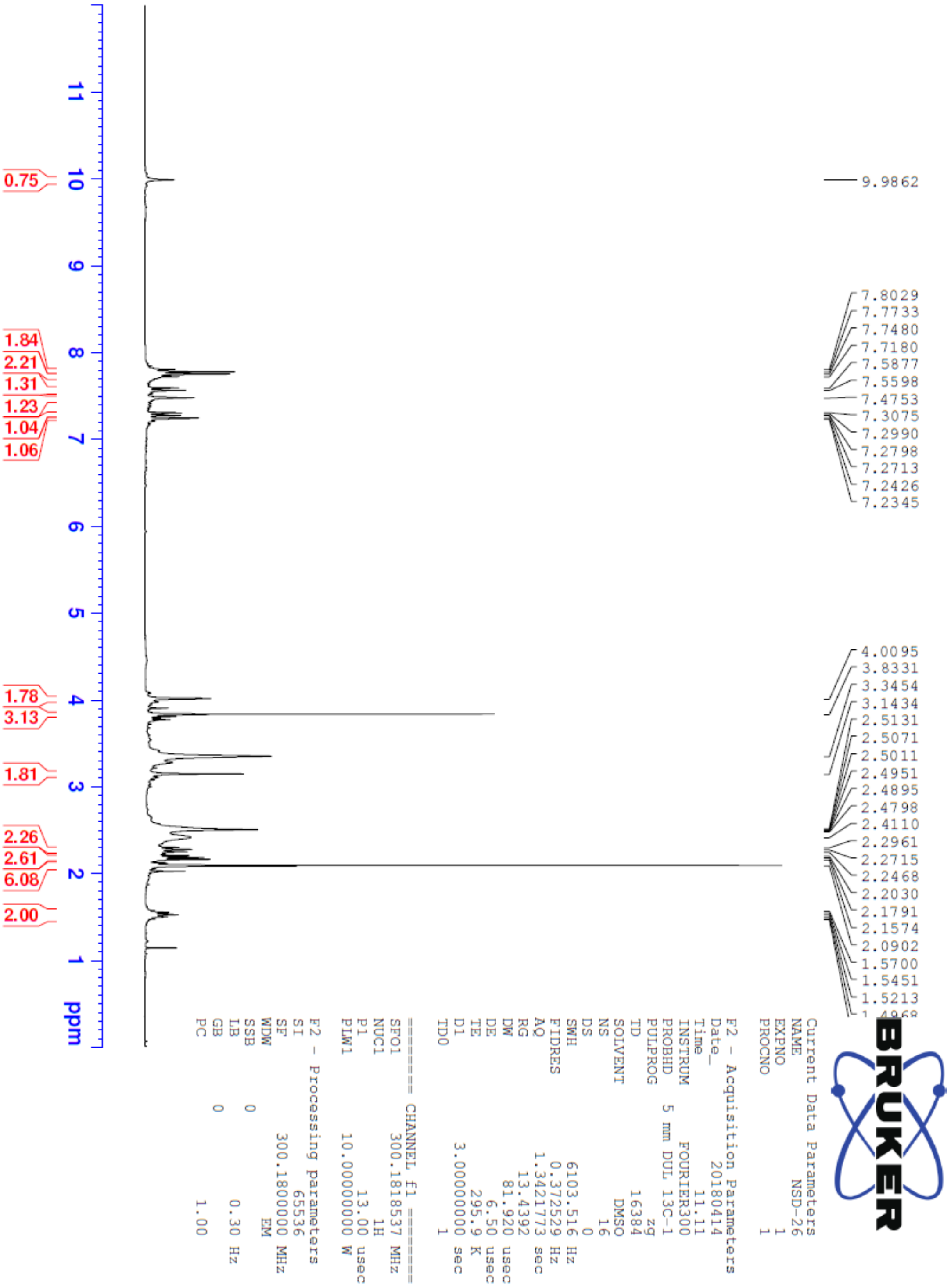

Figure S154. Compound **D26** ^{1}H-NMR spectrum.

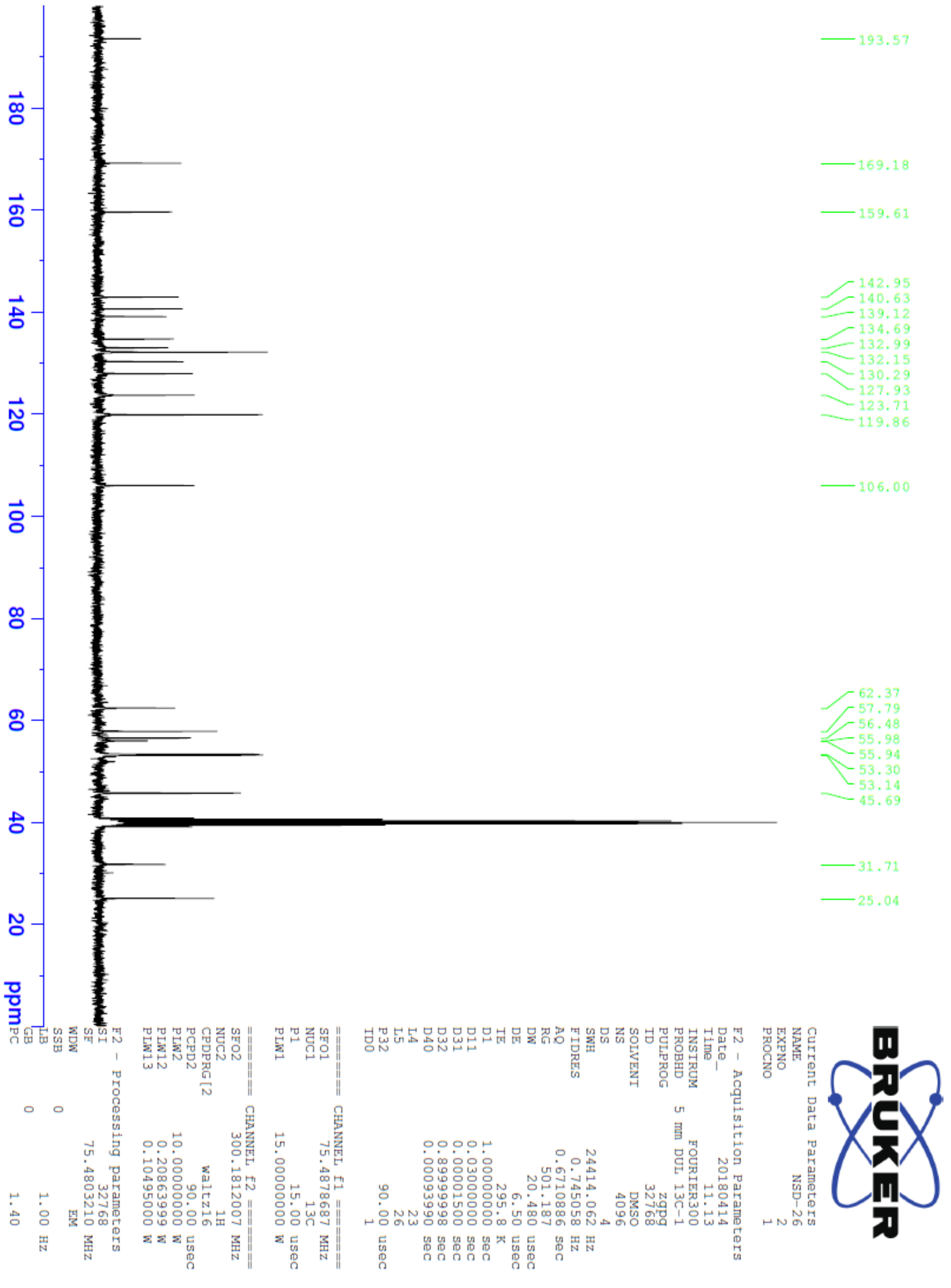

Figure S155. Compound **D26** ^{13}C-NMR spectrum.

Data File: C:\LabSolutions\Data\Analiz\Serkan\NSD-26_66.lcd

Elmt	Val.	Min	Max	Elmt	Val.	Min	Max	Elmt	Val.	Min	Max	Elmt	Val.	Min	Max	Use Adduct
H	1	5	40	O	2	3	5	S	2	0	0	Ru	2	0	0	H
C	4	0	35	F	1	0	0	Cl	1	0	0	I	3	0	0	
N	3	3	6	P	3	0	0	Br	1	0	0					

Error Margin (ppm):	5	DBE Range:	10.0 - 17.0	Electron Ions:	both
HC Ratio:	unlimited	Apply N Rule:	yes	Use MSn Info:	yes
Max Isotopes:	3	Isotope RI (%):	1.00	Isotope Res:	9000
MSn Iso RI (%):	10.00	MSn Logic Mode:	AND	Max Results:	500

Event#: 1 MS(E+) Ret. Time : 1.587 -> 1.707 Scan# : 239 -> 257

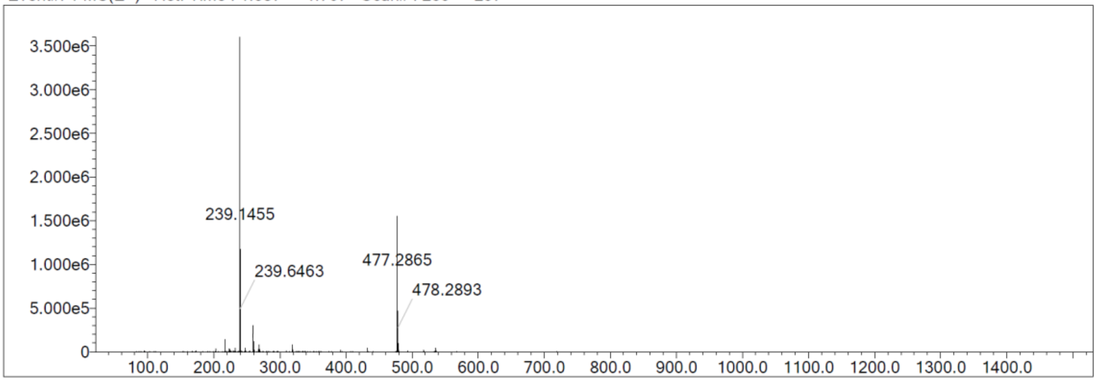

Measured region for 477.2865 m/z

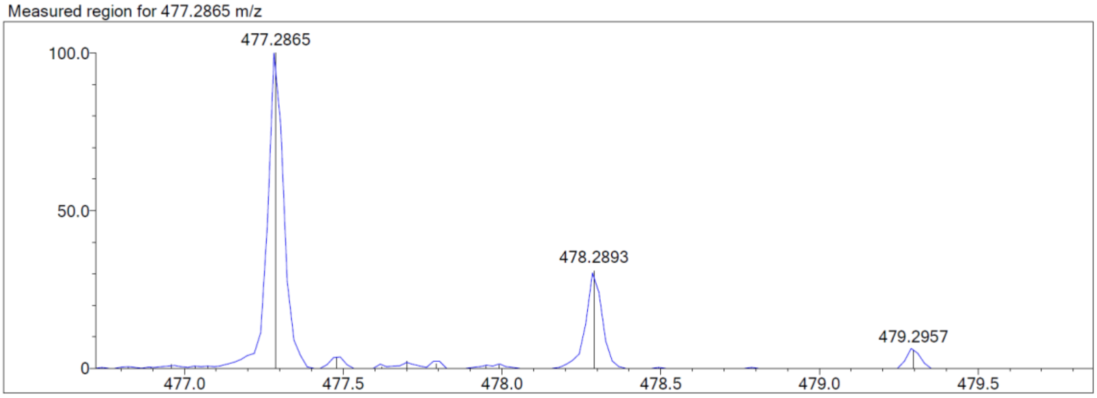

C28 H36 N4 O3 [M+H]+ : Predicted region for 477.2860 m/z

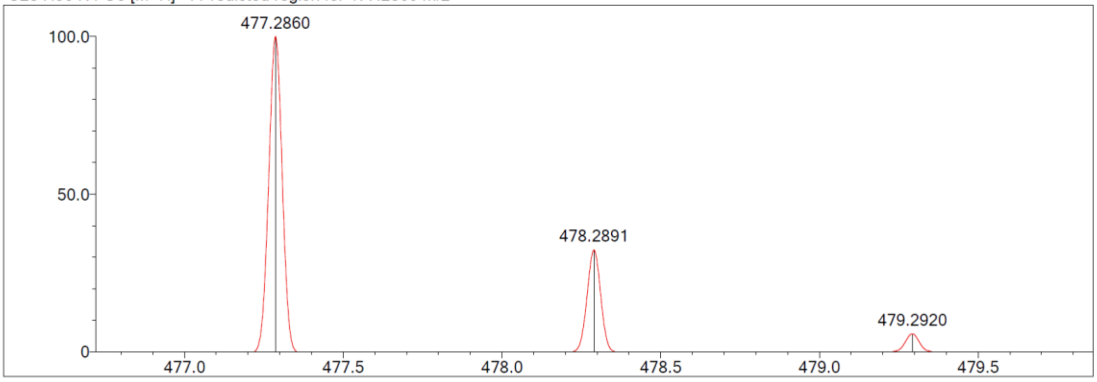

Rank	Score	Formula (M)	Ion	Meas. m/z	Pred. m/z	Df. (mDa)	Df. (ppm)	Iso	DBE
1	89.49	C28 H36 N4 O3	[M+H]+	477.2865	477.2860	0.5	1.05	89.60	13.0

Figure S156. Compound **D26** HRMS report.

*2-(4-(3-(Dimethylamino)propyl)piperazine-1-yl)-N-(4-((5,6-dimethoxy-1-oxo-2,3-dihydro-1H-inden-2-ylidene))methyl)phenyl)acetamide (**D27**)*

Brown powder. M.P.: 138.7 °C. Yield: 89%.

IR (ATR) ν_{max} (cm^{-1}): 3323 (N-H), 1643 (indanone C=O), 1616 (amide C=O), 1556-1456 (C=C), 1184 (C-N), 1020 (C-O), 856 (1,4-disubstituted benzene).

^{1}H-NMR (300 MHz, DMSO-d_6) δ (ppm): 1.53 (2H, p, *J*=7.28 Hz, CH_2), 2.09 (6H, s, CH_3), 2.18 (2H, t, *J*=7.26 Hz, CH_2), 2.28 (2H, t, *J*=7.40 Hz, CH_2), 2.42 (8H, bs, piperazine CH_2), 3.14 (2H, m, CH_2), 3.84 (3H, s, OCH_3), 3.89 (3H, s, OCH_3), 3.97 (2H, s, CH_2), 7.20 (1H, s, methoxy-1-oxo-indenylidene CH), 7.21 (1H, s, methoxy-1-oxo-indenylidene CH), 7.38 (1H, s, C=CH), 7.70 (2H, d, *J*=8.85 Hz, disubstituted benzene CH), 7.78 (2H, d, *J*=8.80 Hz, disubstituted benzene CH), 9.96 (1H, s, NH).

^{13}C-NMR (75 MHz, DMSO-d_6) δ (ppm): 25.0, 32.1, 45.7, 53.1, 53.3, 56.1, 56.4, 56.5, 57.8, 62.4, 104.8, 108.5, 119.9, 130.5, 130.5, 131.3, 131.8, 134.8, 140.3, 145.4, 149.7, 155.6, 169.1, 192.3.

HRMS (ESI) (m/z) $[M+H]^+$: $C_{29}H_{38}N_4O_4$ calculated: 507.2966, found: 507.2986.

DOPNALAB

	Item	Value
	Acquired Date&Time	22.08.2019 13:01:32
	Acquired by	System Administrator
	Filename	C:\Users\dopnalab\Desktop\NURPELİN\DOKTORA TEZ\D271.ispd
	Spectrum name	D271
	Sample name	D27
	Sample ID	
	Option	
	Comment	
	No. of Scans	50
	Resolution	4 [cm-1]
	Apodization	Happ-Genzel

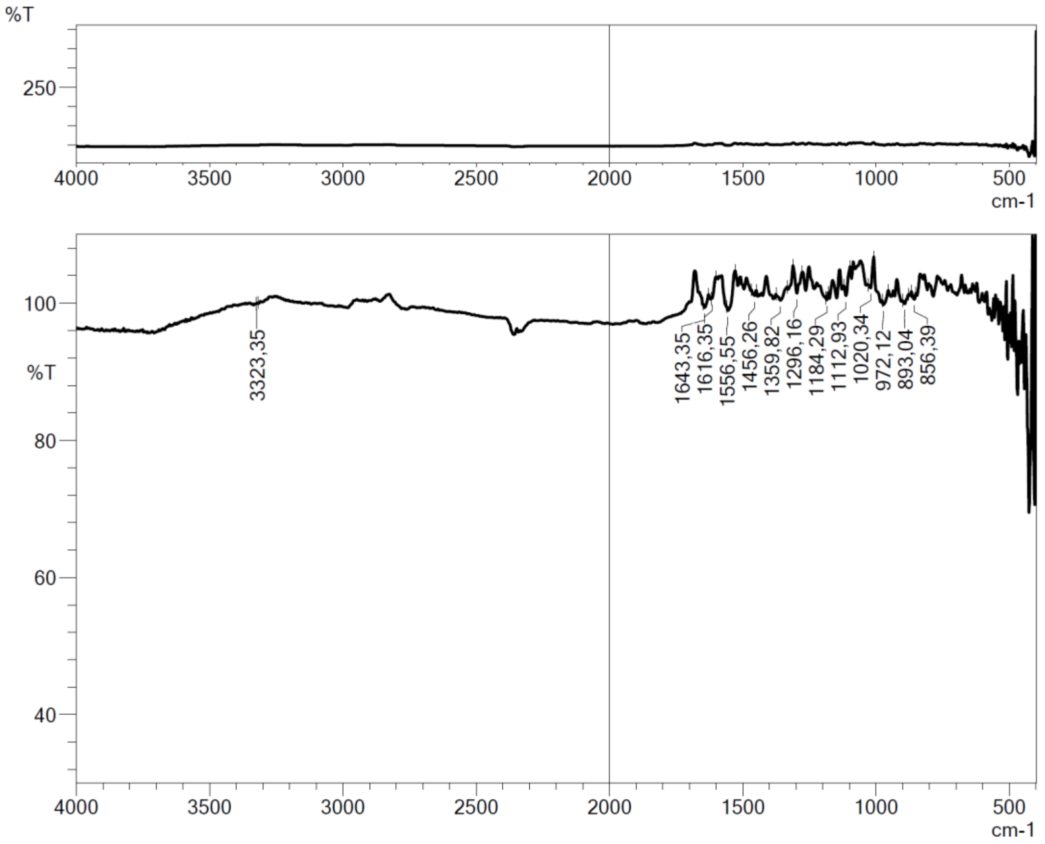

Figure S157. Compound **D27** IR report.

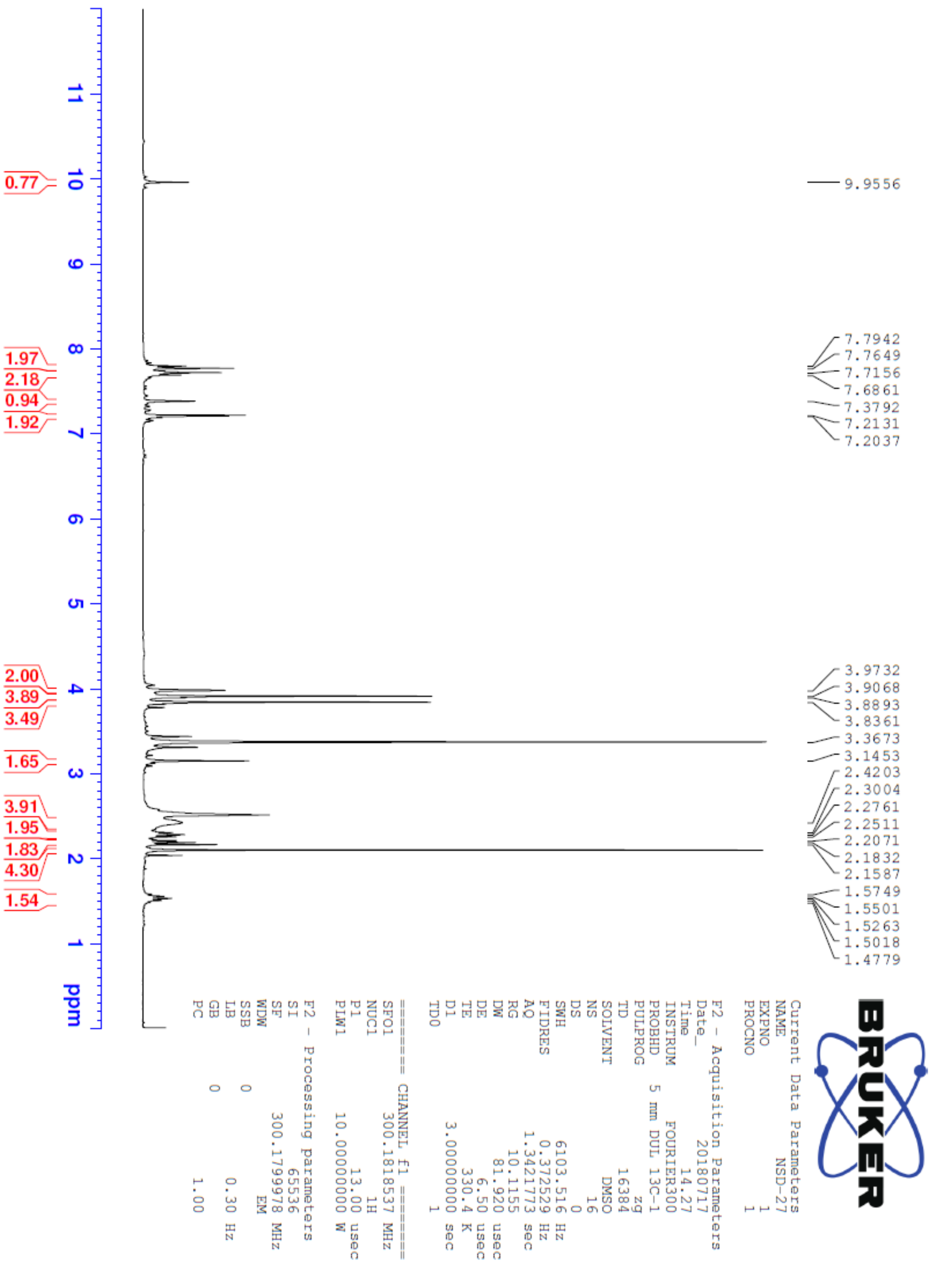

Figure S158. Compound **D27** ^{1}H-NMR spectrum.

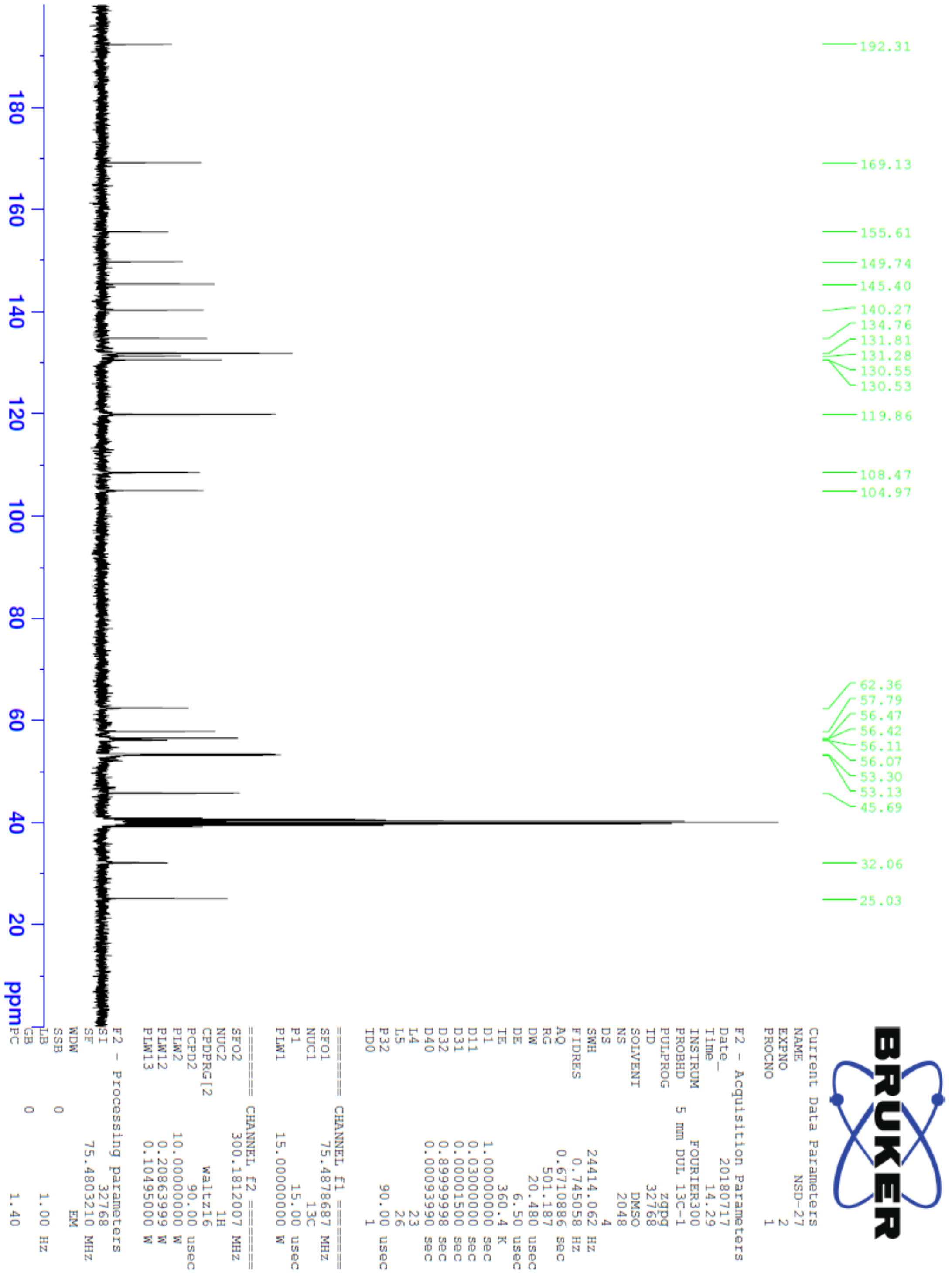

Figure S159. Compound **D27** ^{13}C-NMR spectrum.

Formula Predictor Report - NSD-27_6.lcd

Page 1 of 1

Data File: C:\LabSolutions\Data\Analiz\bns\NSD-27_6.lcd

Elmt	Val.	Min	Max	Elmt	Val.	Min	Max	Elmt	Val.	Min	Max	Elmt	Val.	Min	Max	Use Adduct
H	1	5	40	O	2	3	5	S	2	0	0	Ru	2	0	0	H
C	4	0	35	F	1	0	0	Cl	1	0	0	I	3	0	0	
N	3	3	6	P	3	0	0	Br	1	0	0					

Error Margin (ppm):	5	DBE Range:	10.0 - 17.0	Electron Ions:	both
HC Ratio:	unlimited	Apply N Rule:	yes	Use MSn Info:	yes
Max Isotopes:	3	Isotope RI (%):	1.00	Isotope Res:	9000
MSn Iso RI (%):	10.00	MSn Logic Mode:	AND	Max Results:	500

Event#: 1 MS(E+) Ret. Time : 4.413 -> 4.467 Scan# : 663 -> 671

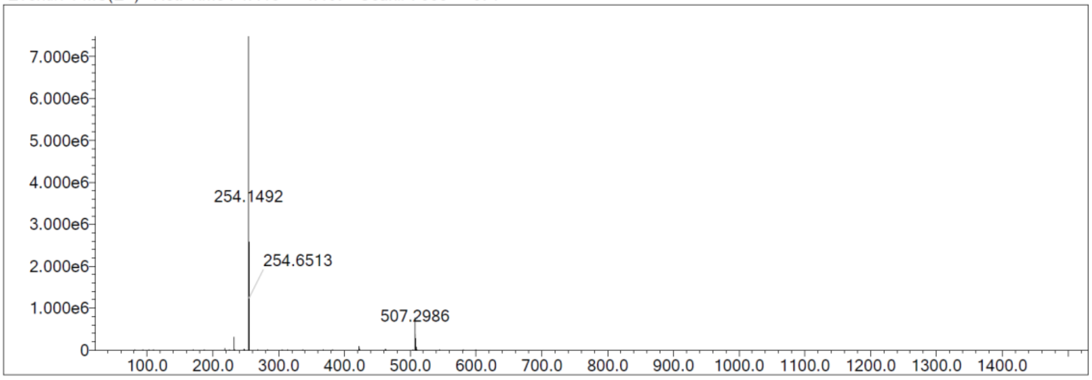

Measured region for 507.2986 m/z

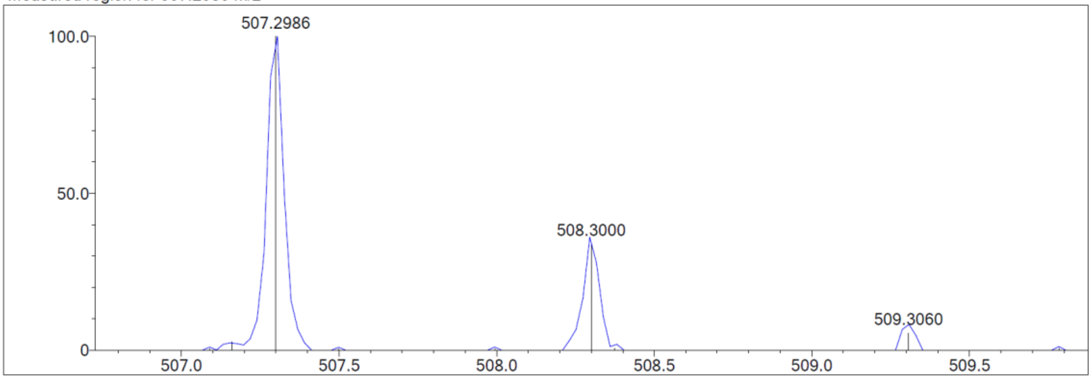

C29 H38 N4 O4 [M+H]+ : Predicted region for 507.2966 m/z

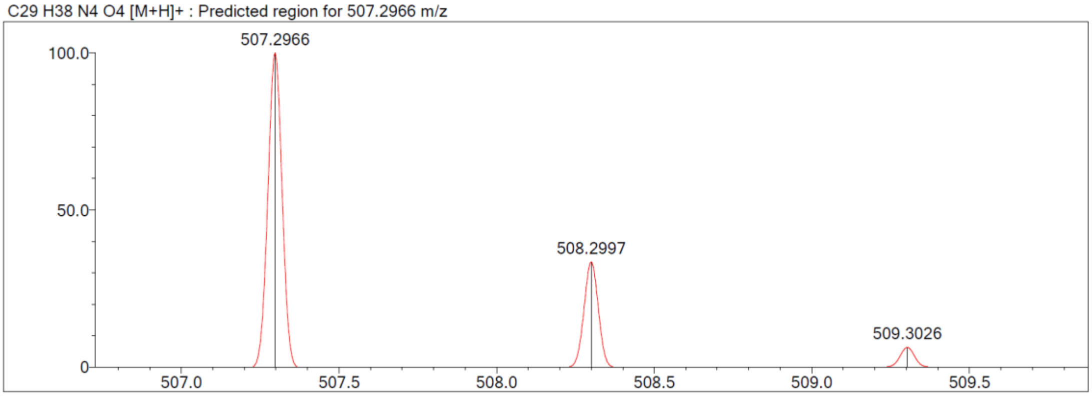

Rank	Score	Formula (M)	Ion	Meas. m/z	Pred. m/z	Df. (mDa)	Df. (ppm)	Iso	DBE
1	84.86	C29 H38 N4 O4	[M+H]+	507.2986	507.2966	2.0	3.94	91.59	13.0

Figure S160. Compound **D27** HRMS report.

*2-(N-methyl-N-propylamino)-N-(4-((5-methoxy-1-oxo-2,3-dihydro-1H-inden-2-ylidene) methyl)phenyl)acetamide (**D28**)*

Dark beige powder. M.P.: 155.6 °C. Yield: 82%.

IR (ATR) ν_{max} (cm^{-1}): 3284 (N-H), 1687 (indanone C=O), 1631 (amide C=O), 1506-1402 (C=C), 1141 (C-N), 1085 (C-O), 817 (1,4-disubstituted benzene).

^{1}H-NMR (300 MHz, DMSO-d_6) δ (ppm): 0.87 (2H, t, J=7.35 Hz, CH_3), 1.47 (2H, st, J=7.35 Hz, CH_2), 2.30 (3H, s, CH_3), 2.40 (2H, t, J=7.41 Hz, CH_2), 3.15 (2H, m, CH_2), 3.89 (3H, s, OCH_3), 4.04 (2H, s, CH_2), 7.02 (1H, dd, J_1=8.49 Hz, J_2=2.25 Hz, methoxy-1-oxo-indenylidene CH), 7.17 (1H, J=1.98 Hz, methoxy-1-oxo-indenylidene CH), 7.40 (1H, s, C=CH), 7.70-7.72 (3H, m, disubstituted benzene CH, methoxy-1-oxo-indenylidene CH), 7.79 (2H, d, J=8.76 Hz, disubstituted benzene CH), 9.89 (1H, s, NH).

^{13}C-NMR (75 MHz, DMSO-d_6) δ (ppm): 12.2, 20.4, 32.5, 42.9, 56.3, 59.6, 62.0, 110.6, 115.8, 119.8, 125.8, 130.4, 131.1, 131.7, 131.9, 134.5, 140.3, 153.3, 165.3, 169.9, 192.0.

HRMS (ESI) (m/z) $[M+H]^+$: $C_{23}H_{26}N_2O_3$ calculated: 379.2016, found: 379.2010.

DOPNALAB

	Item	Value
	Acquired Date&Time	22.08.2019 13:04:41
	Acquired by	System Administrator
	Filename	C:\Users\dopnalab\Desktop\NURPELİN\DOKTORA TEZ\D281.ispd
	Spectrum name	D281
	Sample name	D28
	Sample ID	
	Option	
	Comment	
	No. of Scans	50
	Resolution	4 [cm-1]
	Apodization	Happ-Genzel

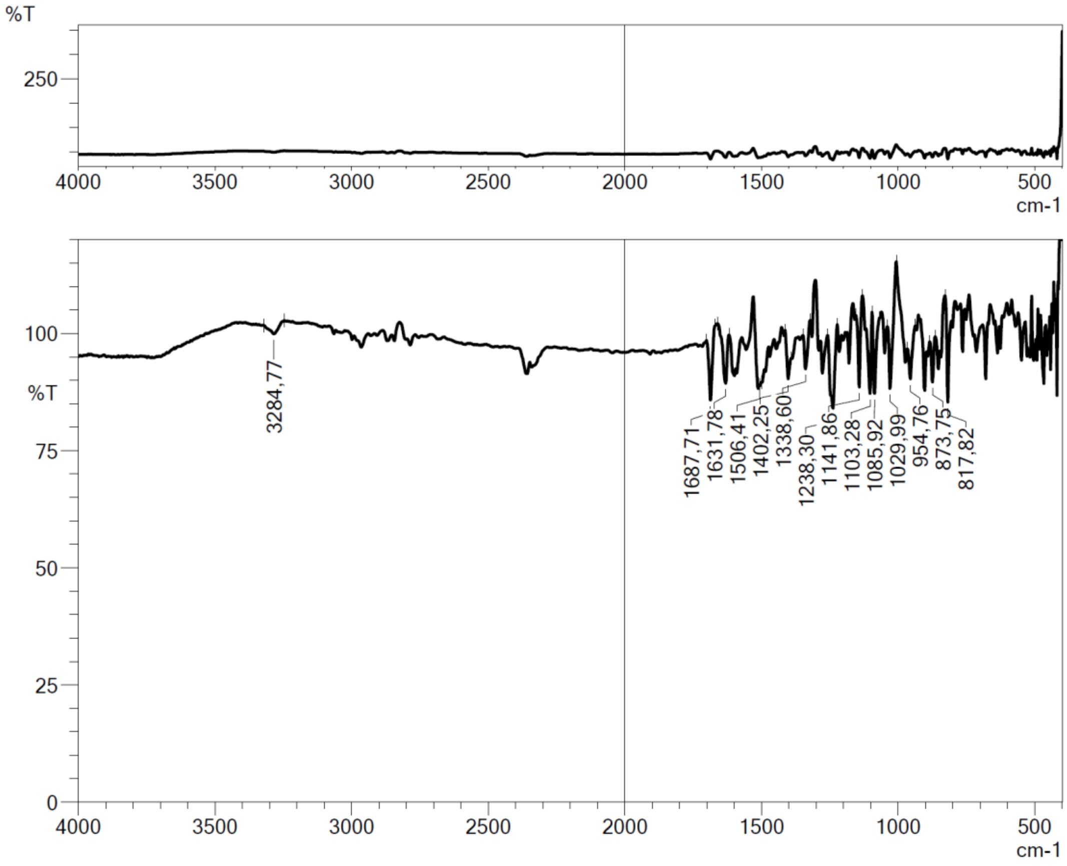

Figure S161. Compound **D28** IR report.

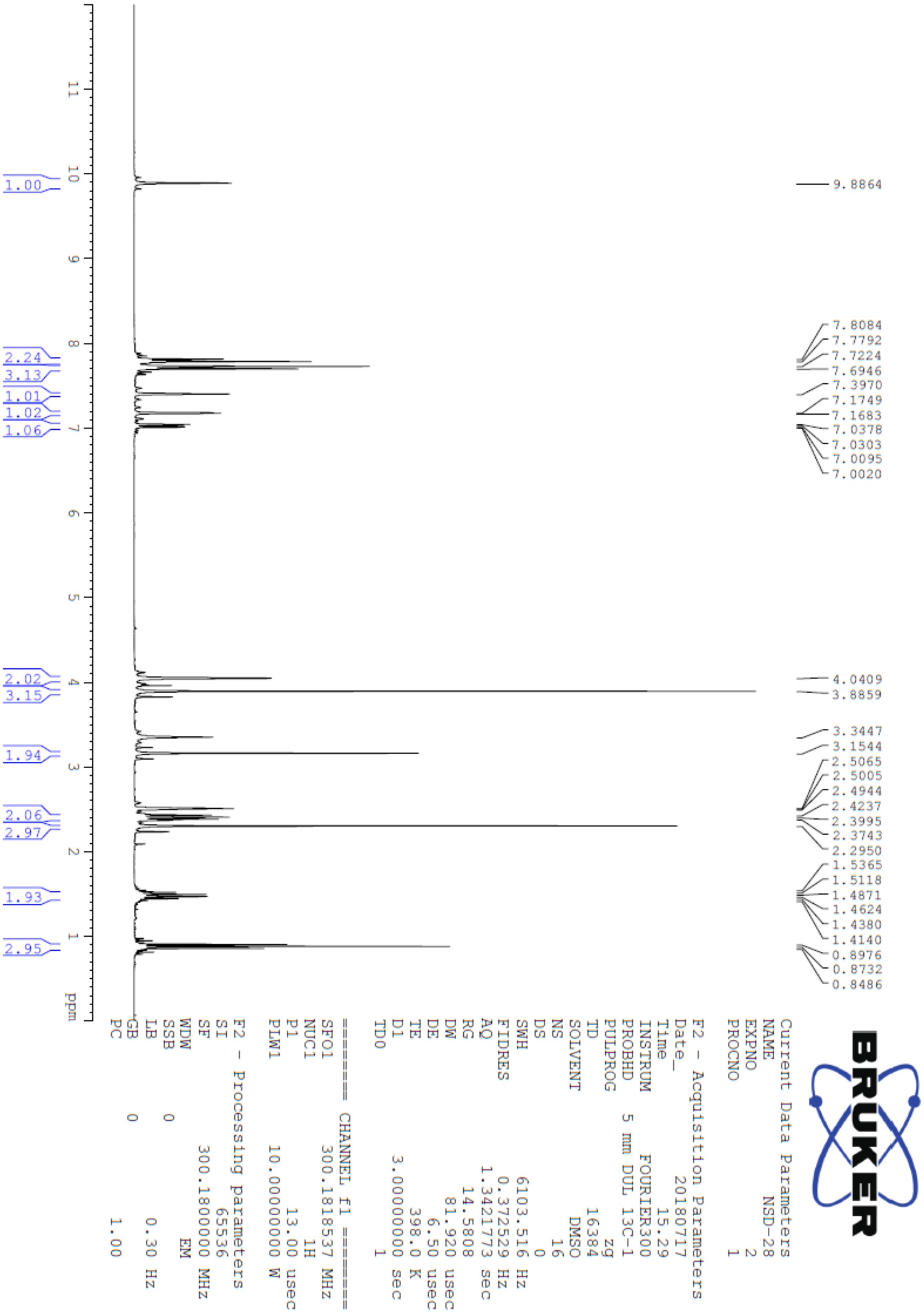

Figure S162. Compound **D28** ^{1}H-NMR spectrum.

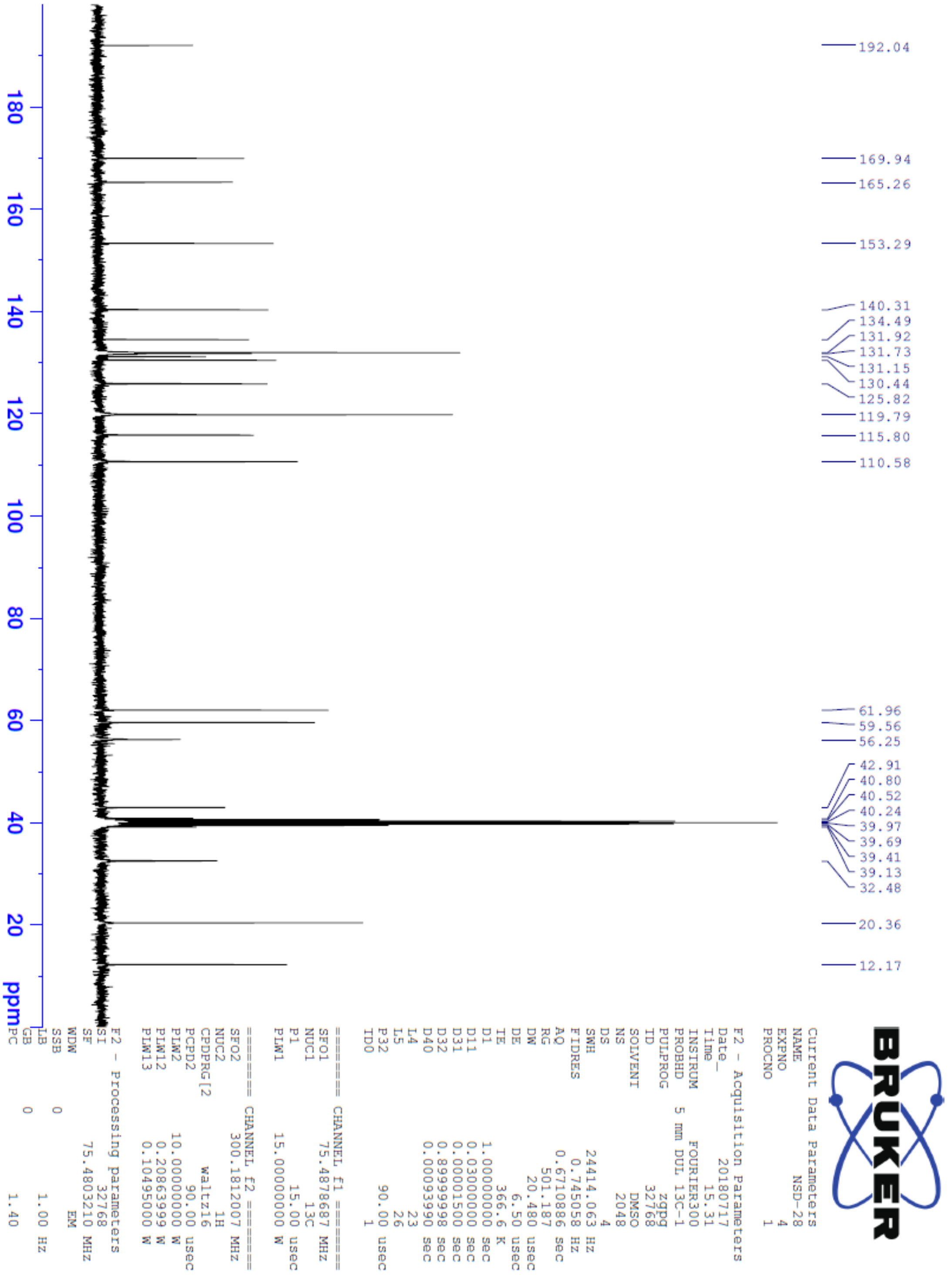

Figure S163. Compound **D28** ^{13}C-NMR spectrum.

Data File: C:\LabSolutions\Data\Analiz\bns\NSD-28_7.lcd

Elmt	Val.	Min	Max	Elmt	Val.	Min	Max	Elmt	Val.	Min	Max	Elmt	Val.	Min	Max	Use Adduct
H	1	5	40	O	2	3	5	S	2	0	0	Ru	2	0	0	H
C	4	0	35	F	1	0	0	Cl	1	0	0	I	3	0	0	
N	3	2	6	P	3	0	0	Br	1	0	0					

Error Margin (ppm): 10
HC Ratio: unlimited
Max Isotopes: 3
MSn Iso RI (%): 10.00

DBE Range: 10.0 - 17.0
Apply N Rule: yes
Isotope RI (%): 1.00
MSn Logic Mode: AND

Electron Ions: both
Use MSn Info: yes
Isotope Res: 9000
Max Results: 500

Event#: 1 MS(E+) Ret. Time : 5.453 -> 5.573 Scan# : 819 -> 837

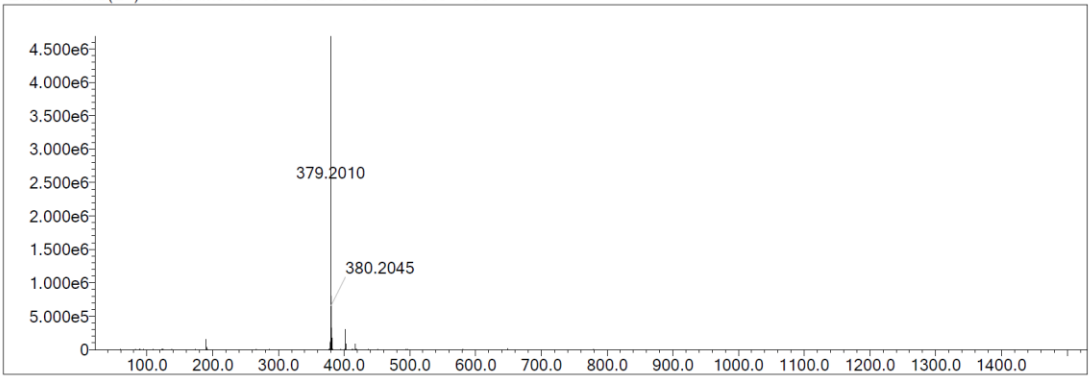

Measured region for 379.2010 m/z

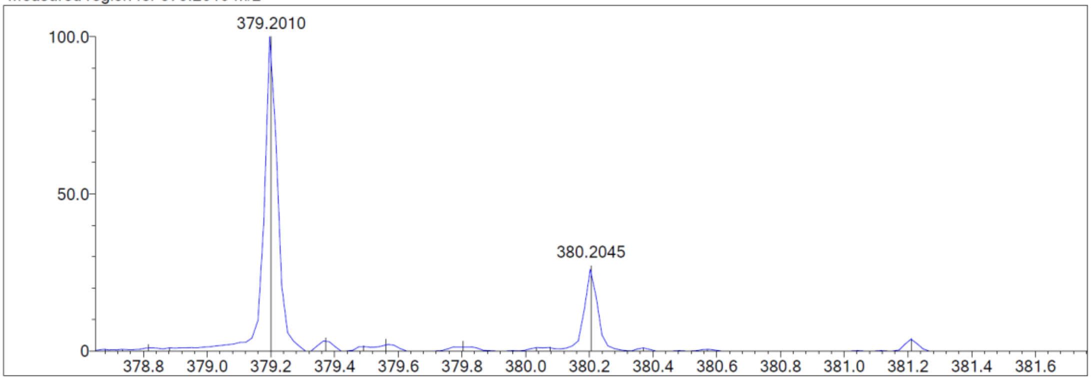

C23 H26 N2 O3 [M+H]+ : Predicted region for 379.2016 m/z

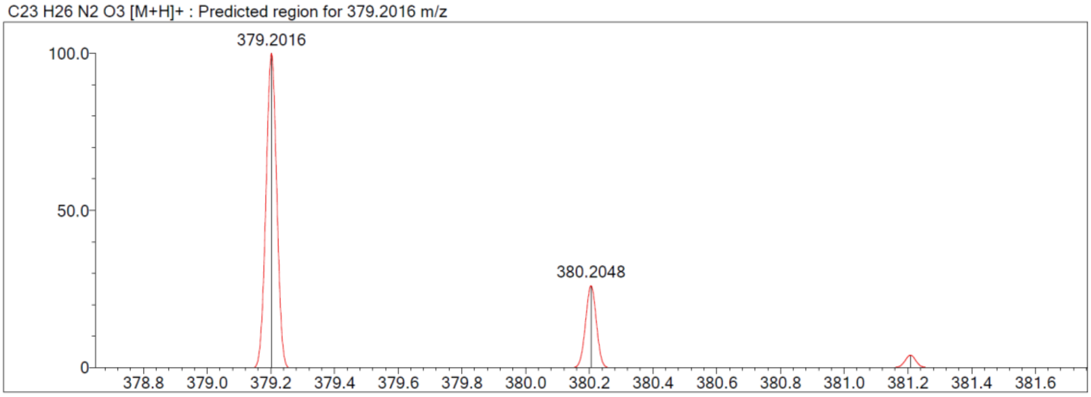

Rank	Score	Formula (M)	Ion	Meas. m/z	Pred. m/z	Df. (mDa)	Df. (ppm)	Iso	DBE
1	81.56	C23 H26 N2 O3	[M+H]+	379.2010	379.2016	-0.6	-1.58	82.76	12.0

Figure S164. Compound **D28** HRMS report.

*2-(N-methyl-N-propylamino)-N-(4-((6-methoxy-1-oxo-2,3-dihydro-1H-inden-2-ylidene) methyl)phenyl)acetamide (**D29**)*

Brown powder. M.P.: 95.1 °C. Yield: 78%.

IR (ATR) ν_{max} (cm^{-1}): 3296 (N-H), 1687 (indanone C=O), 1630 (amide C=O), 1517-1487 (C=C), 1184 (C-N), 1026 (C-O), 835 (1,4-disubstituted benzene).

^{1}H-NMR (300 MHz, DMSO-d_6) δ (ppm): 0.88 (3H, t, J=7.35 Hz, CH_3), 1.46 (2H, st, J=7.34 Hz, CH_2), 2.30 (3H, s, CH_3), 2.40 (2H, t, J=7.43 Hz, CH_2), 3.16 (2H, m, CH_2), 3.83 (3H, s, OCH_3), 4.01 (2H, s, CH_2), 7.24 (1H, J=2.46 Hz, methoxy-1-oxo-indenylidene CH), 7.29 (1H, dd, J_1=8.31 Hz, J_2=2.55 Hz, methoxy-1-oxo-indenylidene CH), 7.48 (1H, s, C=CH), 7.57 (1H, d, J=8.38 Hz, methoxy-1-oxo-indenylidene CH), 7.74 (2H, d, J=8.98 Hz, disubstituted benzene CH), 7.80 (2H, d, J=8.86 Hz, disubstituted benzene CH), 9.90 (1H, s, NH).

^{13}C-NMR (75 MHz, DMSO-d_6) δ (ppm): 12.8, 20.4, 31.7, 42.9, 56.0, 59.6, 62.0, 106.0, 119.8, 123.7, 127.9, 130.3, 132.2, 133.0, 134.7, 139.1, 140.6, 143.0, 159.6, 170.0, 193.6.

HRMS (ESI) (m/z) $[M+H]^+$: $C_{23}H_{26}N_2O_3$ calculated: 379.2016, found: 379.2023.

DOPNALAB

	Item	Value
	Acquired Date&Time	22.08.2019 13:07:19
	Acquired by	System Administrator
	Filename	C:\Users\dopnalab\Desktop\NURPELİN\DOKTORA TEZ\D291.ispd
	Spectrum name	D291
	Sample name	D29
	Sample ID	
	Option	
	Comment	
	No. of Scans	50
	Resolution	4 [cm-1]
	Apodization	Happ-Genzel

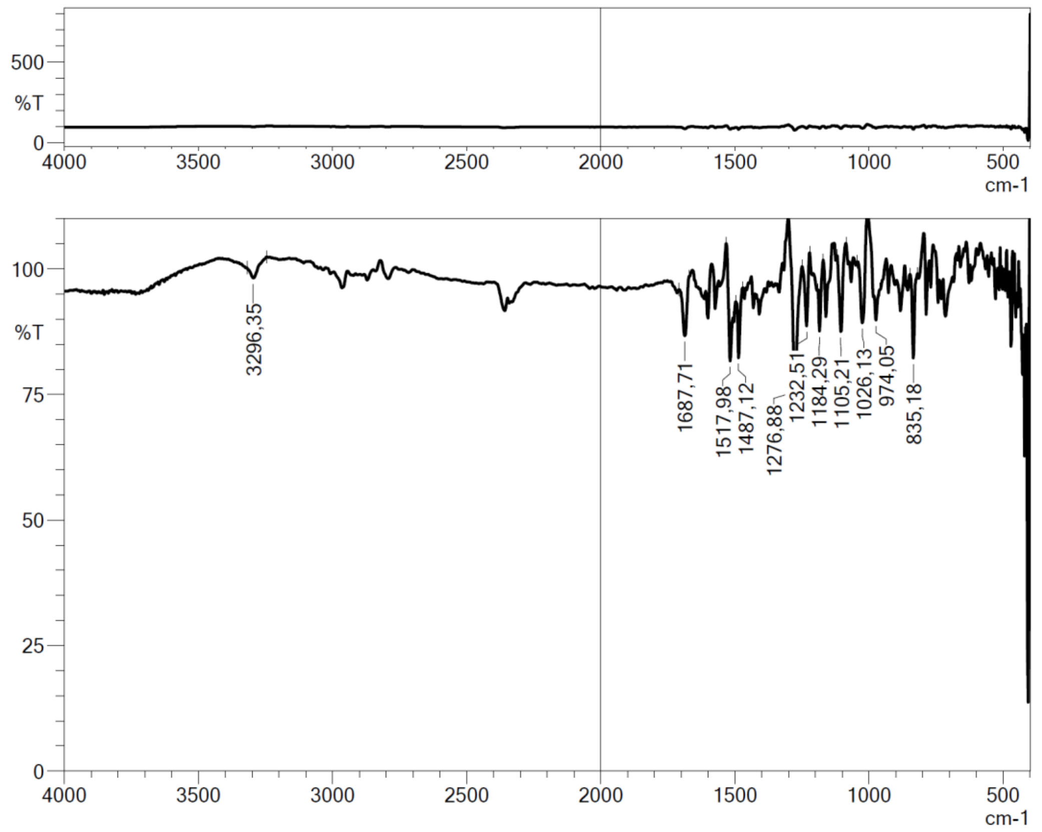

Figure S165. Compound **D29** IR report.

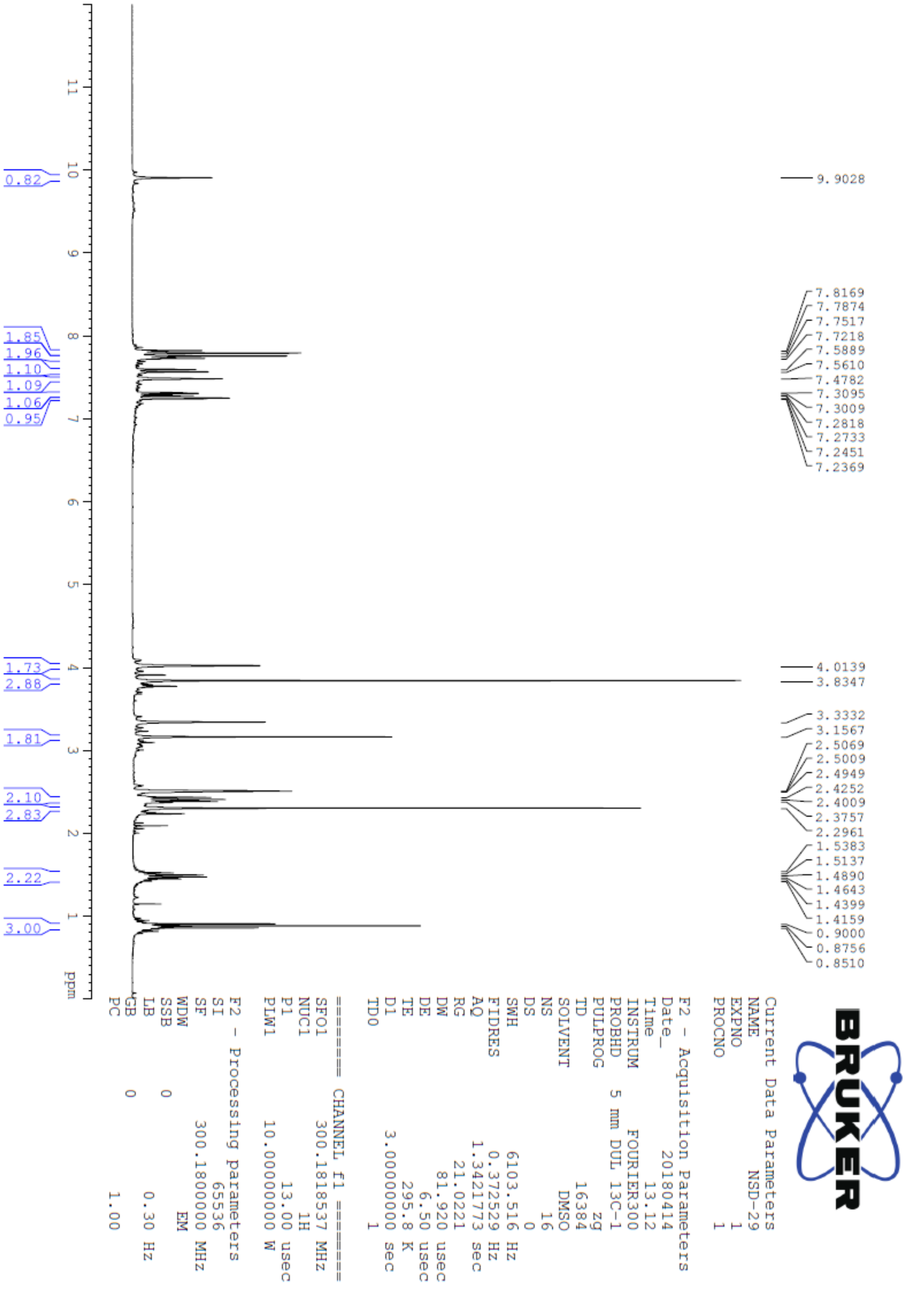

Figure S166. Compound **D29** ^{1}H-NMR spectrum.

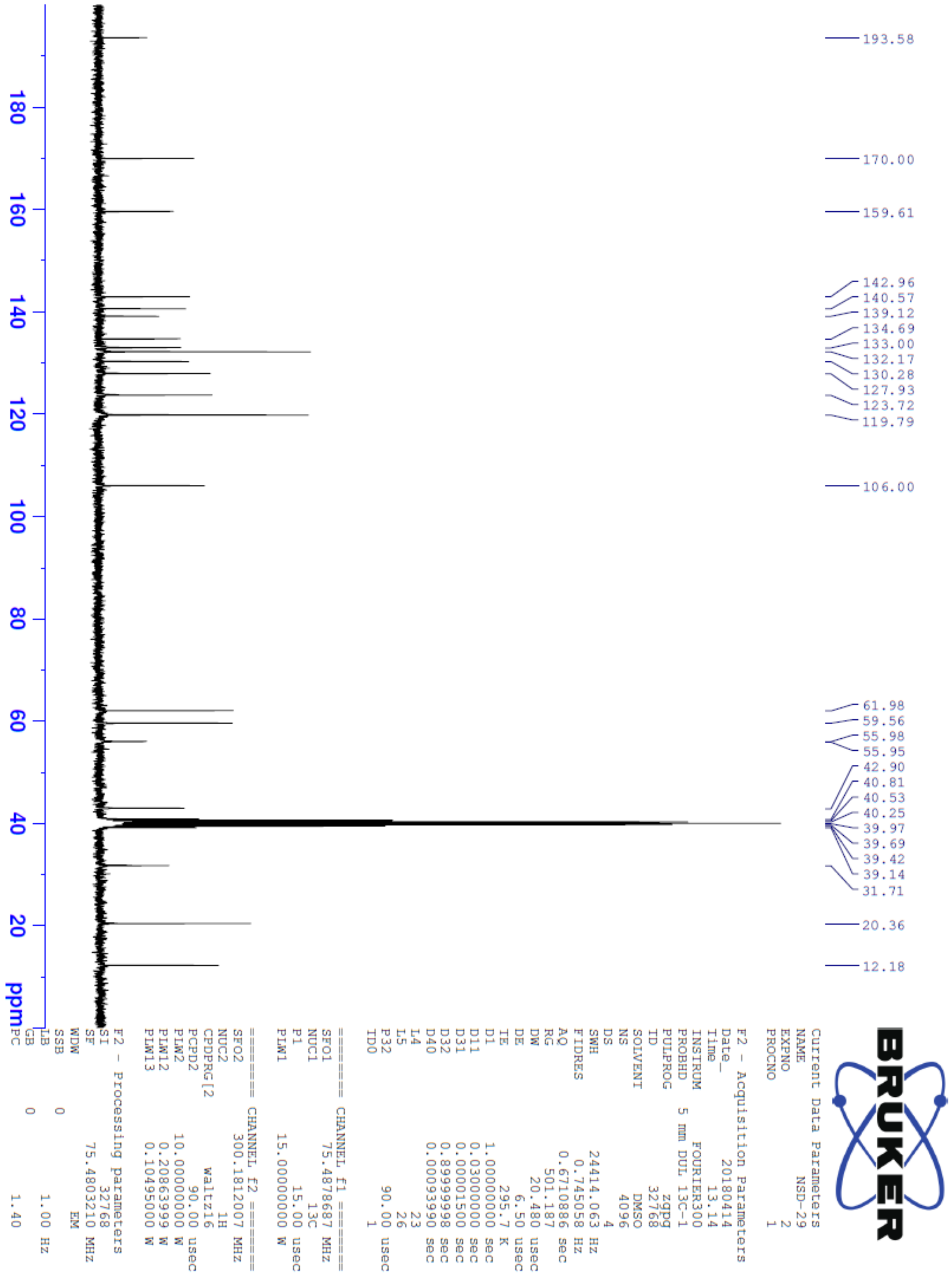

Figure S167. Compound **D29** ^{13}C-NMR spectrum.

Formula Predictor Report - NSD-29_67.lcd

Data File: C:\LabSolutions\Data\Analiz\Serkan\NSD-29_67.lcd

Elmt	Val.	Min	Max	Elmt	Val.	Min	Max	Elmt	Val.	Min	Max	Elmt	Val.	Min	Max	Use Adduct
H	1	5	40	O	2	3	5	S	2	0	0	Ru	2	0	0	H
C	4	0	35	F	1	0	0	Cl	1	0	0	I	3	0	0	
N	3	2	6	P	3	0	0	Br	1	0	0					

Error Margin (ppm):	10	DBE Range:	10.0 - 17.0	Electron Ions:	both
HC Ratio:	unlimited	Apply N Rule:	yes	Use MSn Info:	yes
Max Isotopes:	3	Isotope RI (%):	1.00	Isotope Res:	9000
MSn Iso RI (%):	10.00	MSn Logic Mode:	AND	Max Results:	500

Event#: 1 MS(E+) Ret. Time : 2.213 -> 2.373 Scan# : 333 -> 357

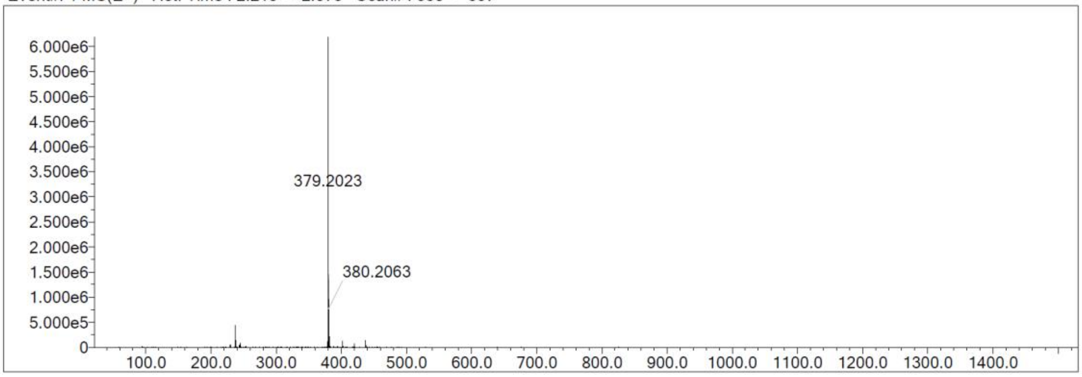

Measured region for 379.2023 m/z

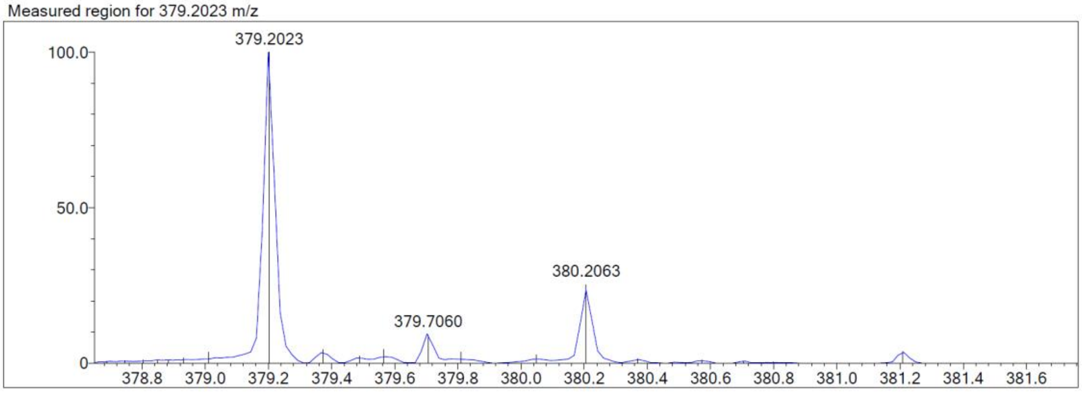

C23 H26 N2 O3 [M+H]+ : Predicted region for 379.2016 m/z

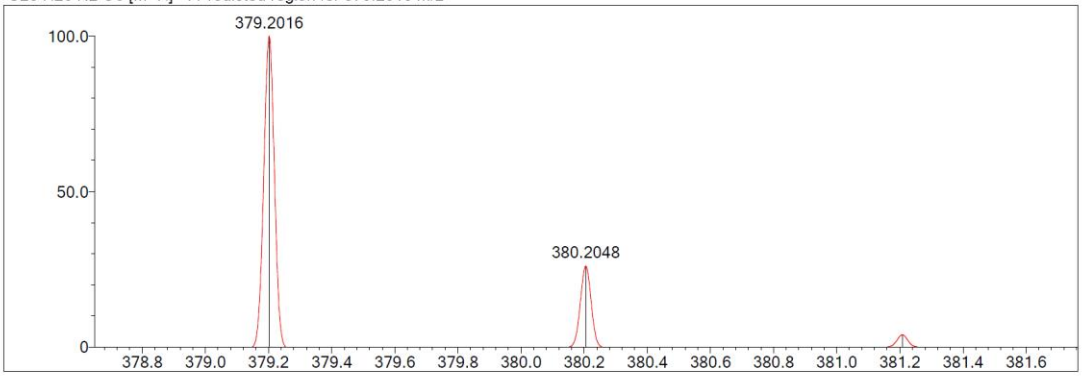

Rank	Score	Formula (M)	Ion	Meas. m/z	Pred. m/z	Df. (mDa)	Df. (ppm)	Iso	DBE
1	89.55	C23 H26 N2 O3	[M+H]+	379.2023	379.2016	0.7	1.85	91.50	12.0

Figure S168. Compound **D29** HRMS report.

*2-(N-methyl-N-propylamino)-N-(4-((5,6-dimethoxy-1-oxo-2,3-dihydro-1H-inden-2-ylidene) methyl)phenyl)acetamide (**D30**)*

Dark brown powder. M.P.: 130.4 °C. Yield: 84%.

IR (ATR) ν_{max} (cm^{-1}): 3317 (N-H), 1710 (indanone C=O), 1630 (amide C=O), 1583-1496 (C=C), 1159 (C-N), 1093 (C-O), 842 (1,4-disubstituted benzene).

^{1}H-NMR (300 MHz, DMSO-*d$_6$*) δ (ppm): 0.88 (3H, t, *J*=7.35 Hz, CH_3), 1.46 (2H, st, *J*=7.34 Hz, CH_2), 2.30 (3H, s, CH_3), 2.40 (2H, t, *J*=7.43 Hz, CH_2), 3.15 (2H, m, CH_2), 3.83 (3H, s, OCH_3), 3.90 (3H, s, OCH_3), 3.97 (2H, s, CH_2), 7.20 (1H, s, methoxy-1-oxo-indenylidene CH), 7.21 (1H, s, methoxy-1-oxo-indenylidene CH), 7.38 (1H, s, C=CH), 7.70 (2H, d, *J*=8.77 Hz, disubstituted benzene CH), 7.79 (2H, d, *J*=8.77 Hz, disubstituted benzene CH), 9.87 (1H, s, NH).

^{13}C-NMR (75 MHz, DMSO-*d$_6$*) δ (ppm): 12.2, 20.4, 32.1, 42.9, 56.1, 56.4, 59.6, 62.0, 105.0, 108.5, 119.8, 130.5, 130.6, 131.3, 131.8, 134.8, 140.2, 145.4, 149.7, 155.6, 169.9, 192.3.

HRMS (ESI) (m/z) $[M+H]^+$: $C_{24}H_{28}N_2O_4$ calculated: 409.2122, found: 409.2132.

DOPNALAB

	Item	Value
	Acquired Date&Time	22.08.2019 13:10:06
	Acquired by	System Administrator
	Filename	C:\Users\dopnalab\Desktop\NURPELİN\DOKTORA TEZ\D301.ispd
	Spectrum name	D301
	Sample name	D30
	Sample ID	
	Option	
	Comment	
	No. of Scans	50
	Resolution	4 [cm-1]
	Apodization	Happ-Genzel

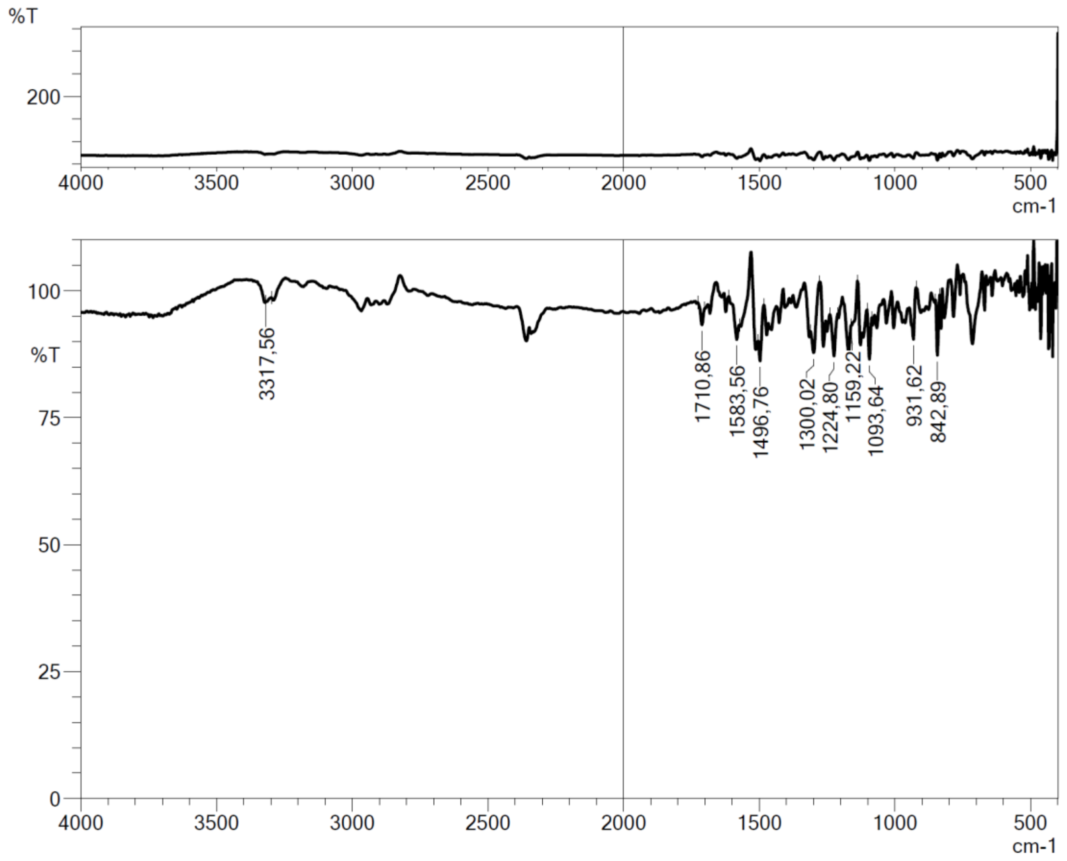

Figure S169. Compound **D30** IR report.

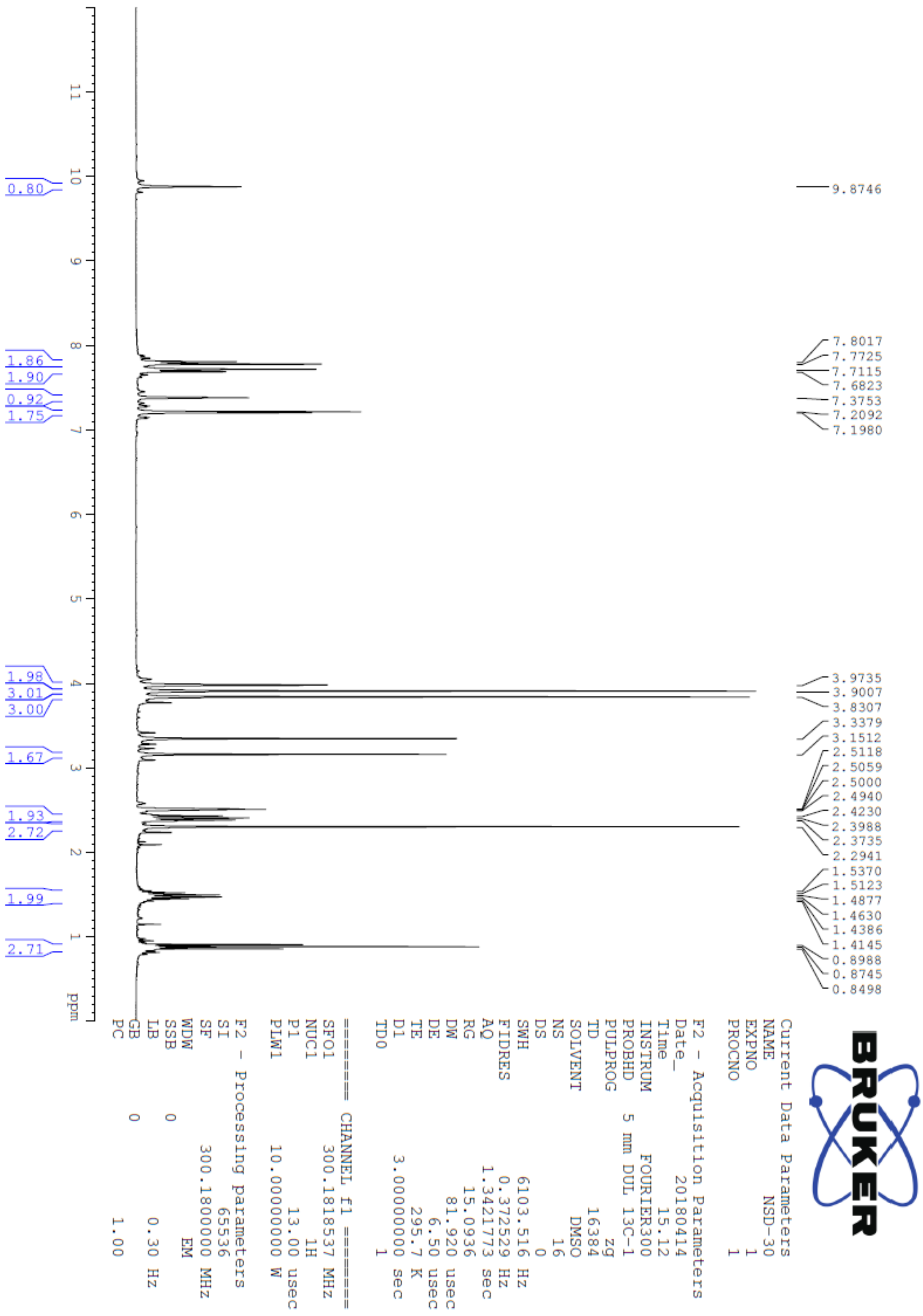

Figure S170. Compound **D30** ^{1}H-NMR spectrum.

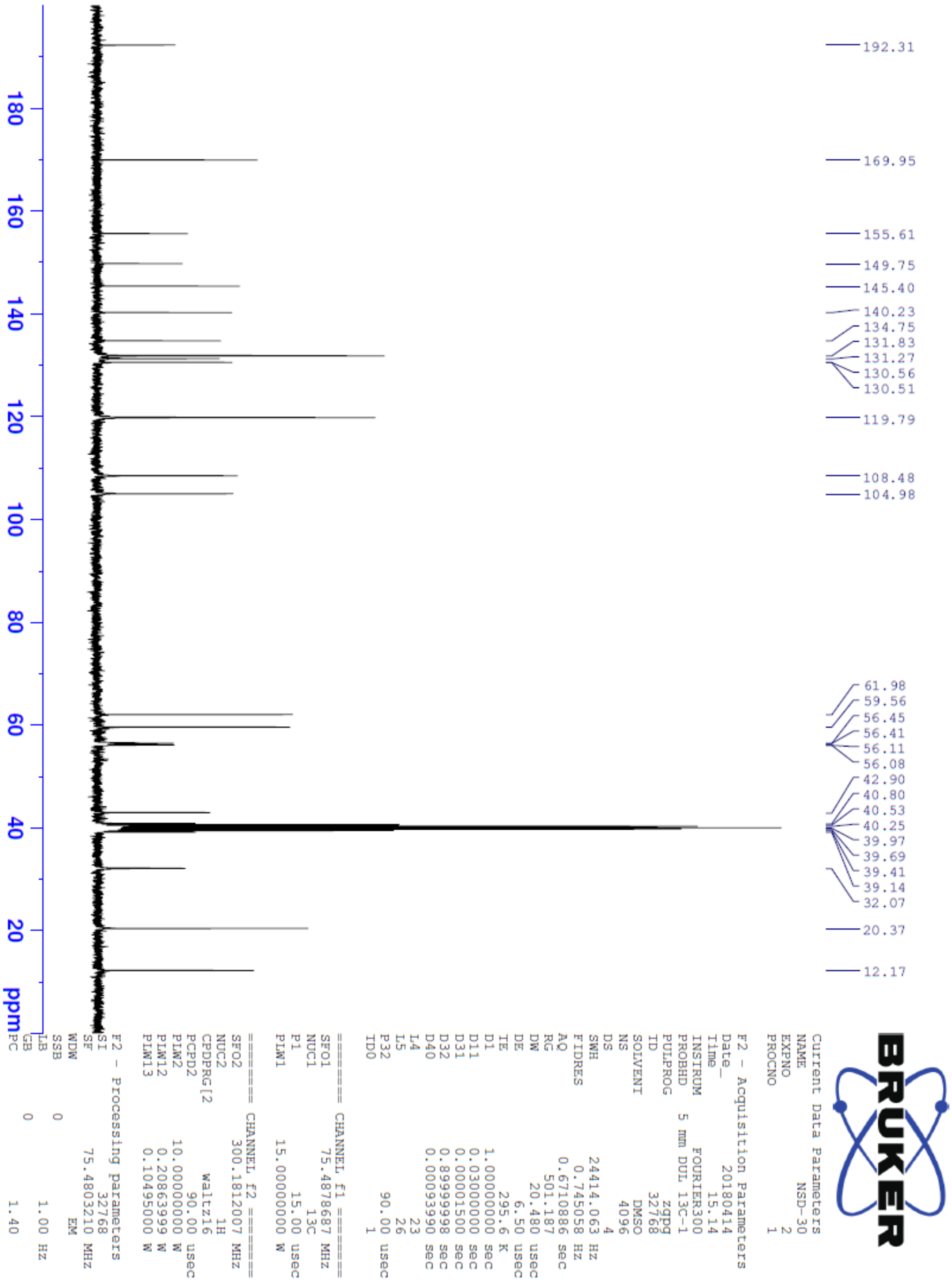

Figure S171. Compound **D30** ^{13}C-NMR spectrum.

Formula Predictor Report - NSD-30_68.lcd Page 1 of 1

Data File: C:\LabSolutions\Data\Analiz\Serkan\NSD-30_68.lcd

Elmt	Val.	Min	Max	Elmt	Val.	Min	Max	Elmt	Val.	Min	Max	Elmt	Val.	Min	Max	Use Adduct
H	1	5	40	O	2	3	5	S	2	0	0	Ru	2	0	0	H
C	4	0	35	F	1	0	0	Cl	1	0	0	I	3	0	0	
N	3	2	6	P	3	0	0	Br	1	0	0					

Error Margin (ppm):	10	DBE Range:	10.0 - 17.0	Electron Ions:	both
HC Ratio:	unlimited	Apply N Rule:	yes	Use MSn Info:	yes
Max Isotopes:	3	Isotope RI (%):	1.00	Isotope Res:	9000
MSn Iso RI (%):	10.00	MSn Logic Mode:	AND	Max Results:	500

Event#: 1 MS(E+)   Ret. Time : 2.147 -> 2.333   Scan# : 323 -> 351

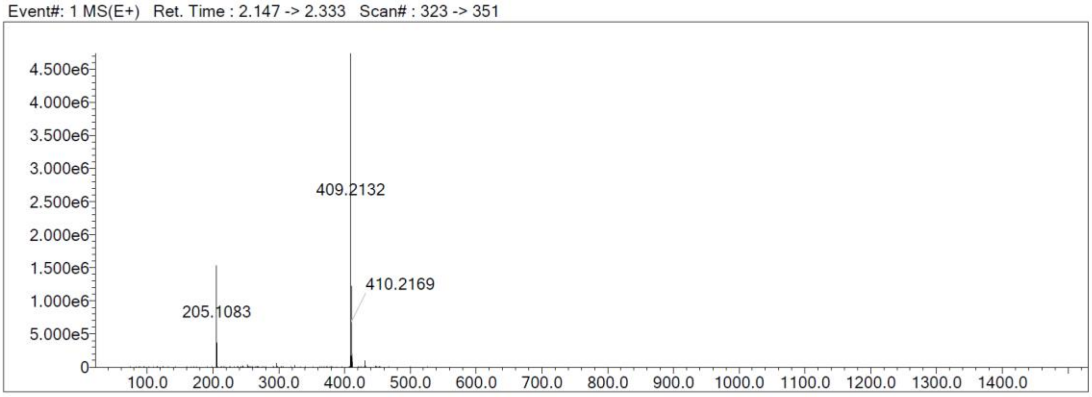

Measured region for 409.2132 m/z

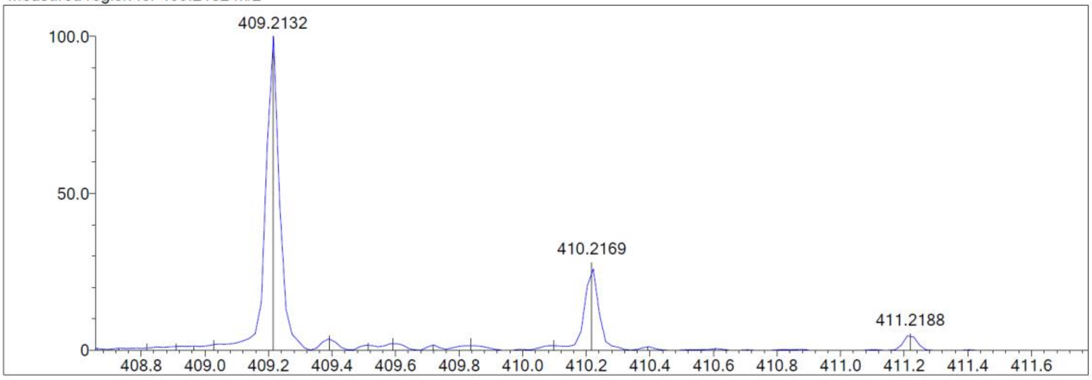

C24 H28 N2 O4 [M+H]+ : Predicted region for 409.2122 m/z

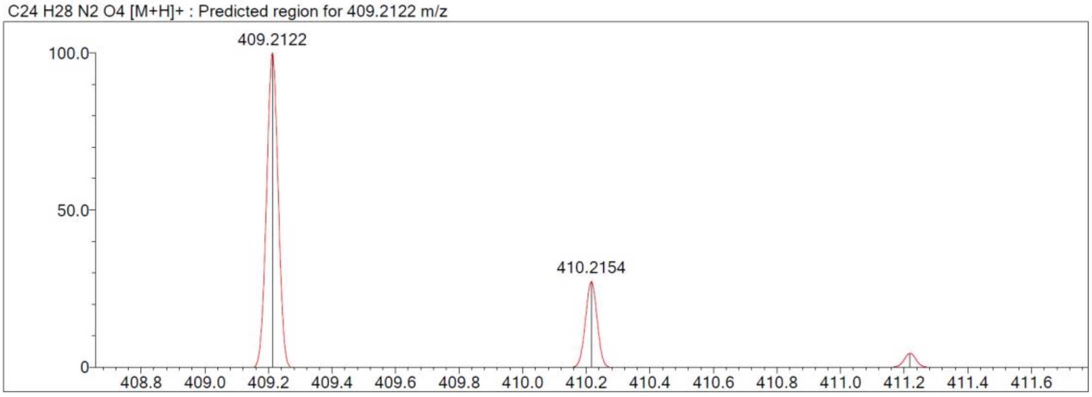

Rank	Score	Formula (M)	Ion	Meas. m/z	Pred. m/z	Df. (mDa)	Df. (ppm)	Iso	DBE
1	83.22	C24 H28 N2 O4	[M+H]+	409.2132	409.2122	1.0	2.44	86.32	12.0

Figure S172. Compound **D30** HRMS report.

*2-(N-methyl-N-propargyl-amino)-N-(4-((5-methoxy-1-oxo-2,3-dihydro-1H-inden-2-ylidene) methyl)phenyl)acetamide (**D31**)*

Dark brown powder. M.P.: 102.4 °C. Yield: 79%.

IR (ATR) ν_{max} (cm^{-1}): 3288 (N-H), 2968-2872 (aliphatic C-H), 1685 (indanone C=O), 1630 (amide C=O), 1583-1512 (C=C), 1226 (C-N), 1091 (C-O), 842 (1,4-disubstituted benzene).

^{1}H-NMR (300 MHz, DMSO-d_6) δ (ppm): 0.87 (2H, s, *J*=7.35 Hz, CH_3), 1.47 (2H, s, *J*=7.35 Hz, CH_2), 2.30 (3H, s, CH_3), 2.40 (2H, t, *J*=7.41 Hz, CH_2), 3.15 (2H, m, CH_2), 3.89 (3H, s, OCH_3), 4.04 (2H, s, CH_2), 7.02 (1H, dd, J_1=8.49 Hz, J_2=2.25 Hz, methoxy-1-oxo-indenylidene CH), 7.17 (1H, *J*=1.98 Hz, methoxy-1-oxo-indenylidene CH), 7.40 (1H, s, C=CH), 7.70-7.72 (3H, m, disubstituted benzene CH, methoxy-1-oxo-indenylidene CH), 7.79 (2H, d, *J*=8.76 Hz, disubstituted benzene CH), 9.89 (1H, s, NH).

^{13}C-NMR (75 MHz, DMSO-d_6) δ (ppm): 12.2, 20.4, 32.5, 42.9, 56.3, 59.6, 62.0, 110.6, 115.8, 119.8, 125.8, 130.4, 131.1, 131.7, 131.9, 134.5, 140.3, 153.3, 165.3, 169.9, 192.0.

HRMS (ESI) (m/z) $[M+H]^+$: $C_{23}H_{22}N_2O_3$ calculated: 375.1703, found: 375.1712.

DOPNALAB

	Item	Value
	Acquired Date&Time	22.08.2019 13:13:08
	Acquired by	System Administrator
	Filename	C:\Users\dopnalab\Desktop\NURPELİN\DOKTORA TEZ\D311.ispd
	Spectrum name	D311
	Sample name	D31
	Sample ID	
	Option	
	Comment	
	No. of Scans	50
	Resolution	4 [cm-1]
	Apodization	Happ-Genzel

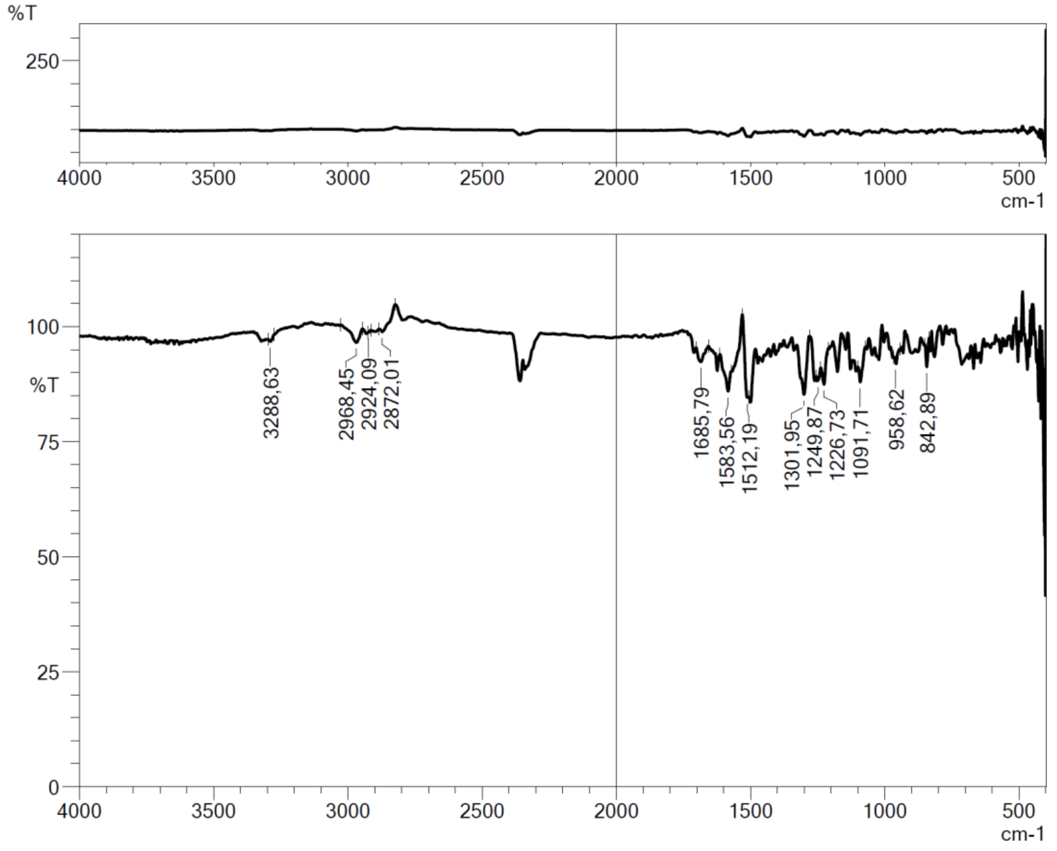

Figure S173. Compound **D31** IR report.

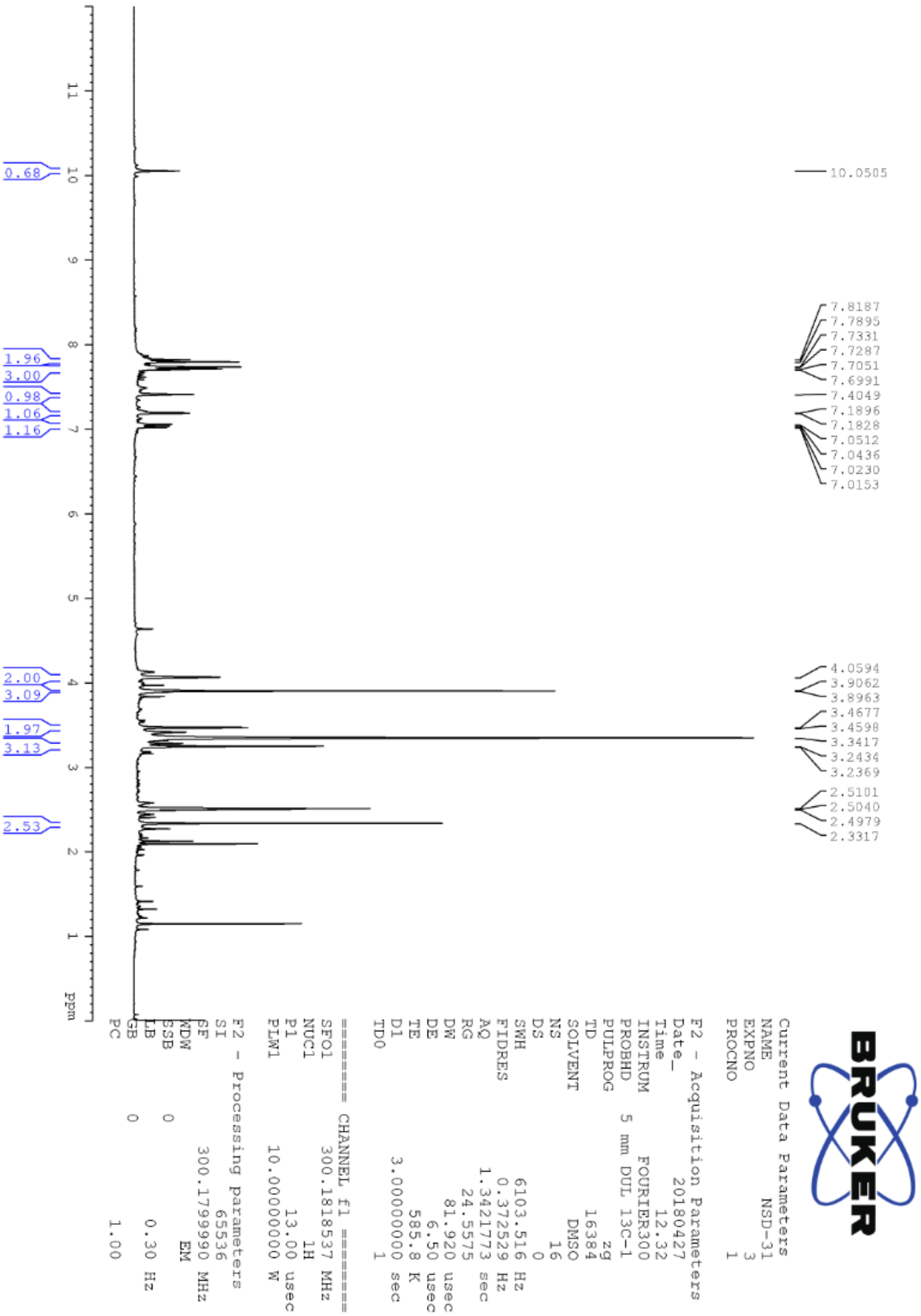

Figure S174. Compound **D31** ^{1}H-NMR spectrum.

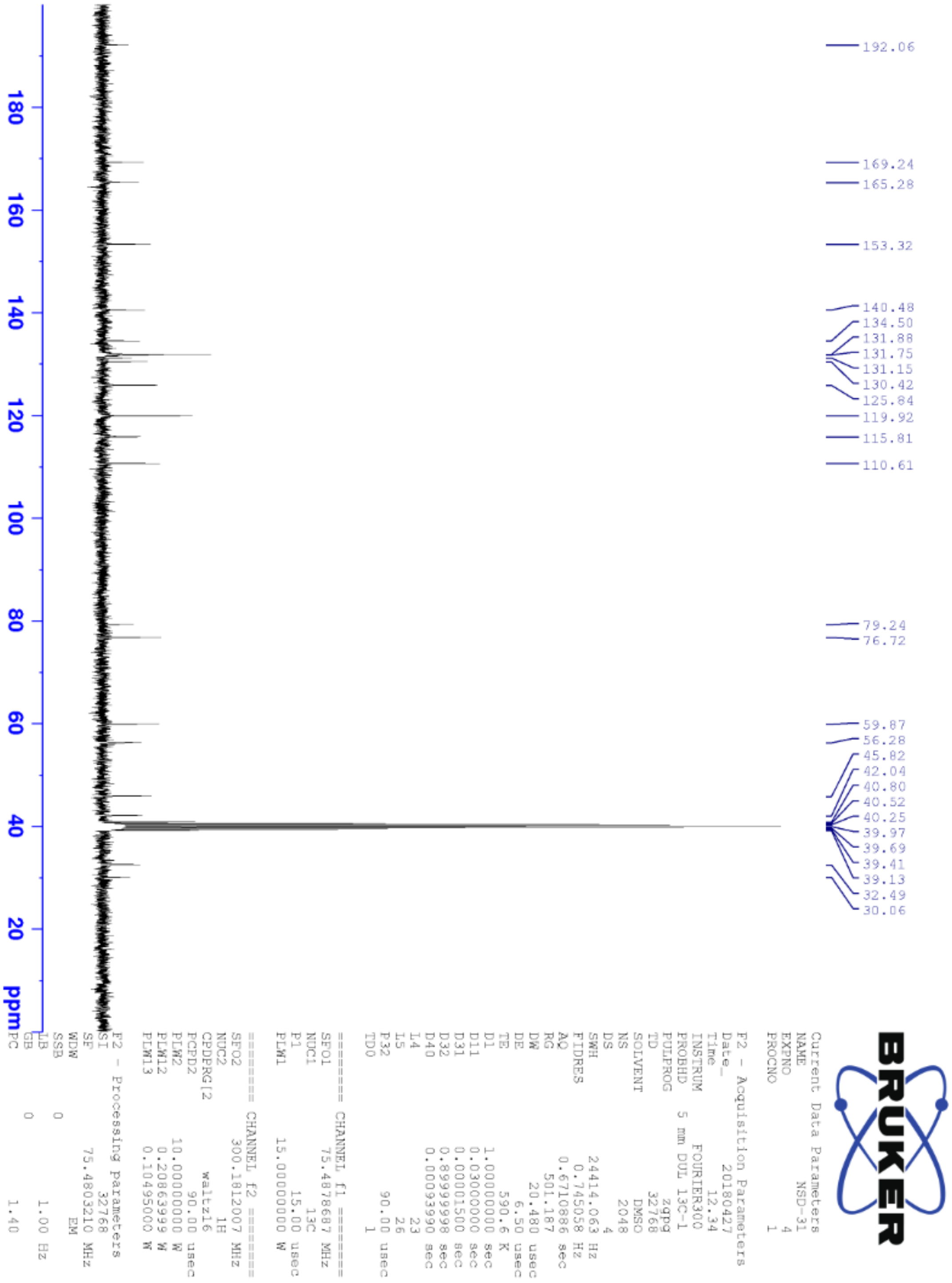

Figure S175. Compound **D31** ^{13}C-NMR spectrum.

Data File: C:\LabSolutions\Data\Analiz\Serkan\NSD-31_69.lcd

Elmt	Val.	Min	Max	Elmt	Val.	Min	Max	Elmt	Val.	Min	Max	Elmt	Val.	Min	Max	Use Adduct
H	1	5	40	O	2	3	5	S	2	0	0	Ru	2	0	0	H
C	4	0	35	F	1	0	0	Cl	1	0	0	I	3	0	0	
N	3	2	6	P	3	0	0	Br	1	0	0					

Error Margin (ppm): 10
HC Ratio: unlimited
Max Isotopes: 3
MSn Iso RI (%): 10.00
DBE Range: 10.0 - 17.0
Apply N Rule: yes
Isotope RI (%): 1.00
MSn Logic Mode: AND
Electron Ions: both
Use MSn Info: yes
Isotope Res: 9000
Max Results: 500

Event#: 1 MS(E+) Ret. Time : 2.320 -> 2.480 Scan# : 349 -> 373

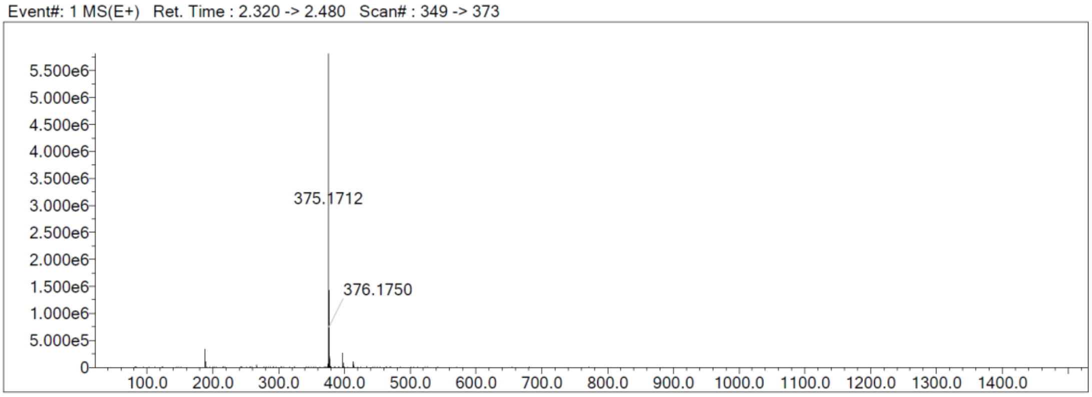

Measured region for 375.1712 m/z

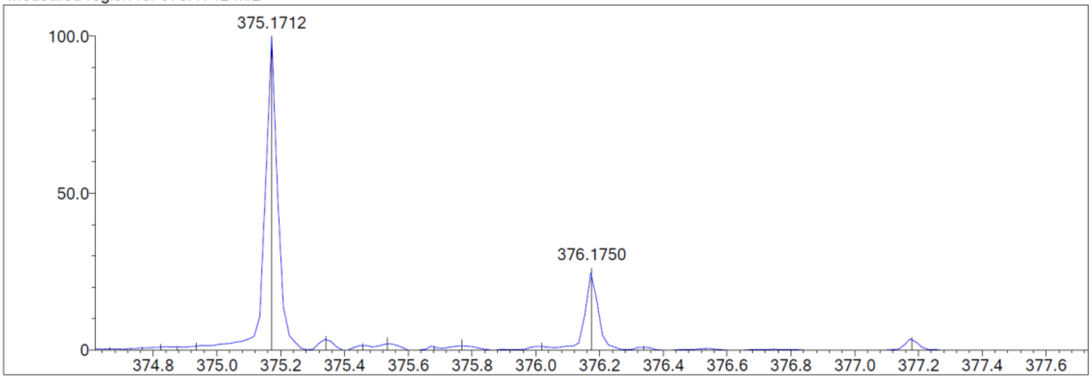

C23 H22 N2 O3 [M+H]+ : Predicted region for 375.1703 m/z

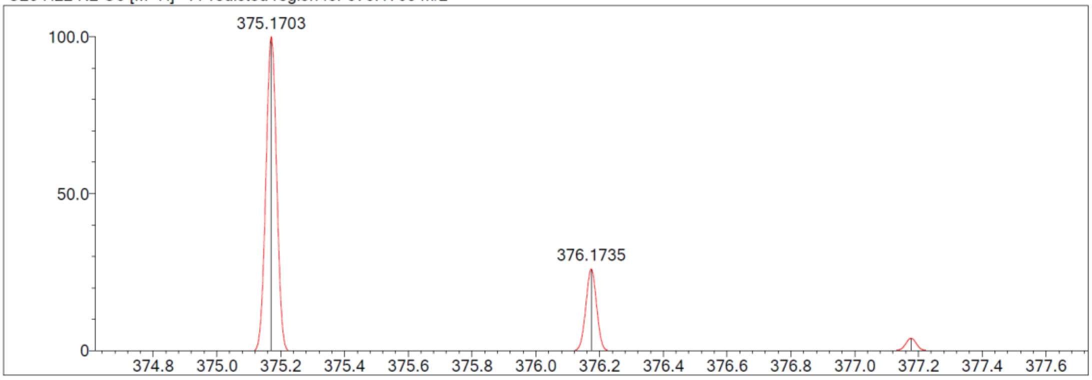

Rank	Score	Formula (M)	Ion	Meas. m/z	Pred. m/z	Df. (mDa)	Df. (ppm)	Iso	DBE
1	96.50	C23 H22 N2 O3	[M+H]+	375.1712	375.1703	0.9	2.40	100.00	14.0

Figure S176. Compound **D31** HRMS report.

*2-(N-methyl-N-propargyl-amino)-N-(4-((6-methoxy-1-oxo-2,3-dihydro-1H-inden-2-ylidene) methyl)phenyl)acetamide (**D32**)*

Yellow powder. M.P.: 118.7 °C. Yield: 81%.

IR (ATR) ν_{max} (cm^{-1}): 3305 (N-H), 2970-2897 (aliphatic C-H), 1681 (indanone C=O), 1624 (amide C=O), 1585-1514 (C=C), 1178 (C-N), 1091 (C-O), 821 (1,4-disubstituted benzene).

^{1}H-NMR (300 MHz, DMSO-*d*$_6$) δ (ppm): 2.33 (3H, s, CH_3), 3.23-3.24 (3H, m, CH, CH_2), 3.46 (1H, d, *J*=2.34 Hz, CH_2), 3.84 (3H, s, OCH_3), 4.02 (2H, s, CH_2), 7.25 (1H, d, *J*=2.49 Hz, methoxy-1-oxo-indenylidene CH), 7.28 (1H, dd, J_1=8.34 Hz, J_2=2.55 Hz, methoxy-1-oxo-indenylidene CH), 7.48 (1H, s, C=CH), 7.58 (1H, d, *J*=8.38 Hz, methoxy-1-oxo-indenylidene CH), 7.74 (2H, d, *J*=8.86 Hz, disubstituted benzene CH), 7.81 (2H, d, *J*=8.80 Hz, disubstituted benzene CH), 10.04 (1H, s, NH).

^{13}C-NMR (75 MHz, DMSO-*d*$_6$) δ (ppm): 31.75, 42.0, 45.8, 56.0, 59.9, 63.2, 76.7, 79.2, 106.0, 119.9, 123.7, 127.9, 130.3, 132.1, 133.0, 134.7, 139.1, 140.7, 143.0, 159.6, 169.3, 193.6.

HRMS (ESI) (m/z) $[M+H]^+$: $C_{23}H_{22}N_2O_3$ calculated: 375.1703, found: 375.1711.

DOPNALAB

	Item	Value
	Acquired Date&Time	22.08.2019 13:15:50
	Acquired by	System Administrator
	Filename	C:\Users\dopnalab\Desktop\NURPELİN\DOKTORA TEZ\D321.ispd
	Spectrum name	D321
	Sample name	D32
	Sample ID	
	Option	
	Comment	
	No. of Scans	50
	Resolution	4 [cm-1]
	Apodization	Happ-Genzel

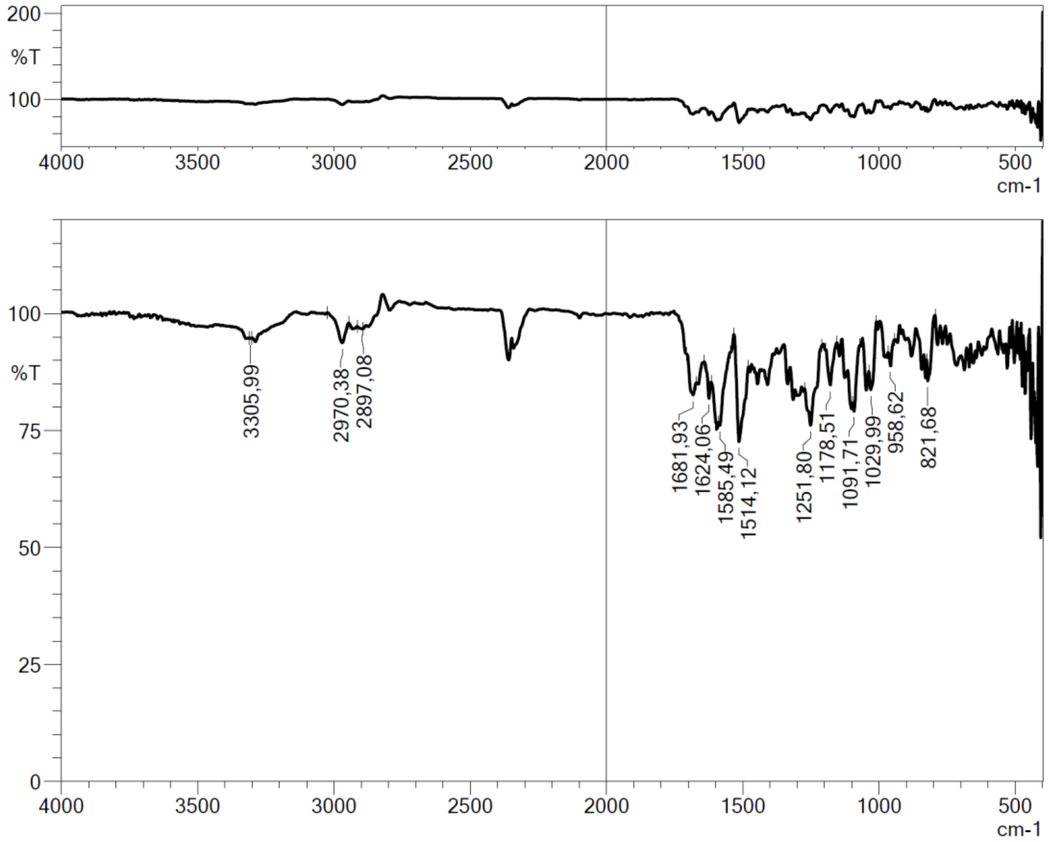

Figure S177. Compound **D32** IR report.

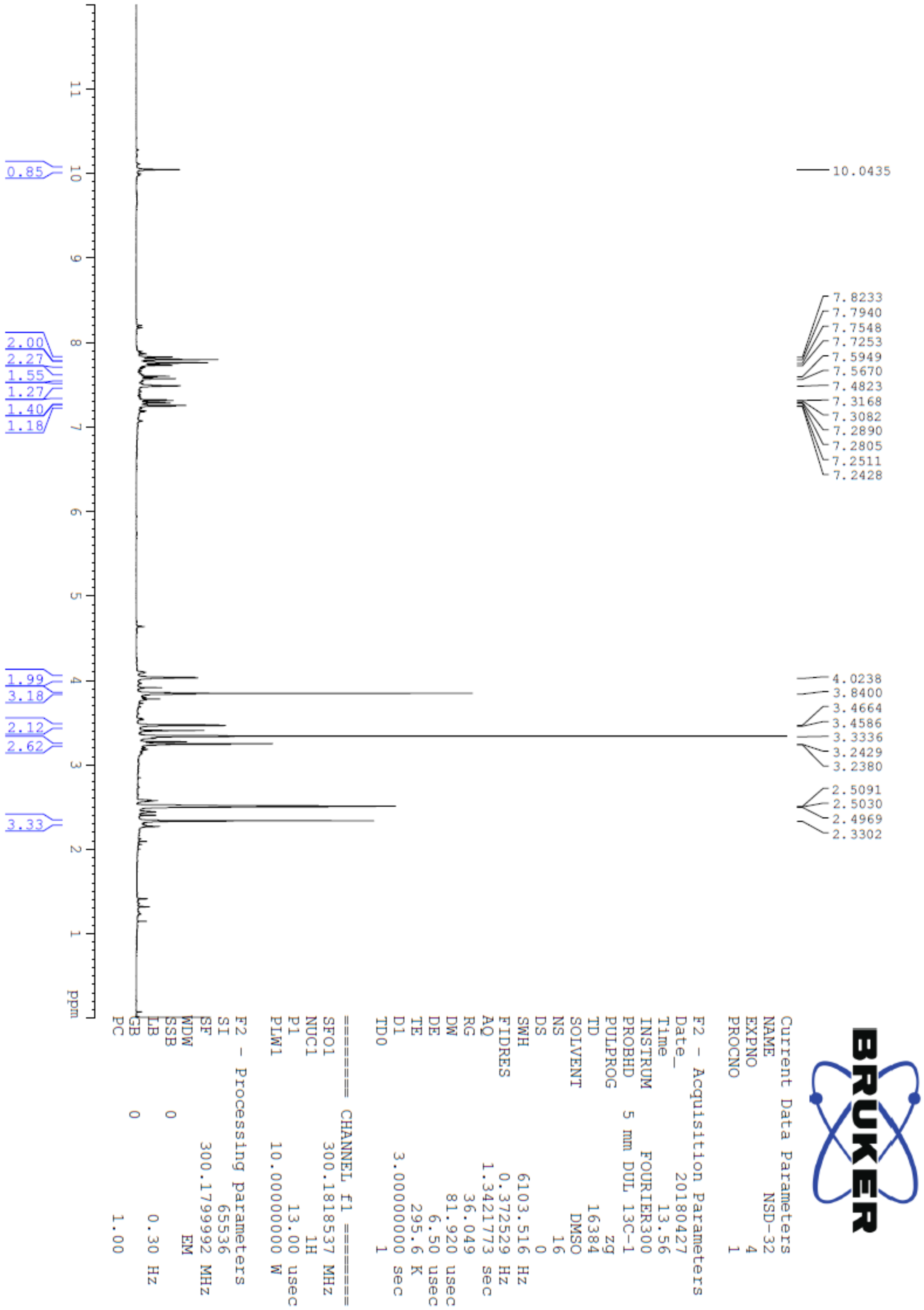

Figure S178. Compound **D32** ^{1}H-NMR spectrum.

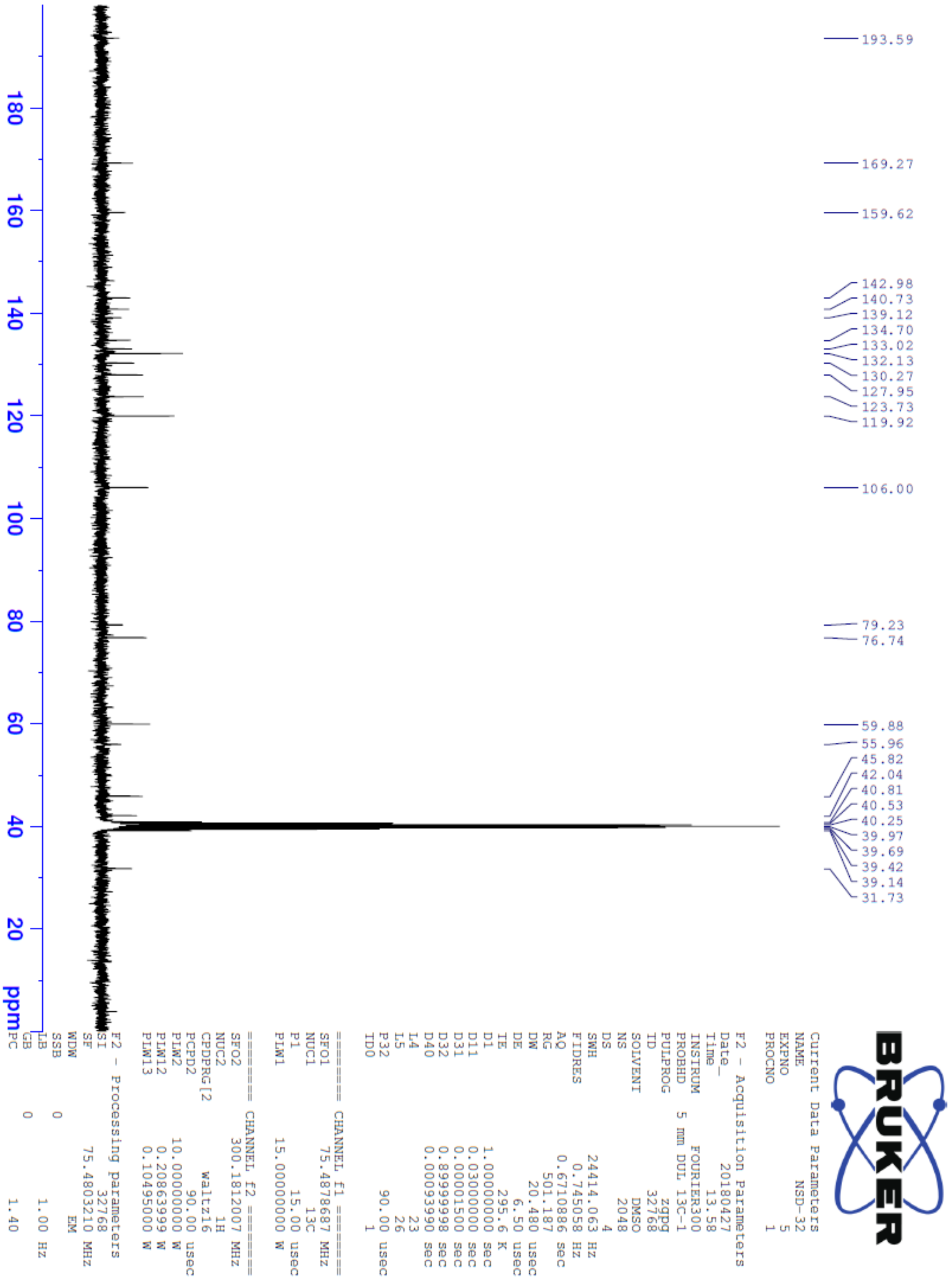

Figure S179. Compound **D32** ^{13}C-NMR spectrum.

Data File: C:\LabSolutions\Data\Analiz\Serkan\NSD-32_71.lcd

Elmt	Val.	Min	Max	Elmt	Val.	Min	Max	Elmt	Val.	Min	Max	Elmt	Val.	Min	Max	Use Adduct
H	1	5	40	O	2	3	5	S	2	0	0	Ru	2	0	0	H
C	4	0	35	F	1	0	0	Cl	1	0	0	I	3	0	0	
N	3	2	6	P	3	0	0	Br	1	0	0					

Error Margin (ppm):	10	DBE Range:	10.0 - 17.0	Electron Ions:	both
HC Ratio:	unlimited	Apply N Rule:	yes	Use MSn Info:	yes
Max Isotopes:	3	Isotope RI (%):	1.00	Isotope Res:	9000
MSn Iso RI (%):	10.00	MSn Logic Mode:	AND	Max Results:	500

Event#: 1 MS(E+) Ret. Time : 2.307 -> 2.427 Scan# : 347 -> 365

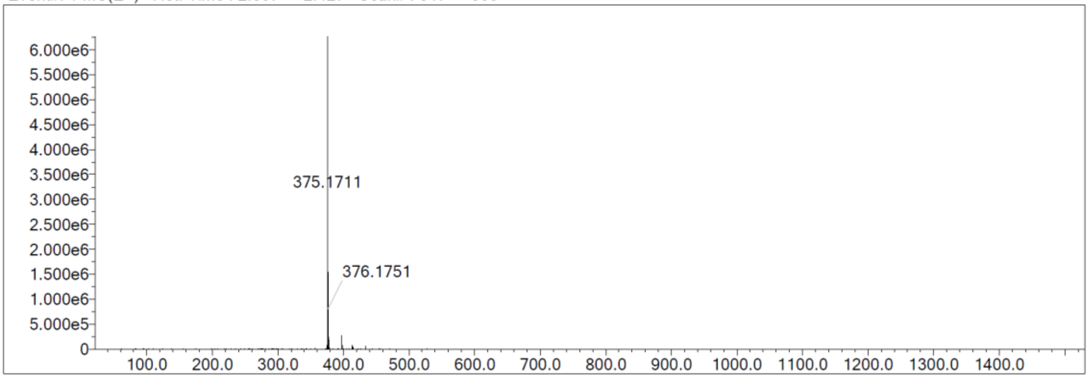

Measured region for 375.1711 m/z

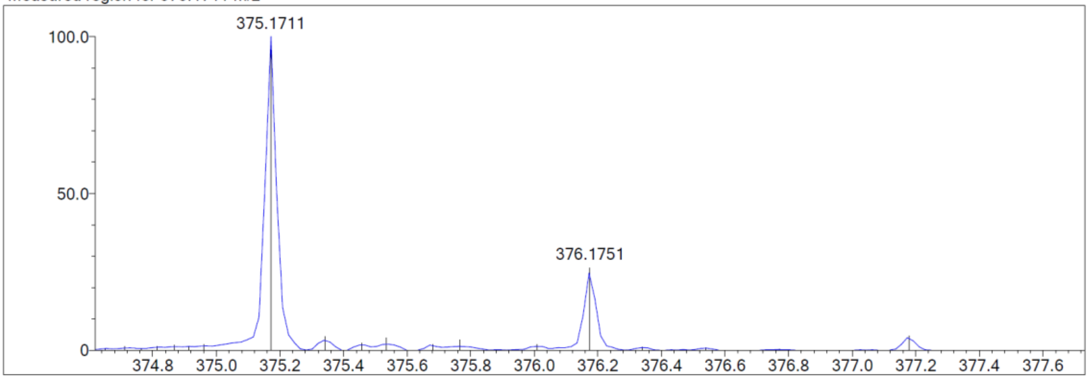

C23 H22 N2 O3 [M+H]+ : Predicted region for 375.1703 m/z

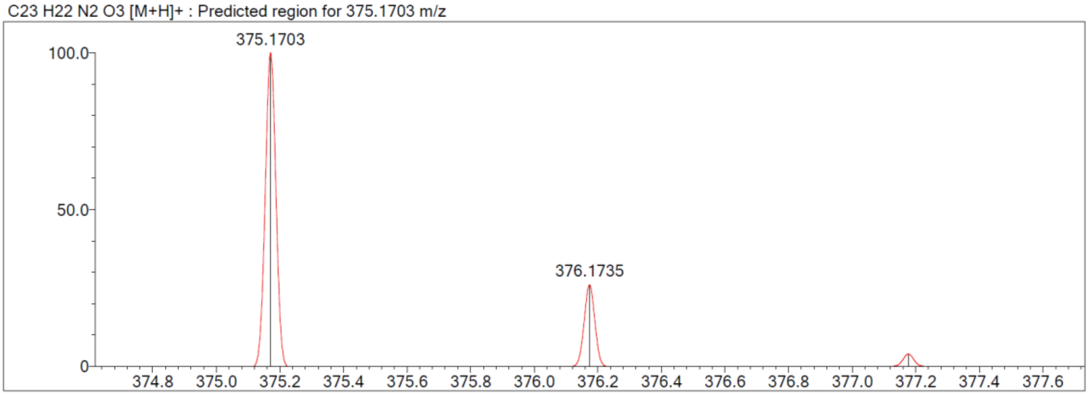

Rank	Score	Formula (M)	Ion	Meas. m/z	Pred. m/z	Df. (mDa)	Df. (ppm)	Iso	DBE
1	97.17	C23 H22 N2 O3	[M+H]+	375.1711	375.1703	0.8	2.13	100.00	14.0

Figure S180. Compound **D32** HRMS report.

*2-(N-methyl-N-propargyl-amino)-N-(4-((5,6-dimethoxy-1-oxo-2,3-dihydro-1H-inden-2-ylidene)methyl)phenyl)acetamide (**D33**)*

Yellow powder. M.P.: 197.8 °C. Yield: 78%.

IR (ATR) ν_{max} (cm^{-1}): 3319 (N-H), 2835 (aliphatic C-H), 1674 (indanone C=O), 1625 (amide C=O), 1589-1498 (C=C), 1132 (C-N), 1095 (C-O), 827 (1,4-disubstituted benzene).

^{1}H-NMR (300 MHz, DMSO-*d$_6$*) δ (ppm): 2.33 (3H, s, CH_3), 3.23-3.24 (3H, m, CH, CH_2), 3.46 (1H, d, *J*=2.28 Hz, CH_2), 3.84 (3H, s, OCH_3), 3.91 (3H, s, OCH_3), 3.99 (2H, s, CH_2), 7.21 (1H, s, methoxy-1-oxo-indenylidene CH), 7.22 (1H, s, methoxy-1-oxo-indenylidene CH), 7.48 (1H, s, C=CH), 7.70 (2H, d, *J*=8.86 Hz, disubstituted benzene CH), 7.79 (2H, d, *J*=8.71 Hz, disubstituted benzene CH), 10.02 (1H, s, NH).

^{13}C-NMR (75 MHz, DMSO-*d$_6$*) δ (ppm): 32.1, 42.0, 45.8, 56.1, 56.4, 59.9, 76.7, 79.2, 105.0, 108.5, 119.9, 130.5, 130.6, 131.3, 131.8, 134.9, 140.4, 145.4, 149.7, 155.6, 169.2, 192.3.

HRMS (ESI) (m/z) $[M+H]^+$: $C_{24}H_{24}N_2O_4$ calculated: 405.1809, found: 405.1821.

DOPNALAB

	Item	Value
	Acquired Date&Time	22.08.2019 13:20:32
	Acquired by	System Administrator
	Filename	C:\Users\dopnalab\Desktop\NURPELİN\DOKTORA TEZ\D331.ispd
	Spectrum name	D331
	Sample name	D33
	Sample ID	
	Option	
	Comment	
	No. of Scans	50
	Resolution	4 [cm-1]
	Apodization	Happ-Genzel

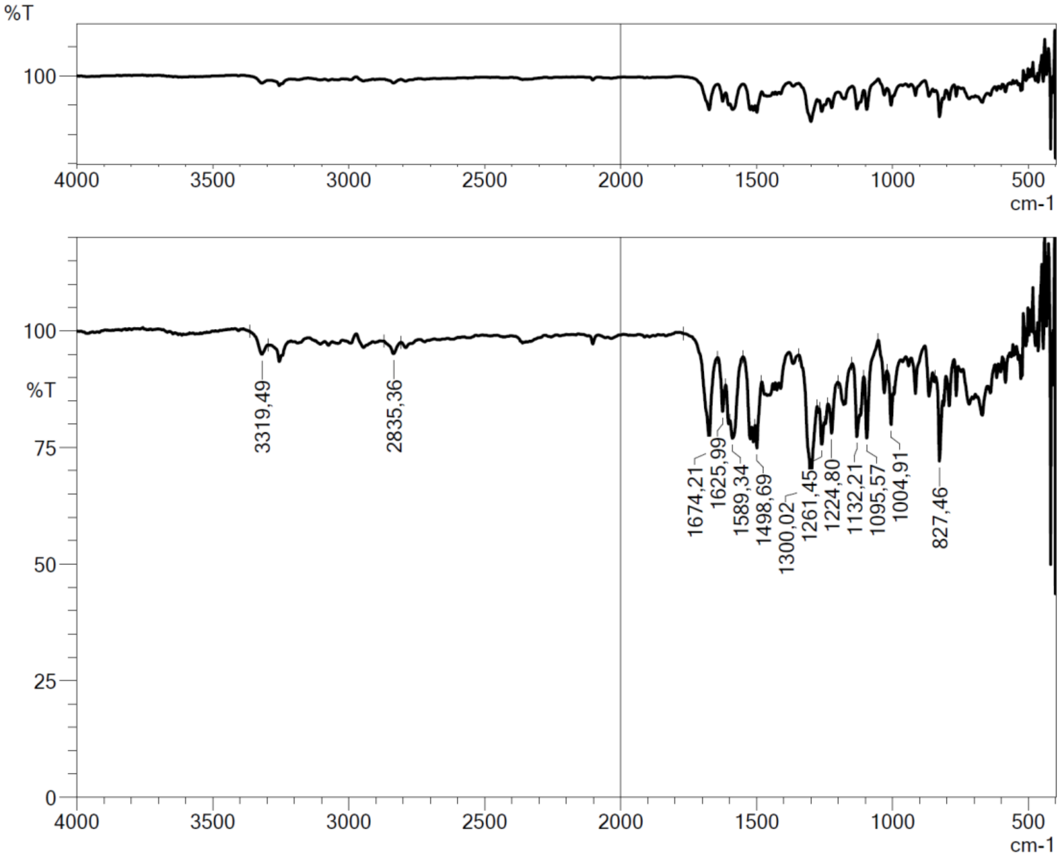

Figure S181. Compound **D33** IR report.

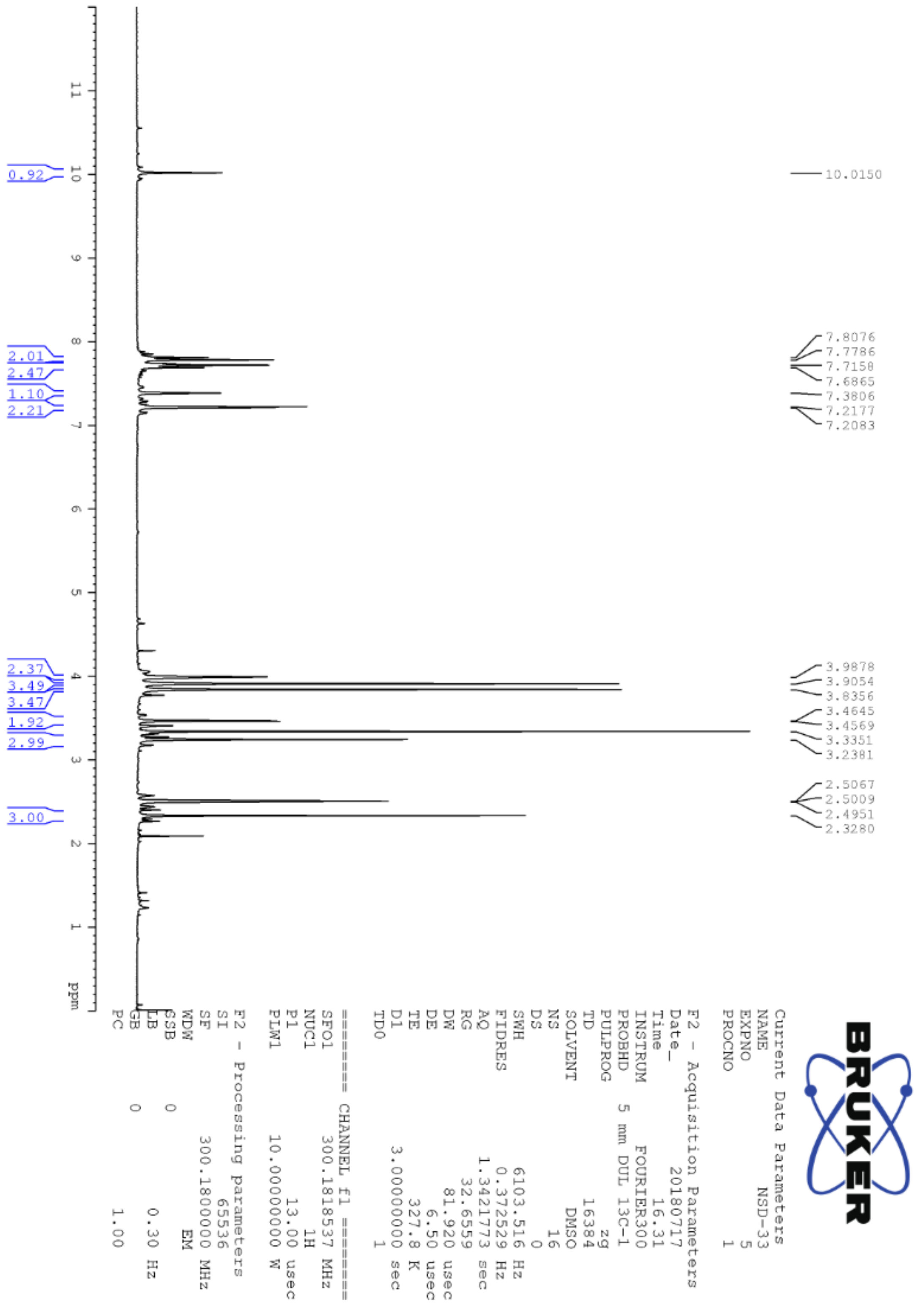

Figure S182. Compound **D33** ^{1}H-NMR spectrum.

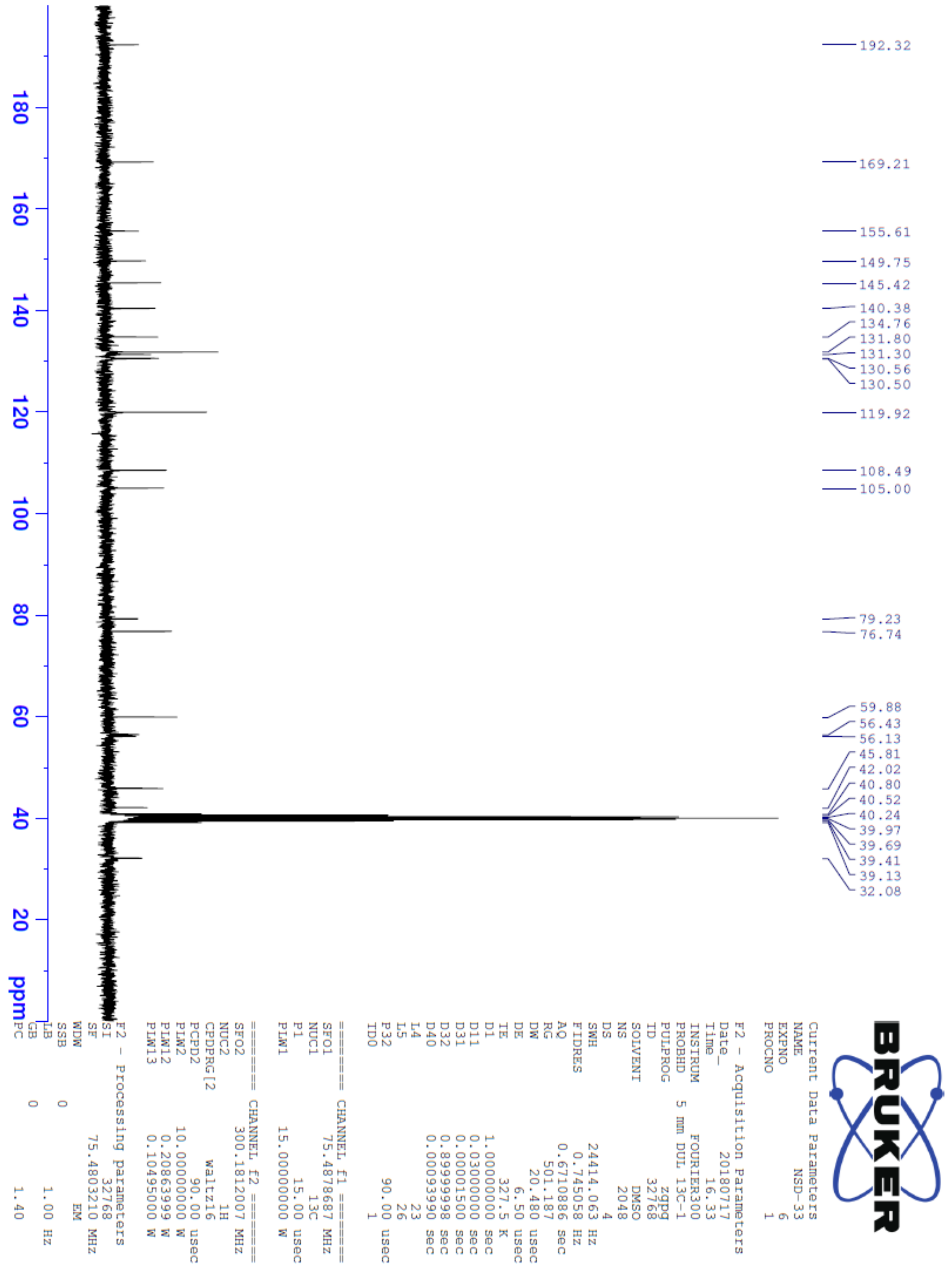

Figure S183. Compound **D33** ^{13}C-NMR spectrum.

Data File: C:\LabSolutions\Data\Analiz\Serkan\NSD-33_72.lcd

Elmt	Val.	Min	Max	Elmt	Val.	Min	Max	Elmt	Val.	Min	Max	Elmt	Val.	Min	Max	Use Adduct
H	1	5	40	O	2	3	5	S	2	0	0	Ru	2	0	0	H
C	4	0	35	F	1	0	0	Cl	1	0	0	I	3	0	0	
N	3	2	6	P	3	0	0	Br	1	0	0					

Error Margin (ppm):	10	DBE Range:	10.0 - 17.0	Electron Ions:	both
HC Ratio:	unlimited	Apply N Rule:	yes	Use MSn Info:	yes
Max Isotopes:	3	Isotope RI (%):	1.00	Isotope Res:	9000
MSn Iso RI (%):	10.00	MSn Logic Mode:	AND	Max Results:	500

Event#: 1 MS(E+) Ret. Time : 2.213 -> 2.373 Scan# : 333 -> 357

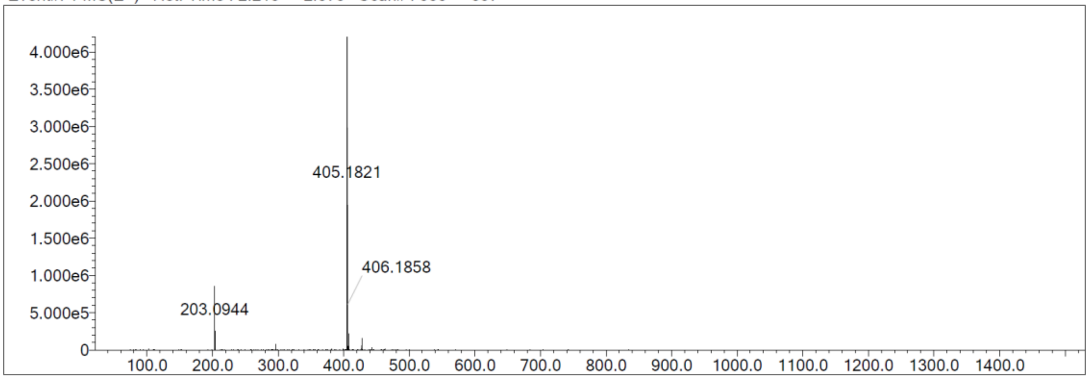

Measured region for 405.1821 m/z

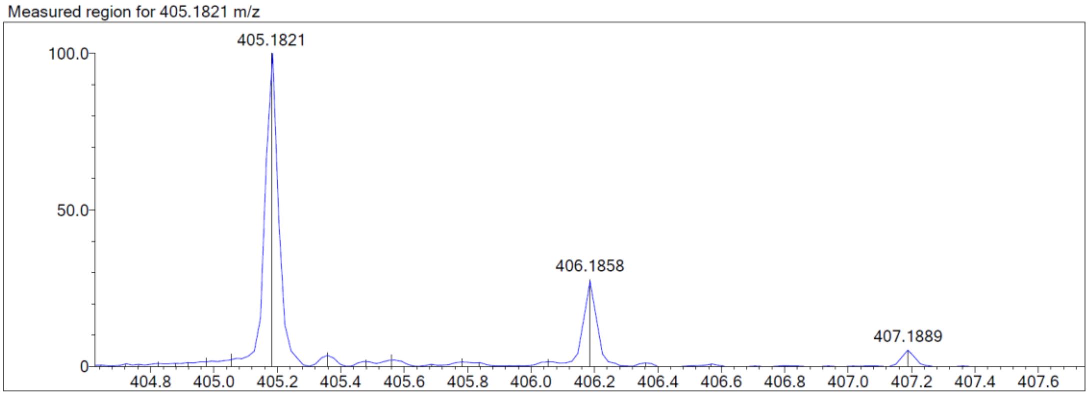

C24 H24 N2 O4 [M+H]+ : Predicted region for 405.1809 m/z

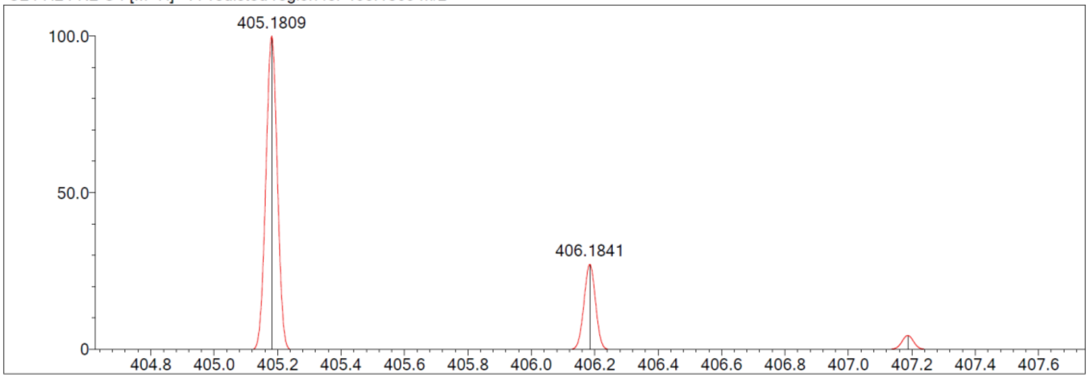

Rank	Score	Formula (M)	Ion	Meas. m/z	Pred. m/z	Df. (mDa)	Df. (ppm)	Iso	DBE
1	82.79	C24 H24 N2 O4	[M+H]+	405.1821	405.1809	1.2	2.96	87.06	14.0

Figure S184. Compound **D33** HRMS report.

*2-(N-methyl-N-(2-(dimethylamino)ethyl)amino)-N-(4-((5-methoxy-1-oxo-2,3-dihydro-1H-inden-2-ylidene)methyl)phenyl)acetamide (**D34**)*

Brown powder. M.P.: 141.1 °C. Yield: 87%.

IR (ATR) ν_{max} (cm^{-1}): 3319 (N-H), 2831 (aliphatic C-H), 1678 (indanone C=O), 1635 (amide C=O), 1579-1514 (C=C), 1165 (C-N), 1095 (C-O), 825 (1,4-disubstituted benzene).

^{1}H-NMR (300 MHz, DMSO-*d$_6$*) δ (ppm): 0.87 (2H, s, *J*=7.35 Hz, CH_3), 1.47 (2H, s, *J*=7.35 Hz, CH_2), 2.30 (3H, s, CH_3), 2.40 (2H, t, *J*=7.41 Hz, CH_2), 3.15 (2H, m, CH_2), 3.89 (3H, s, OCH_3), 4.04 (2H, s, CH_2), 7.02 (1H, dd, J_1=8.49 Hz, J_2=2.25 Hz, methoxy-1-oxo-indenylidene CH), 7.17 (1H, *J*=1.98 Hz, methoxy-1-oxo-indenylidene CH), 7.40 (1H, s, C=CH), 7.70-7.72 (3H, m, disubstituted benzene CH, methoxy-1-oxo-indenylidene CH), 7.79 (2H, d, *J*=8.76 Hz, disubstituted benzene CH), 9.89 (1H, s, NH).

^{13}C-NMR (75 MHz, DMSO-*d$_6$*) δ (ppm): 12.2, 20.4, 32.5, 42.9, 56.3, 59.6, 62.0, 110.6, 115.8, 119.8, 125.8, 130.4, 131.1, 131.7, 131.9, 134.5, 140.3, 153.3, 165.3, 169.9, 192.0.

HRMS (ESI) (m/z) $[M+H]^+$: $C_{24}H_{29}N_3O_3$ calculated: 408.2282, found: 408.2283.

DOPNALAB

	Item	Value
	Acquired Date&Time	22.08.2019 13:23:32
	Acquired by	System Administrator
	Filename	C:\Users\dopnalab\Desktop\NURPELİN\DOKTORA TEZ\D341.ispd
	Spectrum name	D341
	Sample name	D34
	Sample ID	
	Option	
	Comment	
	No. of Scans	50
	Resolution	4 [cm-1]
	Apodization	Happ-Genzel

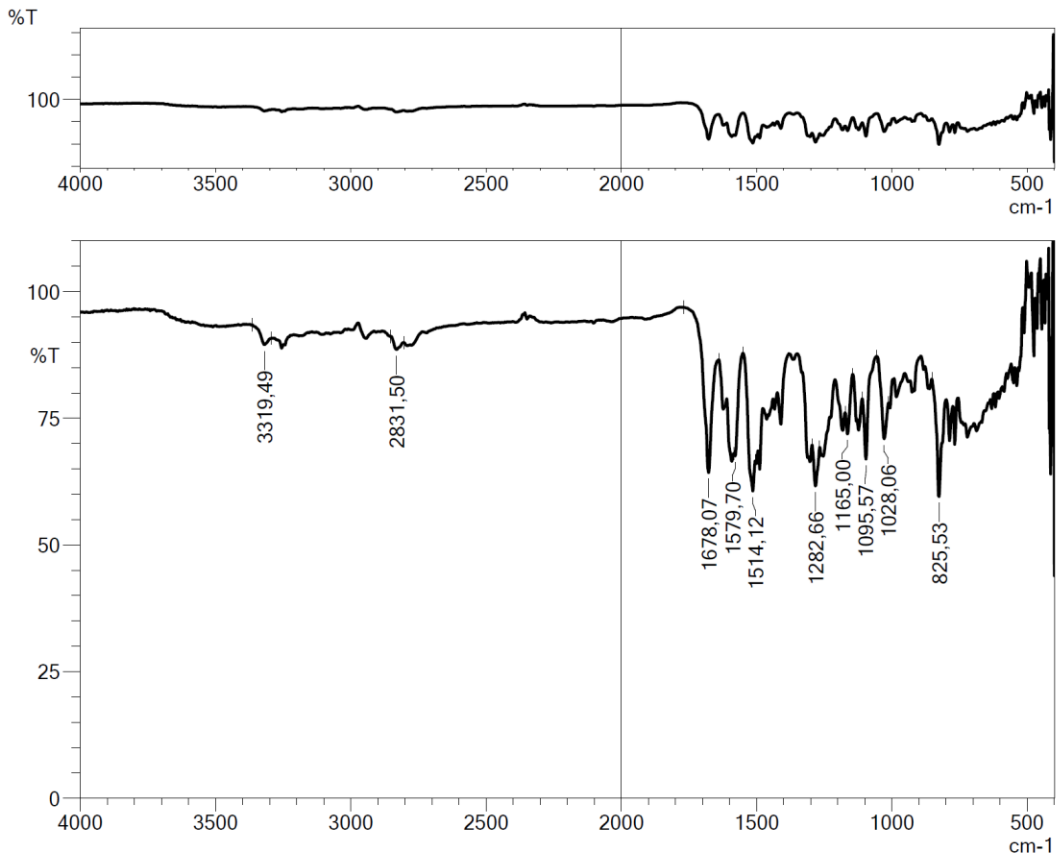

Figure S185. Compound **D34** IR report.

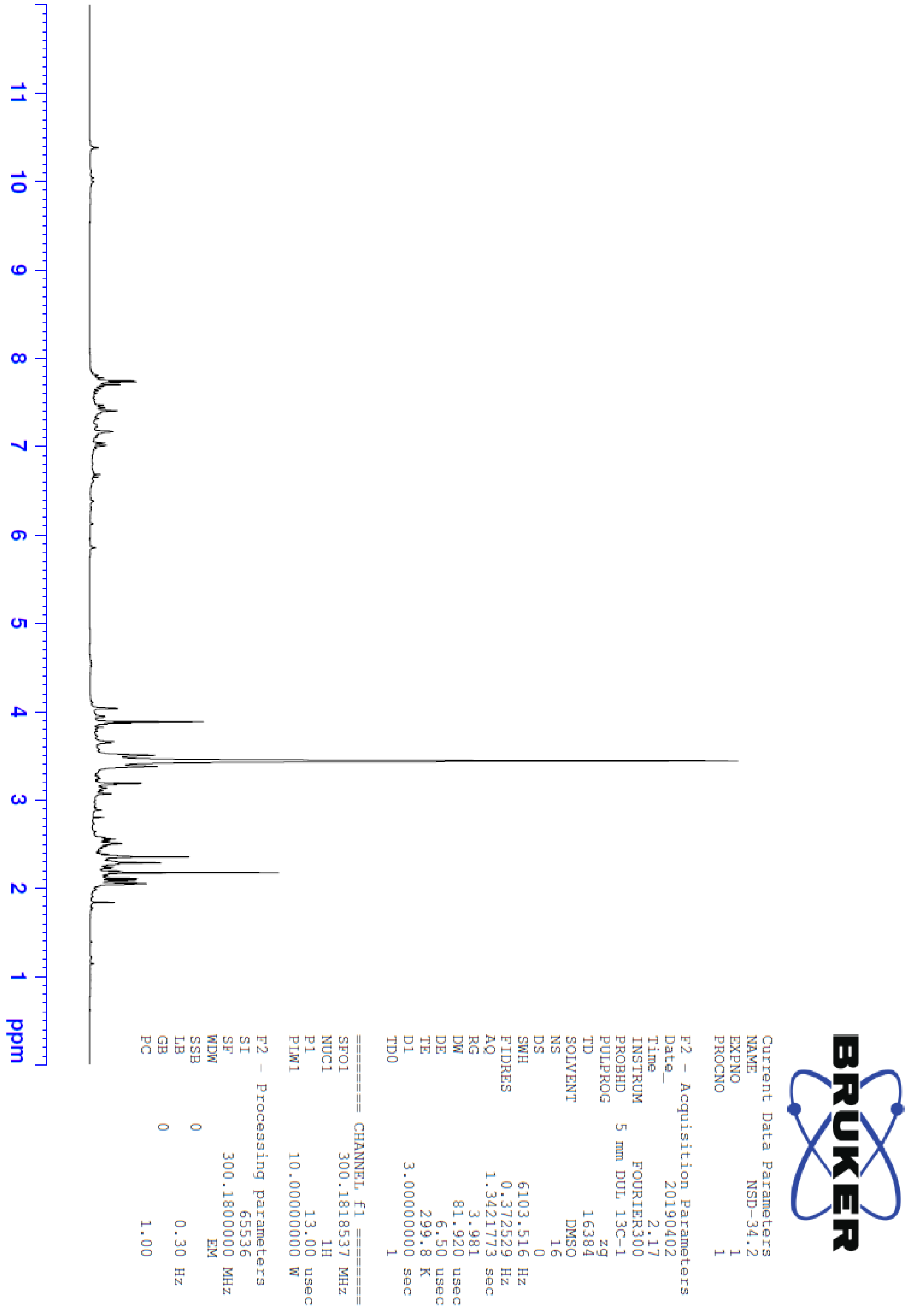

Figure S186. Compound **D34** ^{1}H-NMR spectrum.

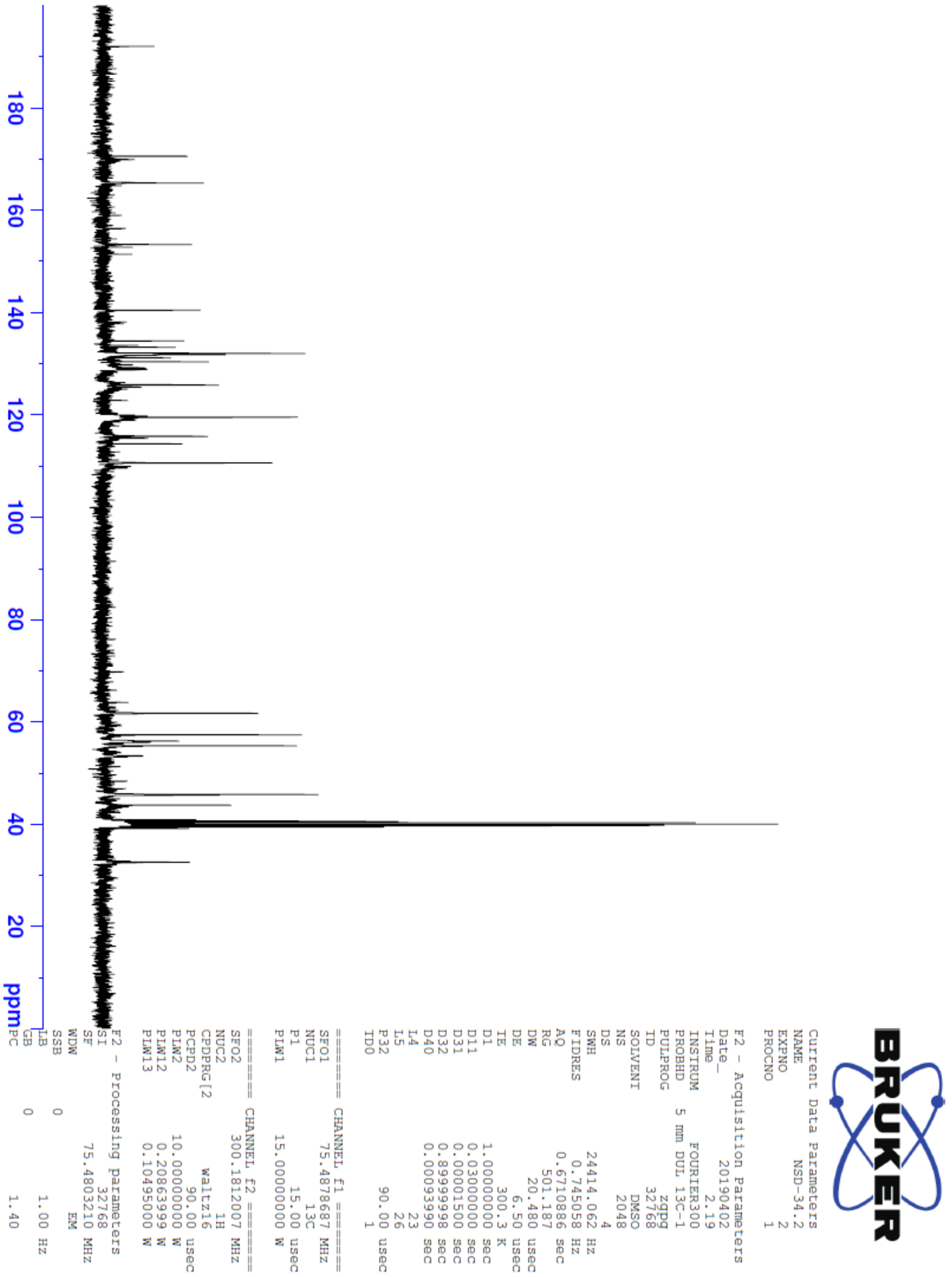

Figure S187. Compound **D34** ^{13}C-NMR spectrum.

Data File: C:\LabSolutions\Data\Analiz\bns\NSD-34_9.lcd

Elmt	Val.	Min	Max	Elmt	Val.	Min	Max	Elmt	Val.	Min	Max	Elmt	Val.	Min	Max	Use Adduct
H	1	5	40	O	2	3	5	S	2	0	0	Ru	2	0	0	H
C	4	0	35	F	1	0	0	Cl	1	0	0	I	3	0	0	
N	3	2	6	P	3	0	0	Br	1	0	0					

Error Margin (ppm):	10	DBE Range:	10.0 - 17.0	Electron Ions:	both
HC Ratio:	unlimited	Apply N Rule:	yes	Use MSn Info:	yes
Max Isotopes:	3	Isotope RI (%):	1.00	Isotope Res:	9000
MSn Iso RI (%):	10.00	MSn Logic Mode:	AND	Max Results:	500

Event#: 1 MS(E+) Ret. Time : 5.200 -> 5.360 Scan# : 781 -> 805

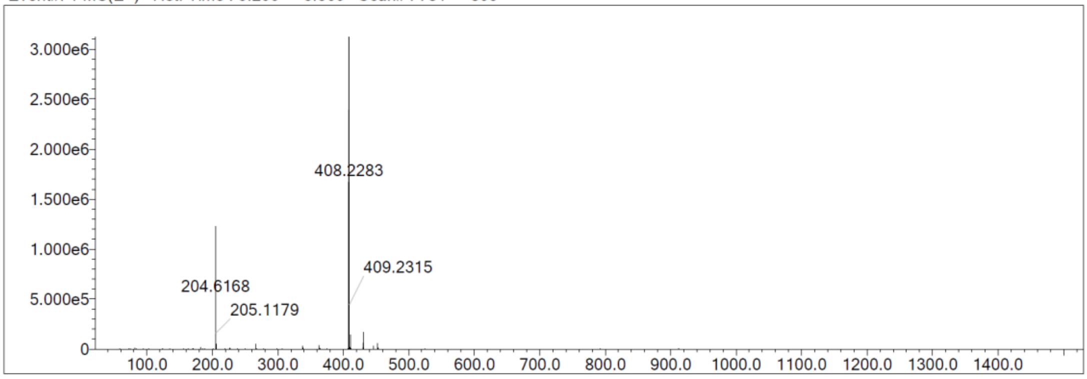

Measured region for 408.2283 m/z

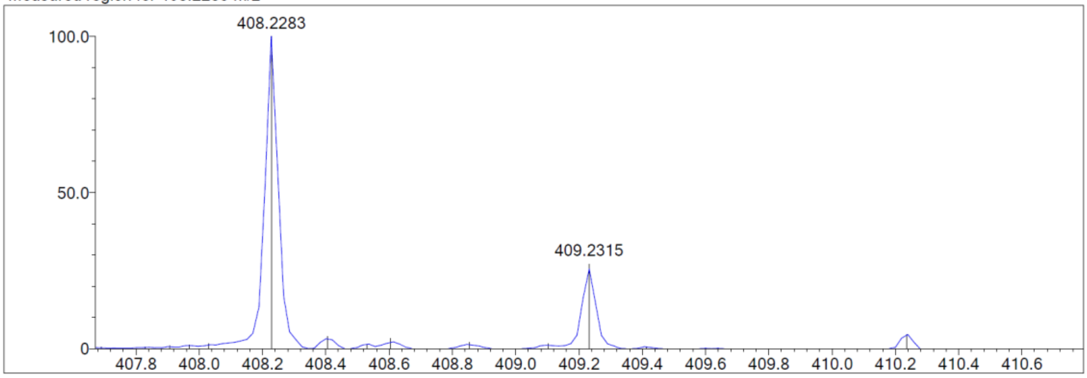

C24 H29 N3 O3 [M+H]+ : Predicted region for 408.2282 m/z

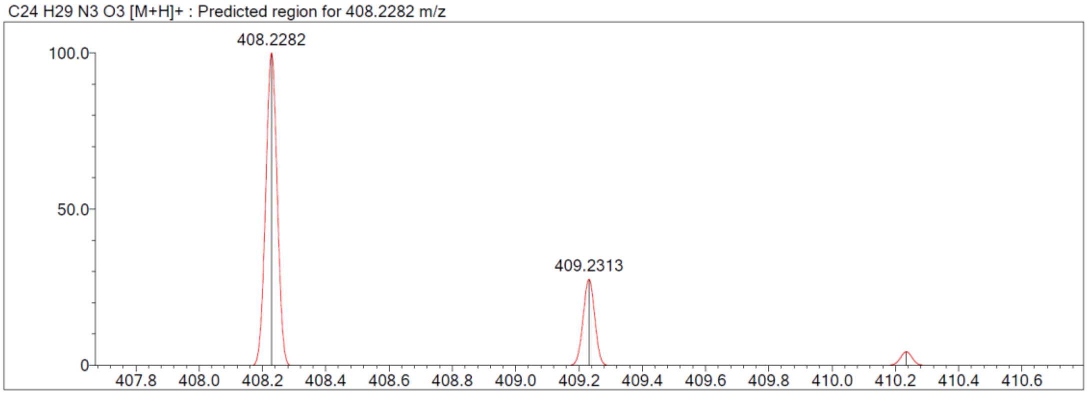

Rank	Score	Formula (M)	Ion	Meas. m/z	Pred. m/z	Df. (mDa)	Df. (ppm)	Iso	DBE
1	100.00	C24 H29 N3 O3	[M+H]+	408.2283	408.2282	0.1	0.24	100.00	12.0

Figure S188. Compound **D34** HRMS report.

*2-(N-methyl-N-(2-(dimethylamino)ethyl)amino)-N-(4-((6-methoxy-1-oxo-2,3-dihydro-1H-inden-2-ylidene)methyl)phenyl)acetamide (**D35**)*

Brown powder. M.P.: 107.2 °C. Yield: 83%.

IR (ATR) ν_{max} (cm^{-1}): 3255 (N-H), 1678 (indanone C=O), 1630 (amide C=O), 1591-1514 (C=C), 1165 (C-N), 1095 (C-O), 825 (1,4-disubstituted benzene).

^{1}H-NMR (300 MHz, DMSO-*d_6*) δ (ppm): 2.19 (6H, s, CH_3), 2.36 (3H, s, CH_3), 2.39 (2H, t, *J*=5.94 Hz, CH_2), 2.57 (2H, t, *J*=6.09 Hz, CH_2), 3.19 (2H, m, CH_2), 3.83 (3H, s, OCH_3), 4.00 (2H, s, CH_2), 7.23 (1H, *J*=2.52 Hz, methoxy-1-oxo-indenylidene CH), 7.28 (1H, dd, J_1=8.31 Hz, J_2=2.58 Hz, methoxy-1-oxo-indenylidene CH), 7.47 (1H, s, C=CH), 7.56 (1H, *J*=8.41 Hz, methoxy-1-oxo-indenylidene CH), 7.75 (4H, s, disubstituted benzene CH), 10.40 (1H, s, NH).

^{13}C-NMR (75 MHz, DMSO-*d_6*) δ (ppm): 31.7, 43.7, 45.7, 55.2, 56.0, 57.4, 61.6, 106.0, 119.5, 123.7, 127.9, 130.2, 132.3, 133.0, 134.6, 139.1, 140.7, 142.9, 159.6, 170.6, 193.6.

HRMS (ESI) (m/z) $[M+H]^+$: $C_{24}H_{29}N_3O_3$ calculated: 408.2282, found: 408.2288.

DOPNALAB

	Item	Value
	Acquired Date&Time	22.08.2019 13:25:56
	Acquired by	System Administrator
	Filename	C:\Users\dopnalab\Desktop\NURPELİN\DOKTORA TEZ\D351.ispd
	Spectrum name	D351
	Sample name	D35
	Sample ID	
	Option	
	Comment	
	No. of Scans	50
	Resolution	4 [cm-1]
	Apodization	Happ-Genzel

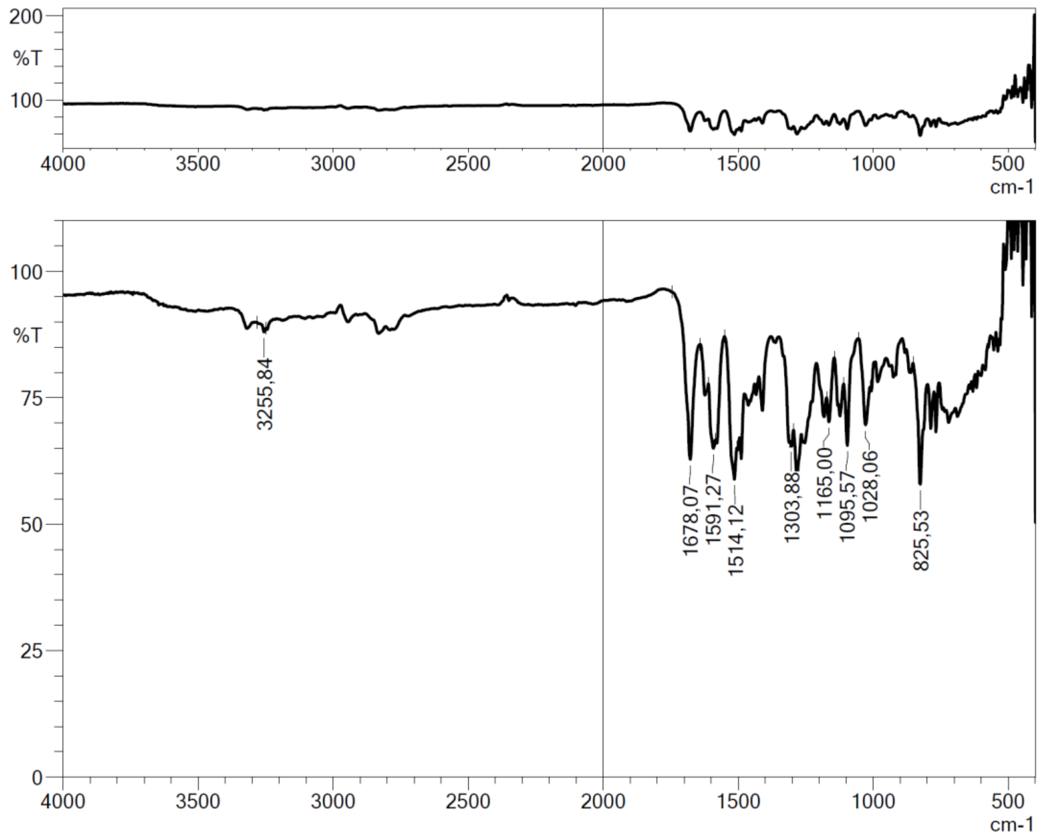

Figure S189. Compound **D35** IR report.

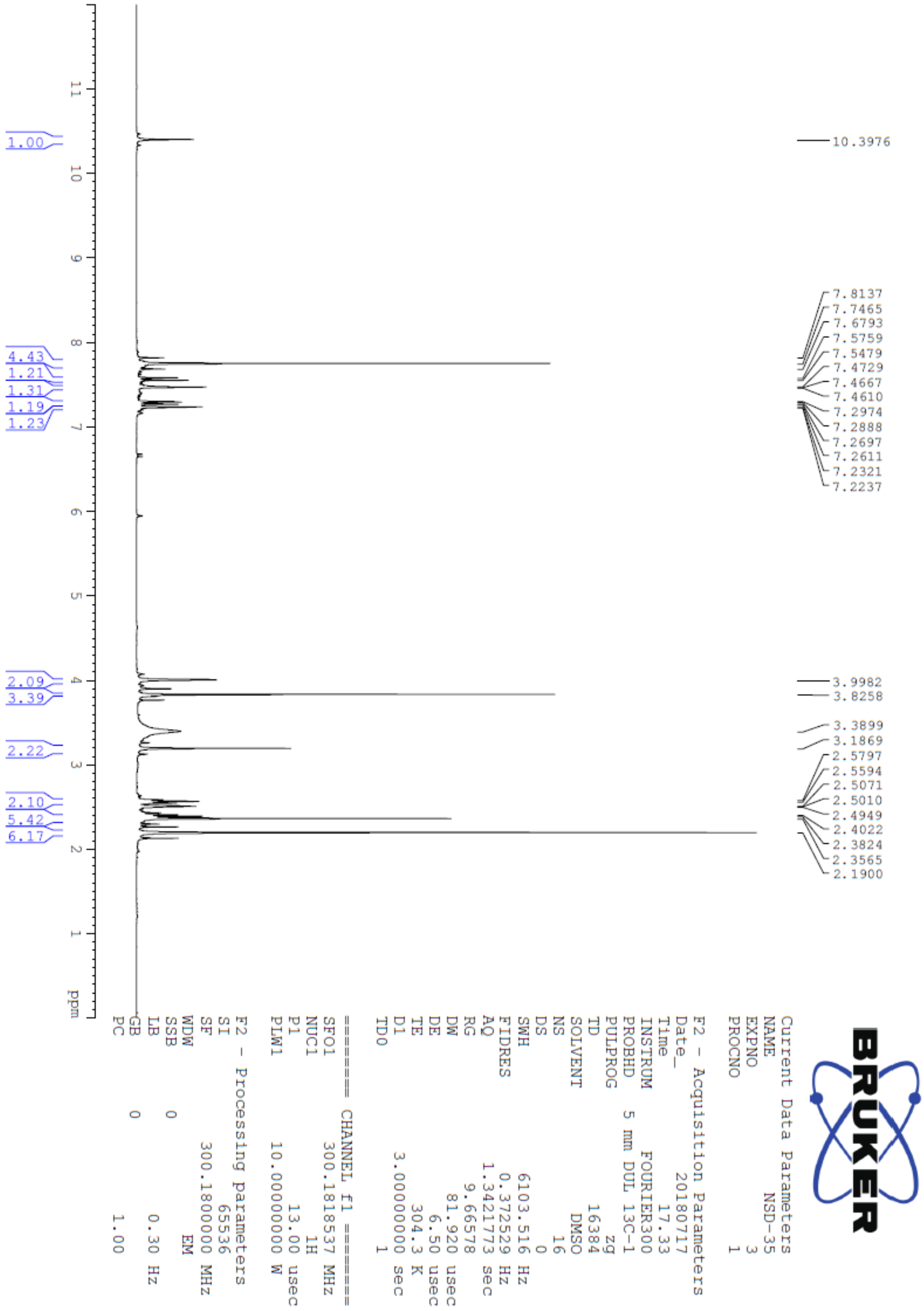

Figure S190. Compound **D35** ^{1}H-NMR spectrum.

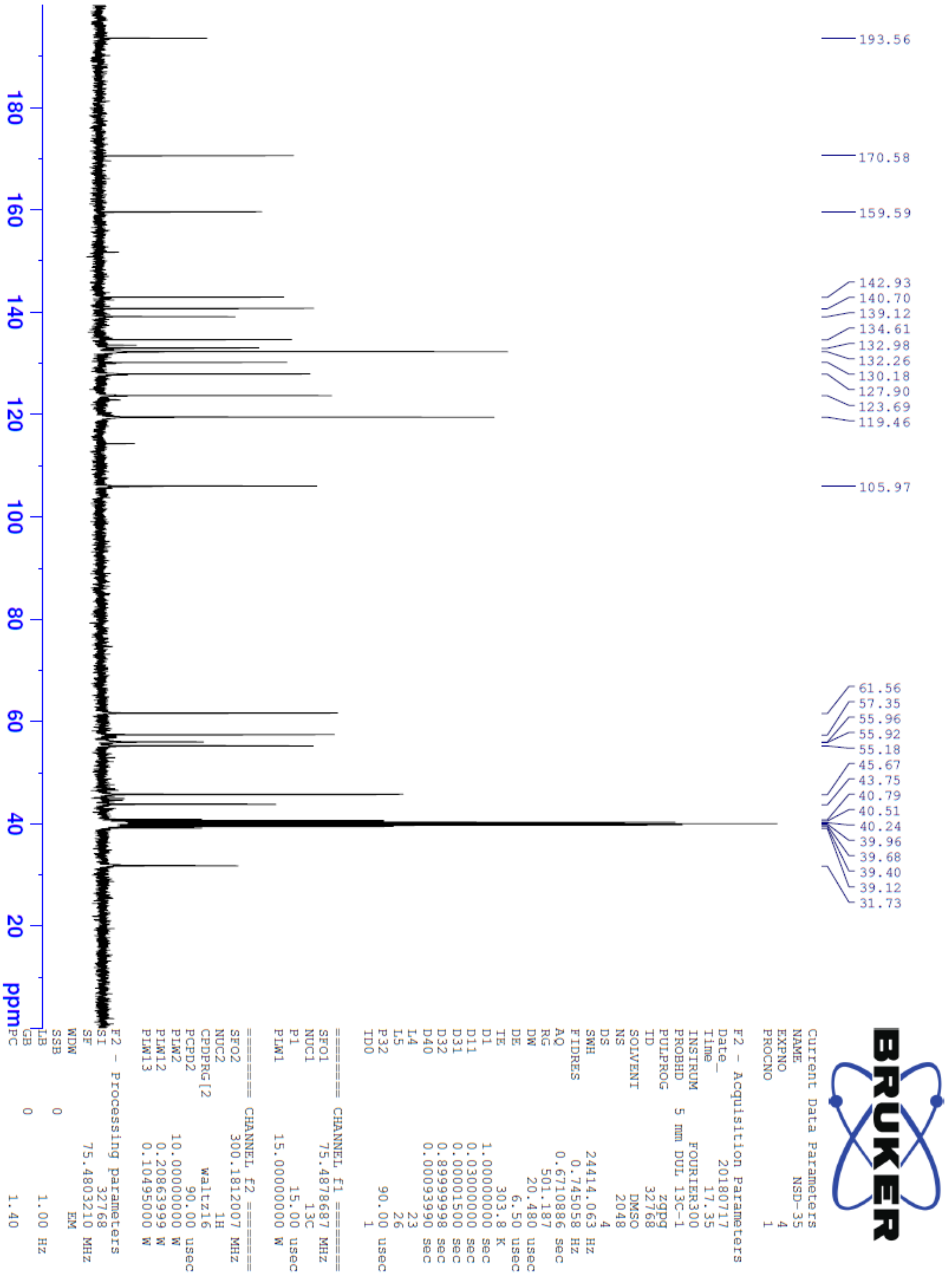

Figure S191. Compound **D35** ^{13}C-NMR spectrum.

Data File: C:\LabSolutions\Data\Analiz\bns\NSD-35_10.lcd

Elmt	Val.	Min	Max	Elmt	Val.	Min	Max	Elmt	Val.	Min	Max	Elmt	Val.	Min	Max	Use Adduct
H	1	5	40	O	2	3	5	S	2	0	0	Ru	2	0	0	H
C	4	0	35	F	1	0	0	Cl	1	0	0	I	3	0	0	
N	3	2	6	P	3	0	0	Br	1	0	0					

Error Margin (ppm): 10
HC Ratio: unlimited
Max Isotopes: 3
MSn Iso RI (%): 10.00

DBE Range: 10.0 - 17.0
Apply N Rule: yes
Isotope RI (%): 1.00
MSn Logic Mode: AND

Electron Ions: both
Use MSn Info: yes
Isotope Res: 9000
Max Results: 500

Event#: 1 MS(E+) Ret. Time : 5.307 -> 5.520 Scan# : 797 -> 829

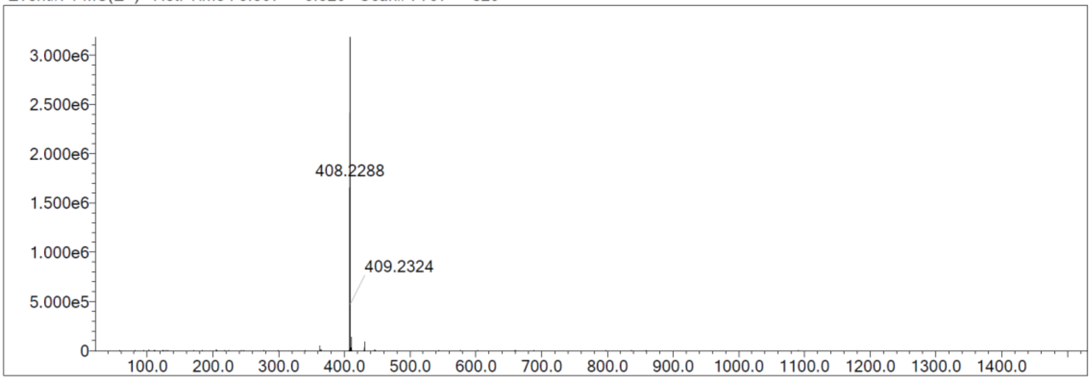

Measured region for 408.2288 m/z

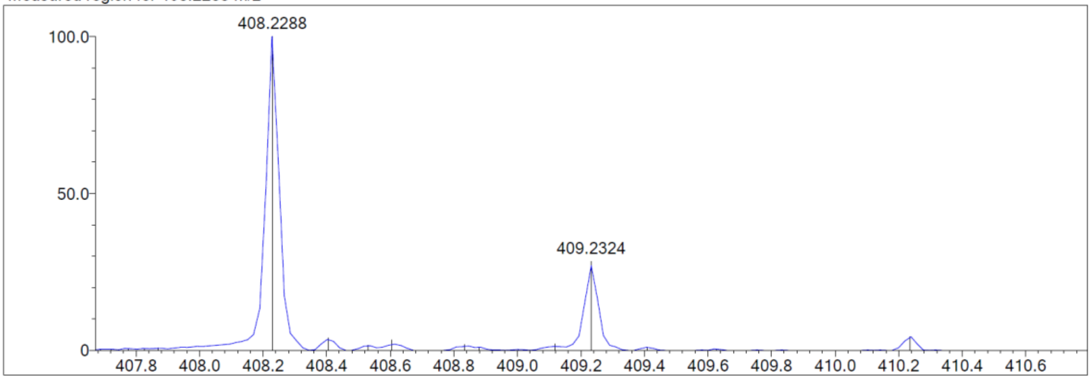

C24 H29 N3 O3 [M+H]+ : Predicted region for 408.2282 m/z

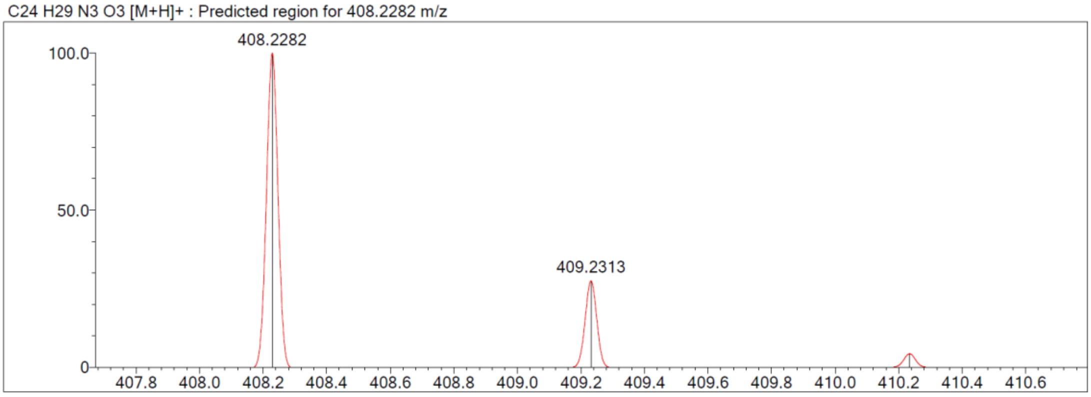

Rank	Score	Formula (M)	Ion	Meas. m/z	Pred. m/z	Df. (mDa)	Df. (ppm)	Iso	DBE
1	98.83	C24 H29 N3 O3	[M+H]+	408.2288	408.2282	0.6	1.47	100.00	12.0

Figure S192. Compound **D35** HRMS report.

*2-(N-methyl-N-(2-(dimethylamino)ethyl)amino)-N-(4-((5,6-dimethoxy-1-oxo-2,3-dihydro-1H-inden-2-ylidene))methyl)phenyl)acetamide (**D36**)*

Dark brown liquid. M.P.: 180.5 °C. Yield: 81%.

IR (ATR) ν_{max} (cm^{-1}): 3255 (N-H), 2829 (aliphatic C-H), 1678 (indanone C=O), 1630 (amide C=O), 1591-1514 (C=C), 1165 (C-N), 1095 (C-O), 825 (1,4-disubstituted benzene).

^{1}H-NMR (300 MHz, DMSO-d_6) δ (ppm): 2.21 (6H, s, CH_3), 2.36 (3H, s, CH_3), 2.42 (2H, t, *J*=6.04 Hz, CH_2), 2.57 (2H, t, *J*=6.03 Hz, CH_2), 3.19 (2H, m, CH_2), 3.83 (3H, s, OCH_3), 3.90 (3H, s, OCH_3), 3.98 (2H, s, CH_2), 7.20 (1H, s, methoxy-1-oxo-indenylidene CH), 7.21 (1H, s, methoxy-1-oxo-indenylidene CH), 7.38 (1H, s, C=CH), 7.71 (2H, d, *J*=9.03 Hz, disubstituted benzene CH), 7.75 (2H, d, *J*=8.95 Hz, disubstituted benzene CH), 10.40 (1H, s, NH).

^{13}C-NMR (75 MHz, DMSO-d_6) δ (ppm): 32.1, 43.7, 45.6, 55.1, 56.1, 56.4, 57.3, 61.6, 105.0, 108.5, 119.5, 130.4, 130.6, 131.3, 131.9, 134.7, 140.4, 145.4, 149.8, 155.6, 170.5, 192.3.

HRMS (ESI) (m/z) $[M+H]^+$: $C_{25}H_{31}N_3O_4$ calculated: 438.2387, found: 438.2398.

DOPNALAB

	Item	Value
	Acquired Date&Time	22.08.2019 13:28:12
	Acquired by	System Administrator
	Filename	C:\Users\dopnalab\Desktop\NURPELİN\DOKTORA TEZ\D361.ispd
	Spectrum name	D361
	Sample name	D36
	Sample ID	
	Option	
	Comment	
	No. of Scans	50
	Resolution	4 [cm-1]
	Apodization	Happ-Genzel

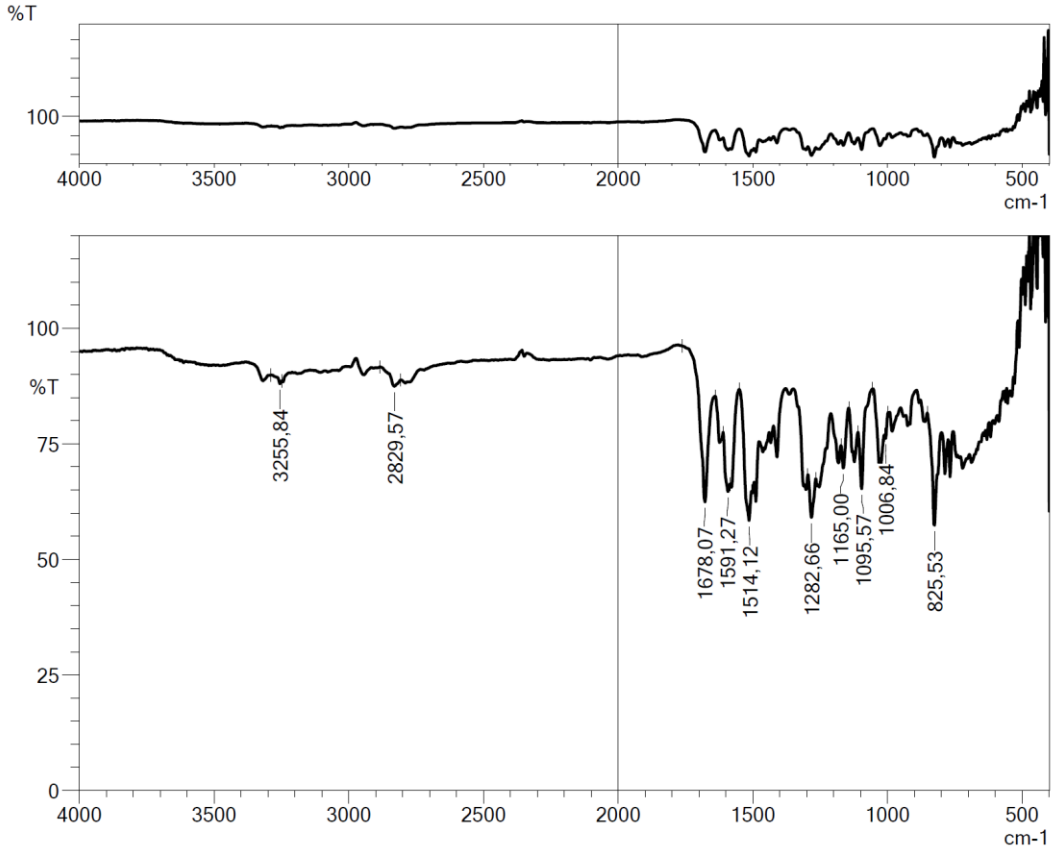

Figure S193. Compound **D36** IR report.

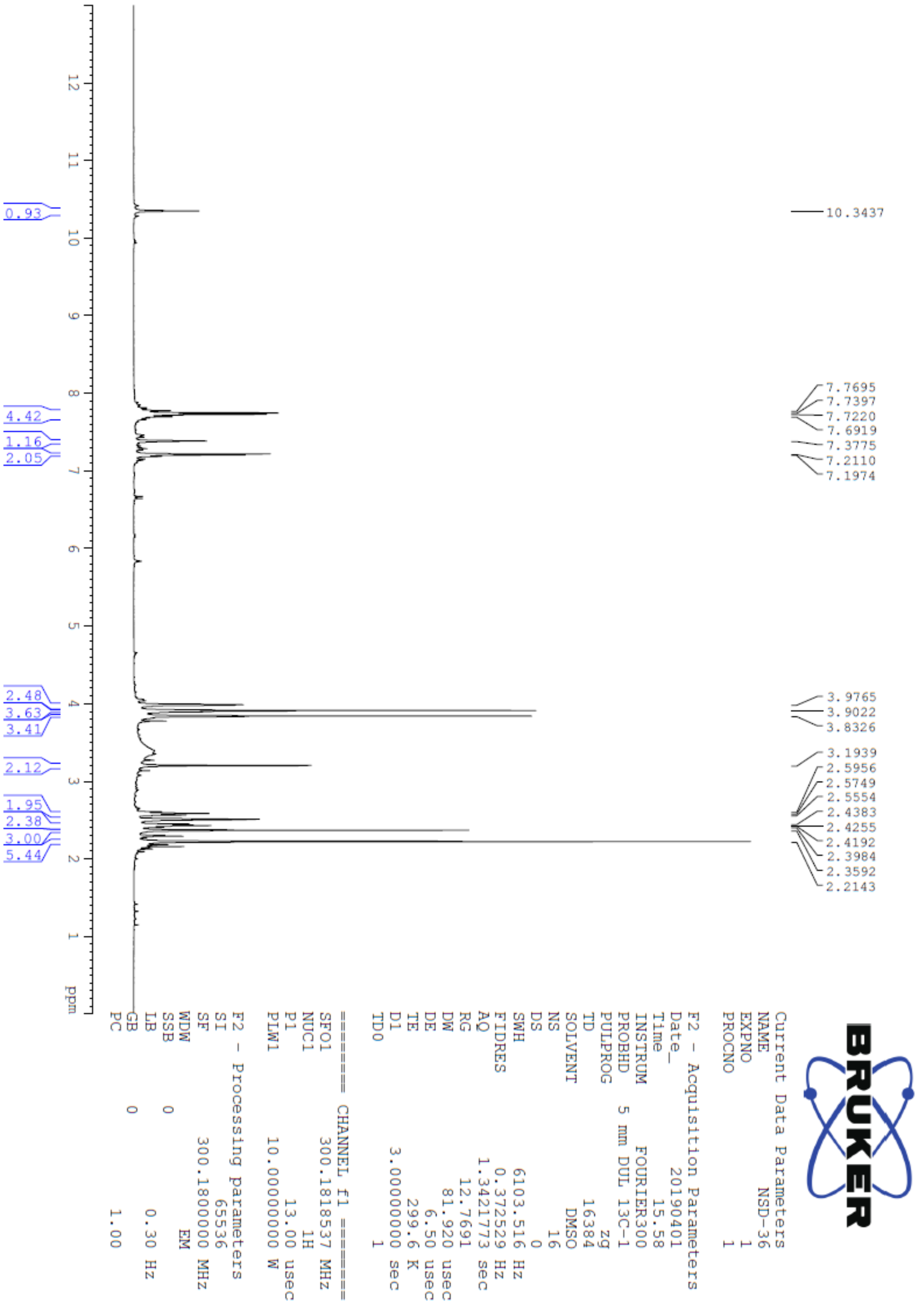

Figure S194. Compound **D36** ^{1}H-NMR spectrum.

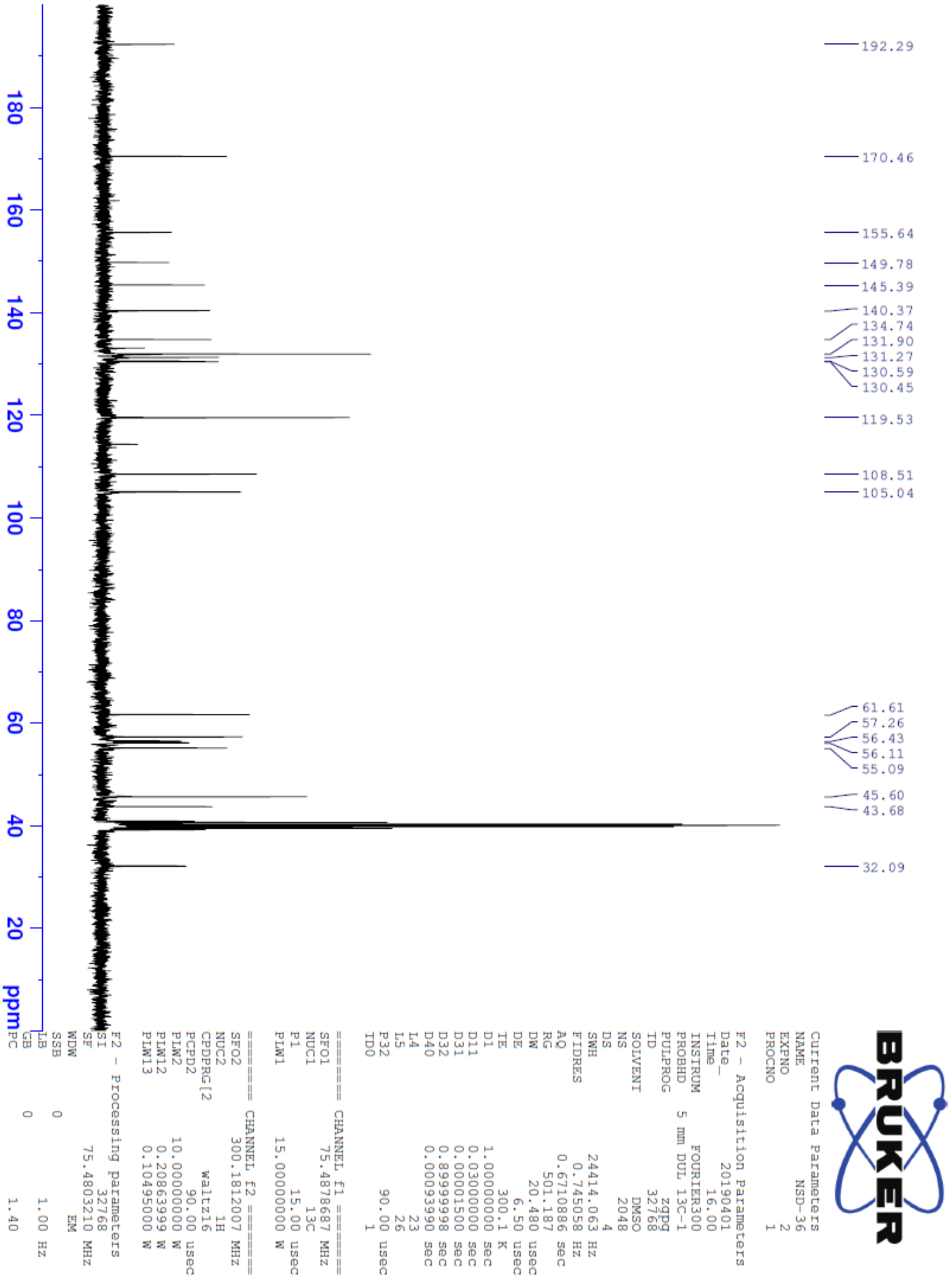

Figure S195. Compound **D36** ^{13}C-NMR spectrum.

Data File: C:\LabSolutions\Data\Analiz\bns\NSD-36_11.lcd

Elmt	Val.	Min	Max	Elmt	Val.	Min	Max	Elmt	Val.	Min	Max	Elmt	Val.	Min	Max	Use Adduct
H	1	5	40	O	2	3	5	S	2	0	0	Ru	2	0	0	H
C	4	0	35	F	1	0	0	Cl	1	0	0	I	3	0	0	
N	3	2	6	P	3	0	0	Br	1	0	0					

Error Margin (ppm): 10
HC Ratio: unlimited
Max Isotopes: 3
MSn Iso RI (%): 10.00

DBE Range: 10.0 - 17.0
Apply N Rule: yes
Isotope RI (%): 1.00
MSn Logic Mode: AND

Electron Ions: both
Use MSn Info: yes
Isotope Res: 9000
Max Results: 500

Event#: 1 MS(E+) Ret. Time : 4.880 -> 5.040 Scan# : 733 -> 757

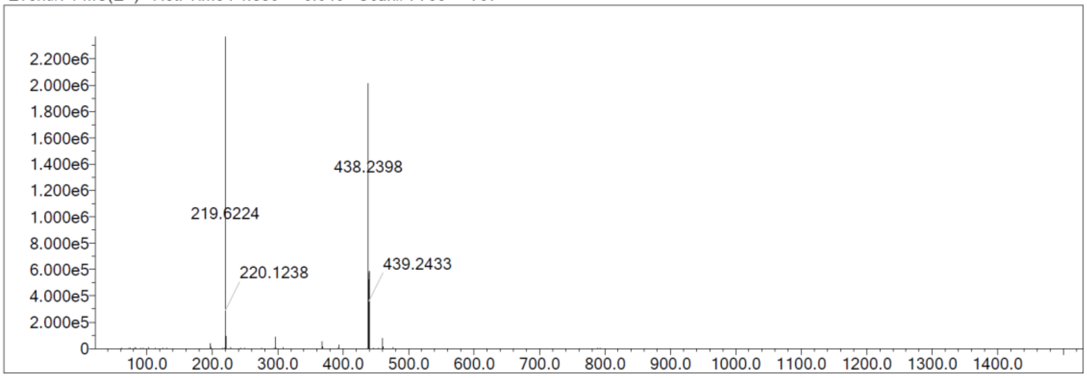

Measured region for 438.2398 m/z

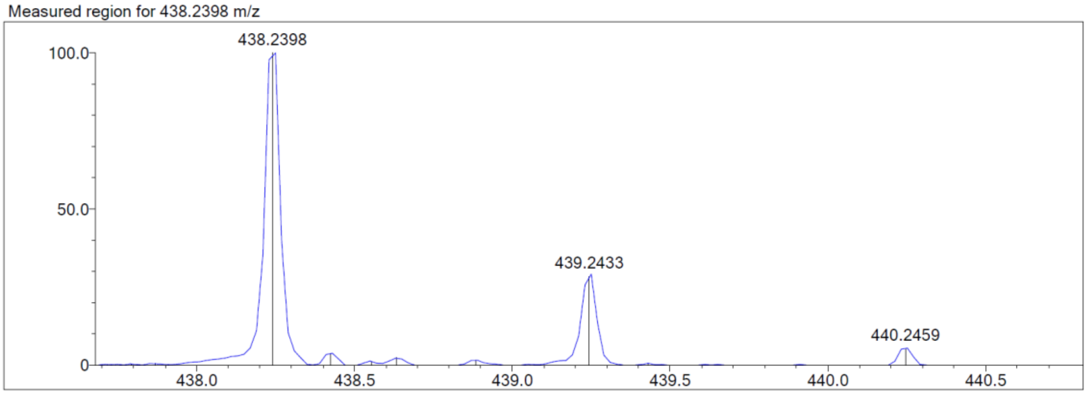

C25 H31 N3 O4 [M+H]+ : Predicted region for 438.2387 m/z

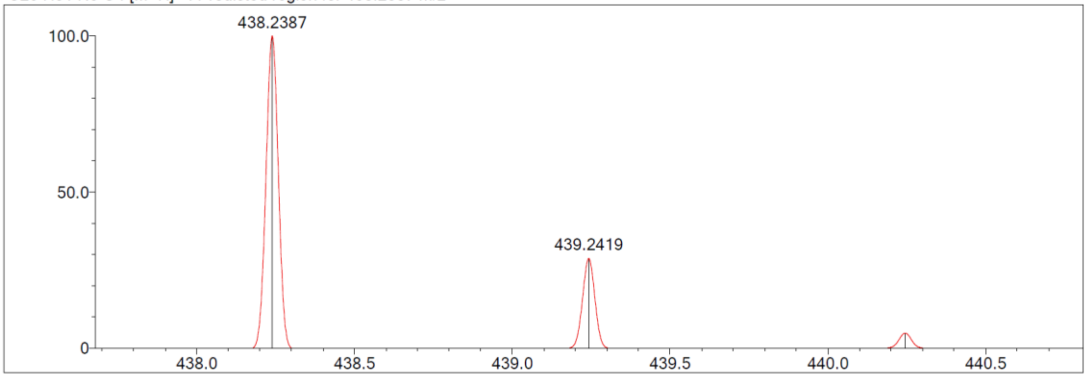

Rank	Score	Formula (M)	Ion	Meas. m/z	Pred. m/z	Df. (mDa)	Df. (ppm)	Iso	DBE
1	96.22	C25 H31 N3 O4	[M+H]+	438.2398	438.2387	1.1	2.51	100.00	12.0

Figure S196. Compound **D36** HRMS report.

*2-(N-methyl-N-(3-(dimethylamino)propyl)amino)-N-(4-((5-methoxy-1-oxo-2,3-dihydro-1H-inden-2-ylidene)methyl)phenyl)acetamide (**D37**)*

Brown powder. M.P.: 168.7 °C. Yield: 89%.

IR (ATR) ν_{max} (cm^{-1}): 3317 (N-H), 2943-2789 (aliphatic C-H), 1678 (indanone C=O), 1597 (amide C=O), 1579-1489 (C=C), 1251 (C-N), 1087 (C-O), 821 (1,4-disubstituted benzene).

^{1}H-NMR (300 MHz, DMSO-*d$_6$*) δ (ppm): 1.59 (2H, p, *J*=7.16 Hz, CH_3), 2.09 (6H, s, CH_3), 2.22 (2H, t, *J*=7.07 Hz, CH_2), 2.30 (3H, s, CH_3), 2.45 (2H, t, *J*=7.32 Hz, CH_2), 3.16 (2H, s, CH_2), 3.89 (3H, s, OCH_3), 4.05 (2H, s, CH_2), 7.02 (1H, dd, J_1=8.49 Hz, J_2=2.25 Hz, methoxy-1-oxo-indenylidene CH), 7.18 (1H, *J*=2.07 Hz, methoxy-1-oxo-indenylidene CH), 7.40 (1H, s, C=CH), 7.70-7.73 (3H, m, disubstituted benzene CH, methoxy-1-oxo-indenylidene CH), 7.78 (2H, d, *J*=8.79 Hz, disubstituted benzene CH), 9.91 (1H, s, NH).

^{13}C-NMR (75 MHz, DMSO-*d$_6$*) δ (ppm): 25.2, 32.5, 43.0, 45.7, 55.6, 56.3, 57.6, 62.1, 110.6, 115.8, 120.0, 125.8, 130.5, 131.1, 131.7, 131.9, 134.5, 140.3, 153.3, 165.3, 170.0, 192.1.

HRMS (ESI) (m/z) [M+H]$^+$: $C_{25}H_{31}N_3O_3$ calculated: 422.2438, found: 422.2458.

DOPNALAB

Item	Value
Acquired Date&Time	22.08.2019 13:30:47
Acquired by	System Administrator
Filename	C:\Users\dopnalab\Desktop\NURPELİN\DOKTORA TEZ\D371.ispd
Spectrum name	D371
Sample name	D37
Sample ID	
Option	
Comment	
No. of Scans	50
Resolution	4 [cm-1]
Apodization	Happ-Genzel

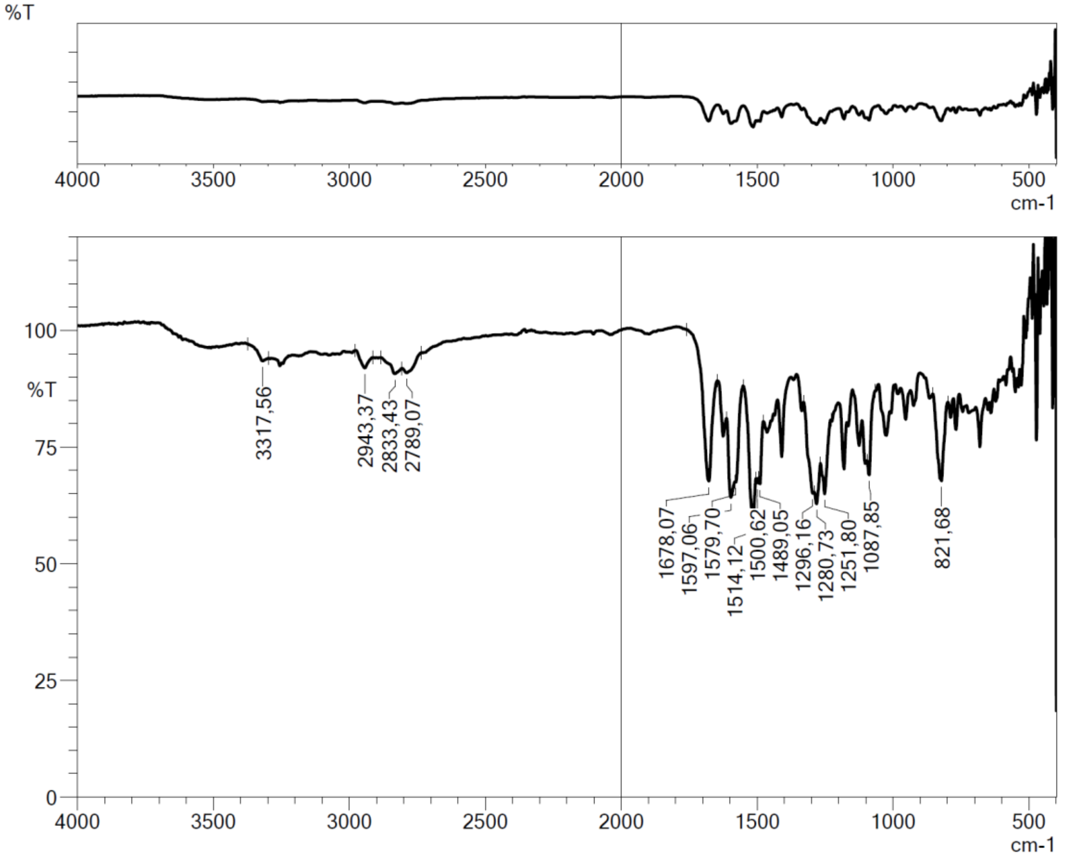

Figure S197. Compound **D37** IR report.

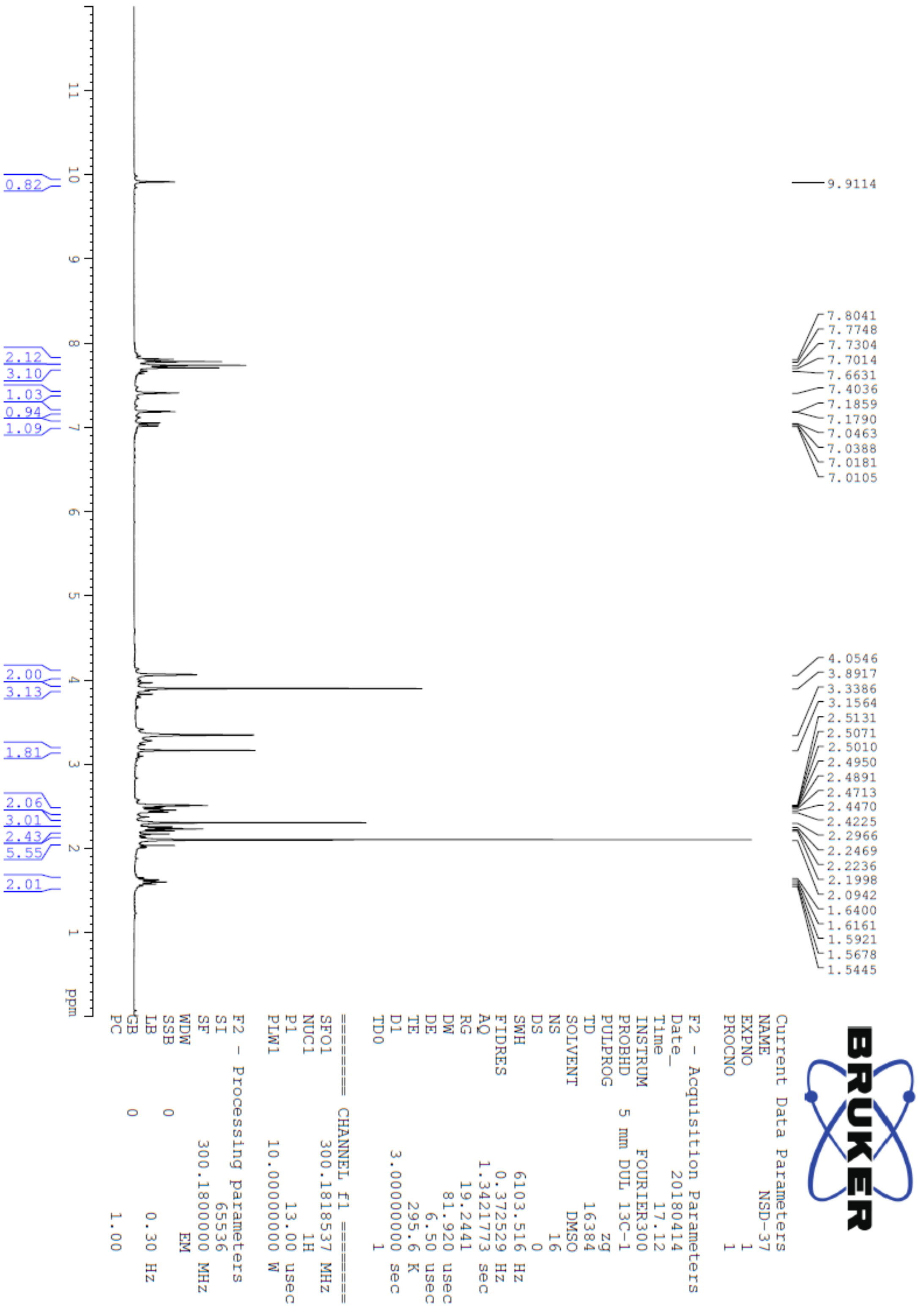

Figure S198. Compound **D37** ^{1}H-NMR spectrum.

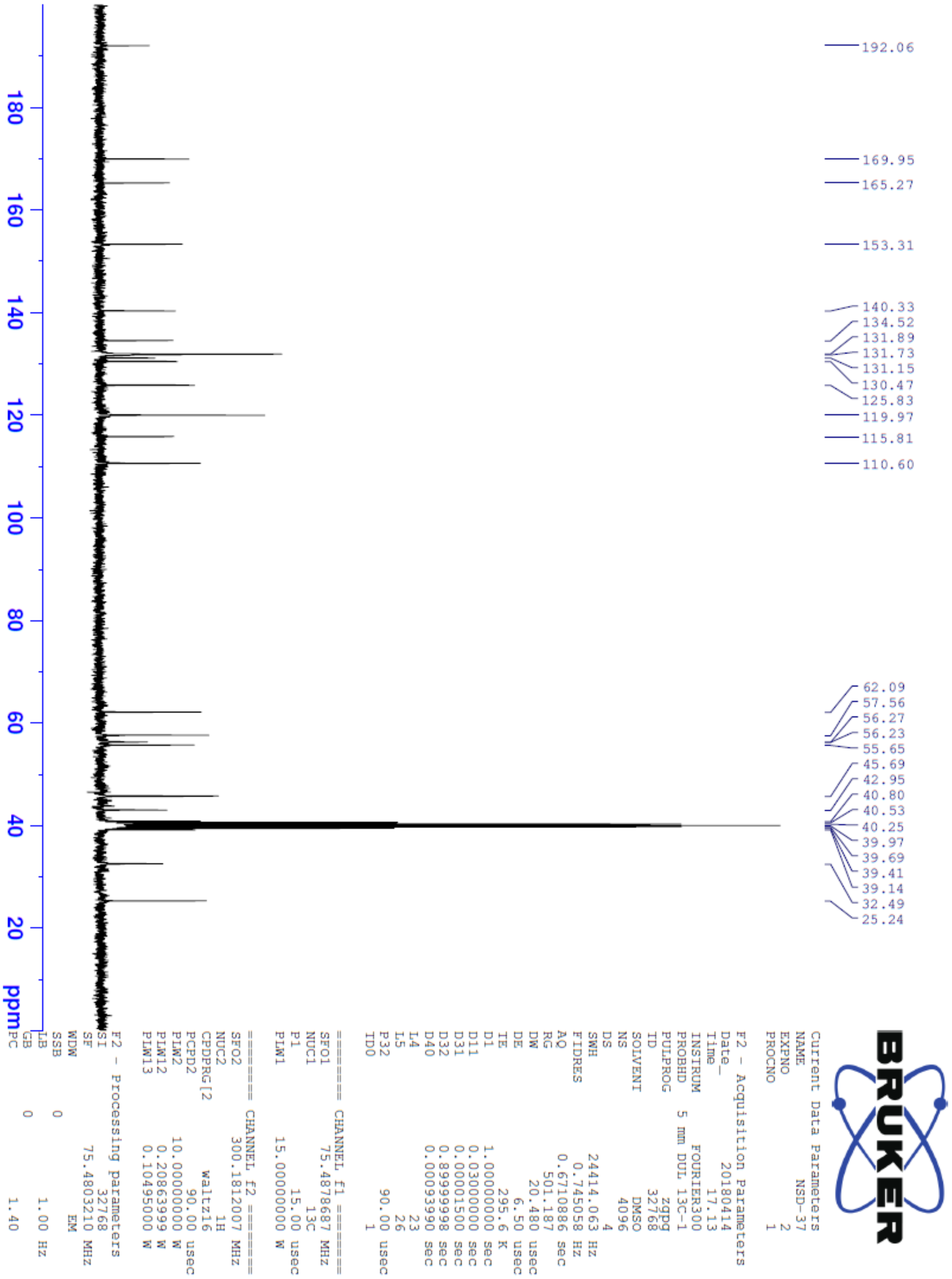

Figure S199. Compound **D37** ^{13}C-NMR spectrum.

Data File: C:\LabSolutions\Data\Analiz\Serkan\NSD-37_74.lcd

Elmt	Val.	Min	Max	Elmt	Val.	Min	Max	Elmt	Val.	Min	Max	Elmt	Val.	Min	Max	Use Adduct
H	1	5	40	O	2	3	5	S	2	0	0	Ru	2	0	0	H
C	4	0	35	F	1	0	0	Cl	1	0	0	I	3	0	0	
N	3	2	6	P	3	0	0	Br	1	0	0					

Error Margin (ppm):	10	DBE Range:	10.0 - 17.0	Electron Ions:	both
HC Ratio:	unlimited	Apply N Rule:	yes	Use MSn Info:	yes
Max Isotopes:	3	Isotope RI (%):	1.00	Isotope Res:	9000
MSn Iso RI (%):	10.00	MSn Logic Mode:	AND	Max Results:	500

Event#: 1 MS(E+) Ret. Time : 1.320 -> 1.653 Scan# : 199 -> 249

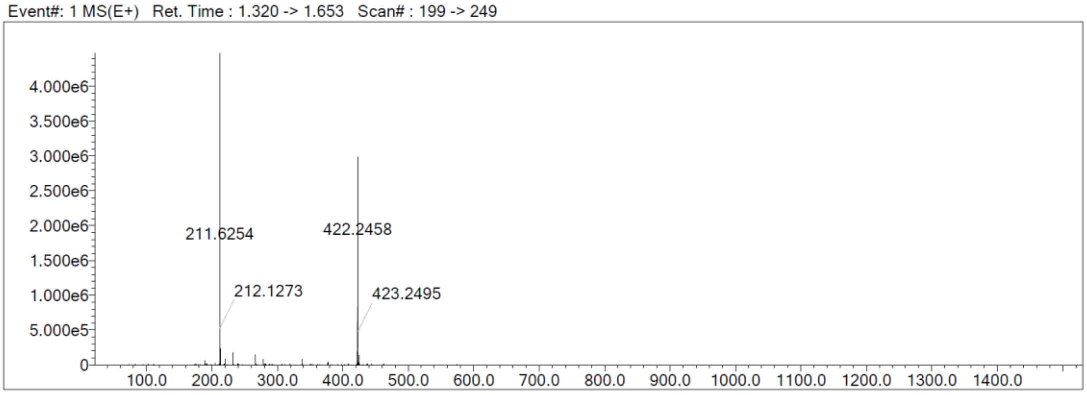

Measured region for 422.2458 m/z

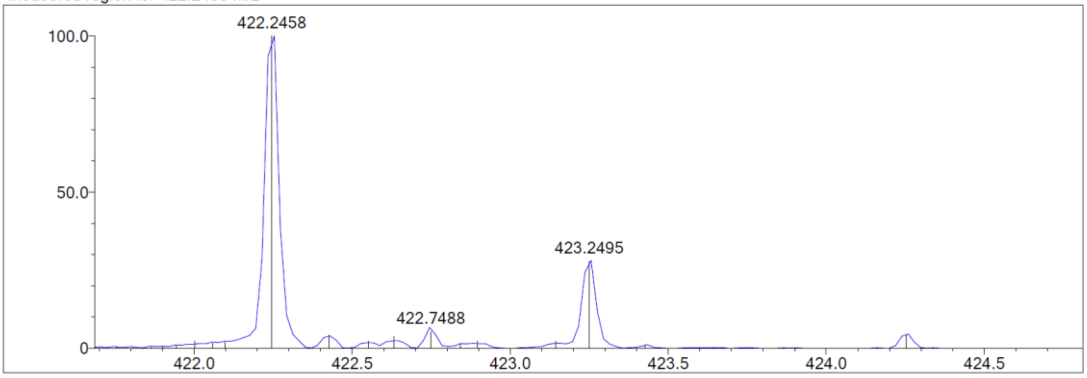

C25 H31 N3 O3 [M+H]+ : Predicted region for 422.2438 m/z

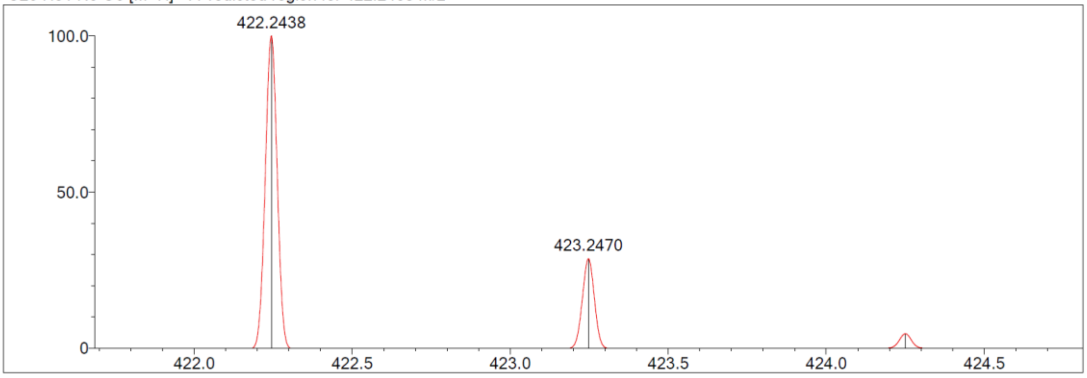

Rank	Score	Formula (M)	Ion	Meas. m/z	Pred. m/z	Df. (mDa)	Df. (ppm)	Iso	DBE
1	90.65	C25 H31 N3 O3	[M+H]+	422.2458	422.2438	2.0	4.74	100.00	12.0

Figure S200. Compound **D37** HRMS report.

*2-(N-methyl-N-(3-(dimethylamino)propyl)amino)-N-(4-((6-methoxy-1-oxo-2,3-dihydro-1H-inden-2-ylidene)methyl)phenyl)acetamide (**D38**)*

Dark brown powder. M.P.: 125.5 °C. Yield: 75%.

IR (ATR) ν_{max} (cm^{-1}): 3319 (N-H), 2943-2789 (aliphatic C-H), 1678 (indanone C=O), 1597 (amide C=O), 1514-1409 (C=C), 1251 (C-N), 1087 (C-O), 821 (1,4-disubstituted benzene).

^{1}H-NMR (300 MHz, DMSO-*d*$_6$) δ (ppm): 1.59 (2H, p, *J*=7.22 Hz, CH_3), 2.10 (6H, s, CH_3), 2.22 (2H, t, *J*=7.08 Hz, CH_2), 2.30 (3H, s, CH_3), 2.45 (2H, t, *J*=7.28 Hz, CH_2), 3.16 (2H, s, CH_2), 3.84 (3H, s, OCH_3), 4.02 (2H, s, CH_2), 7.24 (1H, *J*=2.31 Hz, methoxy-1-oxo-indenylidene CH), 7.29 (1H, dd, J_1=8.31 Hz, J_2=2.55 Hz, methoxy-1-oxo-indenylidene CH), 7.48 (1H, s, C=CH), 7.58 (1H, *J*=8.35 Hz, methoxy-1-oxo-indenylidene CH), 7.74 (2H, d *J*=9.00 Hz, disubstituted benzene CH), 7.79 (2H, d, *J*=8.89 Hz, disubstituted benzene CH), 9.92 (1H, s, NH).

^{13}C-NMR (75 MHz, DMSO-*d*$_6$) δ (ppm): 25.3, 31.7, 43.0, 45.7, 55.7, 56.0, 57.6, 62.1, 106.0, 120.0, 123.7, 127.9, 130.3, 132.1, 133.0, 134.7, 139.1, 140.6, 143.0, 159.6, 170.0, 193.6.

HRMS (ESI) (m/z) [M+H]$^+$: $C_{25}H_{31}N_3O_3$ calculated: 422.2438, found: 422.2449.

DOPNALAB

	Item	Value
	Acquired Date&Time	22.08.2019 13:32:47
	Acquired by	System Administrator
	Filename	C:\Users\dopnalab\Desktop\NURPELİN\DOKTORA TEZ\D381.ispd
	Spectrum name	D381
	Sample name	D38
	Sample ID	
	Option	
	Comment	
	No. of Scans	50
	Resolution	4 [cm-1]
	Apodization	Happ-Genzel

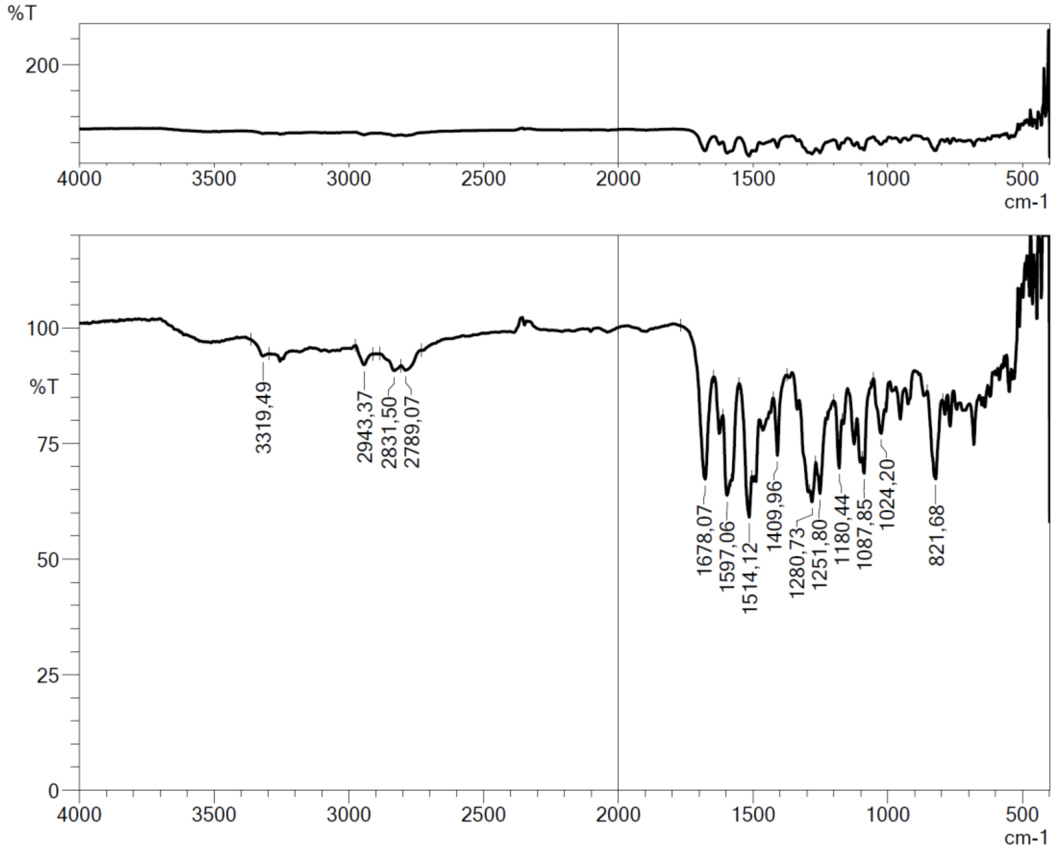

Figure S201. Compound **D38** IR report.

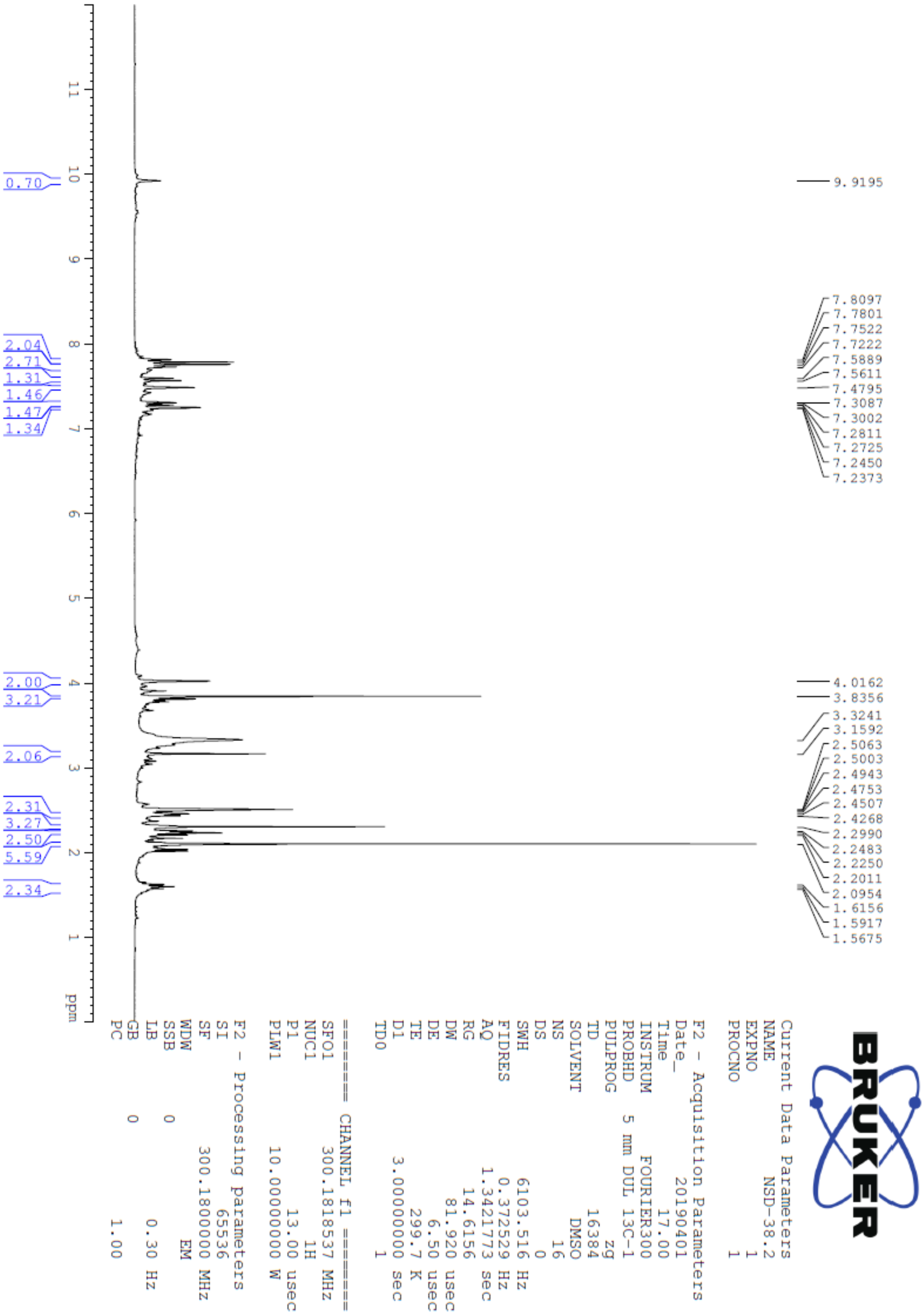

Figure S202. Compound **D38** ^{1}H-NMR spectrum.

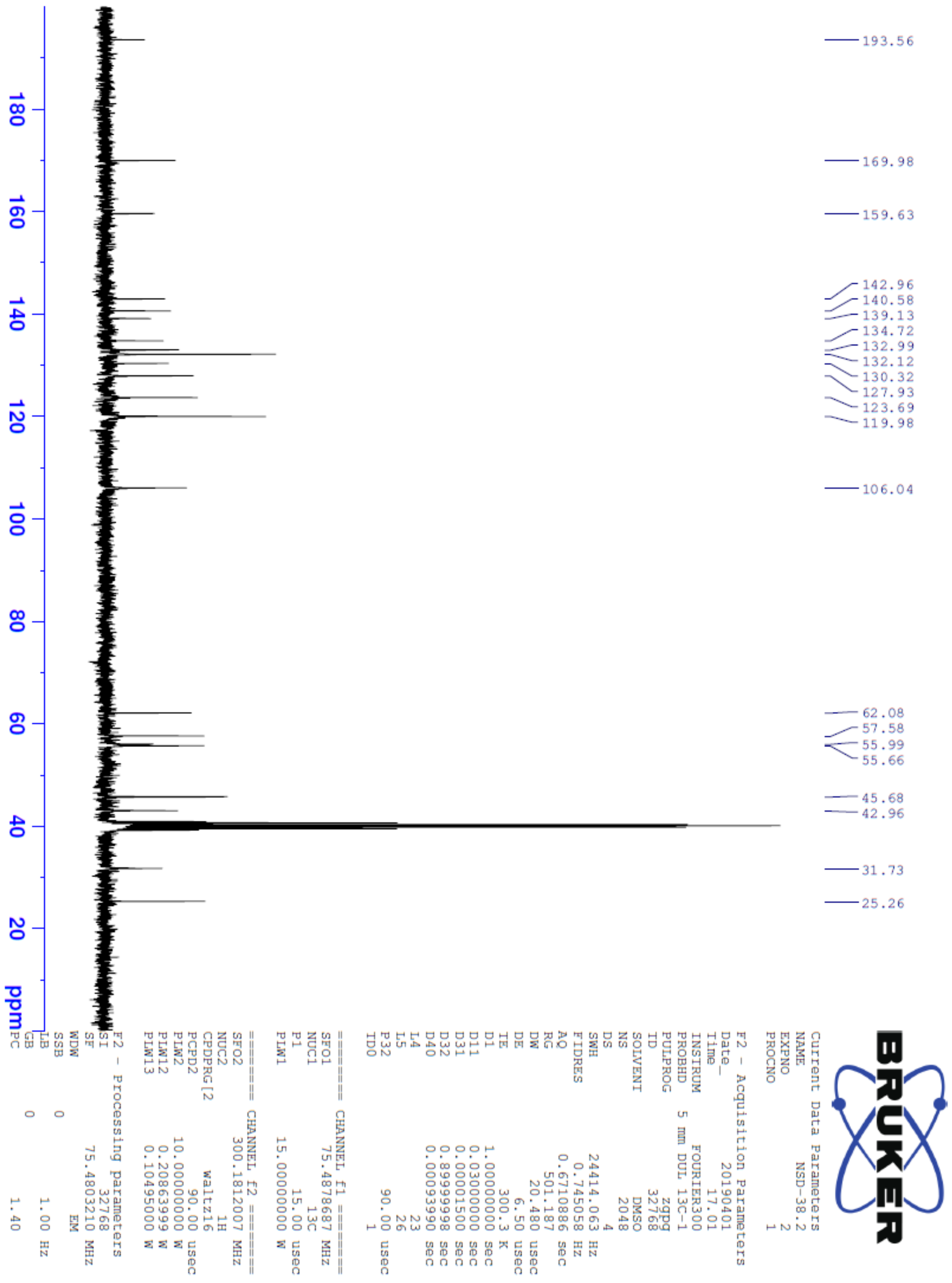

Figure S203. Compound **D38** ^{13}C-NMR spectrum.

Data File: C:\LabSolutions\Data\Analiz\bns\NSD-38_12.lcd

Elmt	Val.	Min	Max	Elmt	Val.	Min	Max	Elmt	Val.	Min	Max	Elmt	Val.	Min	Max	Use Adduct
H	1	5	40	O	2	3	5	S	2	0	0	Ru	2	0	0	H
C	4	0	35	F	1	0	0	Cl	1	0	0	I	3	0	0	
N	3	2	6	P	3	0	0	Br	1	0	0					

Error Margin (ppm): 10
HC Ratio: unlimited
Max Isotopes: 3
MSn Iso RI (%): 10.00

DBE Range: 10.0 - 17.0
Apply N Rule: yes
Isotope RI (%): 1.00
MSn Logic Mode: AND

Electron Ions: both
Use MSn Info: yes
Isotope Res: 9000
Max Results: 500

Event#: 1 MS(E+) Ret. Time : 4.653 -> 4.707 Scan# : 699 -> 707

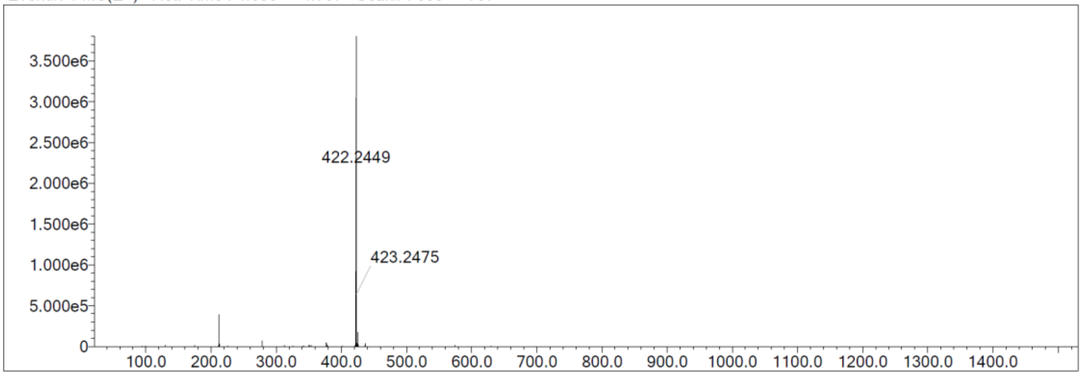

Measured region for 422.2449 m/z

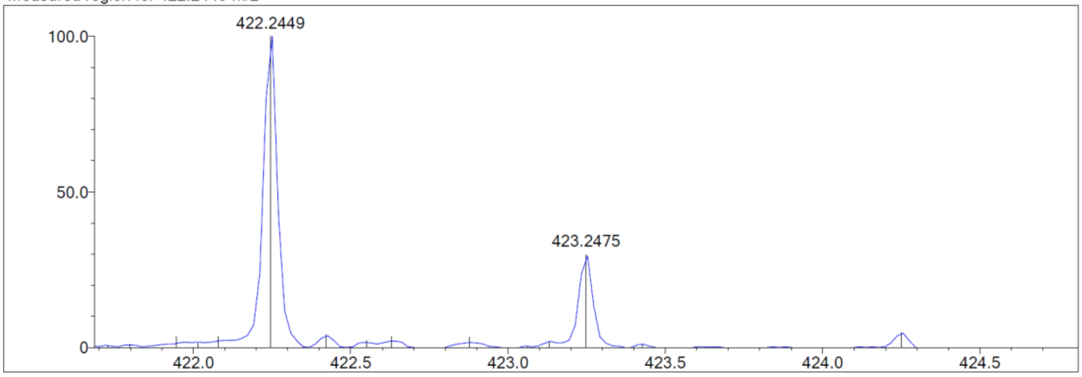

C25 H31 N3 O3 [M+H]+ : Predicted region for 422.2438 m/z

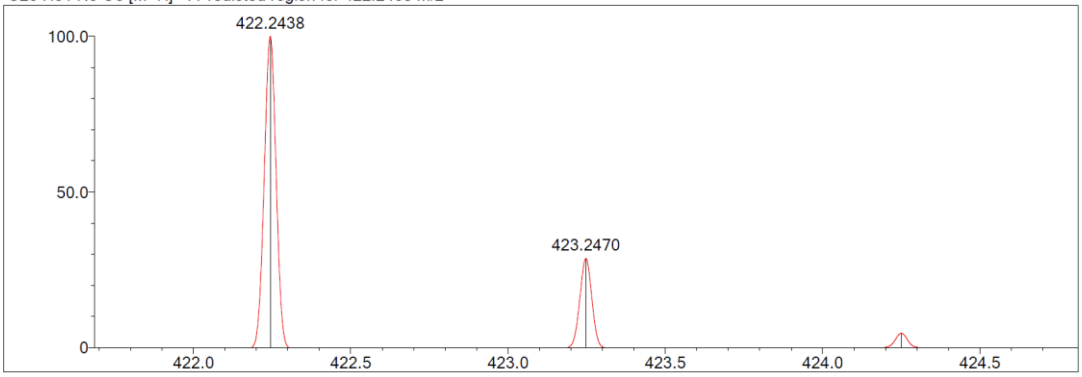

Rank	Score	Formula (M)	Ion	Meas. m/z	Pred. m/z	Df. (mDa)	Df. (ppm)	Iso	DBE
1	85.28	C25 H31 N3 O3	[M+H]+	422.2449	422.2438	1.1	2.61	88.86	12.0

Figure S204. Compound **D38** HRMS report.

*2-(N-methyl-N-(3-(dimethylamino)propyl)amino)-N-(4-((5,6-dimethoxy-1-oxo-2,3-dihydro-1H-inden-2-ylidene))methyl)phenyl)acetamide (**D39**)*

Dark brown powder. M.P.: 121.6 °C. Yield: 88%.

IR (ATR) ν_{max} (cm^{-1}): 3319 (N-H), 1678 (indanone C=O), 1625 (amide C=O), 1585-1498 (C=C), 1222 (C-N), 1091 (C-O), 829 (1,4-disubstituted benzene).

^{1}H-NMR (300 MHz, DMSO-d_6) δ (ppm): 1.59 (2H, p, *J*=7.22 Hz, CH_3), 2.09 (6H, s, CH_3), 2.22 (2H, t, *J*=7.08 Hz, CH_2), 2.30 (3H, s, CH_3), 2.45 (2H, t, *J*=7.28 Hz, CH_2), 3.16 (2H, s, CH_2), 3.84 (3H, s, OCH_3), 3.90 (3H, s, OCH_3), 3.97 (2H, s, CH_2), 7.20 (1H, s, methoxy-1-oxo-indenylidene CH), 7.21 (1H, s, methoxy-1-oxo-indenylidene CH), 7.38 (1H, s, C=CH), 7.70 (2H, d, *J*=8.82 Hz, disubstituted benzene CH), 7.78 (2H, d, *J*=8.85 Hz, disubstituted benzene CH), 9.93 (1H, s, NH).

^{13}C-NMR (75 MHz, DMSO-d_6) δ (ppm): 25.3, 31.7, 43.0, 45.7, 55.7, 56.0, 57.6, 62.1, 106.0, 120.0, 123.7, 127.9, 130.3, 132.1, 133.0, 134.7, 139.1, 140.6, 143.0, 159.6, 170.0, 193.6.

HRMS (ESI) (m/z) $[M+H]^+$: $C_{26}H_{33}N_3O_4$ calculated: 452.2544, found: 452.2562.

DOPNALAB

	Item	Value
	Acquired Date&Time	22.08.2019 13:36:18
	Acquired by	System Administrator
	Filename	C:\Users\dopnalab\Desktop\NURPELİN\DOKTORA TEZ\D391.ispd
	Spectrum name	D391
	Sample name	D39
	Sample ID	
	Option	
	Comment	
	No. of Scans	50
	Resolution	4 [cm-1]
	Apodization	Happ-Genzel

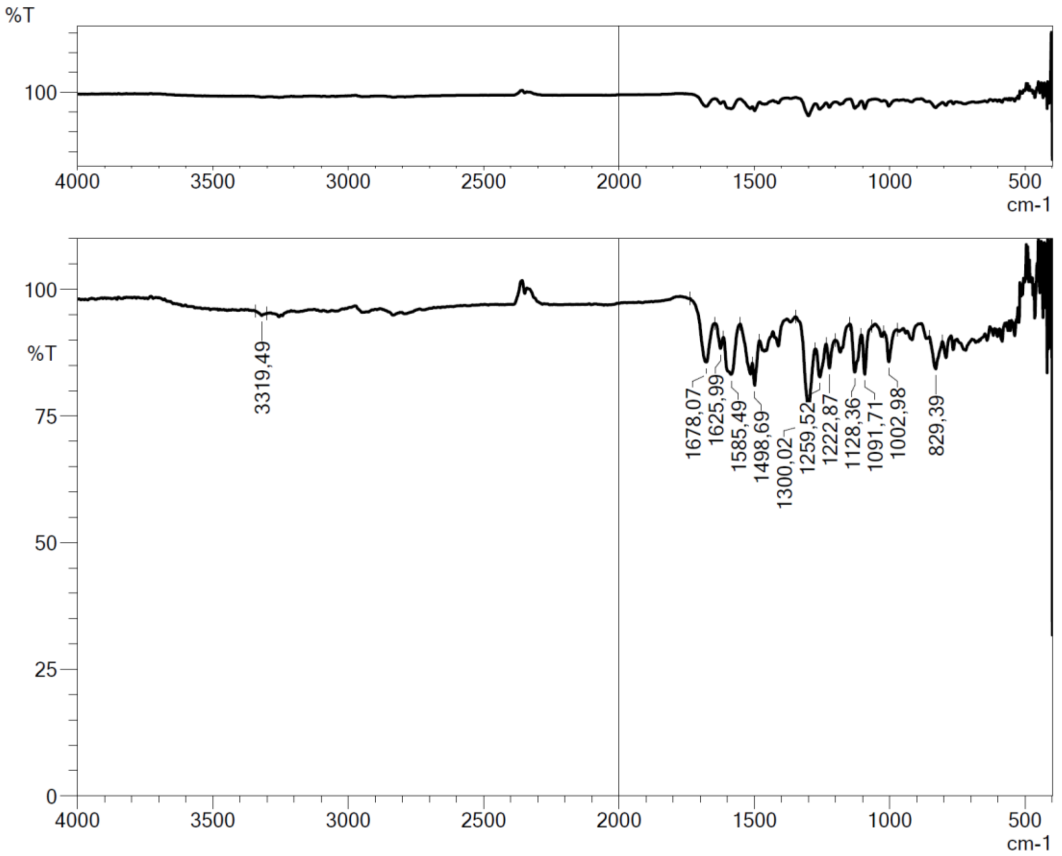

Figure S205. Compound **D39** IR report.

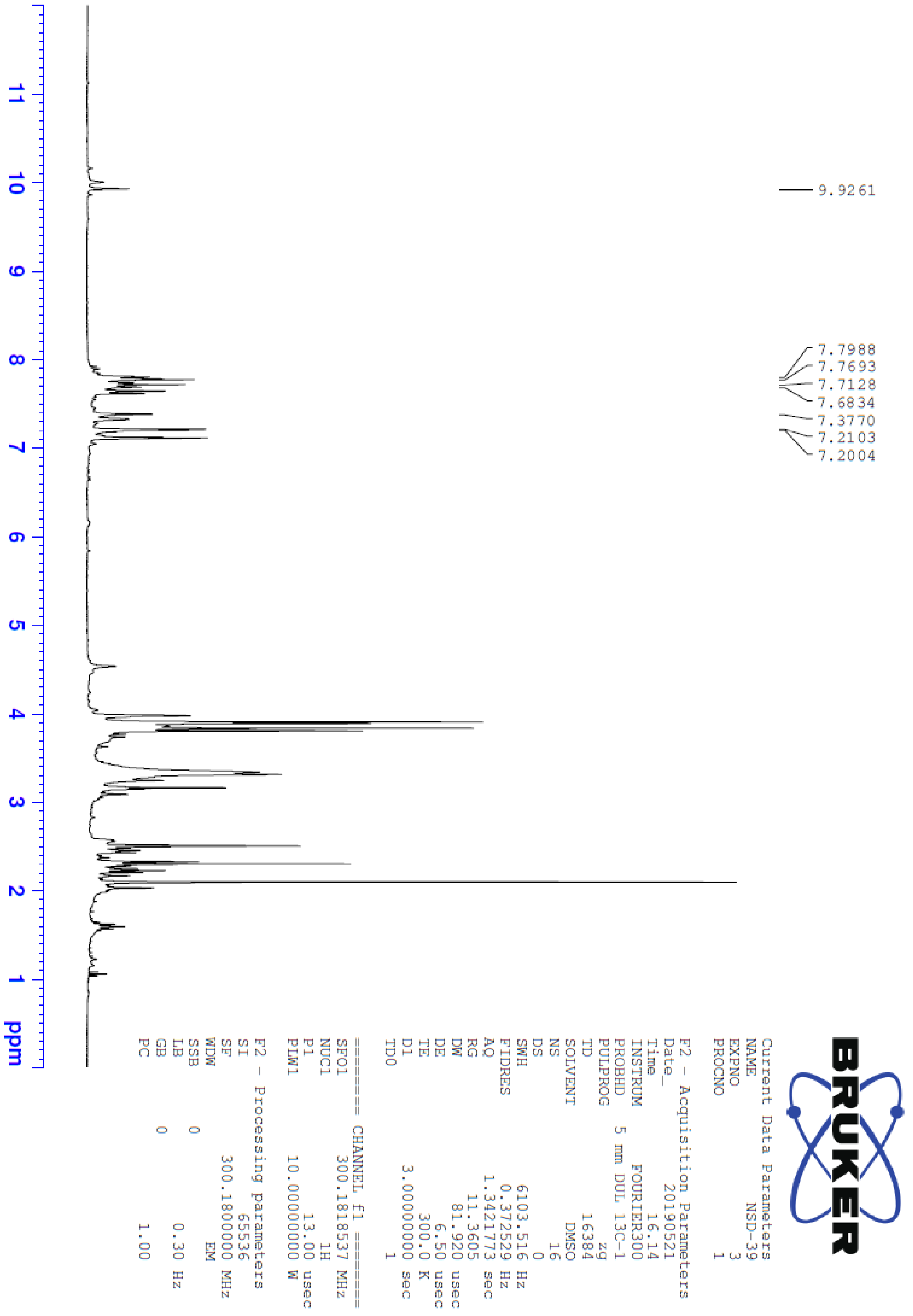

Figure S206. Compound **D39** ^{1}H-NMR spectrum.

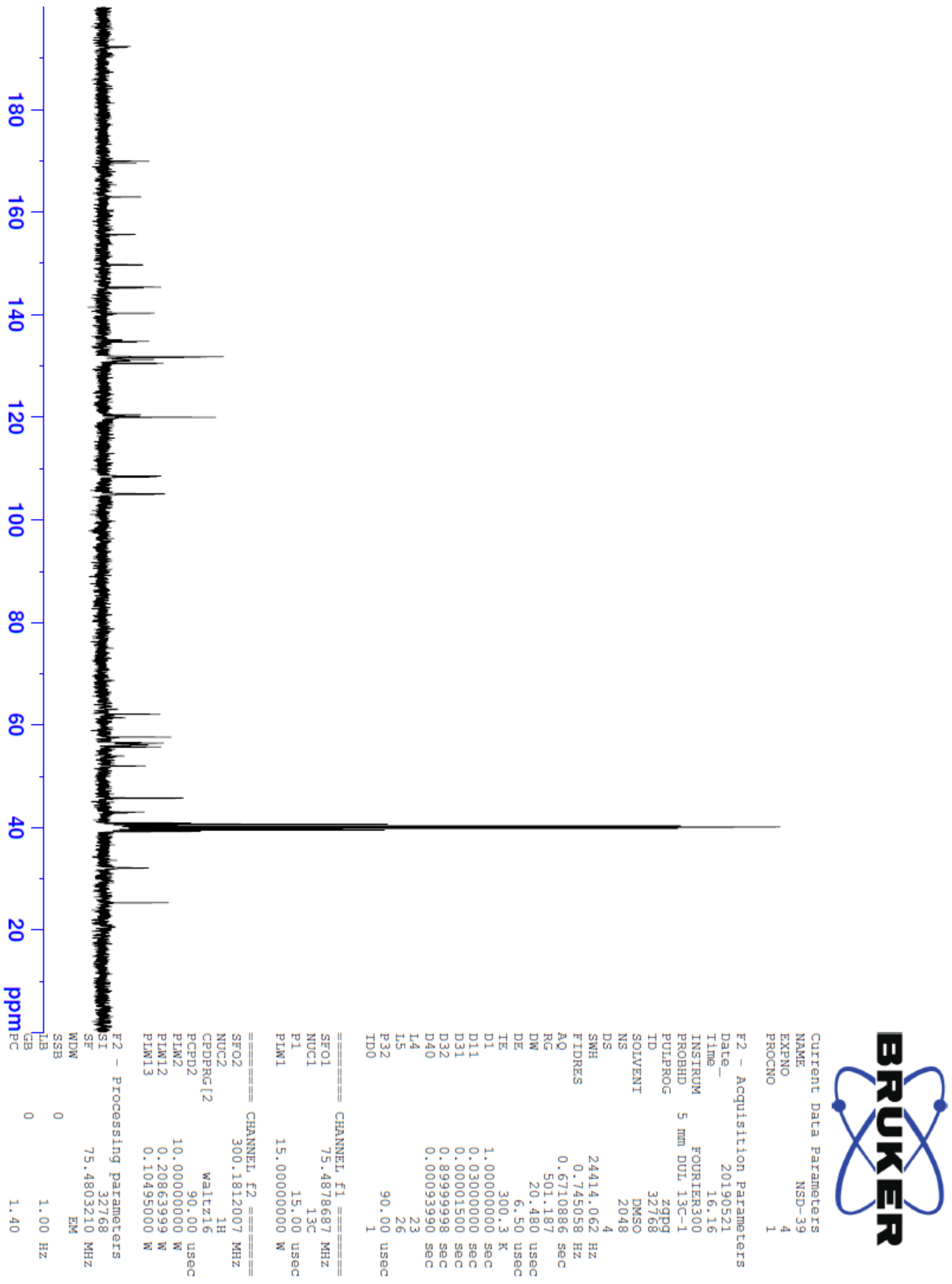

Figure S207. Compound **D39** ^{13}C-NMR spectrum.

Data File: C:\LabSolutions\Data\Analiz\bns\NSD-39_10.lcd

Elmt	Val.	Min	Max	Elmt	Val.	Min	Max	Elmt	Val.	Min	Max	Elmt	Val.	Min	Max	Use Adduct
H	1	5	40	O	2	3	5	S	2	0	0	Ru	2	0	0	H
C	4	0	35	F	1	0	0	Cl	1	0	0	I	3	0	0	
N	3	2	6	P	3	0	0	Br	1	0	0					

Error Margin (ppm):	10	DBE Range:	10.0 - 17.0	Electron Ions:	both
HC Ratio:	unlimited	Apply N Rule:	yes	Use MSn Info:	yes
Max Isotopes:	3	Isotope RI (%):	1.00	Isotope Res:	9000
MSn Iso RI (%):	10.00	MSn Logic Mode:	AND	Max Results:	500

Event#: 1 MS(E+) Ret. Time : 4.320 -> 4.413 Scan# : 649 -> 663

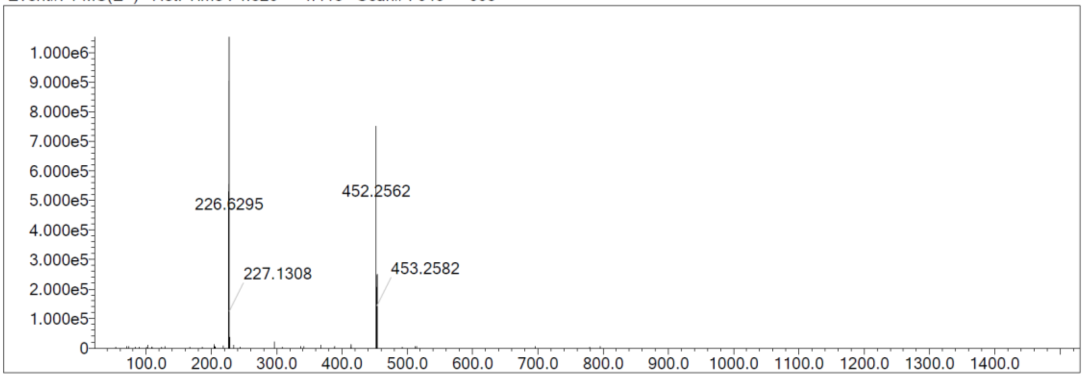

Measured region for 452.2562 m/z

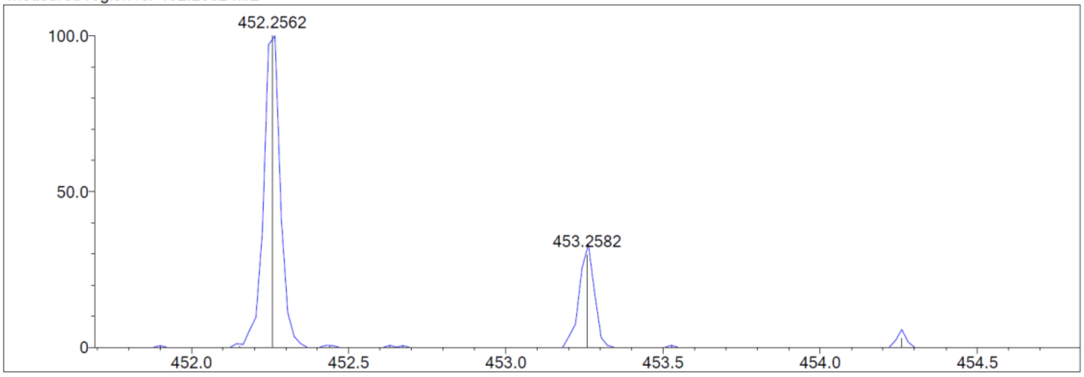

C26 H33 N3 O4 [M+H]+ : Predicted region for 452.2544 m/z

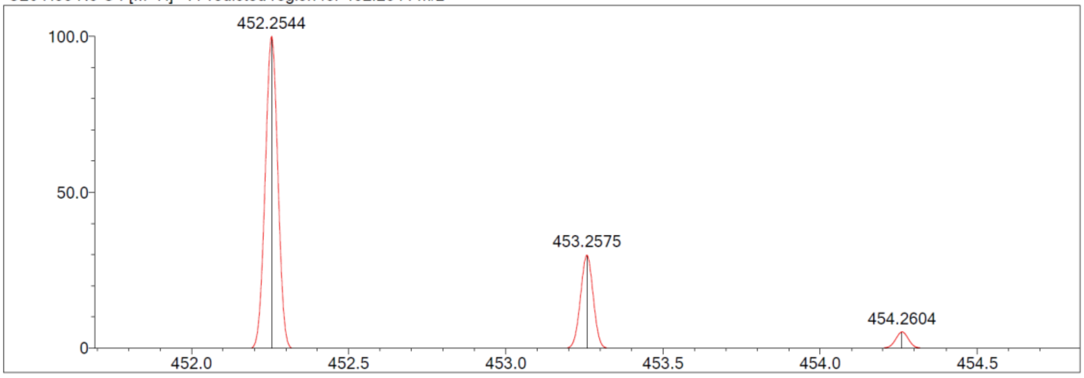

Rank	Score	Formula (M)	Ion	Meas. m/z	Pred. m/z	Df. (mDa)	Df. (ppm)	Iso	DBE
1	92.55	C26 H33 N3 O4	[M+H]+	452.2562	452.2544	1.8	3.98	100.00	12.0

Figure S208. Compound **D39** HRMS report.

*2-(N-ethyl-N-(2-(diethylamino)ethyl)amino)-N-(4-((5-methoxy-1-oxo-2,3-dihydro-1H-inden-2-ylidene)methyl)phenyl)acetamide (**D40**)*

Dark brown liquid. M.P.: 152.7 °C. Yield: 80%.

IR (ATR) ν_{max} (cm^{-1}): 3224 (N-H), 2821 (aliphatic C-H), 1675 (indanone C=O), 1620 (amide C=O), 1514-1498 (C=C), 1220 (C-N), 1089 (C-O), 831 (1,4-disubstituted benzene).

^{1}H-NMR (300 MHz, DMSO-*d_6*) δ (ppm): 0.95 (6H, t, *J*=7.11 Hz, CH_3), 1.00 (3H, t, *J*=7.02 Hz, CH_3), 2.49-2.65 (10H, m, CH_2), 3.22 (2H, s, CH_2), 3.89 (3H, s, OCH_3), 4.06 (2H, s, CH_2), 7.03 (1H, dd, J_1=8.49 Hz, J_2=2.16 Hz, methoxy-1-oxo-indenylidene CH), 7.18 (1H, *J*=1.86 Hz, methoxy-1-oxo-indenylidene CH), 7.40 (1H, s, C=CH), 7.70-7.73 (5H, m, disubstituted benzene CH, methoxy-1-oxo-indenylidene CH), 10.37 (1H, s, NH).

^{13}C-NMR (75 MHz, DMSO-*d_6*) δ (ppm): 11.7, 12.5, 32.5, 47.2, 49.4, 50.4, 52.9, 56.3, 58.3, 110.6, 115.8, 119.5, 125.8, 130.4, 131.2, 131.7, 132.0, 134.5, 140.2, 153.3, 165.3, 171.4, 192.1.

HRMS (ESI) (m/z) $[M+H]^+$: $C_{27}H_{35}N_3O_3$ calculated: 450.2751, found: 450.2763.

DOPNALAB

	Item	Value
	Acquired Date&Time	22.08.2019 13:40:47
	Acquired by	System Administrator
	Filename	C:\Users\dopnalab\Desktop\NURPELİN\DOKTORA TEZ\D401.ispd
	Spectrum name	D401
	Sample name	D40
	Sample ID	
	Option	
	Comment	
	No. of Scans	50
	Resolution	4 [cm-1]
	Apodization	Happ-Genzel

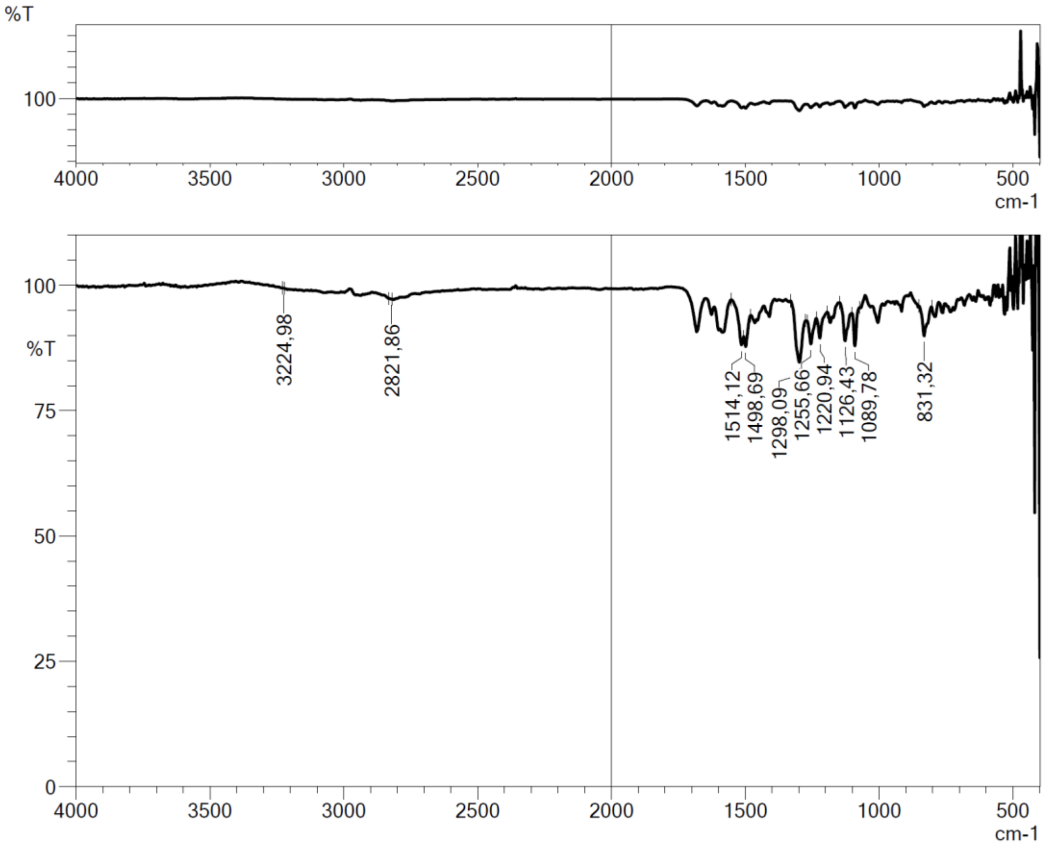

Figure S209. Compound **D40** IR report.

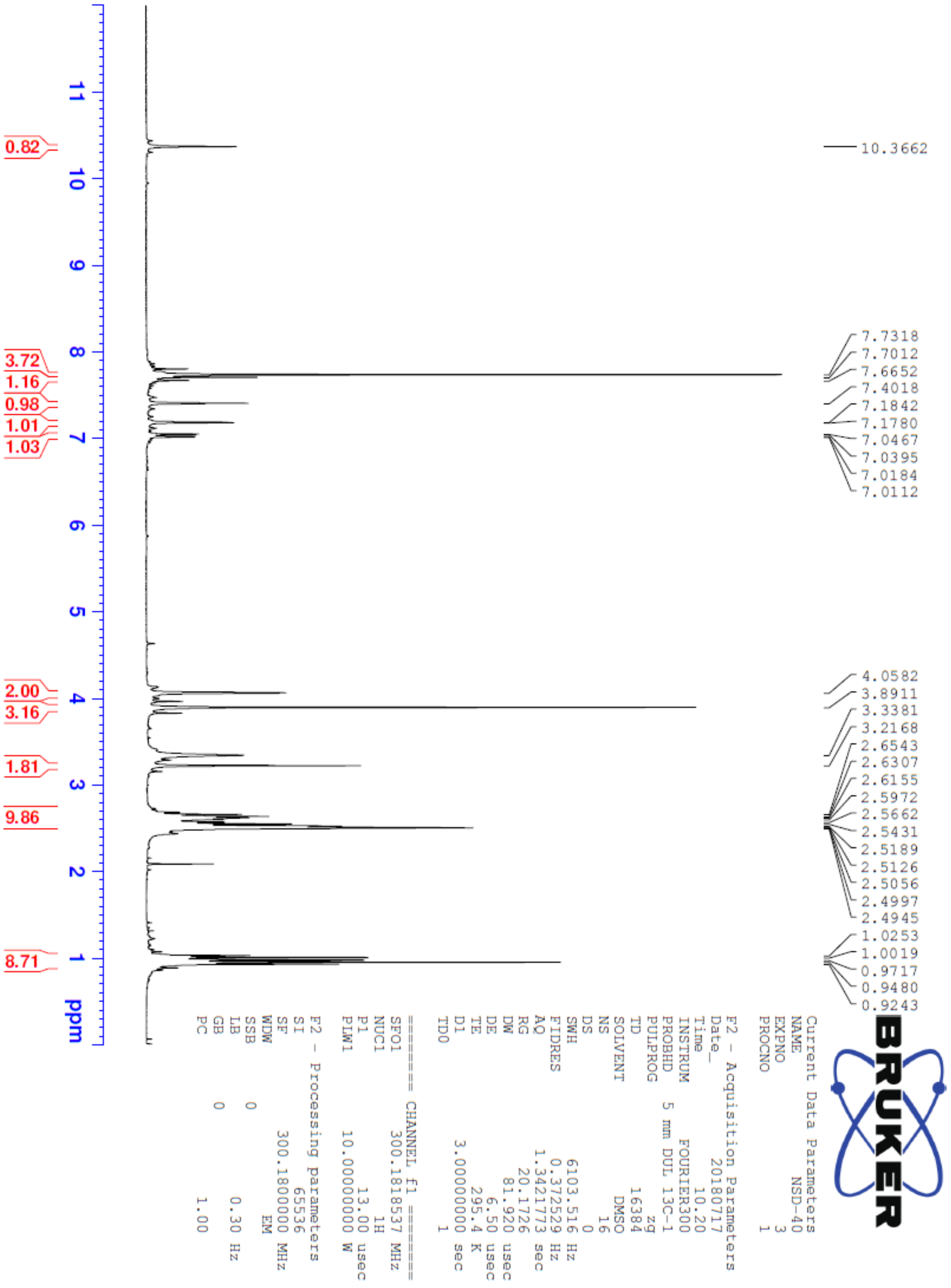

Figure S210. Compound **D40** ^{1}H-NMR spectrum.

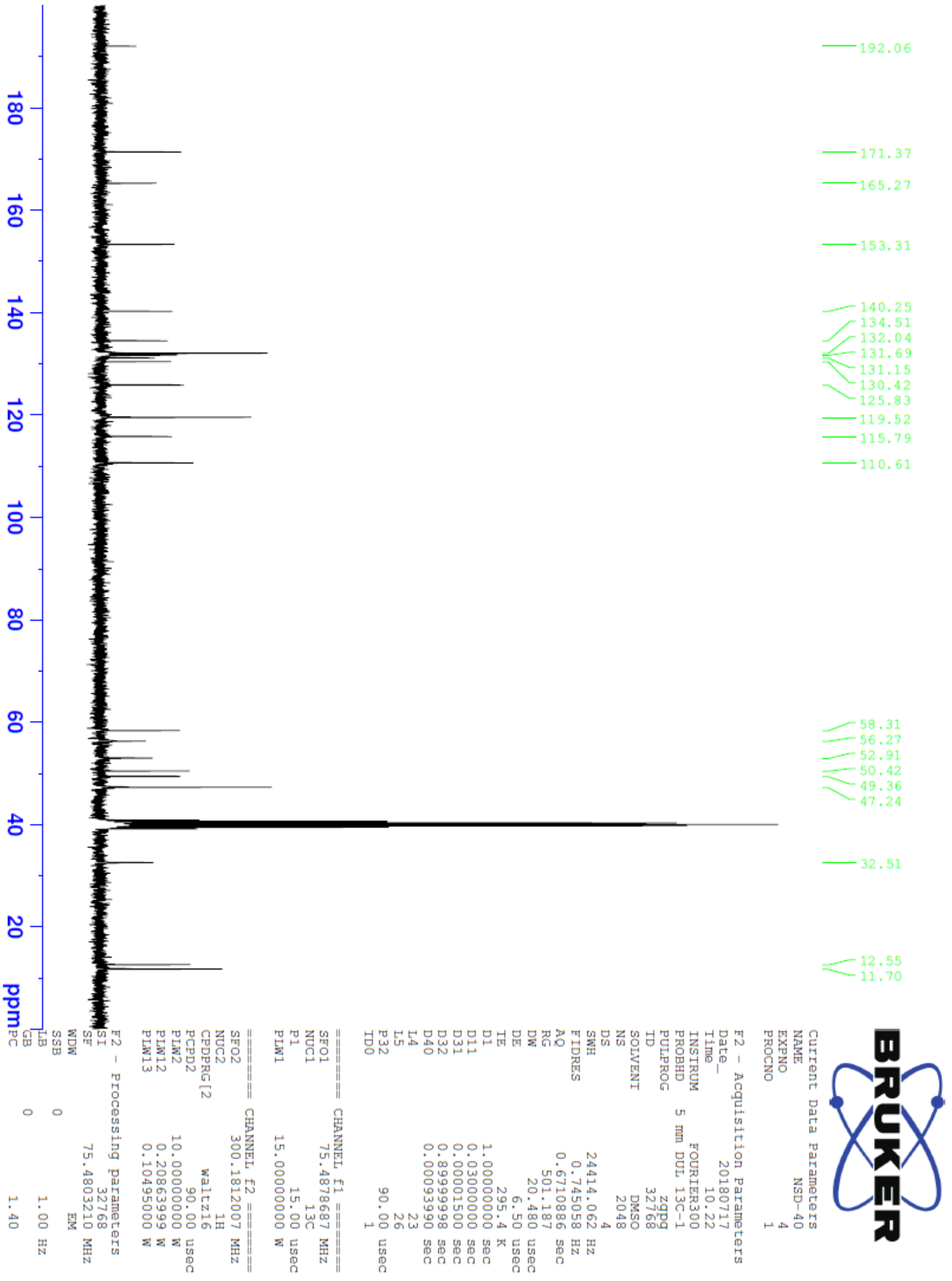

Figure S211. Compound **D40** ^{13}C-NMR spectrum.

Data File: C:\LabSolutions\Data\Analiz\bns\NSD-40_11.lcd

Elmt	Val.	Min	Max	Elmt	Val.	Min	Max	Elmt	Val.	Min	Max	Elmt	Val.	Min	Max	Use Adduct
H	1	5	40	O	2	3	5	S	2	0	0	Ru	2	0	0	H
C	4	0	35	F	1	0	0	Cl	1	0	0	I	3	0	0	
N	3	2	6	P	3	0	0	Br	1	0	0					

Error Margin (ppm): 10
HC Ratio: unlimited
Max Isotopes: 3
MSn Iso RI (%): 10.00

DBE Range: 10.0 - 17.0
Apply N Rule: yes
Isotope RI (%): 1.00
MSn Logic Mode: AND

Electron Ions: both
Use MSn Info: yes
Isotope Res: 9000
Max Results: 500

Event#: 1 MS(E+) Ret. Time : 5.733 -> 5.933 Scan# : 861 -> 891

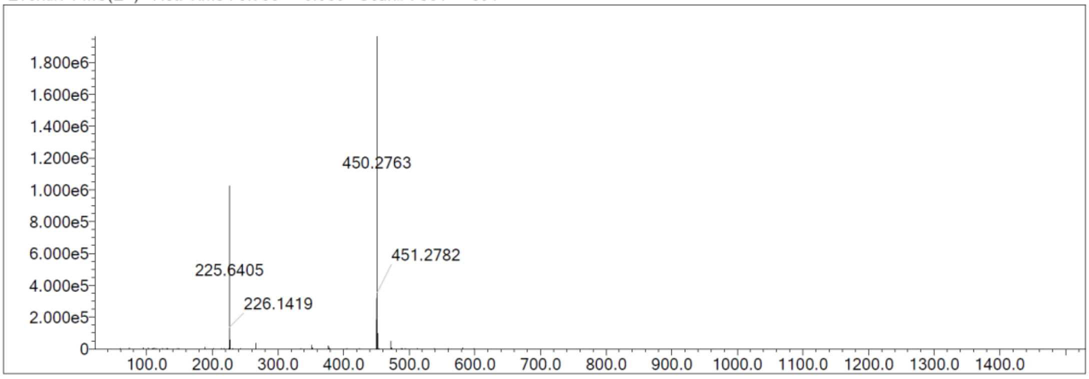

Measured region for 450.2763 m/z

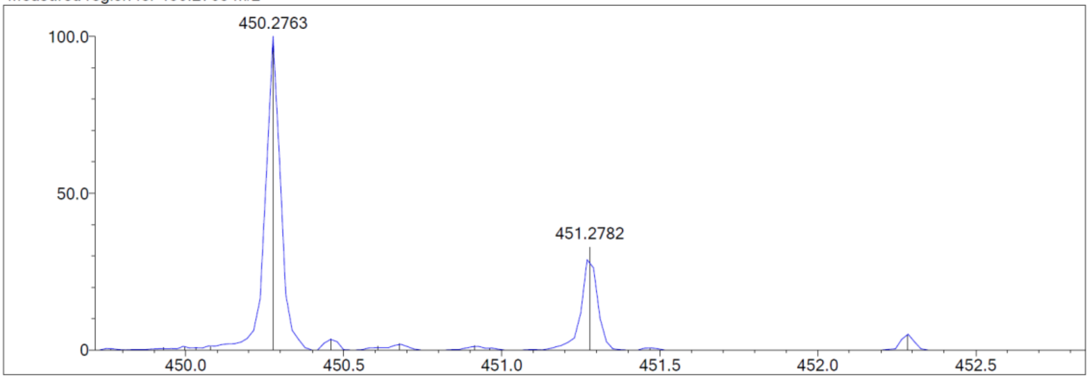

C27 H35 N3 O3 [M+H]+ : Predicted region for 450.2751 m/z

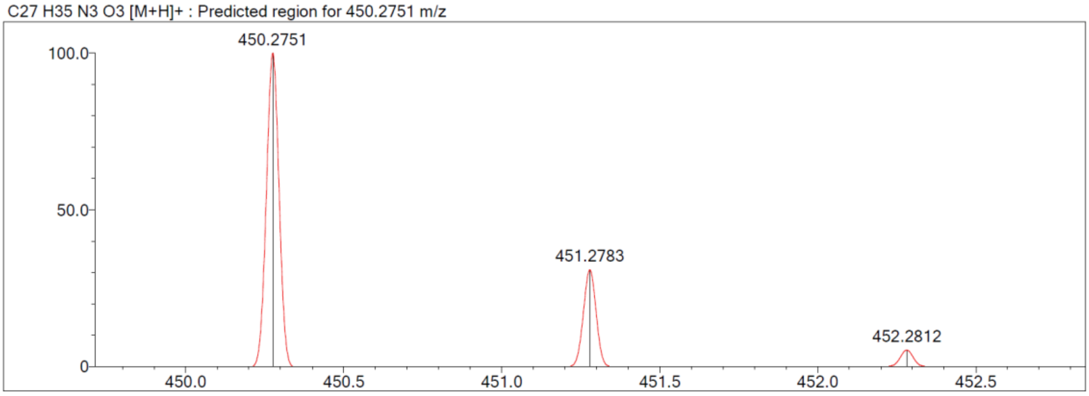

Rank	Score	Formula (M)	Ion	Meas. m/z	Pred. m/z	Df. (mDa)	Df. (ppm)	Iso	DBE
1	95.83	C27 H35 N3 O3	[M+H]+	450.2763	450.2751	1.2	2.67	100.00	12.0

Figure S212. Compound **D40** HRMS report.

*2-(N-ethyl-N-(2-(diethylamino)ethyl)amino)-N-(4-((6-methoxy-1-oxo-2,3-dihydro-1H-inden-2-ylidene)methyl)phenyl)acetamide (**D41**)*

Dark brown liquid. M.P.: 130.5 °C. Yield: 76%.

IR (ATR) ν_{max} (cm^{-1}): 3336 (N-H), 2935 (aliphatic C-H), 1681 (indanone C=O), 1625 (amide C=O), 1583-1498 (C=C), 1220 (C-N), 1089 (C-O), 831 (1,4-disubstituted benzene).

^{1}H-NMR (300 MHz, DMSO-*d6*) δ (ppm): 0.95 (6H, t, *J*=7.11 Hz, CH_3), 1.00 (3H, t, *J*=7.02 Hz, CH_3), 2.49-2.65 (10H, m, CH_2), 3.22 (2H, s, CH_2), 3.89 (3H, s, OCH_3), 4.06 (2H, s, CH_2), 7.03 (1H, dd, J_1=8.49 Hz, J_2=2.16 Hz, methoxy-1-oxo-indenylidene CH), 7.18 (1H, *J*=1.86 Hz, methoxy-1-oxo-indenylidene CH), 7.40 (1H, s, C=CH), 7.70-7.73 (5H, m, disubstituted benzene CH, methoxy-1-oxo-indenylidene CH), 10.37 (1H, s, NH).

^{13}C-NMR (75 MHz, DMSO-*d6*) δ (ppm): 11.7, 12.5, 32.5, 47.2, 49.4, 50.4, 52.9, 56.3, 58.3, 110.6, 115.8, 119.5, 125.8, 130.4, 131.2, 131.7, 132.0, 134.5, 140.2, 153.3, 165.3, 171.4, 192.1.

HRMS (ESI) (m/z) $[M+H]^+$: $C_{27}H_{35}N_3O_3$ calculated: 450.2751, found: 450.2763.

DOPNALAB

	Item	Value
	Acquired Date&Time	22.08.2019 13:43:23
	Acquired by	System Administrator
	Filename	C:\Users\dopnalab\Desktop\NURPELİN\DOKTORA TEZ\D411.ispd
	Spectrum name	D411
	Sample name	D41
	Sample ID	
	Option	
	Comment	
	No. of Scans	50
	Resolution	4 [cm-1]
	Apodization	Happ-Genzel

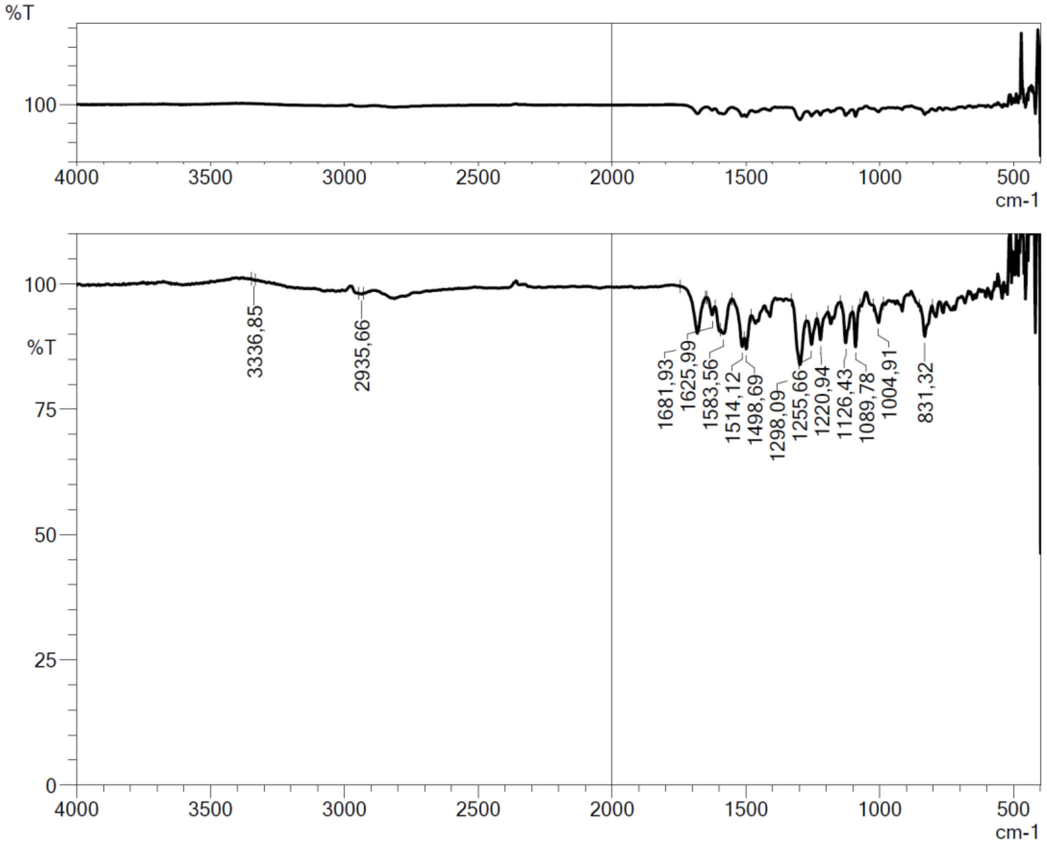

Figure S213. Compound **D41** IR report.

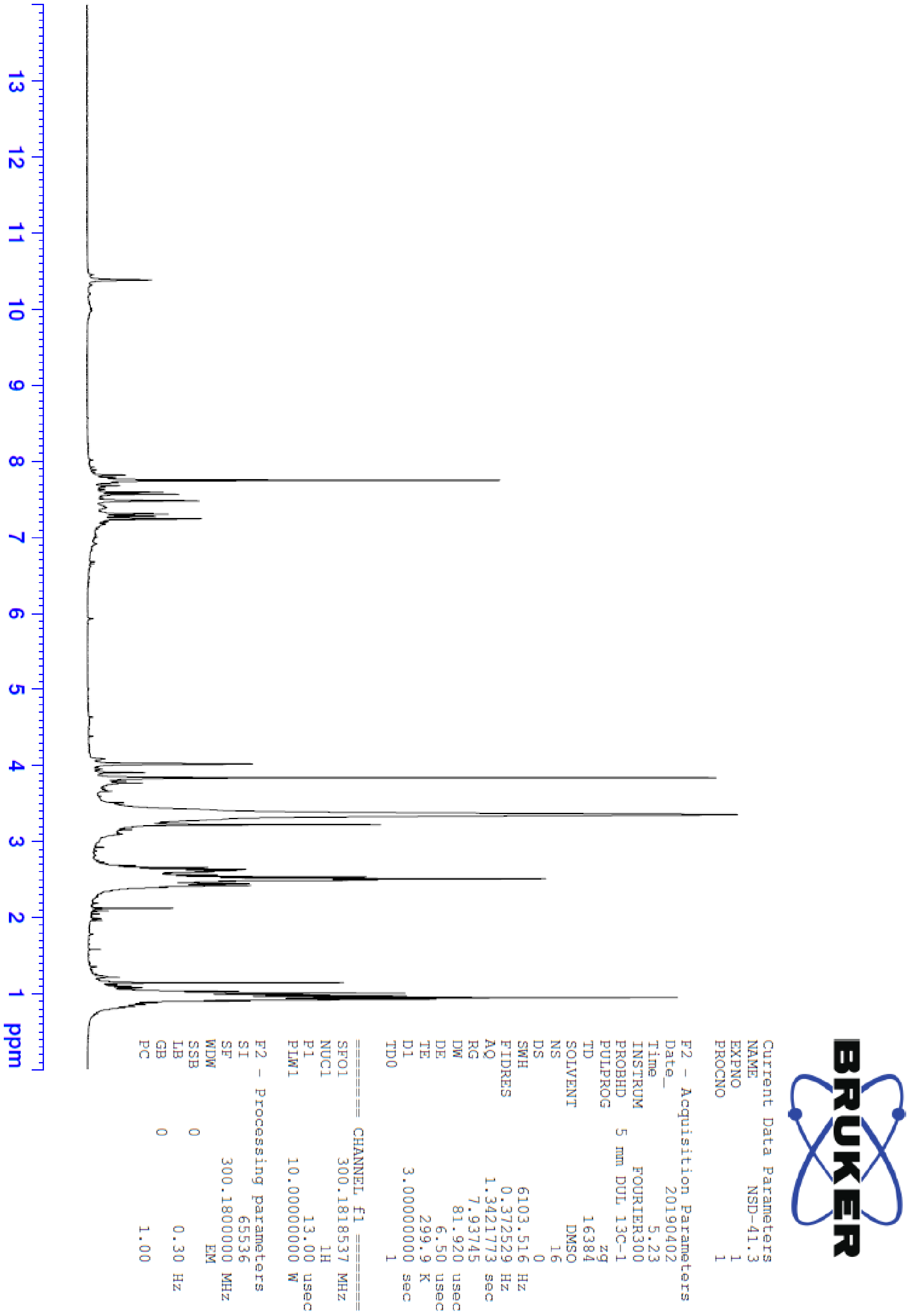

Figure S214. Compound **D41** ^{1}H-NMR spectrum.

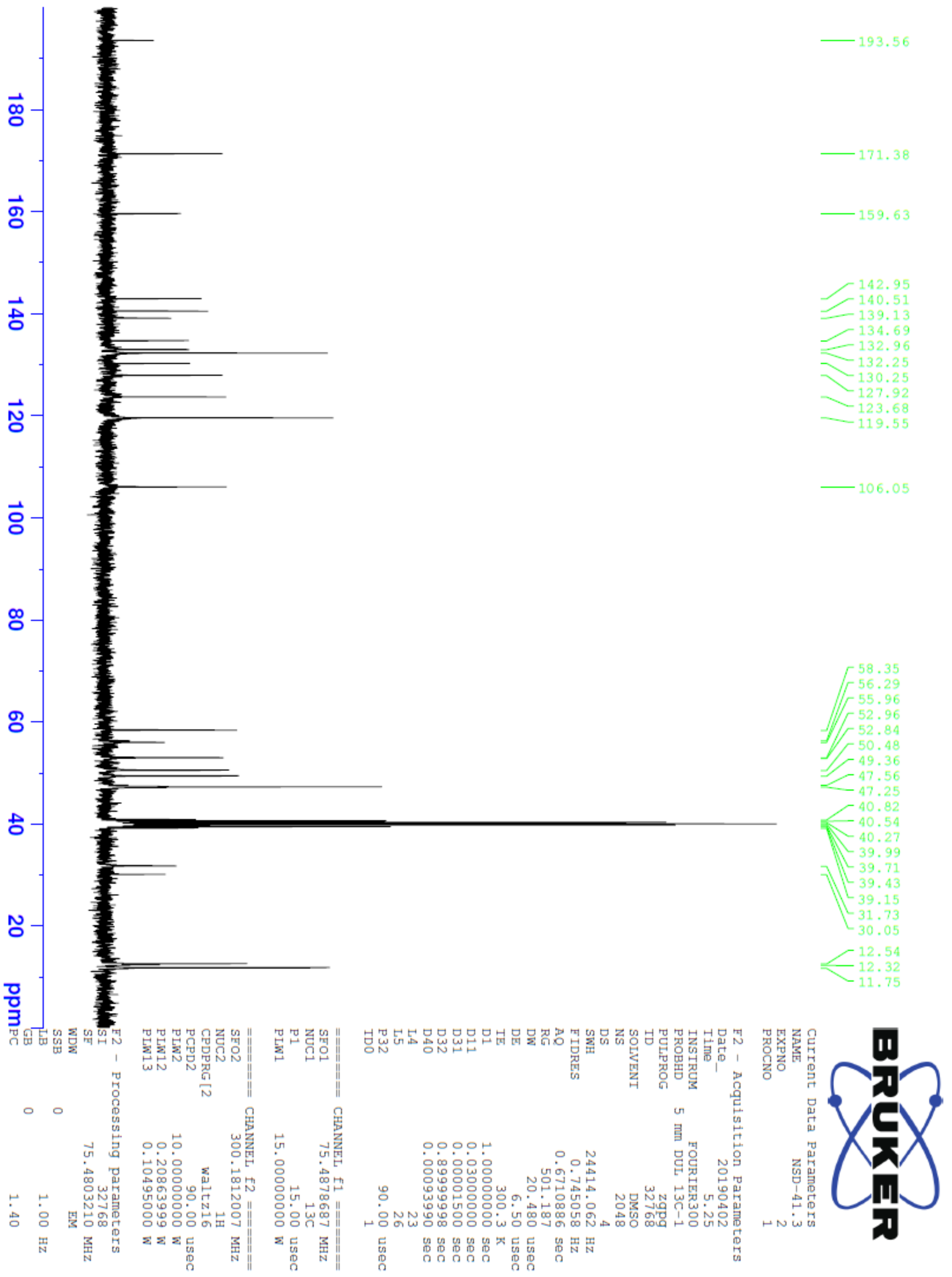

Figure S215. Compound **D41** ^{13}C-NMR spectrum.

Data File: C:\LabSolutions\Data\Analiz\Serkan\NSD-41_76.lcd

Elmt	Val.	Min	Max	Elmt	Val.	Min	Max	Elmt	Val.	Min	Max	Elmt	Val.	Min	Max	Use Adduct
H	1	5	40	O	2	3	5	S	2	0	0	Ru	2	0	0	H
C	4	0	35	F	1	0	0	Cl	1	0	0	I	3	0	0	
N	3	2	6	P	3	0	0	Br	1	0	0					

Error Margin (ppm):	10	DBE Range:	10.0 - 17.0	Electron Ions:	both
HC Ratio:	unlimited	Apply N Rule:	yes	Use MSn Info:	yes
Max Isotopes:	3	Isotope RI (%):	1.00	Isotope Res:	9000
MSn Iso RI (%):	10.00	MSn Logic Mode:	AND	Max Results:	500

Event#: 1 MS(E+) Ret. Time : 2.227 -> 2.720 Scan# : 335 -> 409

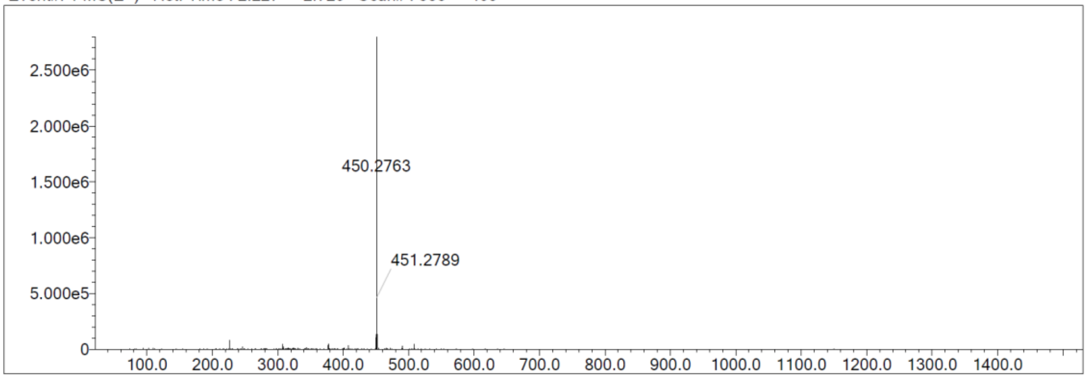

Measured region for 450.2763 m/z

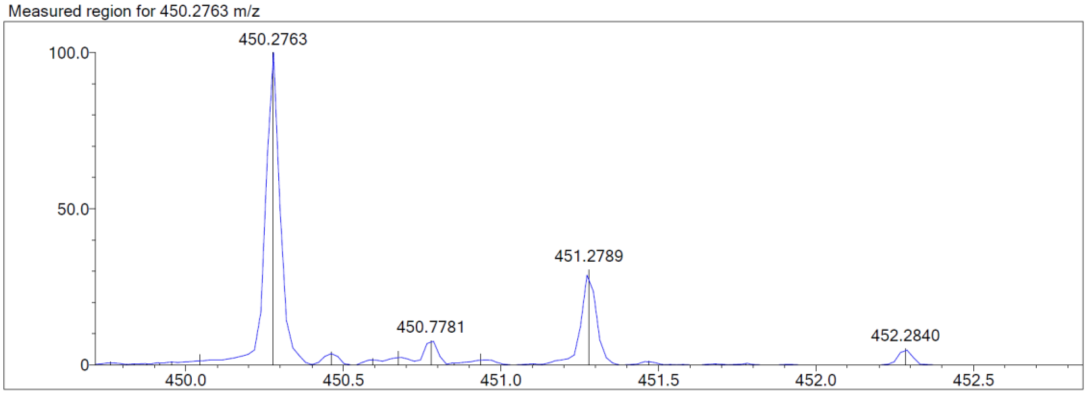

C27 H35 N3 O3 [M+H]+ : Predicted region for 450.2751 m/z

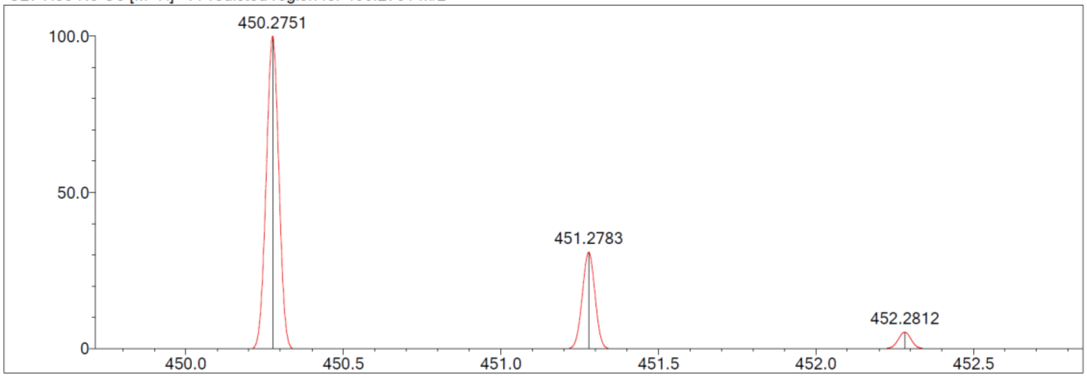

Rank	Score	Formula (M)	Ion	Meas. m/z	Pred. m/z	Df. (mDa)	Df. (ppm)	Iso	DBE
1	84.04	C27 H35 N3 O3	[M+H]+	450.2763	450.2751	1.2	2.67	87.70	12.0

Figure S216. Compound **D41** HRMS report.

*2-(N-ethyl-N-(2-(diethylamino)ethyl)amino)-N-(4-((5,6-dimethoxy-1-oxo-2,3-dihydro-1H-inden-2-ylidene))methyl)phenyl)acetamide (**D42**)*

Yellow powder. M.P.: 154.3 °C. Yield: 78%.

IR (ATR) ν_{max} (cm^{-1}): 3194 (N-H), 2937-2812 (aliphatic C-H), 1681 (indanone C=O), 1585 (amide C=O), 1514-1498 (C=C), 1220 (C-N), 1089 (C-O), 831 (1,4-disubstituted benzene).

^{1}H-NMR (300 MHz, DMSO-d_6) δ (ppm): 0.92 (6H, t, *J*=7.11 Hz, CH_3), 1.00 (3H, t, *J*=7.02 Hz, CH_3), 2.49-2.65 (10H, m, CH_2), 3.21 (2H, s, CH_2), 3.83 (3H, s, OCH_3), 3.90 (3H, s, OCH_3), 3.97 (2H, s, CH_2), 7.19 (1H, s, methoxy-1-oxo-indenylidene CH), 7.20 (1H, s, methoxy-1-oxo-indenylidene CH), 7.37 (1H, s, C=CH), 7.71 (4H, s, disubstituted benzene CH), 10.36 (1H, s, NH).

^{13}C-NMR (75 MHz, DMSO-d_6) δ (ppm): 11.7, 12.5, 32.1, 47.2, 49.4, 50.4, 53.0, 56.1, 56.4, 58.3, 105.0, 108.5, 119.5, 130.5, 130.6, 131.2, 131.9, 134.8, 140.2, 145.4, 149.7, 155.6, 171.3, 192.1.

HRMS (ESI) (m/z) $[M+H]^+$: $C_{28}H_{37}N_3O_4$ calculated: 480.2857, found: 480.2868.

DOPNALAB

	Item	Value
	Acquired Date&Time	22.08.2019 13:45:51
	Acquired by	System Administrator
	Filename	C:\Users\dopnalab\Desktop\NURPELİN\DOKTORA TEZ\D421.ispd
	Spectrum name	D421
	Sample name	D42
	Sample ID	
	Option	
	Comment	
	No. of Scans	50
	Resolution	4 [cm-1]
	Apodization	Happ-Genzel

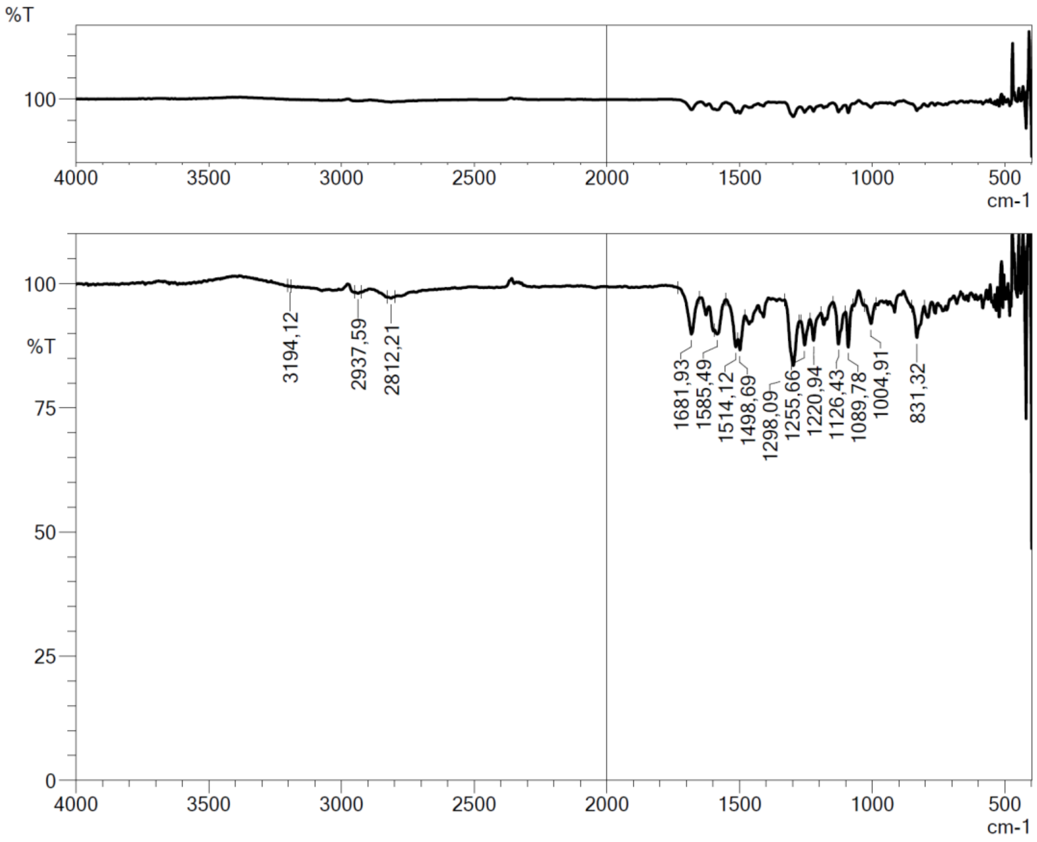

Figure S217. Compound **D42** IR report.

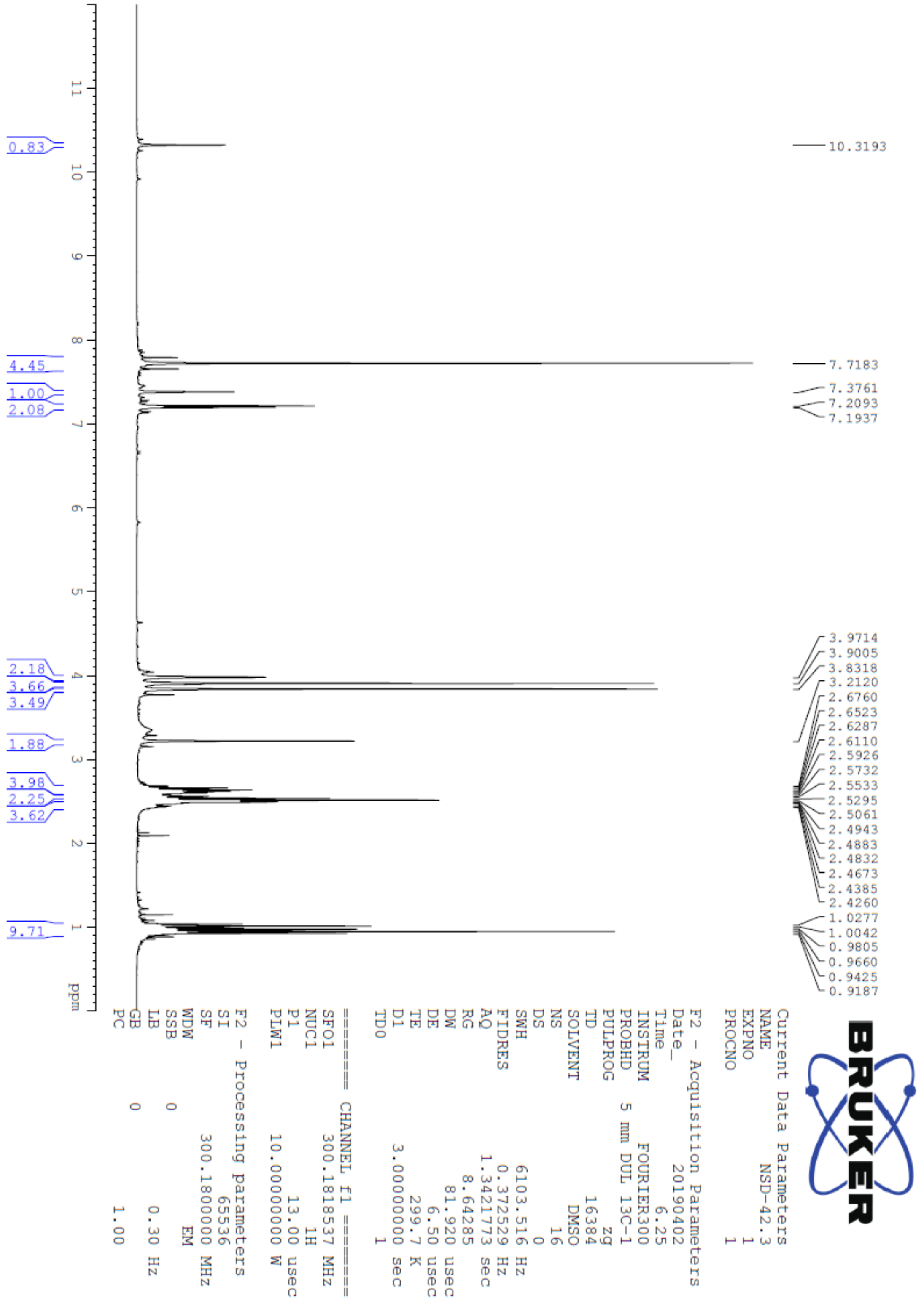

Figure S218. Compound **D42** ^{1}H-NMR spectrum.

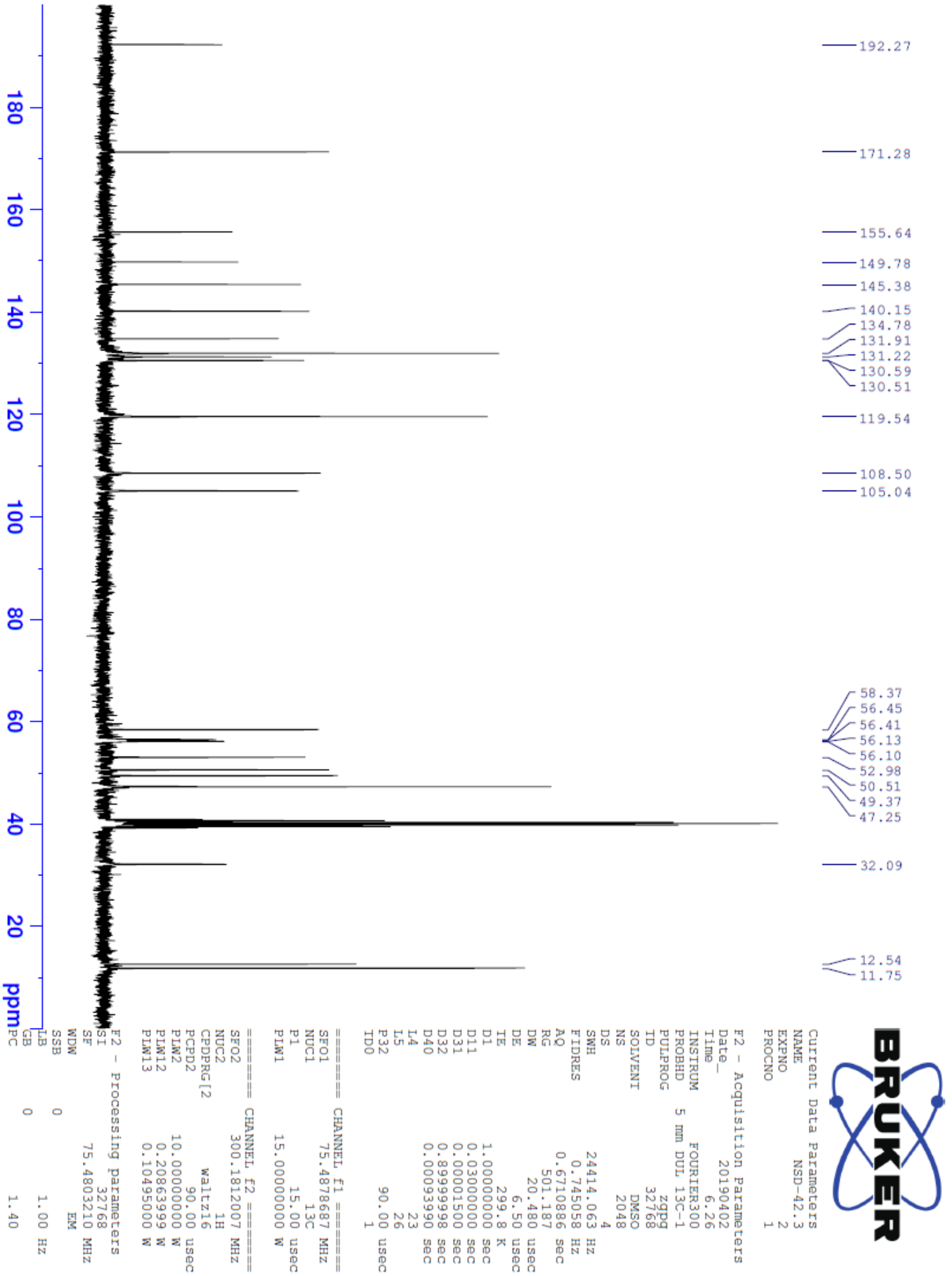

Figure S219. Compound **D42** ^{13}C-NMR spectrum.

Data File: C:\LabSolutions\Data\Analiz\bns\NSD-42_16.lcd

Elmt	Val.	Min	Max	Elmt	Val.	Min	Max	Elmt	Val.	Min	Max	Elmt	Val.	Min	Max	Use Adduct
H	1	5	40	O	2	3	5	S	2	0	0	Ru	2	0	0	H
C	4	0	35	F	1	0	0	Cl	1	0	0	I	3	0	0	
N	3	2	6	P	3	0	0	Br	1	0	0					

Error Margin (ppm):	10	DBE Range:	10.0 - 17.0	Electron Ions:	both
HC Ratio:	unlimited	Apply N Rule:	yes	Use MSn Info:	yes
Max Isotopes:	3	Isotope RI (%):	1.00	Isotope Res:	9000
MSn Iso RI (%):	10.00	MSn Logic Mode:	AND	Max Results:	500

Event#: 1 MS(E+) Ret. Time : 5.507 -> 5.653 Scan# : 827 -> 849

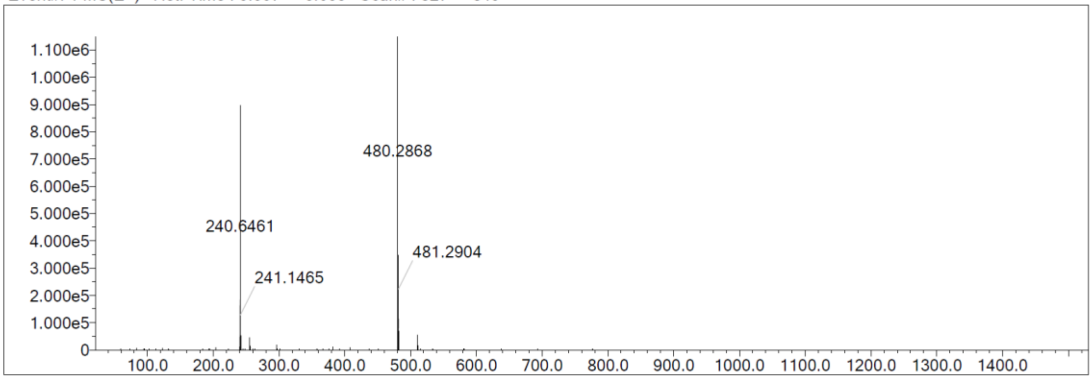

Measured region for 480.2868 m/z

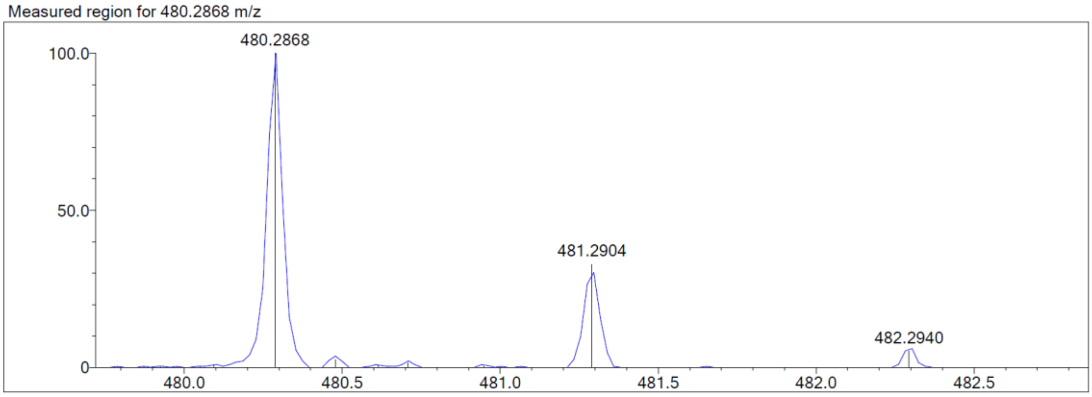

C28 H37 N3 O4 [M+H]+ : Predicted region for 480.2857 m/z

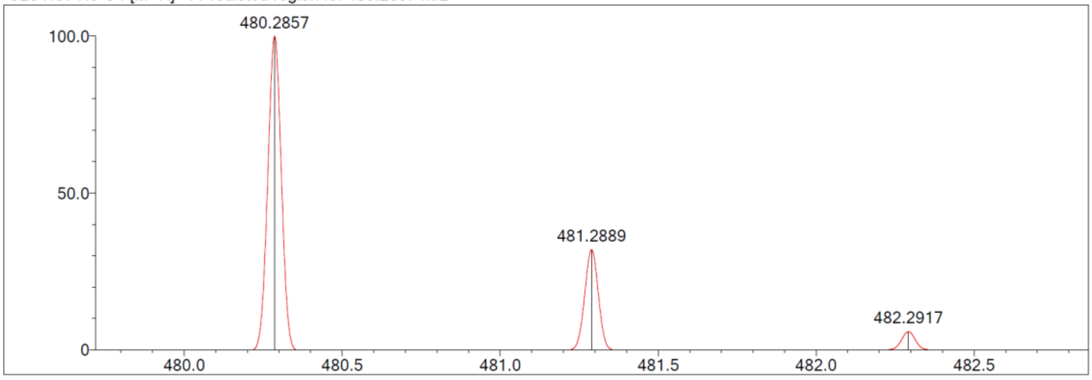

Rank	Score	Formula (M)	Ion	Meas. m/z	Pred. m/z	Df. (mDa)	Df. (ppm)	Iso	DBE
1	86.83	C28 H37 N3 O4	[M+H]+	480.2868	480.2857	1.1	2.29	89.73	12.0

Figure S220. Compound **D42** HRMS report.
